# Supplementary material for: Functional group tolerant hydrogen borrowing C-alkylation
Source: Nat Commun. 2024 Jun 15;15:5131. doi: 10.1038/s41467-024-49249-2 (PMC11180204; doi:10.1038/s41467-024-49249-2)
Supplement: Supplementary file 1 — Supplementary Information [file 41467_2024_49249_MOESM1_ESM.pdf]

Supplementary information for

Functional group tolerant hydrogen borrowing C-alkylation

Elliot P. Bailey, Timothy J. Donohoe*, Martin D. Smith*

Chemistry Research Laboratory, University of Oxford, 12 Mansfield Road, Oxford, OX1 3TA, UK.

* To whom correspondence should be addressed:

timothy.donohoe@chem.ox.ac.uk; homepage: http://donohoe.chem.ox.ac.uk

martin.smith@chem.ox.ac.uk; homepage: http://msmith.chem.ox.ac.uk/

Supplementary Methods

General Experimental Details 3

General Procedures 5

Synthetic Procedures and Characterization Data 7

Discovery and Optimization of Reaction at 23 °C 7

Scope of Reaction with Ph* Methyl Ketone at 23 °C 10

Unsuccessful Alcohol Substrates at 23 °C (which are not exemplified at 85 °C) 30

Scope of Reaction with Ph* Methyl Ketone at 85 °C 31

Unsuccessful Alcohol Substrates at 85 °C 45

Optimization of Reaction with Acetophenone at 23 °C 46

Scope of Reaction with Diverse Ketones at 23 °C, 85 °C and 115 °C 47

Unsuccessful Ketone Substrates 60

Derivatization of Hydrogen Borrowing Products 61

Unsuccessful Ph* Derivatizations 67

Scale-Up of the Reaction at 23 °C including Ph* Derivatization 68

Mechanistic Studies 70

Observation of Iridium Hydrides 70

Effect of Gas Identity in Reaction Headspace 79

Deactivation of Catalyst Before Degassing Reaction 80

NMR Spectra 82

Supplementary References 182

General Experimental Details

Compounds. Unless otherwise stated, all reagents were purchased from major commercial suppliers (Sigma-Aldrich, Merck, Fluorochem, Apollo Scientific, Fischer Scientific, Tokyo Chemical Industry, Acros Organics, Alfa Aesar) and used without further purification.

Compound Naming. Systematic compound names were generated using ChemBioDraw® Ultra version 22 (Perkin Elmer) following IUPAC nomenclature. Numbering used for C and H in NMR spectra follows IUPAC nomenclature as best fits, however can generally be considered as arbitrary.

Solvents and Inert Conditions. Anhydrous tetrahydrofuran (THF) was purified by filtration through activated alumina columns employing the method of Grubbs *et al.* Water was purified with an Elix® UV-10 system. Other solvents and reagents were used directly as received from commercial suppliers, unless otherwise state. Procedures using moisture-sensitive materials were performed with anhydrous solvents (*vide infra*) under an atmosphere of anhydrous nitrogen in flame-dried flasks, using standard Schlenk techniques. Characterisation of iridium hydride species necessitated the use of oxygen free solvent, achieved by the freeze-pump-thaw method.

Glovebox. Characterisation of highly air sensitive iridium hydride complexes was achieved using a Belle Technology Ltd Glovebox. This apparatus maintained an atmosphere of <1 ppm oxygen and moisture.

Chromatography. Analytical thin-layer chromatography was performed on Merck Kieselgel 60 F254 0.25 mm pre-coated aluminium plates and visualised using a combination of UV light (254 nm), aq. basic potassium permanganate or phosphomolybdic acid (PMA) stains. The removal of solvents *in vacuo* was achieved using a Büchi rotary evaporator with a diaphragm pump (15 mmHg) at bath temperatures up to 40 ˚C. Flash column chromatography was carried out using Apollo Scientific silica gel 60 (0.040–0.063 nm), Merck 60 Å silica gel, VWR (40-63 μm) silica gel and Sigma Aldrich silica gel. On occasion 'fine silica', Fluorochem silica gel 60A (0.020–0.045 nm), was used which is noted for each compound for which this was necessary. Pressure was applied at the column head *via* a flow of nitrogen with the solvent system used in parentheses.

Reaction Temperature. Reactions at −78 °C were performed using a Julabo FT902 immersion cooler. Reactions at 0 °C were performed using an ice-water bath. Reactions at 23 °C were performed at ambient temperature in a fume hood. Reactions >30 °C were performed using a silicone oil bath equipped with stir bar.

Nuclear Magnetic Resonance Spectroscopy. Nuclear magnetic resonance (NMR) spectra were acquired at 298 K on Bruker Avance spectrometers and referenced to residual non-deuterated solvent

signals. This includes Bruker Avance III HD nanobay NMR equipped with a 9.4T magnet (^{1}H, 400 MHz; ^{13}C, 101 MHz; ^{19}F, 377 MHz), Bruker Avance III HD NMR equipped with a 11.75T magnet (^{1}H, 500 MHz; ^{13}C, 126 MHz; ^{19}F, 471 MHz) and Bruker NEO 600 with broadband helium cryoprobe equipped with a 14.1T magnet (^{1}H, 600 MHz; ^{13}C, 151 MHz). Mnova (version 14) was used for processing and viewing NMR data. Chemical shifts were referenced to residual solvent signals in the spectra. Chemical shifts (δ) are given in ppm and coupling constants (*J*) are quoted to the nearest 0.1 hertz (Hz). ^{1}H NMR spectra are reported as follows: δ / ppm (number of protons, multiplicity, coupling constant J / Hz [where appropriate], atom assignment). Multiplicity is abbreviated as follows: s (singlet), d (doublet), t (triplet), q (quartet), p (pentet) and m (multiplet). CH-Ar refers to an aryl carbon bonded to a proton. C-Ar refers to a quaternary aryl carbon. H-X_a or H-X_b where X is a number, refers to two distinct proton resonances from one carbon atom (or in the case of a ring system, from two equivalent carbon atoms). H-CH_2 refers to a methylene unit. Assignments were made with the assistance of COSY, HSQC, HMBC, H2BC or NOESY NMR spectra. Characterisation of iridium hydride species necessitated the use of J-Young NMR tubes.

Mass Spectrometry. Low-resolution mass spectra (LRMS) were recorded using a Micro Mass LCT Premier Spectrometer under electrospray ionisation (ESI) conditions. High-resolution mass spectrometry (HRMS) was performed on Bruker MicroTOF or Waters Micromass GCT / BioAccord instruments under electrospray ionisation (ESI) or electron ionization (EI) conditions. Methanol and acetonitrile were used as solvents depending on the polarity on the compound of interest.

Infra-red Spectrometry. Fourier Transform Infra-red (FT-IR) spectra were recorded as thin films or solids (as indicated) on a Tensor 27 FT-IR spectrometer equipped with a diamond ATR absorption module. Selected maximum absorbances (ν_{max}) of the most intense peaks are reported (cm^{-1}) in the range 600-4000 cm^{-1}.

Melting Points. Melting points (M.p) were obtained using a Reichert melting point apparatus. Values are given in °C and are uncorrected. The solvent(s) from which the sample was crystallised are given in parentheses.

General Procedures

General Procedure A – Hydrogen Borrowing Reaction with Ph* Methyl Ketone and $[Cp^*IrCl_2]_2$

$[Cp^*IrCl_2]_2$ (2.4 mg, 1 mol%), Ph* methyl ketone (57.1 mg, 0.3 mmol, 1 eq.), an alcohol (if solid, 0.3 mmol, 1 eq.) and powdered potassium *tert*-butoxide (16.8 mg, 0.15 mmol, 0.5 eq.) were added sequentially to a microwave vial equipped with a stir bar. The vial was capped and evacuated and backfilled with nitrogen five times. *tert*-Amyl alcohol (0.1 or 0.3 mL, 3 or 1 M) and an alcohol (if liquid, 1.0 eq.), both nitrogen-sparged for >15 min, were then added sequentially. The reaction mixture was then stirred at 23 °C, 85 °C or 115 °C for 18 h.

N.b. Due to the high concentration of the reaction, vials stirred at 23 °C were placed at the centre of a stir plate, such that the solids remained in contact with the solution for the duration of the reaction.

Modification I: An alternate, operationally simpler and more effective method for setting up the reaction under a nitrogen atmosphere was later used during the development of this reaction.

$[Cp^*IrCl_2]_2$ (2.4 mg, 1 mol%), Ph* methyl ketone (57.1 mg, 0.3 mmol, 1 eq.), an alcohol (if solid, 1 eq.) and powdered potassium *tert*-butoxide (16.8 mg, 0.15 mmol, 0.5 eq.) were added sequentially to a microwave vial equipped with a stir bar*.* *tert*-Amyl alcohol (0.3 mL, 1 M) and an alcohol (if liquid, 1.0 eq.) were added quickly and the vial was capped, and evacuated and backfilled with nitrogen (6 × 10 sec). The reaction mixture was then stirred at 23 °C, 85 °C or 115 °C for 18 h.

The reaction mixture was filtered through a short pad of silica gel (elution with diethyl ether, ethyl acetate or methanol depending on assumed relative polarity of the product) and concentrated *in vacuo.* The resulting oil was redissolved in $CDCl_3$ (2.0 mL) and an NMR standard was added (32 µL 0.3 mmol, 1,1,2,2-tetrachloroethane) to determine yield *via* ^{1}H NMR spectroscopy. Further purification was achieved by silica gel column chromatography (elution conditions stated for each compound).

N.b. For reactions set up under air, the reaction components were combined in a microwave vial equipped with stir bar. Liquids were used directly from their source (and were not air-sparged). For reactions set up under oxygen, liquids were oxygen-sparged (>15 min).

N.b. If alcohol substrate was a viscous liquid (e.g., 1,5-pentanediol or (3-(pyrrolidin-1-ylmethyl)bicyclo[1.1.1]pentan-1-yl)methanol) it was treated as a solid and was added to the reaction as follows. The reaction vial containing the reaction solids was placed on a measuring balance and the substrate was deposited on the wall of the reaction vial *via* a glass pipette. The vial was capped and **General Procedure A** was followed as above.

General Procedure B – Hydrogen Borrowing Reaction with non-Ph* Methyl Ketones and $[Cp^*IrCl_2]_2$

$[Cp^*IrCl_2]_2$ (4.8 mg, 2 mol%), a ketone (if solid, 0.3 mmol, 1 eq.), an alcohol (if solid, 0.3 mmol, 1 eq.), powdered potassium *tert*-butoxide (16.8 mg, 0.15 mmol, 0.5 eq.) were added sequentially to a microwave vial equipped with a stir bar. *tert*-Amyl alcohol (3.0 mL, 0.1 M) and an alcohol (if liquid, 0.3 mmol, 1.0 eq.) were added quickly and the vial was capped, and evacuated and backfilled with nitrogen (6 × 10 sec). The reaction mixture was then stirred at 23 ˚C, 85 ˚C or 115 ˚C for 18 h.

The reaction mixture was then filtered through a short pad of silica gel (elution with diethyl ether, ethyl acetate or methanol depending on assumed relative polarity of the product) and concentrated *in vacuo.* The resulting oil was redissolved in $CDCl_3$ (2.0 mL) and an NMR standard was added (32 μL 0.3 mmol, 1,1,2,2-tetrachloroethane) to determine yield *via* ^1H NMR spectroscopy. Further purification was achieved by silica gel column chromatography (elution conditions stated for each compound).

N.b. For reactions set up under air, the reaction components were combined in a microwave vial equipped with stir bar. Liquids were used directly from their source (and were not air-sparged).

General Procedure C for Ph* Deprotection with HCl

Hydrochloric acid (37% aq. 12 M, 0.130 or 0.260 mL) was added to a solution of substrate (0.1 mmol, 1.0 eq.) in HFIP (0.88 mL, therefore 2 or 4 M HCl in HFIP respectively) in a microwave vial equipped with a stir bar. The vial was capped and the reaction solution was stirred for 16 h at 40 ˚C or 65 ˚C. The product was then purified with or without column chromatography as follows.

Chromatography free purification (substrates containing basic nitrogen functionality which is/are protonated under the reaction conditions)**:** The vial was then cooled to rt and water (2 mL) and diethyl ether (3 mL) were added. The aqueous layer was further washed with diethyl ether (4 × 3 mL) and then concentrated *in vacuo* to give the corresponding carboxylic acid HCl salt of the product.

Purification by column chromatography (otherwise)**:** H_2O (5 mL) was added and the reaction mixture was extracted with $CHCl_3$ (3 × 5 mL); the combined organic extracts were dried using Mg_2SO_4, filtered and concentrated *in vacuo* to yield a crude oil. This oil was purified by silica gel column chromatography (elution conditions stated for each compound).

Synthetic Procedures and Characterization Data

Discovery and Optimization of Reaction at 23 °C

Commentary: A hit on the room temperature reaction between Ph* methyl ketone **1** and 1-butanol was found (**Supplementary Table 1, Entry 1,** 71% yield **S2**). This result was optimised with the aims of observing full conversion of Ph* methyl ketone (**Supplementary Table 1, Entry 17,** 93% yield, 90% isolated yield **S2**).

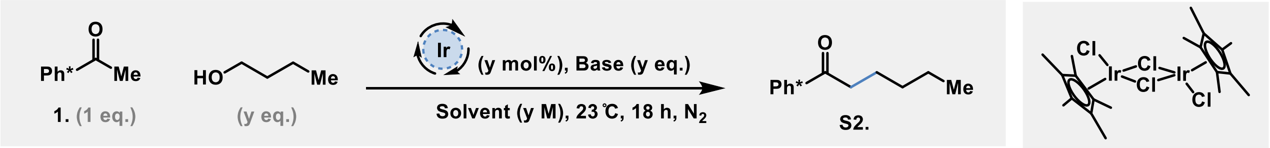

Entry	Ir mol%	1-butanol eq.	Solvent	Base	Recovery 1.	Yield S2.
1	1%	1	2 M 2:1 *tert*-amyl-OH / toluene	KOH (2 eq.)	17%	71%
2	**1.5%**	1	2 M 2:1 *tert*-amyl-OH / toluene	KOH (2 eq.)	38%	37%
3	1%	**1.2**	2 M 2:1 *tert*-amyl-OH / toluene	KOH (2 eq.)	25%	60%
4	1%	1	2 M 2:1 *tert*-amyl-OH / toluene	KOH (**1 eq.**)	38%	55%
5	1%	1	2 M 2:1 *tert*-amyl-OH / toluene	KOH (**0.5 eq.**)	45%	48%
6	1%	1	2 M **1:1** *tert*-amyl-OH / toluene	KOH (2 eq.)	57%	24%
7	1%	1	2 M **1:2** *tert*-amyl-OH / toluene	KOH (2 eq.)	70%	25%
8	1%	1	**2 M *tert*-amyl-OH**	KOH (2 eq.)	11%	80%
9	1%	1	**2.5 M** *tert*-amyl-OH	KOH (2 eq.)	1%	91%
10	1%	1	**3 M** *tert*-amyl-OH	KOH (2 eq.)	1%	91%
11	**0.5%**	1	3 M *tert*-amyl-OH	KOH (2 eq.)	37%	57%
12	1%	1	**3.5 M** *tert*-amyl-OH	KOH (2 eq.)	6%	83%
13	1%	1	**4 M** *tert*-amyl-OH	KOH (2 eq.)	1%	87%
14	1%	1	3 M *tert*-amyl-OH	KO^tBu (**2 eq.**)	83%	4%
15	1%	1	3 M *tert*-amyl-OH	KO^tBu (**1 eq.**)	51%	30%
16	1%	1	3 M *tert*-amyl-OH	KO^tBu (**0.2 eq.**)	42%	50%
17	1%	1	3 M *tert*-amyl-OH	KO^tBu (**0.5 eq.**)	1%	93% (90%)
18[a] (air)	1%	1	3 M *tert*-amyl-OH	KO^tBu (0.5 eq.)	72%	6%
19	1%	1	**1 M** *tert*-amyl-OH	KO^tBu (0.5 eq.)	1%	96%
20[a] (air)	1%	1	**1 M** *tert*-amyl-OH	KO^tBu (0.5 eq.)	85%	0%

Supplementary Table 1. Optimisation of the room temperature hydrogen borrowing reaction using unactivated alcohol *n*-butanol. Yield determined by quantitative ^1H NMR using 1,1,2,2-tetrachlorethane as internal standard, isolated yield is shown in parentheses. [a] Reaction set up under air.

Procedure: Data obtained following **General Procedure A** with individual modifications as detailed per entry, (including **Modification I** as indicated).

Screening of Metal Catalysts and Additives in Room Temperature Hydrogen Borrowing Reaction

Commentary: With the newly found optimised conditions in hand, a screen of other commercially available metal catalysts was undertaken (**Supplementary Table 2**). We were particularly interested whether commercially available metal catalysts with precedent for room temperature hydrogen borrowing reactions, could effect this developed enolate alkylation reaction.[1,2] Other commercially available metal catalysts (and/or, ligand or activator combinations) were chosen due to their general precedent in hydrogen borrowing reactions.[3–5]

It was surprising to find that Ru-MACHO proved an excellent catalyst in this reaction (**Entry 10,** 79% yield). Running the same reaction under air led to no product formation (**Entry 11,** 0% yield **P2**). To the best of our knowledge, there is no precedent for room temperature hydrogen borrowing reactions using Ru-MACHO.

Procedure: Data obtained following **General Procedure A** with different metal catalyst and activators/ligands listed below.

$[Cp^*IrCl_2]_2$ (2.4 mg, 1 mol%), $[Cp^*RhCl_2]_2$ (1.9 mg, 1 mol%), $[Cp^*RuCl]_n$ (1.9 mg, 2 mol%), [Fe] (2.5 mg) + Me_3NO (0.7 mg, 3 mol%), [(*p*-cymene)$RuCl_2]_2$ (1.8 mg, 1 mol%), [Mn] (3.0 mg, 2 mol%), [Ir(cod)Cl_2] (2 mol%) + dPPBz (2.7 mg, 2 mol%), [Ru-MACHO] (3.6 mg, 2 mol%).

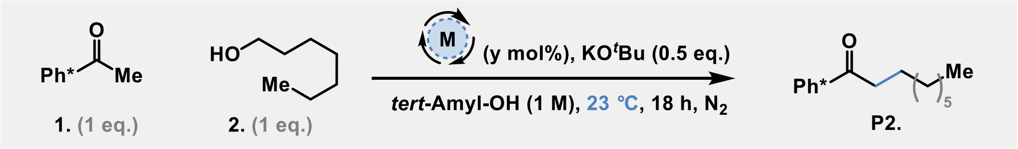

Entry	Catalyst	Catalyst mol%	Additive/Note	Recovery 1.	Recovery 2.	Yield P2.
1	$[Cp^*IrCl_2]_2$	1	-	3%	6%	**91% (90%)**
2[a]	$[Cp^*IrCl_2]_2$	1	Air Atmosphere	76%	60%	**3%**
3[b]	$[Cp^*IrCl_2]_2$	1	Degas Reaction over 1 min	1%	0%	**97%**
4	$[Cp^*RhCl_2]_2$	1	-	78%	74%	**12%**
5	$[Cp^*RuCl_2]n$	2	-	92%	72%	**0%**
6	[Fe]	2	Me_3NO (3 mol%)	75%	94%	**0%**
7	[(*p*-Cymene)$RuCl_2]_2$	1	-	92%	70%	**0%**
8	[Mn]	2	-	94%	78%	**2%**
9	$[IrCodCl_2]$	1	dPPBz (2 mol%)	93%	69%	**0%**
10	Ru-MACHO	2	-	10%	8%	**79%**
11[a]	Ru-MACHO	2	Air atmosphere	96%	95%	**0%**

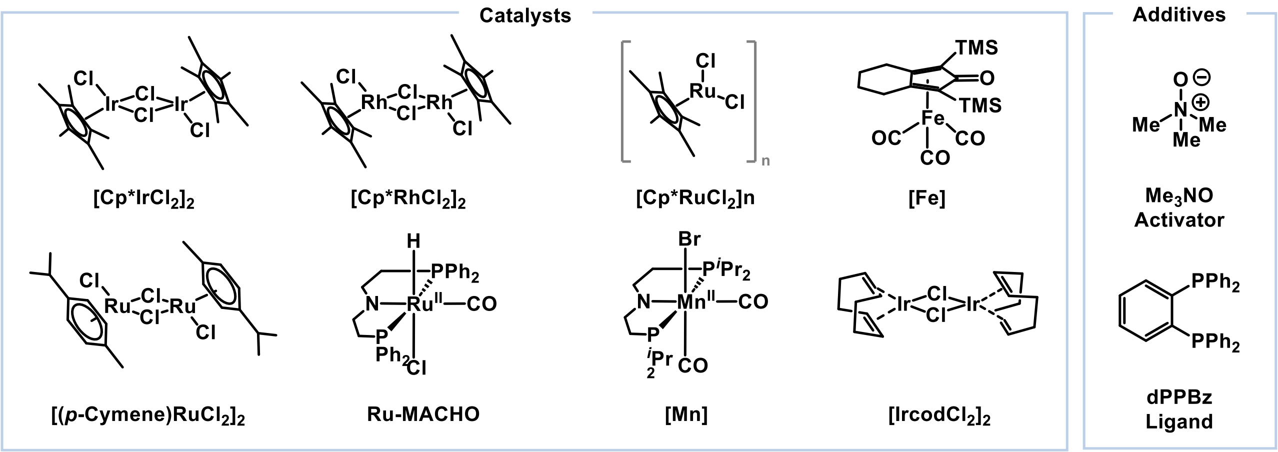

Supplementary Table 2. Catalyst screen with optimised reaction conditions. Yield determined by quantitative ^1H NMR using 1,1,2,2-tetrachlorethane as internal standard, isolated yield is shown in parentheses. [a] Reaction set up under air. [b] Reaction was setup *via* **General Procedure A Modification I.**

Scope of Reaction with Ph* Methyl Ketone at 23 °C

1-(2,3,4,5,6-Pentamethylphenyl)ethan-1-one (Ph* methyl ketone) (1)

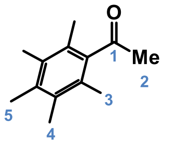

Aluminium trichloride (5.60 g, 42.2 mmol, 1.25 eq.) was added portionwise to a solution of pentamethylbenzene (5.00 g, 33.7 mmol, 1 eq.) and acetyl chloride (2.64 mL, 37.1 mmol, 1.1 eq.) in CH_2Cl_2 (400 mL, 0.08 M) at 0 ˚C. The solution was stirred for 2 h at rt after which TLC (10% diethyl ether in pentane) indicated full consumption of the starting material. The reaction mixture was poured onto ice (~250 g), extracted with CH_2Cl_2 (3 × 30 mL); the combined organic extracts were dried using Mg_2SO_4, filtered and concentrated *in vacuo* to yield a crude colourless solid. This solid was purified by silica gel column chromatography (elution with 5% diethyl ether in pentane) to give the title product as a colourless solid (6.16 g, 96%).

Doubling the scale of the above preparation led to 11.94 g, 93% of product.

This product was also synthesised on 100 g scale.

Pentamethylbenzene (100 g, 674 mmol, 1 eq.), acetyl chloride (52.8 mL, 742 mmol, 1.1 eq.) and CH_2Cl_2 (1000 mL, 0.67 M) were combined in a three neck 2 L round bottom flask equipped with a mechanical stirrer and thermometer. The temperature of the solution was brought down to ~5 ˚C using a large ice bath. Aluminium trichloride (98.9 g, 742 mmol, 1.1 eq.) was added portionwise to this solution over 0.5 h and the reaction mixture was stirred for a further 1 h, after which H_2O (50 mL) and saturated aq. $NaHCO_3$ (50 mL) were added. The reaction mixture was extracted with CH_2Cl_2 (3 × 50 mL); the combined organic extracts were dried using $MgSO_4$, filtered and concentrated *in vacuo*. This crude solid was recrystallised with methanol (50 mL; from 65–23 °C) in a 500 mL round bottom flask. The resulting crystalline solid was filtered and washed with **cold** methanol (50 mL, cooled by surrounding beaker of methanol with dry ice for 30 min), to give the product as a colourless crystalline solid (104 g, 81%).

M.p.: 70–72 °C (from diethyl ether); **LRMS** (ESI^+): 213.0 $[M+Na]^+$; **^{1}H NMR** (400 MHz, $CDCl_3$) δ: 2.46 (3H, s, H-2), 2.24 (3H, s, H-5), 2.19 (6H, s, H-3 or H-4), 2.13 (6H, s, H-4 or H-3); **^{13}C NMR** (101 MHz, $CDCl_3$) δ: 210.2 (C1), 141.1 (C-Ar), 135.5 (C-Ar), 133.2 (C-Ar), 127.1 (C-Ar), 33.3 (C2), 17.2 (C4 or C3), 16.8 (C5), 16.1 (C3 or C4).

The spectral data matched that previously reported in the literature. [6]

1-(2,3,4,5,6-Pentamethylphenyl)hexan-1-one (S2)

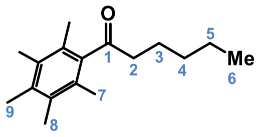

Prepared according to **General Procedure A** with 1-butanol (27.5 μL, 0.3 mmol, 1 eq.) and *tert*-amyl alcohol (0.1 mL, 3 M) at 23 ˚C. The crude product was purified by silica gel column chromatography (elution with 0–2% diethyl ether in pentane) to give the title product as a colourless solid (66.2 mg, 90%).

The title product was also observed in 97% yield by quantitative ^{1}H NMR spectroscopy by following **General Procedure A Modification I** with 1-butanol (27.5 μL, 0.3 mmol, 1 eq.) and *tert*-amyl alcohol (0.1 mL, 3 M) at 23 ˚C.

M.p.: 40–42°C (from diethyl ether); **HRMS** (ESI$^+$) 247.2056 [M+H]$^+$, expected 247.2056; **^{1}H NMR** (400 MHz, $CDCl_3$) δ: 2.67 (2H, t, *J* = 7.5 Hz, H-2), 2.23 (3H, s, H-9), 2.18 (6H, s, H-7 or H-8), 2.10 (6H, s, H-8 or H-7), 1.72 (2H, ddt, *J* = 14.8, 10.0, 5.8 Hz, H-3), 1.41–1.31 (4H, m, H-4, H-5), 0.95–0.88 (3H, m, H-6); **^{13}C NMR** (101 MHz, $CDCl_3$) δ: 212.3 (C1), 141.0 (C-Ar), 135.4 (C-Ar), 133.2 (C-Ar), 127.7 (C-Ar), 45.8 (C2), 31.5 (C3), 23.1 (C4), 22.7 (C5), 17.3 (C7 or C8), 16.8 (C9), 16.1 (C8 or C7), 14.1 (C6); **IR** (solid) (cm^{-1}): 2953, 2919, 1691, 1458, 1379, 1262, 1132, 1099, 1020, 793.

1-(2,3,4,5,6-Pentamethylphenyl)nonan-1-one (P2 or 94)

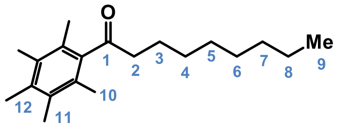

Prepared according to **General Procedure A Modification I** with 1-heptanol (42.6 μL, 0.3 mmol, 1 eq.) and *tert*-amyl alcohol (0.3 mL, 1 M) at 23 ˚C. The crude product was purified by silica gel column chromatography (elution with 2% diethyl ether in pentane) to give the title product as a colourless solid (78.3 mg, 90%).

The title product was also observed in 97% yield by quantitative ^{1}H NMR spectroscopy following **General Procedure A Modification I** with 1-heptanol (42.6 μL, 0.3 mmol, 1 eq.) and *tert*-amyl alcohol (0.3 mL, 1 M) at 23 ˚C.

M.p.: 34–36 °C (from diethyl ether); **HRMS** (ESI$^+$) 289.2522 [M+H]$^+$, expected 289.2526; **^{1}H NMR** (400 MHz, $CDCl_3$) δ: 2.68 (2H, t, *J* = 7.4 Hz, H-2), 2.24 (3H, s, H-12), 2.19 (6H, s, H-10 or H-11), 2.10 (6H, s, H-

11 or H-10), 1.72 (2H, p, J = 7.4 Hz, H-3), 1.40–1.24 (10H, m, H-4, H-5, H-6, H-7, H-8Z), 0.89 (3H, t, J = 6.9 Hz, H-9); **^{13}C NMR** (101 MHz, $CDCl_3$) δ: 212.3 (C1), 141.0 (C-Ar), 135.4 (C-Ar), 133.2 (C-Ar), 127.4 (C-Ar), 45.8 (C2), 32.0 (C7), 29.6 (C5), 29.3 (C6), 29.3 (C4), 23.4 (C3), 22.8 (C8), 17.3 (C11 or C10), 16.8 (C12), 16.1 (C10 or C11), 14.2 (C9); **IR** (solid) (cm^{-1}): 1698, 1468, 1407, 1334, 1309, 1130, 1107, 931, 720, 699.

1-(2,3,4,5,6-Pentamethylphenyl)-3-phenylpropan-1-one (P3)

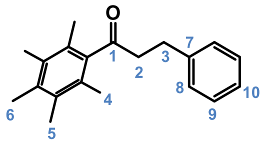

Prepared according to **General Procedure A Modification I** with anhydrous benzyl alcohol (31.0 µL, 0.3 mmol, 1.0 eq.) and *tert*-amyl alcohol (0.1 mL, 3 M) at 23 °C. The crude product was purified by silica gel column chromatography (elution with 0–2% diethyl ether in pentane) to give the title product as a colourless solid (80.6 mg, 96%).

M.p.: 67–69 °C (from diethyl ether); **LRMS** (ESI^+) 303.2 $[M+Na]^+$; **^{1}H NMR** (400 MHz, $CDCl_3$) δ: 7.35–7.13 (5H, m, H-8, H-9, H-10), 3.12–3.03 (2H, m, H-3), 3.00–2.96 (2H, m, H-2), 2.22 (3H, s, H-6), 2.17 (6H, s, H-4 or H-5), 2.03 (6H, s, H-5 or H-4); **^{1}H NMR** (400 MHz, C_6D_6) δ: 7.21–7.09 (4H, m, H-8, H-9), 7.09–7.01 (1H, m, H-10), 3.06 (2H, t, J = 7.5 Hz, H-3), 2.78 (2H, t, J = 7.5 Hz, H-2), 1.99 (3H, s, H-6), 1.91 (6H, s, H-4 or H-5), 1.89 (6H, s, H-5 or H-4); **^{1}H NMR** (400 MHz, CD_3OD) δ: 7.33–7.20 (4H, m, H-8, H-9), 7.20–7.11 (1H, m, H-10), 3.08–2.94 (4H, m, H-2, H-3), 2.21 (3H, s, H-6), 2.15 (6H, s, H-4 or H-5), 1.95 (6H, s, H-5 or H-4); **^{13}C NMR** (101 MHz, $CDCl_3$) δ: 211.0 (C1), 141.1 (C7), 140.6 (C-Ar), 135.5 (C-Ar), 133.2 (C-Ar), 128.6 (C8), 128.6 (C9), 127.5 (C-Ar), 126.2 (C10), 47.2 (C2), 29.5 (C3), 17.2 (C5 or C4), 16.8 (C6), 16.0 (C4 or C5).

The spectral data matched that previously reported in the literature. [5]

4-Methyl-1-(2,3,4,5,6-pentamethylphenyl)hexan-1-one (P4)

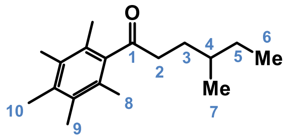

Prepared according to **General Procedure A** with 2-methyl-1-butanol (33.0 µL, 0.3 mmol, 1 eq.) and *tert*-amyl alcohol (0.1 mL, 3 M) at 23 °C. The crude product was purified by silica gel column chromatography (elution with 0–2% diethyl ether in pentane) to give the title product as a colourless solid (68.8 mg, 88%).

M.p.: 50–52 °C (from diethyl ether); **HRMS** (ESI+) 261.2211 [M+H]+, expected 261.2213; **^{1}H NMR** (400 MHz, $CDCl_3$) δ: 2.78–2.58 (2H, m, H-2), 2.24 (3H, s, H-10), 2.20 (6H, s, H-8 or H-9), 2.11 (6H, s, H-9 or H-8), 1.79 (1H, ddt, J = 13.6, 9.8, 5.6 Hz, H-3_a), 1.55 (1H, dddd, J = 13.5, 9.9, 7.4, 5.8 Hz, H-3_b), 1.48–1.31 (2H, m, H-4, H-5_a), 1.27–1.12 (1H, m, H-5_a), 0.94–0.85 (6H, m, H-6, H-7); **^{13}C NMR** (101 MHz, $CDCl_3$) δ: 212.6 (C1), 141.2 (C-Ar), 135.4 (C-Ar), 133.2 (C-Ar), 127.4 (C-Ar), 43.5 (C2), 34.0 (C4), 29.8 (C3), 29.4 (C5), 19.2 (C7), 17.3 (C9 or C8), 16.8 (C10), 16.1 (C8 or C9), 11.4 (C6); **IR** (solid) (cm^{-1}): 2962, 2929, 1699, 1461, 1408, 1379, 1104, 935, 692.

4-Cyclohexyl-1-(2,3,4,5,6-pentamethylphenyl)pentan-1-one (P5)

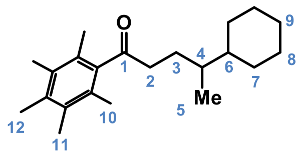

Prepared according to **General Procedure A** with 2-cyclohexylpropan-1-ol (46.7 µL, 0.3 mmol, 1 eq.) and *tert*-amyl alcohol (0.1 mL, 3 M) at 23 ˚C. The crude product was purified by silica gel column chromatography (elution with 0–2% diethyl ether in pentane) to give the title product as a colourless solid (76.6 mg, 81%).

M.p.: 64–66 °C (from diethyl ether); **HRMS** (ESI+) 315.2681 [M+H]+, expected 315.2682; **^{1}H NMR** (400 MHz, $CDCl_3$) δ: 2.79–2.55 (2H, m, H-2), 2.24 (3H, s, H-12), 2.19 (6H, s, H-10 or H-11), 2.11 (6H, s, H-11 or H-10), 1.85 (1H, ddt, J = 13.6, 10.4, 5.2 Hz, H-3_a), 1.78–1.71 (2H, m, H-8_{aa}, H-8_{ba}), 1.70–1.59 (3H, m, H-7_{aa}, H-7_{ba}, H-9_a), 1.58–1.48 (1H, m, H-3_b), 1.35 (1H, dddq, J = 8.7, 6.7, 4.1, 2.1 Hz, H-4), 1.29–0.94 (6H, m, H-6, H-7_{ab}, H-7_{bb}, H-8_{ab}, H-8_{bb}, H-9_b), 0.85 (3H, d, J = 6.8 Hz, H-5); **^{13}C NMR** (101 MHz, $CDCl_3$) δ: 212.6 (C1), 141.2 (C-Ar), 135.4 (C-Ar), 133.2 (C-Ar), 127.4 (C-Ar), 43.9 (C2), 42.8 (C6), 37.7 (C4), 30.8 (C7_a), 28.8 (C7_b), 27.5 (C3), 27.1 (C8_a), 27.0 (C9), 26.9 (C8_b), 17.3 (C11 or C10), 16.8 (C12), 16.1 (C5), 16.1 (C10 or C11); **IR** (solid) (cm^{-1}): 2923, 2852, 1698, 1448, 1408, 1380, 1323, 1117, 934, 695.

7-(Benzyloxy)-1-(2,3,4,5,6-pentamethylphenyl)heptan-1-one (P6)

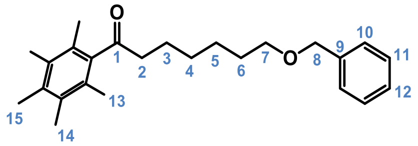

Prepared according to **General Procedure A** with 5-(benzyloxy)pentan-1-ol (57.8 µL, 0.3 mmol, 1 eq.) and *tert*-amyl alcohol (0.1 mL, 3 M) at 23 ˚C. The crude product was purified by silica gel column chromatography (elution with 0–2% diethyl ether in pentane) to give the title product as a colourless solid (98.6 mg, 90%).

M.p.: 38–40 °C (from diethyl ether); **HRMS** (ESI+) 367.2629 [M+H]+, expected 367.2632; **^{1}H NMR** (400 MHz, $CDCl_3$) δ: 7.36 (4H, app d, *J* = 4.4 Hz, H-10, H-11), 7.33–7.26 (1H, m, H-12), 4.52 (2H, s, H-8), 3.49 (2H, t, *J* = 6.5 Hz, H-7), 2.69 (2H, t, *J* = 7.4 Hz, H-2), 2.25 (3H, s, H-15), 2.20 (6H, s, H-13 or H-14), 2.11 (6H, s, H-14 or H-13), 1.82–1.70 (2H, m, H-3), 1.65 (2H, dt, *J* = 8.7, 6.4 Hz, H-6), 1.51–1.35 (4H, m, H-4, H-5); **^{13}C NMR** (101 MHz, $CDCl_3$) δ: 212.1 (C1), 141.0 (C-Ar), 138.8 (C-Ar), 135.4 (C-Ar), 133.1 (C-Ar), 128.5 (C11), 127.7 (C10), 127.6 (C12), 127.4 (C9), 73.0 (C8), 70.5 (C7), 45.7 (C2), 29.7 (C6), 29.1 (C4), 26.2 (C5), 23.3 (C3), 17.3 (C14 or C13), 16.8 (C15), 16.0 (C13 or C14); **IR** (solid) (cm^{-1}): 2932, 2861, 1686, 1415, 1317, 1123, 1099, 1029, 730, 694.

5-((*tert*-Butyldimethylsilyl)oxy)pentan-1-ol (7)

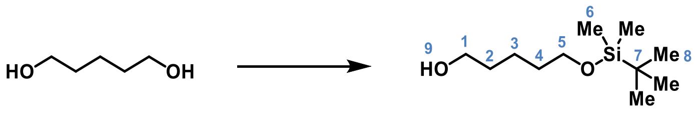

n-BuLi (2.5 M in hexanes, 7.68 mL, 19.2 mmol, 1 eq.) was added dropwise to a solution of pentane-1,5-diol (2.01 mL, 19.2 mmol, 1 eq.) in anhydrous THF (32.0 mL, 0.6 M) in a flame dried flask equipped with a stir bar at −78 ˚C. The reaction mixture was stirred for 0.5 h, after which a solution of *tert*-butyldimethylsilyl chloride (2.89 g, 19.2 mmol, 1 eq.) in anhydrous THF (7.7 mL) was added quickly, and the reaction mixture was stirred for 2 h at rt. The reaction was then quenched with water (20 mL) and the reaction mixture extracted with diethyl ether (3 × 20 mL); the combined organic extracts were dried using Mg_2SO_4, filtered and concentrated *in vacuo* to yield a crude oil. This oil was purified by silica gel column chromatography (elution with 4–10% ethyl acetate in pentane) to give the title product as a colourless oil (1.94 g, 46%).

HRMS (ESI+) 219.1770 [M+H]+, expected 219.1775; **^{1}H NMR** (400 MHz, $CDCl_3$) δ: 3.63 (4H, app dt, *J* = 10.5, 6.5 Hz, H-1, H-5), 1.64–1.48 (5H, m, H-2, H-4, H-9), 1.46–1.33 (2H, m, H-3), 0.89 (9H, s, H-8), 0.04 (6H, s, H-6); **^{13}C NMR** (101 MHz, $CDCl_3$) δ: 63.3 (C5), 63.1 (C1), 32.6 (C2), 32.6 (C4), 26.1 (C8), 22.2 (C3), 18.5 (C7), −5.2 (C6); **ρ** = 0.884 g/mL (23 ˚C).

The spectral data matched that previously reported in the literature.[7]

7-((*tert*-Butyldimethylsilyl)oxy)-1-(2,3,4,5,6-pentamethylphenyl)heptan-1-one (P7)

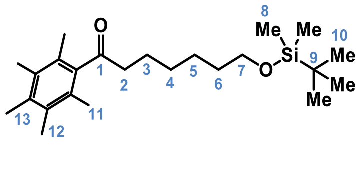

Prepared according to **General Procedure A** with 5-((*tert*-butyldimethylsilyl)oxy)pentan-1-ol (65.5 mg or 74.1 µL, 0.3 mmol, 1 eq.) and *tert*-amyl alcohol (0.1 mL, 3 M) at 23 ˚C. The crude product was purified

by silica gel column chromatography (elution with 0–2% diethyl ether in pentane) to give the title product as a colourless oil (98.2 mg, 84%).

The title product was observed in 26% yield by quantitative ^{1}H NMR spectroscopy following **General Procedure A** with 5-((*tert*-butyldimethylsilyl)oxy)pentan-1-ol (65.5 mg or 74.1 µL, 0.3 mmol, 1 eq.) and *tert*-amyl alcohol (0.1 mL, 3 M) at 85 ˚C**.**

HRMS (ESI^{+}) 391.3017 [M+H]$^{+}$, expected 391.3027; **^{1}H NMR** (400 MHz, $CDCl_3$) δ: 3.61 (2H, t, *J* = 6.5 Hz, H-7), 2.67 (2H, t, *J* = 7.4 Hz, H-2), 2.23 (3H, s, H-13), 2.18 (6H, s, H-11 or H-12), 2.09 (6H, s, H-12 or H-11), 1.78–1.66 (2H, m, H-3), 1.53 (2H, p, *J* = 6.7 Hz, H-6), 1.44–1.32 (4H, m, H-4, H-5), 0.89 (9H, s, H-10), 0.05 (6H, s, H-8); **^{13}C NMR** (101 MHz, $CDCl_3$) δ: 212.3 (C1), 141.0 (C-Ar), 135.4 (C-Ar), 133.2 (C-Ar), 127.4 (C-Ar), 63.4 (C7), 45.7 (C2), 32.9 (C6), 29.2 (C5), 26.1 (C10), 25.9 (C4), 23.4 (C3), 18.5 (C9), 17.3 (C12 or C11), 16.8 (C13), 16.1 (C11 or C12), −5.1 (C8); **IR** (thin film) (cm^{-1}): 1702, 1463, 1255, 1101, 1006, 934, 836, 775, 757, 666.

3-Cyclopropyl-1-(2,3,4,5,6-pentamethylphenyl)propan-1-one (P8)

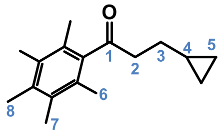

Prepared according to **General Procedure A** with cyclopropanemethanol (24.3 µL, 0.3 mmol, 1 eq.) and *tert*-amyl alcohol (0.1 mL, 3 M) at 23 ˚C. The crude product was purified by silica gel column chromatography (elution with 0–2% diethyl ether in pentane) to give the title product as a colourless oil (68.9 mg, 94%).

The title product was observed in 46% yield by quantitative ^{1}H NMR spectroscopy following **General Procedure A** with cyclopropanemethanol (24.3 µL, 0.3 mmol, 1 eq.) and *tert*-amyl alcohol (0.1 mL, 3 M) at 85 ˚C.

M.p.: 34–36°C (from diethyl ether); **HRMS** (ESI^{+}) 245.1899 [M+H]$^{+}$, expected 245.1900; **^{1}H NMR** (600 MHz, $CDCl_3$) δ: 2.79 (2H, t, *J* = 7.3 Hz, H-2), 2.23 (3H, s, H-8), 2.19 (6H, s, H-6 or H-7), 2.11 (6H, s, H-7 or H-6), 1.62 (2H, app q, *J* = 7.3 Hz, H-3), 0.83–0.75 (1H, m, H-4), 0.48–0.40 (2H, m, H-5_a), 0.12–0.04 (2H, m, H-5_b); **^{13}C NMR** (150 MHz, $CDCl_3$) δ: 212.2 (C1), 141.0 (C-Ar), 135.42 (C-Ar), 133.2 (C-Ar), 127.4 (C-Ar), 45.7 (C2), 28.4 (C3), 17.3 (C7 or C6), 16.8 (C8), 16.1 (C6 or C7), 10.7 (C4), 4.8 (C5); **IR** (solid) (cm^{-1}): 2992, 2160, 2033, 1696, 1398, 1348, 1096, 1001, 871, 821.

Isolation of Side Product at 85 °C

Commentary: Many alcohols which gave good yield of product at 23 °C were trailed at 85 °C. It was generally found that the yield of the product was diminished, even for relatively benign substrates such as cyclopropylmethanol. In most cases complex mixtures were observed, however with cyclopropylmethanol a major side product was observed at 85 °C which was not observed at 23 °C. This product was isolated (**Supplementary Figure 1**).

Procedure:

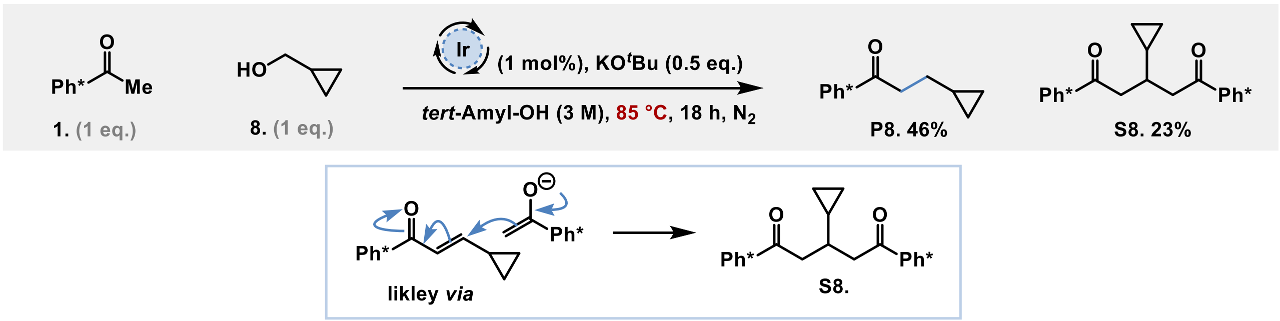

Supplementary Figure 1. Side product observation in high temperature reaction with cyclopropylmethanol. Yield is of isolated material.

3-Cyclopropyl-1,5-bis(2,3,4,5,6-pentamethylphenyl)pentane-1,5-dione (S8)

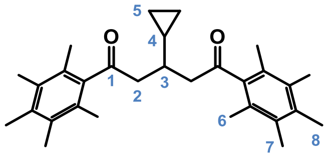

Prepared according to **General Procedure A** with cyclopropanemethanol (24.3 µL, 0.3 mmol, 1 eq.) and *tert*-amyl alcohol (0.1 mL, 3 M) at 85 ˚C. The crude product was purified by silica gel column chromatography (elution with 2% diethyl ether in pentane) to give the title product as a colourless solid (29.5 mg, 23%).

M.p.: 162–164 °C (from diethyl ether); **HRMS** (ESI$^+$) 433.3112 [M+H]$^+$, expected 433.3101; **^{1}H NMR** (400 MHz, $CDCl_3$) δ: 3.18–3.01 (4H, m, H-2), 2.24 (6H, s, H-8), 2.19 (12H, s, H-6 or H-7), 2.14 (12H, s, H-7 or H-6), 2.01–1.90 (1H, m, H-3), 1.00 (1H, dtt, J = 10.0, 8.0, 5.0 Hz, H-4), 0.55–0.44 (2H, m, H-5$_a$), 0.39–0.30 (2H, m, H-5$_b$); **^{13}C NMR** (101 MHz, $CDCl_3$) δ: 211.2 (C1), 140.7 (C-Ar), 135.4 (C-Ar), 133.2 (C-Ar), 127.5 (C-Ar), 49.8 (C2), 34.1 (C3), 17.1 (C7 or C6), 16.8 (C8), 16.5 (C4), 16.1 (C6 or C7), 5.0 (C5); **IR** (solid) (cm^{-1}): 1701, 1688, 1402, 1348, 1114, 1015, 941, 876, 846, 822.

3-(2,2-Difluorocyclopropyl)-1-(2,3,4,5,6-pentamethylphenyl)propan-1-one (P9)

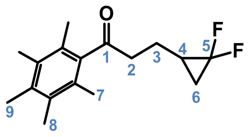

Prepared according to **General Procedure A** with (2,2-difluorocyclopropyl)methanol (25.5 µL, 0.3 mmol, 1 eq.) and *tert*-amyl alcohol (0.1 mL, 3 M) at 23 ˚C. The crude product was purified by silica gel column chromatography (elution with 0–2% diethyl ether in pentane) to give the title product as a colourless oil (49.6 mg, 59%).

The title product was observed in 14% yield by quantitative ^{1}H NMR spectroscopy following **General Procedure A** with (2,2-difluorocyclopropyl)methanol (25.5 µL, 0.3 mmol, 1 eq.) and *tert*-amyl alcohol (0.1 mL, 3 M) at 85 ˚C.

M.p.: 26–28°C (from diethyl ether); **HRMS** (ESI$^+$) 281.1710 [M+H]$^+$, expected 281.1712; **^{1}H NMR** (400 MHz, $CDCl_3$) δ: 2.80 (2H, t, *J* = 7.1 Hz, H-2), 2.24 (3H, s, H-9), 2.19 (6H, s, H-7 or H-8), 2.10 (6H, s, H-8 or H-7), 2.04–1.90 (1H, m, H-3_a), 1.88–1.77 (1H, m, H-3_b), 1.70 (app 1H, ddq, *J* = 15.0, 11.2, 7.4 Hz, H-4), 1.42 (1H, dddd, *J* = 12.6, 11.3, 9.8, 6.1 Hz, H-6_a), 0.98 (1H, dtd, *J* = 13.0, 7.4, 3.5 Hz, H-6_b); **^{13}C NMR** (101 MHz, $CDCl_3$) δ: 211.0 (C1), 140.4 (C-Ar), 135.7 (C-Ar), 133.3 (C-Ar), 127.4 (C-Ar), 114.6 (app t, *J* = 284.0 Hz, C5), 44.5 (d, *J* = 1.9 Hz, C2), 21.9 (t, *J* = 10.7 Hz, C4), 21.0 (d, *J* = 4.3 Hz, C3), 17.2 (C8 or C7), 16.8 (C9), 16.4 (t, *J* = 10.8 Hz, C6), 16.1 (C7 or C8); **^{19}F NMR** (376 MHz, $CDCl_3$) δ: −128.3 (ddd, *J* = 156.7, 13.5, 3.0 Hz), −145.2 (ddd, *J* = 156.7, 13.1, 4.0 Hz); **IR** (solid) (cm^{-1}): 1693, 1475, 1220, 1263, 1180, 1109, 1085, 1026, 969, 914.

3-(3-Methyloxetan-3-yl)-1-(2,3,4,5,6-pentamethylphenyl)propan-1-one (P10)

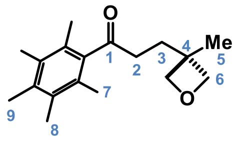

Prepared according to **General Procedure A** with 3-methyl-3-oxetanemethanol (29.9 µL, 0.3 mmol, 1 eq.) and *tert*-amyl alcohol (0.1 mL, 3 M) at 23 ˚C. The crude product was purified by silica gel column chromatography (elution with 0–10% ethyl acetate in pentane) to give the title product as a colourless solid (77.1 mg, 94%).

The title product was observed in 51% yield by quantitative ^{1}H NMR spectroscopy following **General Procedure A** with 3-methyl-3-oxetanemethanol (29.9 µL, 0.3 mmol, 1 eq.) and *tert*-amyl alcohol (0.1 mL, 3 M) at 85 ˚C.

M.p.: 60–62°C (from ethyl acetate); **HRMS** (ESI+) 275.2012 [M+H]+, expected 275.2006; **¹H NMR** (400 MHz, $CDCl_3$) δ: 4.44 (2H, d, *J* = 5.7 Hz, H-6$_a$), 4.36 (2H, d, *J* = 5.7 Hz, H-6$_b$), 2.69–2.61 (2H, m, H-2), 2.24 (3H, s, H-9), 2.19 (6H, s, H-7 or H-8), 2.11 (6H, s, H-8 or H-7), 2.10–2.04 (2H, m, H-3), 1.30 (3H, s, H-5); **¹³C NMR** (101 MHz, $CDCl_3$) δ: 211.6 (C1), 140.7 (C-Ar), 135.7 (C-Ar), 133.3 (C-Ar), 127.3 (C-Ar), 82.5 (C6), 40.9 (C2), 38.7 (C4), 32.2 (C3), 23.5 (C5), 17.4 (C8 or C7), 16.8 (C9), 16.1 (C7 or C8); **IR** (solid) (cm^{-1}): 1698, 1456, 1389, 1264, 1112, 1068, 1044, 979, 927, 806.

8-Bromo-1-(2,3,4,5,6-pentamethylphenyl)octan-1-one (P11)

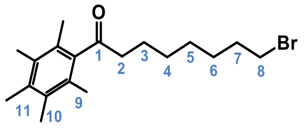

Prepared according to a modification to **General Procedure A Modification I** with $[Cp^*IrCl_2]_2$ (4.8 mg, **2 mol%**), 6-bromohexanol (39.3 µL, 0.3 mmol, 1 eq.) and *tert*-amyl alcohol (0.3 mL, 1 M) at 23 ˚C. The crude product was purified by silica gel column chromatography (elution with 40–60% toluene in pentane) to give the title product as a colourless oil (69.0 mg, 65%).

The title product was observed in 24% yield by quantitative ¹H NMR spectroscopy following **General Procedure A Modification I** with $[Cp^*IrCl_2]_2$ (4.8 mg, **2 mol%**), 6-bromohexanol (39.3 µL, 0.3 mmol, 1 eq.) and *tert*-amyl alcohol (0.3 mL, 1 M) at 85 ˚C.

HRMS (ESI+) 353.1478, 355.1460 [1:1, M+H]+, expected 353.1475, 355.1454 [1:1]; **¹H NMR** (400 MHz, $CDCl_3$): δ 3.41 (2H, t, *J* = 6.8 Hz, H-8), 2.67 (2H, t, *J* = 7.4 Hz, H-2), 2.23 (3H, s, H-11), 2.19 (6H, s, H-9 or H-10), 2.10 (6H, s, H-10 or H-9), 1.86 (2H, p, *J* = 7.0 Hz, H-7), 1.72 (2H, p, *J* = 7.3 Hz, H-3), 1.52–1.43 (2H, m, H-6), 1.43–1.33 (4H, m, H-4, H-5); **¹³C NMR** (101 MHz, $CDCl_3$): δ 212.1 (C1), 140.9 (C-Ar), 135.5 (C-Ar), 133.2 (C-Ar), 127.4 (C-Ar), 45.7 (C2), 34.1 (C8), 32.9 (C7), 29.1 (C4), 28.8 (C5), 28.1 (C6), 23.2 (C3), 17.3 (C10 or C9), 16.8 (C11), 16.1 (C9 or C10); **IR** (thin film) (cm^{-1}): 2931, 1702, 1461, 1404, 1305, 1256, 1116, 914, 745, 645.

8-Chloro-1-(2,3,4,5,6-pentamethylphenyl)octan-1-one (P12)

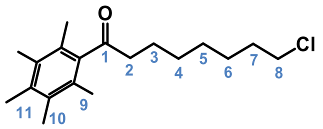

Prepared according to a modification to **General Procedure A** with $[Cp^*IrCl_2]_2$ (4.8 mg, **2 mol%**), 6-chlorohexanol (40.0 µL, 0.3 mmol, 1 eq.) and *tert*-amyl alcohol (0.3 mL, 1 M) at 23 ˚C. The crude product

was purified by silica gel column chromatography (elution with 0–4% diethyl ether in pentane) to give the title product as a colourless oil (87.0 mg, 95%).

The title product was observed in 89% yield by quantitative ^{1}H NMR spectroscopy following **General Procedure A** with $[Cp^{*}IrCl_2]_2$ (2.4 mg, **1 mol%**), 6-chlorohexanol (40.0 µL, 0.3 mmol, 1 eq.) and *tert*-amyl alcohol (0.3 mL, 1 M) at 23 ˚C. The product was isolated as a mixture with Ph* methyl ketone (observed in 7% yield). Increasing concentration to 3 M, or using 1.5 eq. of 6-chlorohexanol did not improve conversion, however increasing the catalyst loading to 2% $[Cp^{*}IrCl_2]_2$ proved successful.

The title product was observed in 24% yield by quantitative ^{1}H NMR spectroscopy following **General Procedure A** with $[Cp^{*}IrCl_2]_2$ (2.4 mg, **1 mol%**), 6-chlorohexanol (40.0 µL, 0.3 mmol, 1 eq.) and *tert*-amyl alcohol (0.3 mL, 1 M) at 85 ˚C.

HRMS (ESI^{+}) 309.1989 $[M+H]^{+}$, expected 309.1980 ; **^{1}H NMR** (400 MHz, $CDCl_3$) δ: 3.54 (2H, t, *J* = 6.7 Hz, H-8), 2.68 (2H, t, *J* = 7.4 Hz, H-2), 2.23 (3H, s, H-11), 2.18 (6H, s, H-9 or H-10), 2.10 (6H, s, H-10 or H-9), 1.83–1.66 (4H, m, H-3, H-7), 1.52–1.32 (6H, m, H-4, H-5, H-6); **^{13}C NMR** (101 MHz, $CDCl_3$) δ: δ 212.2 (C1), 140.9 (C-Ar), 135.5 (C-Ar), 133.2 (C-Ar), 127.4 (C-Ar), 45.7 (C2), 45.2 (C8), 32.7 (C7), 29.1 (C5), 28.9 (C4), 26.8 (C6), 23.2 (C3), 17.3 (C10 or C9), 16.8 (C11), 16.1 (C9 or C10); **IR** (thin film) (cm^{-1}): 3018, 1698, 1447, 1403, 1382, 1352, 1306, 1218, 757, 668.

5-(2-Bromophenyl)-1-(2,3,4,5,6-pentamethylphenyl)pentan-1-one (P13)

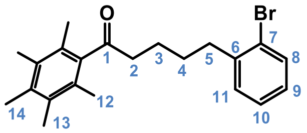

Prepared according to **General Procedure A** with 3-(2-bromophenyl)propan-1-ol (46.1 µL, 0.3 mmol, 1 eq.) and *tert*-amyl alcohol (0.1 mL, 3 M) at 23 ˚C. The crude product was purified by silica gel column chromatography (elution with 60–80% toluene in pentane) to give the title product as a colourless solid (92.8 mg, 80%).

M.p.: 42–44 °C (from toluene); **HRMS** (ESI^{+}) 387.1330 $[M+H]^{+}$, expected 387.1318; **^{1}H NMR** (400 MHz, $CDCl_3$) δ: 7.56–7.50 (1H, d, *J* = 8.0 Hz, H-8), 7.25–7.21 (2H, m, H-10, H-11), 7.10–7.03 (1H, m, H-9), 2.79 (2H, t, *J* = 7.7 Hz, H-5), 2.74 (2H, t, *J* = 7.3 Hz, H-2), 2.24 (3H, s, H-14), 2.19 (6H, s, H-12 or H-13), 2.11 (6H, s, H-13 or H-12), 1.82 (2H, ddd, *J* = 14.0, 7.3, 4.8 Hz, H-3), 1.76–1.65 (2H, m, H-4); **^{13}C NMR** (101 MHz, $CDCl_3$) δ: 211.9 (C1), 141.6 (C-Ar), 140.8 (C6), 135.5 (C-Ar), 133.2 (C-Ar), 132.9 (C8), 130.5 (C11), 127.7 (C9), 127.5 (C10), 127.4 (C-Ar), 124.5 (C7), 45.5 (C2), 36.2 (C5), 29.6 (C4), 23.1 (C3), 17.4 (C13 or C12), 16.8 (C14), 16.1 (C12 or C13); **IR** (solid) (cm^{-1}): 1697, 1471, 1456, 1402, 1363, 1127, 1020, 1107, 751, 658.

5,9-Dimethyl-1-(2,3,4,5,6-pentamethylphenyl)dec-8-en-1-one (P14)

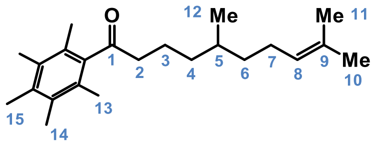

Prepared according to **General Procedure A** with 3,7-dimethyloct-6-en-1-ol (54.8 µL, 0.3 mmol, 1 eq.) and *tert*-amyl alcohol (0.1 mL, 3 M) at 23 °C. The crude product was purified by silica gel column chromatography (elution with 0–2% diethyl ether in pentane) to give the title product as a colourless oil (56.2 mg, 57%).

The title product was observed in 58% yield by quantitative ^{1}H NMR spectroscopy following **General Procedure A** with 3,7-dimethyloct-6-en-1-ol (54.8 µL, 0.3 mmol, 1 eq.) and *tert*-amyl alcohol (0.1 mL, 3 M) at 85 °C.

HRMS (ESI$^+$) 329.2833 [M+H]$^+$, expected 329.2839; **^{1}H NMR** (400 MHz, $CDCl_3$) δ: 5.11 (1H, tt, *J* = 7.1, 1.4 Hz, H-8), 2.66 (2H, ddd, *J* = 8.0, 6.8, 1.2 Hz, H-2), 2.24 (3H, s, H-15), 2.19 (6H, s, H-13 or H-14), 2.10 (6H, s, H-14 or H-13), 2.05–1.88 (2H, m, H-7), 1.84–1.66 (2H, m, H-3), 1.69 (3H, d, *J* = 1.4 Hz, H-10), 1.61 (3H, d, *J* = 1.4 Hz, H-11), 1.52–1.30 (3H, m, H-5, H-6), 1.29–1.10 (2H, m, H-4), 0.91 (3H, d, *J* = 6.5 Hz, H-12); **^{13}C NMR** (101 MHz, $CDCl_3$) δ: 212.2 (C1), 141.0 (C-Ar), 135.4 (C-Ar), 133.2 (C-Ar), 131.2 (C9), 127.4 (C-Ar), 125.0 (C8), 46.1 (C2), 37.2 (C6), 36.6 (C4), 32.5 (C5), 25.9 (C10), 25.7 (C7), 20.9 (C3), 19.6 (C12), 17.8 (C11), 17.3 (C14 or C13), 16.8 (C15), 16.1 (C13 or C14); **IR** (thin film) (cm^{-1}): 2923, 1701, 1452, 1404, 1378, 1305, 1120, 912, 733.

1-(2,3,4,5,6-Pentamethylphenyl)-3-ferrocenylpropan-1-one (P15)

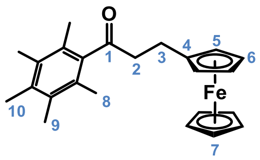

Prepared according to **General Procedure A** with ferrocenemethanol (64.8 mg, 0.3 mmol, 1 eq.) and *tert*-amyl alcohol (0.3 mL, 1 M) at 23 °C. The crude product was purified by silica gel column chromatography (elution with 0–2% diethyl ether in pentane) to give the title product as an orange oil (109.6 mg, 94%).

The title product was observed in 86% yield by quantitative ^{1}H NMR spectroscopy following **General Procedure A** with ferrocenemethanol (64.8 mg, 0.3 mmol, 1 eq.) and *tert*-amyl alcohol (0.3 mL, 1 M) at 85 °C.

HRMS (ESI$^+$) 389.1543 [M+H]$^+$, expected 389.1562; **^{1}H NMR** (400 MHz, CDCl$_3$) δ: 4.12 (5H, s, H-7), 4.10 (2H, dd, *J* = 1.9, 1.9 Hz, H-6), 4.07 (2H, dd, *J* = 1.9, 1.9 Hz, H-5), 3.00–2.91 (2H, m, H-2), 2.79 (2H, ddd, *J* = 8.7, 6.6, 1.9 Hz, H-3), 2.25 (3H, s, H-10), 2.20 (6H, s, H-8 or H-9), 2.10 (6H, s, H-9 or H-8); **^{13}C NMR** (101 MHz, CDCl$_3$) δ: 211.3 (C1), 140.7 (C-Ar), 135.6 (C-Ar), 133.2 (C-Ar), 127.5 (C-Ar), 87.9 (C4), 68.6 (C7), 68.2 (C6), 67.4 (C5), 46.9 (C2), 23.1 (C3), 17.3 (C9 or C8), 16.8 (C10), 16.1 (C8 or C9); **IR** (solid) (cm^{-1}): 1694, 1381, 1350, 1114, 1106, 1037, 1027, 1000, 928, 816.

1-Morpholino-6-(2,3,4,5,6-pentamethylphenyl)hexane-1,6-dione (P16)

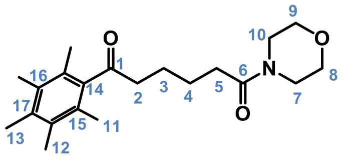

Prepared according to **General Procedure A** with 4-hydroxy-1-morpholinobutan-1-one (52.0 mg, 0.3 mmol, 1 eq.) and *tert*-amyl alcohol (0.1 mL, 3 M) at 23 ˚C. The crude product was purified by silica gel column chromatography (elution with 50–60% ethyl acetate in pentane) to give the title product as a colourless solid (35.5 mg, 34%).

The title product was not observed by quantitative ^{1}H NMR spectroscopy following **General Procedure A** with 4-hydroxy-1-morpholinobutan-1-one (52.0 mg, 0.3 mmol, 1 eq.) and *tert*-amyl alcohol (0.1 mL, 3 M) at 85 ˚C.

M.p.: 72–74 °C (from ethyl acetate); **HRMS** (ESI$^+$) 346.2384 [M+H]$^+$, expected 346.2377; **^{1}H NMR** (400 MHz, CDCl$_3$) δ: 3.67 (4H, d, *J* = 5.1 Hz, H-8, H-9), 3.62 (2H, d, *J* = 5.1 Hz, H-7$_a$, H-10$_a$), 3.47 (2H, dd, *J* = 8.7, 5.5 Hz, H-7$_b$, H-10$_b$), 2.71 (2H, t, *J* = 6.6 Hz, H-2), 2.37 (2H, t, *J* = 6.9 Hz, H-5), 2.23 (3H, s, H-13), 2.18 (6H, s, H-12), 2.09 (6H, s, H-11), 1.75 (4H, app p, *J* = 3.7 Hz, H-3, H-4); **^{13}C NMR** (101 MHz, CDCl$_3$) δ: 211.7 (C1), 171.5 (C6), 140.7 (C14), 135.5 (C17), 133.2 (C-16), 127.4 (C15), 67.1 (C8 or C9), 66.8 (C9 or C8), 46.1 (C7 or C10), 45.5 (C2), 42.0 (C10 or C7), 33.1 (C5), 24.9 (C3), 23.2 (C4), 17.3 (C11), 16.8 (C13), 16.1 (C12); **IR** (solid) (cm^{-1}): 1695, 1654, 1640, 1436, 1270, 1238, 1213, 1117, 1067, 1024.

4-(Dibenzylamino)-1-(2,3,4,5,6-pentamethylphenyl)pentan-1-one (P17)

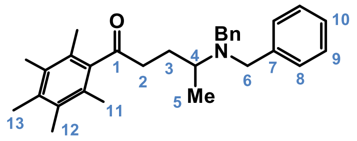

Prepared according to **General Procedure A** with 2-(dibenzylamino)propan-1-ol (76.6 mg, 0.3 mmol, 1 eq.) and *tert*-amyl alcohol (0.1 mL, 3 M) at 23 ˚C. The crude product was purified by silica gel column

chromatography (elution with 0–2% diethyl ether in pentane) to give the title product as a colourless oil (114 mg, 89%).

M.p.: 72–74 °C (from diethyl ether); **HRMS** (ESI+) 428.2960 [M+H]+, expected 428.2948; **^{1}H NMR** (400 MHz, $CDCl_3$) δ: 7.36–7.31 (4H, m, H-8), 7.29–7.23 (4H, m, H-9), 7.23–7.16 (2H, m, H-10), 3.75 (2H, d, *J* = 13.7 Hz, H-7_a), 3.39 (2H, d, *J* = 13.8 Hz, H-7_b), 3.06–2.93 (1H, m, H-2_a), 2.81–2.68 (1H, m, H-4), 2.62–2.49 (1H, m, H-2_b), 2.23 (3H, s, H-13), 2.17 (6H, s, H-11 or H-12), 2.09–1.97 (1H, m, H-3_a), 2.01 (6H, s, H-12 or H-11), 1.71 (1H, ddt, *J* = 14.2, 10.5, 5.2 Hz, H-3_b), 1.05 (3H, d, *J* = 6.5 Hz, H-5); **^{13}C NMR** (101 MHz, $CDCl_3$) δ: 212.7 (C1), 141.1(C-Ar), 140.5 (C-Ar), 135.4 (C-Ar), 133.2 (C-Ar), 128.8 (C8), 128.3 (C9), 127.5 (C7), 126.9 (C10), 53.4 (C7), 52.4 (C4), 43.3 (C2), 27.5 (C3), 17.3 (C12 or C11), 16.8 (C13), 16.1 (C11 or C12), 13.3 (C5).

The spectral data matched that previously reported in the literature.[8]

5-(Dimethylamino)-1-(2,3,4,5,6-pentamethylphenyl)pentan-1-one (P18)

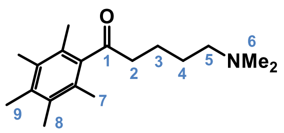

Prepared according to **General Procedure A** with 3-(dimethylamino)propan-1-ol (35.5 μL, 0.3 mmol, 1 eq.) and *tert*-amyl alcohol (0.1 mL, 3 M) at 23 ˚C. The crude product was purified by silica gel column chromatography (elution with 5% triethylamine in ethyl acetate) to give the title product as a pale-yellow oil (66.1 mg, 80%).

M.p.: 28–30 °C (from ethyl acetate); **HRMS** (ESI+) 276.2335 [M+H]+, expected 276.2322; **^{1}H NMR** (400 MHz, $CDCl_3$) δ: 2.70 (2H, t, *J* = 7.3 Hz, H-2), 2.34–2.26 (2H, m, H-5), 2.22 (9H, app s, H-6, H-9), 2.18 (6H, s, H-7 or H-8), 2.09 (6H, s, H-8 or H-7), 1.79–1.67 (2H, m, H-3), 1.61–1.49 (2H, m, H-4); **^{13}C NMR** (101 MHz, $CDCl_3$) δ: 211.9 (C1), 140.9 (C-Ar), 135.4 (C-Ar), 133.2 (C-Ar), 127.4 (C-Ar), 59.8 (C5), 45.7 (C6), 45.6 (C2), 27.5 (C4), 21.3 (C3), 17.3 (C8 or C7), 16.8 (C9), 16.1 (C7 or C8); **IR** (solid) (cm^{-1}): 2816, 2772, 1703, 1466, 1456, 1204, 1119, 1096, 1043, 866.

5-Morpholino-1-(2,3,4,5,6-pentamethylphenyl)pentan-1-one (P19)

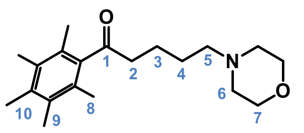

Prepared according to **General Procedure A** with 3-morpholinopropan-1-ol (41.6 μL, 0.3 mmol, 1 eq.) and *tert*-amyl alcohol (0.1 mL, 3 M) at 23 ˚C. The crude product was purified by silica gel column

chromatography (elution with 60% ethyl acetate in pentane + 5% triethylamine) to give the title product as a pale-yellow solid (65.5 mg, 72%).

M.p.: 50–52 °C (from ethyl acetate); **HRMS** (ESI+) 340.2244 [M+Na]+, expected 340.2247; **^{1}H NMR** (400 MHz, $CDCl_3$) δ: 3.72 (4H, t, *J* = 4.8 Hz, H-7), 2.70 (2H, t, *J* = 7.3 Hz, H-2), 2.44 (4H, t, *J* = 4.8 Hz, H-6), 2.37 (2H, t, *J* = 7.3 Hz, H-5), 2.23 (3H, s, H-10), 2.18 (6H, s, H-8 or H-9), 2.09 (6H, s, H-9 or H-8), 1.74 (2H, p, *J* = 7.3 Hz, H-3), 1.64–1.52 (2H, m, H-4); **^{13}C NMR** (101 MHz, $CDCl_3$) δ: 211.9 (C1), 140.8 (C-Ar), 135.5 (C-Ar), 133.2 (C-Ar), 127.4 (C-Ar), 67.1 (C7), 59.0 (C5), 53.9 (C6), 45.5 (C2), 26.2 (C4), 21.3 (C3), 17.3 (C9 or C8), 16.8 (C10), 16.1 (C8 or C9); **IR** (solid) (cm^{-1}): 2851, 2811, 697, 1444, 1328, 1309, 1271, 1136, 1120, 867.

3-(1-Benzylpiperidin-4-yl)-1-(2,3,4,5,6-pentamethylphenyl)propan-1-one (P20)

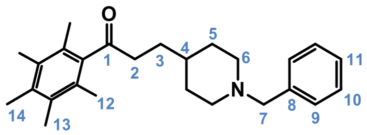

Prepared according to **General Procedure A** with (1-benzylpiperidin-4-yl)methanol (61.6 mg, 0.3 mmol, 1 eq.) and *tert*-amyl alcohol (0.1 mL, 3 M) at 23 ˚C. The crude product was purified by silica gel column chromatography (elution with 10–30% ethyl acetate in pentane + 6% triethylamine) to give the title product as a pale-yellow solid (104.6 mg, 96%).

M.p.: 90–92 °C (from ethyl acetate); **HRMS** (ESI+) 378.2804 [M+H]+, expected 378.2791; **^{1}H NMR** (400 MHz, $CDCl_3$) δ: 7.34–7.28 (4H, m, H-9, H-10), 7.27–7.21 (1H, m, H-11), 3.49 (2H, s, H-7), 2.94–2.82 (2H, m, H-6_a), 2.74–2.63 (2H, m, H-2), 2.23 (3H, s, H-14), 2.18 (6H, s, H-12 or H-13), 2.08 (6H, s, H-13 or H-12), 1.94 (2H, app td, *J* = 9.8, 4.4 Hz, H-6_b), 1.73–1.61 (4H, m, H-3, H-5_a,), 1.37–1.22 (3H, m, H-4, H-5_b); **^{13}C NMR** (101 MHz, $CDCl_3$) δ: 212.3 (C1), 141.0 (C-Ar), 138.6 (C-8), 135.4 (C-Ar), 133.2 (C-Ar), 129.3 (C9), 128.2 (C10), 127.4 (C-Ar), 127.0 (C11), 63.6 (C7), 53.9 (C6), 43.1 (C2), 35.4 (C4), 32.4 (C5), 29.9 (C3), 17.3 (C13 or C12), 16.8 (C14), 16.1 (C12 or C13); **IR** (solid) (cm^{-1}): 2805, 2764, 1698, 1262, 1114, 1074, 1025, 799, 733, 696.

3-(1-Methyl-1*H*-indol-5-yl)-1-(2,3,4,5,6-pentamethylphenyl)propan-1-one (P21)

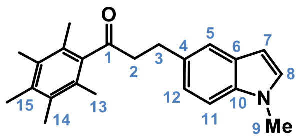

Prepared according to **General Procedure A** with (1-methyl-1*H*-indol-5-yl)methanol (48.4 mg, 0.3 mmol, 1 eq.) and *tert*-amyl alcohol (0.3 mL, 1 M) at 23 ˚C. The crude product was purified by silica gel

column chromatography (elution with 5% ethyl acetate in pentane + 1% triethylamine) to give the title product as a pale-yellow solid (96.8 mg, 97%).

The title product was observed in 96% yield by quantitative ^{1}H NMR spectroscopy following **General Procedure A** with (1-methyl-1*H*-indol-5-yl)methanol (48.4 mg, 0.3 mmol, 1 eq.) and *tert*-amyl alcohol (0.3 mL, 1 M) at 85 ˚C.

M.p.: 80–82 °C (from ethyl acetate); **HRMS** (ESI+) 334.2155 [M+H]+, expected 334.2165; **^{1}H NMR** (400 MHz, $CDCl_3$) δ: 7.46 (1H, d, *J* = 1.6 Hz, H-5), 7.23 (1H, d, *J* = 8.2 Hz, H-11), 7.10 (1H, dd, *J* = 8.4, 1.7 Hz, H-12), 7.01 (1H, d, *J* = 3.0 Hz, H-8), 6.40 (1H, d, *J* = 3.0 Hz, H-7), 3.76 (3H, s, H-9), 3.20–3.10 (2H, m, H-3), 3.10–3.00 (2H, m, H-2), 2.22 (3H, s, H-15), 2.17 (6H, s, H-13 or H-14), 2.07 (6H, s, H-14 or H-13); **^{13}C NMR** (101 MHz, $CDCl_3$) δ: 211.7 (C1), 140.8 (C-Ar), 135.6 (C10), 135.5 (C-Ar), 133.2 (C-Ar), 131.9 (C4), 129.1 (C8), 128.8 (C6), 127.5 (C-Ar), 122.5 (C12), 120.2 (C5), 109.2 (C11), 100.6 (C7), 48.2 (C2), 33.0 (C9), 29.6 (C3), 17.3 (C14 or C13), 16.8 (C15), 16.1 (C13 or C14); **IR** (solid) (cm^{-1}): 1698, 1493, 1354, 1304, 1245, 1114, 933, 800, 761, 717.

(1-((1-Methyl-1*H*-indol-3-yl)methyl)piperidin-4-yl)methanol (22)

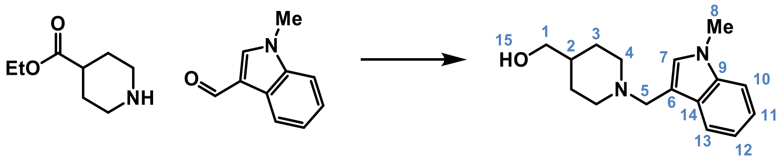

Sodium triacetoxyborohydride (2.00 mL, 9.4 mmol, 1.5 eq.) was added to a solution of 1-methyl-1*H*-indole-3-carbaldehyde (1.00 g, 6.28 mmol, 1.0 eq.) and ethyl piperidine-4-carboxylate (0.968 mL, 6.28 mmol, 1.0 eq.) in CH_2Cl_2 (31.4 mL, 0.2 M) at rt. The reaction mixture was stirred for 16 h, quenched with water (15 mL) and extracted with CH_2Cl_2 (3 × 20 mL); the combined organic extracts were dried using Mg_2SO_4, filtered and concentrated *in vacuo* to yield a crude oil. $LiAlH_4$ (2 M in THF, 6.28 mL, 12.6 mmol, 2.0 eq.) was added dropwise to a solution of this oil in anhydrous THF (31.4 mL) at 0 ˚C, which was stirred for 1 h at rt. The reaction solution was quenched by dropwise addition of 5% w/v aq. potassium sodium tartrate (Rochelle's salt, 5 mL) at 0 ˚C. The reaction mixture was extracted with CH_2Cl_2 (3 × 20 mL); the combined organic extracts were dried using Mg_2SO_4, filtered and concentrated *in vacuo* to yield a crude oil. This oil was purified by silica gel column chromatography (elution with 5% methanol in CH_2Cl_2 + 1% triethylamine) to give the title product as a pale-yellow solid (0.998 g, 62%).

M.p.: 58–60 °C (from CH_2Cl_2); **HRMS** (ESI+) 259.1799 [M+H]+, expected 259.1805; **^{1}H NMR** (400 MHz, $CDCl_3$) δ: 7.70 (1H, dt, *J* = 7.9, 1.0 Hz, H-13), 7.30 (1H, dt, *J* = 8.2, 1.0 Hz, H-10), 7.23 (1H, ddd, *J* = 8.2, 6.9, 1.2 Hz, H-11), 7.12 (1H, ddd, *J* = 7.9, 6.9, 1.1 Hz, H-12), 7.04 (1H, s, H-7), 3.77 (3H, s, H-8), 3.72 (2H, s, H-5), 3.46 (2H, d, *J* = 6.4 Hz, H-1), 3.02 (2H, app dd, *J* = 11.6, 2.6 Hz, H-4_a), 2.03 (2H, td, *J* = 11.7, 2.6 Hz, H-

4b), 1.76–1.65 (2H, m, H-3a), 1.46 (1H, dtt, *J* = 15.1, 6.4, 3.9 Hz, H-2), 1.29 (2H, qd, *J* = 12.1, 3.9 Hz, H-3b); **^{13}C NMR** (101 MHz, $CDCl_3$) δ: 136.9 (C9), 128.8 (C7), 128.8 (C14), 121.6 (C11), 119.5 (C13), 119.1 (C12), 110.8 (C6), 109.3 (C10), 67.9 (C1), 53.5 (C5), 53.3 (C4), 38.6 (C2), 32.8 (C8), 28.9 (C3); **IR** (solid) (cm^{-1}): 3143, 2914, 1475, 1326, 1141, 1126, 1048, 1008, 776, 741.

3-(1-((1-Methyl-1*H*-indol-3-yl)methyl)piperidin-4-yl)-1-(2,3,4,5,6-pentamethylphenyl)propan-1-one (P22)

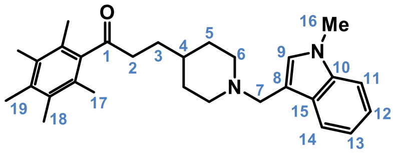

Prepared according to **General Procedure A** with (1-((1-methyl-1*H*-indol-3-yl)methyl)piperidin-4-yl)methanol (77.5 mg, 0.3 mmol, 1 eq.) and *tert*-amyl alcohol (0.3 mL, 1 M) at 23 ˚C. The crude product was purified by silica gel column chromatography (elution with 0–2% methanol in CH_2Cl_2 + 1% triethylamine) to give the title product as a pale-yellow solid (112.4 mg, 87%).

The title product was observed in 42% yield by quantitative ^{1}H NMR spectroscopy following **General Procedure A** with (1-((1-Methyl-1*H*-indol-3-yl)methyl)piperidin-4-yl)methanol (77.5 mg, 0.3 mmol, 1 eq.) and *tert*-amyl alcohol (0.3 mL, 1 M) at 85 ˚C.

M.p.: 110–112 °C (from CH_2Cl_2); **HRMS** (ESI$^+$) 431.3043 [M+H]$^+$, expected 431.3057; **^{1}H NMR** (400 MHz, $CDCl_3$) δ: 7.66 (1H, dt, *J* = 7.9, 1.0 Hz, H-14), 7.31 (1H, dt, *J* = 8.3, 1.0 Hz, H-11), 7.23 (1H, dd, *J* = 8.2, 1.2 Hz, H-12), 7.16–7.14 (1H, m, H-9), 7.14–7.11 (1H, m, H-13), 3.82 (2H, s, H-7), 3.78 (3H, s, H-16), 3.08 (2H, d, *J* = 11.2 Hz, H-6a), 2.66 (2H, dd, *J* = 8.3, 7.0 Hz, H-2), 2.22 (3H, s, H-19), 2.17 (6H, s, H-17 or H-18), 2.17–2.10 (2H, m, H-6b), 2.07 (6H, s, H-18 or H-17), 1.68 (4H, app tt, *J* = 13.3, 5.1 Hz, H-3, H-5a), 1.48–1.34 (3H, m, H-4, H-5b); **^{13}C NMR** (101 MHz, $CDCl_3$) δ: 212.2 (C1), 140.9 (C-Ar), 136.9 (C10), 135.5 (C-Ar), 133.2 (C-Ar), 129.6 (C9), 128.7 (C15), 127.3 (C-Ar), 121.7 (C12), 119.4 (C13), 119.2 (C14), 109.4 (C11), 53.3 (C6), 53.1 (C7), 42.9 (C2), 35.0 (C4), 32.9 (C16), 31.7 (C5), 29.7 (C3), 17.3 (C18 or C17), 16.8 (C19), 16.1 (C17 or C18); **IR** (solid) (cm^{-1}): 1696, 1474, 1407, 1329, 1271, 1125, 1110, 1011, 975, 724.

(3-(Pyrrolidin-1-ylmethyl)bicyclo[1.1.1]pentan-1-yl)methanol (23)

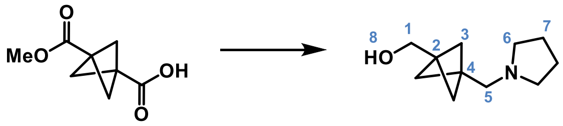

Oxalyl chloride (0.151 mL, 1.76 mmol, 1.0 eq.) was added dropwise to a solution of 3-(methoxycarbonyl)bicyclo[1.1.1]pentane-1-carboxylic acid (0.300 g, 1.76 mmol, 1.0 eq.) in CH_2Cl_2 (8.8

mL, 0.2 M) and DMF (1 drop) at rt. The solution was stirred for 30 min, after which effervescence ceased. Pyrrolidine (0.290 mL, 3.52 mmol, 2.0 eq.) was added dropwise to this solution at 0 ˚C and stirred for 30 min at rt. The reaction mixture was extracted with CH_2Cl_2 (3 × 20 mL); the combined organic extracts were dried using Mg_2SO_4, filtered and concentrated *in vacuo* to yield a crude oil. $LiAlH_4$ (2 M in THF, 1.76 mL, 3.52 mmol, 2.0 eq.) was added dropwise to a solution of this oil in anhydrous THF (8.8 mL) at 0 ˚C, which was stirred for 1 h at rt. The reaction solution was quenched by dropwise addition of 5% w/v aq. potassium sodium tartrate (Rochelle's salt, 5 mL) at 0 ˚C. The reaction mixture was extracted with CH_2Cl_2 (3 × 20 mL); the combined organic extracts were dried using Mg_2SO_4, filtered and concentrated *in vacuo* to yield a crude oil. This oil was purified by silica gel column chromatography (elution with 5–10% methanol in CH_2Cl_2 + 1% triethylamine) to give the title product as a pale-yellow oil (0.234 g, 73%).

HRMS (ESI^+) 182.1536 $[M+H]^+$, expected 182.1539.; **^{1}H NMR** (400 MHz, $CDCl_3$) δ: 4.14 (1H, s, H-8), 3.54 (2H, s, H-1), 2.57 (2H, s, H-5), 2.53 (4H, tt, *J* = 4.4, 1.9 Hz, H-6), 1.80–1.72 (4H, m, H-7), 1.65 (6H, s, H-3); **^{13}C NMR** (101 MHz, $CDCl_3$) δ: 63.2 (C1), 58.5 (C5), 55.0 (C6), 49.6 (C3), 40.6 (C1), 39.3 (C4), 23.5 (C7); **IR** (thin film) (cm^{-1}): 3386, 2907, 2800, 1268, 1143, 1090, 1018, 875, 753, 666.

1-(2,3,4,5,6-Pentamethylphenyl)-3-(3-(pyrrolidin-1-ylmethyl)bicyclo[1.1.1]pentan-1-yl)propan-1-one (P23)

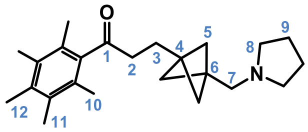

Prepared according to **General Procedure A** with (3-(pyrrolidin-1-ylmethyl)bicyclo[1.1.1]pentan-1-yl)methanol (54.4 mg, 0.3 mmol, 1 eq.) and *tert*-amyl alcohol (0.3 mL, 1 M) at 23 ˚C. The crude product was purified by silica gel column chromatography (elution with 0–2% methanol in CH_2Cl_2 + 1% triethylamine) to give the title product as a pale-yellow solid (77.2 mg, 73%).

The title product was observed in 25% yield by quantitative ^{1}H NMR spectroscopy following **General Procedure A** with (3-(pyrrolidin-1-ylmethyl)bicyclo[1.1.1]pentan-1-yl)methanol (54.4 mg, 0.3 mmol, 1 eq.) and *tert*-amyl alcohol (0.3 mL, 1 M) at 85 ˚C.

M.p.: 78–80 °C (from CH_2Cl_2); **HRMS** (ESI^+) 354.2781 $[M+H]^+$, expected 354.2791; **^{1}H NMR** (400 MHz, $CDCl_3$) δ: 2.69–2.56 (6H, m, H-2, H-7, H-8), 2.22 (3H, s, H-12), 2.18 (6H, s, H-10 or H-11), 2.08 (6H, s, H-11 or H-10), 1.89–1.79 (6H, m, H-3, H-9), 1.64 (6H, s, H-5); **^{13}C NMR** (101 MHz, $CDCl_3$) δ: 211.9 (C1), 140.8 (C-Ar), 135.5 (C-Ar), 133.2 (C-Ar), 127.4 (C-Ar), 58.2 (C7), 55.0 (C8), 51.0 (C5), 42.8 (C2), 39.9

(C6), 38.3 (C4), 25.6 (C3), 23.6 (C9), 17.4 (C11 or C10), 16.8 (C12), 16.1 (C10 or C11); **IR** (solid) (cm^{-1}): 3418, 1699, 1444, 1264, 1131, 935, 755, 665.

3-4-(1*H*-Imidazol-1-yl)phenyl)-1-(2,3,4,5,6-pentamethylphenyl)propan-1-one (P24)

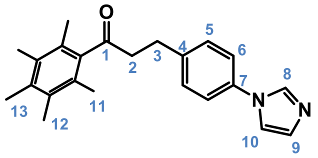

Prepared according to **General Procedure A** with (4-(1*H*-imidazol-1-yl)phenyl)methanol (34.1 µL, 0.3 mmol, 1 eq.) and *tert*-amyl alcohol (0.3 mL, 1 M) at 23 ˚C. The crude product was purified by silica gel column chromatography (elution with 60% ethyl acetate in pentane + 5% triethylamine) to give the title product as a colourless solid (81.5 mg, 78%).

M.p.: 120–122 °C (from ethyl acetate); **HRMS** (ESI$^+$) 347.2111 [M+H]$^+$, expected 347.2118; **^{1}H NMR** (400 MHz, $CDCl_3$) δ: 7.83 (1H, app t, *J* = 1.2 Hz, H-8), 7.36 (2H, d, *J* = 8.5 Hz, H-5), 7.31 (2H, d, *J* = 8.5 Hz, H-6), 7.26 (1H, app s, H-10), 7.20 (1H, app t, *J* = 1.2 Hz, H-9), 3.11 (2H, td, *J* = 7.2, 1.5 Hz, H-3), 3.02 (2H, td, *J* = 7.3, 1.6 Hz, H-2), 2.23 (3H, s, H-13), 2.17 (6H, s, H-11 or H-12), 2.04 (6H, s, H-12 or H-11); **^{13}C NMR** (101 MHz, $CDCl_3$) δ: 210.5 (C1), 140.8 (C4), 140.4 (C-Ar), 135.7 (C8-Ar), 135.7 (C7), 135.7 (C8), 133.3 (C-Ar), 130.5 (C9), 130.1 (C5), 127.4 (C-Ar), 121.7 (C6), 118.4 (C10), 46.9 (C2), 28.9 (C3), 17.2 (C12 or C11), 16.8 (C13), 16.1 (C11 or C12); **IR** (solid) (cm^{-1}): 1736, 1693, 1553, 1303, 1248, 1057, 833, 818 735, 663.

1-(2,3,4,5,6-Pentamethylphenyl)-3-(pyridin-3-yl)propan-1-one (P25)

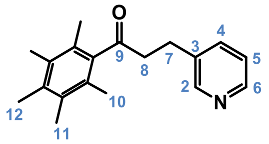

Prepared according to **General Procedure A** with 3-pyridinemethanol (29.1 µL, 0.3 mmol, 1 eq.) and *tert*-amyl alcohol (0.3 mL, 1 M) at 23 ˚C. The crude product was purified by silica gel column chromatography (elution with 0–15% ethyl acetate in pentane + 1% triethylamine) to give the title product as a colourless solid (8.4 mg, 10%).

Prepared according to **General Procedure A** with 3-pyridinemethanol (29.1 µL, 0.3 mmol, 1 eq.) and *tert*-amyl alcohol (0.3 mL, 1 M) at 85 ˚C. The crude product was purified by silica gel column chromatography (elution with 10% ethyl acetate in pentane + 1% triethylamine) to give the title product as a colourless solid (77.2 mg, 91%).

M.p.: 48–50 °C (from diethyl ether); **HRMS** (ESI+) 282.1847 [M+H]+, expected 282.1852; **^{1}H NMR** (400 MHz, $CDCl_3$) δ: 8.62–8.37 (2H, m, H-2, H-6), 7.60 (1H, dt, *J* = 7.8, 2.0 Hz, H-4), 7.22 (1H, dd, *J* = 7.9, 4.7 Hz, H-5), 3.12–2.94 (4H, m, H-7, H-8), 2.22 (3H, s, H-12), 2.16 (6H, s, H-10 or H-11), 2.01 (6H, s, H-11 or H-10); **^{13}C NMR** (101 MHz, $CDCl_3$) δ: 210.3 (C9), 150.1 (C2), 147.8 (C6), 140.1 (C-Ar), 136.6 (C3), 136.3 (C4), 135.7 (C-Ar), 133.3 (C-Ar), 127.4 (C-Ar), 123.5 (C5), 46.6 (C8), 26.7 (C7), 17.2 (C11 or C10), 16.8 (C12), 16.1 (C10 or C11); **IR** (solid) (cm^{-1}): 2925, 1700, 1480, 1445, 1116, 1029, 931, 805, 722.

(2-Methyl-6-(trifluoromethyl)pyridin-3-yl)methanol (26)

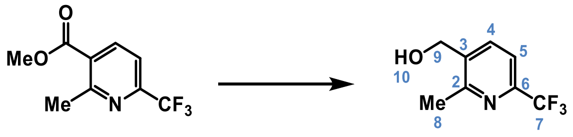

$LiAlH_4$ (2 M in THF, 1.37 mL, 2.74 mmol, 1.0 eq.) was added dropwise to a solution of methyl 2-methyl-6-(trifluoromethyl)nicotinate (0.600 g, 2.74 mmol, 1.0 eq.) in anhydrous THF (11.0 mL, 0.25 M) at 0 ˚C. The solution was stirred for 0.5 h at rt after which TLC (30% ethyl acetate in pentane) indicated full consumption of the starting material. The reaction solution was quenched by dropwise addition of 5% w/v aq. potassium sodium tartrate (Rochelle's salt, 5 mL) at 0 ˚C and the reaction mixture was stirred for a further 10 min at rt. The reaction mixture was extracted with ethyl acetate (3 × 20 mL); the combined organic extracts were dried using Mg_2SO_4, filtered and concentrated *in vacuo* to yield a crude oil. This oil was purified by silica gel column chromatography (elution with 20–50% ethyl acetate in pentane) to give the title product as a colourless solid (0.502 g, 96%).

M.p.: 40–42 °C (from ethyl acetate); **HRMS** (ESI+) 192.0636 [M+H]+, expected 192.0631; **^{1}H NMR** (400 MHz, $CDCl_3$) δ: 7.92 (1H, d, *J* = 7.9 Hz, H-4), 7.54 (1H, d, *J* = 7.9 Hz, H-5), 4.78 (2H, s, H-9), 2.56 (3H, s, H-8), 2.26 (1H, s, H-10); **^{13}C NMR** (101 MHz, $CDCl_3$) δ: 156.9 (C2), 146.2 (q, *J* = 34.2 Hz, C6), 137.7 (C3), 135.2 (C4), 121.8 (q, *J* = 273.8 Hz, C7), 118.2 (d, *J* = 2.9 Hz, C5), 61.6 (C9), 21.7 (C8); **^{19}F NMR** (376 MHz, $CDCl_3$) δ: −67.8 (s); **IR** (solid) (cm^{-1}): 3319, 1424, 1345, 1189, 1175, 1136, 1121, 1099, 1056, 839.

3-(2-Methyl-6-(trifluoromethyl)pyridin-3-yl)-1-(2,3,4,5,6-pentamethylphenyl)propan-1-one (P26)

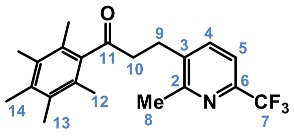

Prepared according to **General Procedure A** with (2-methyl-6-(trifluoromethyl)pyridin-3-yl)methanol (57.4 mg, 0.3 mmol, 1 eq.) and *tert*-amyl alcohol (0.3 mL, 1 M) at 23 ˚C. The crude product was purified

by silica gel column chromatography (elution with 4–10% ethyl acetate in pentane) to give the title product as a colourless solid (103.7 mg, 95%).

M.p.: 112–114 °C (from ethyl acetate); **HRMS** (ESI$^+$) 364.1871 [M+H]$^+$, expected 364.1883; **^{1}H NMR** (400 MHz, $CDCl_3$) δ: 7.69 (1H, d, *J* = 7.9 Hz, H-4), 7.45 (1H, d, *J* = 7.9 Hz, H-5), 3.14 (2H, t, *J* = 7.4 Hz, H-9), 3.07–2.89 (2H, m, H-10), 2.64 (3H, s, H-8), 2.23 (3H, s, H-14), 2.17 (6H, s, H-12 or H-13), 2.03 (6H, s, H-13 or H-12); **^{13}C NMR** (101 MHz, $CDCl_3$) δ: 209.9 (C11), 158.0 (C2), 145.6 (q, *J* = 34.3 Hz, C6), 140.0 (C-Ar), 137.9 (C3), 137.7 (C4), 136.0 (C-Ar), 133.4 (C-Ar), 127.3 (C-Ar), 121.8 (q, *J* = 273.8 Hz, C7), 118.1 (d, *J* = 2.8 Hz, C5), 44.7 (C10), 26.2 (C9), 22.4 (C8), 17.2 (C13 or C12), 16.8 (C14), 16.1 (C12 or C13); **^{19}F NMR** (376 MHz, $CDCl_3$) δ: −67.8 (s); **IR** (solid) (cm^{-1}): 1696, 1409, 1341, 1188, 1176, 1131, 1113, 934, 857, 697.

Unsuccessful Alcohol Substrates at 23 °C (which are not exemplified at 85 °C)

Commentary: A range of alcohols were found to be incompatible with the reaction conditions at 23 °C (**Supplementary Figure 2**).

Procedure: Experiments prepared according to **General Procedure A.**

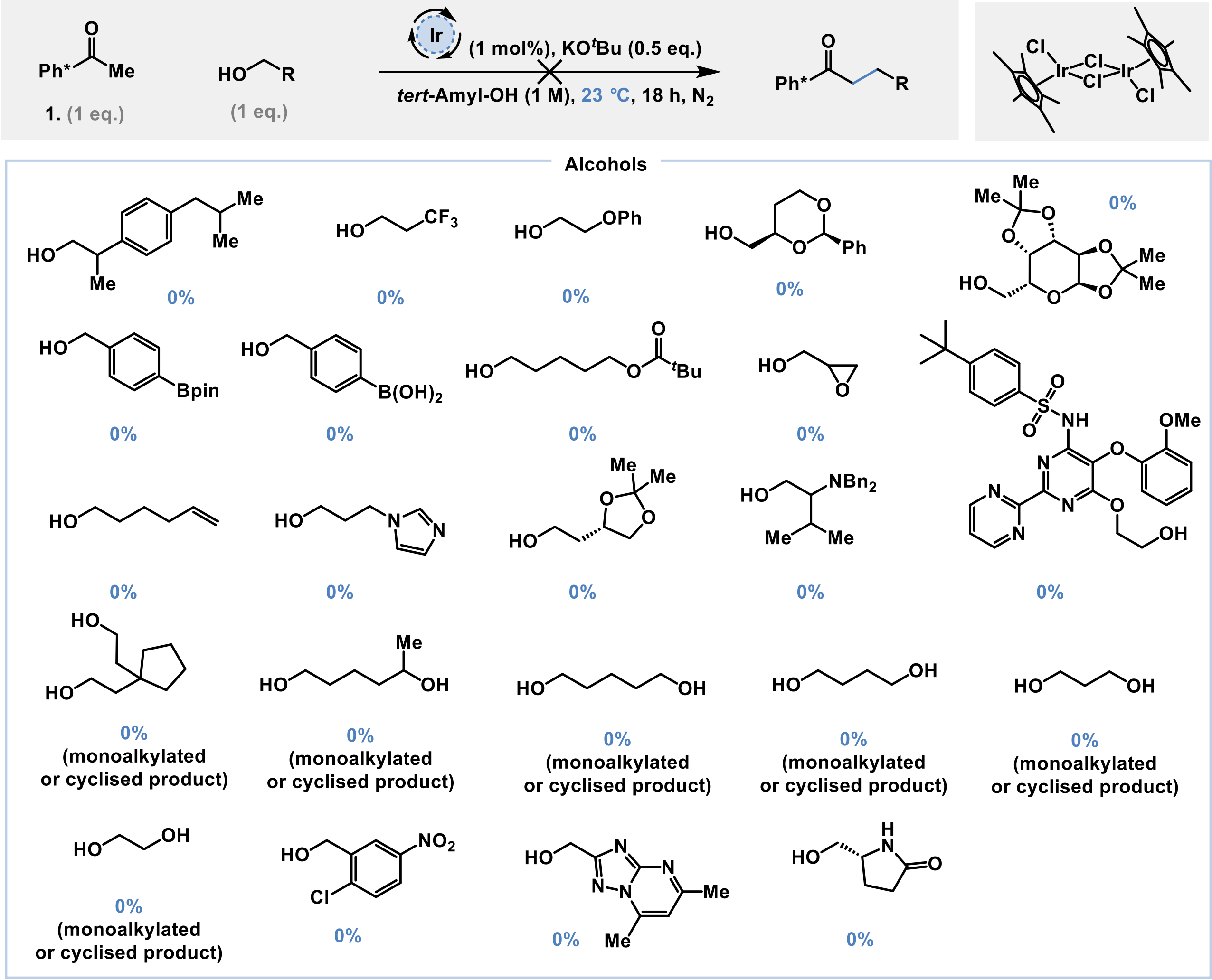

Supplementary Figure 2. Unsuccessful substrates.

Scope of Reaction with Ph* Methyl Ketone at 85 °C

1-(2,3,4,5,6-Pentamethylphenyl)-5-(pyridin-3-yl)pentan-1-one (P35)

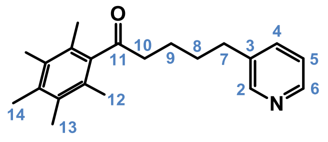

Prepared according to **General Procedure A** with 3-(pyridin-3-yl)propan-1-ol (38.7 µL, 0.3 mmol, 1 eq.) and *tert*-amyl alcohol (0.3 mL, **1 M**) at 85 ˚C. The crude product was purified by silica gel column chromatography (elution with 0–10% ethyl acetate in pentane + 1% triethylamine) to give the title product as a colourless solid (71.5 mg, 77%).

Prepared according to **General Procedure A** with 3-(pyridin-3-yl)propan-1-ol (38.7 µL, 0.3 mmol, 1 eq.) and *tert*-amyl alcohol (0.1 mL, **3 M**) at 85 ˚C. The crude product was purified by silica gel column chromatography (elution with 0–10% ethyl acetate in pentane + 1% triethylamine) to give the title product as a colourless solid (48.4 mg, 52%).

M.p.: 34–36 °C (from ethyl acetate); **HRMS** (ESI^+) 332.1995 $[M+Na]^+$, expected 332.1985; ^{1}**H NMR** (400 MHz, $CDCl_3$) δ: 8.45 (2H, app d, *J* = 6.3 Hz, H-2, H-6), 7.50 (1H, dt, *J* = 7.8, 2.0 Hz, H-4), 7.21 (1H, dd, *J* = 7.8, 4.7 Hz, H-5), 2.70 (2H, t, *J* = 7.0 Hz, H-10), 2.66 (2H, t, *J* = 7.3 Hz, H-7), 2.23 (3H, s, H-14), 2.18 (6H, s, H-12 or H-13), 2.08 (6H, s, H-13 or H-12), 1.75 (4H, tddd, *J* = 14.3, 12.4, 7.2, 3.4 Hz, H-8, H-9); ^{13}**C NMR** (101 MHz, $CDCl_3$) δ: 211.7 (C11), 150.1 (C2), 147.5 (C6), 140.7 (C-Ar), 137.5 (C3), 135.9 (C4), 135.5 (C-Ar), 133.2 (C-Ar), 127.4 (C-Ar), 123.4 (C5), 45.4 (C10), 33.1 (C7), 30.8 (C8), 22.9 (C9), 17.3 (C13 or C12), 16.8 (C14), 16.1 (C12 or C13); **IR** (solid) (cm^{-1}): 1697, 1422, 1576, 1479, 1457, 1117, 1026, 795, 733, 712.

(2-(Piperidin-1-yl)thiazol-5-yl)methanol (36)

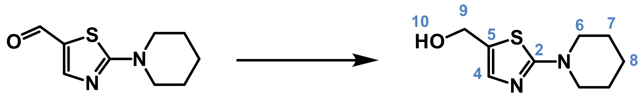

$NaBH_4$ (72.0 mg, 1.90 mmol, 1.0 eq.) was added portionwise to a solution of 2-(piperidin-1-yl)thiazole-5-carbaldehyde (0.400 g, 1.90 mmol, 1.0 eq.) in methanol (9.5 mL, 0.2 M) at 0 ˚C. The solution was stirred for 2 h at rt after which TLC (30% ethyl acetate in pentane) indicated full consumption of the starting material. The reaction mixture was concentrated *in vacuo* and extracted with ethyl acetate (3 × 20 mL); the combined organic extracts were dried using Mg_2SO_4, filtered and concentrated *in vacuo* to yield a crude oil. This oil was purified by silica gel column chromatography (elution with 70% ethyl acetate in pentane+ 1% triethylamine) to give the title product as a colourless solid (320 mg, 85%).

M.p.: 50–52 °C (from ethyl acetate); **HRMS** (ESI+) 199.0895 [M+H]+, expected 199.0900; **^{1}H NMR** (400 MHz, $CDCl_3$) δ: 6.93 (1H, s, H-4), 4.62 (2H, d, *J* = 0.8 Hz, H-9), 3.46–3.38 (4H, m, H-6), 2.59 (1H, s, H-10), 1.64 (6H, qt, *J* = 3.9, 2.9 Hz, H-7, H-8); **^{13}C NMR** (101 MHz, $CDCl_3$) δ: 172.9 (C2), 137.7 (C4), 125.8 (C5), 57.9 (C9), 49.6 (C6), 25.2 (C7), 24.2 (C8); **IR** (solid) (cm^{-1}): 3232, 1524, 1448, 1386, 1283, 1249, 1152, 1117, 1033, 847.

1-(2,3,4,5,6-Pentamethylphenyl)-3-(2-(piperidin-1-yl)thiazol-5-yl)propan-1-one (P36)

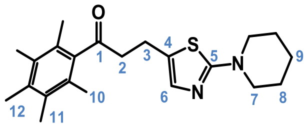

Prepared according to **General Procedure A** with (2-(piperidin-1-yl)thiazol-5-yl)methanol (63.7 mg, 0.3 mmol, 1 eq.) and *tert*-amyl alcohol (0.3 mL, 1 M) at 85 ˚C. The crude product was purified by silica gel column chromatography (elution with 20% ethyl acetate in pentane + 1% triethylamine) to give the title product as a colourless solid (75.1 mg, 65%).

M.p.: 76–78 °C (from ethyl acetate); **HRMS** (ESI+) 371.2141 [M+H]+, expected 371.2152; **^{1}H NMR** (400 MHz, $CDCl_3$) δ: 6.87 (1H, s, H-6), 3.40 (4H, t, *J* = 5.0 Hz, H-7), 3.08 (2H, t, *J* = 7.2 Hz, H-3), 2.96 (2H, t, *J* = 6.9 Hz, H-2), 2.23 (3H, s, H-12), 2.18 (6H, s, H10 or H-11), 2.07 (6H, s, H-11 or H-10), 1.71–1.57 (6H, m, H-8 or H-9); **^{13}C NMR** (101 MHz, $CDCl_3$) δ: 210.5 (C1), 171.4 (C5), 140.2 (C-Ar), 136.0 (C6), 135.7 (C-Ar), 133.3 (C-Ar), 127.5 (C-Ar), 125.3 (C4), 49.7 (C7), 46.8 (C2), 25.2 (C8), 24.3 (C9), 21.0 (C3), 17.3 (C11 or C10), 16.8 (C12), 16.1 (C10 or C11); **IR** (solid) (cm^{-1}): 3017, 1700, 1525, 1448, 1306, 1263, 1252, 1155, 1117, 856.

(4-Methyl-2-phenyloxazol-5-yl)methanol (37)

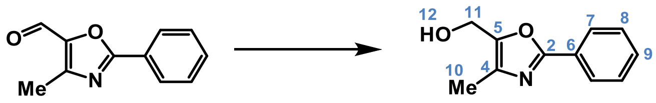

$NaBH_4$ (80.8 mg, 2.14 mmol, 1.0 eq.) was added portionwise to a solution of 4-methyl-2-phenyloxazole-5-carbaldehyde (0.400 g, 2.14 mmol, 1.0 eq.) in methanol (10.7 mL, 0.2 M) at 0 ˚C. The solution was stirred for 2 h at rt after which TLC (30% ethyl acetate in pentane) indicated full consumption of the starting material. The reaction mixture was concentrated *in vacuo* and extracted with ethyl acetate (3 × 20 mL); the combined organic extracts were dried using Mg_2SO_4, filtered and concentrated *in vacuo* to yield a crude oil. This oil was purified by silica gel column chromatography (elution with 30% ethyl acetate in pentane) to give the title product as a colourless solid (0.237 g, 58%).

M.p.: 96–98 °C (from ethyl acetate); **HRMS** (ESI+) 190.0859 [M+H]+, expected 190.0863; **^{1}H NMR** (400 MHz, $CDCl_3$) δ: 8.04–7.92 (2H, m, H-7), 7.42 (3H, app dd, *J* = 5.2, 2.1 Hz, H-8, H-9), 4.60 (2H, s, H-11), 3.94 (1H, br s, H-12), 2.38 (3H, s, H-10); **^{13}C NMR** (101 MHz, $CDCl_3$) δ: 160.1 (C2), 145.4 (C5), 135.6 (C6), 130.3 (C9), 128.9 (C8), 127.4 (C4), 126.2 (C7), 55.9 (C11), 10.4 (C10); **IR** (solid) (cm^{-1}): 3232, 1732, 1554, 1485, 1108, 794, 780, 719, 704, 692.

3-(4-Methyl-2-phenyloxazol-5-yl)-1-(2,3,4,5,6-pentamethylphenyl)propan-1-one (P37)

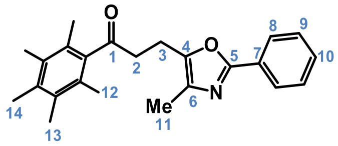

Prepared according to **General Procedure A** with (4-methyl-2-phenyloxazol-5-yl)methanol (56.8 mg, 0.3 mmol, 1 eq.) and *tert*-amyl alcohol (0.3 mL, 1 M) at 85 ˚C. The crude product was purified by silica gel column chromatography (elution with 5% ethyl acetate in pentane) to give the title product as a colourless solid (90.8 mg, 84%).

M.p.: 100–102 °C (from ethyl acetate); **HRMS** (ESI+) 362.2103 [M+H]+, expected 362.2115; **^{1}H NMR** (400 MHz, $CDCl_3$) δ: 8.00–7.92 (2H, m, H-8), 7.49–7.36 (3H, m, H-9, H-10), 3.12 (2H, t, *J* = 7.0 Hz, H-2), 2.92 (2H, t, *J* = 7.0 Hz, H-3), 2.42 (3H, s, H-11), 2.22 (3H, s, H-13), 2.16 (6H, s, H-12 or H-13), 2.05 (6H, s, H-13 or H-12); **^{13}C NMR** (101 MHz, $CDCl_3$) δ: 211.2 (C1), 159.4 (C5), 144.1 (C4), 140.5 (C-Ar), 135.5 (C-Ar), 134.9 (C7), 133.1 (C-Ar), 129.8 (C10), 128.8 (C9), 128.0 (C6), 127.5 (C-Ar), 126.0 (C8), 44.3 (C2), 19.5 (C3), 17.1 (C13 or C12), 16.8 (C14), 16.0 (C12 or C13), 10.3 (C11); **IR** (solid) (cm^{-1}): 1703, 1264, 1134, 1105, 1067, 947, 811, 780, 715, 697.

(3-(3-Methyl-1,2,4-oxadiazol-5-yl)phenyl)methanol (38)

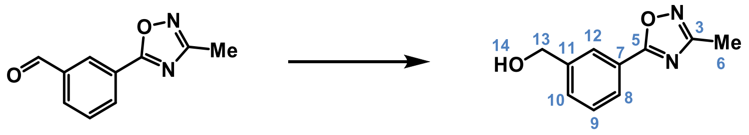

$NaBH_4$ (80.4 mg, 2.13 mmol, 1.0 eq.) was added portionwise to a solution of 3-(3-methyl-1,2,4-oxadiazol-5-yl)benzaldehyde (0.400 g, 2.13 mmol, 1.0 eq.) in methanol (10.7 mL, 0.2 M) at 0 ˚C. The solution was stirred for 2 h at rt after which TLC (30% ethyl acetate in pentane) indicated full consumption of the starting material. The reaction mixture was concentrated *in vacuo* and extracted with ethyl acetate (3 × 20 mL); the combined organic extracts were dried using Mg_2SO_4, filtered and concentrated *in vacuo* to yield a crude oil. This oil was purified by silica gel column chromatography (elution with 20–70% ethyl acetate in pentane) to give the title product as a colourless solid (311 mg, 77%).

M.p.: 62–64 °C (from ethyl acetate); **HRMS** (ESI+) 191.0811 [M+H]+, expected 191.0815; **^{1}H NMR** (400 MHz, $CDCl_3$) δ: 8.08 (1H, dt, *J* = 1.7, 0.9 Hz, H-12), 7.99 (1H, dt, *J* = 7.7, 1.5 Hz, H-8), 7.57 (1H, ddd, *J* = 7.6, 1.9, 1.0 Hz, H-10), 7.48 (1H, t, *J* = 7.7 Hz, H-9), 4.76 (2H, s, H-13), 2.54–2.45 (4H, m, H-6, H-14); **^{13}C NMR** (101 MHz, $CDCl_3$) δ: 175.5 (C5), 167.9 (C3), 142.3 (C11), 131.2 (C10), 129.4 (C9), 127.2 (C8), 126.4 (C12), 124.3 (C7), 64.5 (C13), 11.8 (C6); **IR** (solid) (cm^{-1}): 3346, 1603, 1567, 1443, 1397, 1344, 1039, 744, 711, 687.

3-(3-(3-Methyl-1,2,4-oxadiazol-5-yl)phenyl)-1-(2,3,4,5,6-pentamethylphenyl)propan-1-one (P38)

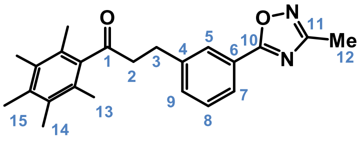

Prepared according to **General Procedure A** with (3-(3-methyl-1,2,4-oxadiazol-5-yl)phenyl)methanol (57.1 mg, 0.3 mmol, 1 eq.) and *tert*-amyl alcohol (0.3 mL, 1 M) at 85 ˚C. The crude product was purified by silica gel column chromatography (elution with 5% ethyl acetate in pentane + 1% triethylamine) to give the title product as a colourless solid (57.0 mg, 52%).

M.p.: 110–112 °C (from ethyl acetate); **HRMS** (ESI+) 363.2057 [M+H]+, expected 363.2067; **^{1}H NMR** (400 MHz, $CDCl_3$) δ: 7.98 (1H, t, *J* = 1.8 Hz, H-5), 7.94 (1H, dt, *J* = 7.5, 1.5 Hz, H-7), 7.50 (1H, dt, *J* = 7.8, 1.6 Hz, H-9), 7.44 (1H, app t, *J* = 7.6 Hz, H-8), 3.15 (2H, t, *J* = 7.7 Hz, H-3), 3.04 (2H, ddd, *J* = 8.3, 6.8, 1.0 Hz, H-2), 2.47 (3H, s, H-12), 2.22 (3H, s, H-15), 2.16 (6H, s, H-13 or H-14), 2.03 (6H, s, H-14 or H-13); **^{13}C NMR** (101 MHz, $CDCl_3$) δ: 210.5 (C1), 175.6 (C10), 167.9 (C11), 142.4 (C4), 140.3 (C-Ar), 135.7 (C-Ar), 133.3 (C9), 133.3 (C-Ar), 129.4 (C8), 128.1 (C5), 127.4 (C-Ar), 126.0 (C7), 124.4 (C6), 46.8 (C2), 29.3 (C3), 17.3 (C14 or C13), 16.8 (C15), 16.1 (C13 or C14), 11.9 (C12); **IR** (solid) (cm^{-1}): 1698, 1562, 1473, 1428, 1393, 1314, 1268, 1091, 764, 736.

(5-Methyl-3-phenylisoxazol-4-yl)methanol (39)

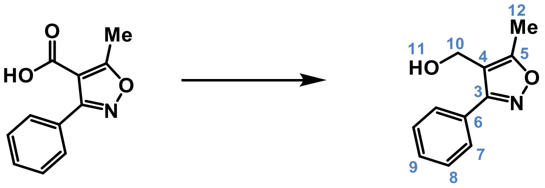

Oxalyl chloride (0.211 mL, 2.46 mmol, 1.0 eq.) was added dropwise to a solution of 5-methyl-3-phenylisoxazole-4-carboxylic acid (0.500 g, 2.46 mmol, 1.0 eq.) in CH_2Cl_2 (12.3 mL, 0.2 M) and DMF (1 drop) at rt. The solution was stirred 15 min (after which effervescence ceased), concentrated *in vacuo,* and redissolved in anhydrous THF (12.3 mL, 0.2 M). $LiAlH_4$ (2 M in THF, 1.37 mL, 2.74 mmol, 1.0 eq.)

was added dropwise to this solution at 0 ˚C, which was then stirred for 2 h at rt. The reaction solution was quenched by dropwise addition of 5% w/v aq. potassium sodium tartrate (Rochelle's salt, 5 mL) at 0 ˚C. The reaction mixture was extracted with ethyl acetate (3 × 20 mL); the combined organic extracts were dried using Mg_2SO_4, filtered and concentrated *in vacuo* to yield a crude oil. This oil was purified by silica gel column chromatography (elution with 20–50% ethyl acetate in pentane) to give the title product as a colourless oil (0.419 g, 90%).

HRMS (ESI$^+$) 190.0858 [M+H]$^+$, expected 190.0863; **^{1}H NMR** (400 MHz, $CDCl_3$) δ: 7.80–7.73 (2H, m, H-7), 7.49–7.40 (3H, m, H-8, H-9), 4.52 (2H, s, H-10), 2.43 (3H, s, H-12), 2.39 (1H, br s, H-11); **^{13}C NMR** (101 MHz, $CDCl_3$) δ: 168.7 (C5), 162.6 (C3), 129.9 (C9), 129.1 (C6), 129.0 (C8), 128.4 (C7), 113.0 (C4), 53.7 (C10), 11.2 (C12).

The spectral data matched that previously reported in the literature.[9]

3-(5-Methyl-3-phenylisoxazol-4-yl)-1-(2,3,4,5,6-pentamethylphenyl)propan-1-one (P39)

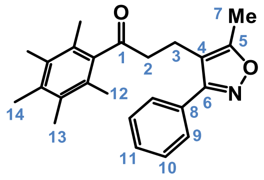

Prepared according to **General Procedure A** with (5-methyl-3-phenylisoxazol-4-yl)methanol (56.8 mg, 0.3 mmol, 1 eq.) and *tert*-amyl alcohol (0.3 mL, 1 M) at 85 ˚C. The crude product was purified by silica gel column chromatography (elution with 5% ethyl acetate in pentane) to give the title product as a colourless solid (93.7 mg, 86%).

M.p.: 92–94 °C (from ethyl acetate); **HRMS** (ESI$^+$) 362.2104 [M+H]$^+$, expected 362.2115; **^{1}H NMR** (400 MHz, $CDCl_3$) δ: 7.64–7.55 (2H, m, H-9), 7.43 (3H, app q, *J* = 2.6 Hz, H-10, H-11), 2.97 (2H, t, *J* = 7.2 Hz, H-3), 2.69 (2H, t, *J* = 7.2 Hz, H-2), 2.51 (3H, s, H-7), 2.19 (3H, s, H-14), 2.12 (6H, s, H-12 or H-13), 1.90 (6H, s, H-13 or H-12); **^{13}C NMR** (101 MHz, $CDCl_3$) δ: 210.4 (C1), 166.8 (C5), 162.2 (C6), 140.0 (C-Ar), 135.7 (C-Ar), 133.2 (C-Ar), 130.1 (C8), 129.6 (C11), 129.0 (C10), 127.9 (C9), 127.3 (C-Ar), 111.8 (C4), 44.7 (C2), 17.0 (C13 or C12), 16.8 (C14), 16.3 (C3), 16.0 (C12 or C13), 11.3 (C7); **IR** (solid) (cm^{-1}): 1687, 1404, 1264, 1076, 915, 815, 780, 723, 702, 673.

Pyridazin-4-ylmethanol (40)

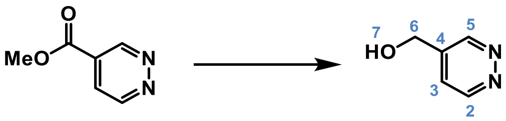

$NaBH_4$ (0.685 g, 18.1 mmol, 5.0 eq.) was added portionwise to a solution of methyl pyridazine-4-carboxylate (0.500 g, 3.62 mmol, 1.0 eq.) in methanol (45.3 mL, 0.08 M) at 0 ˚C. The solution was stirred for 16 h at rt and then quenched by addition of sat. aq. ammonium chloride (10 mL). The reaction mixture was concentrated *in vacuo* and extracted with chloroform/iso-propanol (3:1, 3 × 50 mL); the combined organic extracts were dried using Mg_2SO_4, filtered and concentrated *in vacuo* to yield a crude oil. This oil was purified by silica gel column chromatography (elution with 5% methanol in CH_2Cl_2 +1% triethylamine) to give the title product as a brown solid (56.8 mg, 14%).

N.b. Pyridazin-4-ylmethanol was found to degrade in triethylamine/methanol solution heated to 40 ˚C for >2 h (on concentration *in vacuo* using a rotary evaporator after purification by silica gel column chromatography). ^{1}H and ^{13}C NMR data has been acquired from a sample of pyridazin-4-ylmethanol concentrated *in vacuo* briefly which contains Et_3NHCl (from protonation of residual NEt_3 by trace HCl from $CDCl_3$).

M.p.: 75–77 °C (from methanol); **HRMS** (ESI$^+$) 111.0553 $[M+H]^+$, expected 111.0553; **^{1}H NMR** (400 MHz, $CDCl_3$) δ: 9.21–9.13 (1H, m, H-5), 9.11 (1H, dd, *J* = 5.4, 1.3 Hz, H-2), 7.58–7.52 (1H, m, H-3), 4.82 (2H, s, H-6); **^{13}C NMR** (101 MHz, $CDCl_3$) δ: 151.3 (C2), 150.3 (C3), 141.4 (C4), 124.1 (C3), 61.1 (C6).

The spectral data matched that previously reported in the literature.[10,11]

1-(2,3,4,5,6-Pentamethylphenyl)-3-(pyridazin-4-yl)propan-1-one (P40)

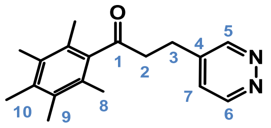

Prepared according to **General Procedure A** with pyridazin-4-ylmethanol (33.1 mg, 0.3 mmol, 1 eq.) and *tert*-amyl alcohol (0.3 mL, 1 M) at 85 ˚C. The crude product was purified by silica gel column chromatography (elution with 0–2% methanol in CH_2Cl_2 + 1% triethylamine) to give the title product as a pale-yellow solid (37.8 mg, 45%).

M.p.: 128–130 °C (from ethyl acetate); **HRMS** (ESI$^+$) 305.1639 $[M+Na]^+$, expected 305.1624; **^{1}H NMR** (400 MHz, $CDCl_3$) δ: 9.18–9.12 (1H, m, H-5), 9.08 (1H, d, *J* = 5.3 Hz, H-6), 7.41 (1H, dd, *J* = 5.4, 2.4 Hz, H-7), 3.08 (2H, dd, *J* = 7.8, 4.5 Hz, H-3), 3.02 (2H, dd, *J* = 8.0, 4.5 Hz, H-2), 2.22 (3H, s, H-10), 2.16 (6H, s, H-8 or H-9), 1.99 (6H, s, H-9 or H-8); **^{13}C NMR** (101 MHz, $CDCl_3$) δ: 209.3 (C1), 152.9 (C5), 151.2 (C6), 140.7 (C-Ar), 139.6 (C4), 136.1 (C-Ar), 133.4 (C-Ar), 127.3 (C-Ar), 126.5 (C7), 44.9 (C2), 26.3 (C3), 17.2 (C9 or C8), 16.8 (C10), 16.1 (C8 or C9); **IR** (thin film) (cm^{-1}): 1700, 1589, 1382, 1118, 932, 907, 901, 846, 756, 609.

(4-Methyl-2-(piperidin-1-yl)thiazol-5-yl)methanol (41)

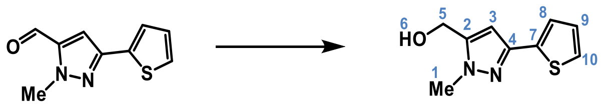

$NaBH_4$ (78.7 mg, 2.08 mmol, 1.0 eq.) was added portionwise to a solution of 1-methyl-3-(thiophen-2-yl)-1*H*-pyrazole-5-carbaldehyde (0.400 g, 2.08 mmol, 1.0 eq.) in methanol (10.4 mL, 0.2 M) at 0 ˚C. The solution was stirred for 2 h at rt after which TLC (30% ethyl acetate in pentane) indicated full consumption of the starting material. The reaction mixture was concentrated *in vacuo* and extracted with ethyl acetate (3 × 20 mL); the combined organic extracts were dried using Mg_2SO_4, filtered and concentrated *in vacuo* to yield a crude oil. This oil was purified by silica gel column chromatography (elution with 50–70% ethyl acetate in pentane) to give the title product as a colourless solid (335 mg, 83%).

M.p.: 76–78 °C (from ethyl acetate); **HRMS** (ESI$^+$) 195.0579 [M+H]$^+$, expected 195.0587; **^{1}H NMR** (400 MHz, $CDCl_3$) δ: 7.24 (1H, dd, *J* = 3.6, 1.2 Hz, H-10), 7.22 (1H, dd, *J* = 5.1, 1.2 Hz, H-8), 7.03 (1H, dd, *J* = 5.1, 3.5 Hz, H-9), 6.31 (1H, s, H-3), 4.60 (2H, s, H-5), 3.82 (3H, s, H-1), 2.84 (1H, s, H-6); **^{13}C NMR** (101 MHz, $CDCl_3$) δ: 145.5 (C2), 143.0 (C4), 136.5 (C7), 127.6 (C9), 124.4 (C8), 123.5 (C10), 103.0 (C3), 55.5 (C5), 36.6 (C1); **IR** (solid) (cm^{-1}): 3337, 1380, 1286, 1161, 1024, 922, 848, 816, 733, 708.

3-(1-Methyl-3-(thiophen-2-yl)-1*H*-pyrazol-5-yl)-1-(2,3,4,5,6-pentamethylphenyl)propan-1-one (P41)

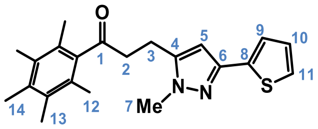

Prepared according to **General Procedure A** with (4-methyl-2-(piperidin-1-yl)thiazol-5-yl)methanol (58.3 mg, 0.3 mmol, 1 eq.) and *tert*-amyl alcohol (0.3 mL, 1 M) at 85 ˚C. The crude product was purified by silica gel column chromatography (elution with 10–20% ethyl acetate in pentane + 1% triethylamine) to give the title product as a colourless solid (65.1 mg, 60%).

M.p.: 116–118 °C (from ethyl acetate); **HRMS** (ESI$^+$) 367.1826 [M+H]$^+$, expected 367.1839; **^{1}H NMR** (400 MHz, $CDCl_3$) δ: 7.23 (1H, dd, *J* = 3.6, 1.2 Hz, H-11), 7.20 (1H, dd, *J* = 5.1, 1.2 Hz, H-9), 7.02 (1H, dd, *J* = 5.1, 3.6 Hz, H-10), 6.24 (1H, s, H-5), 3.88 (3H, s, H-7), 3.06 (4H, app s, H-2, H-3), 2.24 (3H, s, H-14), 2.19 (6H, s, H-12 or H-13), 2.08 (6H, s, H-13 or H-12); **^{13}C NMR** (101 MHz, $CDCl_3$) δ: 209.9 (C1), 145.5 (C4), 143.1 (C6), 140.0 (C-Ar), 137.0 (C8), 136.0 (C-Ar), 133.4 (C-Ar), 127.5 (C10), 127.4 (C-Ar), 124.2 (C9), 123.2 (C11), 101.6 (C5), 44.1 (C2), 36.4 (C7), 19.5 (C3), 17.3 (C13 or C12), 16.8 (C14), 16.1 (C12 or C13); **IR** (solid) (cm^{-1}): 1694, 1383, 1115, 934, 850, 813, 781, 762, 697, 681.

1-(2,3,4,5,6-Pentamethylphenyl)-3-(tetrahydrofuran-2-yl)propan-1-one (P42)

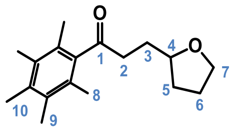

Prepared according to **General Procedure A** with (tetrahydrofuran-2-yl)methanol (29.0 µL, 0.3 mmol, 1 eq.) and *tert*-amyl alcohol (0.3 mL, 1 M) at 85 ˚C. The crude product was purified by silica gel column chromatography (elution with 8% diethyl ether in pentane) to give the title product as a colourless solid (57.4 mg, 70%).

The title product was not observed by quantitative ^{1}H NMR spectroscopy following **General Procedure A** with (tetrahydrofuran-2-yl)methanol (29.0 µL, 0.3 mmol, 1 eq.) and *tert*-amyl alcohol (0.3 mL, 1 M) at 23 ˚C.

M.p.: 46–48 °C (from diethyl ether); **HRMS** (ESI^{+}) 275.2006 [M+H]$^{+}$, expected 275.2006; **^{1}H NMR** (400 MHz, $CDCl_3$) δ: 3.96–3.79 (2H, m, H-4, H-7_a), 3.71 (1H, td, *J* = 8.0, 6.4 Hz, H-7_b), 2.90–2.70 (2H, m, H-2_a, H-2_b), 2.23 (3H, s, H-10), 2.18 (6H, s, H-8 or H-9), 2.10 (6H, s, H-9 or H-8), 2.07–1.81 (5H, m, H-3_a, H-3_b, H-5_a, H-6_a, H-6_b), 1.56–1.45 (1H, m, H-5_b); **^{13}C NMR** (101 MHz, $CDCl_3$) δ: 212.0 (C1), 140.8 (C-Ar), 135.4 (C-Ar), 133.1 (C-Ar), 127.4 (C-Ar), 78.4 (C4), 67.8 (C7), 42.4 (C2), 31.5 (C5), 29.2 (C3), 25.8 (C6), 17.3 (C9 or C8), 16.8 (C10), 16.1 (C8 or C9); **IR** (solid) (cm^{-1}): 1699, 1264, 1104, 1068, 1026, 926, 809, 705

2-(1-Benzylpiperidin-4-yl)-1-(2,3,4,5,6-pentamethylphenyl)ethan-1-one (P43)

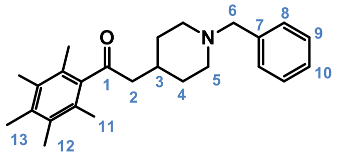

Prepared according to **General Procedure A** with 1-benzylpiperidin-4-ol (57.4 mg, 0.3 mmol, 1 eq.) and *tert*-amyl alcohol (0.3 mL, 1 M) at 85 ˚C. The crude product was purified by silica gel column chromatography (elution with 0–20% ethyl acetate in pentane) to give the title product as a pale-yellow solid (91.0 mg, 83%).

M.p.: 112–114 °C (from ethyl acetate); **HRMS** (ESI^{+}) 364.2636 [M+H]$^{+}$, expected 364.2635; **^{1}H NMR** (400 MHz, $CDCl_3$) δ: 7.33–7.29 (4H, m, H-8, H-9), 7.27–7.20 (1H, m, H-10), 3.49 (2H, s, H-6), 2.87 (2H, dt, *J* = 11.8, 3.4 Hz, H-5_a), 2.61 (2H, d, *J* = 6.5 Hz, H-2), 2.22 (3H, s, H-13), 2.17 (6H, s, H-11 or H-12), 2.09–2.00 (3H, m, H-3, H-5_b), 2.08 (6H, s, H-12 or H-11), 1.88–1.78 (2H, m, H-4_a), 1.39–1.22 (2H, m, H-4_b); **^{13}C NMR** (101 MHz, $CDCl_3$) δ: 211.0 (C1), 140.7 (C-Ar), 138.7 (C7), 135.5 (C-Ar), 133.2 (C-Ar), 129.3 (C8), 128.3

(C9), 127.4 (C-Ar), 127.0 (C10), 63.6 (C6), 53.8 (C5), 52.4 (C2), 32.4 (C4), 30.6 (C3), 17.1 (C12 or C11), 16.8 (C13), 16.1 (C11 or C12).

The spectral data matched that previously reported in the literature.[12]

tert-butyl 4-(3-oxo-3-(2,3,4,5,6-pentamethylphenyl)propyl)piperidine-1-carboxylate (P44)

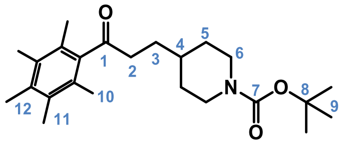

Prepared according to **General Procedure A** with tert-butyl 4-(hydroxymethyl)piperidine-1-carboxylate (64.6 mg, 0.3 mmol, 1 eq.) and _tert_-amyl alcohol (0.3 mL, 1 M) at 85 ˚C. The crude product was purified by silica gel column chromatography (elution with 0–20% ethyl acetate in pentane + 1% triethylamine) to give the title product as a colourless solid (105.6 mg, 91%).

M.p.: 105–107 °C (from ethyl acetate); **HRMS** (ESI$^+$) 388.2836 [M+H]$^+$, expected 388.2846; **^{1}H NMR** (400 MHz, $CDCl_3$) δ: 4.26–3.94 (2H, m, H-6_a), 2.75–2.58 (4H, m, H-2, H-6_b), 2.23 (3H, s, H-12), 2.18 (6H, s, H-10 or H-11), 2.09 (6H, s, H-11 or H-10), 1.73–1.63 (4H, m, H-3, H-5_a), 1.52–1.38 (10H, m, H-4, H-9), 1.12 (2H, qd, J = 12.6, 4.4 Hz, H-5_b); **^{13}C NMR** (101 MHz, $CDCl_3$) δ: 212.0 (C1), 155.0 (C7), 140.9 (C-Ar), 135.6 (C-Ar), 133.2 (C-Ar), 127.3 (C-Ar), 79.4 (C8), 44.7–43.4 (br s, C6), 42.8 (C2), 35.6 (C4), 32.2 (C5), 29.9 (C3), 28.6 (C9), 17.3 (C11 or C10), 16.8 (C12), 16.1 (C10 or C11); **IR** (solid) (cm^{-1}): 1688, 1414, 1364, 1276, 1240, 1185, 1164, 1124, 1098, 876.

tert-Butyl 3-(3-oxo-3-(2,3,4,5,6-pentamethylphenyl)propyl)azetidine-1-carboxylate (P45)

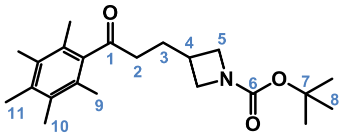

Prepared according to **General Procedure A** with _tert_-butyl 3-(hydroxymethyl)azetidine-1-carboxylate (56.2 mg, 0.3 mmol, 1 eq.) and _tert_-amyl alcohol (0.3 mL, 1 M) at 85 ˚C. The crude product was purified by silica gel column chromatography (elution with 4% ethyl acetate in toluene) to give the title product as a colourless solid (48.4 mg, 45%).

M.p.: 78–80 °C (from toluene); **HRMS** (ESI$^+$) 360.2520 [M+H]$^+$, expected 360.2533; **^{1}H NMR** (400 MHz, $CDCl_3$) δ: 4.01 (2H, t, J = 8.3 Hz, H-5_a), 3.56 (2H, dd, J = 8.4, 5.4 Hz, H-5_b), 2.62 (H, app t, J = 7.3 Hz, H-2, H-4), 2.23 (3H, s, H-11), 2.18 (6H, s, H-9 or H-10), 2.07 (6H, s, H-10 or H-9), 2.00 (2H, q, J = 7.5 Hz, H-3), 1.43 (9H, s, H-8); **^{13}C NMR** (101 MHz, $CDCl_3$) δ: 211.1 (C1), 156.5 (C6), 140.5 (C-Ar), 135.7 (C-Ar), 133.3

(C-Ar), 127.3 (C-Ar), 79.5 (C7), 54.4 (C5), 42.7 (C2), 28.5 (C8), 28.3 (C4), 27.9 (C3), 17.3 (C10 or C9), 16.8 (C11), 16.1 (C9 or C10); **IR** (solid) (cm^{-1}): 1695, 1457, 1365, 1260, 1167, 1133, 1098, 807.

1-(2,3,4,5,6-Pentamethylphenyl)-3-(thiazol-2-yl)propan-1-one (P46)

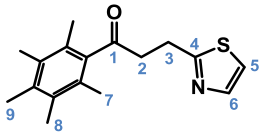

Prepared according to **General Procedure A** with thiazol-2-ylmethanol (56.2 mg, 0.3 mmol, 1 eq.) and *tert*-amyl alcohol (0.1 mL, 3 M) at 85 ˚C. The crude product was purified by silica gel column chromatography (elution with 0–4% ethyl acetate in pentane + 1% triethylamine) to give the title product as a colourless solid (22.4 mg, 26%).

The title product was not observed by quantitative ^{1}H NMR spectroscopy following **General Procedure A** with thiazol-2-ylmethanol (56.2 mg, 0.3 mmol, 1 eq.) and *tert*-amyl alcohol (0.3 mL, 1 M) at 23 ˚C.

M.p.: 72–74 °C (from ethyl acetate); **HRMS** (ESI^{+}) 310.1247 [M+Na]$^{+}$, expected 30.1236; **^{1}H NMR** (400 MHz, $CDCl_3$) δ: 7.67 (1H, d, *J* = 3.3 Hz, H-6), 7.21 (1H, d, *J* = 3.3 Hz, H-5), 3.46 (2H, t, *J* = 7.2 Hz, H-3), 3.23 (2H, t, *J* = 7.2 Hz, H-2), 2.23 (3H, s, H-9), 2.17 (6H, s, H-7 or H-8), 2.07 (6H, s, H-8 or H-7); **^{13}C NMR** (101 MHz, $CDCl_3$) δ: 209.9 (C1), 169.4 (C4), 142.4 (C6), 140.0 (C-Ar), 135.8 (C-Ar), 133.3 (C-Ar), 127.6 (C-Ar), 118.6 (C5), 44.9 (C2), 27.0 (C3), 17.3 (C8 or C7), 16.8 (C9), 16.1 (C7 or C8); **IR** (solid) (cm^{-1}): 3121, 1702, 1389, 1316, 1261, 1128, 1095, 904, 801, 746.

1-(2,3,4,5,6-Pentamethylphenyl)-4-(pyridin-2-yl)butan-1-one (P47)

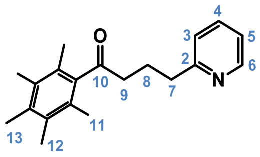

Prepared according to **General Procedure A** with 2-(pyridin-2-yl)ethan-1-ol (38.7 µL, 0.3 mmol, 1 eq.) and *tert*-amyl alcohol (0.1 mL, 3 M) at 85 ˚C. The crude product was purified by silica gel column chromatography (elution with 0–10% ethyl acetate in pentane + 1% triethylamine) to give the title product as a colourless solid (21.4 mg, 23%).

The title product was not observed by quantitative ^{1}H NMR spectroscopy following **General Procedure A** with 2-(pyridin-2-yl)ethan-1-ol (38.7 µL, 0.3 mmol, 1 eq.) and *tert*-amyl alcohol (0.1 mL, 3 M) at 23 ˚C.

M.p.: 42–44 °C (from ethyl acetate); **HRMS** (ESI+) 318.1843 [M+Na]+, expected 318.1828; **^{1}H NMR** (400 MHz, $CDCl_3$) δ: 8.52 (1H, ddd, *J* = 4.9, 1.9, 0.9 Hz, H-6), 7.60 (1H, td, *J* = 7.6, 1.9 Hz, H-4), 7.19 (1H, dt, *J* = 7.8, 1.1 Hz, H-3), 7.11 (1H, ddd, *J* = 7.5, 4.9, 1.2 Hz, H-5), 2.89 (2H, dd, *J* = 8.7, 6.8 Hz, H-7), 2.74 (2H, t, *J* = 7.3 Hz, H-9), 2.22 (3H, s, H-13), 2.20–2.13 (2H, m, H-8), 2.17 (6H, s, H-11 or H-12), 2.07 (6H, s, H-12 or H-11); **^{13}C NMR** (101 MHz, $CDCl_3$) δ: 211.8 (C10), 161.6 (C2), 149.4 (C6), 140.8 (C-Ar), 136.5 (C4), 135.5 (C-Ar), 133.2 (C-Ar), 127.4 (C-Ar), 122.9 (C3), 121.3 (C5), 45.0 (C9), 37.6 (C7), 23.4 (C8), 17.3 (C12 or C11), 16.8 (C13), 16.1 (C11 or C12); **IR** (solid) (cm^{-1}): 1698, 1590, 1474, 1458, 1437, 1403, 1115, 920, 807, 763.

1-(2,3,4,5,6-Pentamethylphenyl)-3-(pyrimidin-2-yl)propan-1-one (P48)

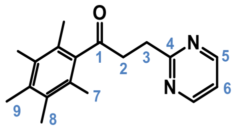

Prepared according to **General Procedure A** with pyrimidin-2-ylmethanol (27.5 µL, 0.3 mmol, 1 eq.) and *tert*-amyl alcohol (0.3 mL, 1 M) at 85 °C. The crude product was purified by silica gel column chromatography (elution with 0–15% ethyl acetate in pentane + 1% triethylamine) to give the title product as a colourless solid (59.2 mg, 70%).

The title product was not observed by quantitative ^{1}H NMR spectroscopy following **General Procedure A** with pyrimidin-2-ylmethanol (27.5 µL, 0.3 mmol, 1 eq.) and *tert*-amyl alcohol (0.1 mL, 3 M) at 23 °C.

M.p.: 72–74 °C (from ethyl acetate); **HRMS** (ESI+) 283.1809 [M+H]+, expected 283.1805; **^{1}H NMR** (400 MHz, $CDCl_3$) δ: 8.66 (2H, d, *J* = 4.9 Hz, H-5), 7.13 (1H, t, *J* = 4.9 Hz, H-6), 3.40 (2H, dd, *J* = 8.0, 6.6 Hz, H-3), 3.34–3.25 (2H, m, H-2), 2.22 (3H, s, H-9), 2.18 (6H, s, H-7 or H-8), 2.14 (6H, s, H-8 or H-7); **^{13}C NMR** (101 MHz, $CDCl_3$) δ: 210.6 (C1), 170.1 (C4), 157.0 (C5), 140.4 (C-Ar), 135.5 (C-Ar), 133.1 (C-Ar), 127.6 (C-Ar), 118.7 (C6), 43.3 (C2), 32.9 (C3), 17.3 (C8 or C7), 16.8 (C9), 16.1 (C7 or C8); **IR** (solid) (cm^{-1}): 2906, 1966, 1563, 1430, 1398, 1383, 1359, 1117, 913, 810.

5-(Benzylamino)-1-(2,3,4,5,6-pentamethylphenyl)pentan-1-one (P49)

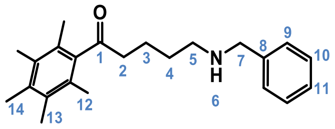

Prepared according to a modification to **General Procedure A Modification I** with 3-(benzylamino)propan-1-ol (49.6 mg, 0.3 mmol, 1 eq.) and *tert*-amyl alcohol (0.3 mL, 1 M) at 85 °C. The

crude product was purified by silica gel column chromatography (elution with 5% methanol in CH_2Cl_2 + 1% triethylamine) to give the title product as a yellow oil (93.0 mg, 92%).

The title product was not observed by quantitative ^{1}H NMR spectroscopy following **General Procedure A Modification I** with 3-(benzylamino)propan-1-ol (49.6 mg, 0.3 mmol, 1 eq.) and *tert*-amyl alcohol (0.3 mL, 1 M) at 23 ˚C**.**

HRMS (ESI$^+$) 338.2479 [M+H]$^+$, expected 338.2478; **^{1}H NMR** (400 MHz, $CDCl_3$) δ: 7.38–7.31 (4H, m, H-9, H-10), 7.28–7.25 (1H, m, H-11), 3.81 (2H, s, H-7), 3.01 (1H, s, H-6), 2.69 (4H, td, *J* = 7.1, 3.4 Hz, H-2, H-5), 2.21 (3H, s, H-14), 2.16 (6H, s, H-12 or H-13), 2.07 (6H, s, H-13 or H-12), 1.83–1.70 (2H, m, H-3), 1.63 (2H, ddt, *J* = 14.7, 11.5, 6.0 Hz, H-4); **^{13}C NMR** (101 MHz, $CDCl_3$) δ: 211.8 (C1), 140.7 (C-Ar), 139.3 (C8), 135.4 (C-Ar), 133.1 (C-Ar), 128.5 (C9), 128.5 (C10), 127.4 (C-Ar), 127.4 (C11), 53.7 (C7), 48.9 (C5), 45.4 (C2), 29.2 (C4), 21.0 (C3), 17.2 (C13 or C12), 16.7 (C14), 16.0 (C12 or C13); **IR** (thin film) (cm^{-1}): 1699, 1454, 1404, 1126, 915, 737, 700, 645.

Methyl 2-(4-(3-oxo-3-(2,3,4,5,6-pentamethylphenyl)propyl)phenyl)acetate (P50)

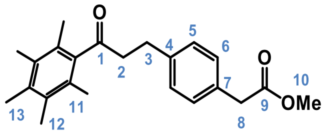

Prepared according to a modification to **General Procedure A Modification I** with 2-(4-(hydroxymethyl)phenyl)acetic acid (49.9 mg, 0.3 mmol, 1 eq.), powdered potassium *tert*-butoxide (50.4 mg, 0.9 mmol, **1.5 eq.**) and *tert*-amyl alcohol (0.3 mL, 1 M) at 85 ˚C. After 18 h the reaction vial was cooled to rt and 2 M HCl in diethyl ether (1.0 mL, 6 mmol, 6.66 eq.) and methanol (3 mL), were added. The reaction mixture was filtered through a short pad of silica gel and concentrated *in vacuo.* The crude product was purified by silica gel column chromatography (elution with 5% ethyl acetate in pentane) to give the title product as a colourless solid (62.3 mg, 59%).

M.p.: 38–40 °C (from ethyl acetate); **HRMS** (ESI$^+$) 353.2106 [M+H]$^+$, expected 353.2111; **^{1}H NMR** (400 MHz, $CDCl_3$) δ 7.20 (4H, s, H-5, H-6), 3.69 (3H, s, H-10), 3.60 (2H, s, H-8), 3.05 (2H, ddd, *J* = 7.6, 6.2, 2.1 Hz, H-3), 3.02–2.93 (2H, m, H-2), 2.23 (3H, s, H-13), 2.17 (6H, s, H-11 or H-12), 2.04 (6H, s, H-12 or H-11); **^{13}C NMR** (101 MHz, $CDCl_3$) δ 211.1 (C1), 172.3 (C9), 140.5 (C-Ar), 140.0 (C4), 135.6 (C-Ar), 133.2 (C-Ar), 131.8 (C7), 129.5 (C6), 128.8 (C5), 127.5 (C-Ar), 52.2 (C10), 47.1 (C2), 40.9 (C8), 29.1 (C3), 17.2 (C12 or C11), 16.8 (C13), 16.1 (C11 or C12); **IR** (thin film) (cm^{-1}): 1742, 1702, 1517, 1437, 1223, 1156, 1118, 1014, 762, 644.

4-(4-(3-(2-Chloro-10*H*-phenothiazin-10-yl)propyl)piperazin-1-yl)-1-(2,3,4,5,6-pentamethylphenyl)butan-1-one (P51)

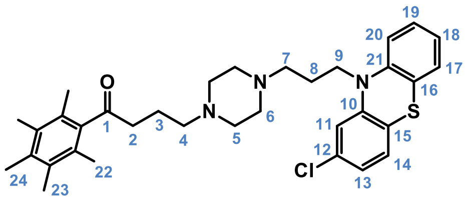

Prepared according to **General Procedure A** on 0.1 mmol scale with [Cp*IrCl$_2$]$_2$ (0.8 mg, 1 mol%), Ph* methyl ketone (19.0 mg, 0.1 mmol, 1 eq.), potassium *tert*-butoxide (5.6 mg, 0.05 mmol, 1 eq.), perphenazine (40.4 mg, 0.1 mmol, 1 eq.) and *tert*-amyl alcohol (0.1 mL, 1 M) at 85 ˚C. The crude product was purified by silica gel column chromatography (elution with 20–50% ethyl acetate in pentane + 2% triethylamine) to give the title product as a colourless solid (42.6 mg, 74%).

The title product was not observed by quantitative ^{1}H NMR spectroscopy following **General Procedure A** with perphenazine (40.4 mg, 0.1 mmol, 1 eq.) and *tert*-amyl alcohol (0.1 mL, 1 M) at 23 ˚C.

M.p.: 112–114 °C (from ethyl acetate); **HRMS** (ESI$^+$) 598.2625 [M+Na]$^+$, expected 598.2629; **^{1}H NMR** (400 MHz, CDCl$_3$) δ: 7.18–7.12 (1H, m, H-19), 7.11 (1H, dd, *J* = 7.6, 1.6 Hz, H-17), 7.01 (1H, d, *J* = 8.0 Hz, H-14), 6.96–6.81 (4H, m, H-11, H-13, H-18, H-20), 3.89 (2H, t, *J* = 6.9 Hz, H-9), 2.71 (2H, t, *J* = 7.2 Hz, H-2), 2.52–2.34 (8H, m, H-5, H-6), 2.46 (2H, t, *J* = 7.1 Hz, H-7), 2.40 (2H, t, *J* = 7.1 Hz, H-4), (3H, s, H-24), 2.17 (6H, s, H-22 or H-23), 2.09 (6H, s, H-23 or H-22), 2.00–1.83 (4H, m, H-3, H-8); **^{13}C NMR** (101 MHz, CDCl$_3$) δ: 211.8 (C1), 146.6 (C10), 144.6 (C21), 140.8 (C-Ar), 135.5 (C-Ar), 133.3 (C12), 133.2 (C-Ar), 128.0 (C14), 127.6 (C17), 127.5 (C19), 127.4 (C-Ar), 124.9 (C16), 123.6 (C15), 123.0 (C18), 122.3 (C13), 116.0 (C11), 115.9 (C20), 57.4 (C4), 55.7 (C7), 53.5 (C6), 53.1 (C5), 45.5 (C9), 43.3 (C2), 24.4 (C8), 20.5 (C3), 17.3 (C23 or C22), 16.8 (C24), 16.1 (C22 or C23); **IR** (thin film) (cm^{-1}): 1698, 1567, 1459, 1408, 1279, 1245, 1128, 918, 801, 752.

3-(2-Butyl-4-chloro-1-((2'-(1-trityl-1*H*-tetrazol-5-yl)-[1,1'-biphenyl]-4-yl)methyl)-1*H*-imidazol-5-yl)-1-(2,3,4,5,6-pentamethylphenyl)propan-1-one (P52)

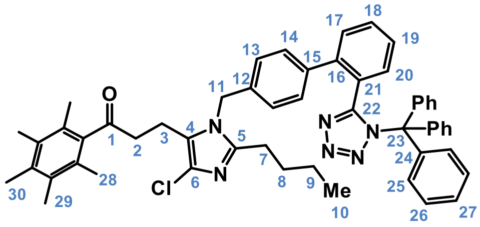

Prepared according to **General Procedure A** with trityl-Losartan (199.6 mg, 0.3 mmol, 1 eq.) and *tert*-amyl alcohol (0.3 mL, 1 M) at 23 ˚C. The crude product was purified by silica gel column chromatography (elution with 10–13% ethyl acetate in pentane + 1% triethylamine) to give the title product as a colourless solid (75.1 mg, 30%).

Prepared according to **General Procedure A** on 0.1 mmol scale with $[Cp^*IrCl_2]_2$ (0.8 mg, 1 mol%), Ph* methyl ketone (19.0 mg, 0.1 mmol, 1 eq.), potassium *tert*-butoxide (5.6 mg, 0.05 mmol, 1 eq.), trityl-Losartan (66.5 mg, 0.1 mmol, 1 eq.) and *tert*-amyl alcohol (0.1 mL, 1 M) at **85 ˚C**. The crude product was purified by silica gel column chromatography (elution with 10–30% ethyl acetate in pentane) to give the title product as a colourless oil (74.9 mg, 89%).

The title product was observed in 55% yield by quantitative ^{1}H NMR spectroscopy following **General Procedure A** on 0.1 mmol scale with trityl-Losartan (66.5 mg, 0.1 mmol, 1 eq.) and *tert*-amyl alcohol (0.1 mL, 1 M) at **115 ˚C**.

M.p.: 150–152 °C (from ethyl acetate); **HRMS** (ESI$^+$) 859.3861 [M+Na]$^+$, expected 859.3862; **^{1}H NMR** (400 MHz, $CDCl_3$) δ: 7.99–7.91 (1H, m, H-20), 7.54–7.42 (2H, m, H-18, H-19), 7.38–7.30 (4H, m, H-17, H-27), 7.27 (6H, app td, *J* = 6.9, 3.1 Hz, H-26), 7.11 (2H, d, *J* = 8.0 Hz, H-14), 6.96–6.89 (6H, m, H-25), 6.74 (2H, d, *J* = 8.0 Hz, H-13), 5.19 (2H, s, H-11), 2.80 (2H, t, *J* = 6.7 Hz, H-2), 2.70 (2H, t, *J* = 6.8 Hz, H-3), 2.52 (2H, t, *J* = 7.8 Hz, H-7), 2.21 (3H, s, H-30), 2.14 (6H, s, H-28 or H-29), 1.89 (6H, s, H-29 or H-28), 1.75–1.57 (2H, m, H-8), 1.35–1.21 (2H, m, H-9), 0.86 (3H, t, *J* = 7.3 Hz, H-10); **^{13}C NMR** (101 MHz, $CDCl_3$) δ: 211.0 (C1), 164.0 (C22), 146.8 (C5), 141.4 (C15), 141.4 (C21), 141.4 (C24), 141.0 (C16), 139.9 (C-Ar), 135.8 (C-Ar), 135.2 (C12), 133.2 (C-Ar), 130.8 (C17), 130.4 (C20), 130.3 (C25), 130.1 (C18), 130.0 (C14), 128.5 (C27), 127.9 (C19), 127.8 (C26), 127.3 (C-Ar), 126.4 (C6), 125.2 (C13), 124.6 (C4), 83.0 (C23), 47.0 (C11), 43.8 (C2), 30.1 (C8), 27.1 (C7), 22.7 (C9), 17.1 (C3), 16.9 (C29 or C28), 16.8 (C30), 16.0 (C28 or C29), 13.9 (C10); **IR** (solid) (cm^{-1}): 1700, 1447, 1260, 1115, 1028, 880, 803, 749, 678, 698.

Unsuccessful Alcohol Substrates at 85 °C

Commentary: A host of alcohols were found to be incompatible at 85 °C (**Supplementary Figure 3**).

Procedure: Experiments were prepared according to **General Procedure A.**

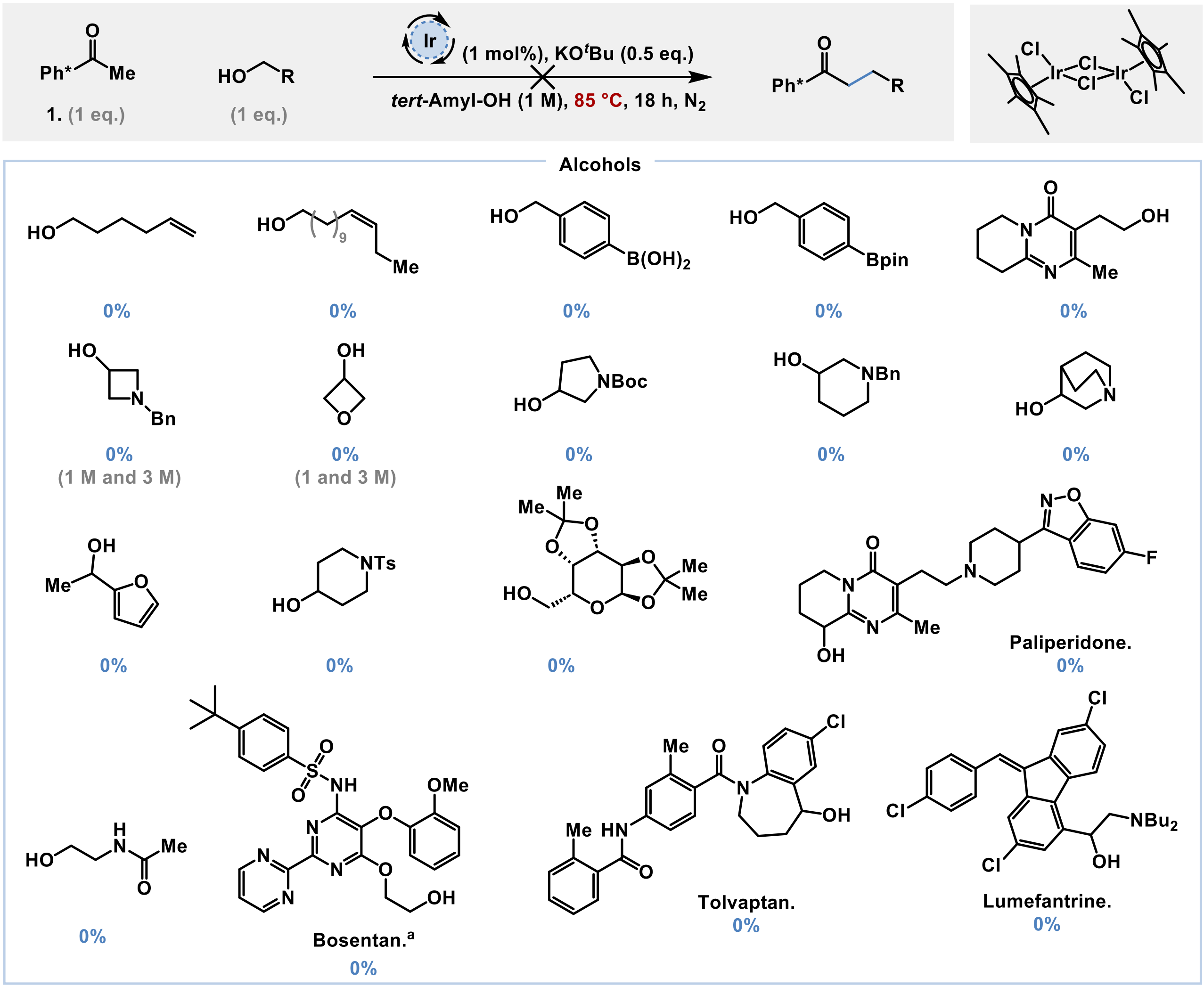

Supplementary Figure 3. Unsuccessful substrates at 85 °C. [a] Also trialled with 1.5 eq. KO^{t}Bu.

Optimisation of Reaction with Acetophenone at 23 °C

Commentary: Acetophenone was trialled in the reaction conditions which were optimised between Ph* methyl ketone and heptanol (**Supplementary Table 3, Entry 1,** 59% yield **58**). Unlike with Ph* methyl ketone the recovery of starting materials was poor. Variations of ketone equivalents, solvent concentration, base equivalents and catalyst loading were trialled before an excellent yield of product was obtained (**Entry 6,** 90% yield, 87% isolated yield **58**).

Procedure: Experiments were prepared according to **General Procedure B** with individual modifications as detailed per entry.

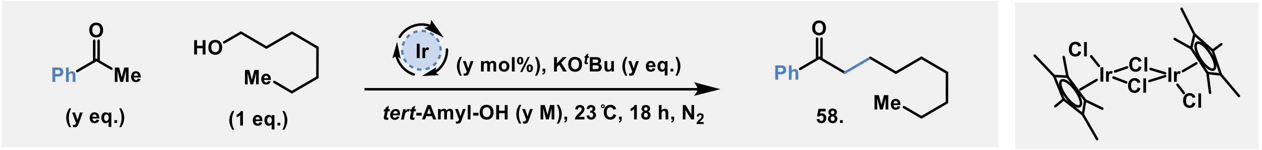

Entry	Ir mol%	PhCOMe. eq.	Solvent Conc.	Base eq.	Recovery PhCOMe.	Recovery HepOH.	Yield 58.
**1**	1%	1	1 M	0.5	0%	2%	59%
**2**	1%	**2**	1 M	0.5	29%	0%	70%
**3**	1%	2	**0.2 M**	0.5	51%	0%	34%
**4**	1%	**1**	0.2 M	0.5	0%	0%	76%
**5**	1%	1	**0.1 M**	0.5	4%	8%	75%
**6**	**2%**	1	0.1 M	0.5	1%	2%	90% **(87%)**
**7**[a]	2%	1	0.1 M	0.5	13%	27%	23%
**8**	2%	1	**0.2 M**	0.5	0%	1%	86%
**9**	**1%**	1	**0.3 M**	0.5	0%%	3%	76%
**10**	1%	1	**0.05 M**	0.5	4%	3%	65%
**11**	1%	1	**0.2 M**	**0.25**	2%	3%	83%
**12**	1%	1	**0.1 M**	0.25	20%	29%	45%
**13**	1%	1	0.1 M	**0.1**	49%	63%	6%
**14**	**1.5%**	1	0.1 M	**0.25**	13%	15%	74%
**15**	1.5%	1	0.1 M	**0.1**	42%	60%	0%
**16**	**1%**	1	**0.2 M**	0.1	22%	29%	16%
**17**	**1.5%**	1	0.2 M	**0.25**	2%	6%	86%
**18**	1.5%	1	0.2 M	**0.1**	29%	43%	4%

Supplementary Table 3. Optimisation of reaction with acetophenone. Yield was determined by quantitative ^{1}H NMR using 1,1,2,2-tetrachlorethane as internal standard. Yield in parentheses is isolated yield.[a] Reaction set up under air.

Scope of Reaction with Diverse Ketones at 23 °C, 85 °C and 115 °C

1-Phenylnonan-1-one (58)

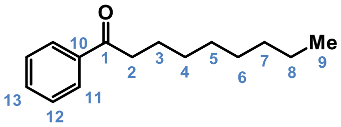

Prepared according to a modification to **General Procedure A** with acetophenone (35.0 µL, 0.3 mmol, 1 eq.), 1-heptanol (42.6 µL, 0.3 mmol, 1 eq.) and *tert*-amyl alcohol (0.3 mL, 1 M) at 23 ˚C. The crude product was purified by silica gel column chromatography (elution with 0–2% diethyl ether in pentane) to give the title product as a colourless oil (38.5 mg, **59%**).

Prepared according to **General Procedure B** with acetophenone (35.0 µL, 0.3 mmol, 1 eq.), 1-heptanol (42.6 µL, 0.3 mmol, 1 eq.) and *tert*-amyl alcohol (3.0 mL, 0.1 M) at 23 ˚C. The crude product was purified by silica gel column chromatography (elution with 0–2% diethyl ether in pentane) to give the title product as a yellow oil (57.3 mg, **87%**).

LRMS (ESI$^+$) 459.2 [M+Na]$^+$; **^{1}H NMR** (400 MHz, $CDCl_3$) δ: 8.01–7.93 (2H, m, H-11), 7.60–7.50 (1H, m, H-13), 7.51–7.41 (2H, m, H-12), 3.03–2.85 (2H, m, H-2), 1.81–1.67 (2H, m, H-3), 1.45–1.19 (10H, m, H-4, H-5, H-6, H-7, H-8), 0.92–0.84 (3H, m, H-9); **^{13}C NMR** (101 MHz, $CDCl_3$) δ: 200.8 (C1), 137.2 (C10), 133.0 (C13), 128.7 (C12), 128.2 (C11), 38.8 (C2), 32.0 (C7), 29.6 (C6), 29.5 (C5), 29.3 (C4), 24.5 (C3), 22.8 (C8), 14.3 (C9).

The spectral data matched that previously reported in the literature.[13]

3-(3-Methyloxetan-3-yl)-1-phenylpropan-1-one (59)

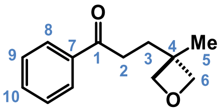

Prepared according to **General Procedure B** with acetophenone (35.0 µL, 0.3 mmol, 1 eq.) and 3-methyl-3-oxetanemethanol (29.9 µL, 0.3 mmol, 1 eq.) at 23 ˚C. The crude product was purified by silica gel column chromatography (elution with 0–20% ethyl acetate in pentane) to give the title product as a colourless oil (47.8mg, 78%).

N.b. The title product was found to be unstable in solution with chloroform.

HRMS (ESI$^+$) 227.1045 [M+Na]$^+$, expected 227.1043; **^{1}H NMR** (400 MHz, $CDCl_3$): δ 8.01–7.91 (2H, m, H-8), 7.61–7.53 (1H, m, H-10), 7.47 (2H, ddt, J = 8.2, 6.6, 1.1 Hz, H-9), 4.47 (2H, d, J = 5.7 Hz, H-6_a), 4.39 (2H,

d, J = 5.7 Hz, H-6$_b$), 3.06–2.92 (2H, m, H-2), 2.16–2.00 (2H, m, H-)3, 1.35 (3H, s, H-5); **^{13}C NMR** (101 MHz, $CDCl_3$): δ 199.9 (C1), 136.9 (C7), 133.3 (C10), 128.8 (C9), 128.2 (C8), 82.5 (C6), 39.0 (C2), 33.9 (C3), 33.0 (C4), 23.6 (C5); **IR** (thin film) (cm^{-1}): 1686, 1159, 1071, 1006, 911, 760, 734, 700, 668, 620.

1-Phenyl-3-(3-(pyrrolidin-1-ylmethyl)bicyclo[1.1.1]pentan-1-yl)propan-1-one (60)

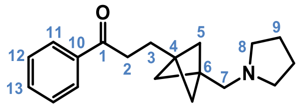

Prepared according to **General Procedure B** with acetophenone (35.0 µL, 0.3 mmol, 1 eq.) and (3-(pyrrolidin-1-ylmethyl)bicyclo[1.1.1]pentan-1-yl)methanol (54.4 mg, 0.3 mmol, 1 eq.) at 23 °C. The crude product was purified by silica gel column chromatography (elution with 0–2% methanol in CH_2Cl_2 + 1% triethylamine) to give the title product as a pale-yellow oil (52.9 mg, 62%).

HRMS (ESI$^+$) 284.2013 [M+H]$^+$, expected 284.2009; **^{1}H NMR** (400 MHz, $CDCl_3$): δ 7.99–7.89 (2H, m, H-11), 7.60–7.51 (1H, m, H-13), 7.45 (2H, ddd, J = 8.3, 6.6, 1.4 Hz, H-12), 2.96–2.87 (2H, m, H-2), 2.53 (2H, s, H-7), 2.54–2.48 (4H, m, H-8$_a$, H-8$_b$), 1.94–1.85 (2H, m, H-3), 1.75 (4H, p, J = 3.2 Hz, H-9$_a$, H-9$_b$), 1.61 (6H, s, H-5); **^{13}C NMR** (101 MHz, $CDCl_3$): δ 200.3 (C1), 137.2 (C10), 133.0 (C13), 128.7 (C12), 128.2 (C11), 58.5 (C7), 55.1 (C8), 50.9 (C5), 39.9 (C4), 38.9 (C6), 35.8 (C2), 26.5 (C3), 23.6 (C9); **IR** (thin film) (cm^{-1}): 3386, 2907, 2800, 1449, 1268, 1143, 1090, 1018, 875, 753, 666.

3-(1-((1-Methyl-1*H*-indol-3-yl)methyl)piperidin-4-yl)-1-phenylpropan-1-one (61)

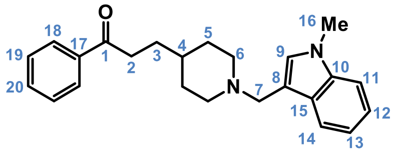

Prepared according to **General Procedure B** with acetophenone (35.0 µL, 0.3 mmol, 1 eq.) and (1-((1-methyl-1*H*-indol-3-yl)methyl)piperidin-4-yl)methanol (77.5 mg, 0.3 mmol, 1 eq.) at 23 °C. The crude product was purified by silica gel column chromatography (elution with 0–10% methanol in CH_2Cl_2 + 1% triethylamine) to give the title product as a yellow oil (88.4 mg, 82%).

HRMS (ESI$^+$) 361.2292 [M+H]$^+$, expected 361.2274; **^{1}H NMR** (500 MHz, $CDCl_3$): δ 7.96–7.90 (2H, m, H-18), 7.67 (1H, d, J = 8.0 Hz, H-14), 7.58–7.51 (1H, m, H-20), 7.45 (2H, dd, J = 8.4, 7.1 Hz, H-19), 7.31 (1H, dt, J = 8.2, 1.0 Hz, H-11), 7.28–7.20 (1H, m, H-12), 7.16–7.10 (2H, m, H-9, H-13), 3.81 (2H, s, H-7), 3.77 (3H, s, H-16), 3.07 (2H, dt, J = 12.1, 3.3 Hz, H-6$_a$), 3.00–2.93 (2H, m, H-2), 2.18–2.08 (2H, m, H-6$_b$), 1.77–1.62 (4H, m, H-5$_a$), 1.41 (3H, dddd, J = 20.4, 17.2, 11.4, 5.6 Hz, H-4, H-5$_b$); **^{13}C NMR** (126 MHz, $CDCl_3$) δ 200.4 (C1), 137.0 (C17), 136.9 (C10), 133.0 (C20), 129.4 (C9), 128.7 (C15), 128.7 (C19), 128.1 (C18), 121.7 (C12), 119.3 (C14), 119.3 (C13), 109.5 (C8), 109.3 (C11), 53.3 (C6), 53.2 (C7), 35.8 (C2), 35.1 (C4),

32.8 (C16), 31.8 (C5), 30.7 (C3); **IR** (thin film) (cm^{-1}): 1684, 1474, 1449, 1374, 1328, 1217, 970, 911, 691, 667.

3-(1-Methyl-1*H*-indol-5-yl)-1-phenylpropan-1-one (62)

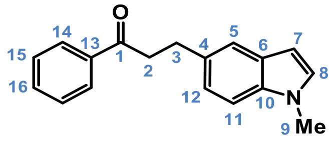

Prepared according to **General Procedure B** with acetophenone (35.0 µL, 0.3 mmol, 1 eq.) and (1-methyl-1*H*-indol-5-yl)methanol (48.4 mg, 0.3 mmol, 1 eq.) at 23 ˚C. The crude product was purified by silica gel column chromatography (elution with 5% ethyl acetate in pentane + 1% triethylamine) to give the title product as a colourless oil (73.3 mg, 93%).

HRMS (ESI^+) 264.1397 $[M+H]^+$, expected 264.1383; **^{1}H NMR** (500 MHz, $CDCl_3$): δ 8.02–7.96 (2H, m, H-14), 7.59–7.53 (1H, m, H-16), 7.51 (1H, d, *J* = 1.7 Hz, H-5), 7.46 (2H, dd, *J* = 8.4, 7.1 Hz, H-15), 7.28 (1H, d, *J* = 8.4 Hz, H-11), 7.15 (1H, dd, *J* = 8.4, 1.7 Hz, H-12), 7.04 (1H, d, *J* = 3.1 Hz, H-8), 6.44 (1H, dd, *J* = 3.2, 0.9 Hz, H-7), 3.78 (3H, s, H-9), 3.36 (2H, dd, *J* = 8.7, 6.8 Hz, H-2), 3.18 (2H, dd, *J* = 8.7, 6.9 Hz, H-3); **^{13}C NMR** (126 MHz, $CDCl_3$): δ 199.9 (C1), 137.1 (C13), 135.6 (C10), 133.1 (C16), 132.2 (C4), 129.2 (C8), 128.9 (C6), 128.7 (C15), 128.2 (C14), 122.5 (C12), 120.2 (C5), 109.3 (C11), 100.7 (C7), 41.7 (C2), 33.0 (C9), 30.5 (C3); **IR** (thin film) (cm^{-1}): 1684, 1513, 1493, 1448, 1362, 1246, 1204, 978, 758, 690.

3-(2-Methyl-6-(trifluoromethyl)pyridin-3-yl)-1-phenylpropan-1-one (63)

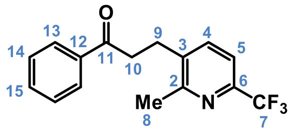

Prepared according to **General Procedure B** with acetophenone (35.0 µL, 0.3 mmol, 1 eq.) and (2-methyl-6-(trifluoromethyl)pyridin-3-yl)methanol (57.4 mg, 0.3 mmol, 1 eq.) at 23 ˚C. The crude product was purified by silica gel column chromatography (elution with 4–10% ethyl acetate in pentane) to give the title product as a yellow oil (75.5 mg, 86%).

HRMS (ESI^+) 294.1098 $[M+H]^+$, expected 294.1100; **^{1}H NMR** (500 MHz, $CDCl_3$): δ 7.98–7.92 (2H, m, H-13), 7.65 (1H, d, *J* = 7.9 Hz, H-4), 7.62–7.54 (1H, m, H-15), 7.51–7.42 (3H, m, H-14, H-5), 3.31 (2H, dd, *J* = 7.9, 6.9 Hz, H-), 3.14 (2H, t, *J* = 7.4 Hz, H-), 2.66 (3H, s, H-); **^{13}C NMR** (126 MHz, $CDCl_3$): δ 198.2 (C11), 158.0 (C2), 145.6 (q, *J* = 34.4 Hz, C6), 138.2 (C3), 137.5 (C4), 136.6 (C12), 133.6 (C15), 128.9 (C14), 128.1 (C13), 121.8 (q, *J* = 273.8 Hz, C7), 118.2 (q, *J* = 2.9 Hz, C5), 37.9 (C10), 26.7 (C9), 22.4 (C8); **^{19}F NMR** (470

MHz, $CDCl_3$): δ −67.8 (3F, s); **IR** (thin film) (cm^{-1}): 1692, 1349, 1261, 1207, 1186, 1169, 1127, 837, 760, 693.

3-(4-Methyl-2-phenyloxazol-5-yl)-1-phenylpropan-1-one (64)

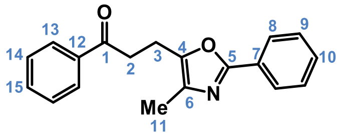

Prepared according to **General Procedure B** with acetophenone (35.0 µL, 0.3 mmol, 1 eq.) and (4-methyl-2-phenyloxazol-5-yl)methanol (56.8 mg, 0.3 mmol, 1 eq.) at 85 ˚C. The crude product was purified by silica gel column chromatography (elution with 5–10% ethyl acetate in pentane) to give the title product as a colourless oil (71.9 mg, 83%).

HRMS (ESI^+) 292.1334 $[M+H]^+$, expected 292.1332; **^{1}H NMR** (400 MHz, $CDCl_3$): δ 8.10–7.87 (4H, m, H-8, H-13), 7.62 7.48 (1H, m, H-15), 7.48–7.32 (5H, m, H-9,H-10, H-14), 3.41 (2H, t, *J* = 7.2 Hz, H-2), 2.94 (2H, t, *J* = 7.2 Hz, H-3), 2.38 (3H, s, H-11); **^{13}C NMR** (101 MHz, $CDCl_3$): δ 199.5 (C1), 159.5 (C5), 144.0 (C4), 137.0 (C12), 135.1 (C6), 133.1 (C15), 129.8 (C10), 128.8 (C9), 128.7 (C14), 128.2 (C13), 127.9 (C7), 126.0 (C8), 37.7 (C2), 20.2 (C3), 10.3 (C11); **IR** (thin film) (cm^{-1}): 1686, 1449, 1366, 1180, 914, 776, 742, 715, 691, 626.

3-(1-Benzylpiperidin-4-yl)-1-(4-methoxyphenyl)propan-1-one (65)

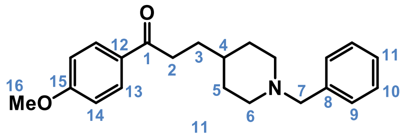

Prepared according to **General Procedure B** with 1-(4-methoxyphenyl)ethan-1-one (45.1 mg, 0.3 mmol, 1 eq.) and (1-benzylpiperidin-4-yl)methanol (61.6 mg, 0.3 mmol, 1 eq.) at 23 ˚C. The crude product was purified by silica gel column chromatography with Fluorochem silica gel 60A (0.020–0.045 nm), (elution with 10% ethyl acetate in pentane, then 0–5% methanol in CH_2Cl_2 + 1% triethylamine) to give the title product as a yellow solid (75.9 mg, 75%).

M.p.: 56–58 °C (from CH_2Cl_2); **HRMS** (ESI^+) 338.2105 $[M+H]^+$, expected 338.2115; **^{1}H NMR** (400 MHz, $CDCl_3$) δ 7.97–7.85 (2H, m, H-13), 7.32 (4H, m, H-9, H-10), 7.28–7.22 (1H, m, H-11), 6.96–6.88 (2H, m, H-14), 3.86 (3H, s, H-16), 3.52 (2H, s, H-7), 2.92 (4H, m, H-2, H-6_a), 2.03–1.89 (2H, m, H-6_b), 1.68 (4H, m, H-3, H-5_a), 1.35 (3H, d, *J* = 8.5 Hz, H-4, H-5_b); **^{13}C NMR** (101 MHz, $CDCl_3$) δ 199.2 (C1), 163.5 (C15), 138.0 (C8), 130.4 (C13), 130.2 (C12), 129.5 (C9), 128.3 (C10), 127.2 (C11), 113.8 (C14), 63.4 (C7), 55.6 (C16),

53.8 (C6), 35.6 (C2), 35.4 (C4), 32.1 (C5), 31.1 (C3); **IR** (thin film) (cm^{-1}): 1677, 1602, 1511, 1455, 1313, 1261, 1173, 1031, 739, 701.

3-(1-Benzylpiperidin-4-yl)-1-(3-methoxyphenyl)propan-1-one (66)

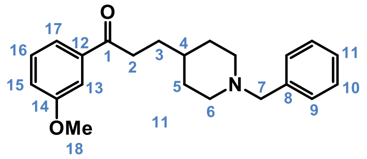

Prepared according to **General Procedure B** with 1-(3-methoxyphenyl)ethan-1-one (41.2 µL, 0.3 mmol, 1 eq.) and (1-benzylpiperidin-4-yl)methanol (61.6 mg, 0.3 mmol, 1 eq.) at 23 ˚C. The crude product was purified by silica gel column chromatography with Fluorochem silica gel 60A (0.020–0.045 nm), (elution with 10% ethyl acetate in pentane, then 0–2% methanol in CH_2Cl_2 + 1% triethylamine) to give the title product as a yellow oil (86.2 mg, 85%).

HRMS (ESI+) 338.2123 [M+H]+, expected 338.2115; **^{1}H NMR** (400 MHz, $CDCl_3$) δ 7.52 (1H, dt, *J* = 7.6, 1.2 Hz, H-17), 7.48 (1H, dd, *J* = 2.7, 1.5 Hz, H-13), 7.36 (1H, app t, *J* = 7.9 Hz, H-16), 7.33–7.27 (4H, m, H-9, H-10), 7.27–7.21 (1H, m, H-11), 7.10 (1H, ddd, *J* = 8.2, 2.7, 1.0 Hz, H-15), 3.85 (3H, s, H-18), 3.49 (2H, s, H-7), 2.96 (2H, dd, *J* = 8.2, 7.0 Hz, H-2), 2.89 (2H, ddd, *J* = 12.8, 4.3, 2.2 Hz, H-6_a), 1.95 (2H, t, *J* = 10.8 Hz, H-6_b), 1.75–1.64 (4H, m, H-3, H-5_a), 1.32 (3H, hept, *J* = 3.0 Hz, H-4, H-5_b); **^{13}C NMR** (101 MHz, $CDCl_3$) δ 200.5 (C1), 156.0 (C14), 138.6 (C8), 138.5 (C12), 129.7 (C16), 129.4 (C9), 128.3 (C10), 127.1 (C11), 120.8 (C17), 119.5 (C15), 112.5 (C13), 63.6 (C7), 55.6 (C18), 53.9 (C6), 36.2 (C2), 35.5 (C4), 32.3 (C5), 31.1 (C3); **IR** (thin film) (cm^{-1}): 1686, 1597, 1584, 1454, 1432, 1258, 1197, 1043, 744, 700.

3-(1-Benzylpiperidin-4-yl)-1-(2-methoxyphenyl)propan-1-one (67)

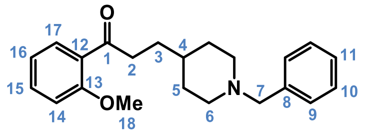

Prepared according to **General Procedure B** with 1-(2-methoxyphenyl)ethan-1-one (41.3 µL, 0.3 mmol, 1 eq.) and (1-benzylpiperidin-4-yl)methanol (61.6 mg, 0.3 mmol, 1 eq.) at 85 ˚C. The crude product was purified by silica gel column chromatography with Fluorochem silica gel 60A (0.020–0.045 nm), (elution with 10% ethyl acetate in pentane, then 0–2% methanol in CH_2Cl_2 + 1% triethylamine) to give the title product as a yellow oil (80.1 mg, 79%).

Following the above procedure **at 23 ˚C**, the title product was isolated as a yellow oil (34.3 mg, 34%).

HRMS (ESI$^+$) 338.2118 [M+H]$^+$, expected 338.2115; ^{1}**H NMR** (400 MHz, $CDCl_3$) δ 7.64 (1H, dd, *J* = 7.6, 1.9 Hz, H-17), 7.44 (1H, ddd, *J* = 8.6, 7.4, 1.8 Hz, H-15), 7.31 (4H, d, *J* = 4.3 Hz, H-9, H-10), 7.27–7.22 (1H, m, H-11), 6.99 (1H, td, *J* = 7.5, 1.0 Hz, H-16), 6.95 (1H, d, *J* = 8.5 Hz, H-14), 3.88 (3H, s, H-18), 3.50 (2H, s, H-7), 3.01–2.91 (2H, m, H-2), 2.88 (2H, d, *J* = 10.6 Hz, H-6_a), 2.00–1.87 (2H, m, H-6_b), 1.73–1.56 (4H, m, H-3, H-5_a), 1.27 (3H, d, *J* = 12.8 Hz, H-4, H-5_b); ^{13}**C NMR** (126 MHz, $CDCl_3$) δ 203.4 (C1), 158.4 (C13), 138.5 (C8), 133.3 (C15), 130.3 (C17), 129.4 (C9), 128.8 (C12), 128.3 (C10), 127.1 (C11), 120.8 (C16), 111.6 (C14), 63.6 (C7), 55.6 (C18), 54.0 (C6), 41.3 (C2), 35.6 (C4), 32.3 (C5), 31.1 (C3); **IR** (thin film) (cm^{-1}): 1674, 1598, 1486, 1395, 1287, 1246, 1025, 761, 701, 669.

***tert*-Butyl 3-(3-(4-bromophenyl)-3-oxopropyl)azetidine-1-carboxylate (68)**

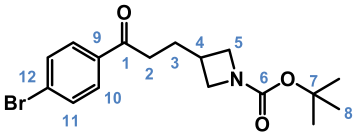

Prepared according to **General Procedure B** with *tert*-butyl 3-(hydroxymethyl)azetidine-1-carboxylate (56.2 mg, 0.3 mmol, 1 eq.) and 1-(4-bromophenyl)ethan-1-one (58.3 mg, 0.3 mmol, 1 eq.) at 85 ˚C. The crude product was purified by silica gel column chromatography (elution with 5% ethyl acetate in toluene + 1% triethylamine) to give the title product as a colourless solid (84.0 mg, 76%).

M.p.: 56–58 °C (from ethyl acetate); **HRMS** (ESI$^+$) 368.0852 [M+H]$^+$, expected 368.0856; ^{1}**H NMR** (400 MHz, $CDCl_3$) δ 7.86–7.73 (2H, m, H-10), 7.68–7.54 (2H, m, H-11), 4.01 (2H, t, *J* = 8.3 Hz, H-5_a), 3.57 (2H, dd, *J* = 8.5, 5.5 Hz, H-5_b), 2.88 (2H, t, *J* = 7.3 Hz, H-2), 2.57 (1H, tt, *J* = 8.0, 5.5 Hz, H-4), 2.01 (2H, q, *J* = 7.4 Hz, H-3), 1.42 (9H, s, H-8); ^{13}**C NMR** (101 MHz, $CDCl_3$) δ 198.3 (C1), 156.5 (C6), 135.5 (C9), 132.1 (C11), 129.7 (C10), 128.5 (C12), 79.5 (C7), 54.3 (C5), 35.8 (C2), 28.7 (C3), 28.5 (C8), 28.4 (C4); **IR** (thin film) (cm^{-1}): 1699, 1586, 1482, 1409, 1367, 1162, 1071, 1008, 774, 636.

***tert*-butyl 4-(3-(4-(dimethylamino)phenyl)-3-oxopropyl)piperidine-1-carboxylate (69)**

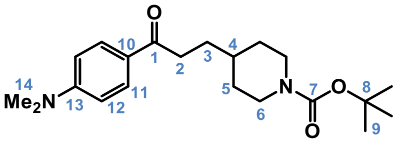

Prepared according to **General Procedure B** with 1-(4-(dimethylamino)phenyl)ethan-1-one (49.0 mg, 0.3 mmol, 1 eq.) and *tert*-butyl 4-(hydroxymethyl)piperidine-1-carboxylate (64.6 mg, 0.3 mmol, 1 eq.) at 85 ˚C. The crude product was purified by silica gel column chromatography (elution with 10–20% ethyl acetate in pentane + 1% triethylamine) to give the title product as a yellow oil (86.1 mg, 80%).

HRMS (ESI+) 361.2495 [M+H]+, expected 361.2486; **^{1}H NMR** (400 MHz, $CDCl_3$) δ 7.90–7.80 (2H, m, H-11), 6.68–6.58 (2H, m, H-12), 4.07 (2H, s, H-6_a), 3.04 (6H, s, H-14), 2.88 (2H, t, *J* = 7.6 Hz, H-2), 2.72–2.61 (2H, m, H-6_b), 1.76–1.62 (4H, m, H-3, H-5_a), 1.55–1.40 (1H, m, H-4), 1.44 (9H, s, H-9), 1.12 (2H, qd, *J* = 12.5, 4.4 Hz, H-5_b); **^{13}C NMR** (101 MHz, $CDCl_3$) δ 198.6 (C1), 155.0 (C7), 153.4 (C13), 130.3 (C11), 125.0 (C10), 110.8 (C12), 79.3 (C8), 44.0 (C6), 40.1 (C14), 35.8 (C4), 35.0 (C2), 32.2 (C5), 31.5 (C3), 28.6 (C9); **IR** (thin film) (cm^{-1}): 2978, 2859, 1692, 1600, 1427, 1367, 1281, 1245, 1169, 762.

***tert*-Butyl 4-(3-(4-amino-3,5-dichlorophenyl)-3-oxopropyl)piperidine-1-carboxylate (70)**

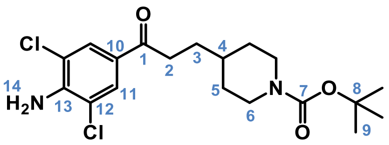

Prepared according to **General Procedure B** with 1-(4-amino-3,5-dichlorophenyl)ethan-1-one (61.2 mg, 0.3 mmol, 1 eq.) and *tert*-butyl 4-(hydroxymethyl)piperidine-1-carboxylate (64.6 mg, 0.3 mmol, 1 eq.) at 85 ˚C. The crude product was purified by silica gel column chromatography (elution with 15–20% ethyl acetate in pentane + 1% triethylamine) to give the title product as a yellow oil (110.1 mg, 91%).

HRMS (ESI+) 423.1225 [M+Na]+, expected 423.1213; **^{1}H NMR** (400 MHz, $CDCl_3$) δ 7.80 (2H, s, H-11), 4.93 (2H, s, H-14), 4.15–3.97 (2H, m, H-6_a), 2.85 (2H, t, *J* = 7.5 Hz, H-2), 2.65 (2H, d, *J* = 12.9 Hz, H-6_b), 1.73–1.60 (4H, m, H-3, H-5_a), 1.52–1.39 (1H, m, H-4), 1.44 (9H, s, H-9), 1.12 (2H, qd, *J* = 12.5, 4.4 Hz, H-5_b); **^{13}C NMR** (101 MHz, $CDCl_3$) δ 196.6 (C1), 155.0 (C7), 144.2 (C13), 128.4 (C11), 127.3 (C10), 118.9 (C12), 79.4 (C8), 43.9 (C6), 35.7 (C4), 35.1 (C2), 32.1 (C5), 30.8 (C3), 28.6 (C9); **IR** (thin film) (cm^{-1}): 3484, 3352, 1681, 1616, 1428, 1409, 1277, 1213, 1164, 761.

1-(4-Aminophenyl)-3-(3-(3-methyl-1,2,4-oxadiazol-5-yl)phenyl)propan-1-one (71)

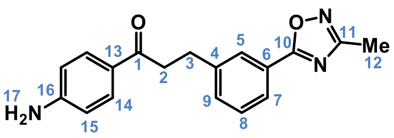

Prepared according to **General Procedure B** with (3-(3-methyl-1,2,4-oxadiazol-5-yl)phenyl)methanol (57.1 mg, 0.3 mmol, 1 eq.) and 1-(4-aminophenyl)ethan-1-one (40.6 mg, 0.3 mmol, 1 eq.) at 85 ˚C. The crude product was purified by silica gel column chromatography (elution with 30–50% ethyl acetate in pentane + 1% triethylamine) to give the title product as a colourless solid (58.0 mg, 63%).

M.p.: 145–147°C (from ethyl acetate); **HRMS** (ESI+) 308.1392 [M+H]+, expected 308.1394; **^{1}H NMR** (400 MHz, $CDCl_3$) δ 8.00 (1H, d, *J* = 1.9 Hz, H-5), 7.93 (1H, dt, *J* = 7.5, 1.6 Hz, H-7), 7.85–7.77 (2H, m, H-14),

7.50–7.46 (1H, m, H-9), 7.43 (1H, t, *J* = 7.6 Hz, H-8), 6.67–6.61 (2H, m, H-15), 4.15 (2H, s, H-17), 3.24 (2H, ddd, *J* = 8.1, 6.8, 1.0 Hz, H-2), 3.12 (2H, dd, *J* = 8.4, 6.7 Hz, H-3), 2.47 (3H, s, H-12); **^{13}C NMR** (101 MHz, $CDCl_3$) δ 196.9 (C1), 175.7 (C10), 167.9 (C11), 151.3 (C16), 143.1 (C4), 133.2 (C9), 130.6 (C14), 129.4 (C8), 128.0 (C5), 127.4 (C13), 125.9 (C7), 124.4 (C6), 113.9 (C15), 39.4 (C2), 30.3 (C3), 11.9 (C12); **IR** (thin film) (cm^{-1}): 3361, 1660, 1629, 1599, 1567, 1393, 1344, 1177, 770, 749.

1-(4-amino-3-bromophenyl)-5-(pyridin-3-yl)pentan-1-one (72)

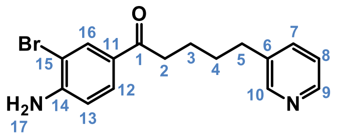

Prepared according to **General Procedure B** with 1-(4-amino-3-bromophenyl)ethan-1-one (64.2 mg, 0.3 mmol, 1 eq.) and 3-(pyridin-3-yl)propan-1-ol (38.7 μL, 0.3 mmol, 1 eq.) at 85 ˚C. The crude product was purified by silica gel column chromatography (elution with 50–70% ethyl acetate in pentane + 1% triethylamine) to give the title product as a colourless solid (70.8 mg, 71%).

M.p.: 114–116 °C (from ethyl acetate); **HRMS** (ESI$^+$) 333.0605, 333.0586 [1:1, M+H]$^+$, expected 333.0597, 335.0577 [1:1]; **^{1}H NMR** (400 MHz, $CDCl_3$) δ 8.50–8.39 (2H, m, H-9, H-10), 8.04 (1H, d, *J* = 2.0 Hz, H-16), 7.72 (1H, dd, *J* = 8.4, 2.0 Hz, H-12), 7.49 (1H, dt, *J* = 7.8, 2.0 Hz, H-7), 7.20 (1H, dd, *J* = 7.8, 4.8 Hz, H-8), 6.73 (1H, d, *J* = 8.4 Hz, H-13), 4.61 (2H, s, H-17), 2.87 (2H, t, *J* = 7.0 Hz, H-2), 2.65 (2H, t, *J* = 7.4 Hz, H-5), 1.73 (4H, dddd, *J* = 17.5, 15.5, 7.9, 4.4 Hz, H-3, H-4); **^{13}C NMR** (101 MHz, $CDCl_3$) δ 197.3 (C1), 150.0 (C10), 148.4 (C14), 147.7 (C9), 137.6 (C6), 135.9 (C7), 133.6 (C16), 129.2 (C12), 128.6 (C11), 123.4 (C8), 114.4 (C13), 108.4 (C15), 37.7 (C2), 33.1 (C5), 30.9 (C4), 24.2 (C3); **IR** (thin film) (cm^{-1}):3458, 3348, 1666, 1625, 1590, 1422, 1221, 1002, 763, 715.

1-(4-amino-3-bromophenyl)-3-(1-methyl-3-(thiophen-2-yl)-1*H*-pyrazol-5-yl)propan-1-one (73)

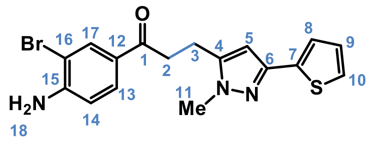

Prepared according to **General Procedure B** with 1-(4-amino-3-bromophenyl)ethan-1-one (64.2 mg, 0.3 mmol, 1 eq.) and (4-methyl-2-(piperidin-1-yl)thiazol-5-yl)methanol (58.3 mg, 0.3 mmol, 1 eq.) at 85 ˚C. The crude product was purified by silica gel column chromatography (elution with 30–50% ethyl acetate in pentane + 1% triethylamine) to give the title product as a colourless solid (90.3 mg, 77%).

M.p.: 116–118 °C (from ethyl acetate); **HRMS** (ESI$^+$) 390.0267 [M+H]$^+$, expected 390.0270; **^{1}H NMR** (400 MHz, $CDCl_3$) δ 8.09 (1H, d, *J* = 2.0 Hz, H-17), 7.76 (1H, dd, *J* = 8.4, 2.0 Hz, H-13), 7.26–7.24 (1H, m, H-8),

7.20 (1H, dd, J = 5.1, 1.1 Hz, H-10), 7.02 (1H, dd, J = 5.0, 3.6 Hz, H-9), 6.74 (1H, d, J = 8.4 Hz, H-14), 6.25 (1H, s, H-5), 4.64 (2H, s, H-18), 3.85 (3H, s, H-11), 3.25 (2H, t, J = 7.5 Hz, H-2), 3.02 (2H, t, J = 7.5 Hz, H-3); **^{13}C NMR** (101 MHz, $CDCl_3$) δ 195.2 (C1), 148.8 (C15), 145.6 (C6), 143.6 (C4), 137.1 (C7), 133.6 (C17), 129.2 (C13), 128.1 (C12), 127.5 (C9), 124.2 (C10), 123.3 (C8), 114.4 (C14), 108.4 (C16), 101.5 (C5), 36.6 (C2), 36.4 (C11), 20.1 (C3); **IR** (thin film) (cm^{-1}): 3465, 3347, 1670, 1618, 1589, 1412, 1215, 762, 670, 635.

1-(3-Aminophenyl)-5-morpholinopentan-1-one (74)

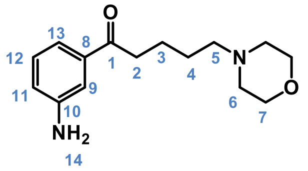

Prepared according to **General Procedure B** with 1-(3-aminophenyl)ethan-1-one (40.6 mg, 0.3 mmol, 1 eq.) and 3-morpholinopropan-1-ol (41.5 µL, 0.3 mmol, 1 eq.) at 85 ˚C. The crude product was purified by silica gel column chromatography with Fluorochem silica gel 60A (0.020–0.045 nm), (elution with 0–2% methanol in CH_2Cl_2 + 1% triethylamine) to give the title product as a yellow oil (54.4 mg, 69%).

HRMS (ESI$^+$) 285.1586 [M+H]$^+$, expected 285.1574; **^{1}H NMR** (400 MHz, $CDCl_3$) δ 7.32 (1H, d, J = 7.6 Hz, H-13), 7.27–7.19 (2H, m, H-9, H-12), 6.85 (1H, dd, J = 7.9, 2.5 Hz, H-11), 3.80 (2H, s, H-14), 3.70 (4H, app t, J = 4.7 Hz, H-7_a, H-7_b), 2.94 (2H, t, J = 7.2 Hz, H-2), 2.53–2.40 (4H, m, H-6_a, H-6_b), 2.37 (2H, t, J = 7.6 Hz, H-2), 1.75 (2H, p, J = 7.4 Hz, H-3), 1.56 (2H, p, J = 7.6 Hz, H-4); **^{13}C NMR** (101 MHz, $CDCl_3$) δ 200.5 (C1), 146.9 (C10), 138.3 (C8), 129.6 (C12), 119.6 (C11), 118.6 (C13), 114.0 (C9), 67.1 (C7), 58.9 (C5), 53.9 (C6), 38.5 (C2), 26.3 (C4), 22.4 (C3); **IR** (thin film) (cm^{-1}): 1678, 1603, 1458, 1328, 1289, 1116, 866, 764, 669, 627.

3-(1-Benzylpiperidin-4-yl)-1-(4-hydroxyphenyl)propan-1-one (75)

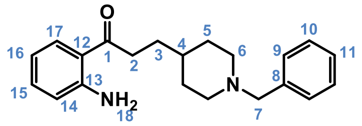

Prepared according to **General Procedure B** with 1-(2-aminophenyl)ethan-1-one (40.6 mg, 0.3 mmol, 1 eq.) and (1-benzylpiperidin-4-yl)methanol (61.6 mg, 0.3 mmol, 1 eq.) at **115 ˚C**. The crude product was purified by silica gel column chromatography with Fluorochem silica gel 60A (0.020–0.045 nm), (elution with 0–2% methanol in CH_2Cl_2 + 1% triethylamine) to give the title product as a yellow oil (63.6 mg, 66%).

HRMS (ESI+) 323.2129 [M+H]+, expected 323.2118; **^{1}H NMR** (400 MHz, $CDCl_3$) δ 7.74–7.70 (1H, m, H-17), 7.34–7.29 (4H, m, H-9, H-10), 7.28–7.22 (2H, m, H-11, H-15), 6.68–6.59 (2H, m, H-14, H-16), 6.28–6.21 (2H, m, H-18), 3.50 (2H, s, H-7), 2.99–2.85 (4H, m, H-2, H-6_a), 2.01–1.92 (2H, m, H-6_b), 1.74–1.62 (4H, m, H-3, H-5_a), 1.32 (3H, app h, *J* = 3.9 Hz, H-4, H-5_b); **^{13}C NMR** (101 MHz, $CDCl_3$) δ 203.2 (C1), 150.5 (C13), 138.3 (C8), 134.3 (C15), 131.3 (C17), 129.5 (C9), 128.3 (C10), 127.1 (C11), 118.0 (C12), 117.5 (C14), 115.9 (C16), 63.6 (C7), 53.9 (C6), 36.7 (C2), 35.6 (C4), 32.3 (C5), 31.6 (C3); **IR** (thin film) (cm^{-1}): 3343, 1651, 1617, 1587, 1551, 1223, 968, 771, 747, 698.

3-(1-Benzylpiperidin-4-yl)-1-(4-hydroxyphenyl)propan-1-one (76)

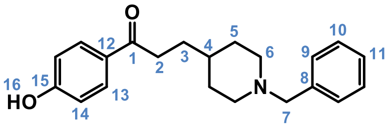

Prepared according to **General Procedure B** with 1-(4-hydroxyphenyl)ethan-1-one (40.8 mg, 0.3 mmol, 1 eq.), (1-benzylpiperidin-4-yl)methanol (61.6 mg, 0.3 mmol, 1 eq.) and powdered potassium *tert*-butoxide (50.4 mg, 0.45 mmol, **1.5 eq.**) at **115 ˚C**. The crude product was purified by silica gel column chromatography with Fluorochem silica gel 60A (0.020–0.045 nm), (elution with 0–4% methanol in CH_2Cl_2 + 1% triethylamine) to give the title product as a yellow oil (70.7 mg, 73%).

M.p.: 125–127 °C (from CH_2Cl_2); **HRMS** (ESI+) 324.1954 [M+H]+, expected 324.1958; **^{1}H NMR** (400 MHz, $CDCl_3$) δ 7.80 (2H, d, *J* = 7.9 Hz, H-13), 7.28 (5H, dd, *J* = 12.5, 3.5 Hz, H-9, H-10, H-11), 6.75 (2H, d, *J* = 8.3 Hz, H-14), 5.89 (1H, s, H-16), 3.56 (2H, s, H-7), 2.97 (2H, d, *J* = 11.2 Hz, H-6_a), 2.85 (2H, t, *J* = 7.6 Hz, H-2), 2.04 (2H, t, *J* = 11.1 Hz, H-6_b), 1.80–1.60 (4H, m, H-3, H-5_a), 1.34 (3H, dt, *J* = 13.1, 7.4 Hz, H-4, H-5_b); **^{13}C NMR** (101 MHz, $CDCl_3$) δ 199.5 (C1), 161.9 (C15), 136.7 (C8), 130.8 (C13), 130.0 (C9), 129.0 (C12), 128.5 (C10), 127.6 (C11), 115.9 (C14), 63.4 (C7), 53.7 (C6), 35.3 (C2), 35.2 (C4), 31.5 (C5), 31.1 (C3); **IR** (thin film) (cm^{-1}): 2928, 1664, 1602, 1583, 1454, 1225, 1167, 844, 761, 639.

3-(1-Benzylpiperidin-4-yl)-1-(3-hydroxyphenyl)propan-1-one (77)

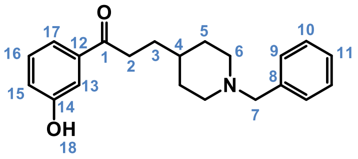

Prepared according to **General Procedure B** with 1-(3-hydroxyphenyl)ethan-1-one (40.8 mg, 0.3 mmol, 1 eq.), (1-benzylpiperidin-4-yl)methanol (61.6 mg, 0.3 mmol, 1 eq.) and powdered potassium *tert*-butoxide (50.4 mg, 0.45 mmol, **1.5 eq.**) at **115 ˚C**. The crude product was purified by silica gel

column chromatography with Fluorochem silica gel 60A (0.020–0.045 nm), (elution with 0–4% methanol in CH_2Cl_2 + 1% triethylamine) to give the title product as a yellow oil (81.4 mg, 84%).

HRMS (ESI+) 324.1955 [M+H]+, expected 324.1958; **^{1}H NMR** (400 MHz, $CDCl_3$) δ 7.42 (1H, d, *J* = 7.6 Hz, H-17), 7.35–7.23 (7H, m, H-9, H-10, H-11, H-13, H-16), 6.96 (1H, dd, *J* = 7.9, 2.4 Hz, H-15), 5.04 (1H, s, H-18), 3.57 (2H, s, H-7), 2.98 (2H, d, *J* = 11.1 Hz, H-6_a), 2.85 (2H, t, *J* = 7.5 Hz, H-2), 2.09–1.97 (2H, m, H-6_b), 1.71–1.58 (4H, m, H-3, H-5_a), 1.35 (3H, d, *J* = 7.5 Hz, H-4, H-5_b); **^{13}C NMR** (101 MHz, $CDCl_3$) δ 200.8 (C1), 157.0 (C14), 138.3 (C12), 136.6 (C8), 130.0 (C16), 129.9 (C9), 128.4 (C10), 127.6 (C11), 120.8 (C15), 119.9 (C17), 115.1 (C13), 63.4 (C7), 53.6 (C6), 35.8 (C2), 35.1 (C4), 31.5 (C5), 30.7 (C3); **IR** (thin film) (cm^{-1}): 1682, 1583, 1455, 1376, 1281, 1244, 997, 763, 703, 650.

3-(1-Benzylpiperidin-4-yl)-1-(4-hydroxyphenyl)propan-1-one (78)

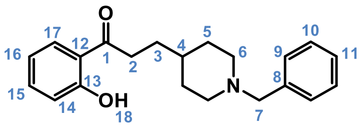

Prepared according to **General Procedure B** with 1-(2-hydroxyphenyl)ethan-1-one (36.1 μL, 0.3 mmol, 1 eq.), (1-benzylpiperidin-4-yl)methanol (61.6 mg, 0.3 mmol, 1 eq.) and powdered potassium *tert*-butoxide (50.4 mg, 0.45 mmol, **1.5 eq.**) at **115 °C**. The crude product was purified by silica gel column chromatography with Fluorochem silica gel 60A (0.020–0.045 nm), (elution with 0–4% methanol in CH_2Cl_2 + 1% triethylamine) to give the title product as a yellow oil (75.5 mg, 78%).

HRMS (ESI+) 324.1955 [M+H]+, expected 324.1958; **^{1}H NMR** (400 MHz, $CDCl_3$) δ 12.36 (1H, s, H-18), 7.74 (1H, dd, *J* = 8.1, 1.7 Hz, H-17), 7.45 (1H, ddd, *J* = 8.7, 7.2, 1.7 Hz, H-15), 7.31 (4H, d, *J* = 4.4 Hz, H-9, H-10), 7.27–7.21 (1H, m, H-11), 6.97 (1H, dd, *J* = 8.4, 1.2 Hz, H-14), 6.88 (1H, ddd, *J* = 8.3, 7.2, 1.2 Hz, H-16), 3.49 (2H, s, H-7), 3.04–2.93 (2H, m, H-2), 2.93–2.82 (2H, m, H-6_a), 1.98–1.88 (2H, m, H-6_b), 1.69 (4H, td, *J* = 7.5, 4.5 Hz, H-3, H-5_a), 1.32 (3H, dp, *J* = 11.6, 4.2 Hz, H-4, H-5_b); **^{13}C NMR** (101 MHz, $CDCl_3$) δ 207.0 (C1), 162.6 (C13), 138.6 (C8), 136.4 (C15), 130.0 (C17), 129.3 (C9), 128.3 (C10), 127.0 (C11), 119.4 (C12), 119.0 (C16), 118.7 (C14), 63.6 (C7), 53.9 (C6), 35.8 (C2), 35.5 (C4), 32.3 (C5), 31.1 (C3); **IR** (thin film) (cm^{-1}): 1642, 1489, 1449, 1311, 973, 759, 700, 664, 634, 612.

***tert*-Butyl 4-(3-oxo-3-(pyridin-3-yl)propyl)piperidine-1-carboxylate (79)**

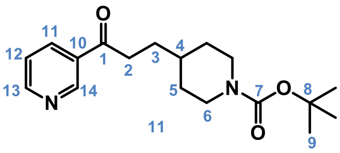

Prepared according to **General Procedure B** with 1-(pyridin-3-yl)ethan-1-one (33.0 µL, 0.3 mmol, 1 eq.) and *tert*-butyl 4-(hydroxymethyl)piperidine-1-carboxylate (64.6 mg, 0.3 mmol, 1 eq.) at 85 ˚C. The crude product was purified by silica gel column chromatography (elution with 10% methanol in toluene) and further purified by preparative thin layer chromatography (elution with diethyl ether) to give the title product as a colourless oil (30.8 mg, 32%).

N.b. The title product was not observed by quantitative ^{1}H NMR spectroscopy in 47% yield.

HRMS (ESI^{+}) 319.2018 [M+H]$^{+}$, expected 319.2016; **^{1}H NMR** (400 MHz, $CDCl_3$) δ: 9.15 (1H, d, *J* = 2.3 Hz, H-14), 8.77 (1H, dd, *J* = 4.8, 1.7 Hz, H-13), 8.21 (1H, dt, *J* = 8.0, 2.0 Hz, H-11), 7.41 (1H, ddd, *J* = 8.0, 4.8, 0.9 Hz, H-12), 4.09 (2H, app s, H-6$_a$), 3.01 (2H, t, *J* = 7.5 Hz, H-2), 2.80–2.59 (2H, m, H-6$_b$), 1.70 (4H, dt, *J* = 12.0, 5.7 Hz, H-3, H-5$_a$), 1.53–1.42 (1H, m, H-4), 1.44 (9H, s, H-9), 1.14 (2H, qd, *J* = 12.5, 4.4 Hz, H-5$_b$); **^{13}C NMR** (101 MHz, $CDCl_3$) δ: 199.0 (C1), 155.0 (C7), 153.6 (C13), 149.7 (C14), 135.5 (C11), 132.2 (C10), 123.8 (C12), 79.5 (C8), 44.0 (C6), 36.1 (C2), 35.7 (C4), 32.1 (C5), 30.4 (C3), 28.6 (C9); **IR** (thin film) (cm^{-1}): 1693, 1587, 1424, 1367, 1281, 1245, 1168, 738, 706, 648.

***tert*-Butyl 4-(3-oxo-3-(pyridin-2-yl)propyl)piperidine-1-carboxylate (80)**

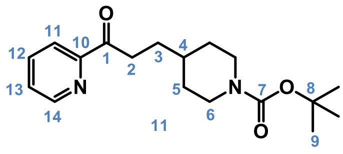

Prepared according to **General Procedure B** with 1-(pyridin-2-yl)ethan-1-one (33.7 µL, 0.3 mmol, 1 eq.) and *tert*-butyl 4-(hydroxymethyl)piperidine-1-carboxylate (64.6 mg, 0.3 mmol, 1 eq.) at 85 ˚C. The crude product was purified by silica gel column chromatography (elution with 5–10% ethyl acetate in pentane + 1% triethylamine) to give the title product as a colourless oil (34.7 mg, 36%).

HRMS (ESI^{+}) 319.2024 [M+H]$^{+}$, expected 319.2016; **^{1}H NMR** (400 MHz, $CDCl_3$) δ: 8.69–8.63 (1H, m, H-14), 8.03 (1H, dt, *J* = 7.8, 1.1 Hz, H-11), 7.83 (1H, td, *J* = 7.7, 1.8 Hz, H-12), 7.46 (1H, ddd, *J* = 7.6, 4.8, 1.3 Hz, H-13), 4.19–3.91 (2H, m, H-6$_a$), 3.25 (2H, t, *J* = 7.6 Hz, H-2), 2.67 (2H, t, *J* = 12.9 Hz, H-6$_b$), 1.80–1.61 (4H, m, H-3, H-5$_a$), 1.52–1.40 (1H, m, H-4), 1.44 (9H, s, H-9), 1.26–1.06 (2H, m, H-5$_b$); **^{13}C NMR** (101 MHz, $CDCl_3$) δ: 202.1 (C1), 155.0 (C7), 153.5 (C10), 149.1 (C14), 137.1 (C12), 127.2 (C13), 121.9 (C11), 79.3 (C8), 44.1 (C6), 35.8 (C4), 35.0 (C2), 32.2 (C5), 30.5 (C3), 28.6 (C9); **IR** (thin film) (cm^{-1}): 1698, 1426, 1367, 1282, 1248, 1168, 996, 772, 642.

4,4-Dimethyl-1-(2-methyl-6-(trifluoromethyl)pyridin-3-yl)pentan-3-one (81)

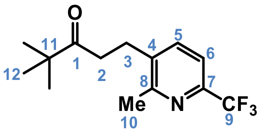

Prepared according to **General Procedure B** with 3,3-dimethylbutan-2-one (37.5 μL, 0.3 mmol, 1 eq.) and (2-methyl-6-(trifluoromethyl)pyridin-3-yl)methanol (57.4 mg, 0.3 mmol, 1 eq.) at 85 ˚C. The crude product was purified by silica gel column chromatography (elution with 5% ethyl acetate in pentane) to give the title product as a colourless oil (69.6 mg, 85%).

HRMS (ESI$^+$) 323.2116 [M+H]$^+$, expected 323.2118; **^{1}H NMR** (400 MHz, $CDCl_3$) δ 7.57 (1H, d, *J* = 7.9 Hz, H-5), 7.42 (1H, d, *J* = 7.9 Hz, H-6), 2.93 (2H, t, *J* = 7.4 Hz, H-3), 2.79 (2H, t, *J* = 7.2 Hz, H-2), 2.61 (3H, s, H-10), 1.12 (9H, s, H-12); **^{13}C NMR** (101 MHz, $CDCl_3$) δ 214.1 (C1), 157.9 (C8), 145.5 (q, *J* = 34.2 Hz, C7), 138.4 (C4), 137.5 (C5), 121.8 (q, *J* = 273.9 Hz, C9), 118.1 (q, *J* = 3.0 Hz, C6), 44.3 (C11), 36.0 (C2), 26.8 (C3), 26.5 (C12), 22.4 (C10); **^{19}F NMR** (376 MHz, $CDCl_3$) δ −67.81 (3F); **IR** (thin film) (cm^{-1}): 1707, 1479, 1407, 1345, 1182, 1141, 1079, 842, 771, 640.

1-Cyclopropyl-3-(2-methyl-6-(trifluoromethyl)pyridin-3-yl)propan-1-one (82)

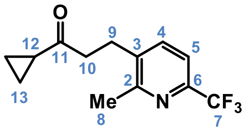

Prepared according to **General Procedure B** with cyclopropyl methyl ketone (29.7 μL, 0.3 mmol, 1 eq.) and (2-methyl-6-(trifluoromethyl)pyridin-3-yl)methanol (57.4 mg, 0.3 mmol, 1 eq.) at 85 ˚C. The crude product was purified by silica gel column chromatography (elution with 4–10% ethyl acetate in pentane) to give the title product as a yellow oil (55.5 mg, 72%).

Following the above procedure at 23 ˚C, the title product was isolated as a yellow oil (28.3 mg, 37%).

HRMS (ESI$^+$) 258.1105 [M+H]$^+$, expected 258.1100; **^{1}H NMR** (500 MHz, $CDCl_3$) δ: 7.58 (1H, d, *J* = 7.9 Hz, H-4), 7.43 (1H, d, *J* = 7.9 Hz, H-5), 2.97 (2H, td, *J* = 7.1, 1.6 Hz, H-9), 2.90 (2H, ddd, *J* = 8.4, 6.9, 1.5 Hz, H-10), 2.61 (3H, s, H-8), 1.92 (1H, tt, *J* = 7.8, 4.6 Hz, H-12), 1.03 (2H, dt, *J* = 4.6, 3.4 Hz, H-13_a), 0.90 (2H, dt, *J* = 8.0, 3.4 Hz, H-13_b); **^{13}C NMR** (126 MHz, $CDCl_3$) δ: 208.9 (C11), 157.9 (C2), 145.5 (q, *J* = 34.3 Hz, C6), 138.1 (C3), 137.4 (C4), 121.8 (q, *J* = 273.7 Hz, C7), 118.1 (q, *J* = 2.9 Hz, C5), 42.5 (C10), 26.5 (C9), 22.4 (C8), 20.8 (C12), 11.2 (C13); **^{19}F NMR** (470 MHz, $CDCl_3$) δ: −67.8 (3F, s) ; **IR** (thin film) (cm^{-1}): 1710, 1412, 1348, 1182, 1142, 1120, 1065, 905, 818, 761.

Unsuccessful Ketone Substrates

Commentary: A range of ketones were found to be incompatible in the reaction at 23 °C or 85 °C (**Supplementary Figures 4** and **5**).

Procedure: Experiments prepared according to **General Procedure B.**

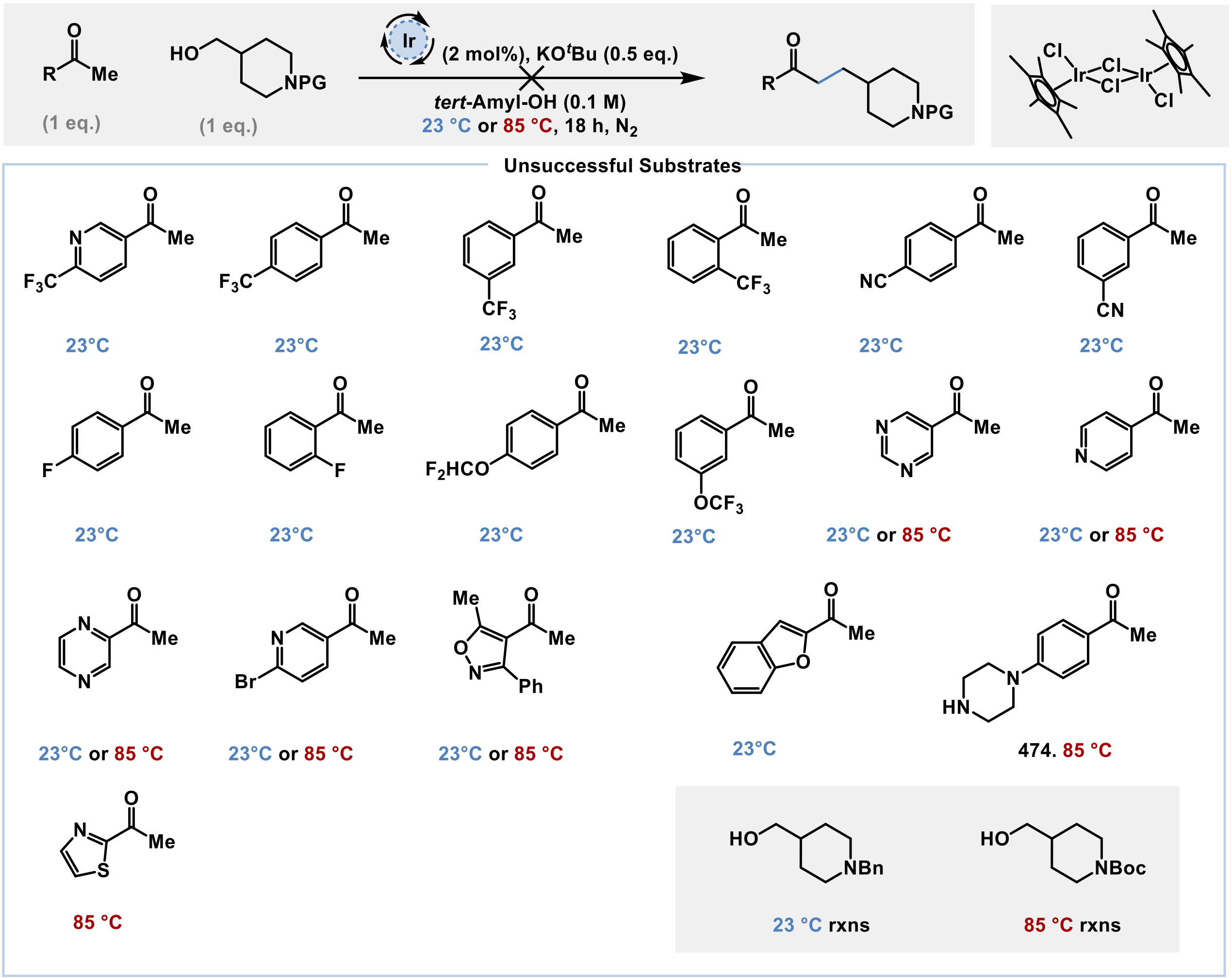

Supplementary Figure 4. Unsuccessful scope of ketone.

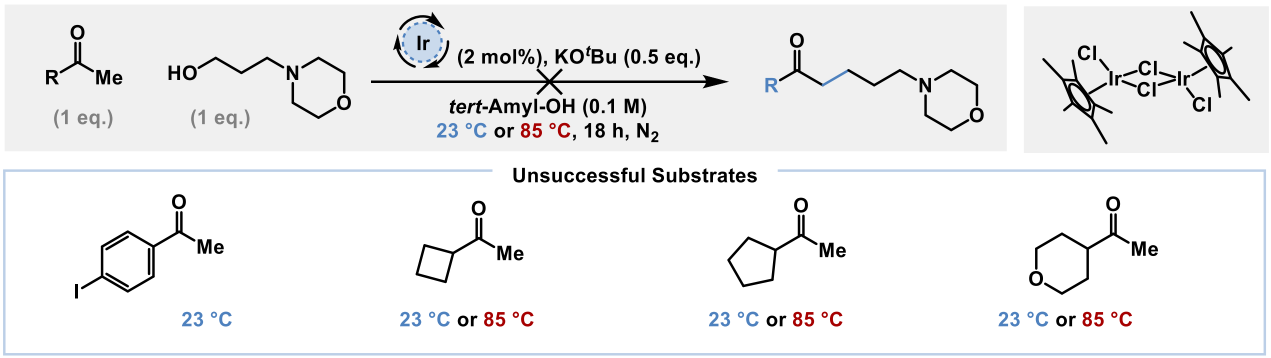

Supplementary Figure 5. Unsuccessful scope of ketone.

Derivatization of Hydrogen Borrowing Products

6-Bromo-1-(2-methyl-6-(trifluoromethyl)pyridin-3-yl)hexan-3-one (S82)

Commentary: Cyclopropyl ketone **82** could be derivatised to a straight chain analogue *via* exposure to HBr.[5]

Procedure:

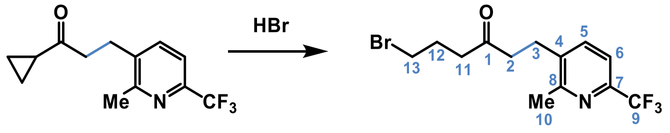

1-Cyclopropyl-3-(2-methyl-6-(trifluoromethyl)pyridin-3-yl)propan-1-one (24.0 mg, 0.093 mmol, 1.0 eq.) and aq. HBr (48% w/w, 0.4 mL) were added to a microwave vial equipped with a stir bar. The vial was capped and stirred at 40 °C for 16 h. The vial was cooled to rt and sat. aq. $NaHCO_3$ (15 mL) was added. The reaction mixture was extracted with CH_2Cl_2 (3 × 10 mL); the combined organic extracts were dried using Mg_2SO_4, filtered and concentrated *in vacuo* to yield a crude oil. This oil was purified by silica gel column chromatography (elution with 15% ethyl acetate in pentane +1% triethylamine) to give the title product as a colourless oil (27.3 mg, 87%).

HRMS (ESI$^+$) 338.0357 [M+H]$^+$, expected 338.0362; **^{1}H NMR** (400 MHz, $CDCl_3$) δ 7.58 (1H, d, *J* = 7.9 Hz, H-5), 7.44 (1H, d, *J* = 7.9 Hz, H-6), 3.44 (2H, t, *J* = 6.3 Hz, H-13), 2.96 (2H, t, *J* = 7.4 Hz, H-3), 2.77 (2H, t, *J* = 7.4 Hz, H-2), 2.63 (2H, t, *J* = 6.9 Hz, H-11), 2.61 (3H, s, H-10), 2.13 (2H, p, *J* = 6.6 Hz, H-12); **^{13}C NMR** (126 MHz, $CDCl_3$) δ 207.6 (C1), 157.9 (C8), 145.6 (q, *J* = 34.2 Hz, C7), 137.8 (C4), 137.4 (C5), 121.8 (q, *J* = 273.6 Hz, C9), 118.2 (q, *J* = 2.9 Hz, C6), 42.0 (C2), 40.8 (C11), 33.3 (C13), 26.3 (C12), 26.2 (C3), 22.4 (C10); **^{19}F NMR** (377 MHz, $CDCl_3$) δ −67.8 (3F); **IR** (thin film) (cm^{-1}): 1718, 1407, 1345, 1261, 1182, 1139, 911, 847, 742, 643.

Derivatization of Ph* Group in Hydrogen Borrowing Products

Commentary: The Ph* group in a host of products of the hydrogen borrowing reaction could be derivatized to a carboxylic acid functional group. If a substrate contained a basic nitrogen functional group, then the product could be isolated *without column chromatography* as the corresponding HCl salt.

4-Carboxy-*N,N*-dimethylbutan-1-aminium chloride (83)

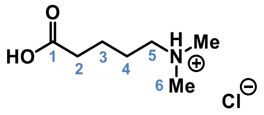

Prepared according to **General Procedure C**, with 5-(dimethylamino)-1-(2,3,4,5,6-pentamethylphenyl)pentan-1-one (27.5 mg, 0.1 mmol, 1.0 eq.) and hydrochloric acid (37% aq. 12 M, 0.130 mL) at 65 ˚C. The title product was obtained as a colourless solid (17.6 mg, 97%).

M.p.: 134–136 °C (from H_2O); **HRMS** (ESI$^+$) 146.1181 [M−Cl]$^+$, expected 146.1176; ^{1}**H NMR** (400 MHz, D_2O) δ: 3.11 (2H, t, *J* = 7.8, 1.7 Hz, H-5), 2.82 (6H, d, *J* = 1.8, Hz, H-6), 2.41 (2H, td, *J* = 7.2, 1.8 Hz, H-2), 1.77–1.65 (2H, m, H-4), 1.65–1.55 (2H, m, H-3); ^{13}**C NMR** (101 MHz, D_2O) δ: 177.9 (C1), 57.2 (C5), 42.5 (C6), 32.8 (C2), 23.3 (C4), 20.9 (C3); **IR** (solid) (cm^{-1}): 2690, 1725, 1686, 1403, 1259, 1187, 1171, 1050, 965, 864.

***N*-Benzyl-4-carboxybutan-1-aminium chloride (84)**

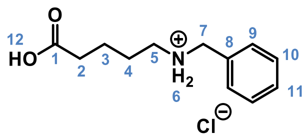

Prepared according to **General Procedure C**, with 5-(benzylamino)-1-(2,3,4,5,6-pentamethylphenyl)pentan-1-one (33.7 mg, 0.1 mmol, 1.0 eq.) and hydrochloric acid (37% aq. 12 M, 0.130 mL) at 65 ˚C. The title product was obtained as a colourless solid (20.4 mg, 84%).

M.p.: 136–138 °C (from H_2O); **HRMS** (ESI$^+$) 208.1332 [M−Cl]$^+$, expected 208.1332; ^{1}**H NMR** (400 MHz, D_2O) δ 7.59–7.43 (5H, m, H-9, H-10, H-11), 4.24 (2H, s, H-7), 3.09 (2H, t, *J* = 7.6 Hz, H-5), 2.43 (2H, t, *J* = 7.0 Hz, H-2), 1.80–1.58 (4H, m, H-3, H-4); ^{13}**C NMR** (101 MHz, D_2O) δ 178.0 (C1), 130.7 (C8), 129.8 (C9), 129.6 (C11), 129.2 (C10), 50.9 (C7), 46.5 (C5), 32.9 (C2), 24.8 (C4), 21.1 (C3); **IR** (solid) (cm^{-1}): 2954, 2776, 2436, 1720, 1461, 1441, 1223, 930, 744, 698.

4-(4-Carboxybutyl)morpholin-4-ium chloride (85)

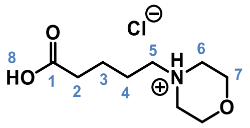

Prepared according to **General Procedure C**, with 5-morpholino-1-(2,3,4,5,6-pentamethylphenyl)pentan-1-one (31.8 mg, 0.1 mmol, 1.0 eq.) and hydrochloric acid (37% aq. 12 M, 0.130 mL) at 65 ˚C. The title product was obtained as a colourless solid (19.7 mg, 88%).

M.p.: 138–140 °C (from H_2O); **HRMS** (ESI$^+$) 188.1280 [M−Cl]$^+$, expected 188.1281 ; ^{1}**H NMR** (400 MHz, D_2O) δ 4.04 (2H, dd, *J* = 13.4, 3.6 Hz, H-7_a), 3.73 (2H, ddd, *J* = 13.6, 11.9, 2.1 Hz, H-7_b), 3.45 (2H, dd, *J* = 12.6, 2.0 Hz, H-6_a), 3.20–3.05 (4H, m, H-5, H-6_b), 2.38 (2H, t, *J* = 7.2 Hz, H-2), 1.79–1.66 (2H, m, H-4),

1.66–1.51 (2H, m, H-3); **^{13}C NMR** (101 MHz, D_2O) δ 177.9 (C1), 63.8 (C7), 56.7 (C5), 51.6 (C6), 32.9 (C2), 22.4 (C4), 21.1 (C3); **IR** (solid) (cm^{-1}): 3431, 2955, 2676, 2621, 1724, 1401, 1254, 1172, 1084, 868.

4-(2-Carboxyethyl)piperidin-1-ium chloride (86)

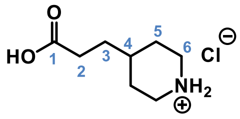

Prepared according to **General Procedure C**, with 1-(2,3,4,5,6-pentamethylphenyl)-3-(pyrimidin-2-yl)propan-1-one (38.8 mg) and hydrochloric acid (37% aq. 12 M, 0.130 mL) at 65 ˚C. The title product was obtained as a colourless solid (18.4 mg, 95%).

M.p.: Decomposed at T >240 °C (from H_2O); **HRMS** (ESI$^+$) 158.1180 [M−Cl]$^+$, expected 158.1176; **^{1}H NMR** (400 MHz, D_2O) δ: 3.32 (2H, dt, *J* = 12.8, 2.5 Hz, H-6$_a$), 2.88 (2H, dd, *J* = 14.4, 11.4 Hz, H-6$_b$), 2.35 (2H, td, *J* = 6.9, 1.7 Hz, H-2), 1.87 (2H, dd, *J* = 14.2, 3.5 Hz, H-5$_a$), 1.53 (3H, app t, *J* = 6.6 Hz, H-3), 1.30 (2H, td, *J* = 13.6, 6.9 Hz, H-5$_b$); **^{13}C NMR** (101 MHz, D_2O) δ: 178.6 (C1), 43.9 (C6), 32.4 (C4), 30.7 (C2), 30.0 (C3), 27.9 (C5); **IR** (solid) (cm^{-1}): 2835, 2812, 1718, 1381, 1235, 1135, 1075, 978, 960, 855.

1-((3-(2-Carboxyethyl)bicyclo[1.1.1]pentan-1-yl)methyl)pyrrolidin-1-ium chloride (87)

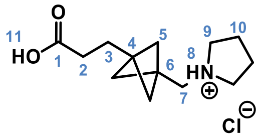

Prepared according to **General Procedure C**, with 3-(1-Benzylpipeoxridin-4-yl)-1-(2,3,4,5,6-pentamethylphenyl)propan-1-one (35.4 mg, 0.1 mmol, 1.0 eq.) and hydrochloric acid (37% aq. 12 M, 0.130 mL) at 65 ˚C. The title product was obtained as a colourless gum (19.2 mg, 74%).

HRMS (ESI$^+$) 224.1644 [M−Cl]$^+$, expected 224.1645; **^{1}H NMR** (400 MHz, D_2O) δ 3.72 (2H, dd, *J* = 11.2, 5.9 Hz, H-9$_a$), 3.37 (2H, s, H-7), 3.11 (2H, dt, *J* = 13.9, 7.7 Hz, H-9$_b$), 2.42 (2H, t, *J* = 7.2 Hz, H-2), 2.23–2.10 (2H, m, H-10$_a$), 2.02 (2H, dt, *J* = 11.6, 5.5 Hz, H-10$_b$), 1.89–1.77 (8H, m, H-3, H-5); **^{13}C NMR** (126 MHz, $CDCl_3$) δ 181.8 (C1), 58.6 (C7), 57.2 (C9), 52.7 (C5), 42.3 (C4), 37.4 (C6), 33.7 (C2), 28.6 (C3), 25.4 (C10); **IR** (thin film) (cm^{-1}): 3417, 2973, 2720, 1731, 1714, 1406, 1267, 1194, 952, 754.

8-Chlorooctanoic acid (88)

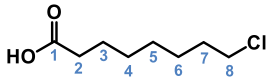

Prepared according to **General Procedure C**, with 8-chloro-1-(2,3,4,5,6-pentamethylphenyl)octan-1-one (27.4 mg, 0.089 mmol, 1 eq.) and hydrochloric acid (37% aq. 12 M, 0.130 mL) at 65 °C. H_2O (5 mL) was added and the reaction mixture was then extracted with $CHCl_3$ (3 × 10 mL); the combined organic extracts were dried using Mg_2SO_4, filtered and concentrated *in vacuo* to yield a crude oil. This oil was purified by silica gel column chromatography (elution with 0–2% methanol in CH_2Cl_2) to give the title product as a colourless oil (11.9 mg, 78%).

HRMS (ESI$^-$) 177.0682, 179.0653 [3:1, M−H]$^-$, expected 177.0688, 179.06582 [3:1]; **^{1}H NMR** (400 MHz, $CDCl_3$) δ 3.53 (2H, t, *J* = 6.7 Hz, H-8), 2.36 (2H, t, *J* = 7.5 Hz, H-2), 1.77 (2H, p, *J* = 6.9 Hz, H-7), 1.65 (2H, ddt, *J* = 14.5, 9.6, 5.8 Hz, H-3), 1.49–1.39 (2H, m, H-6), 1.35 (4H, dq, *J* = 8.6, 4.5 Hz, H-4, H-5); **^{13}C NMR** (126 MHz, $CDCl_3$) δ 179.9 (C1), 45.2 (C8), 34.1 (C2), 32.7 (C7), 29.0 (C4), 28.7 (C5), 26.8 (C6), 24.7 (C3).

The spectral data matched that previously reported in the literature.[14]

8-Bromooctanoic acid (89)

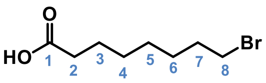

Prepared according to **General Procedure C**, with 8-bromo-1-(2,3,4,5,6-pentamethylphenyl)octan-1-one (30.5 mg, 0.086 mmol, 1 eq.) and hydrochloric acid (37% aq. 12 M, 0.130 mL) at 40 °C. H_2O (5 mL) was added and the reaction mixture was then extracted with $CHCl_3$ (3 × 10 mL); the combined organic extracts were dried using Mg_2SO_4, filtered and concentrated *in vacuo* to yield a crude oil. This oil was purified by silica gel column chromatography (elution with 0–2% methanol in CH_2Cl_2) to give the title product as a colourless oil (13.5 mg, 70%).

HRMS (ESI$^-$) 221.0176, 223.0277 [1:1, M−H]$^-$, expected 221.0183, 223.0162 [1:1]; **^{1}H NMR** (400 MHz, $CDCl_3$) δ 3.40 (2H, t, *J* = 6.8 Hz, H-8), 2.36 (2H, t, *J* = 7.5 Hz, H-2), 1.85 (2H, p, *J* = 6.9 Hz, H-7), 1.73–1.59 (2H, m, H-3), 1.52–1.40 (2H, m, H-6), 1.35 (4H, dt, *J* = 6.7, 3.3 Hz, H-4, H-5); **^{13}C NMR** (126 MHz, $CDCl_3$) δ 179.6 (C1), 34.0 (C2), 34.0 (C8), 32.8 (C7), 29.0 (C4), 28.5 (C5), 28.1 (C6), 24.7 (C3).

The spectral data matched that previously reported in the literature.[15,16]

2-(2-Carboxyethyl)pyrimidin-1-ium chloride (90)

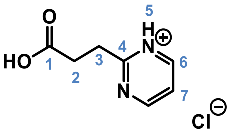

Prepared according to **General Procedure C**, with 1-(2,3,4,5,6-pentamethylphenyl)-3-(pyrimidin-2-yl)propan-1-one (28.2 mg, 0.1 mmol, 1.0 eq.) and hydrochloric acid (37% aq. 12 M, 0.130 mL) at 65 °C. The title product was obtained as a colourless gum (13.5 mg, 72%).

HRMS (ESI+) 153.0657 [M−Cl]+, expected 153.0659; **^{1}H NMR** (400 MHz, D_2O) δ 9.01 (2H, d, *J* = 5.3 Hz, H-6), 7.78 (1H, t, *J* = 5.3 Hz, H-7), 4.85–4.69 (1H, m, H-5), 3.37 (2H, t, *J* = 6.7 Hz, H-3), 2.99 (2H, t, *J* = 6.6 Hz, H-2); **^{13}C NMR** (126 MHz, D_2O) δ 176.3 (C1), 165.7 (C4), 157.5 (C6), 120.6 (C7), 30.7 (C3), 30.5 (C2); **IR** (solid) (cm^{-1}): 3421, 2838, 1737, 1713, 1620, 1577, 1433, 1211, 1001, 675.

3-(4-Carboxybutyl)pyridin-1-ium chloride (91)

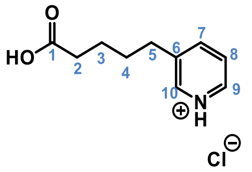

Prepared according to **General Procedure C**, with 1-(2,3,4,5,6-pentamethylphenyl)-5-(pyridin-3-yl)pentan-1-one (31.0 mg, 0.1 mmol, 1.0 eq.) and hydrochloric acid (37% aq. 12 M, 0.260 mL) at 65 °C. The title product was obtained as a colourless solid (20.4 mg, 96%).

M.p.: 118–120 °C (from H_2O); **HRMS** (ESI+) 180.1017 [M−Cl]+, expected 180.1019; **^{1}H NMR** (400 MHz, D_2O) δ 8.64 (1H, d, *J* = 1.9 Hz, H-10), 8.61 (1H, d, *J* = 5.8 Hz, H-9), 8.49 (1H, dt, *J* = 8.2, 1.7 Hz, H-7), 7.98 (1H, dd, *J* = 8.1, 5.8 Hz, H-8), 2.89 (2H, t, *J* = 7.5 Hz, H-5), 2.42 (2H, t, *J* = 7.2 Hz, H-2), 1.74 (2H, ddt, *J* = 11.6, 8.3, 4.3 Hz, H-4), 1.63 (2H, dq, *J* = 8.5, 7.1 Hz, H-3); **^{13}C NMR** (126 MHz, D_2O) δ 178.6 (C1), 147.1 (C7), 142.8 (C6), 140.3 (C10), 138.5 (C9), 126.9 (C8), 33.3 (C2), 31.5 (C5), 28.8 (C4), 23.4 (C3); **IR** (solid) (cm^{-1}): 2948, 1710, 1556, 1397, 1235, 1188, 1173, 804, 687.

1-(4-(2-Carboxyethyl)phenyl)-1*H*-imidazol-3-ium chloride (92)

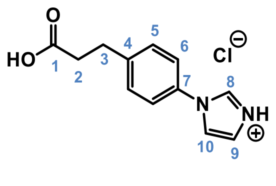

Prepared according to **General Procedure C**, with 3-4-(1*H*-Imidazol-1-yl)phenyl)-1-(2,3,4,5,6-pentamethylphenyl)propan-1-one (34.7 mg, 0.1 mmol, 1.0 eq.) and hydrochloric acid (37% aq. 12 M, 0.260 mL) at 65 °C. The title product was obtained as a colourless solid (24.3 mg, 96%).

M.p.: 156–158 °C (from H_2O); **HRMS** (ESI+) 217.0972 [M−Cl]+, expected 217.0972; **^{1}H NMR** (400 MHz, D_2O) δ 9.11 (1H, d, *J* = 1.6 Hz, H-8), 7.83 (1H, t, *J* = 1.8 Hz, H-9 or H-10), 7.63 (1H, t, *J* = 1.7 Hz, H-10 or H-

9), 7.55 (2H, d, *J* = 8.4 Hz, H-6), 7.48 (2H, d, *J* = 8.4 Hz, H-5), 3.00 (2H, t, *J* = 7.3 Hz, H-3), 2.74 (2H, t, *J* = 7.3 Hz, H-2); **^{13}C NMR** (101 MHz, D_2O) δ 177.5 (C1), 142.9 (C4), 133.5 (C8), 133.0 (C7), 130.0 (C5), 122.4 (C6), 121.3 (C9 or C10), 120.2 (C10 or C9), 34.9 (C2), 29.7 (C3); **IR** (solid) (cm^{-1}): 3358, 3117, 2838, 1721, 1545, 1349, 1206, 1188, 827, 619.

Unsuccessful Ph* Derivatizations

Commentary: A host of Ph* substrates were incompatible under acidic derivatization conditions. Slow conversion was observed with a host of substrates which possessed a basic nitrogen site relatively close to the carbonyl (a mixture of the HCl salt of the product and HCl salt of the starting material was observed, which were inseparable) or no conversion was observed (observe HCl salt of the starting material only, **Supplementary Figure 6**). Complex mixtures were observed with substrates that possessed functionality which was generally unstable to acidic conditions (**Supplementary Figure 7**).

Procedure: Experiments prepared according to **General Procedure C.**

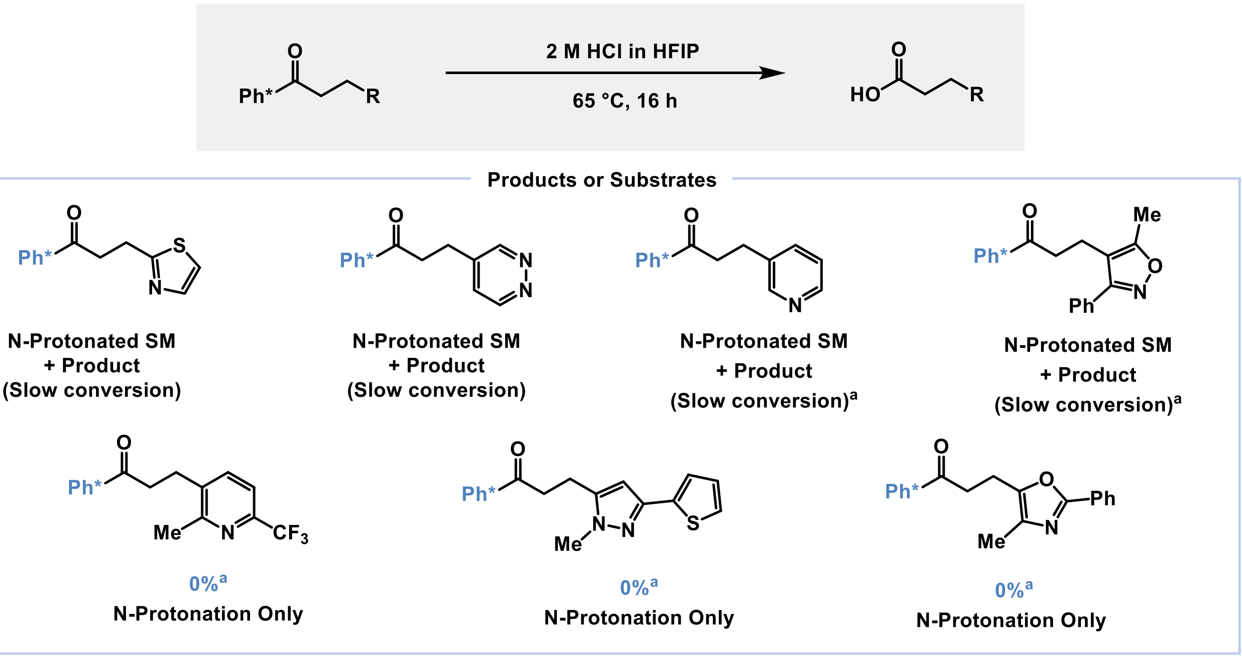

Supplementary Figure 6. Poor yielding or slowly converting substates in the acidic derivatization reaction. [a] 4 M HCl in HFIP.

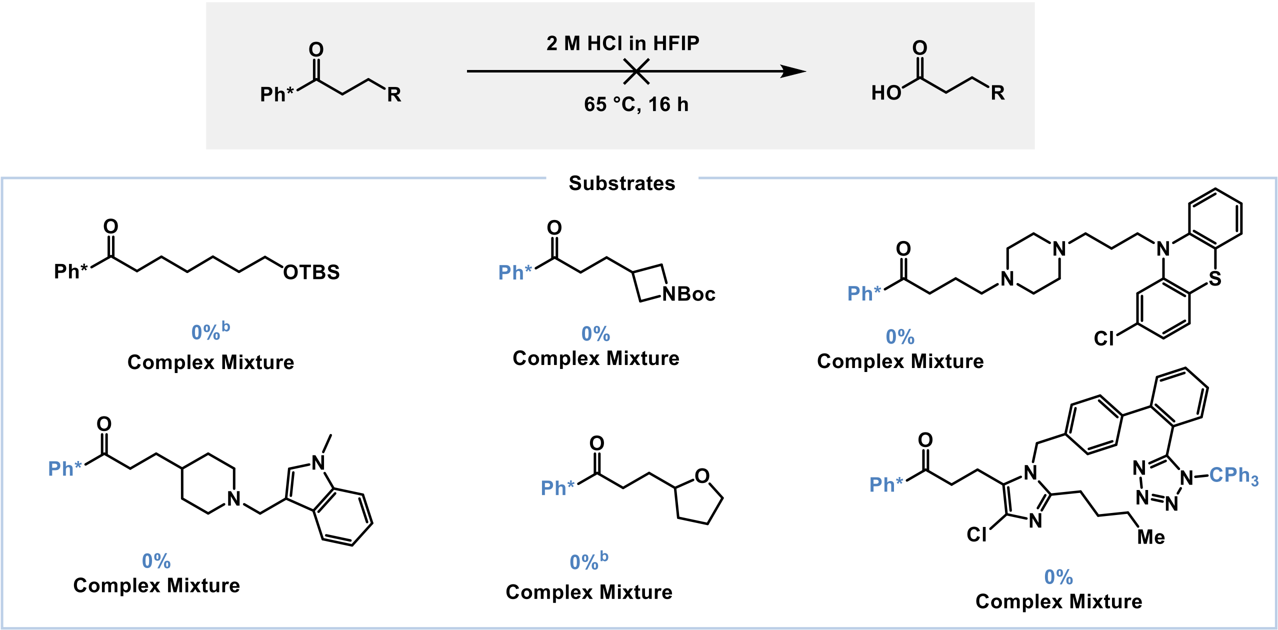

Supplementary Figure 7. Incompatible substrates in the acidic derivatization reaction. [a] 4 M HCl in HFIP. [b] 40 °C.

Scale-Up of the Hydrogen Borrowing Reaction at 23 °C including Ph* Derivatization

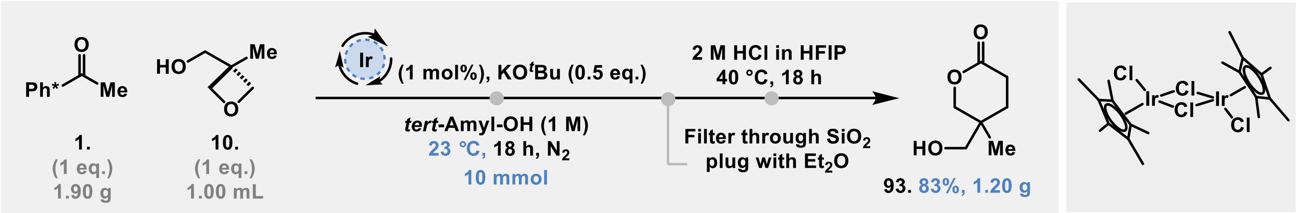

Supplementary Figure 8. Telescoped hydrogen borrowing and derivatisation. Yield is of isolated material.

Procedure: $[Cp^*IrCl_2]_2$ (79.7 mg, 1 mol%), Ph* methyl ketone (1.90 g, 10.0 mmol, 1 eq.) and powdered potassium *tert*-butoxide (0.561 g, 5.0 mmol, 0.5 eq.) were combined sequentially in a 30 mL microwave vial equipped with a large stir bar. The vial was capped and evacuated and backfilled with nitrogen five times. *tert*-Amyl alcohol was sparged in a two-neck flask for 15 minutes and 3-methyloxetan-3-yl)methanol and was sparged in a small vial for 10 minutes. *tert*-Amyl alcohol (10.0 mL, 1 M) and 3-methyloxetan-3-yl)methanol (1.00 mL, 10.0 mmol, 1 eq.) were then added sequentially to the vial. The vial was placed at the centre of a stir plate and stirred at 900 rpm at 23 ˚C for 18 h. The reaction mixture was then filtered through a short pad of silica gel (elution with diethyl ether) and concentrated *in vacuo.*

Hydrochloric acid (37% aq. 12 M, 13.0 mL) was added to a solution of this solid in HFIP (88.0 mL, 2 M HCl in HFIP) in a 200 mL Schlenk tube equipped with a large stir bar. The tube was sealed and the reaction solution was stirred for 16 h at 40 ˚C, after which the vial was cooled to rt and concentrated *in vacuo*. The reaction mixture was extracted with ethyl acetate (3 × 30 mL); the combined organic extracts were dried using $MgSO_4$, filtered, and concentrated *in vacuo*. The resulting oil was then purified by silica gel column chromatography (elution with 0–50% ethyl acetate in pentane) to give 5-(hydroxymethyl)-5-methyltetrahydro-2*H*-pyran-2-one as a pale yellow oil (1.20 g, 83%) (**Supplementary Figure S8**).

5-(Hydroxymethyl)-5-methyltetrahydro-2*H*-pyran-2-one (93)

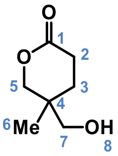

HRMS (ESI+) 145.0855 [M+H]+, expected 145.0859; **^{1}H NMR** (400 MHz, $CDCl_3$) δ: 4.25 (1H, dd, *J* = 11.4, 0.9 Hz, H-5$_a$), 3.97 (1H, dd, *J* = 11.4, 0.8 Hz, H-5$_b$), 3.53 (1H, d, *J* = 10.7 Hz, H-7$_a$), 3.47 (1H, d, *J* = 10.7 Hz, H-7$_b$), 2.55 (2H, t, *J* = 7.4 Hz, H-2), 2.04 (1H, s, H-8) 1.90–1.76 (1H, m, H-3$_a$), 1.63 (1H, dtd, *J* = 13.9, 7.0, 0.8 Hz, H-3$_b$), 1.05 (3H, s, H-6); **^{13}C NMR** (101 MHz, $CDCl_3$) δ: 172.6 (C1), 74.2 (C5), 67.7 (C7), 34.8 (C4), 28.2 (C3), 27.4 (C2), 20.9 (C6).

The spectral data matched that previously reported in the literature.[17]

Mechanistic Studies

Observation of Iridium Hydrides (95)

Commentary: We sought to observe iridium hydride species under the optimised reaction conditions (**General Procedure A**, but without Ph* methyl ketone **1**). This was to validate their air sensitivity to account for the lack of hydrogen borrowing reaction observed between Ph* methyl ketone **1** and heptanol **2** under air at room temperature (**Supplementary Figure 9**).[18]

Procedures and NMR spectra:

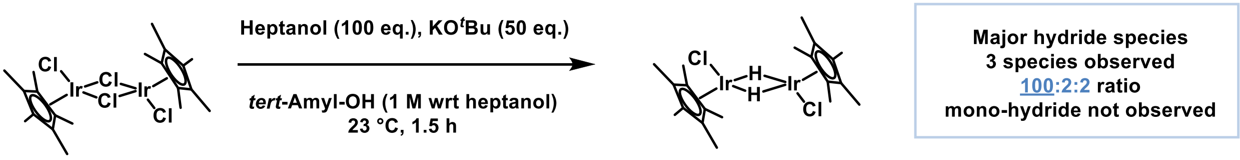

95. δ_H (d^8-toluene)= −13.63 ppm

No Ir-H observed after exposure to O_2 (2 mins)

Supplementary Figure 9. Observation of iridium dihydride **95** and its sensitivity to oxygen.

$[Cp^*IrCl_2]_2$ (4.8 mg, 0.00602 mmol, 1 eq.), powdered potassium *tert*-butoxide (33.7 mg, 0.301 mmol, 50 eq.) and *tert*-amyl alcohol (0.60 mL) were combined in a microwave vial equipped with a stir bar. Heptanol (85 µL, 0.6 mmol, 100 eq.) was added quickly and the vial was capped, evacuated and backfilled with nitrogen five times. The reaction mixture was stirred for 1.5 h at 23 ˚C. The reaction was concentrated *in vacuo*, taken into a glovebox and redissolved in freeze-pump-thawed d^8-toluene (0.75 mL) (**Supplementary Figure S9**).

This solution was transferred to a J-Young NMR tube in the glovebox and the ^{1}H NMR spectrum recorded (**Supplementary Figure S10**). Analysis of the spectra in the region from 0‒−20 ppm revealed the dihydride as the major hydride species at (400 MHz, d^8-toluene) δ: −13.63 ppm. Oxygen was then bubbled through this solution for 2 minutes (*via* a balloon and long needle) and the ^{1}H NMR spectrum recorded; no iridium hydride species were observed (**Supplementary Figure S11**). This solution was then freeze-pump-thawed and the atmosphere replaced with nitrogen, the NMR tube was shaken vigorously and then left to sit for 18 h. The ^{1}H NMR spectrum was then recorded; no iridium hydride species were observed (**Supplementary Figure S12**).

[Monohydride]: **^{1}H NMR** (300 MHz, d^8-toluene) δ: 1.62 (30H, s), −13.85 (1H, s).[18]

[Dihydride]: **^{1}H NMR** (300 MHz, d^8-toluene) δ: 1.48 (30H, s), −13.67 (2H, s).[18]

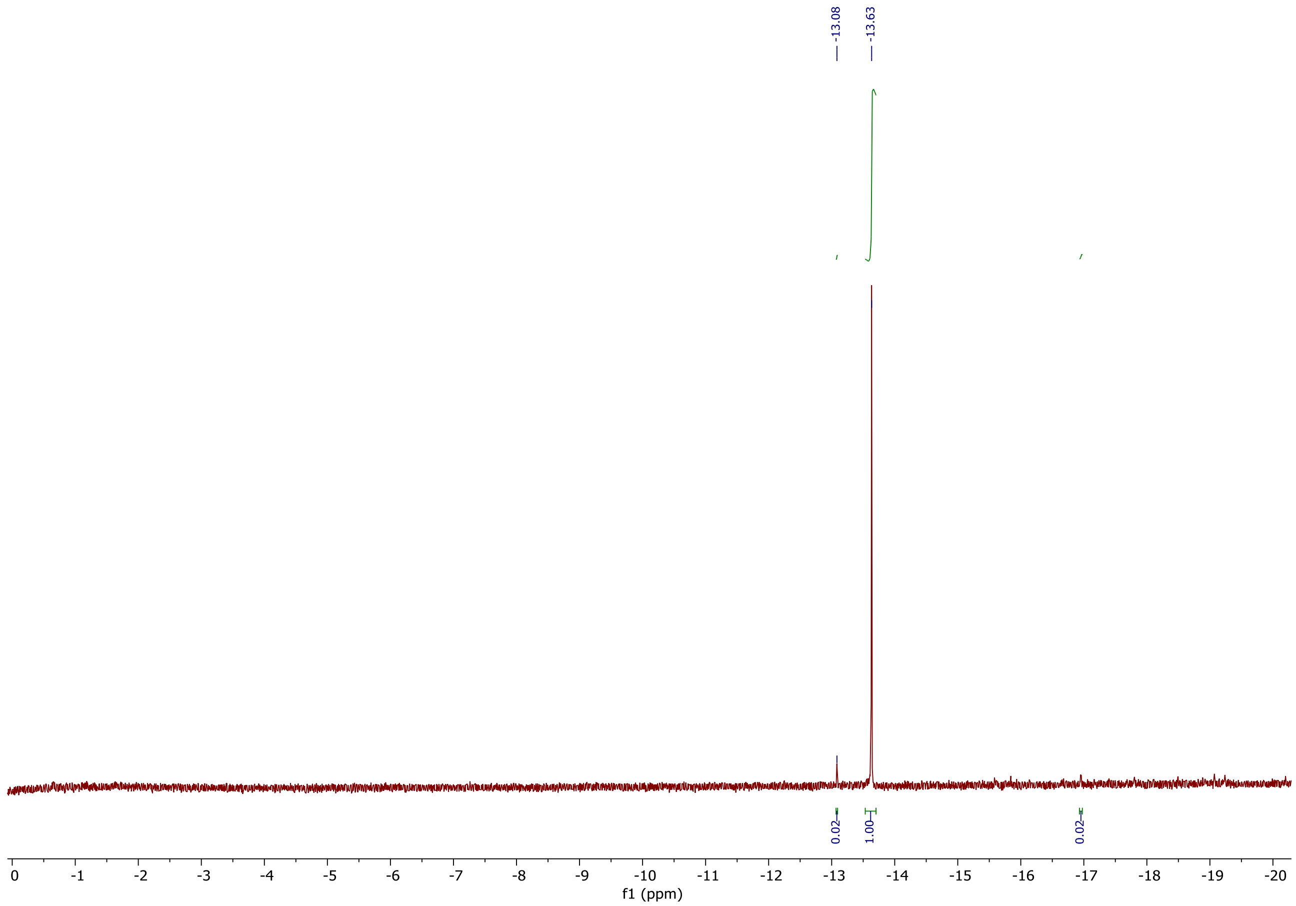

Supplementary Figure 10. Observation of dihydride with 100 eq. heptanol (KO^{t}Bu) after 1.5 h. ^{1}H NMR (400 MHz, d^8-toluene).

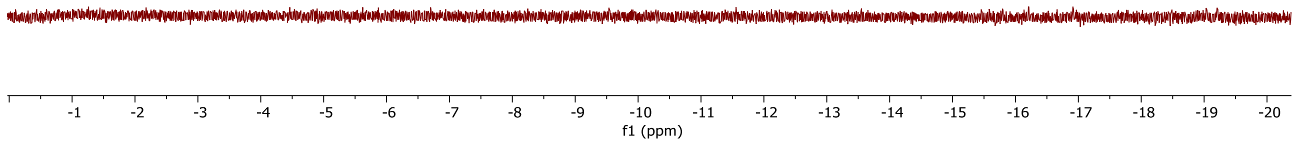

Supplementary Figure 11. After bubbling O_2 through sample for 2 minutes with 100 eq. heptanol (KO^{t}Bu) after 1.5 h. ^{1}H NMR (400 MHz, d^8-toluene).

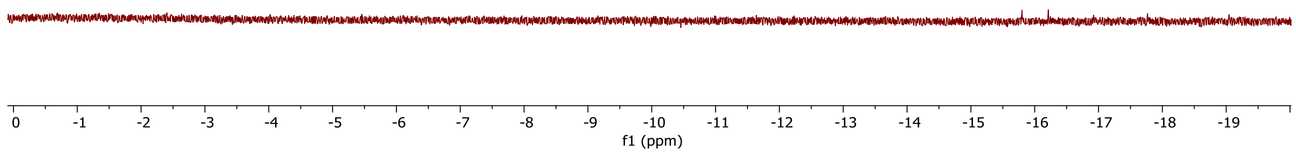

Supplementary Figure 12. Sample with 100 eq. heptanol (KO^{t}Bu) after 1.5 h, then O_2 for 2 minutes, then removing O_2 *via* freeze-pump-thaw and replacement with N_2, then leaving for 18 h. ^{1}H NMR (400 MHz, *d*^{8}-toluene).

Commentary: The oxygen sensitivity of corresponding monohydride was demonstrated by running a similar experiment but using fewer equivalents of alcohol (**Supplementary Figure S13**).

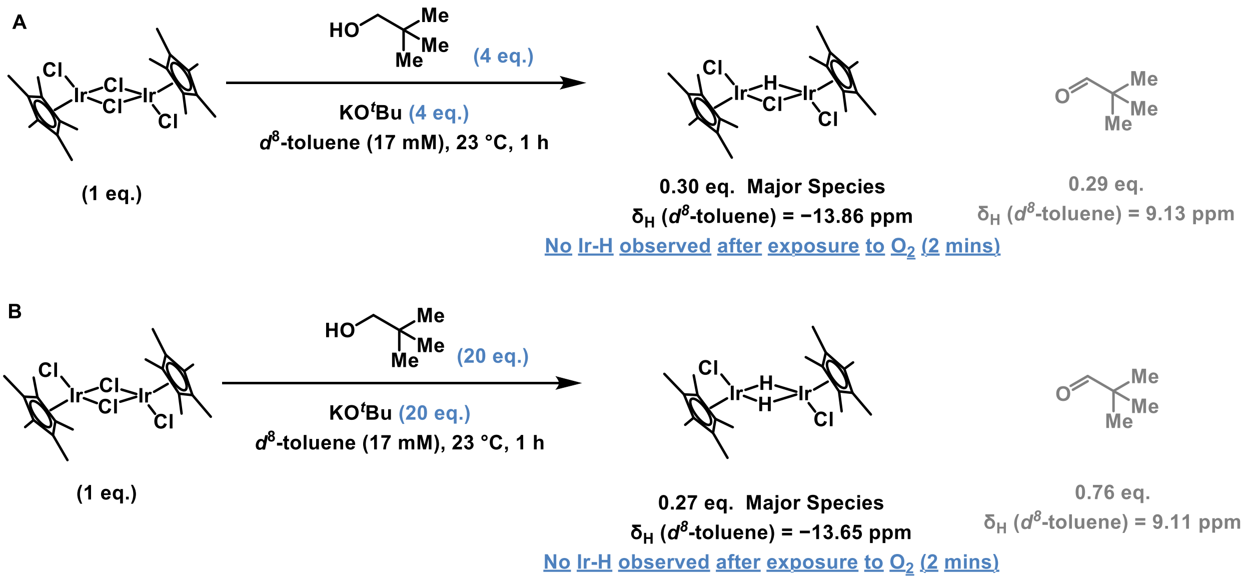

Supplementary Figure 13. Observation of iridium monohydride and dihydride **95** respectively and their sensitivity to oxygen. A. with 4 equivalents of alcohol and base. B. with 20 equivalents of alcohol and base.

$[Cp^*IrCl_2]_2$ (10.0 mg, 0.0126 mmol, 1 eq.), 2,2-dimethylpropanol (4.4 mg, 0.0502 mmol, 4 eq. or 22.1 mg, 0.251 mmol, 20 eq.) and powdered potassium *tert*-butoxide (5.6 mg, 0.0502 mmol, 4 eq. or 28.2 mg, 0.251 mmol, 20 eq.) were combined in a microwave vial equipped with a stir bar. The vial was capped and evacuated and backfilled with nitrogen five times. The vial was taken into a glovebox and freeze-pump-thawed d^8-toluene (0.75 mL) was added. This solution was stirred for 1 h before the vial was pierced with an exit needle and freeze-pump-thawed mesitylene (3.5 µL, 0.0252 mmol, 2 eq.) was added. The solution was transferred to a J-Young NMR tube in the glovebox and the ^{1}H NMR spectrum recorded (**Supplementary Figure S13**).

Analysis of the spectra in the region from 0––20 ppm revealed the monohydride was the major hydride species at (400 MHz, d^8-toluene) δ: −13.86 ppm when using 4 equivalents of 2,2-dimethylpropanol (**Supplementary Figure S14**). With 20 equivalents of 2,2-dimethylpropanol the dihydride **95** was the major hydride species observed at (400 MHz, d^8-toluene) δ: −13.65 ppm (**Supplementary Figure S16**). Oxygen was then bubbled through each solution for 2 minutes (*via* a balloon and long needle) and the ^{1}H NMR spectrum recorded; no iridium hydride species were observed (**Supplementary Figure S15** and **Supplementary Figure S17** respectively).

[Monohydride]: ^{1}H NMR (300 MHz, d^8-toluene) δ: 1.62 (30H, s), −13.85 (1H, s).[18]
[Dihydride]: ^{1}H NMR (300 MHz, d^8-toluene) δ: 1.48 (30H, s), −13.67 (2H, s).[18]
[Pivaldehyde]: ^{1}H NMR (400 MHz, d^8-toluene) δ: 9.13 (1H, s), 0.73 (9H, s).

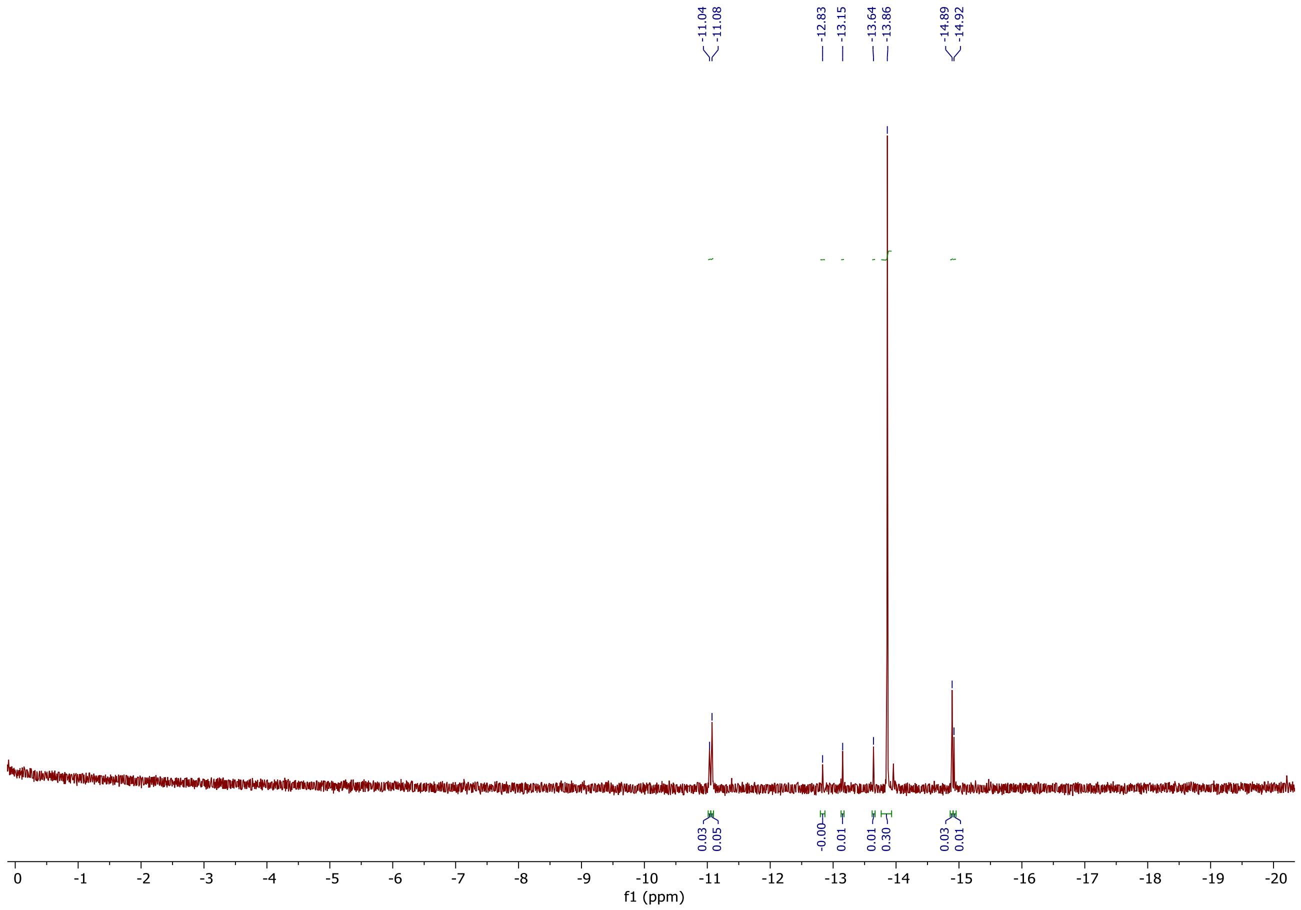

Supplementary Figure 14. Observation of predominantly monohydride with 4 eq. 2,2-dimethylpropanol after 1 h. Integrals are referenced against mesitylene internal standard. ^{1}H NMR (400 MHz, d^8-toluene).

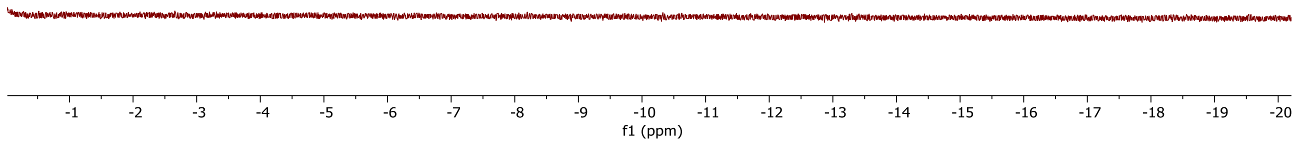

Supplementary Figure 15. After bubbling O_2 through sample of predominantly monohydride for 2 minutes (observed with 4 eq. of 2,2-dimethylpropan-1-ol after 1 h). ^{1}H NMR (400 MHz, d^8-toluene).

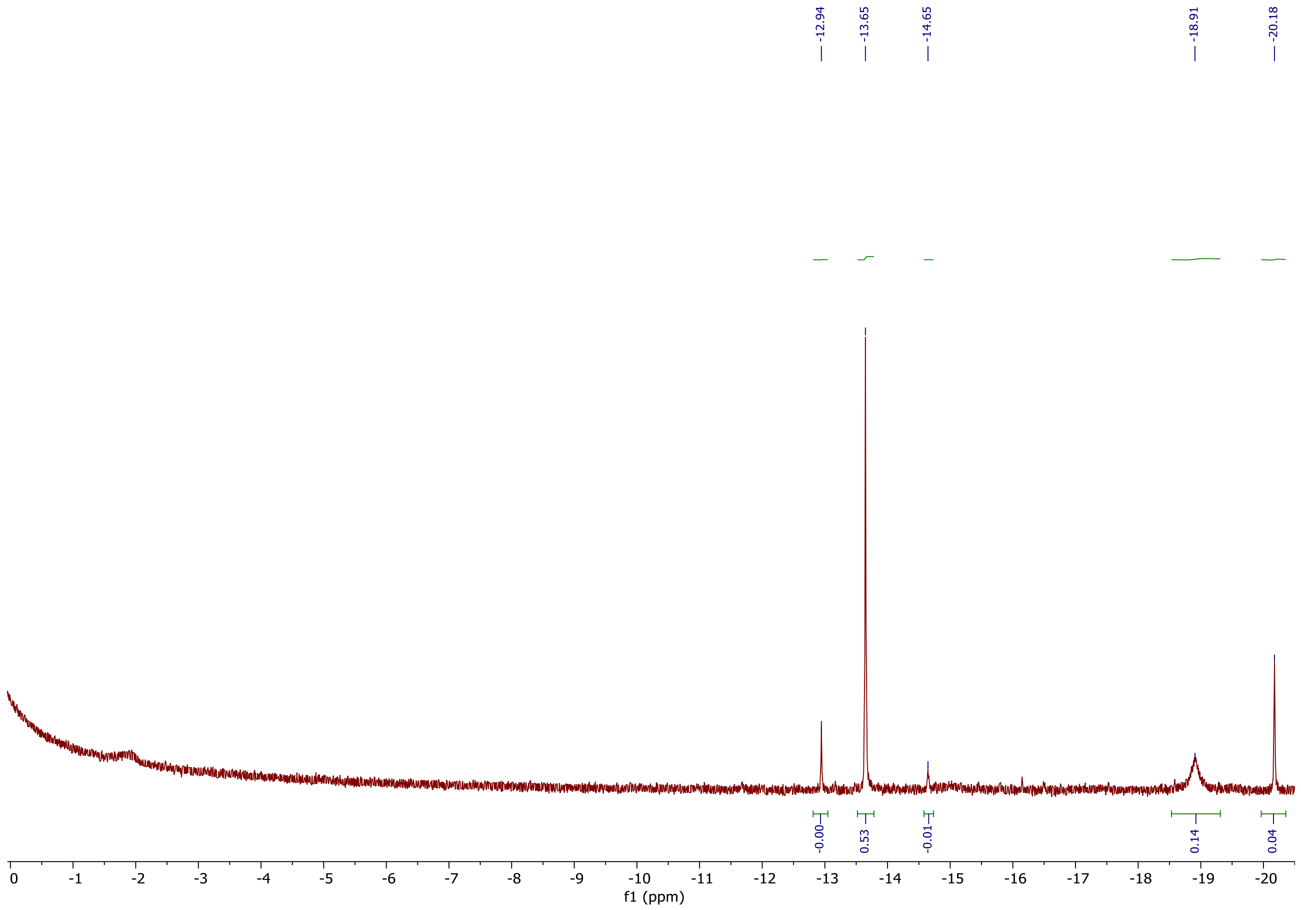

Supplementary Figure 16. Observation of predominantly dihydride with 20 eq. 2,2-dimethylpropanol after 1 h. Integrals are referenced against mesitylene internal standard. ^{1}H NMR (400 MHz, d^{8}-toluene).

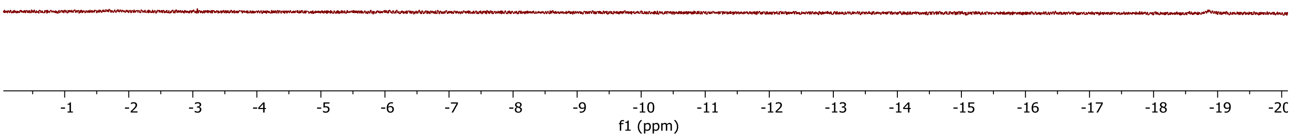

Supplementary Figure 17. After bubbling O_2 through sample of predominantly dihydride for 2 minutes (observed with 4 eq. of 2,2-dimethylpropan-1-ol after 1 h). ^{1}H NMR (400 MHz, d^8-toluene).

Effect of Gas Identity in Reaction Headspace

Commentary: The reaction between Ph* methyl ketone and heptanol was run at 23 ˚C and 85 ˚C under nitrogen, air, oxygen and air with an argon balloon added (**Supplementary Figure 18**). The reactions under oxygen suggests that molecular oxygen is the responsible component in air for shutting down the reaction. The reactions under air with an argon balloon added demonstrate that this is an ineffective procedure to achieve a sufficiently inert atmosphere to allow a reaction the proceed.[6]

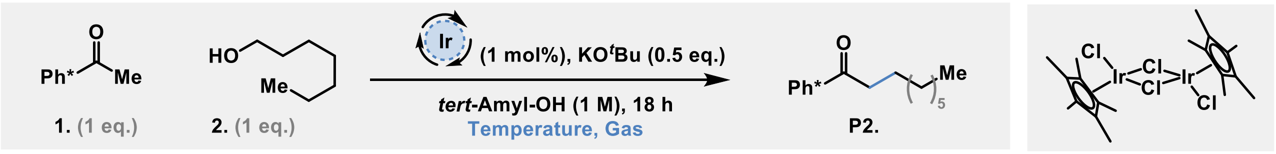

	23 °C				85 °C			
	Entry 1	Entry 2	Entry 3	Entry 4	Entry 5	Entry 6	Entry 7	Entry 8
Gas	N_2	Air	Air then Ar balloon	Oxygen	N_2	Air	Air then Ar balloon	Oxygen
P2	91% (90%)	3%	3%	0%	88%	21%	28%	3%
Ph*COMe	3% Ph*COMe	76% Ph*COMe	76% Ph*COMe	84% Ph*COMe	8% Ph*COMe	43% Ph*COMe	19% Ph*COMe	74% Ph*COMe
HepOH	6% HepOH	60% HepOH	60% HepOH	77% HepOH	0% HepOH	53% HepOH	19% HepOH	63% HepOH

Supplementary Figure 18. Effect of head space gases in the optimised conditions at room temperature and 85 °C. Yield determined by quantitative ^{1}H NMR using 1,1,2,2-tetrachlorethane as internal standard. Isolated yield shown in parentheses. Ph*COMe and HepOH represent **1** and **2** and is depicted as such to allow facile interpretation of data.

Procedure: Experiments prepared according to **General Procedure A Modification I** with the following modifications. Reactions run under an air with an argon balloon were achieved as above but an argon balloon added after the vial was capped. Reactions run under an atmosphere of oxygen achieved by inserting an oxygen balloon into capped reaction vial containing reaction solids after evacuation; *tert*-amyl alcohol and heptanol were also sparged with oxygen for >15 min (*via* balloon) before their addition to the reaction vial. Reactions run under an atmosphere of nitrogen were prepared according to **General Procedure A Modification I.**

Deactivation of Catalyst Before Degassing Hydrogen Borrowing Reaction

Commentary: To investigate catalyst deactivation, the reaction was first run for 1.5 h under an air atmosphere at 23 °C, before being heated to relevant temperature for a further 16.5 h (**Supplementary Figure 19**).

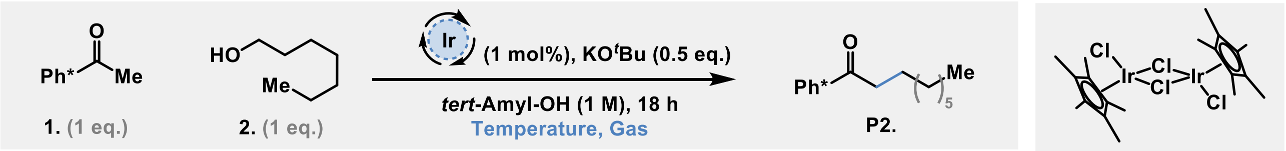

	23 °C			85 °C			115 °C	
**Entry 1**	**Entry 2**	**Entry 3**	**Entry 4**	**Entry 5**	**Entry 6**	**Entry 7**	**Entry 8**	**Entry 9**
N_2	Air, 1.5 h, 23 °C *then* degas *then* 16.5 h, 23 °C	Air	N_2	Air, 1.5 h, 23 °C *then* degas *then* 16.5 h, 85 °C	Air	N_2	Air, 1.5 h, 23 °C *then* degas *then* 16.5 h, 115 °C	Air
91% (90%)	1%	3%	88%	34%	21%	87%	52%	63%
3% Ph*COMe	79% Ph*COMe	76% Ph*COMe	8% Ph*COMe	30% Ph*COMe	43% Ph*COMe	8% Ph*COMe	29% Ph*COMe	9% Ph*COMe
6% HepOH	78% HepOH	60% HepOH	0% HepOH	38% HepOH	53% HepOH	0% HepOH	16% HepOH	10% HepOH

Supplementary Figure 19. Interrogating catalyst deactivation. Yield determined by quantitative ^{1}H NMR using 1,1,2,2-tetrachlorethane as internal standard. Yield in parentheses is of isolated material.

At room temperature and 85 °C the length of 'deactivation' was shortened to 0.5 h (**Supplementary Figure 20**). Comparing this data to entries run under a nitrogen atmosphere with 0.5 mol% and 0.25 mol% catalyst (**Entries 1, 3** and **4, 6**) allowed suggestion of the amount of catalyst that had been deactivated. After 0.5 h under air there was likely 0.25–0.5 mol% active dimeric catalyst available in the reaction mixture and therefore 0.5–0.75 mol% had been consumed or deactivated after just 0.5 h.

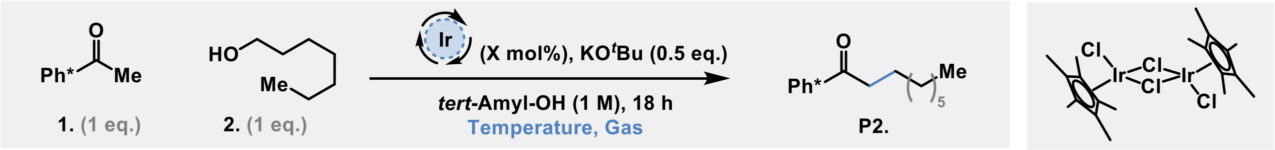

	23 °C			85 °C	
**Entry 1**	**Entry 2**	**Entry 3**	**Entry 4**	**Entry 5**	**Entry 6**
0.5 mol% Ir	1 mol% Ir Air, 0.5 h, 23 °C *then* degas *then* 17.5 h, 23 °C	0.25 mol% Ir	0.5 mol% Ir	1 mol% Ir Air, 0.5 h, 23 °C *then* degas *then* 17.5 h, 85 °C	0.25 mol% Ir
67%	57%	13%	85%	52%	12%
28% Ph*COMe	27% Ph*COMe	84% Ph*COMe	10% Ph*COMe	24% Ph*COMe	85% Ph*COMe
10% HepOH	8% HepOH	67% HepOH	1% HepOH	12% HepOH	79% HepOH

Supplementary Figure 20. Interrogating catalyst deactivation. Yield determined by quantitative ^{1}H NMR using 1,1,2,2-tetrachlorethane as internal standard.

Procedure: Experiments prepared with a modification to **General Procedure A Modification I**. Reactions were set up under air and the reaction mixture was allowed to stir at 23 ˚C for 0.5 h or 1.5 h. The vial was attached to a Schlenk line and evacuated and backfilled with nitrogen (6 × 10 sec). The reaction mixture was then stirred at 23 ˚C for 17.5 or 16.5 h respectively. The reaction mixture was filtered through a short pad of silica gel (elution with diethyl ether) and concentrated *in vacuo.* The resulting oil was redissolved in $CDCl_3$ (2.0 mL) and an NMR standard was added (32 μL 0.3 mmol, 1,1,2,2-tetrachloroethane) to determine yield *via* ^{1}H NMR spectroscopy.

Reaction without Catalyst

Commentary: The reaction was run without catalyst at relevant temperatures under nitrogen and air (**Supplementary Figure 21**). In all cases no product was observed.

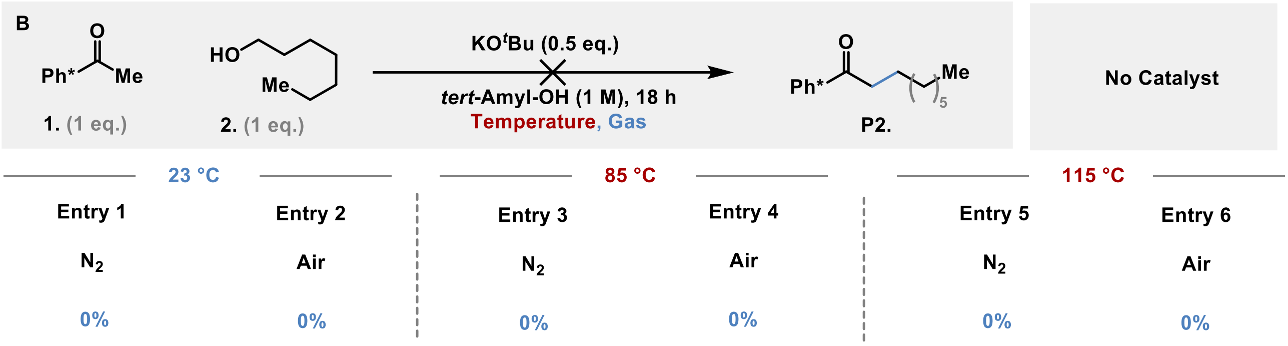

Supplementary Figure 21. Reaction without a catalyst. Yield determined by quantitative ^{1}H NMR using 1,1,2,2-tetrachlorethane as internal standard.

N.b. Screening of other catalysts in 'Discovery and Optimization of Reaction at 23 °C'.

NMR Spectra

1: **^{1}H NMR** (400 MHz, $CDCl_3$) and **^{13}C NMR** (101 MHz, $CDCl_3$) respectively.

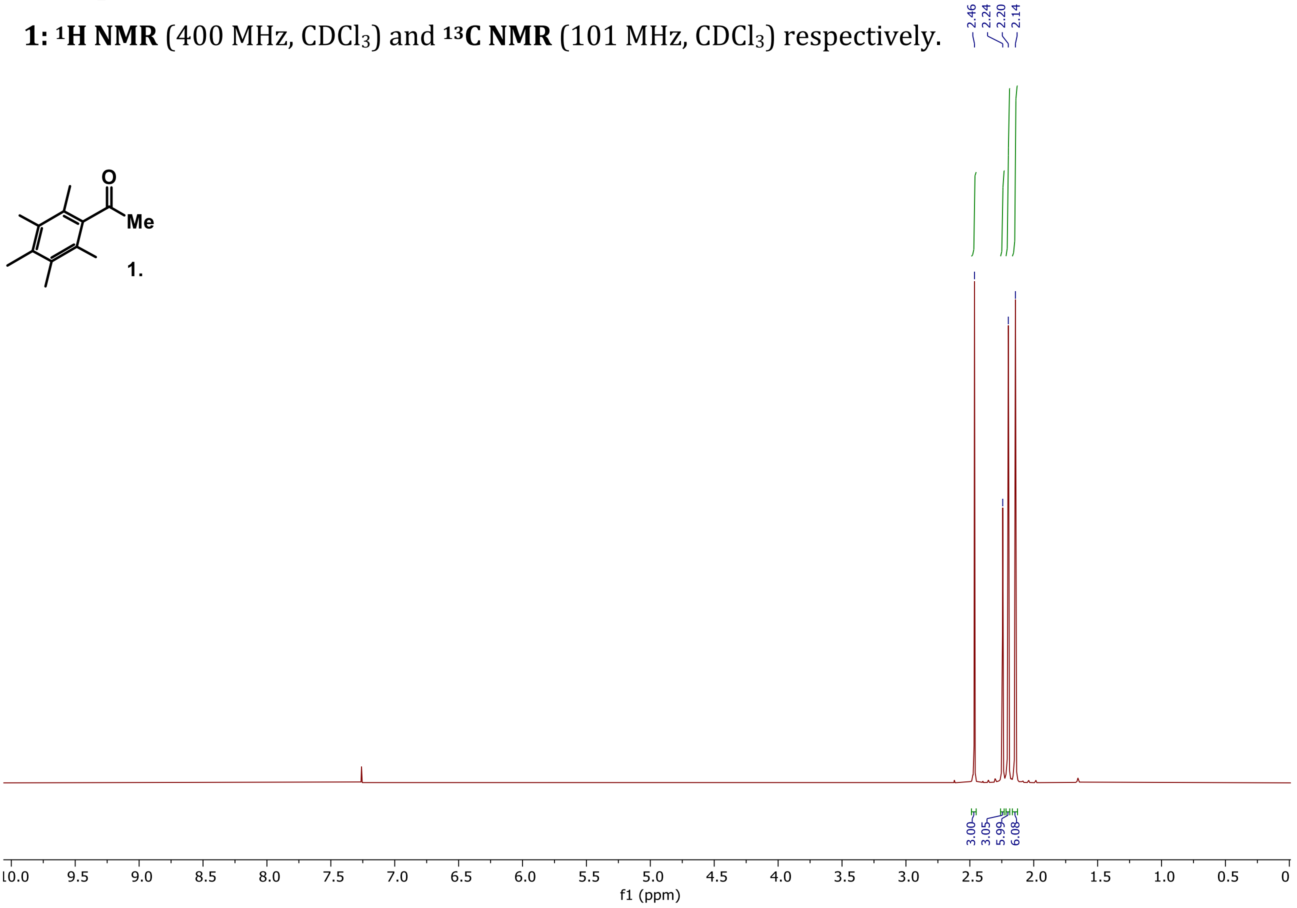

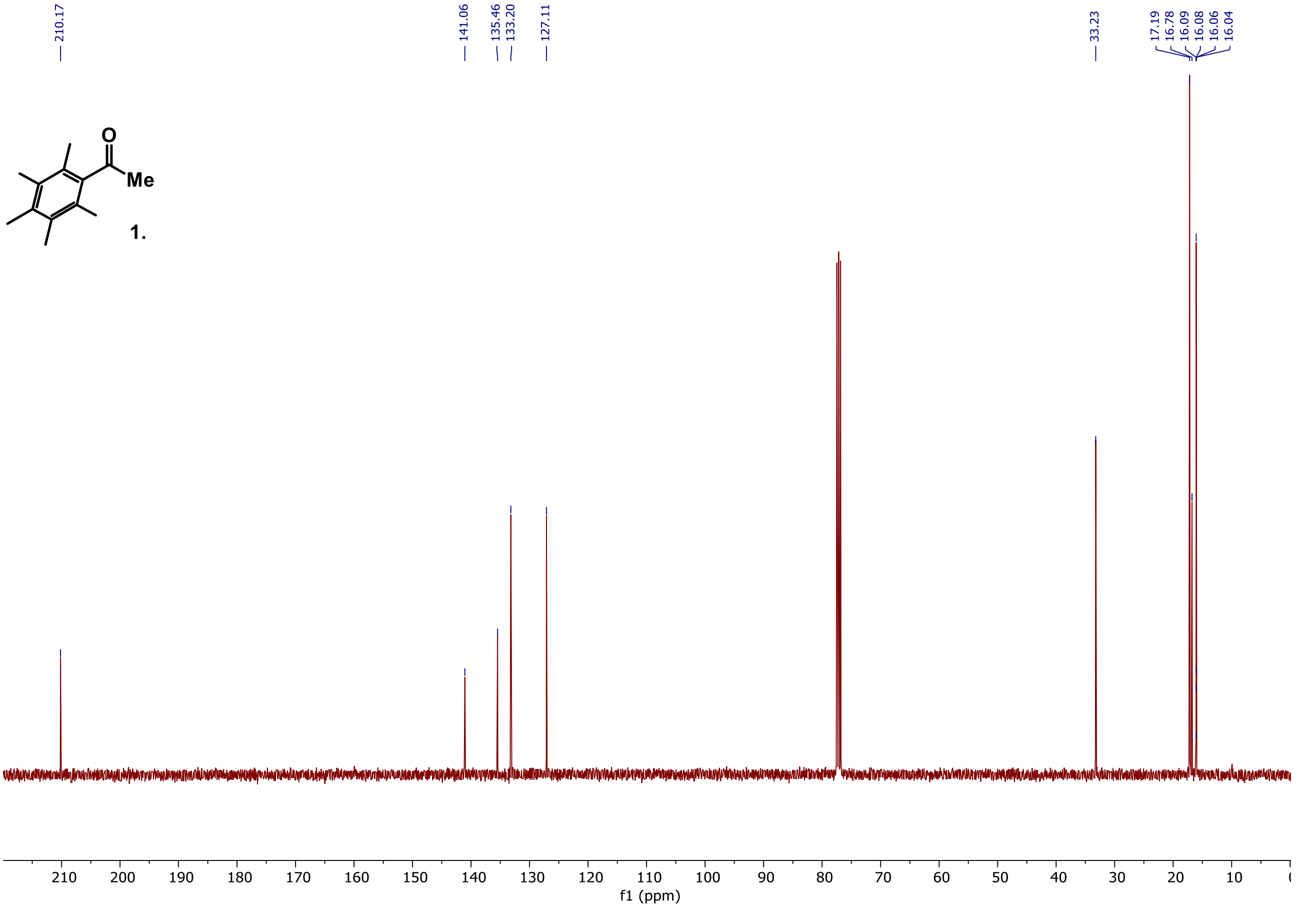

S2: ^{1}**H NMR** (400 MHz, $CDCl_3$) and ^{13}**C NMR** (101 MHz, $CDCl_3$) respectively.

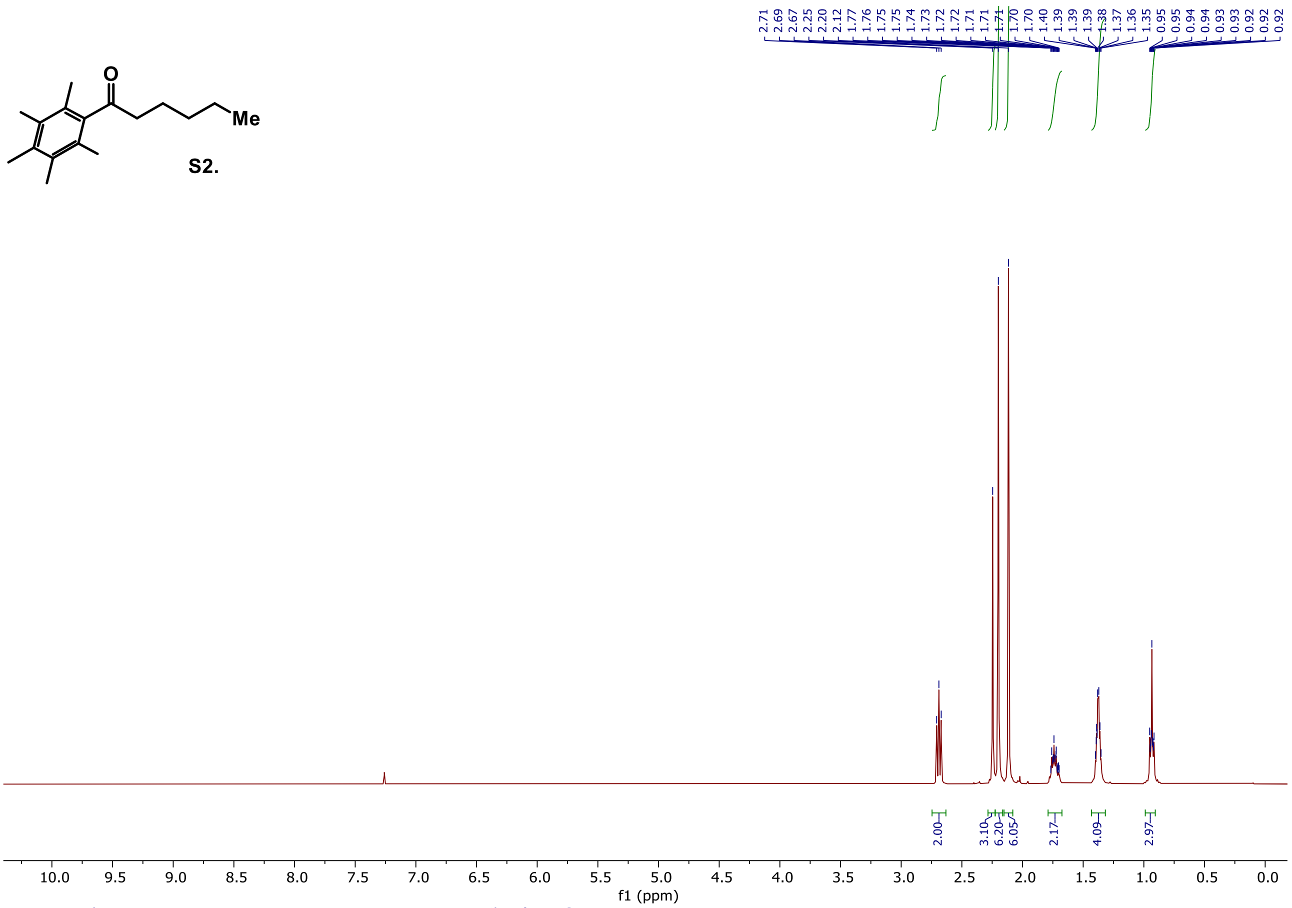

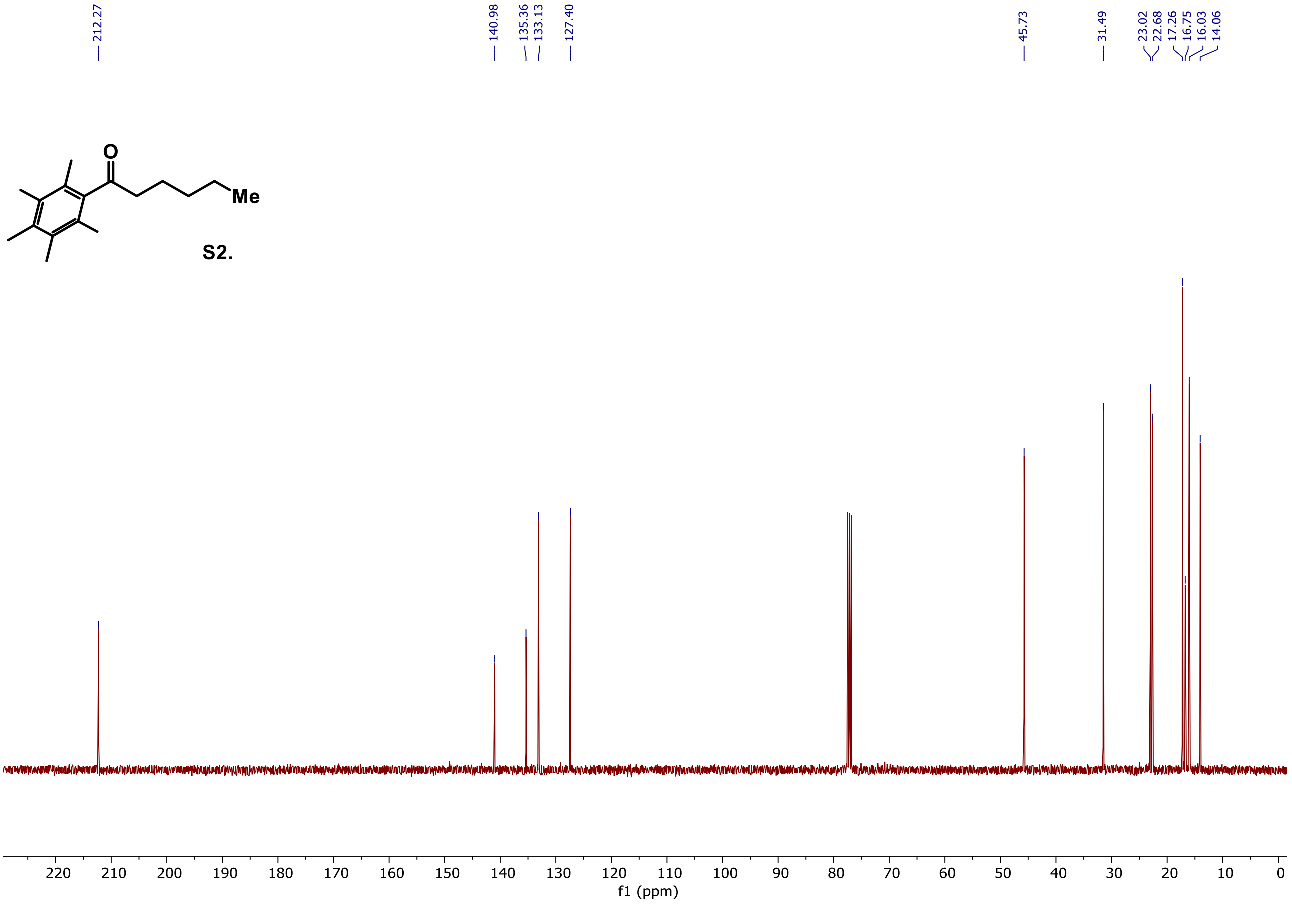

P2 (or 94): ^{1}**H NMR** (400 MHz, $CDCl_3$) and ^{13}**C NMR** (101 MHz, $CDCl_3$) respectively.

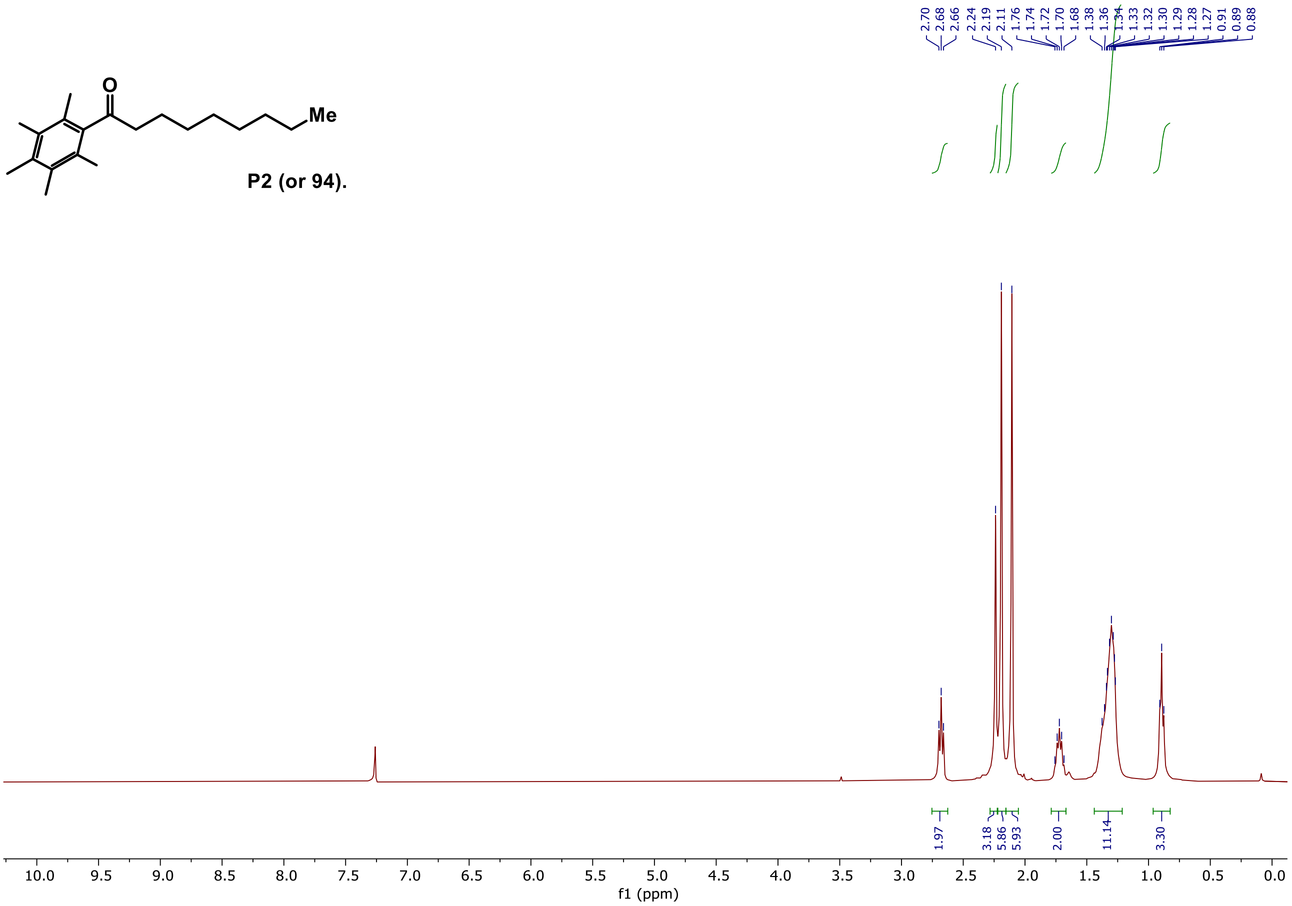

P2 (or 94).

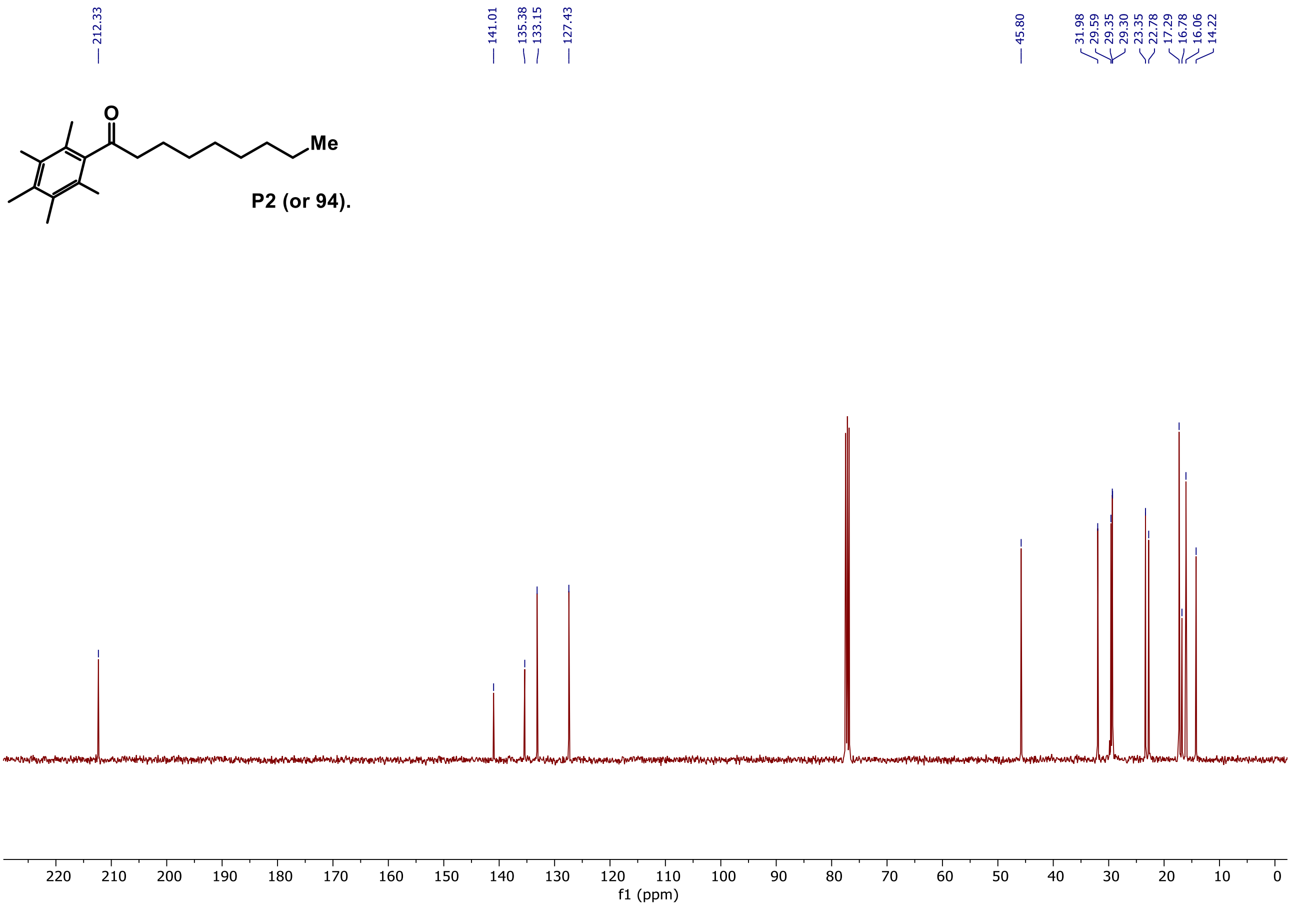

P2 (or 94).

P3: ¹H NMR (400 MHz, $CDCl_3$) and **¹³C NMR** (101 MHz, $CDCl_3$) respectively.

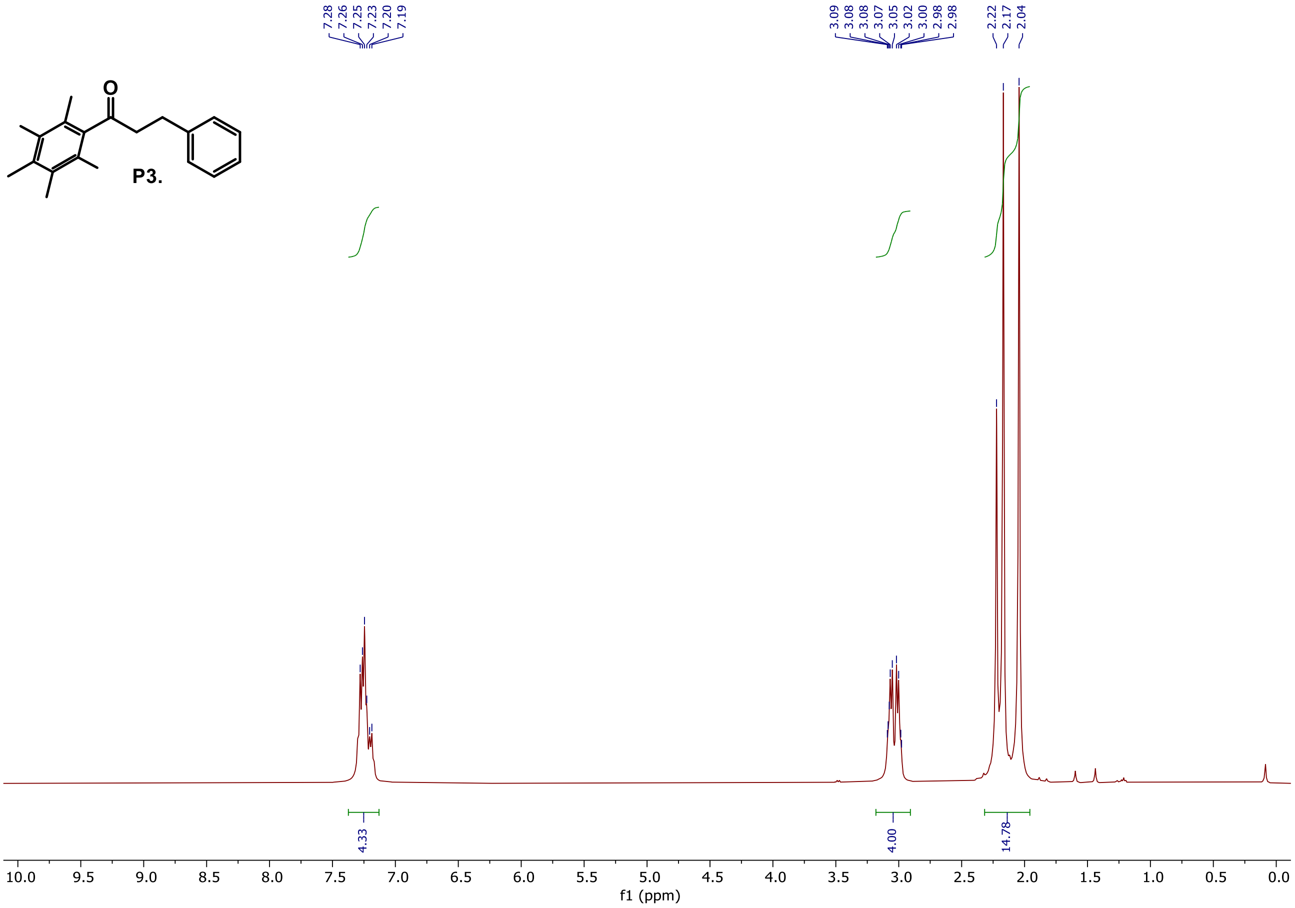

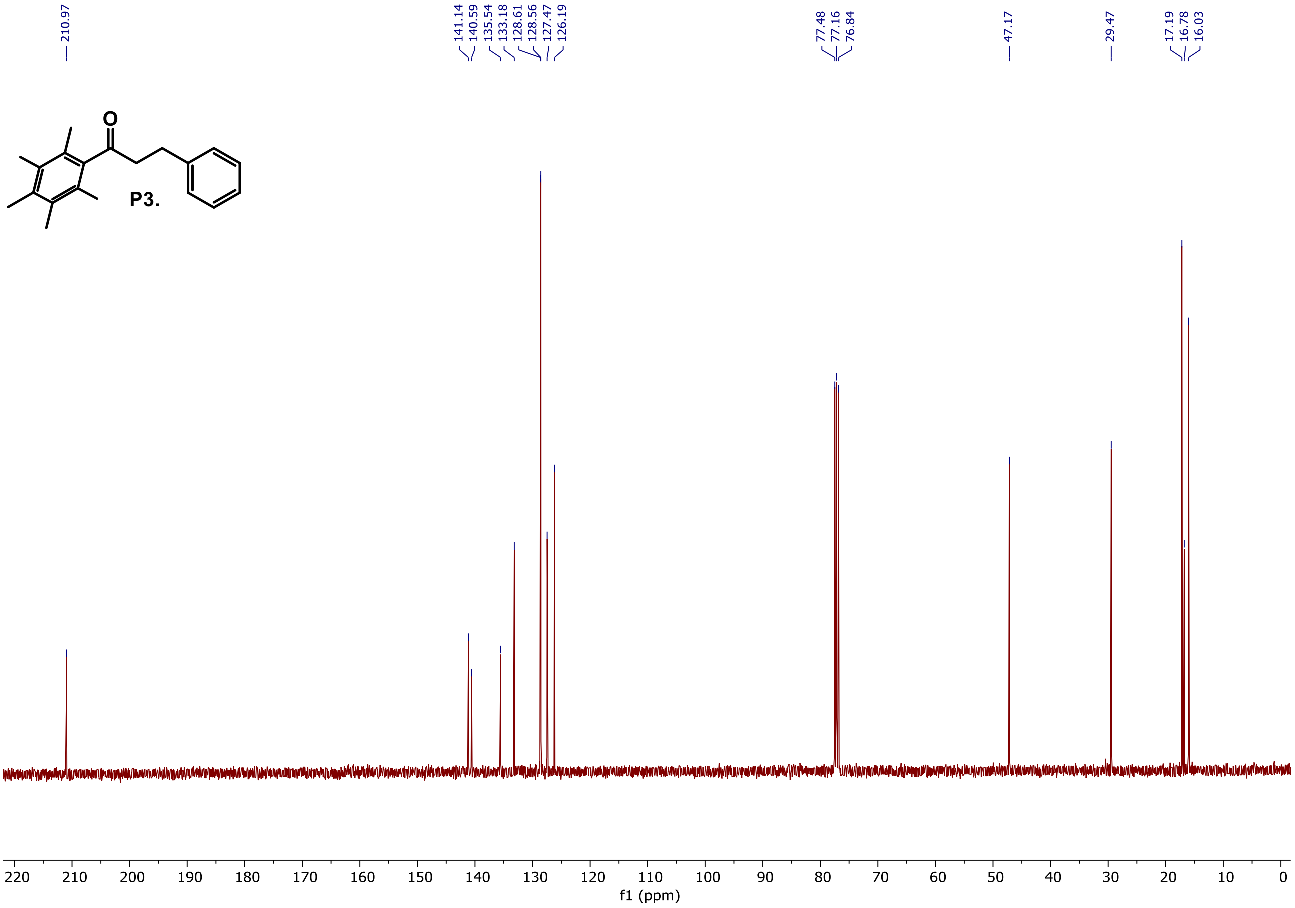

P4: ¹H NMR (400 MHz, $CDCl_3$) and **¹³C NMR** (101 MHz, $CDCl_3$) respectively.

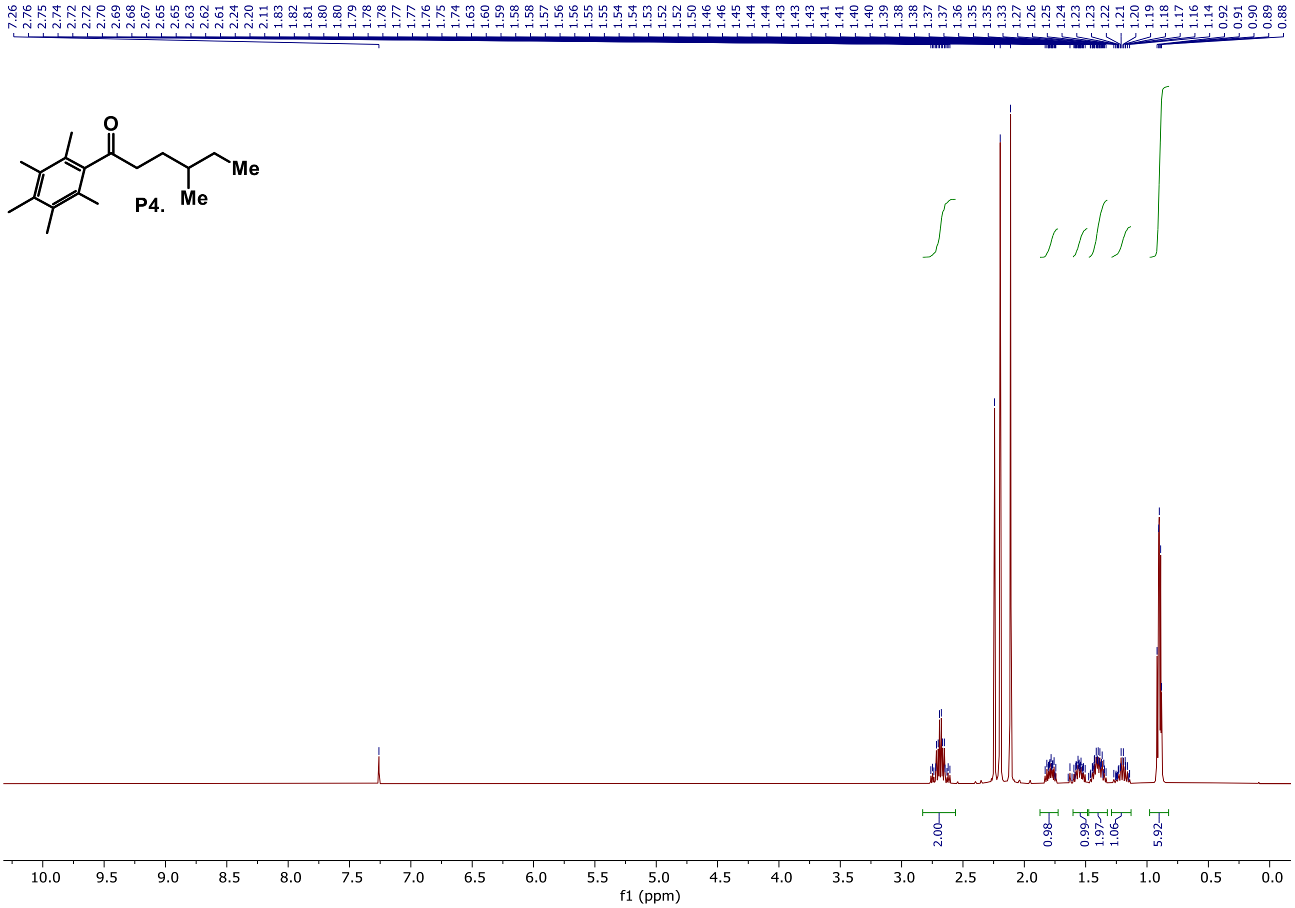

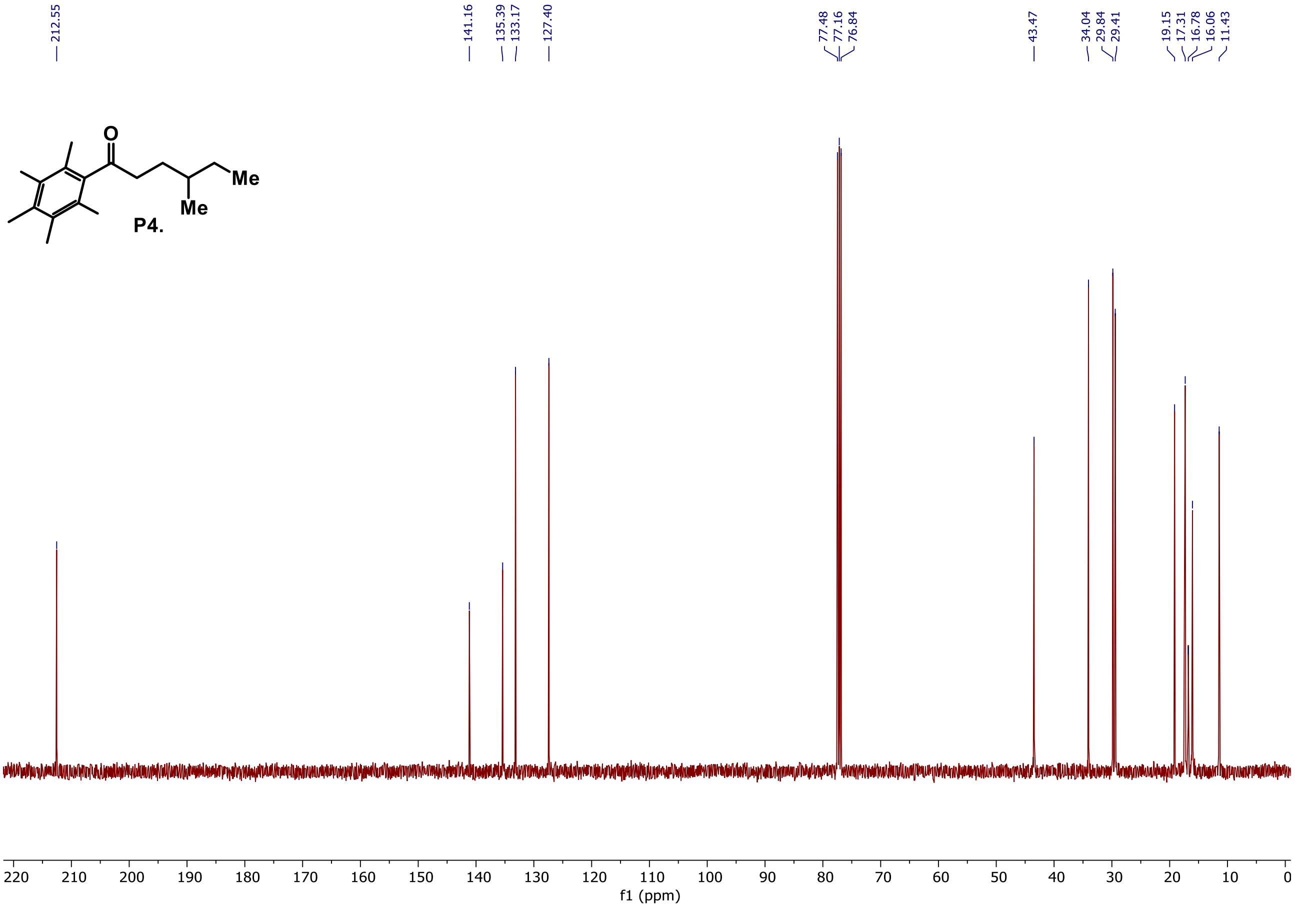

P5: ^{1}H NMR (400 MHz, $CDCl_3$) and **^{13}C NMR** (101 MHz, $CDCl_3$) respectively.

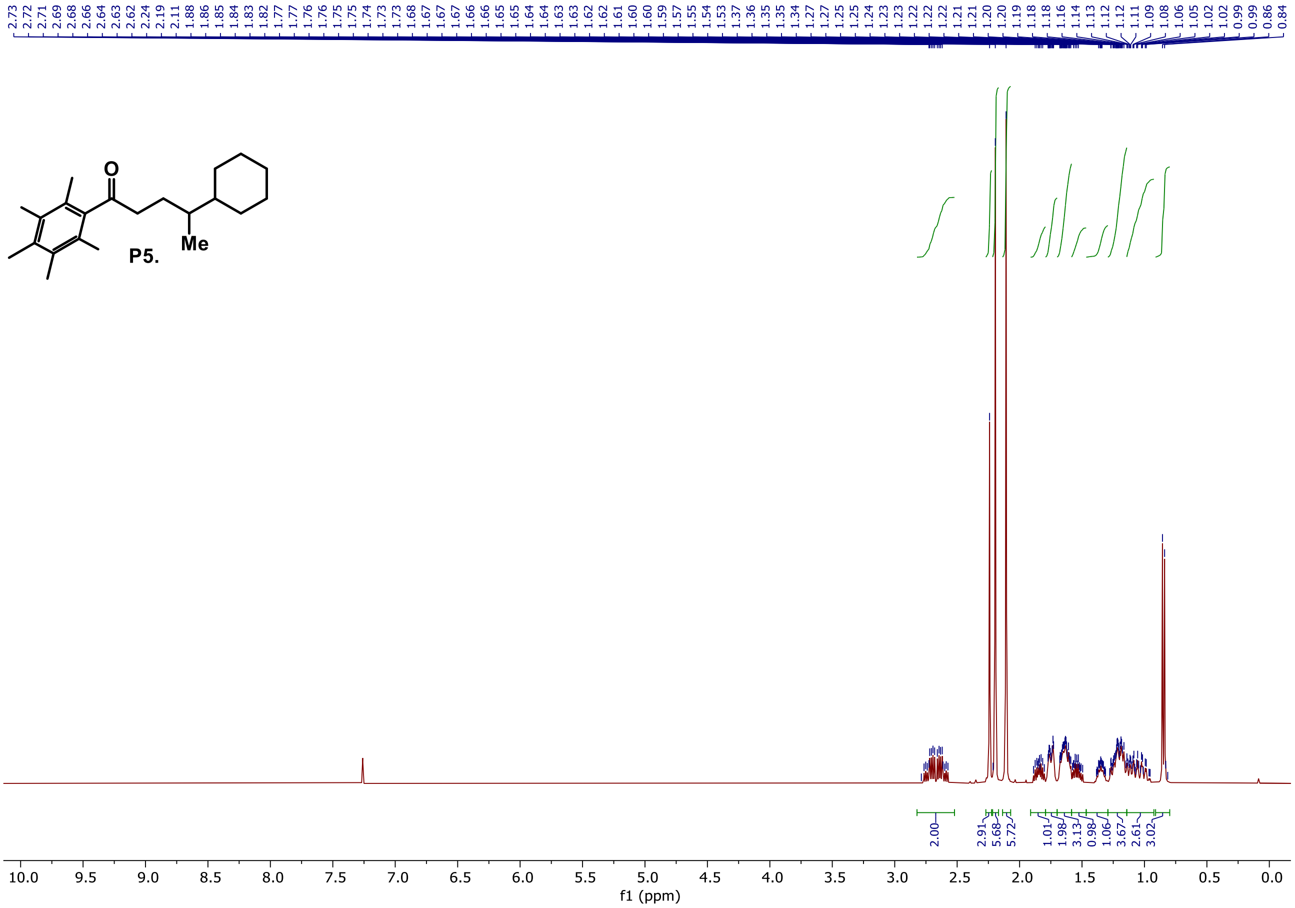

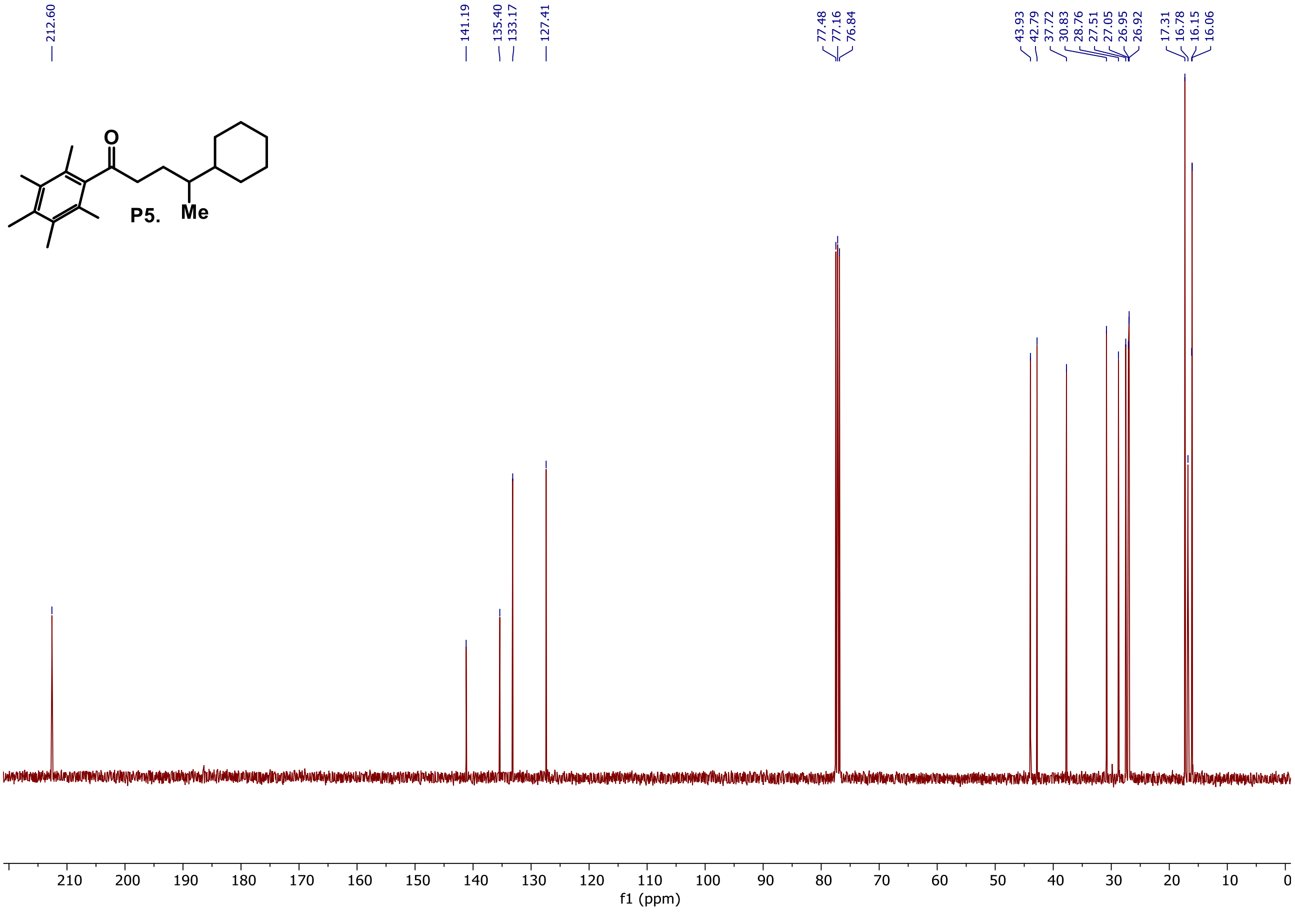

P6: ^{1}H NMR (400 MHz, $CDCl_3$) and **^{13}C NMR** (101 MHz, $CDCl_3$) respectively.

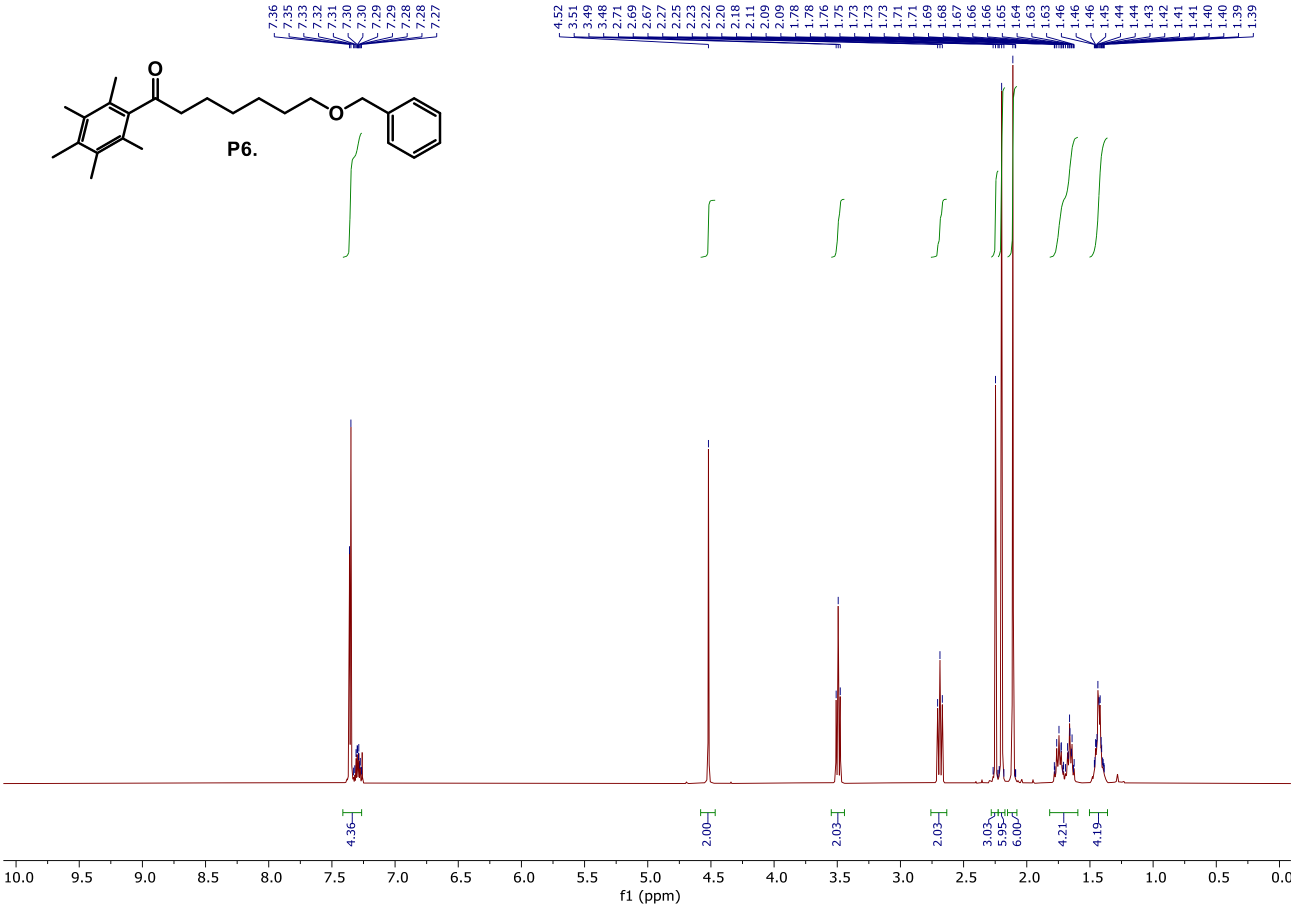

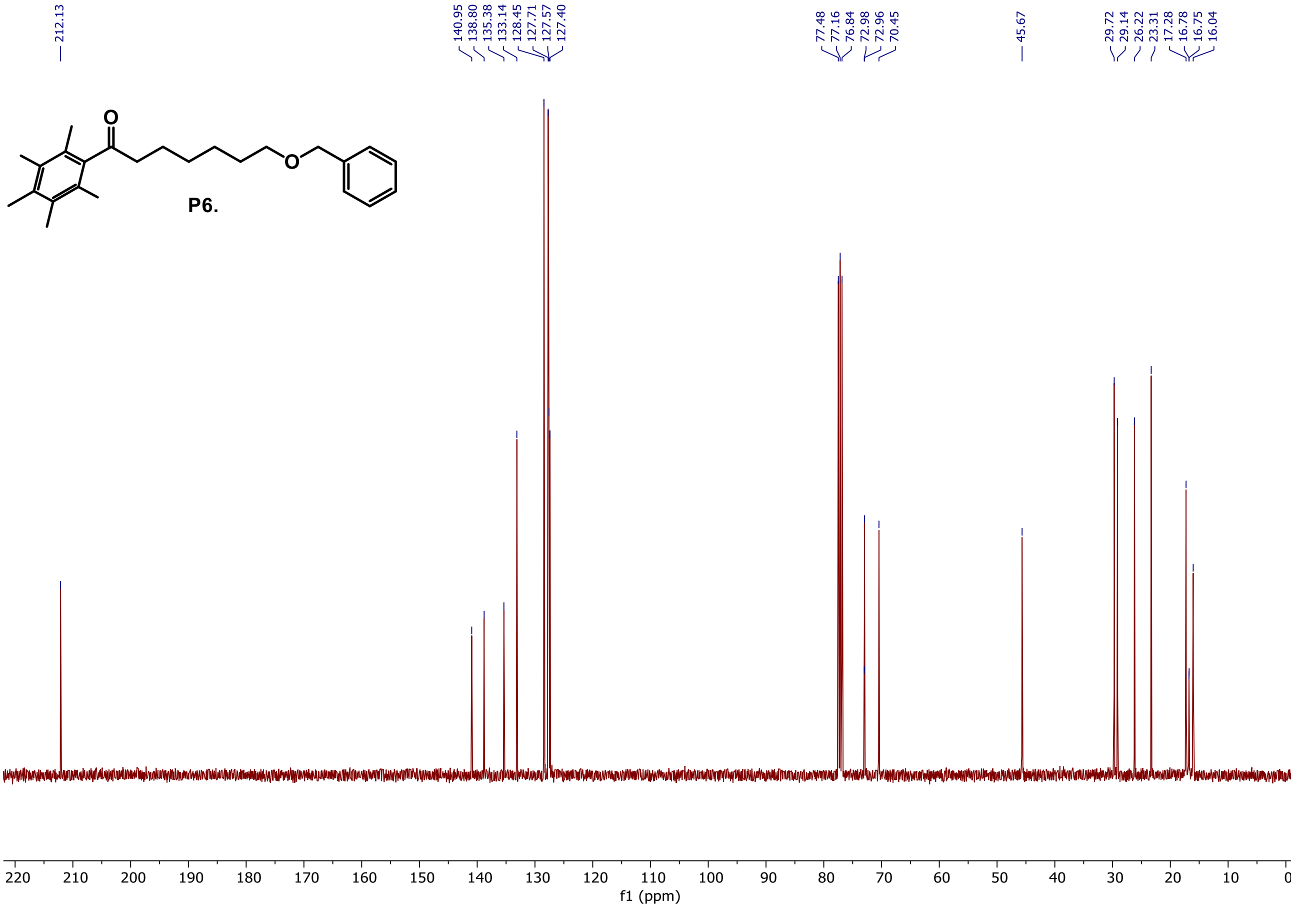

7: ^{1}H NMR (400 MHz, $CDCl_3$) and **^{13}C NMR** (101 MHz, $CDCl_3$) respectively.

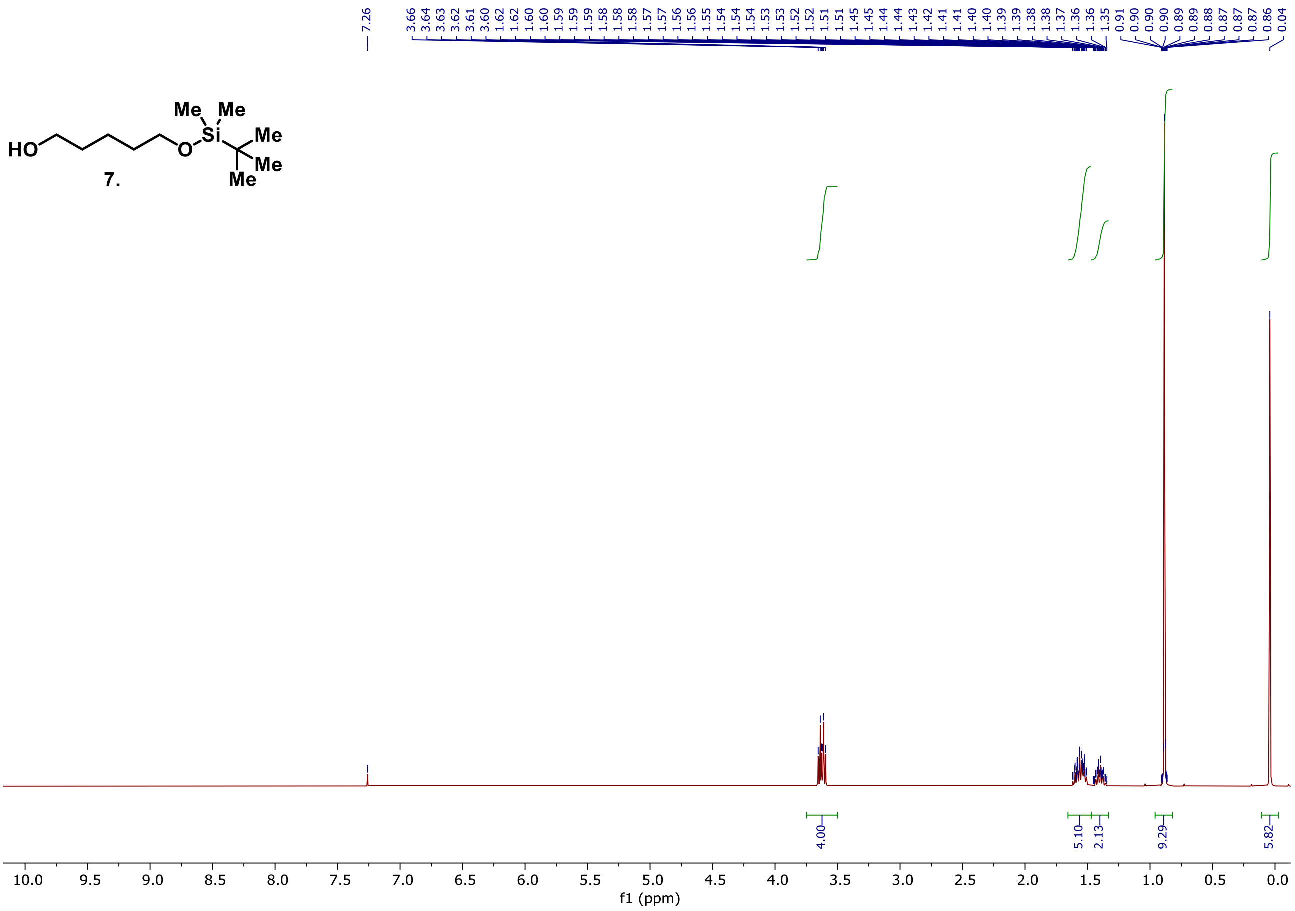

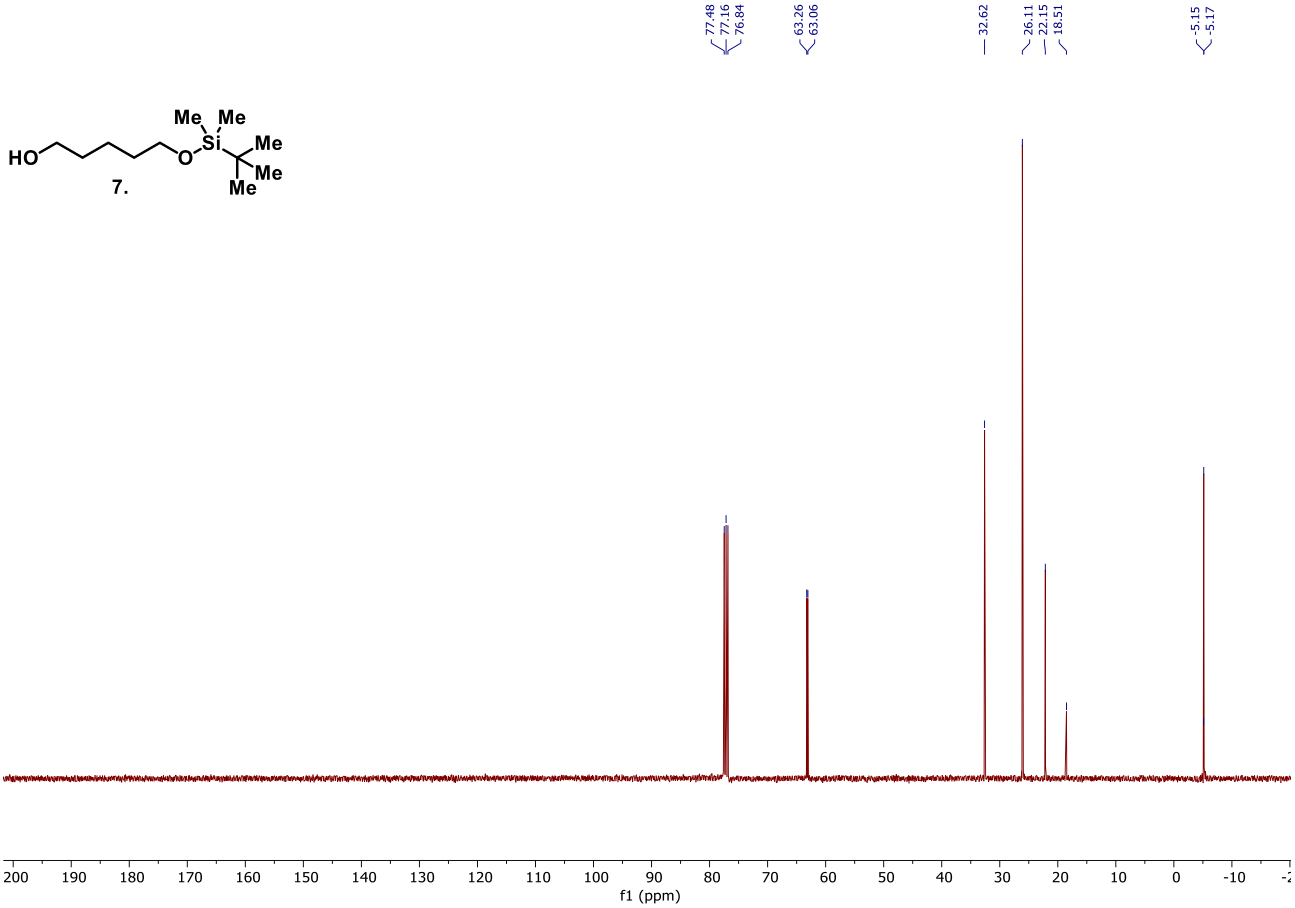

P7: ¹H NMR (400 MHz, $CDCl_3$) and **¹³C NMR** (101 MHz, $CDCl_3$) respectively.

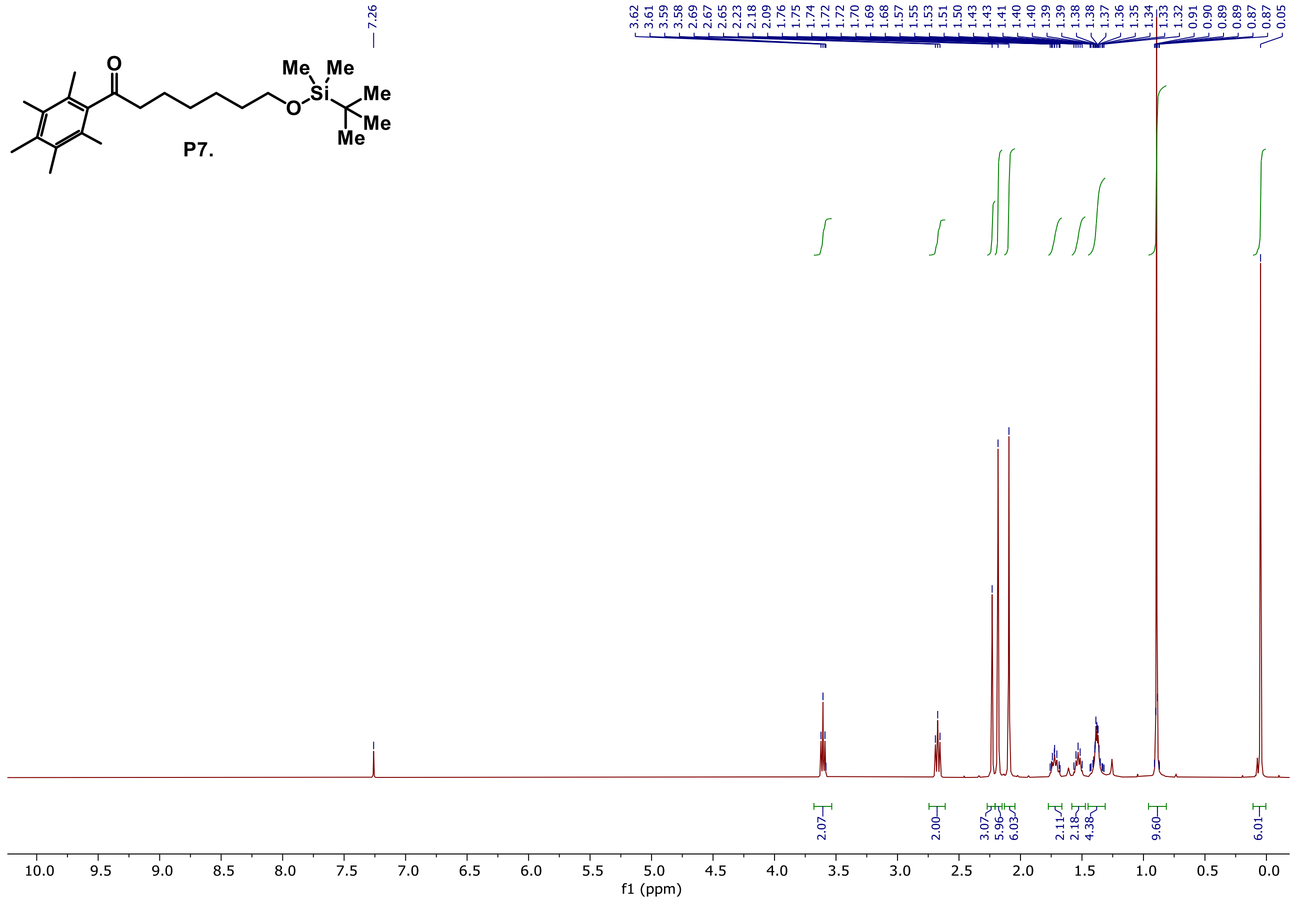

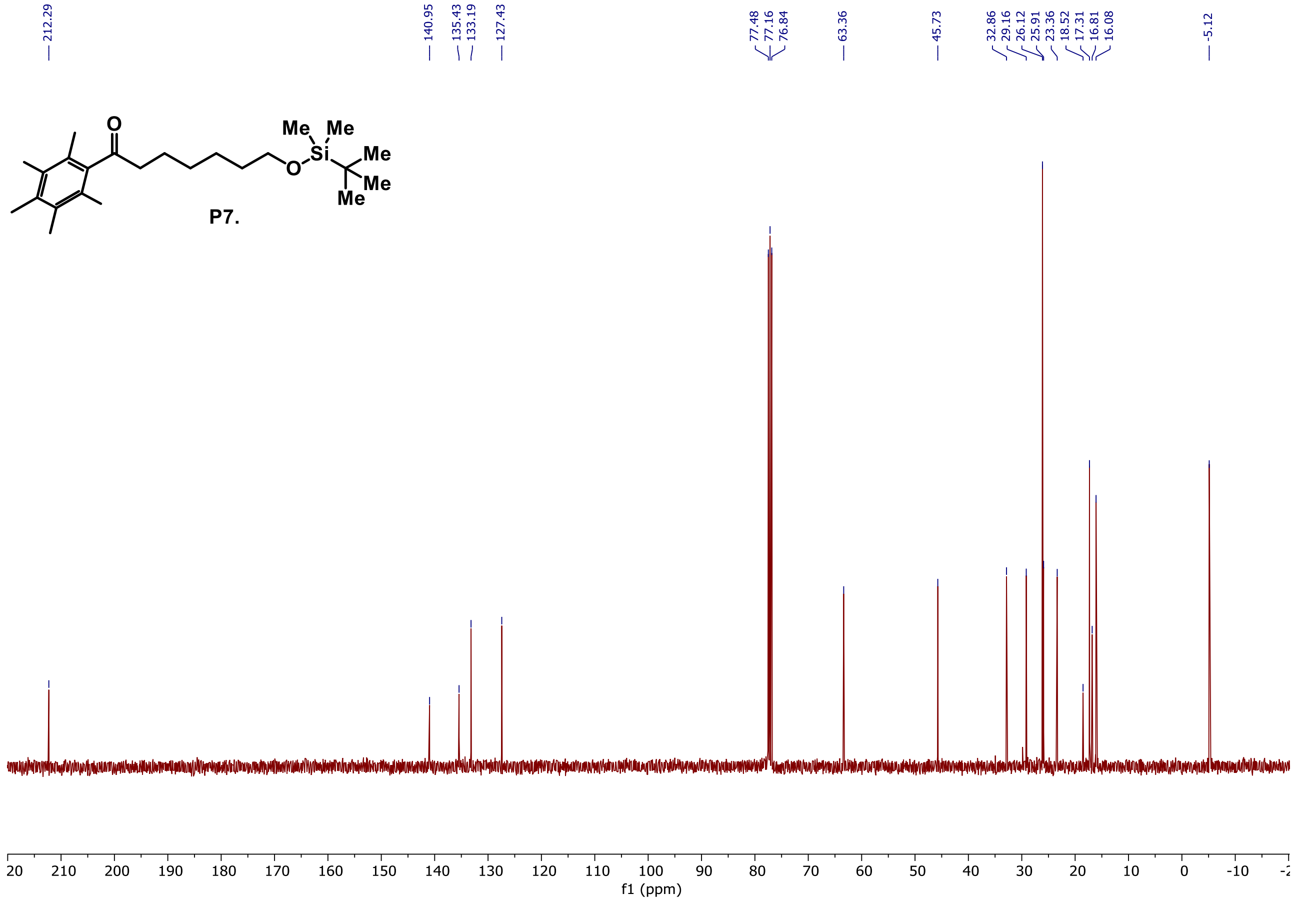

P8: ^{1}H NMR (600 MHz, $CDCl_3$) and **^{13}C NMR** (150 MHz, $CDCl_3$) respectively.

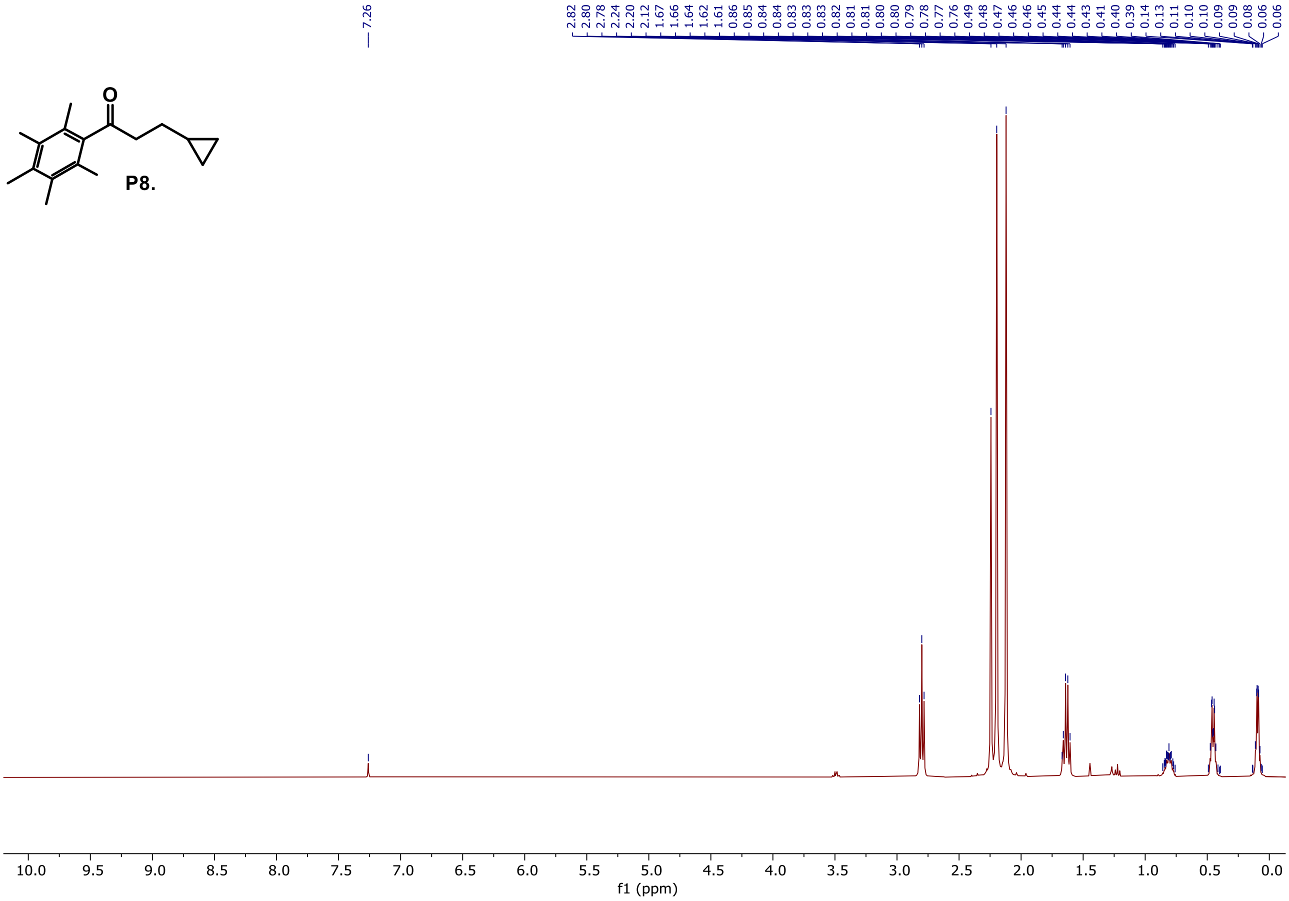

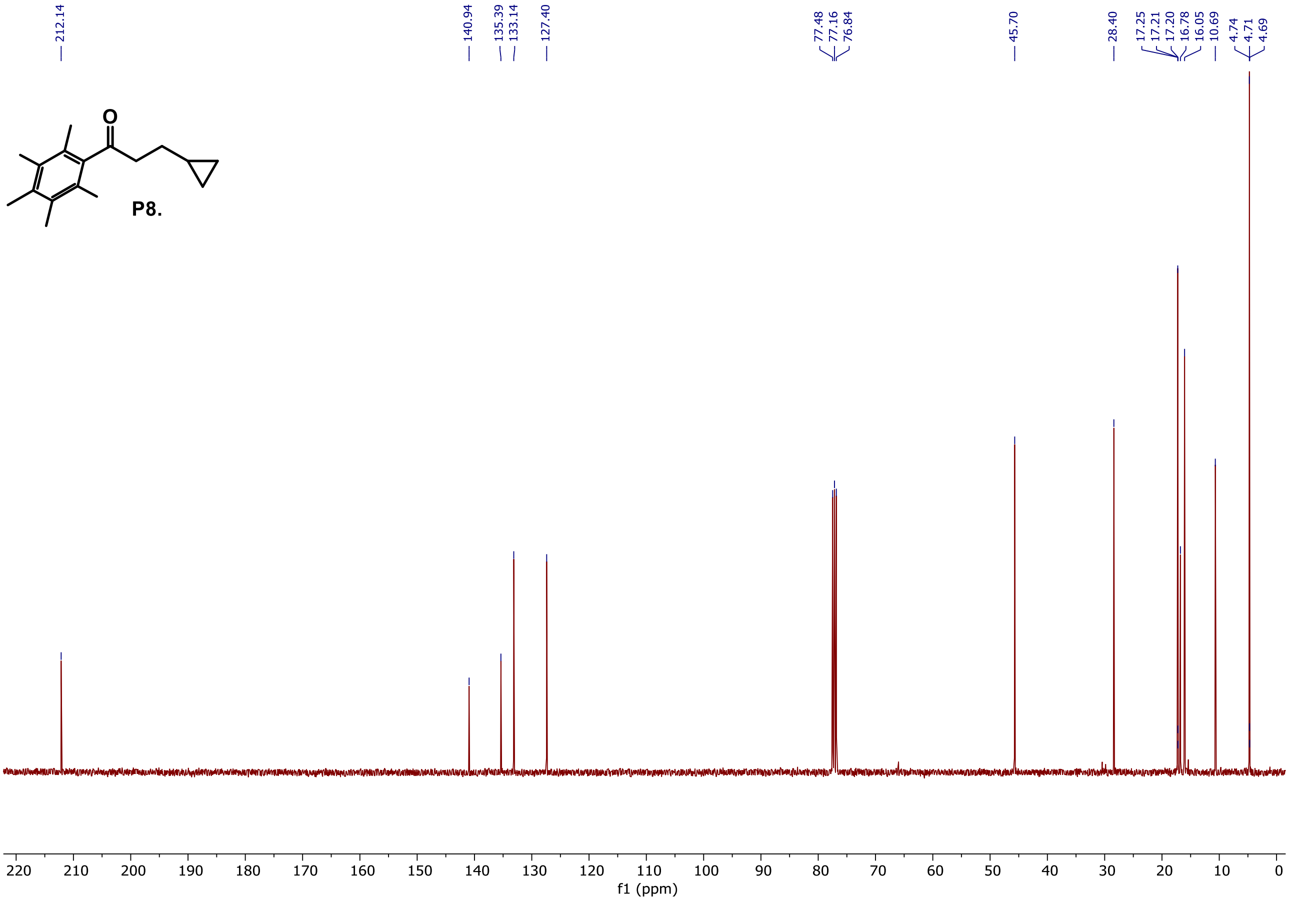

S8: **^{1}H NMR** (400 MHz, $CDCl_3$) and **^{13}C NMR** (101 MHz, $CDCl_3$) respectively.

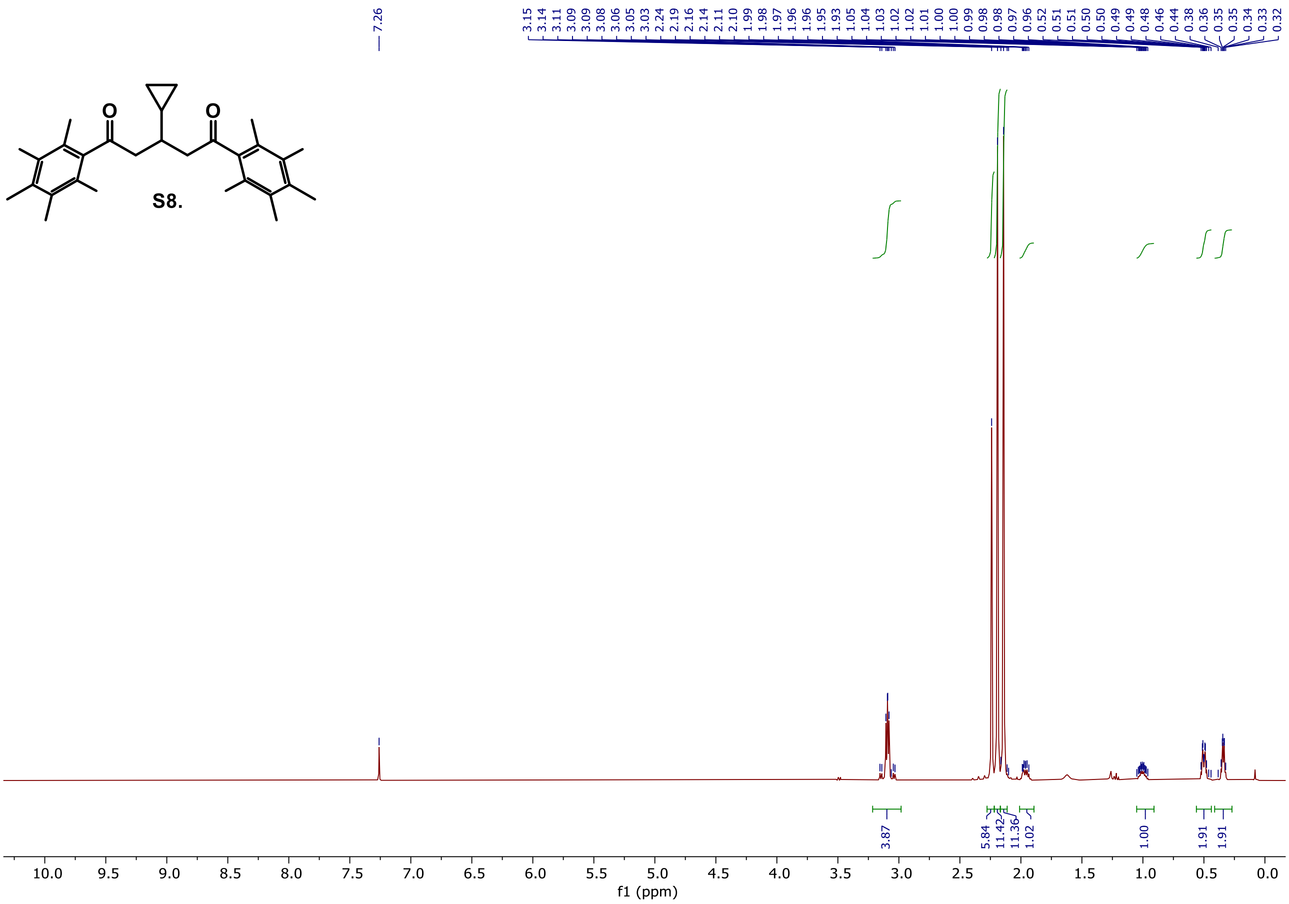

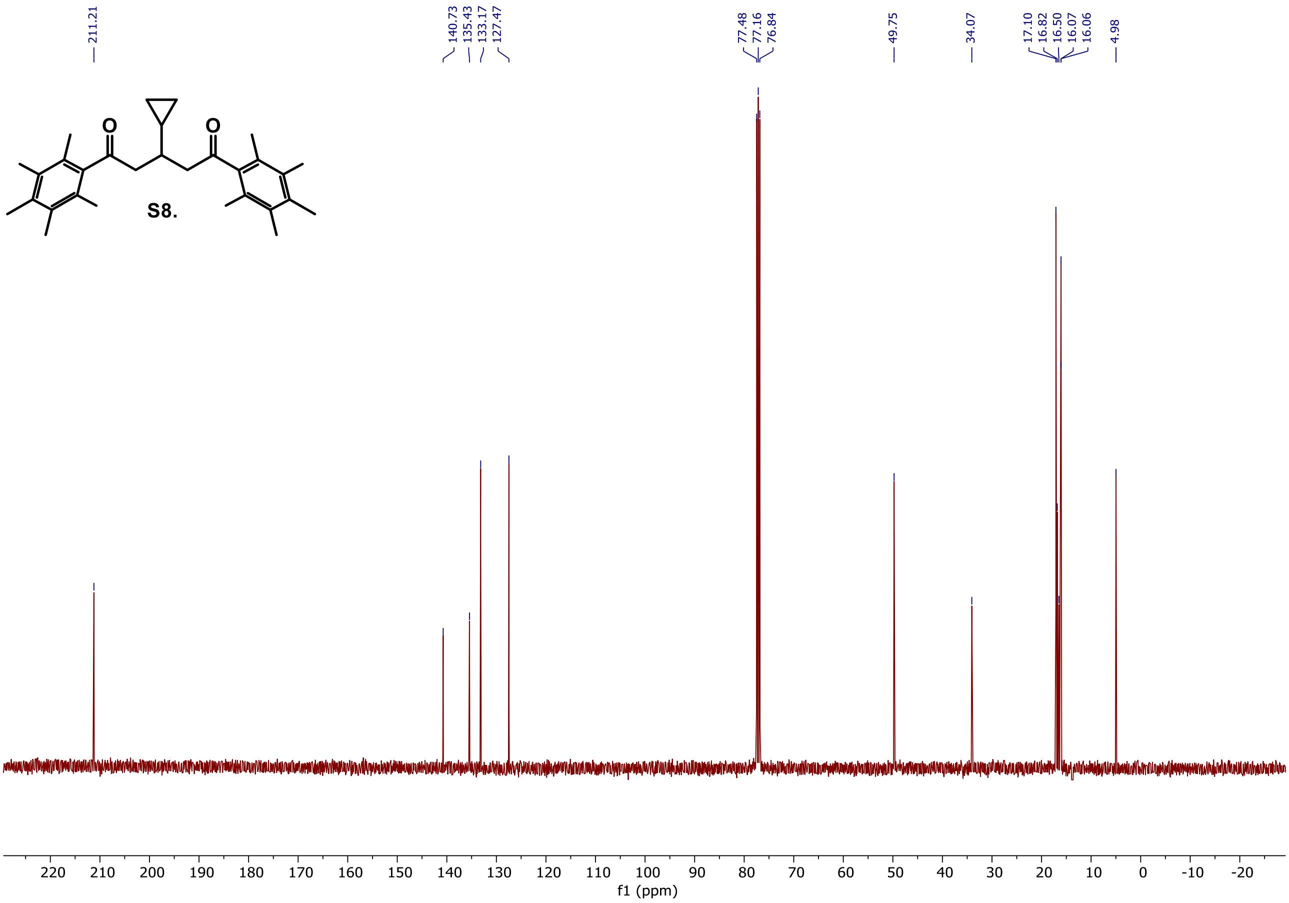

P9: ^{1}H NMR (400 MHz, $CDCl_3$) and **^{13}C NMR** (101 MHz, $CDCl_3$) respectively.

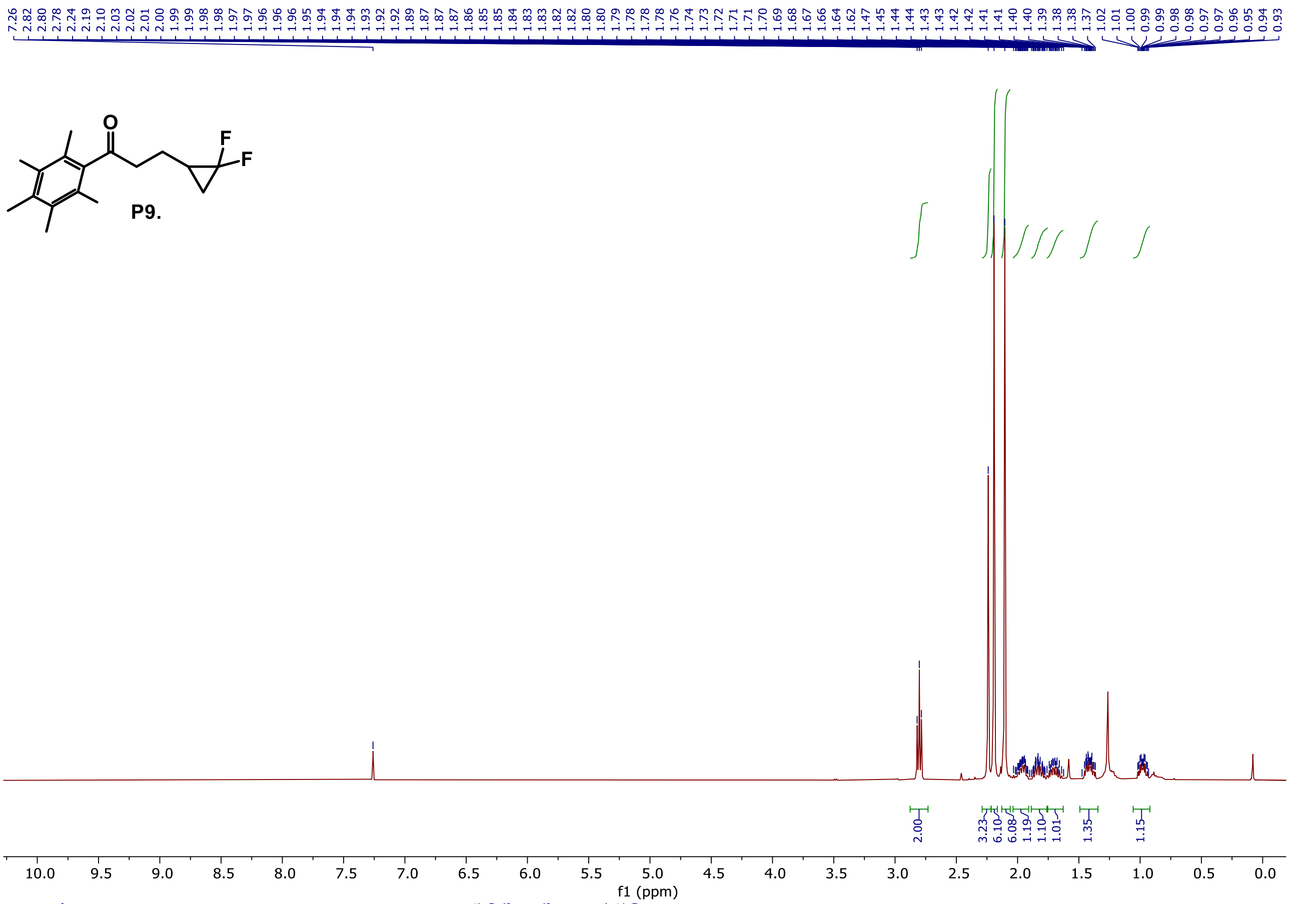

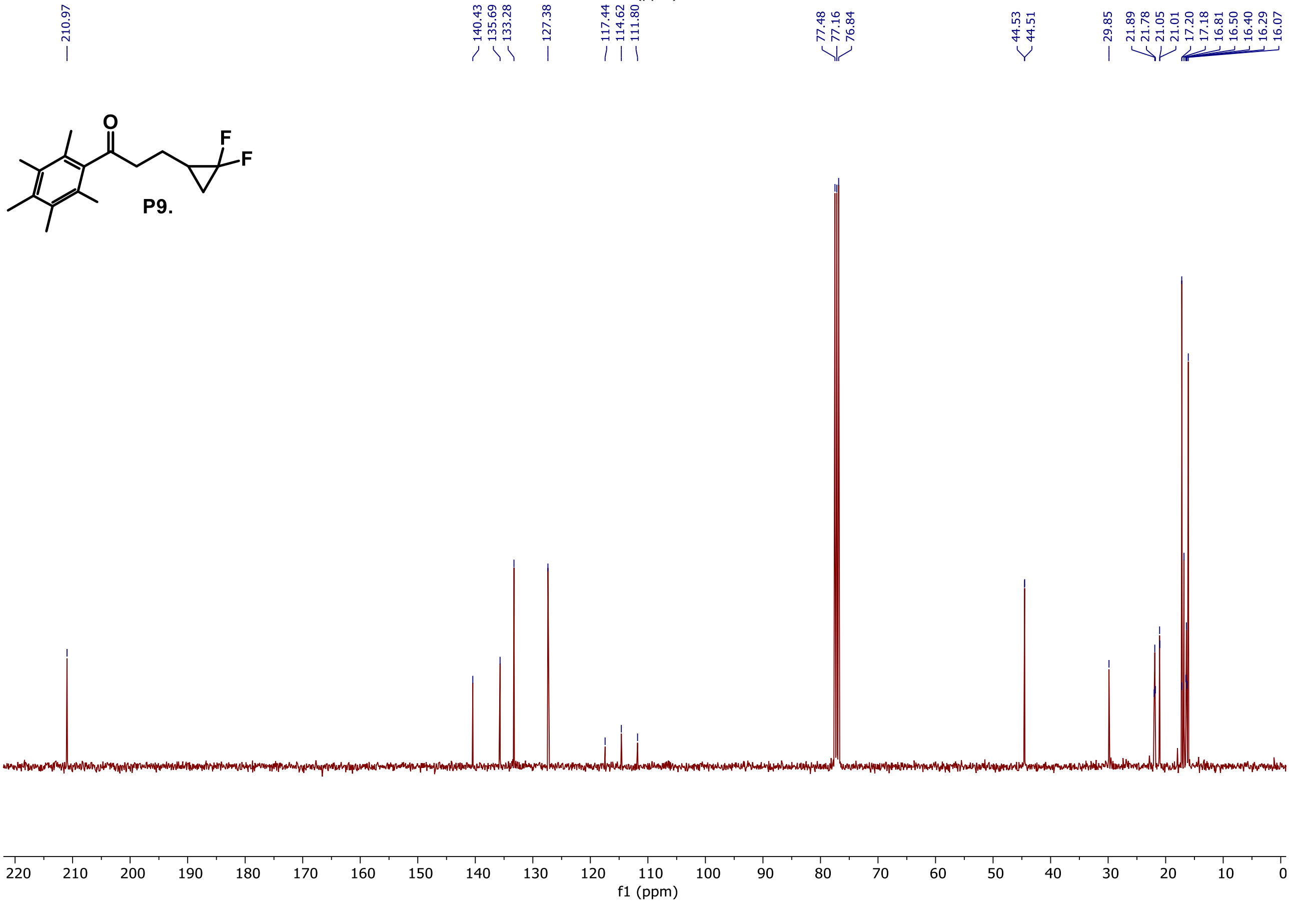

P9: ^{19}F NMR (376 MHz, $CDCl_3$).

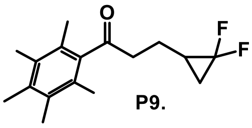

P9.

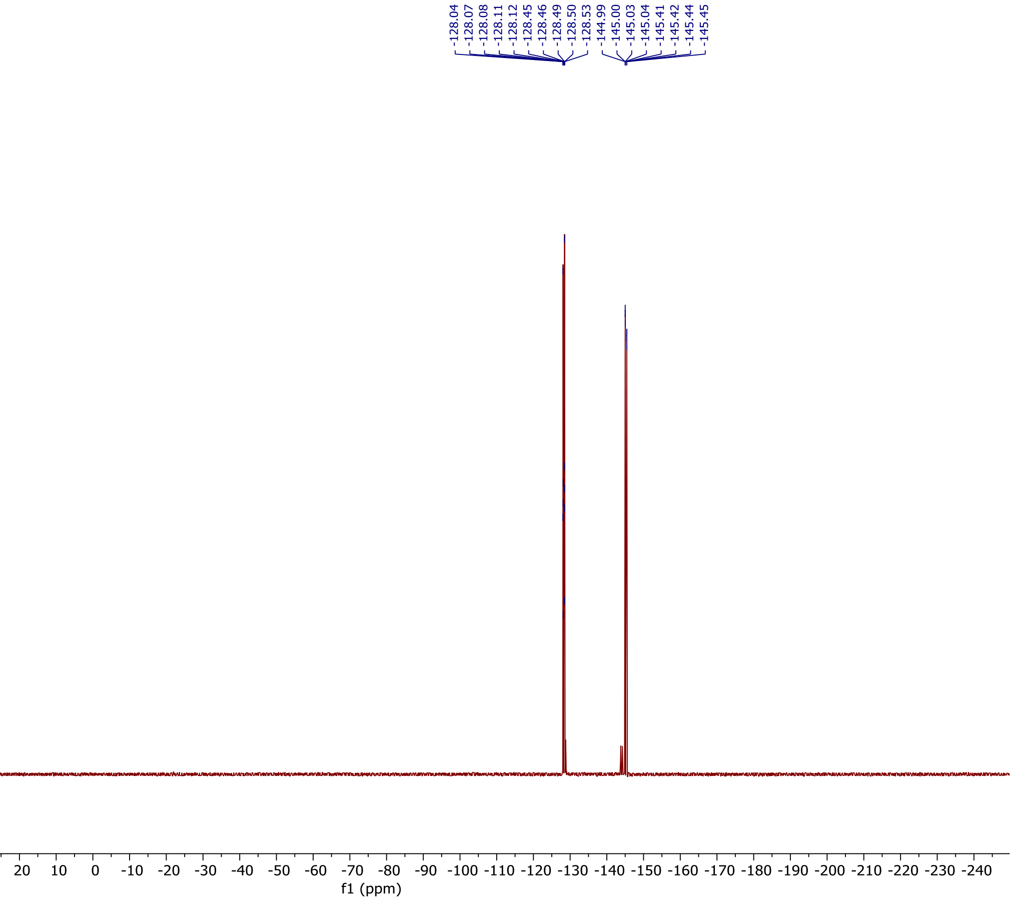

P10: ^{1}**H NMR** (400 MHz, $CDCl_3$) and ^{13}**C NMR** (101 MHz, $CDCl_3$) respectively.

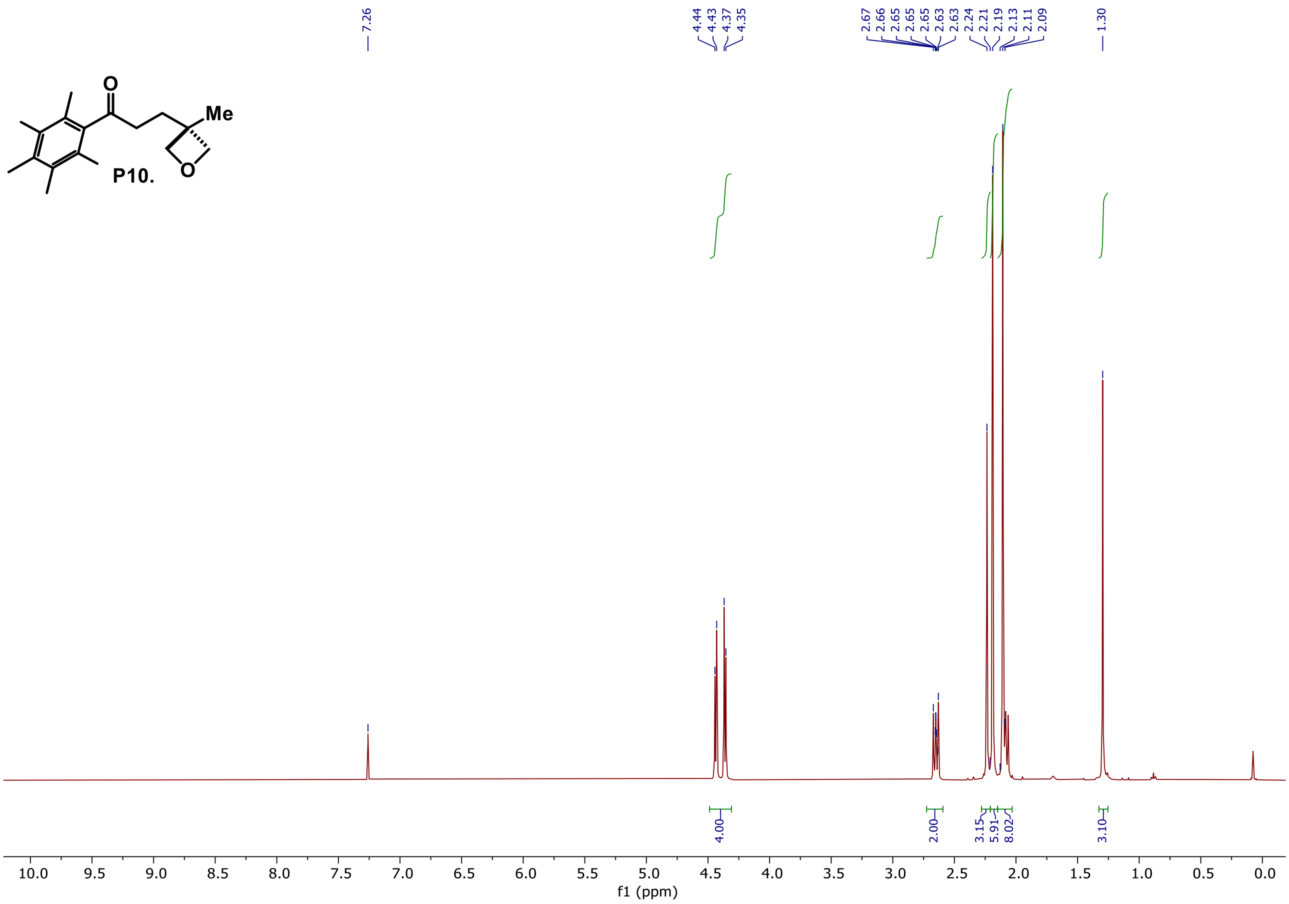

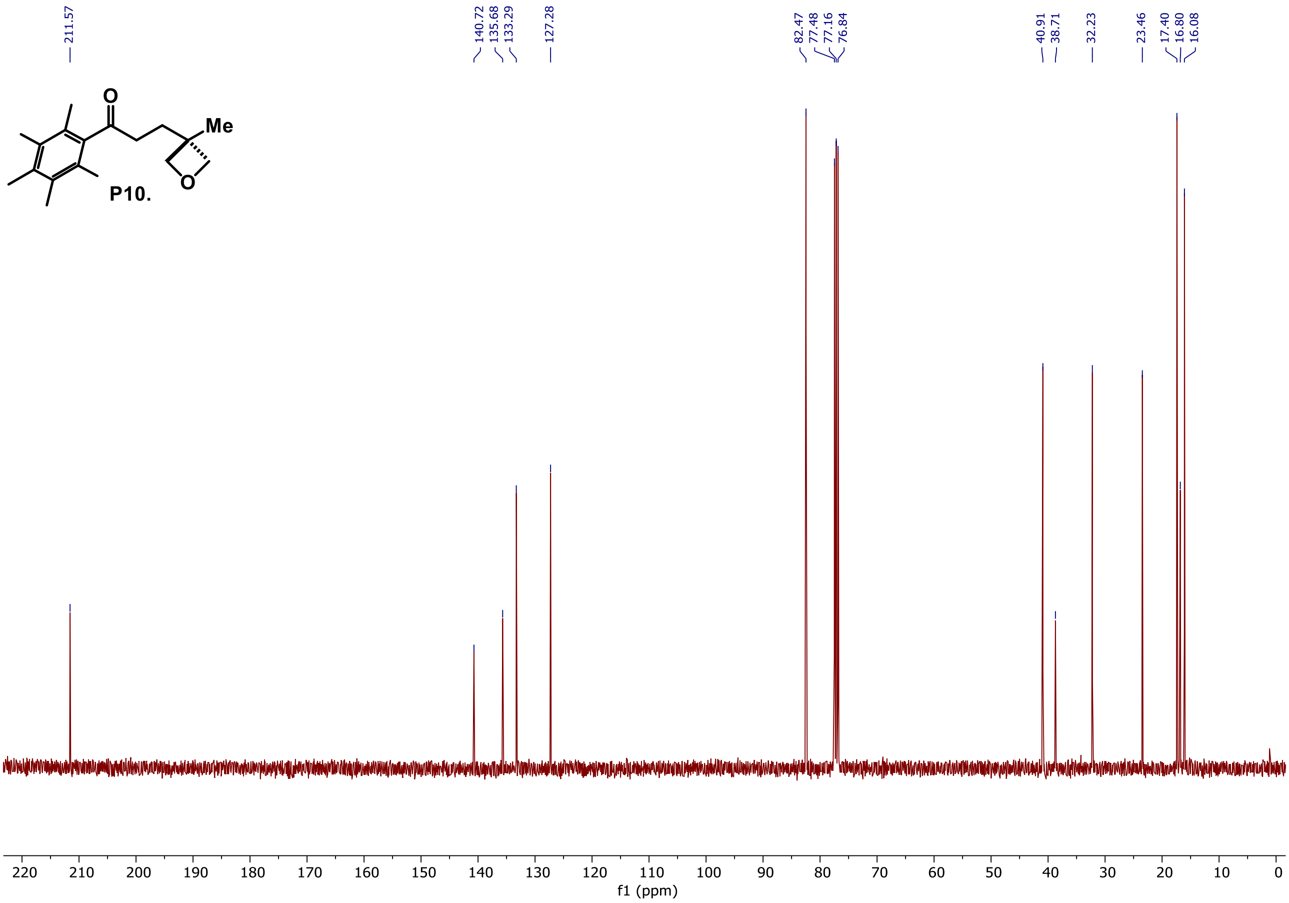

P11: ^{1}**H NMR** (400 MHz, $CDCl_3$) and ^{13}**C NMR** (101 MHz, $CDCl_3$) respectively.

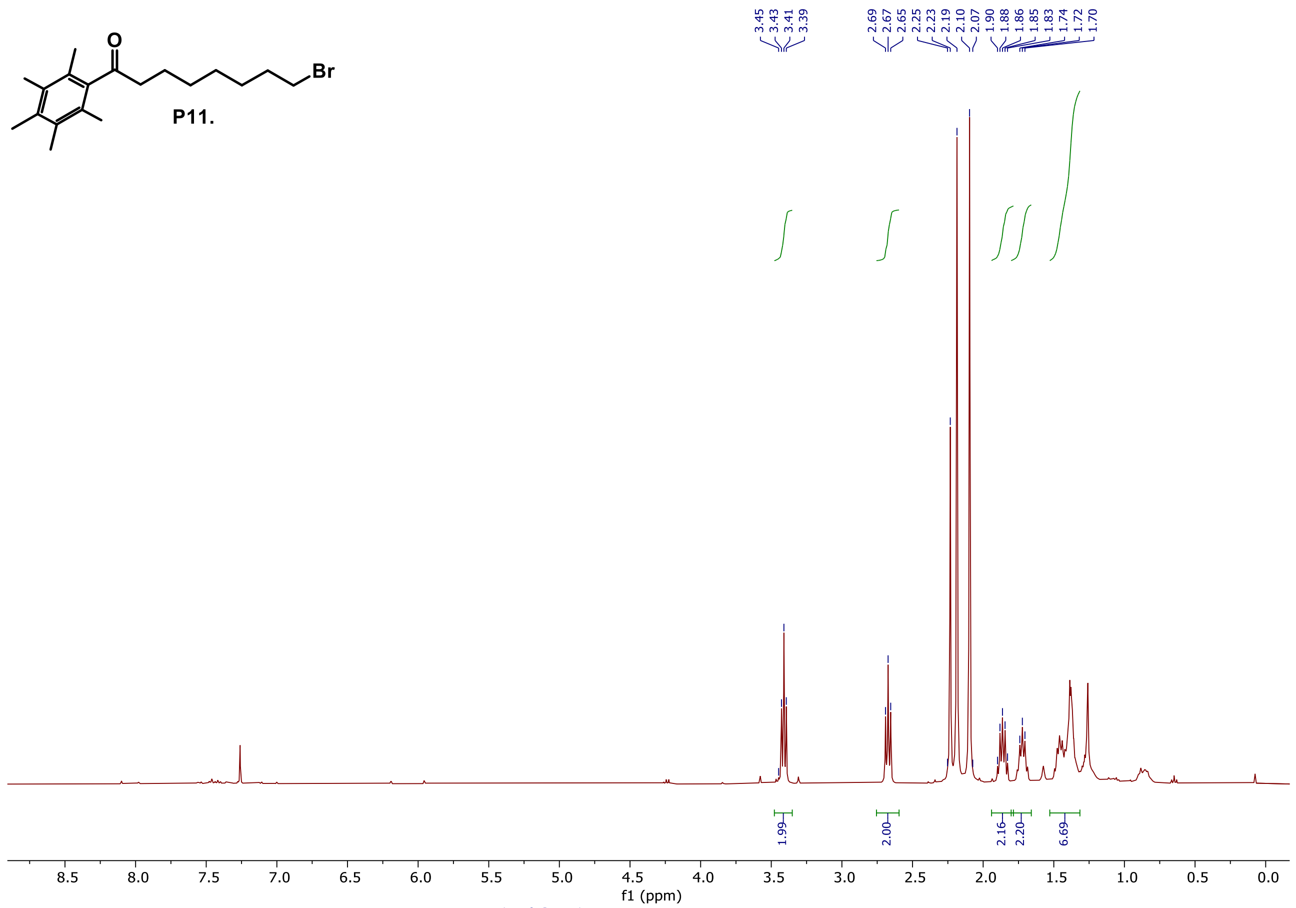

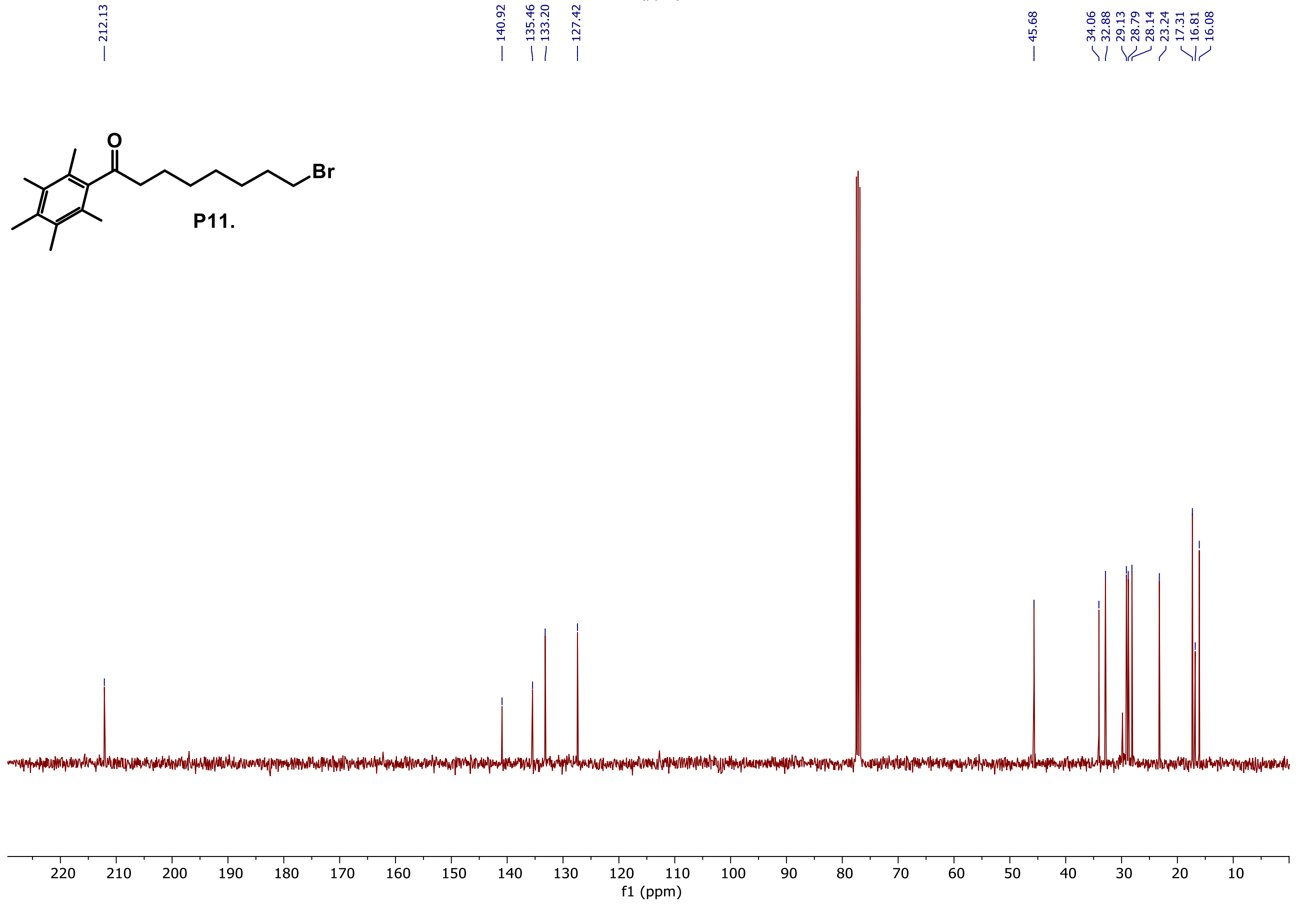

P12: [1]**H NMR** (400 MHz, $CDCl_3$) and [13]**C NMR** (101 MHz, $CDCl_3$) respectively.

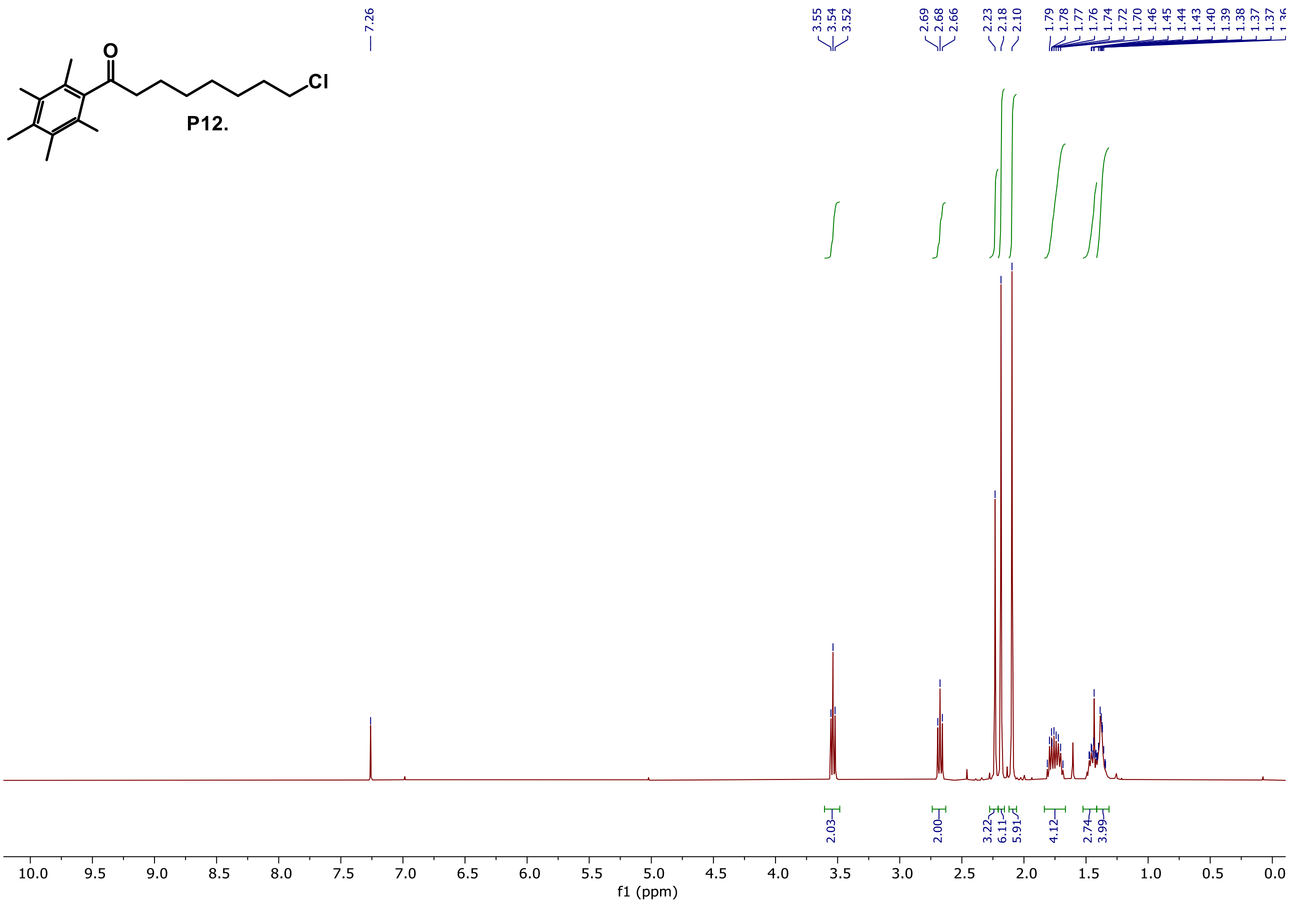

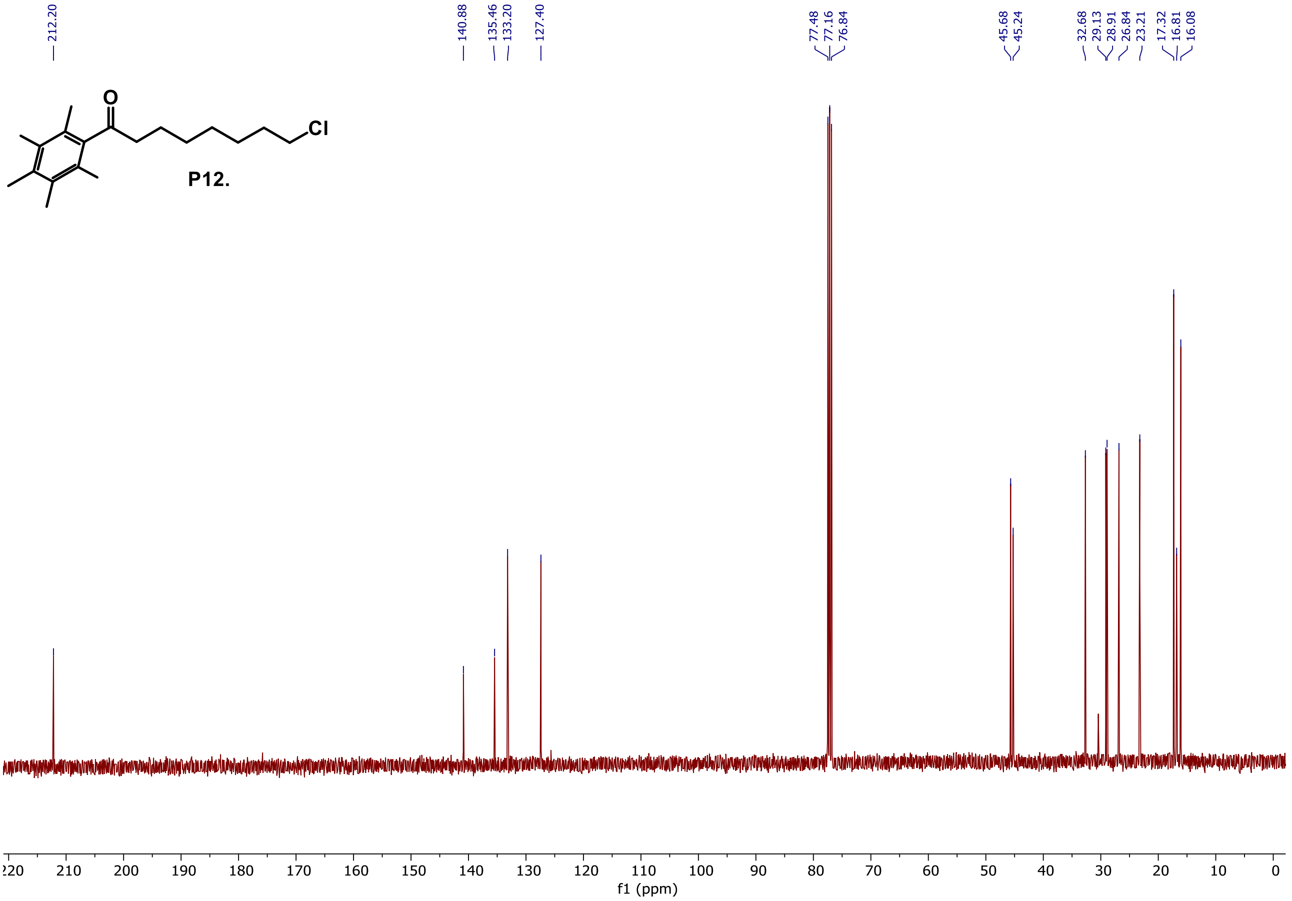

P13: [1]**H NMR** (400 MHz, $CDCl_3$) and [13]**C NMR** (101 MHz, $CDCl_3$) respectively.

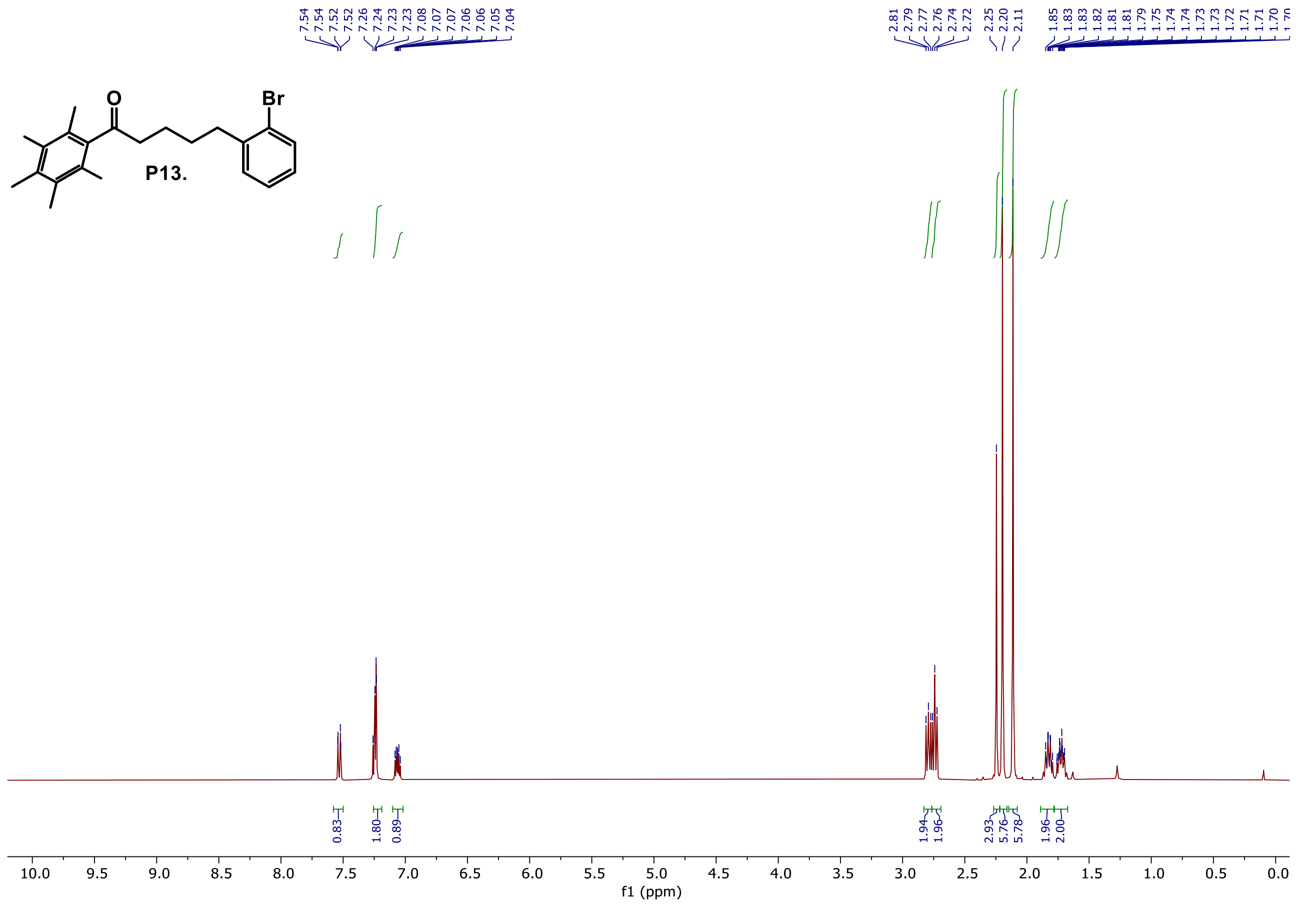

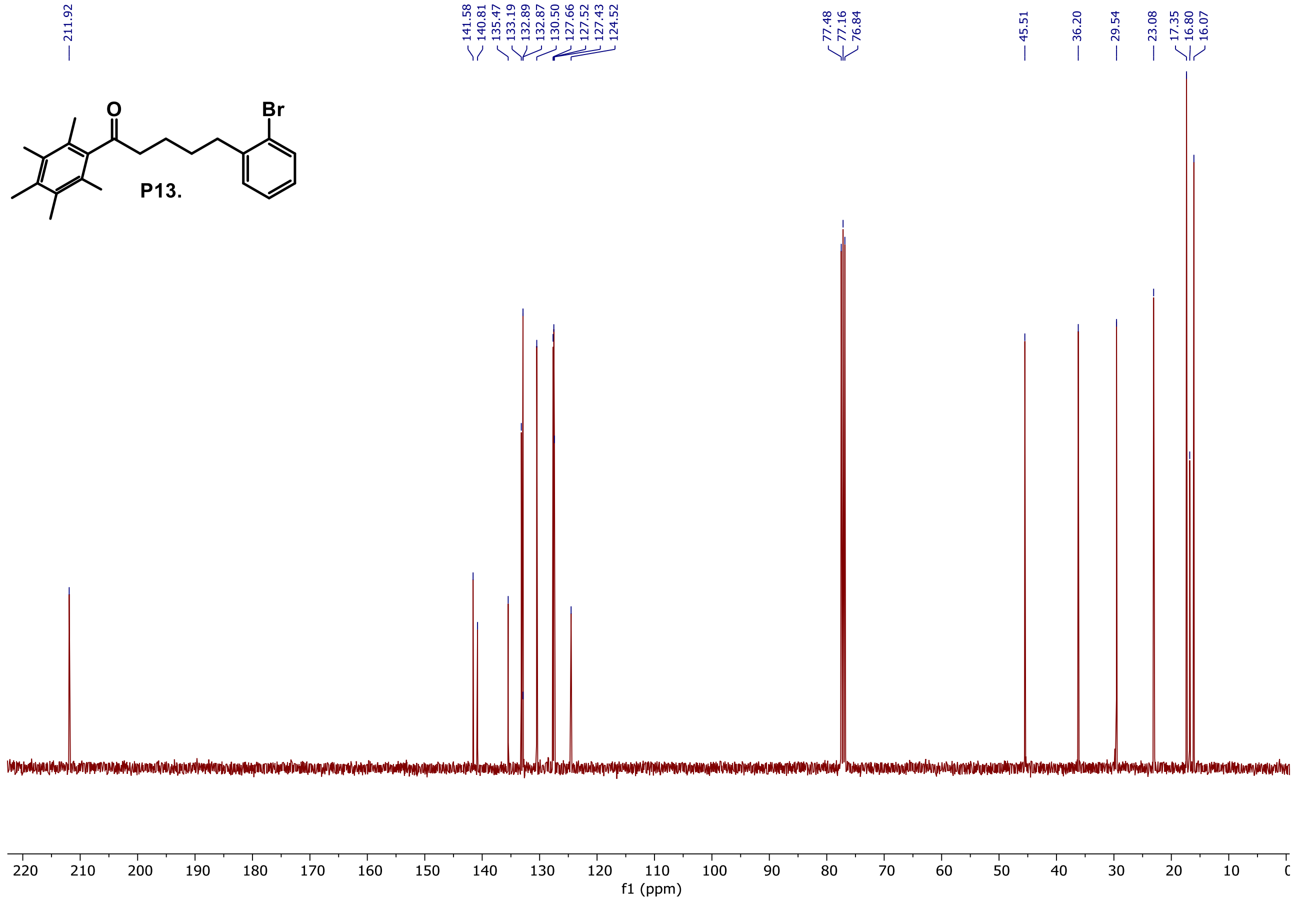

P14: ¹H NMR (400 MHz, $CDCl_3$) and **¹³C NMR** (101 MHz, $CDCl_3$) respectively.

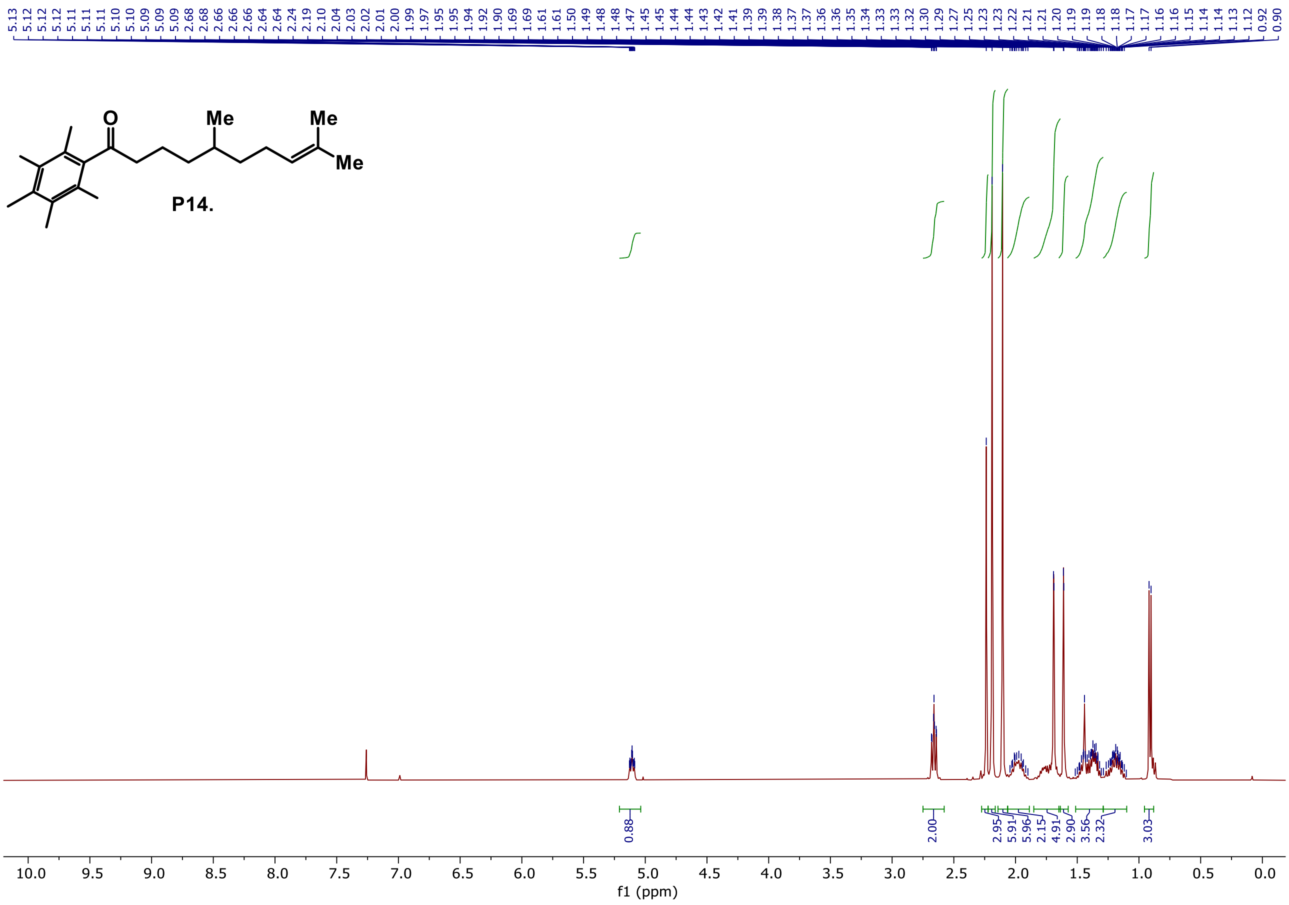

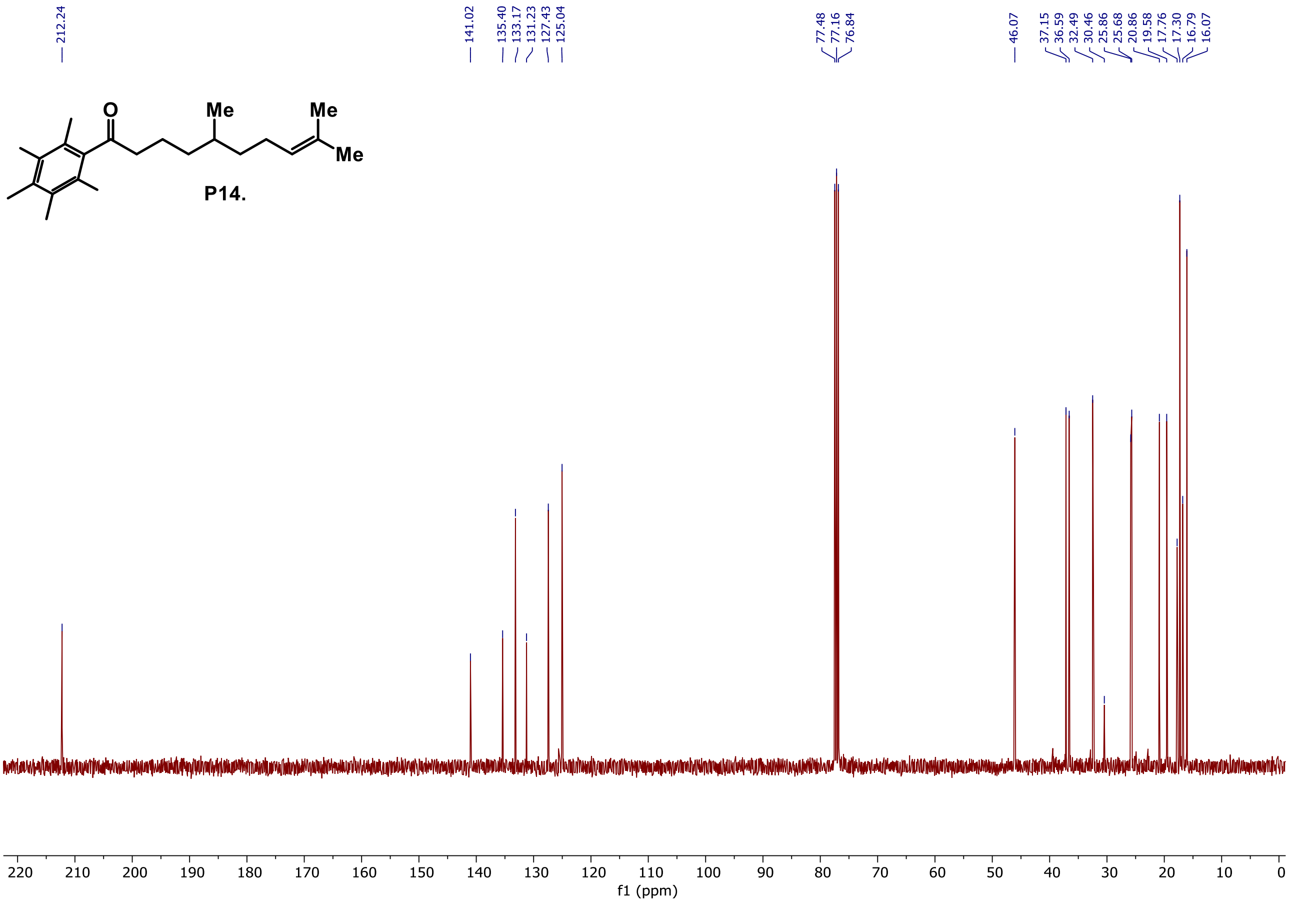

P15: **^{1}H NMR** (400 MHz, $CDCl_3$) and **^{13}C NMR** (101 MHz, $CDCl_3$) respectively.

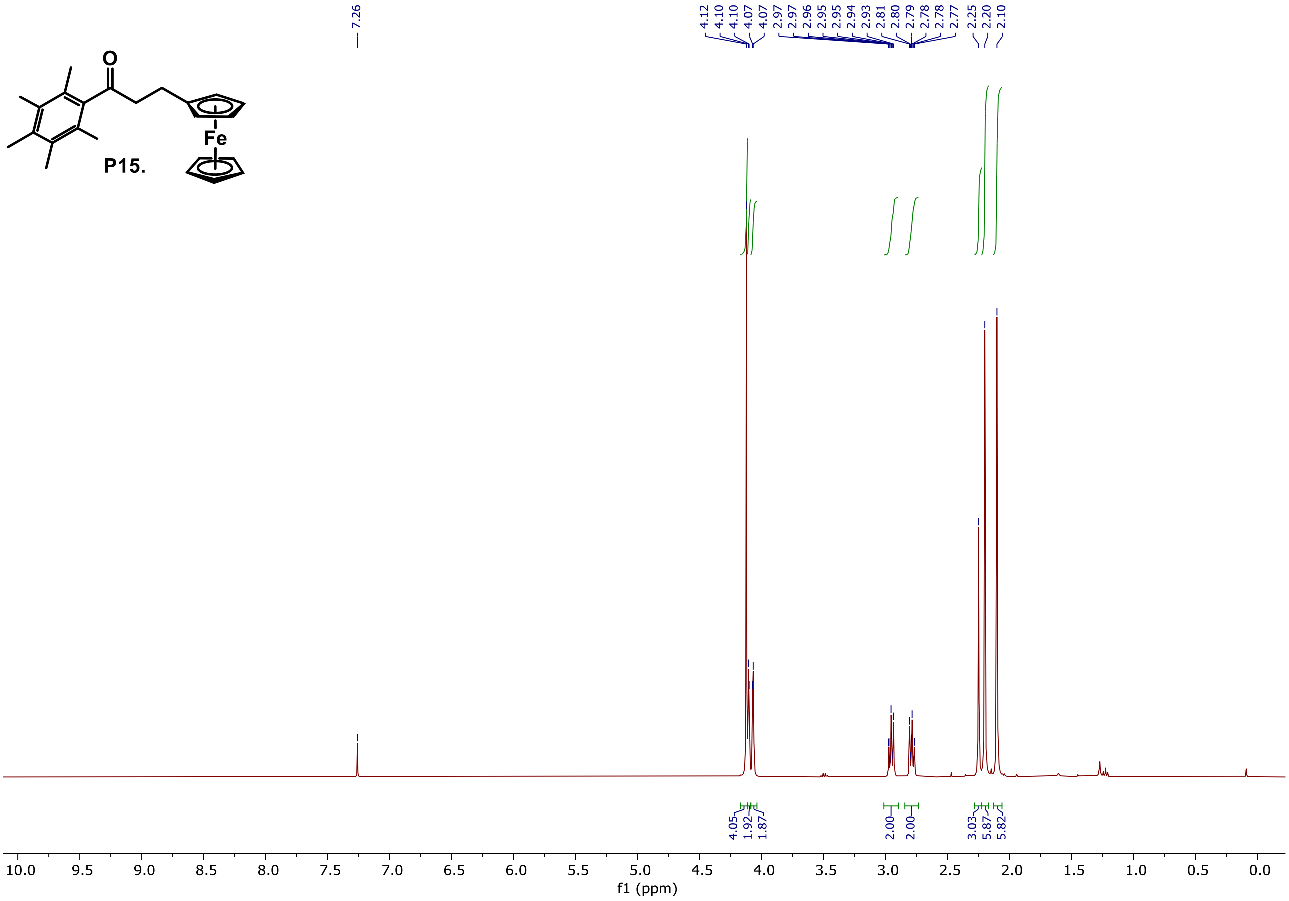

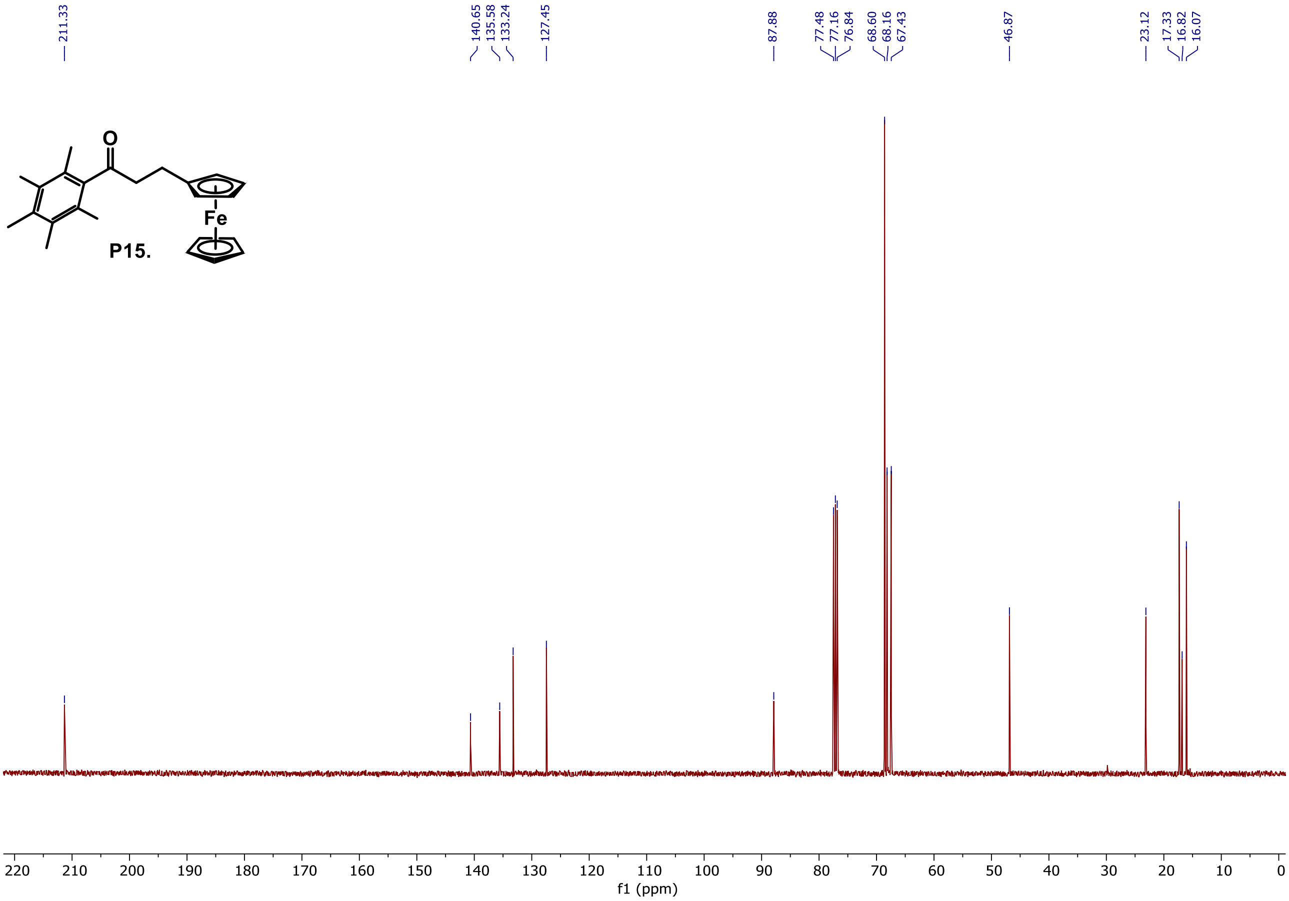

P16: ¹H NMR (400 MHz, $CDCl_3$) and **¹³C NMR** (101 MHz, $CDCl_3$) respectively.

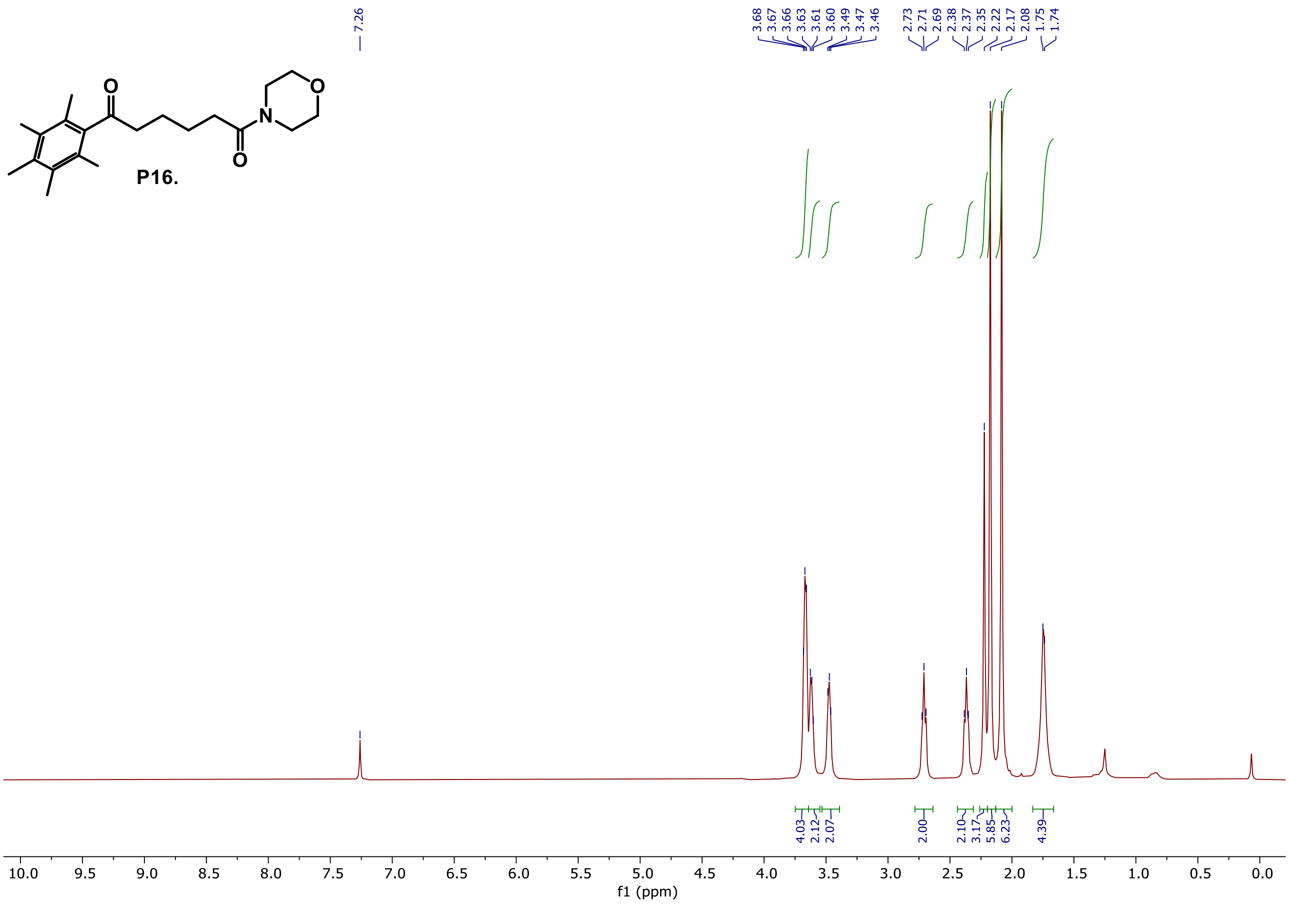

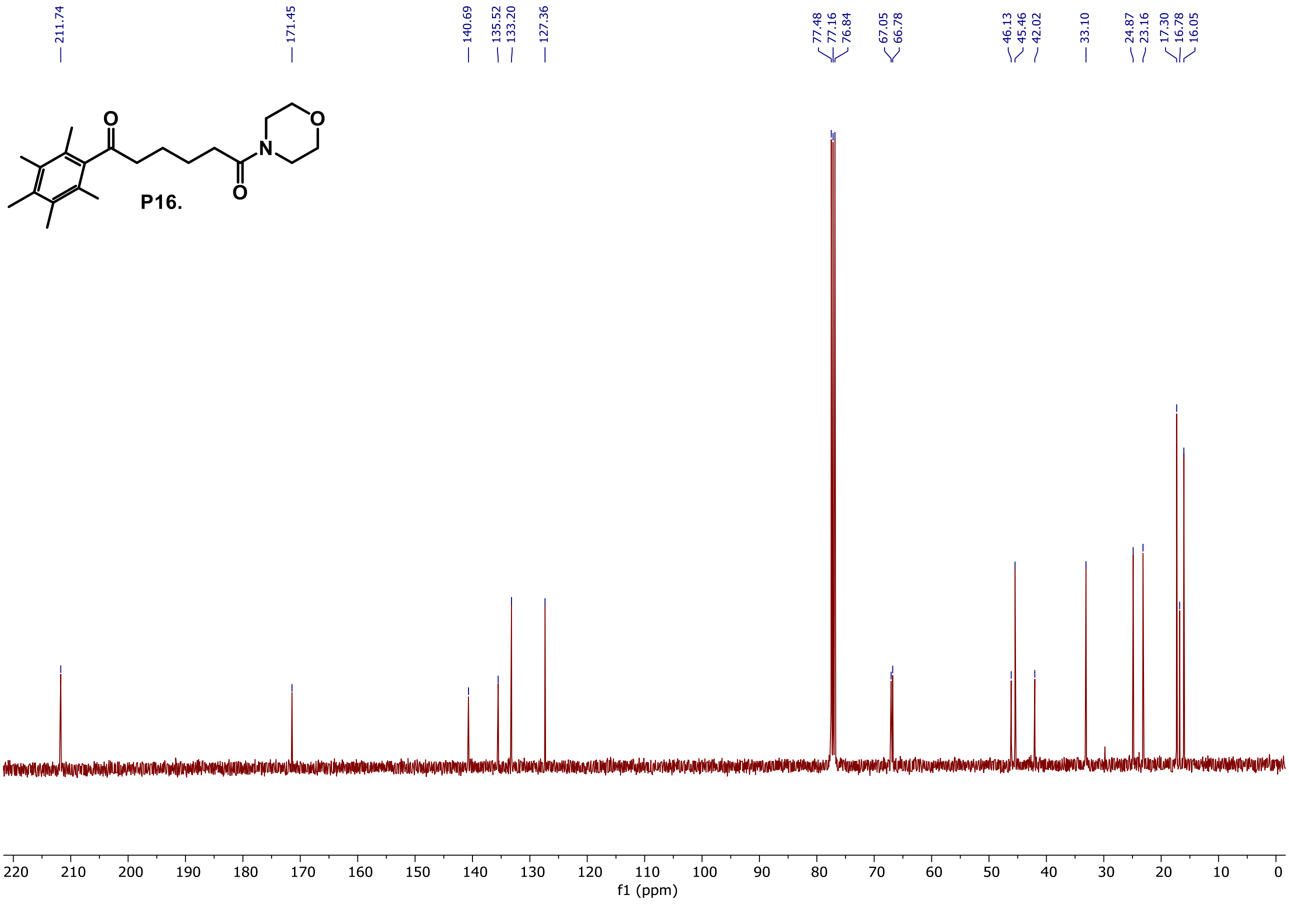

P17: ^{1}**H NMR** (400 MHz, $CDCl_3$) and ^{13}**C NMR** (101 MHz, $CDCl_3$) respectively.

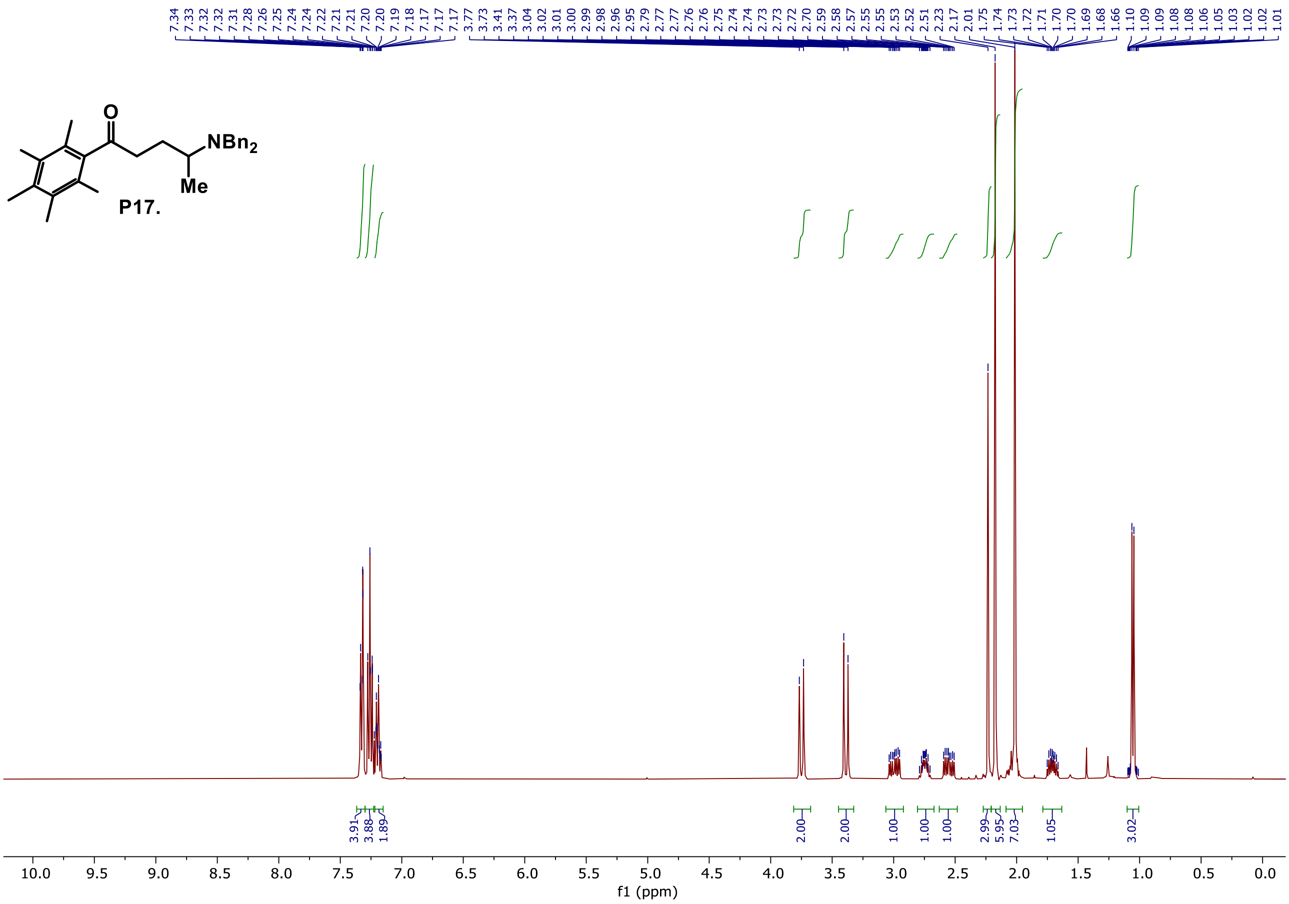

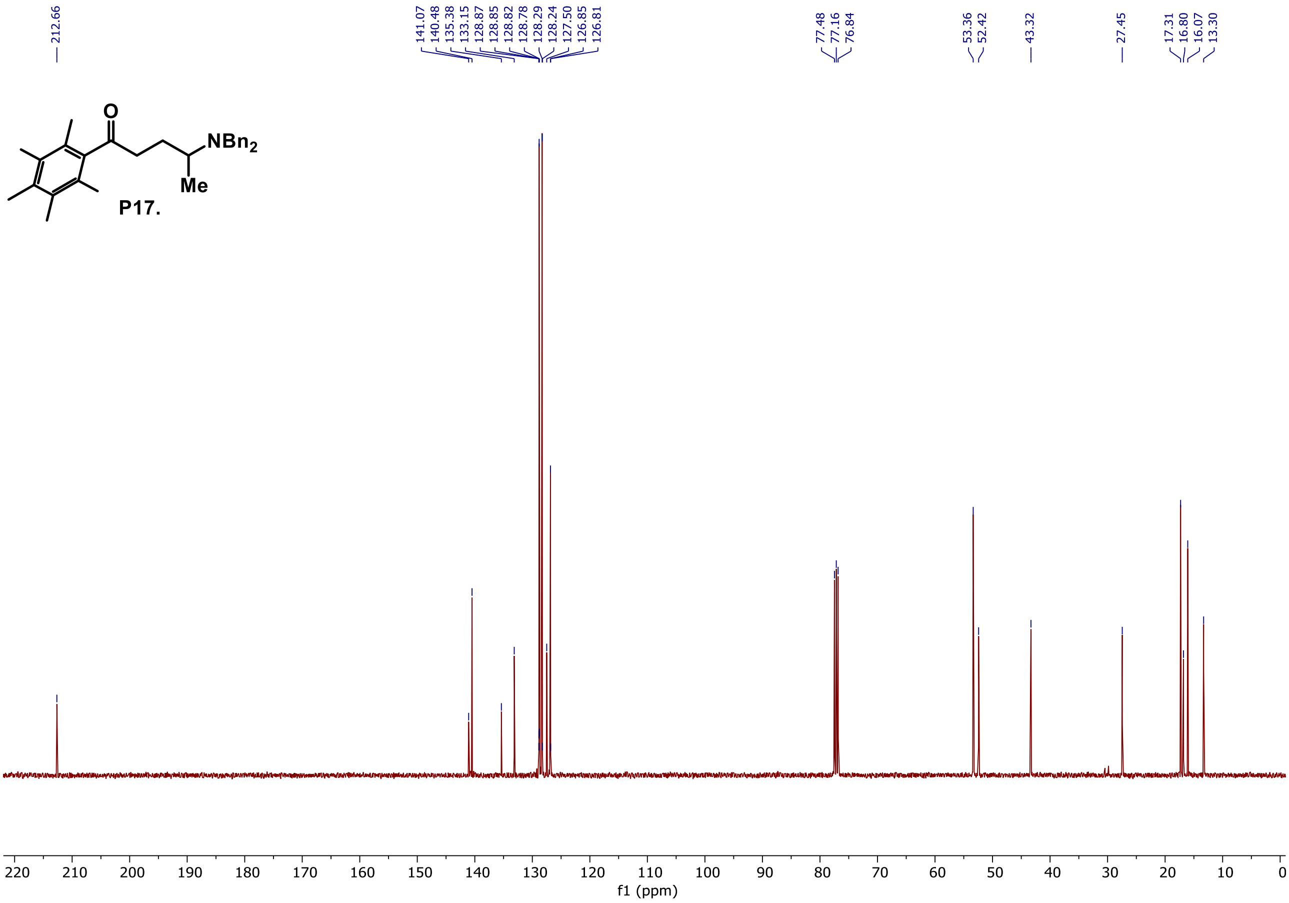

P18: ^{1}**H NMR** (400 MHz, $CDCl_3$) and ^{13}**C NMR** (101 MHz, $CDCl_3$) respectively.

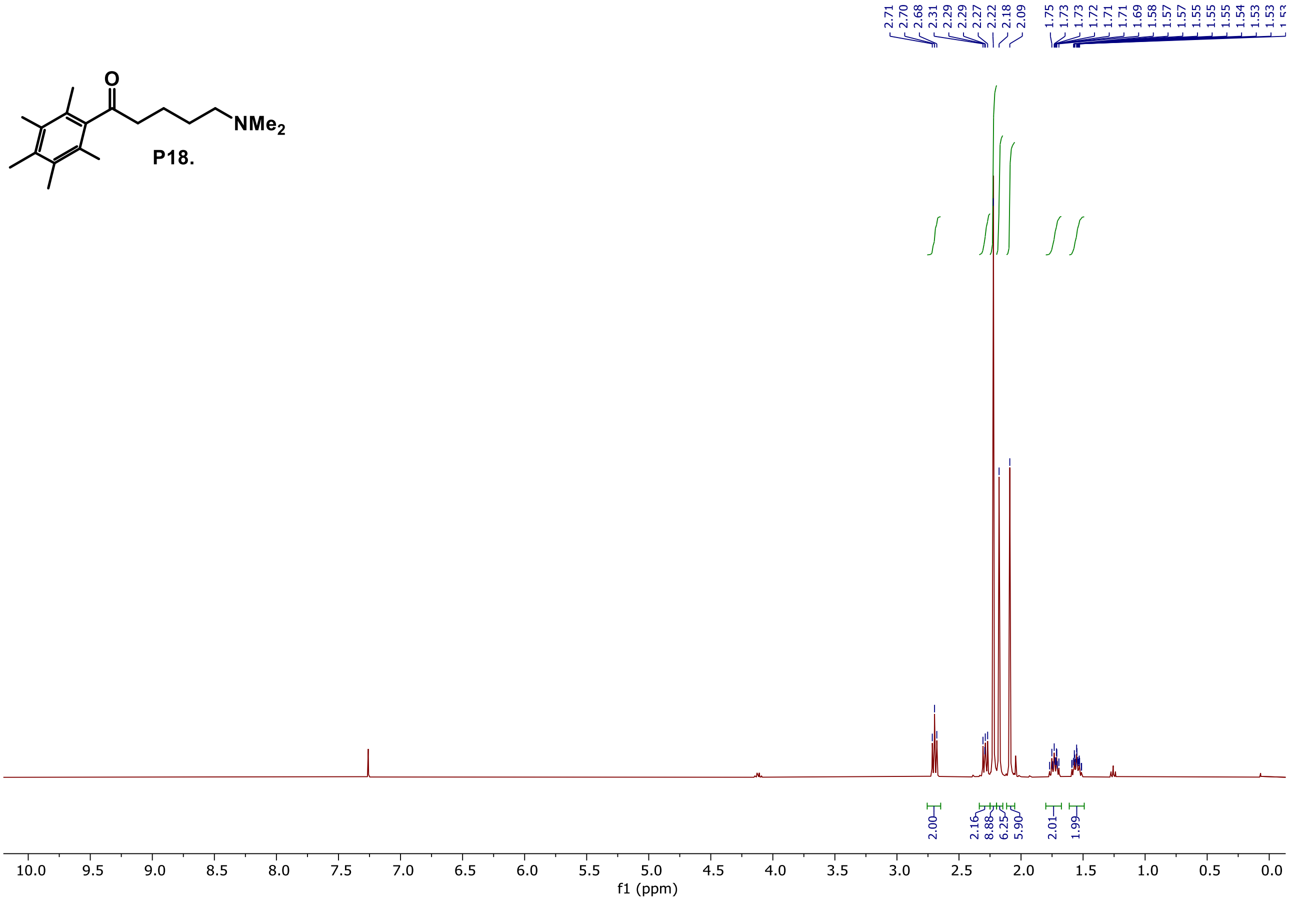

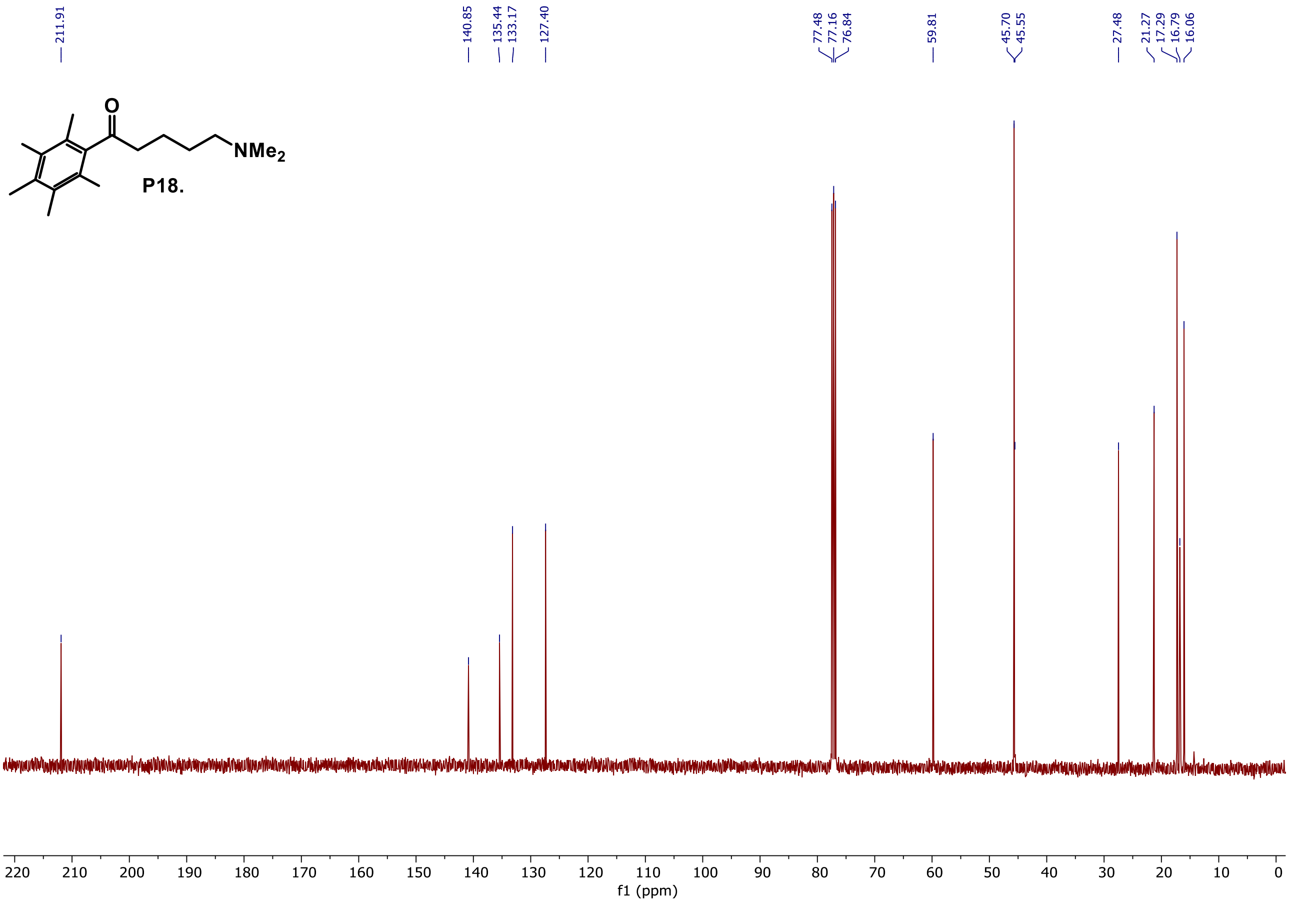

P19: **^{1}H NMR** (400 MHz, $CDCl_3$) and **^{13}C NMR** (101 MHz, $CDCl_3$) respectively.

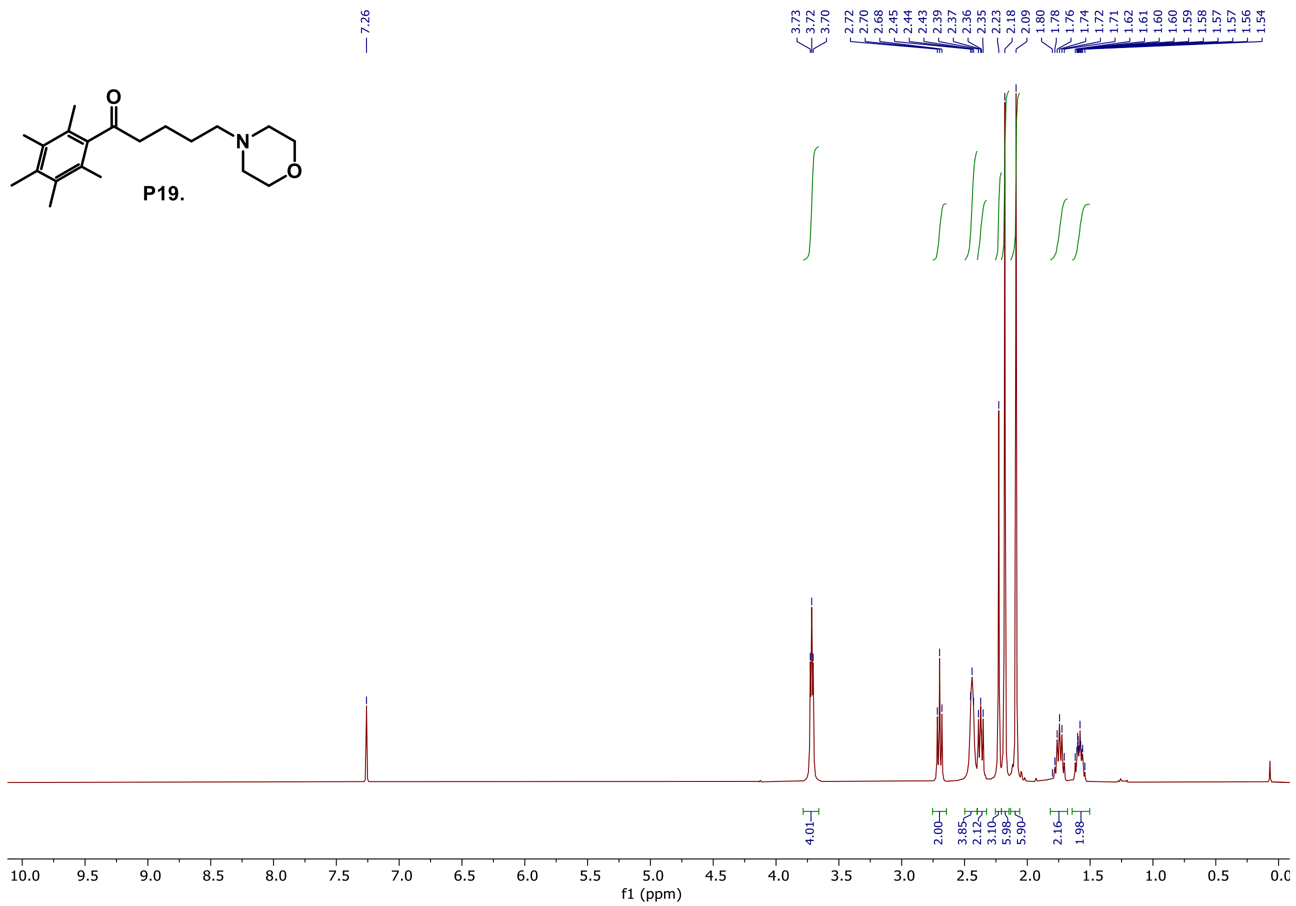

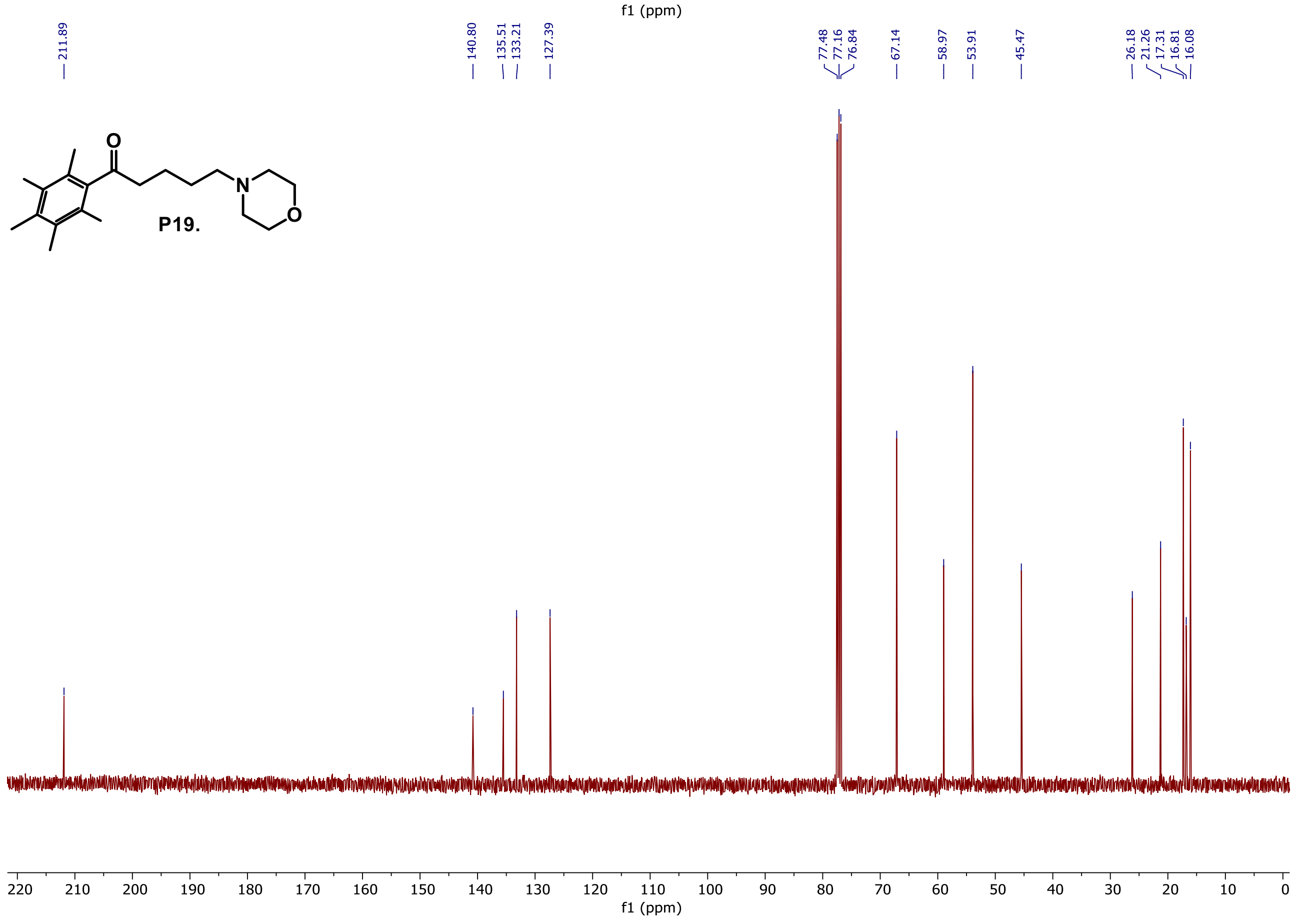

P20: ^{1}**H NMR** (400 MHz, $CDCl_3$) and ^{13}**C NMR** (101 MHz, $CDCl_3$) respectively.

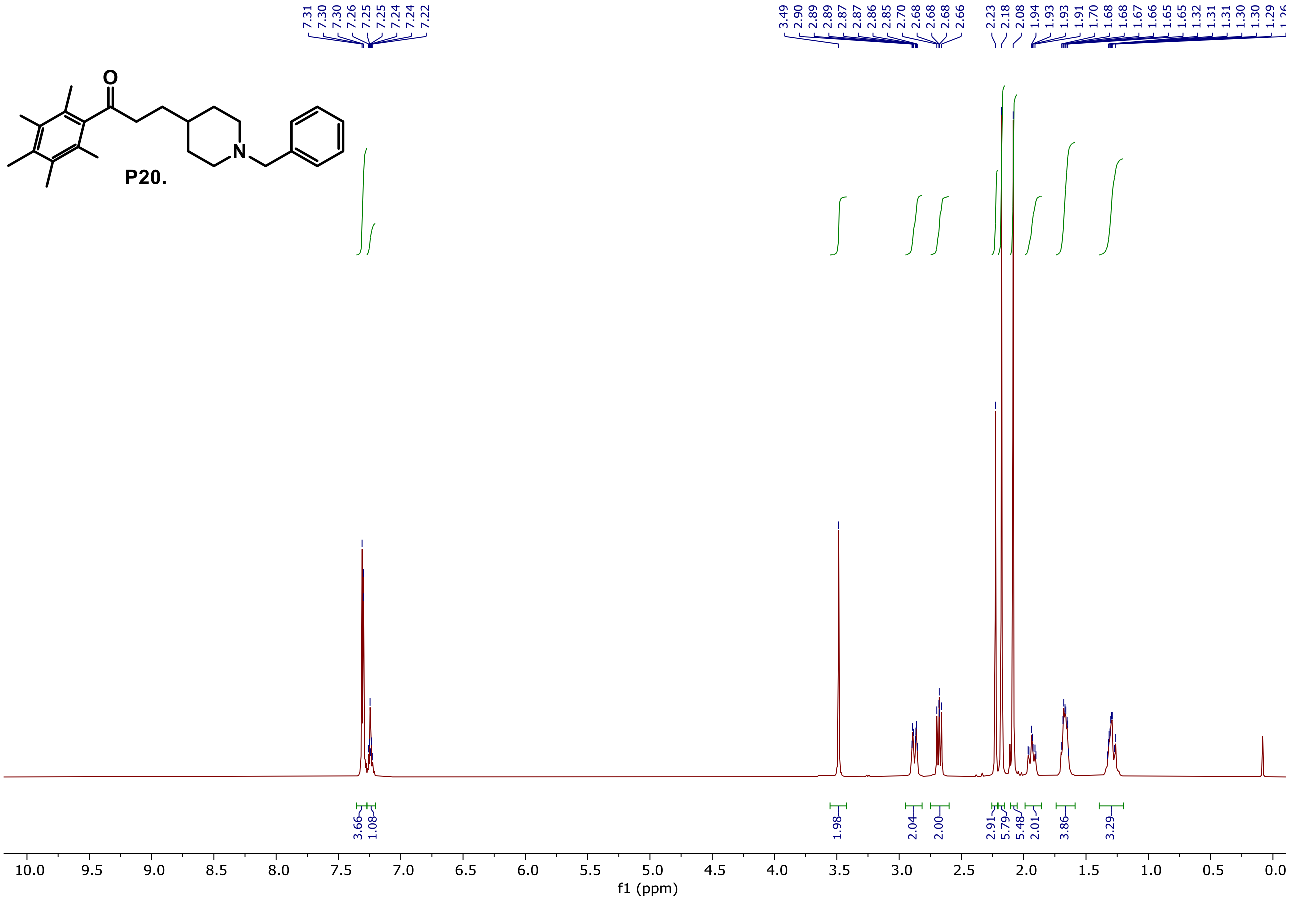

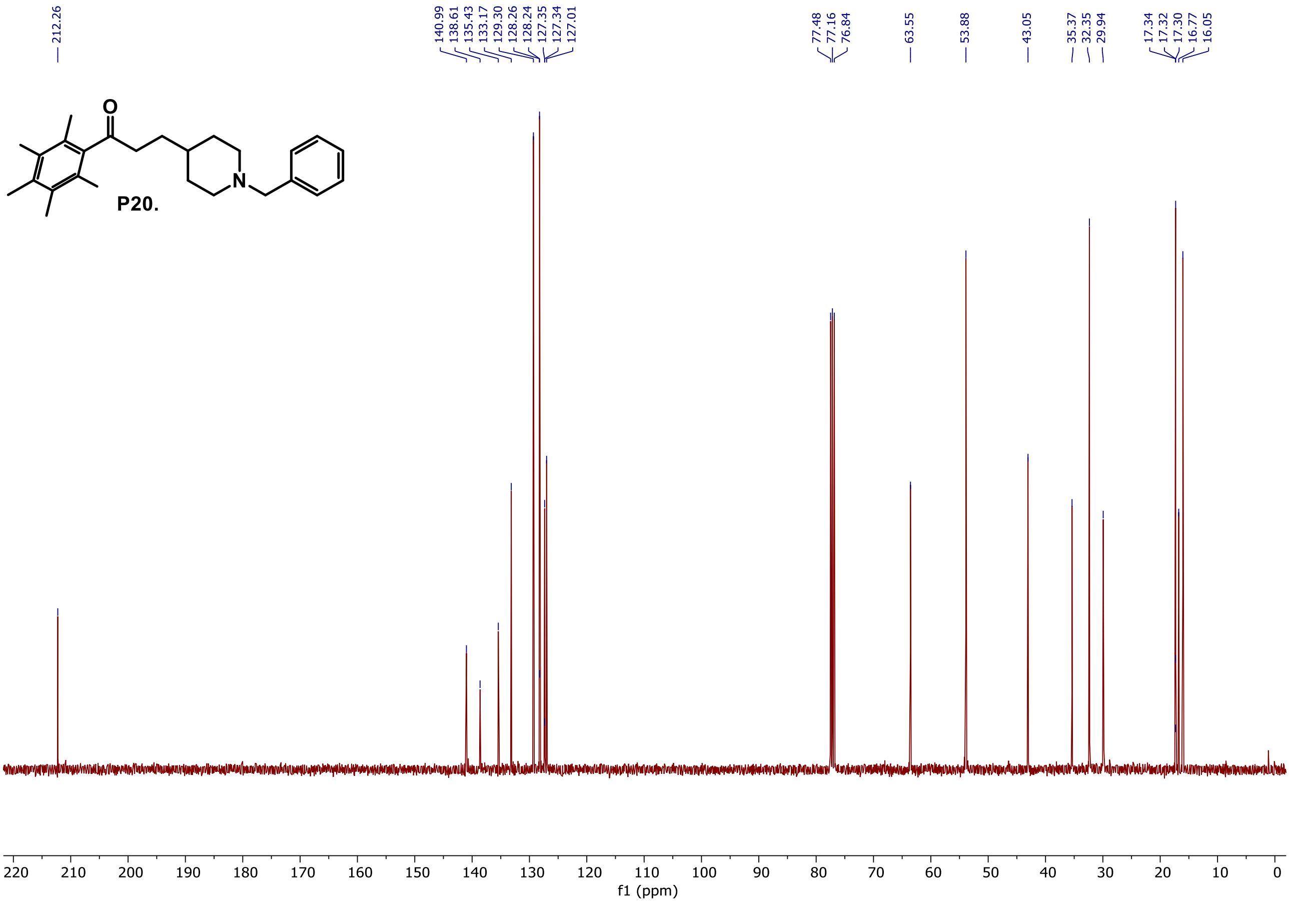

P21: **¹H NMR** (400 MHz, $CDCl_3$) and **¹³C NMR** (101 MHz, $CDCl_3$) respectively.

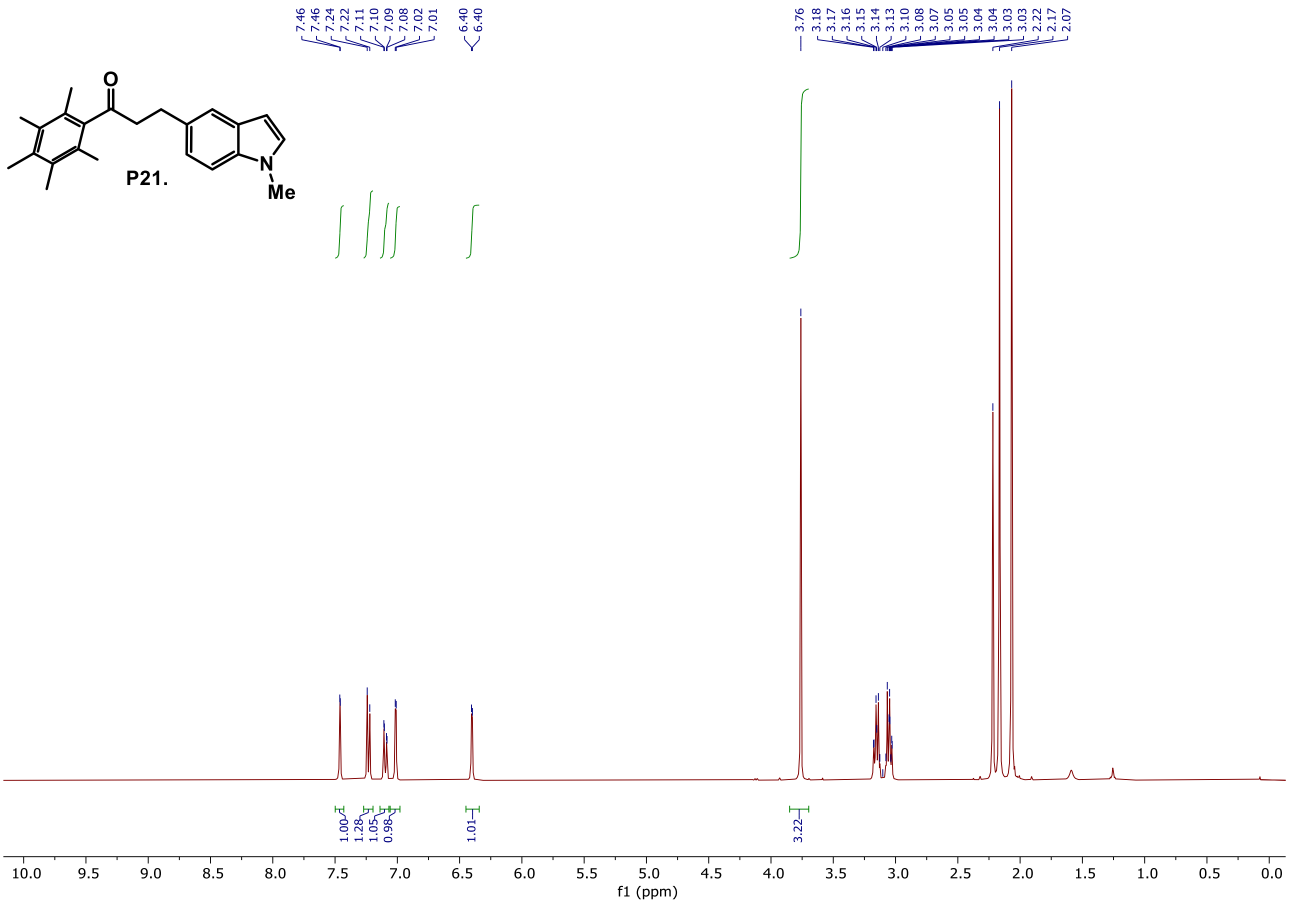

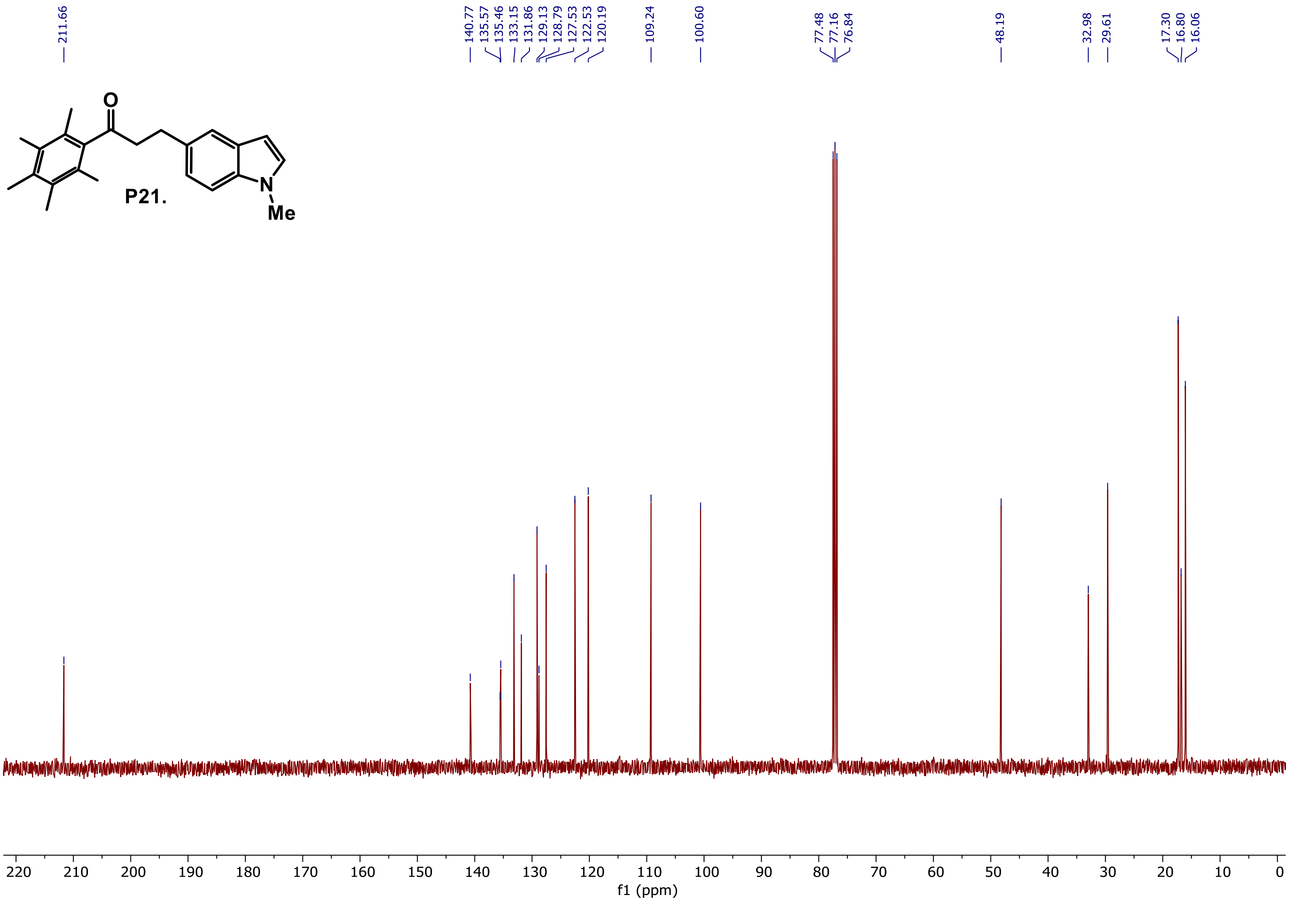

22: **^{1}H NMR** (400 MHz, $CDCl_3$) and **^{13}C NMR** (101 MHz, $CDCl_3$) respectively.

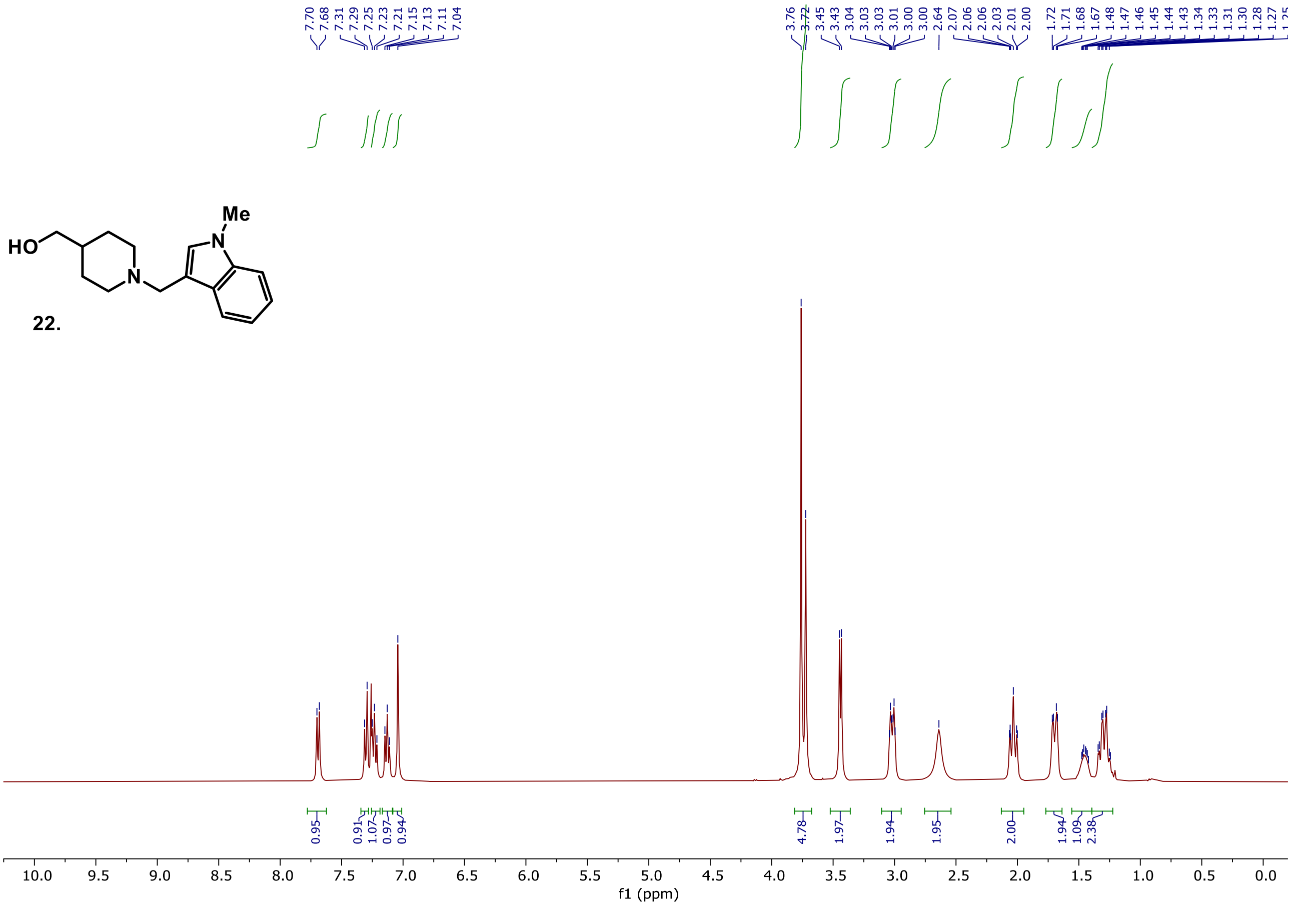

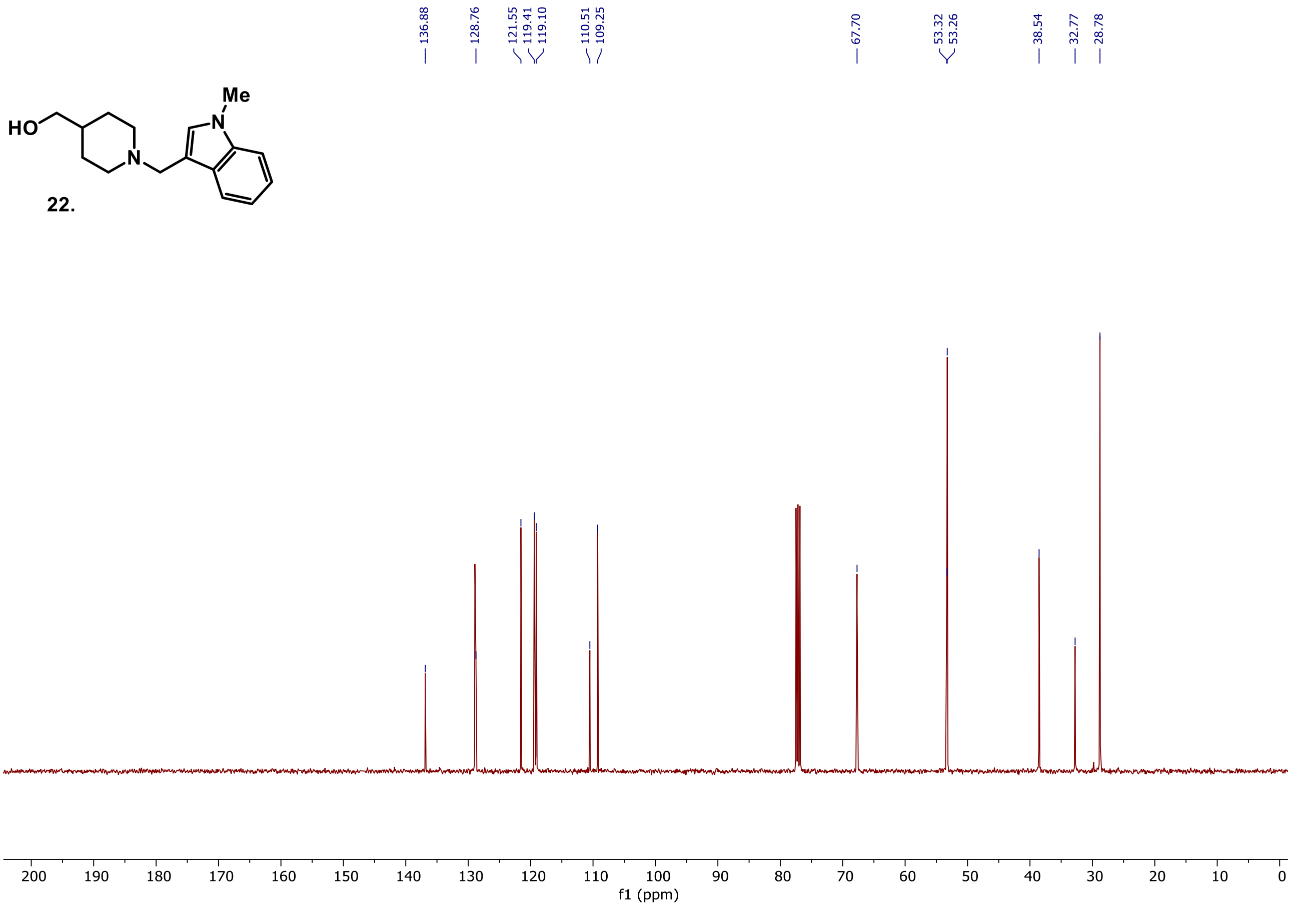

P22: **^{1}H NMR** (400 MHz, $CDCl_3$) and **^{13}C NMR** (101 MHz, $CDCl_3$) respectively.

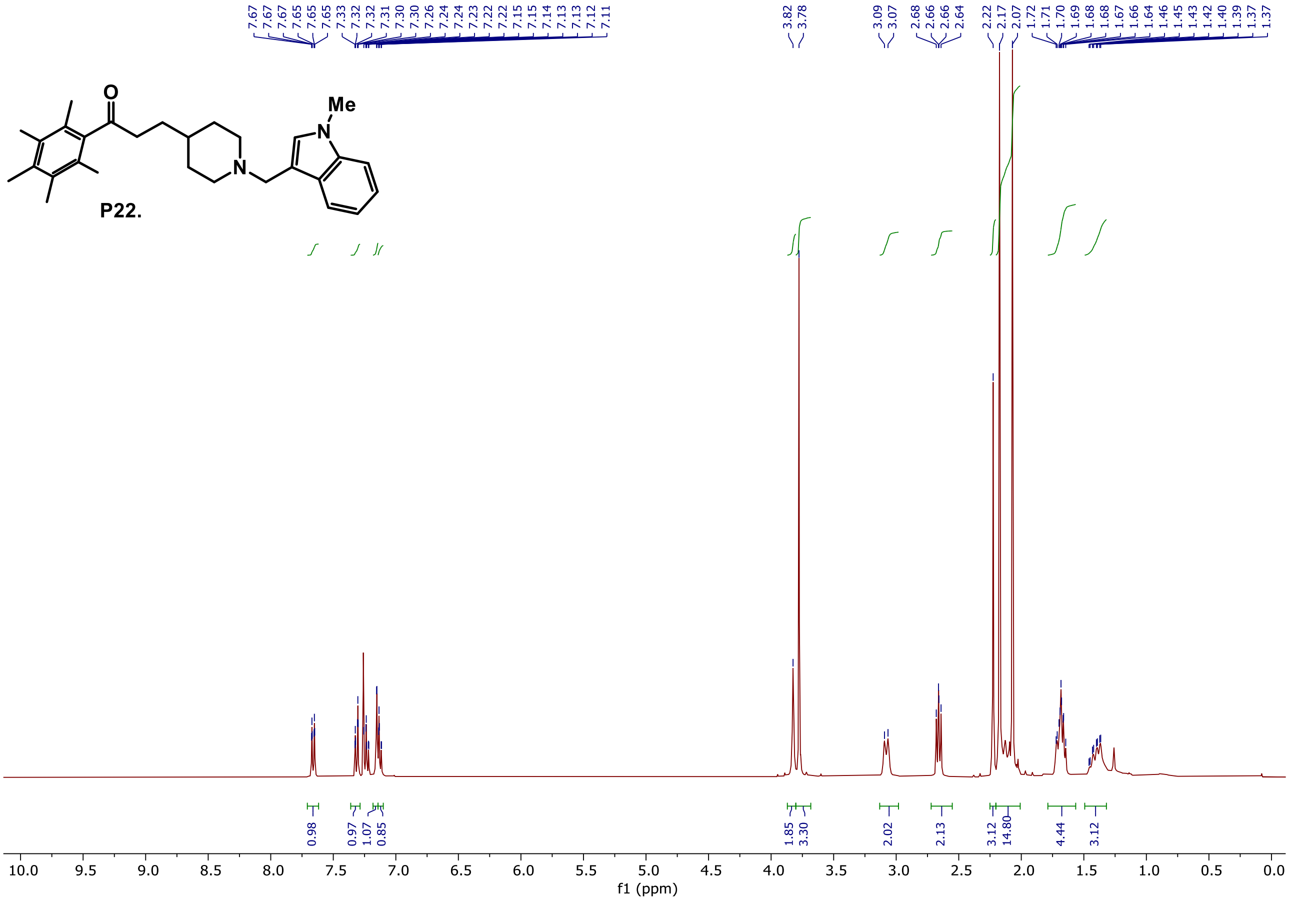

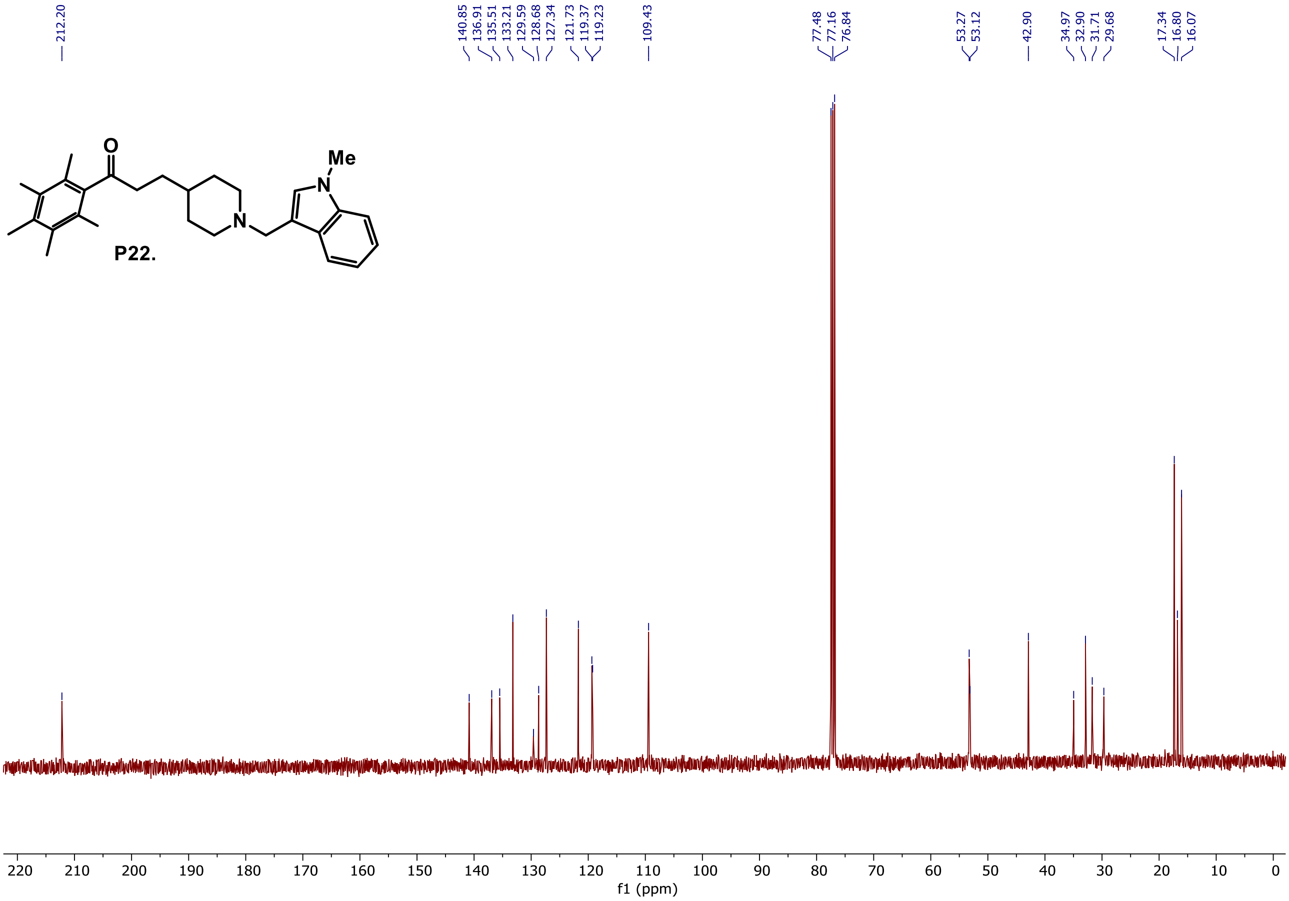

23: ¹H NMR (400 MHz, $CDCl_3$) and **¹³C NMR** (101 MHz, $CDCl_3$) respectively.

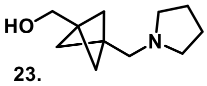

23.

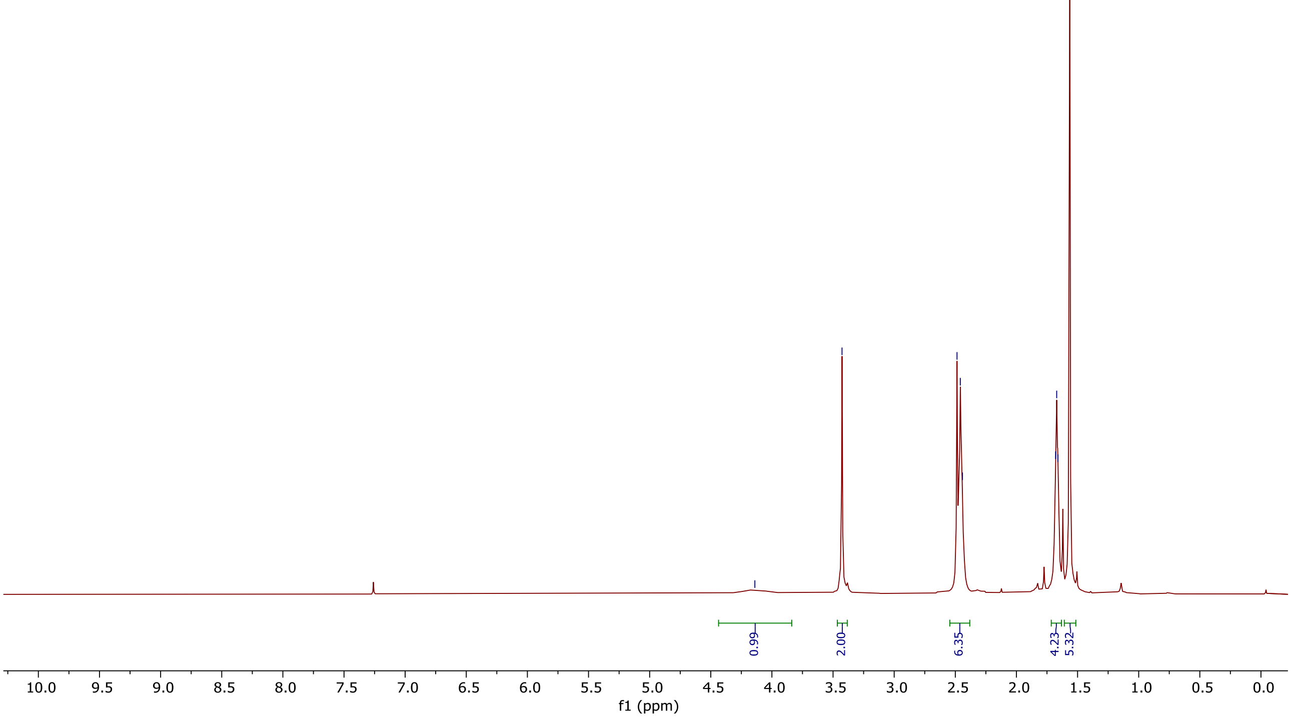

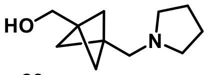

23.

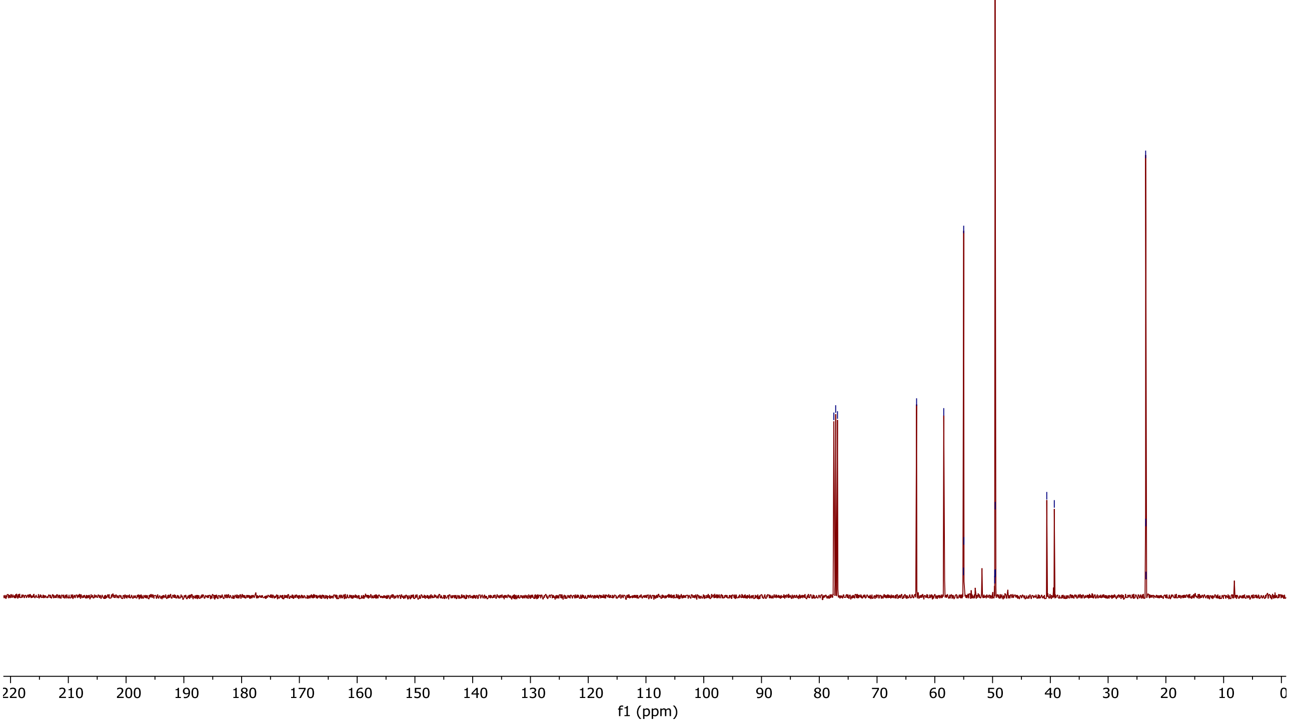

P23: ¹H NMR (400 MHz, CDCl$_3$) and **¹³C NMR** (101 MHz, CDCl$_3$) respectively.

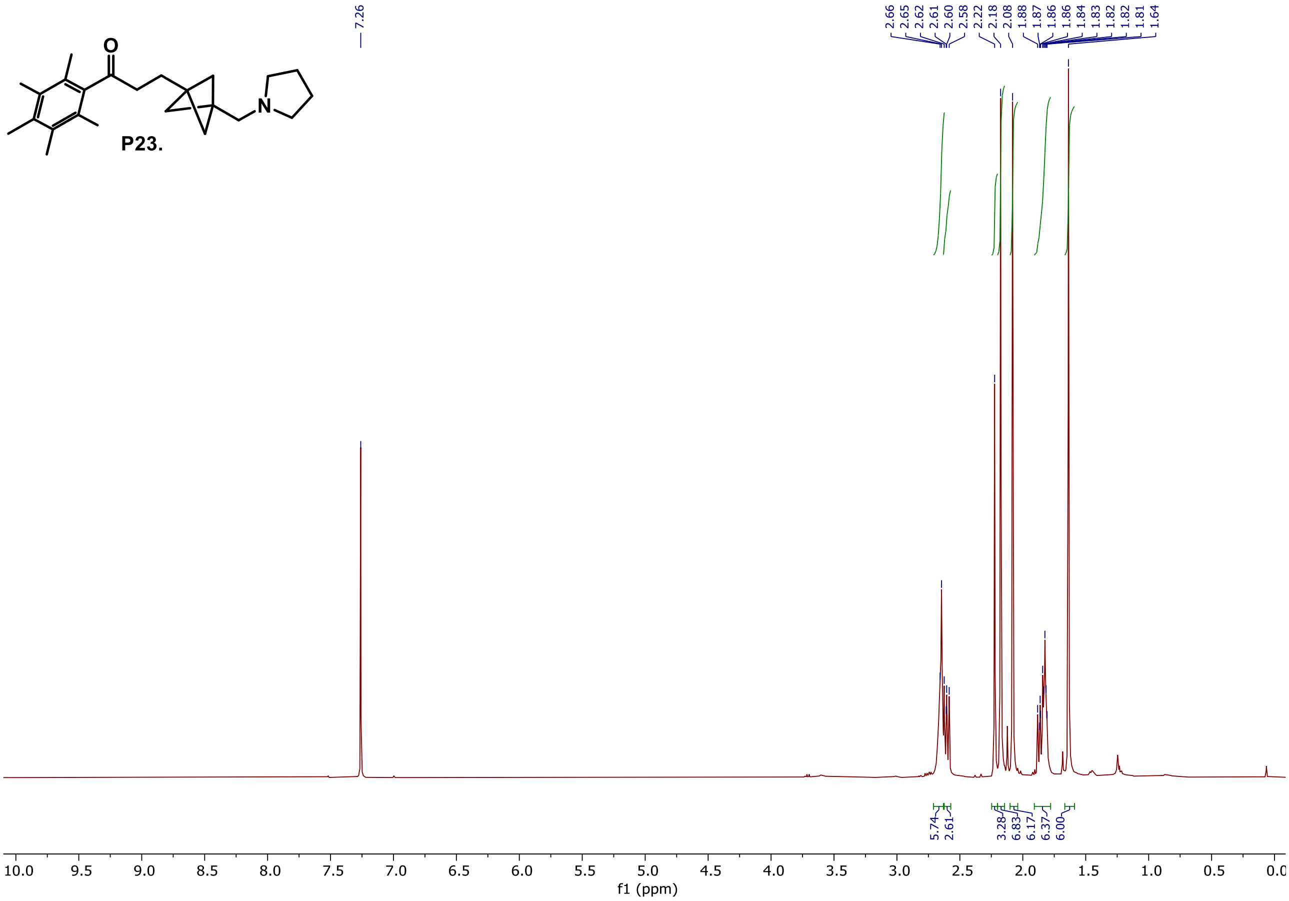

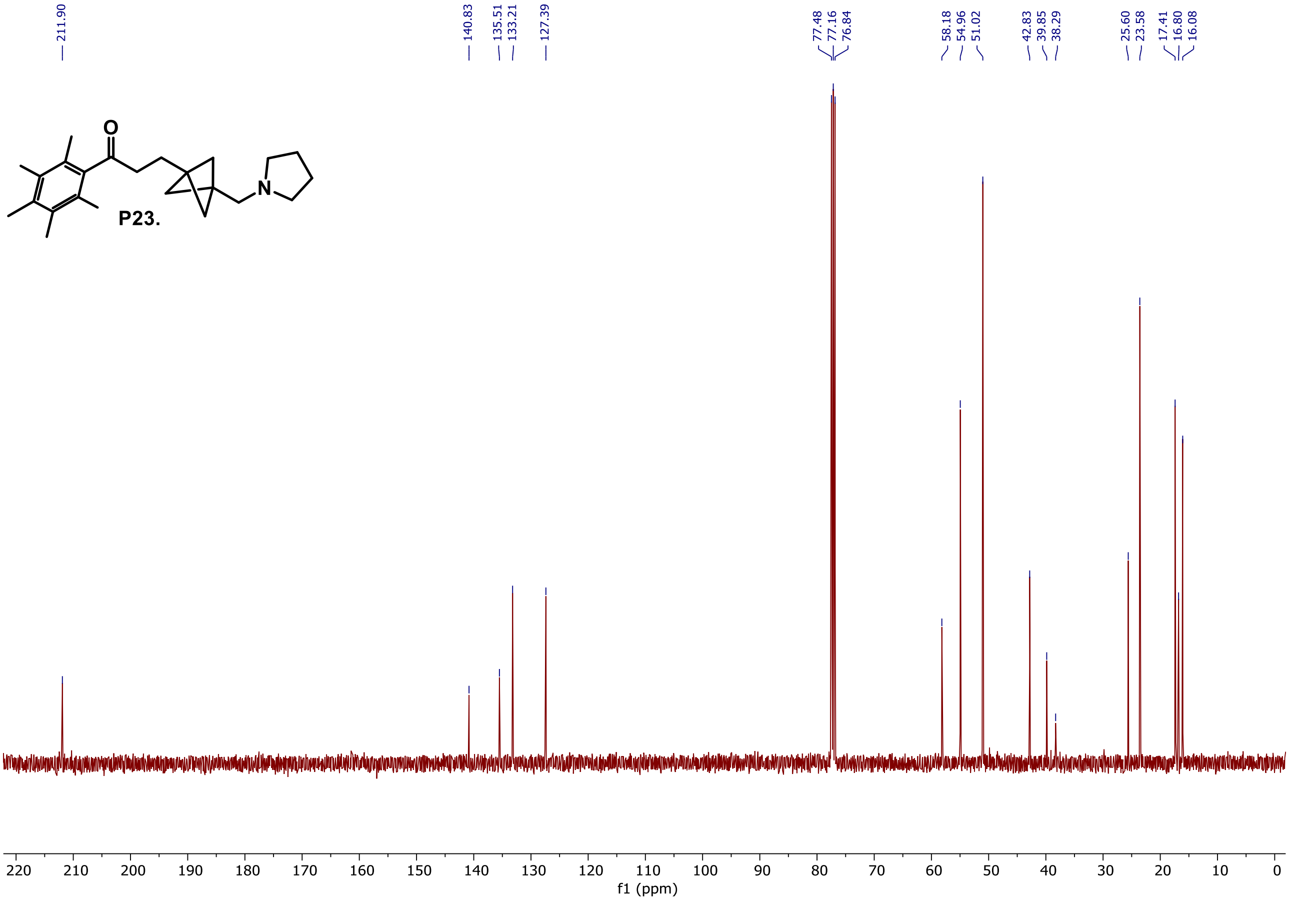

P24: **^{1}H NMR** (400 MHz, $CDCl_3$) and **^{13}C NMR** (101 MHz, $CDCl_3$) respectively.

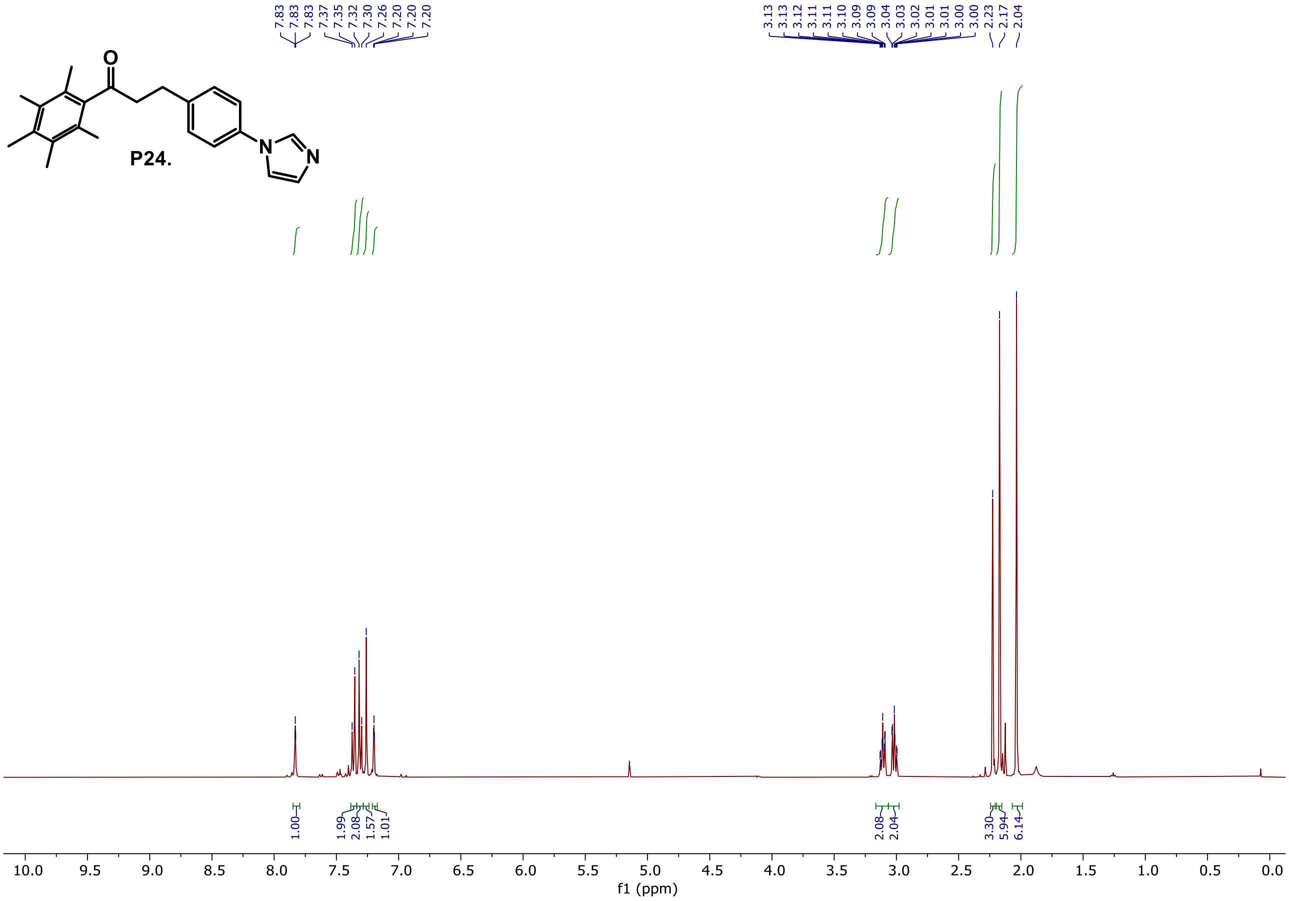

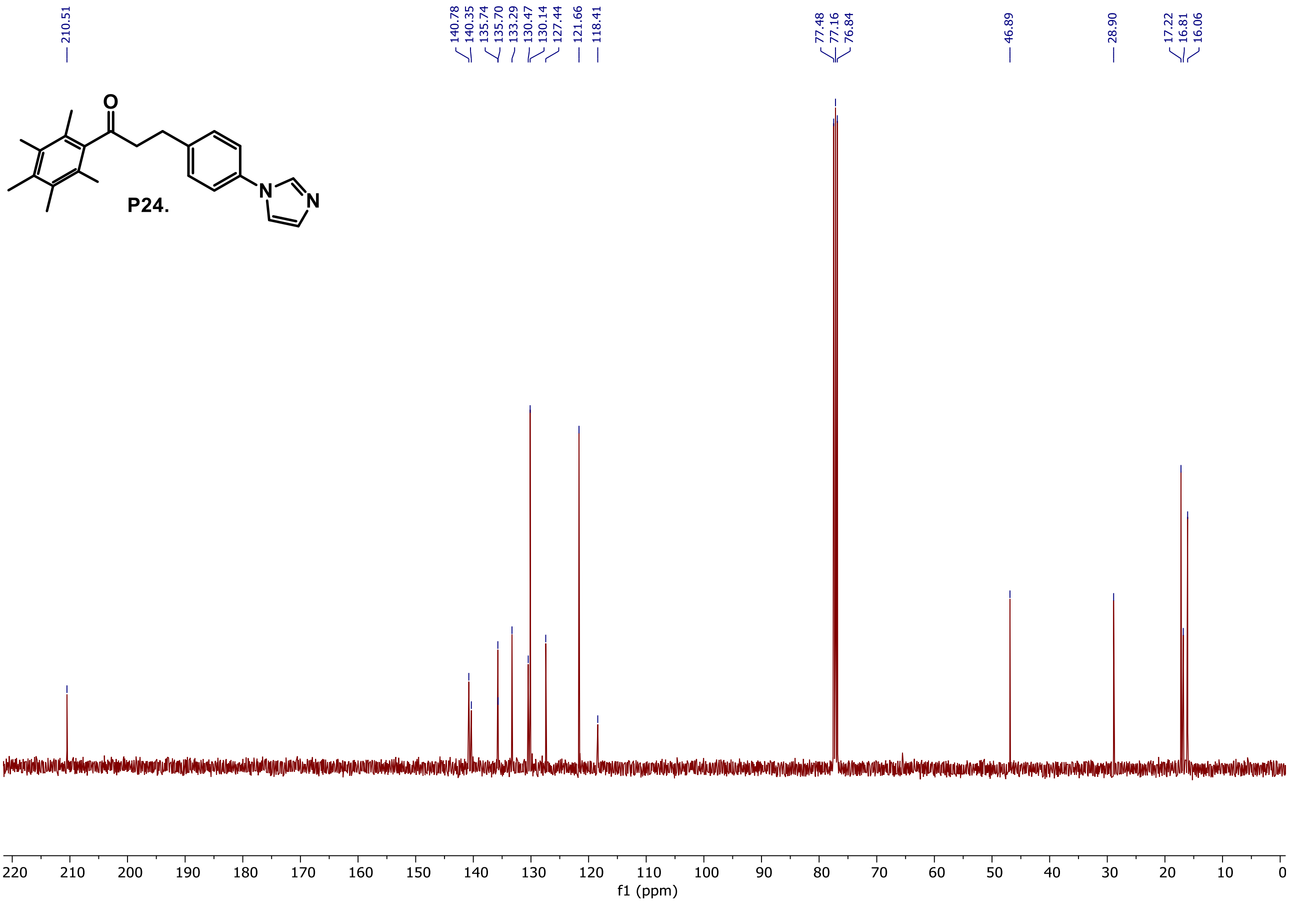

P25: **^{1}H NMR** (400 MHz, $CDCl_3$) and **^{13}C NMR** (101 MHz, $CDCl_3$) respectively.

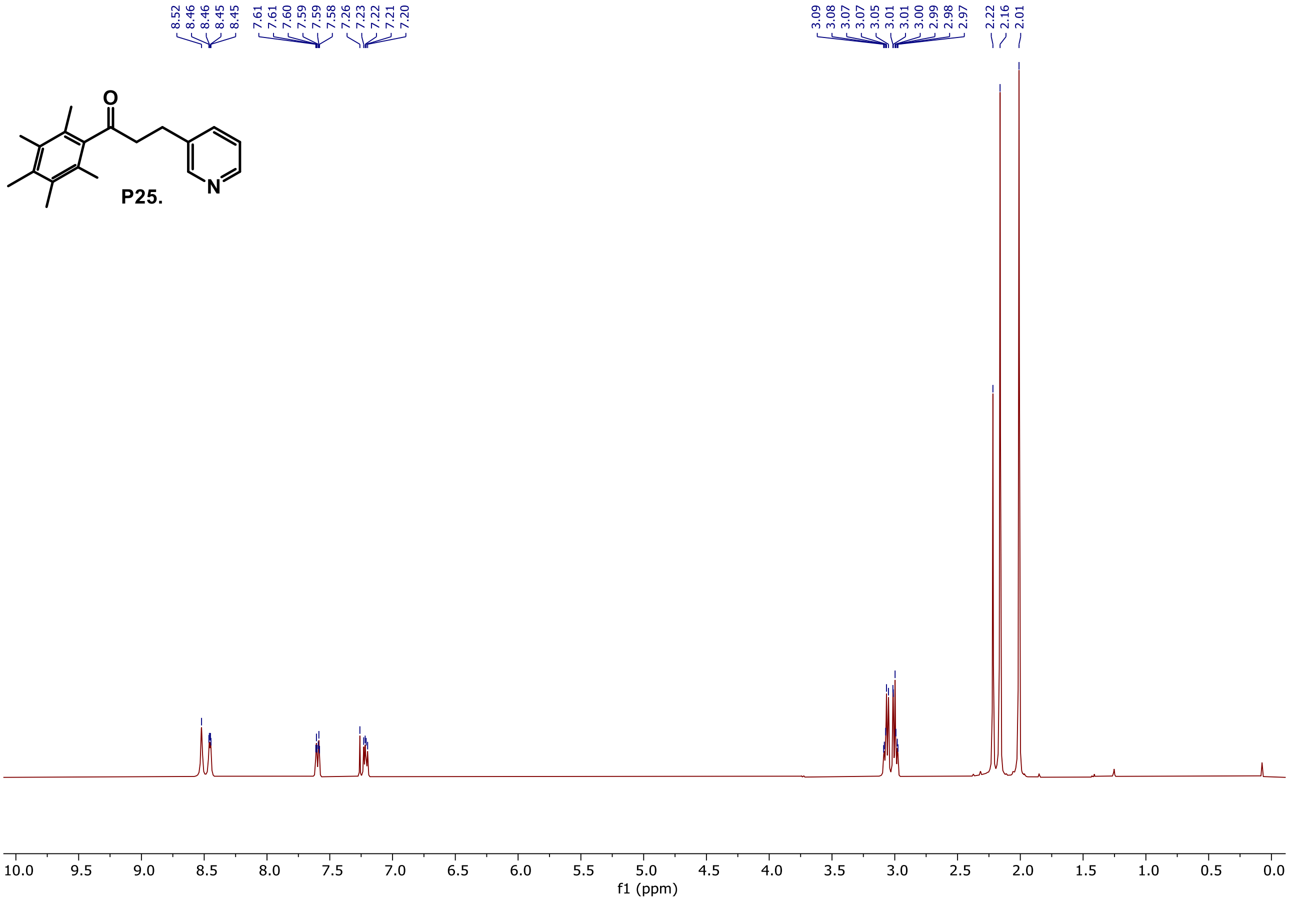

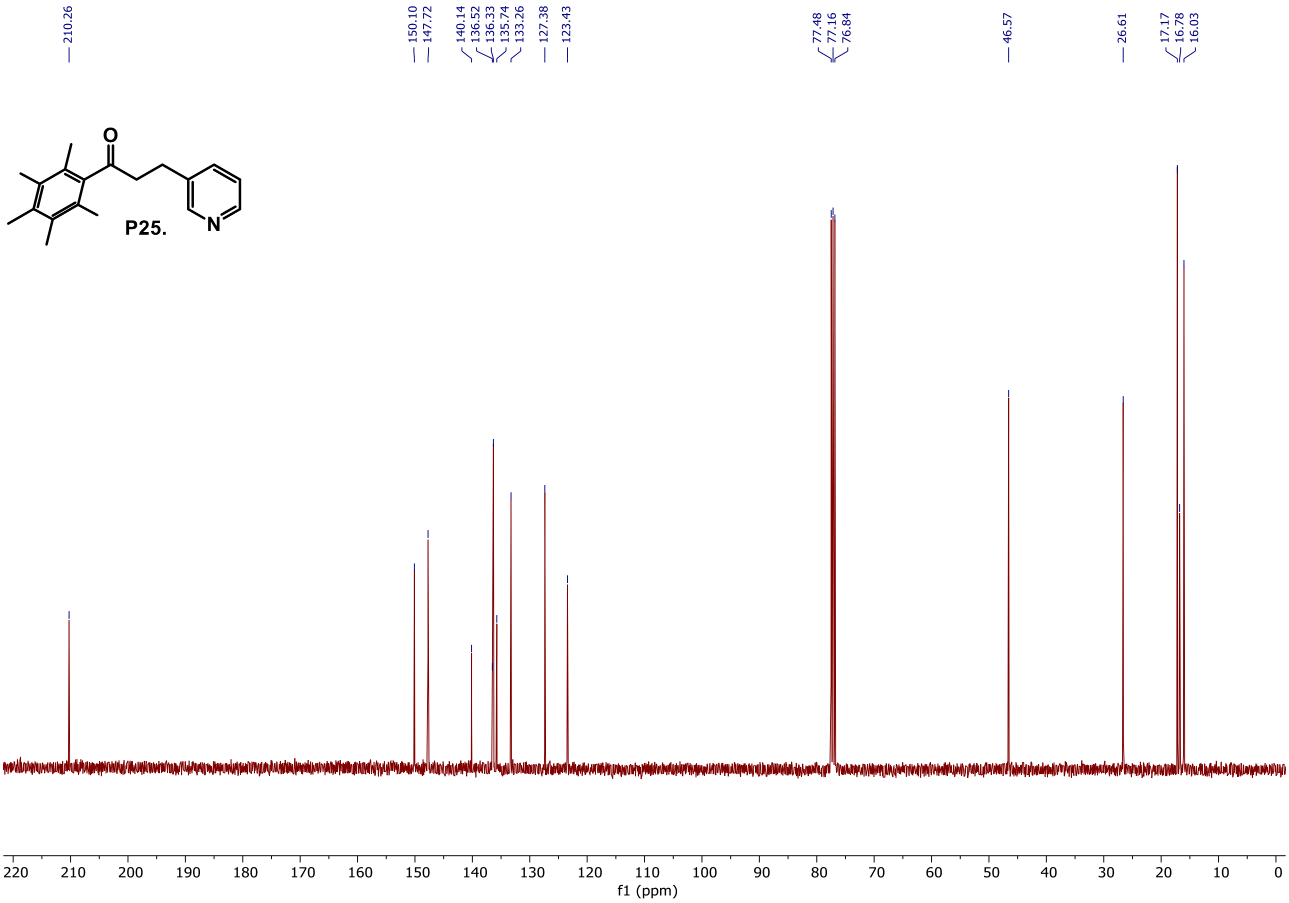

26: ¹H NMR (400 MHz, $CDCl_3$) and **¹³C NMR** (101 MHz, $CDCl_3$ respectively.

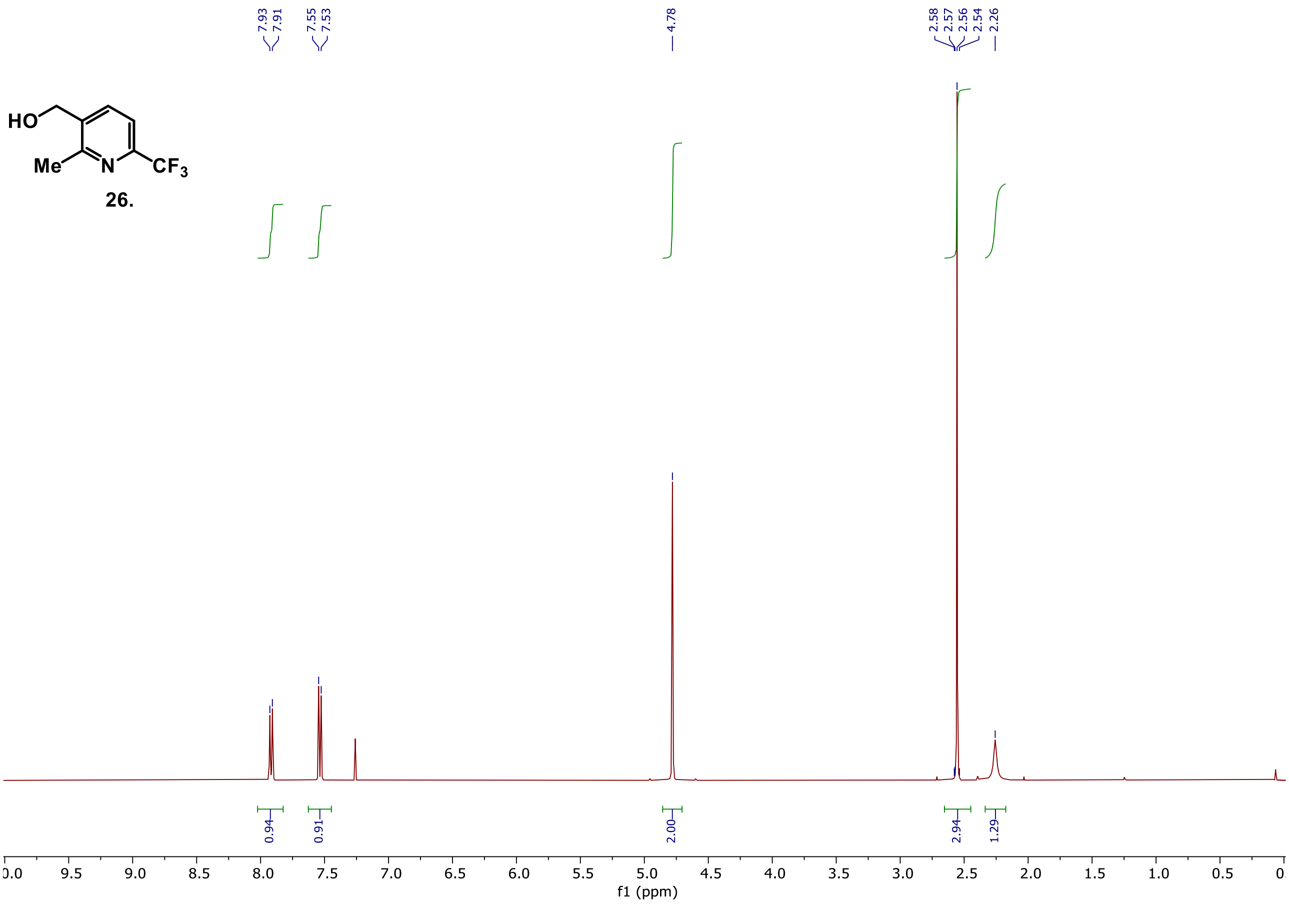

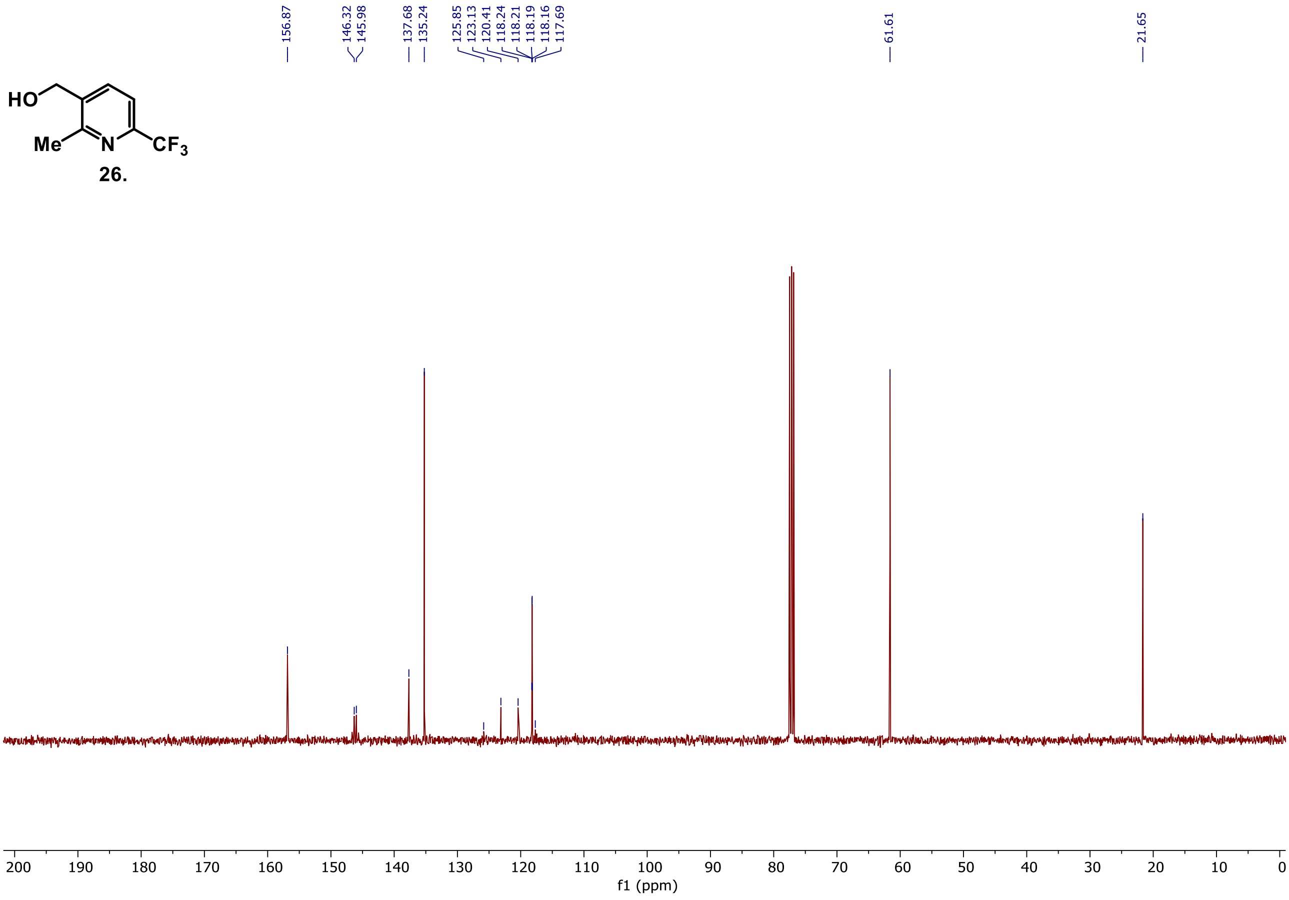

26: ^{19}**F NMR** (376 MHz, $CDCl_3$).

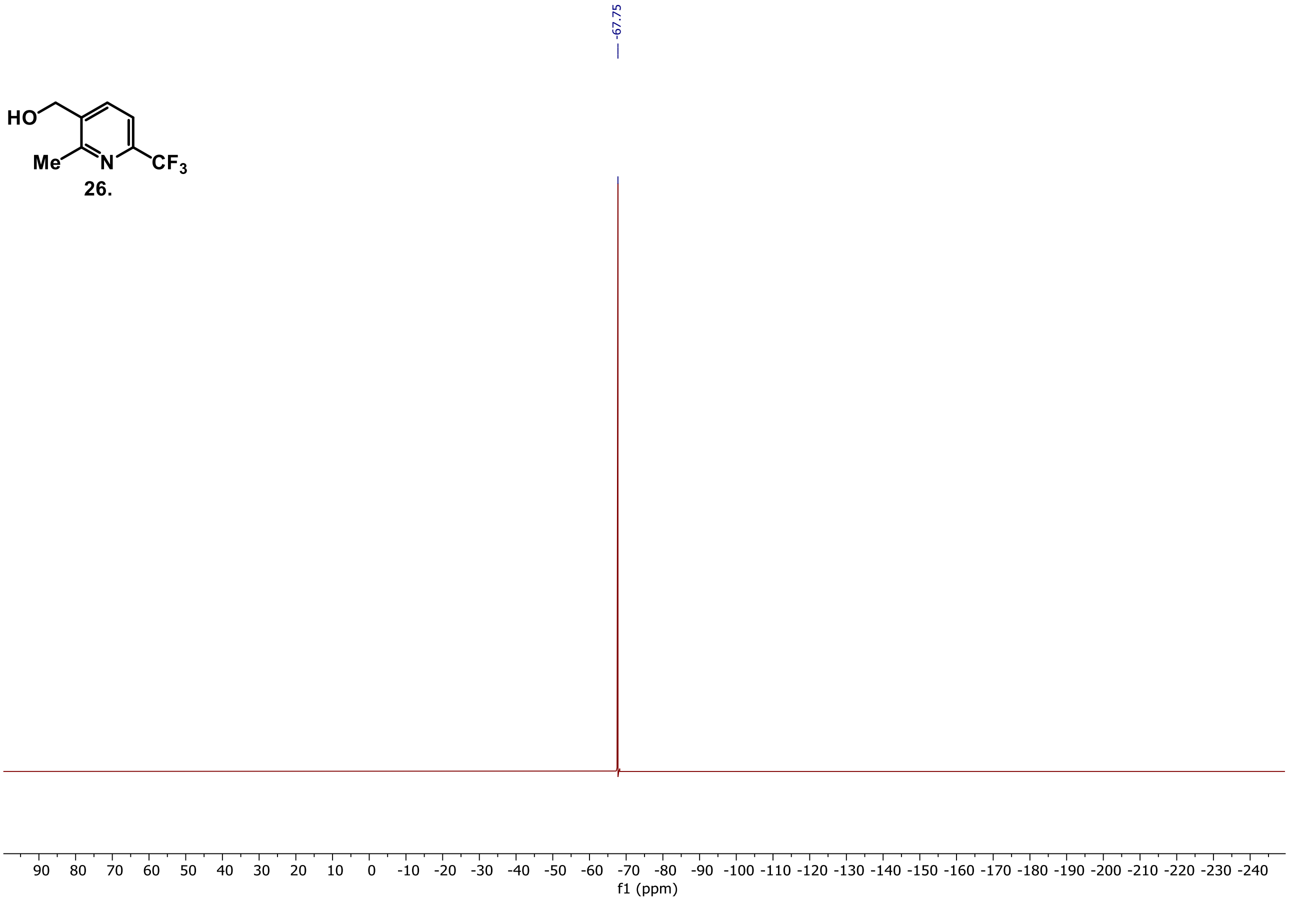

P26: [1]**H NMR** (400 MHz, $CDCl_3$) and [13]**C NMR** (101 MHz, $CDCl_3$) respectively.

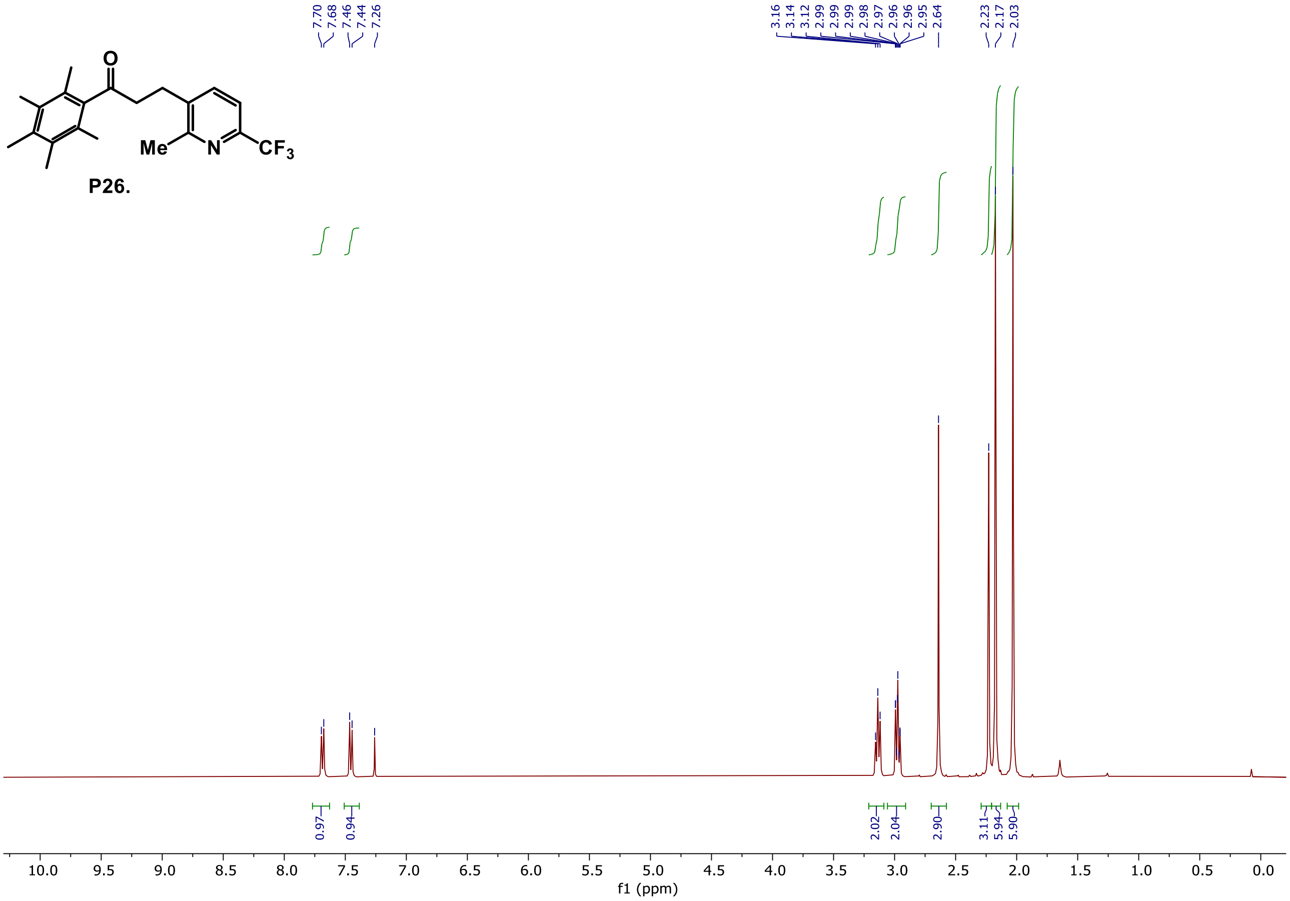

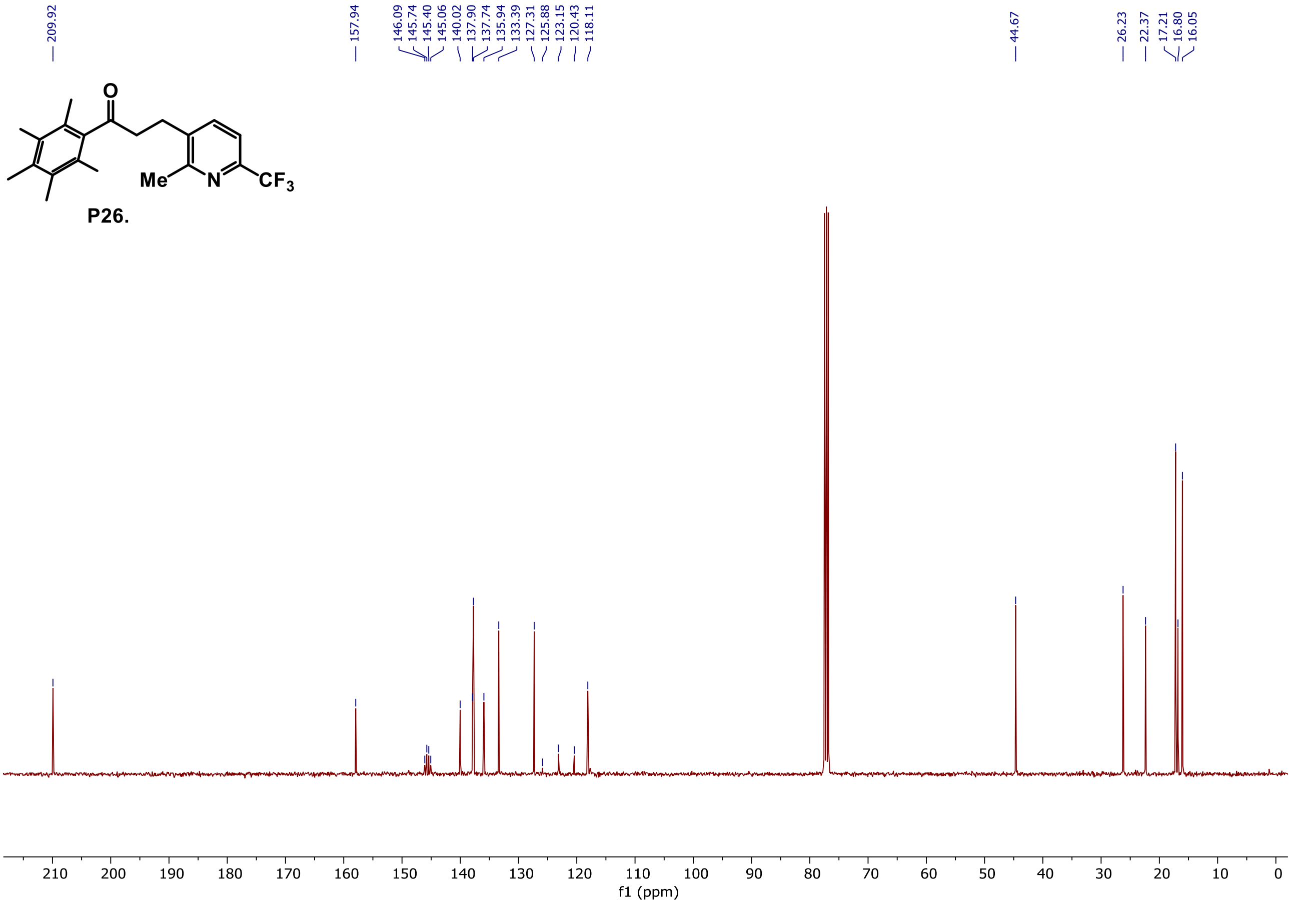

P26: ¹⁹F NMR (376 MHz, $CDCl_3$).

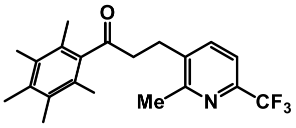

P26.

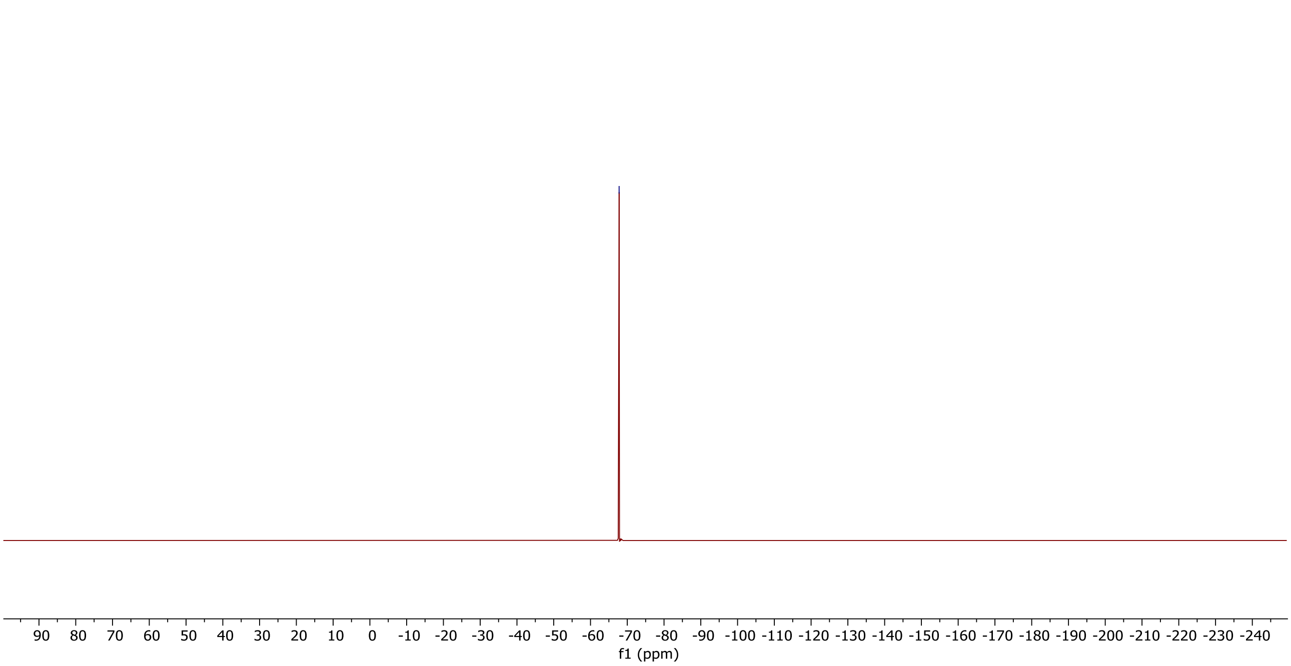

P35: ^{1}**H NMR** (400 MHz, $CDCl_3$) and ^{13}**C NMR** (101 MHz, $CDCl_3$) respectively.

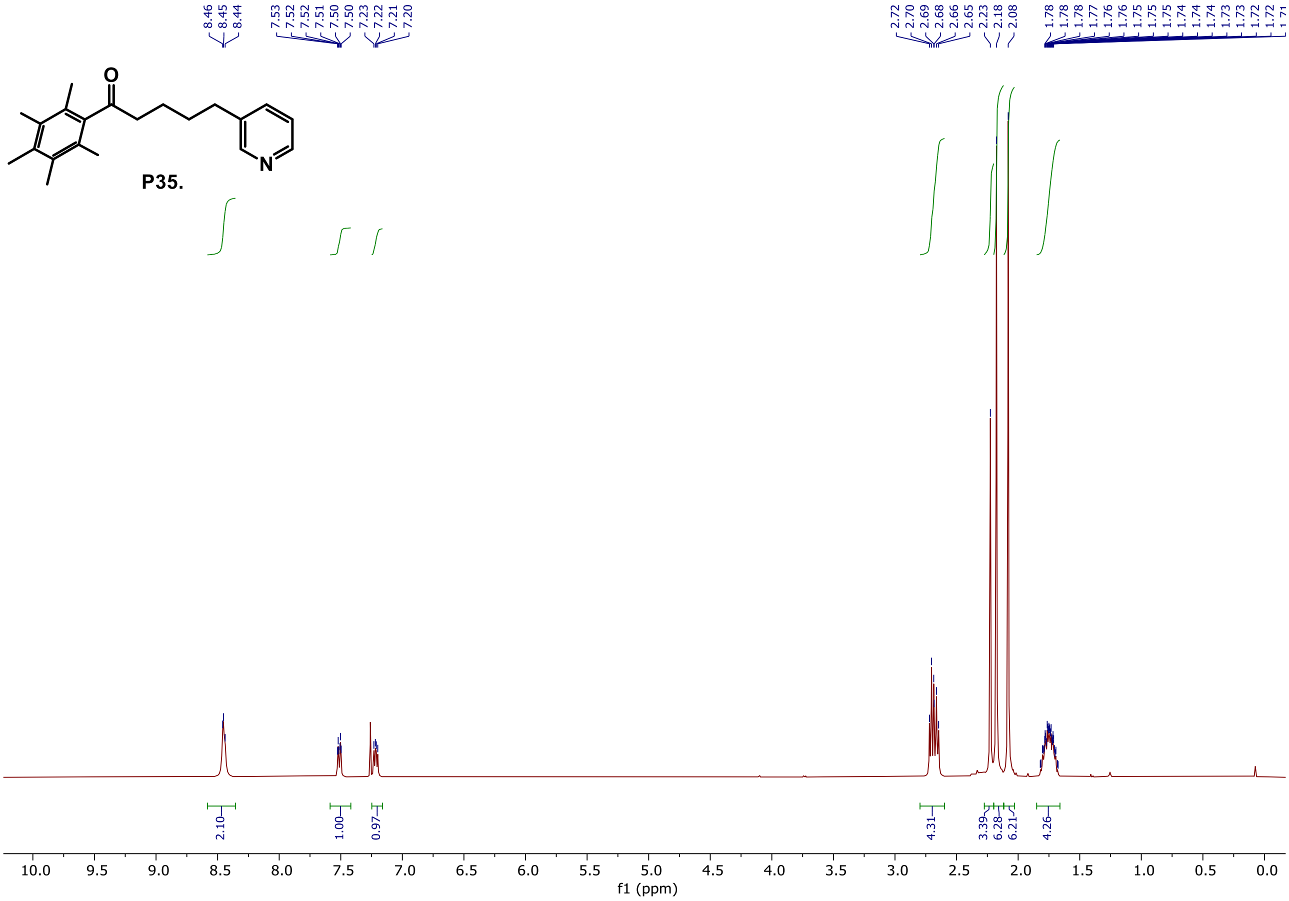

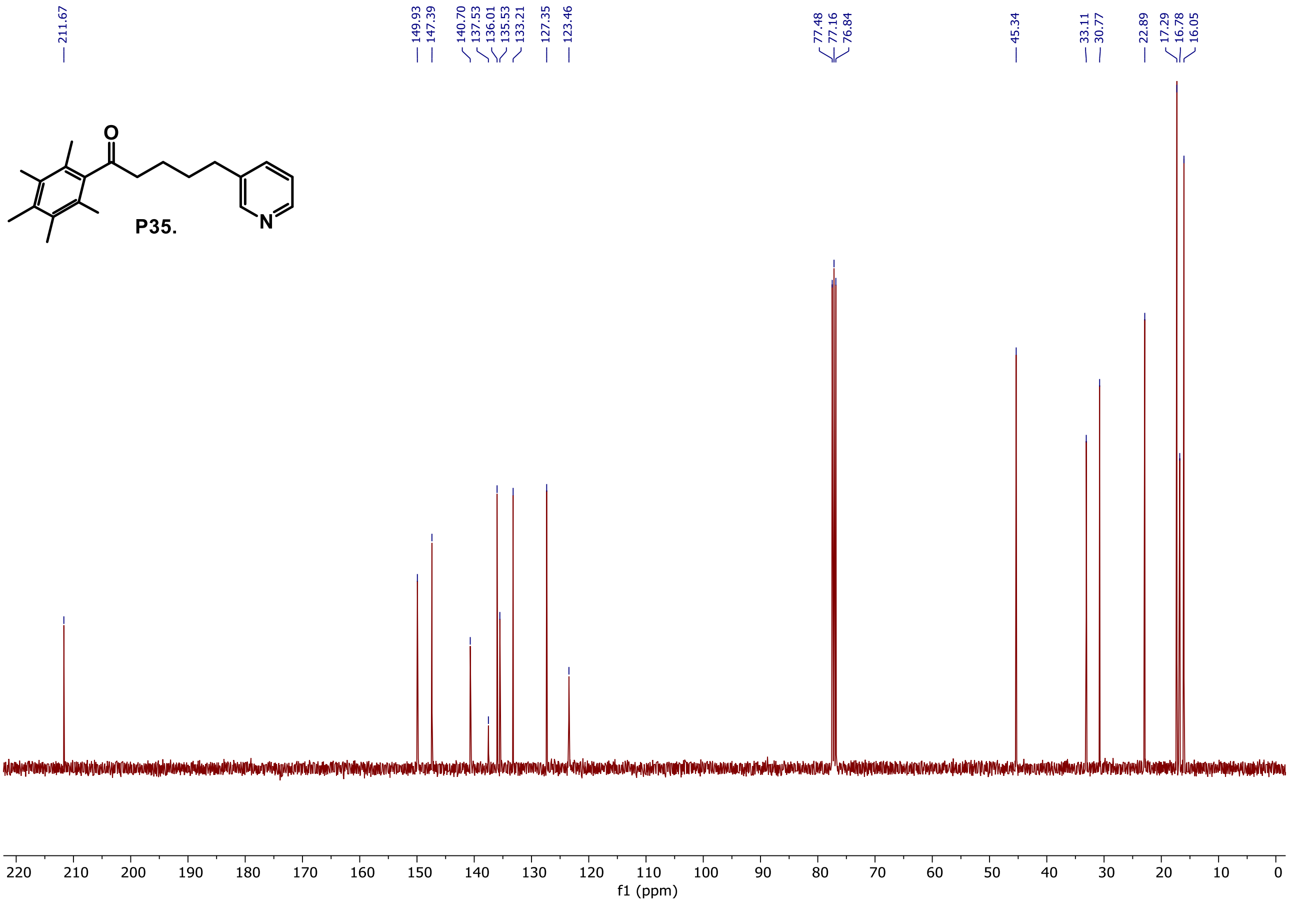

36: ^{1}H NMR (400 MHz, $CDCl_3$) and **^{13}C NMR** (101 MHz, $CDCl_3$) respectively.

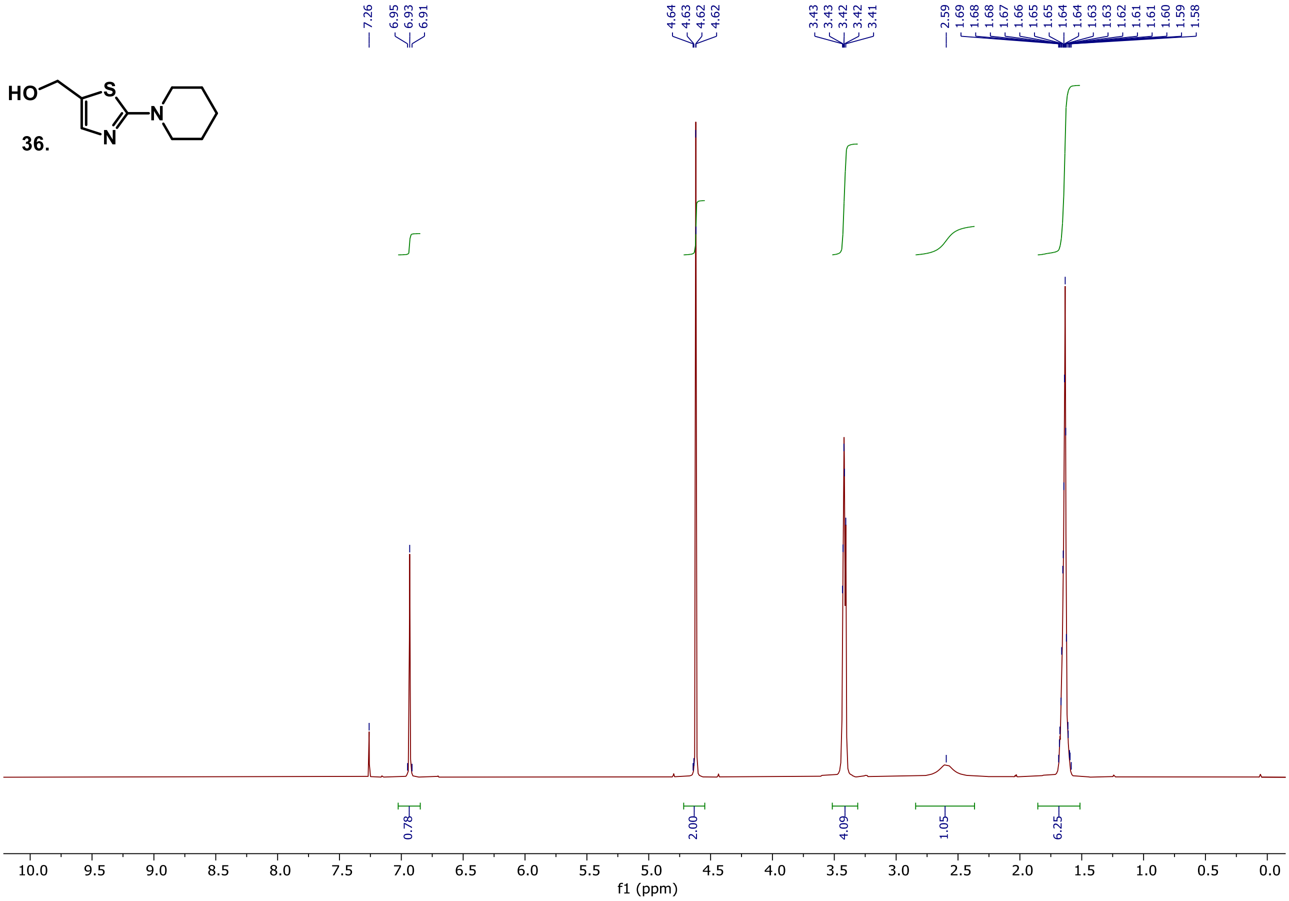

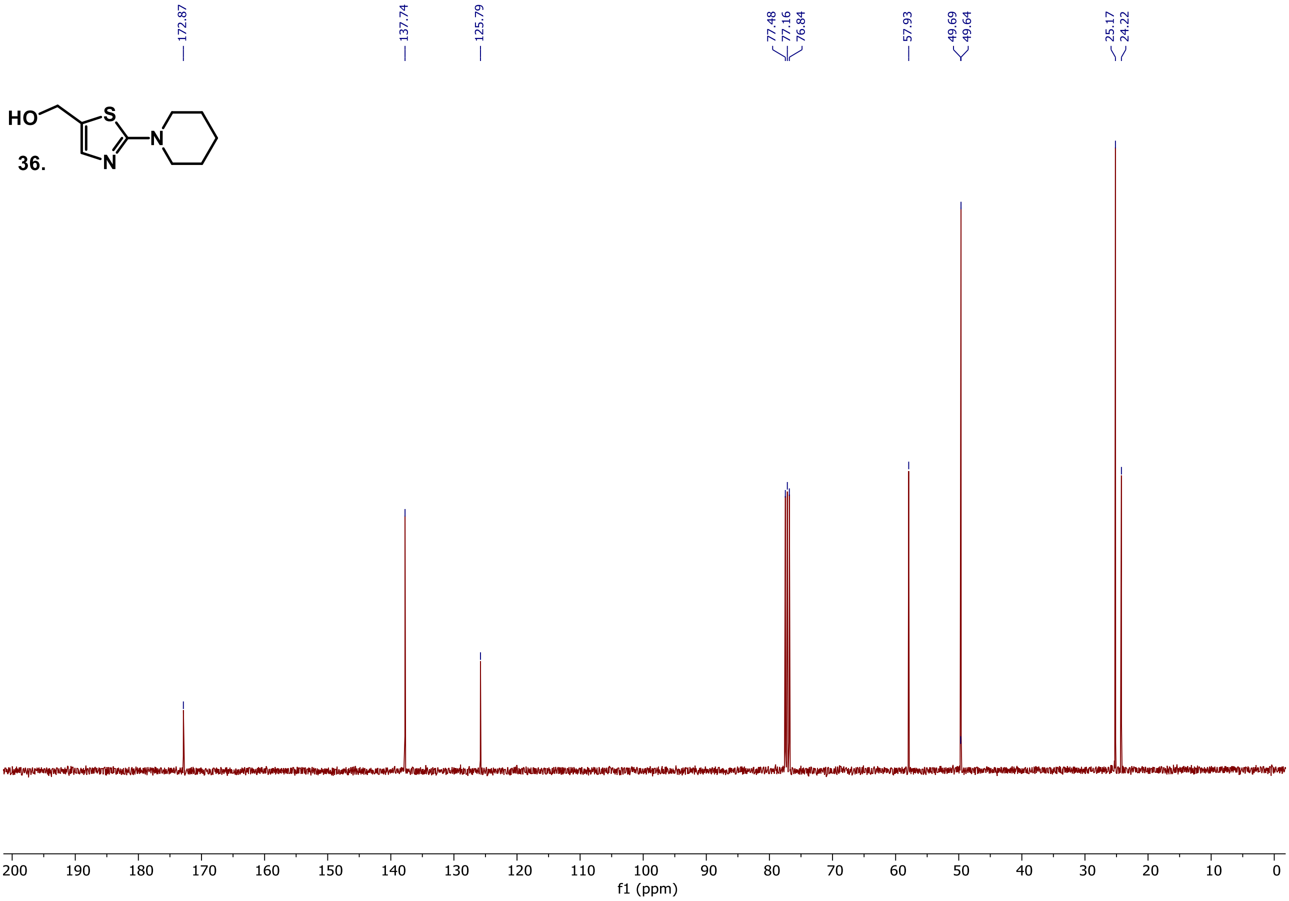

P36: ^{1}**H NMR** (400 MHz, $CDCl_3$) and ^{13}**C NMR** (101 MHz, $CDCl_3$) respectively.

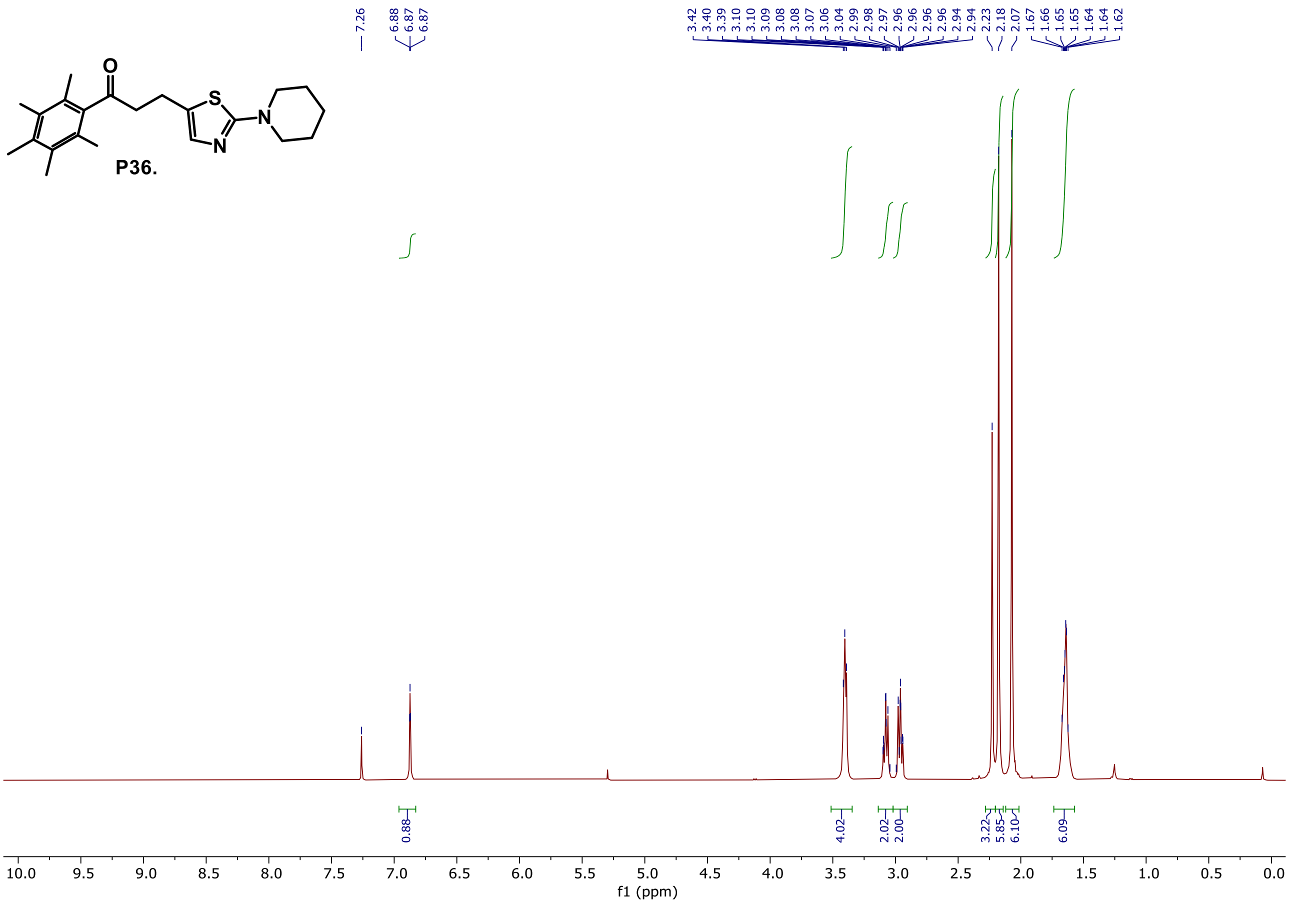

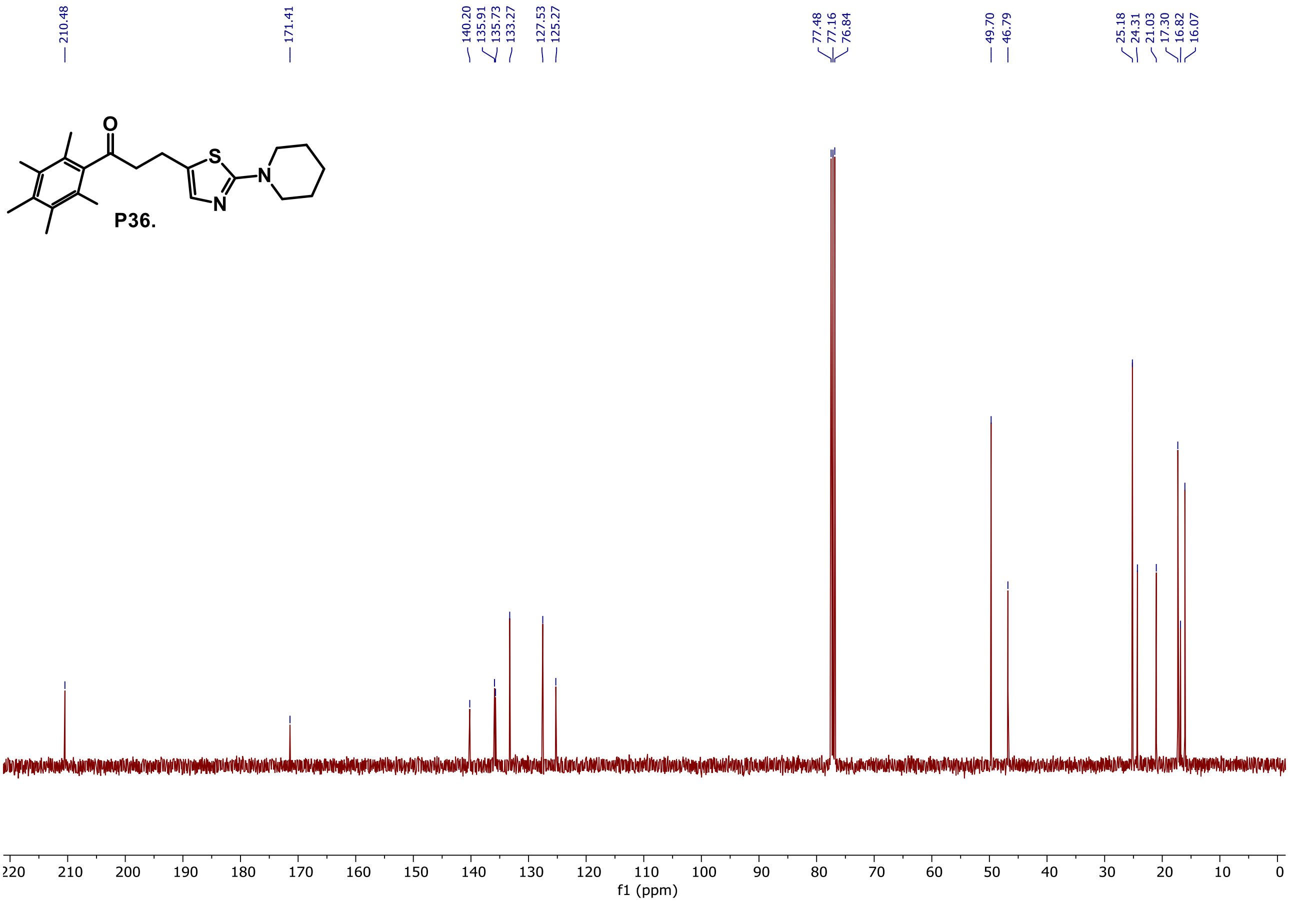

37: **^{1}H NMR** (400 MHz, $CDCl_3$) and **^{13}C NMR** (101 MHz, $CDCl_3$) respectively.

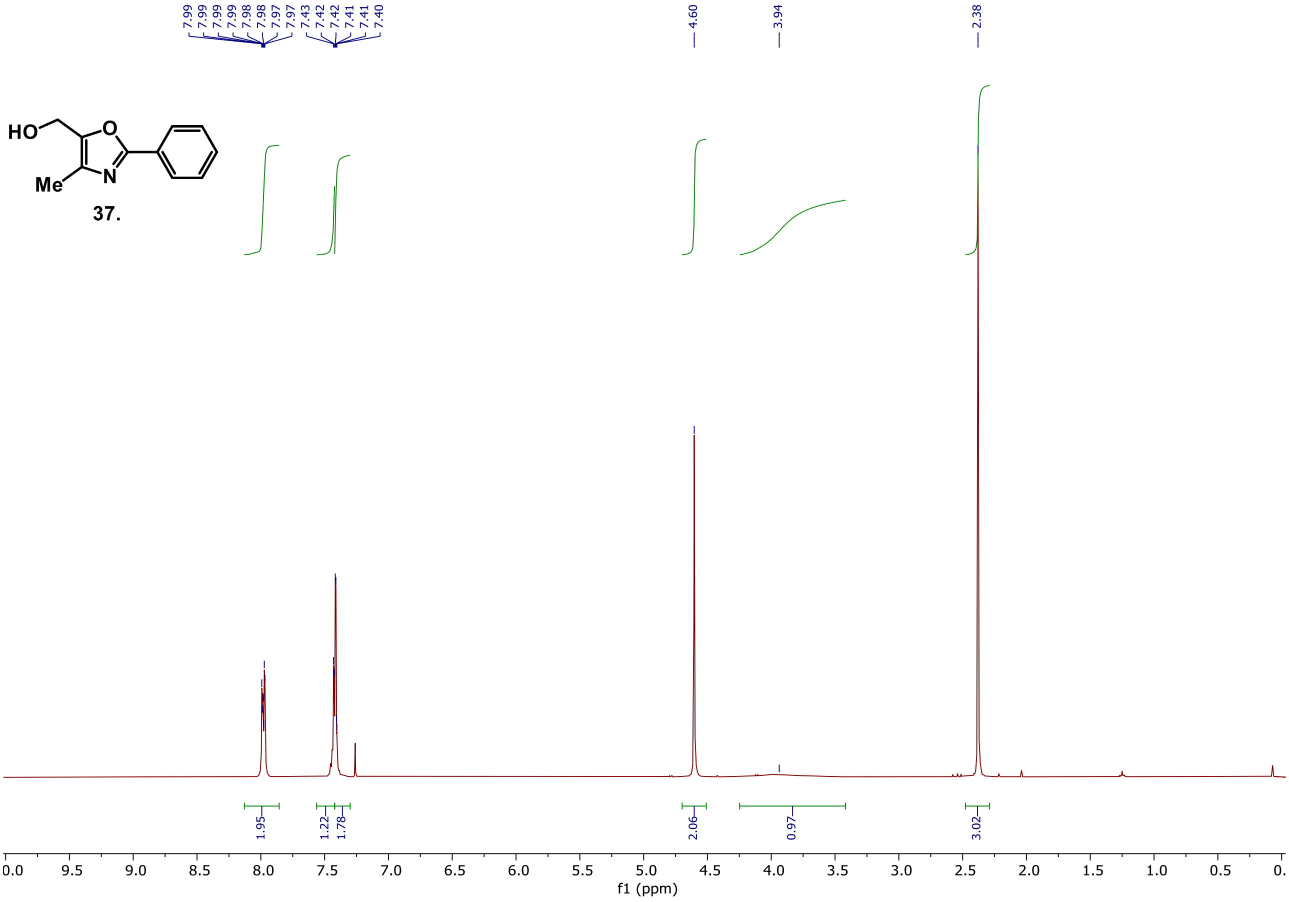

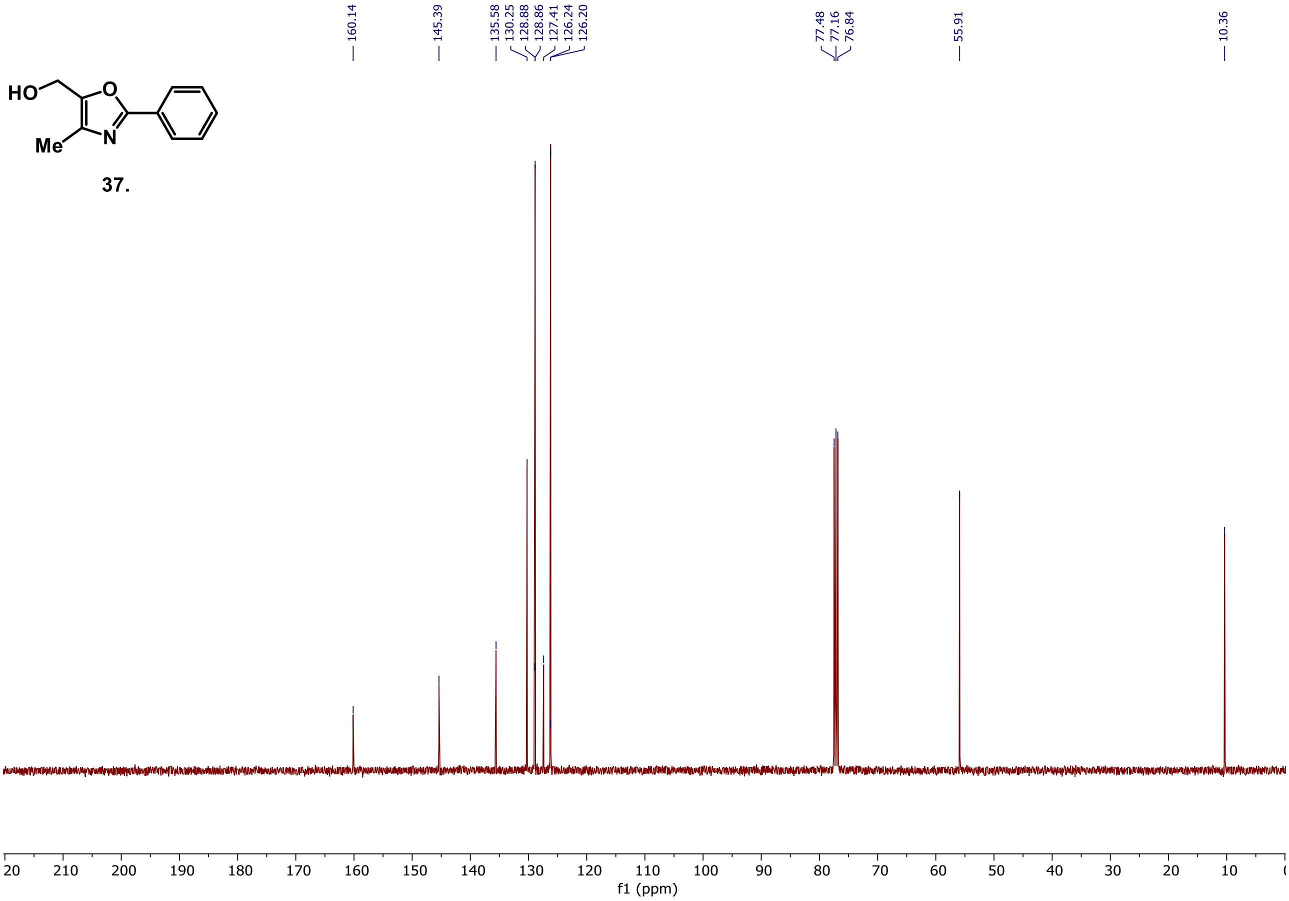

P37: ^{1}**H NMR** (400 MHz, $CDCl_3$) and ^{13}**C NMR** (101 MHz, $CDCl_3$) respectively.

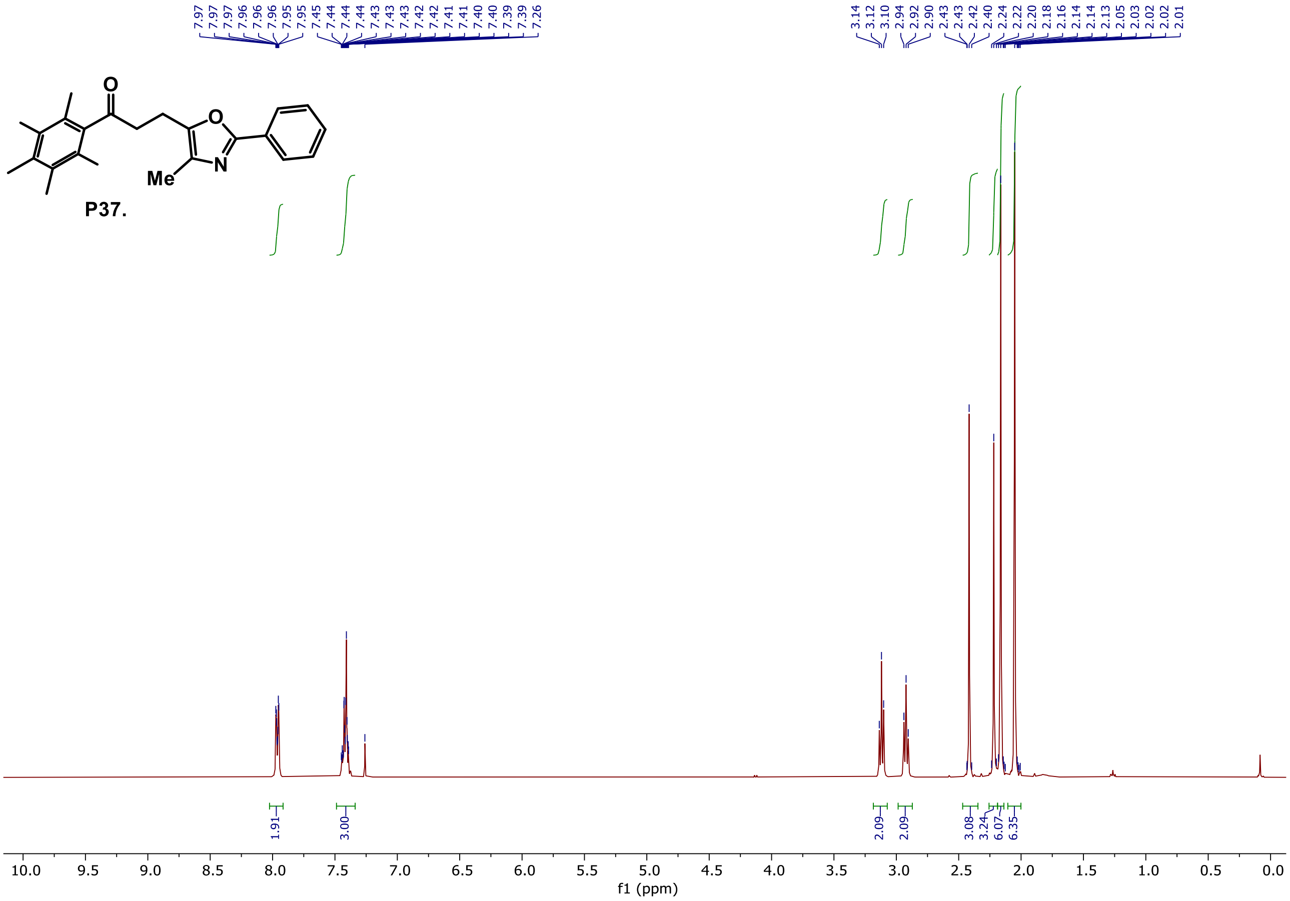

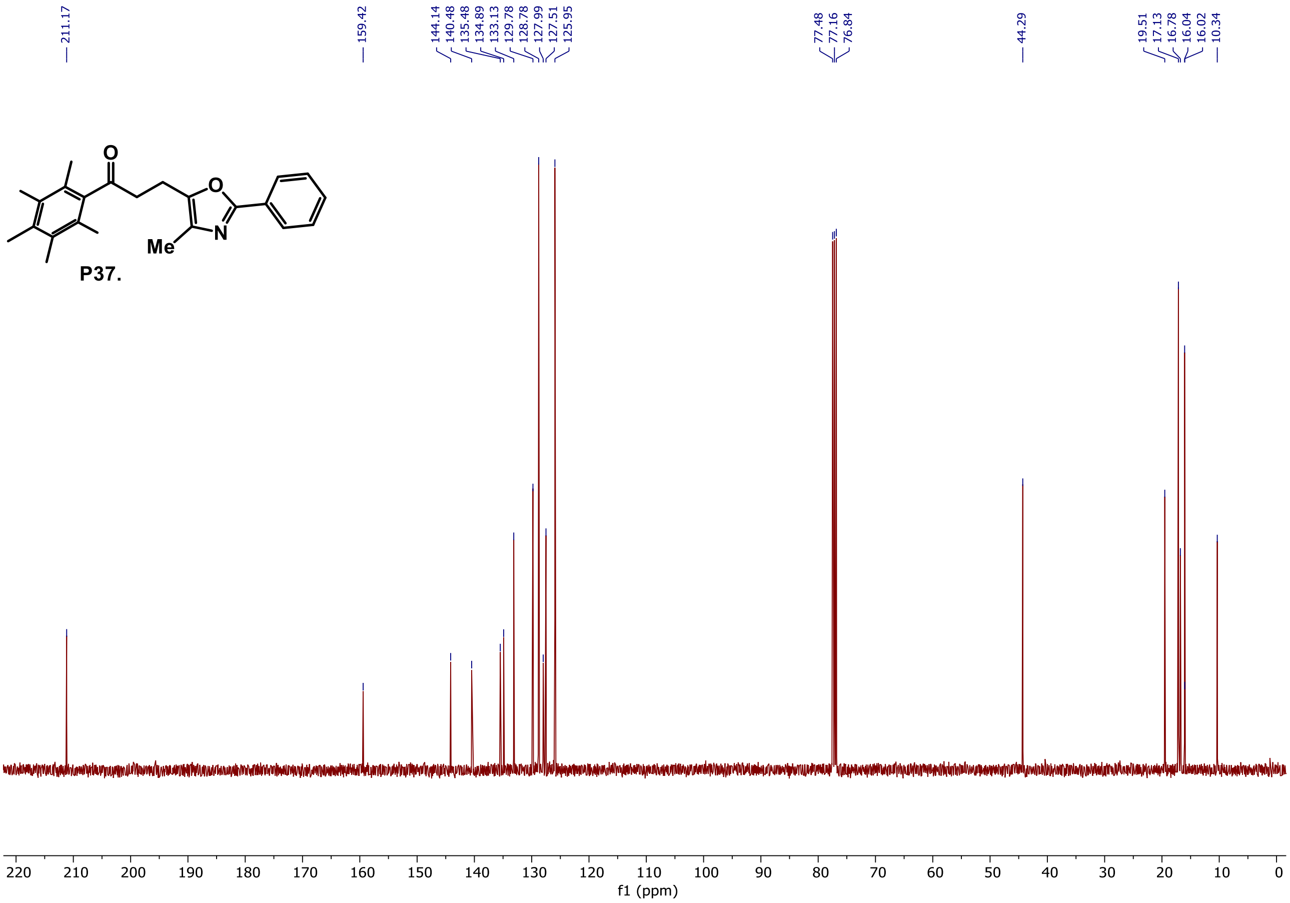

38: **¹H NMR** (400 MHz, $CDCl_3$) and **¹³C NMR** (101 MHz, $CDCl_3$) respectively.

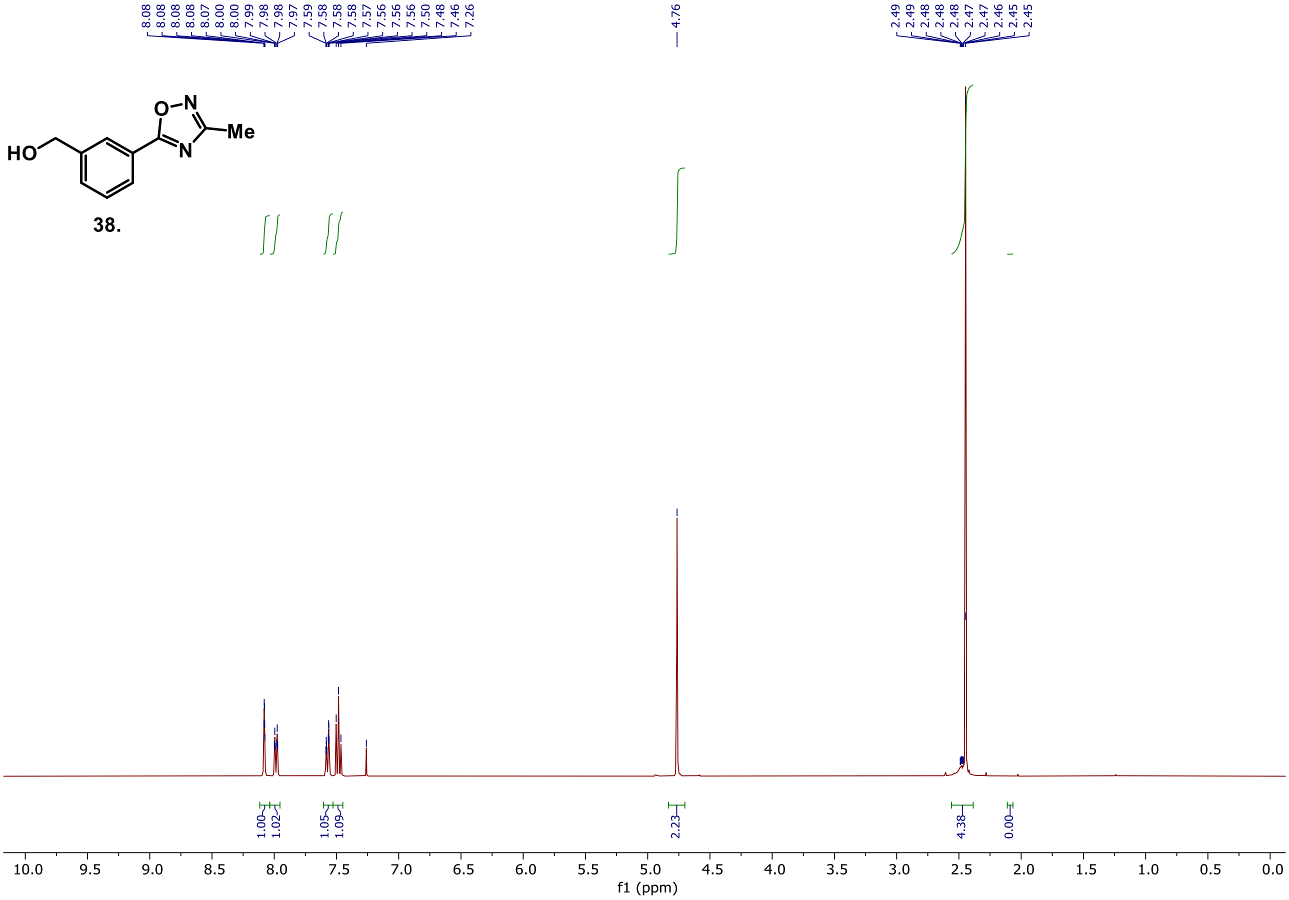

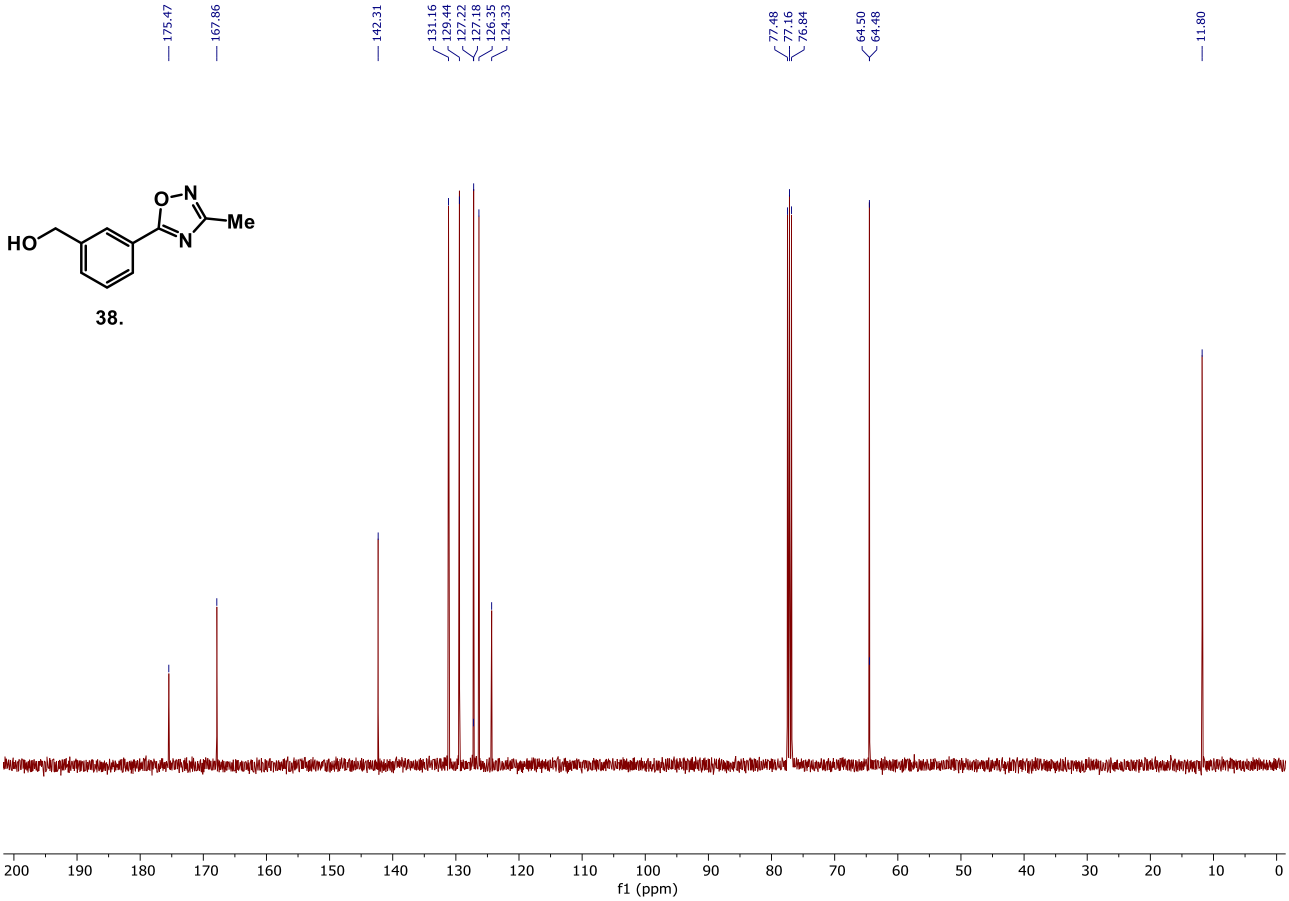

P38: ^{1}**H NMR** (400 MHz, $CDCl_3$) and ^{13}**C NMR** (101 MHz, $CDCl_3$) respectively.

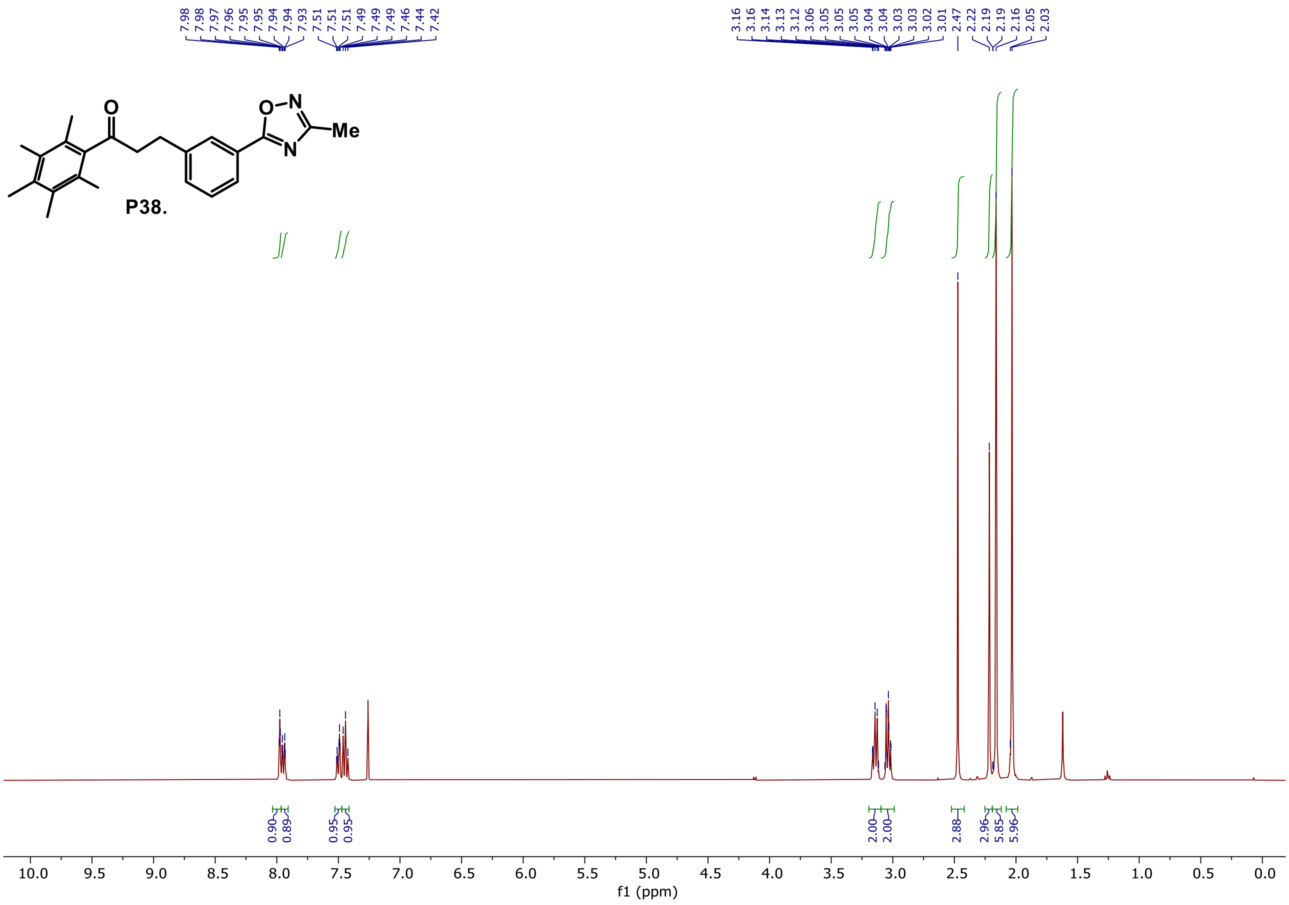

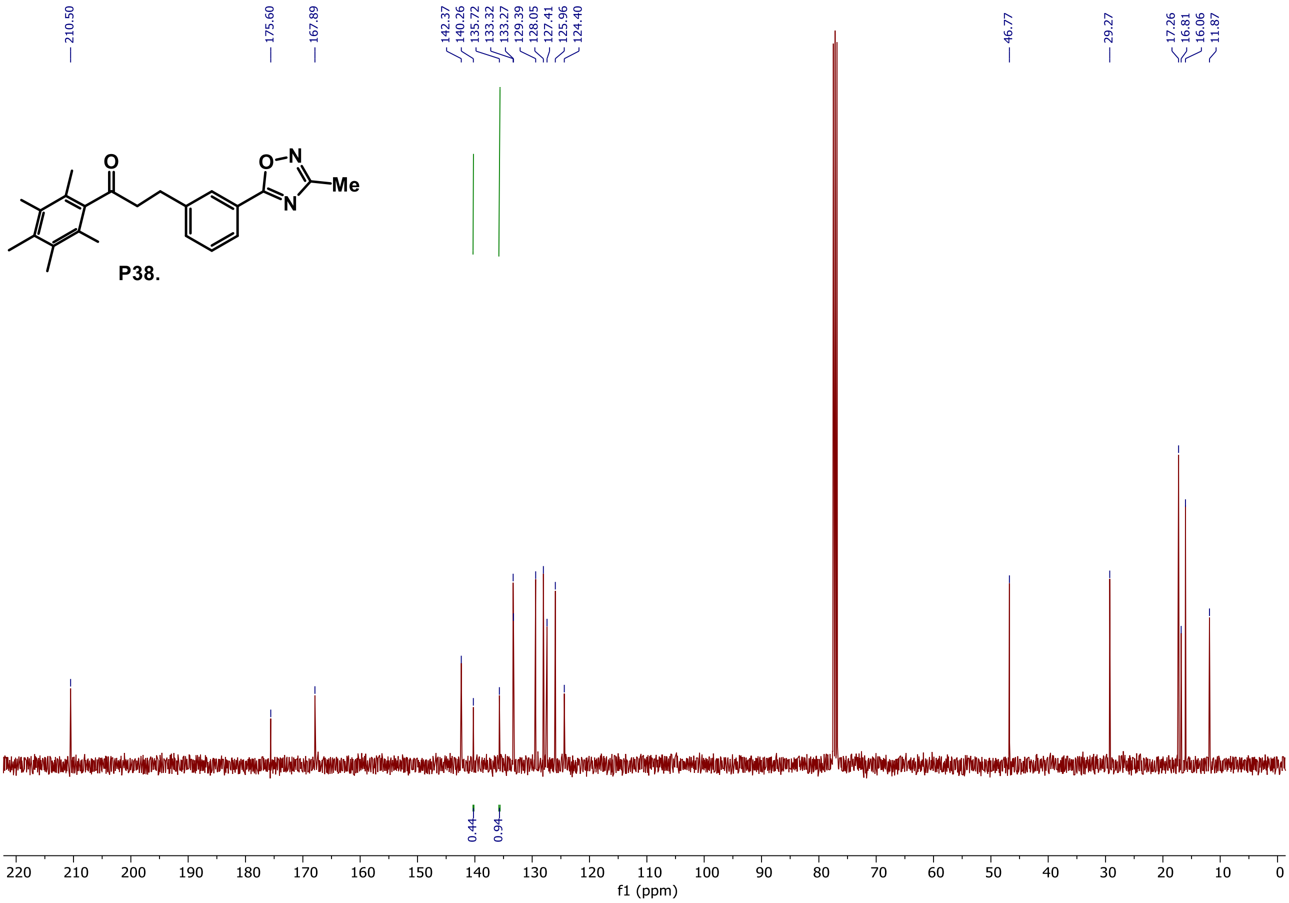

39: **^{1}H NMR** (400 MHz, $CDCl_3$) and **^{13}C NMR** (101 MHz, $CDCl_3$) respectively.

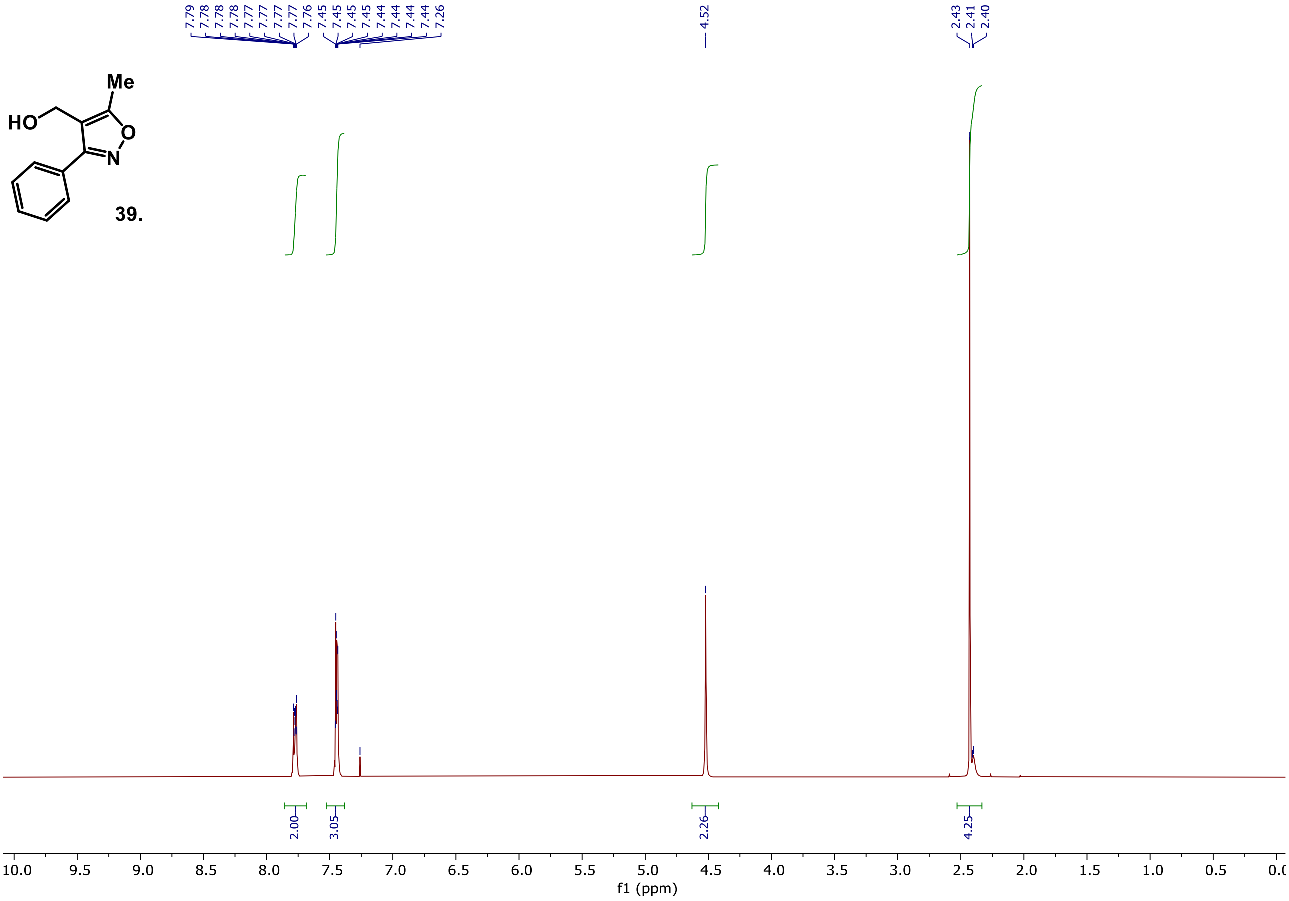

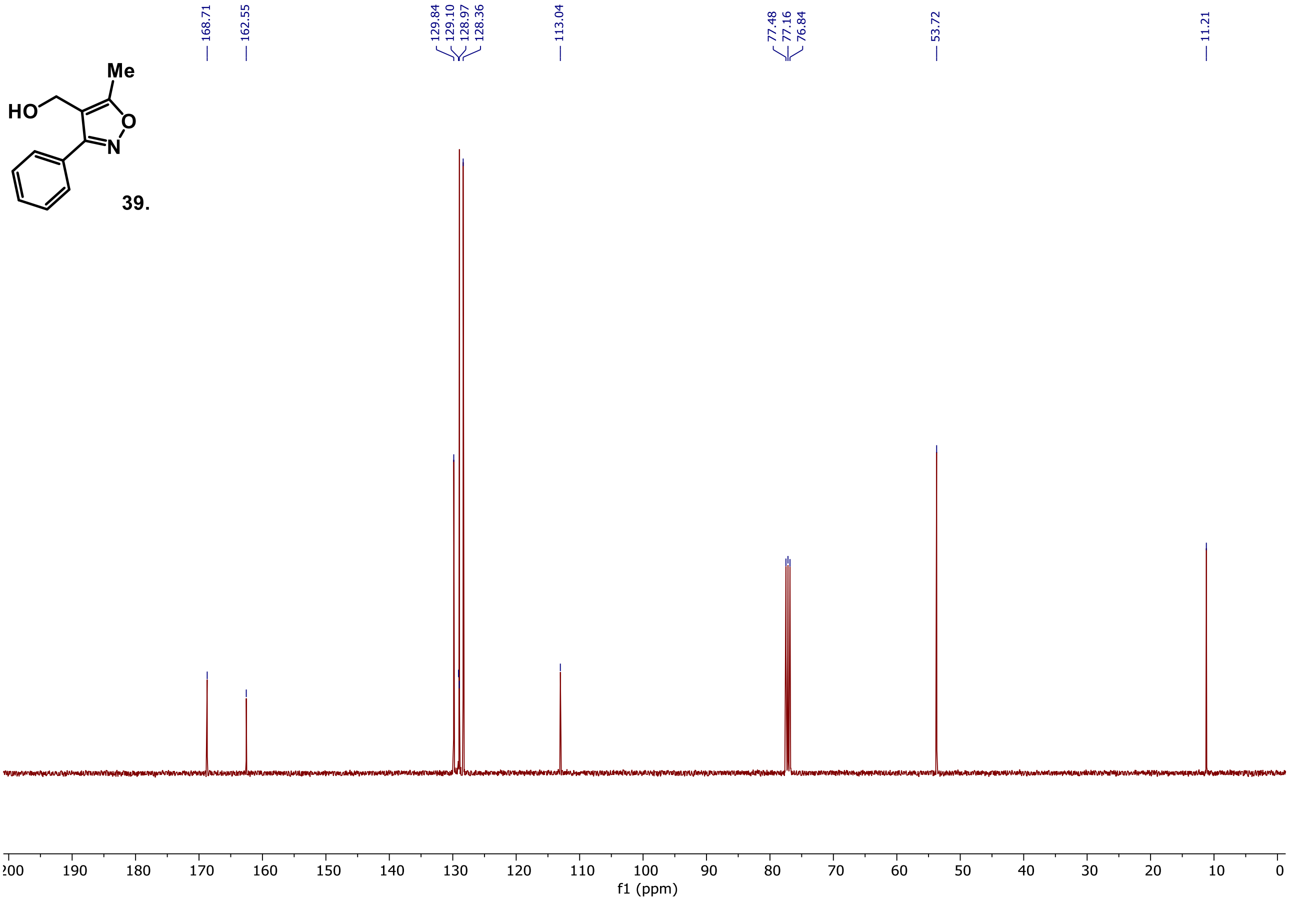

P39: ^{1}H NMR (400 MHz, $CDCl_3$) and **^{13}C NMR** (101 MHz, $CDCl_3$) respectively.

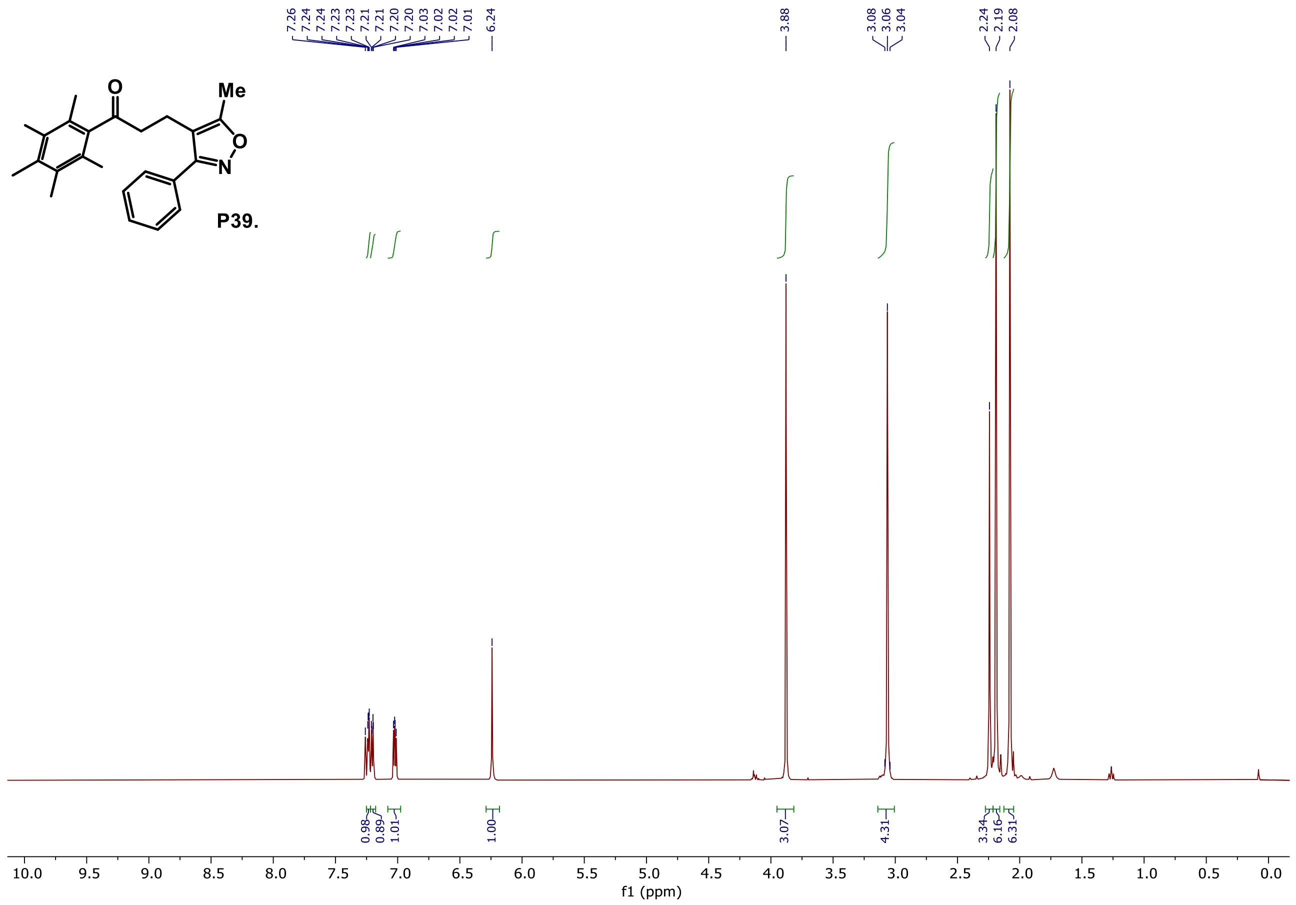

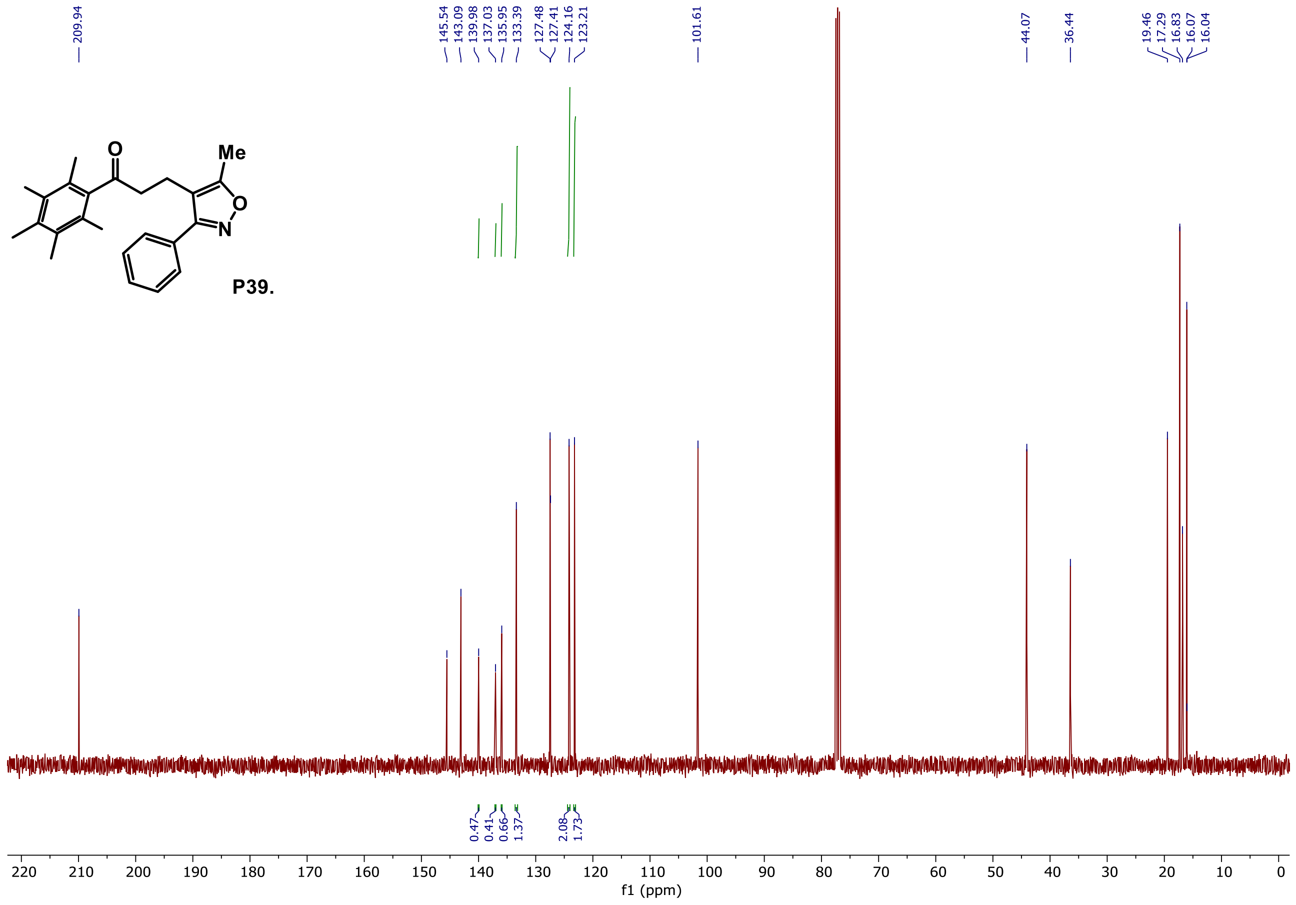

40: ¹H NMR (400 MHz, $CDCl_3$) and **¹³C NMR** (101 MHz, $CDCl_3$) respectively.

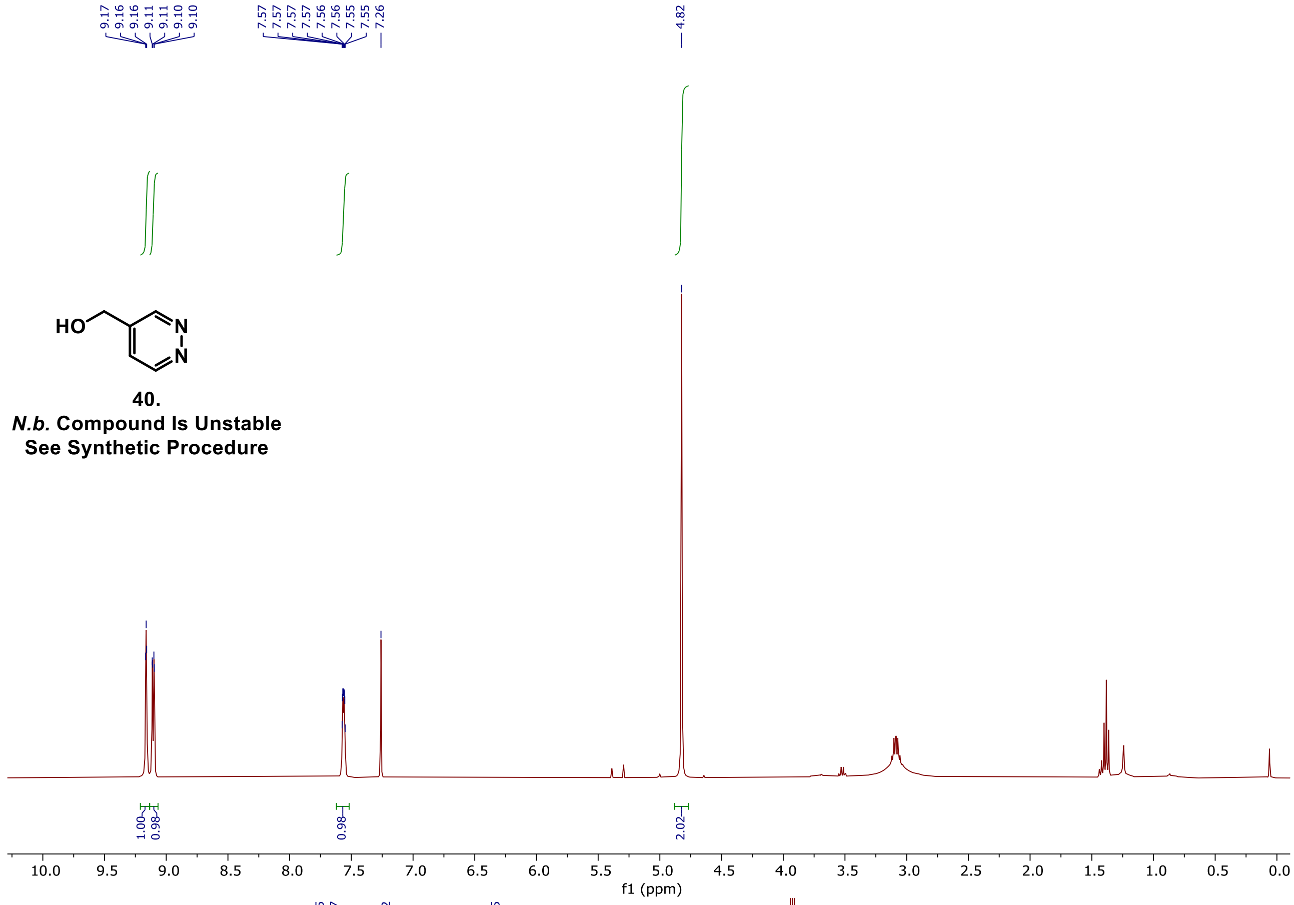

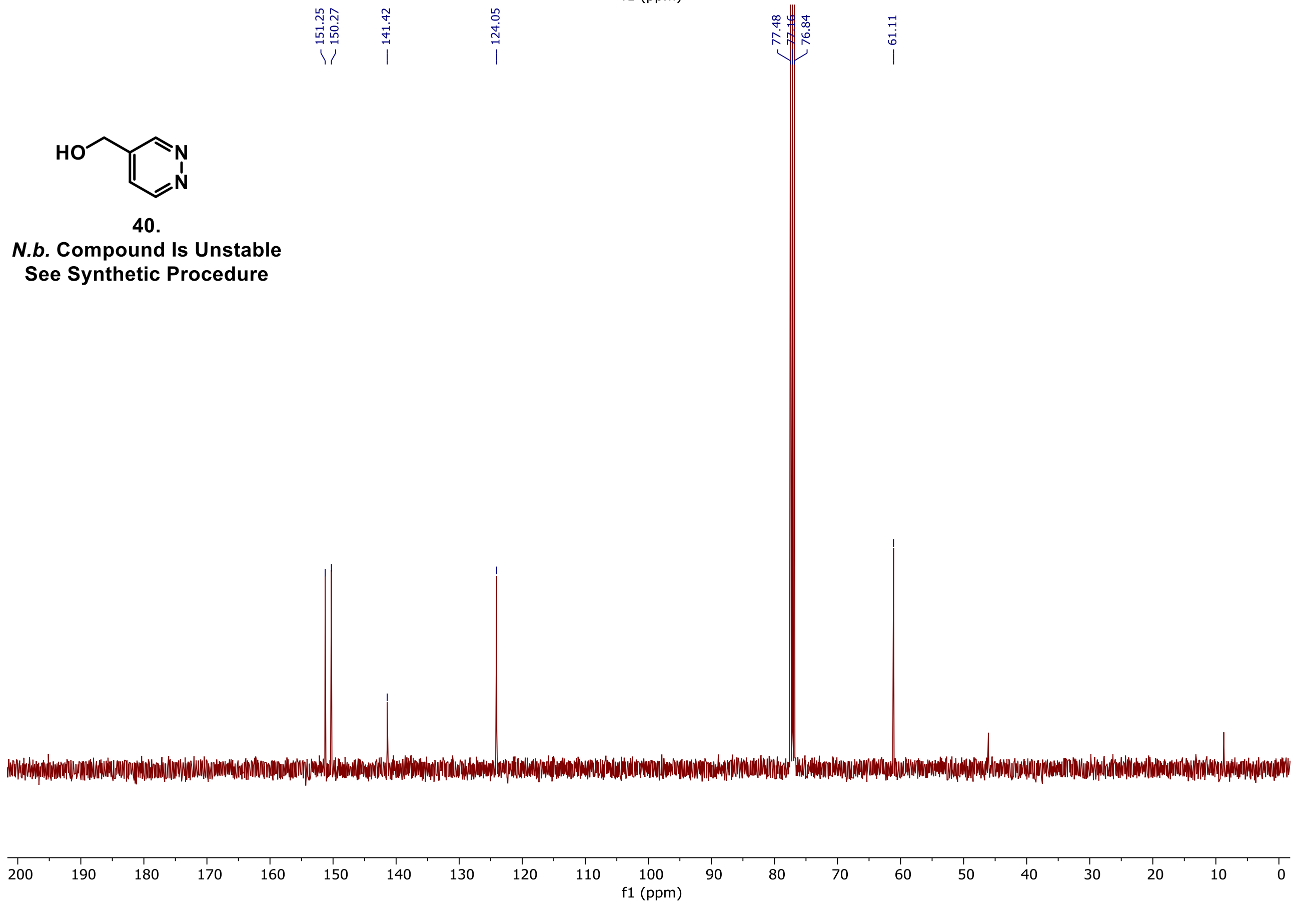

P40: **^{1}H NMR** (400 MHz, $CDCl_3$) and **^{13}C NMR** (101 MHz, $CDCl_3$) respectively.

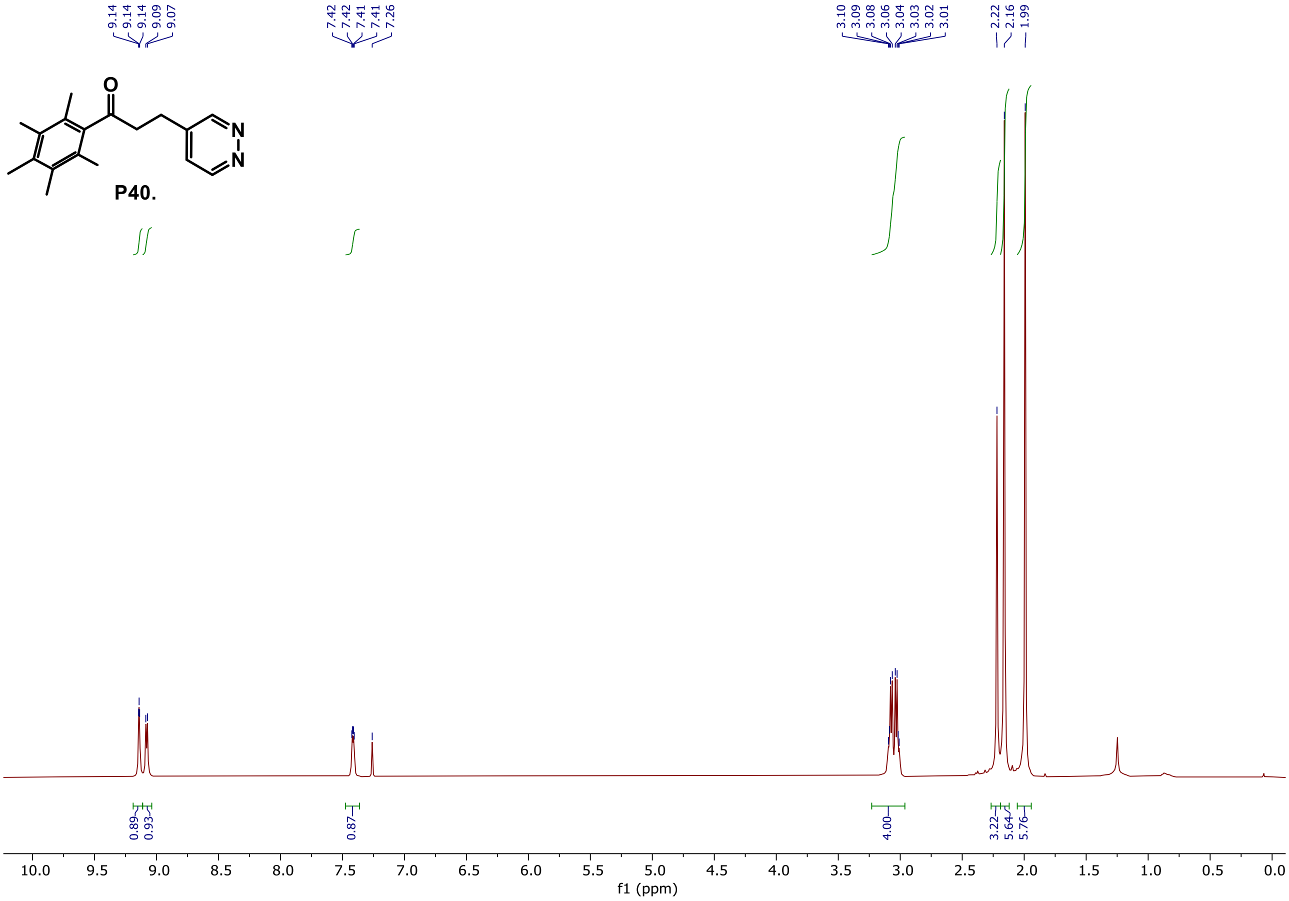

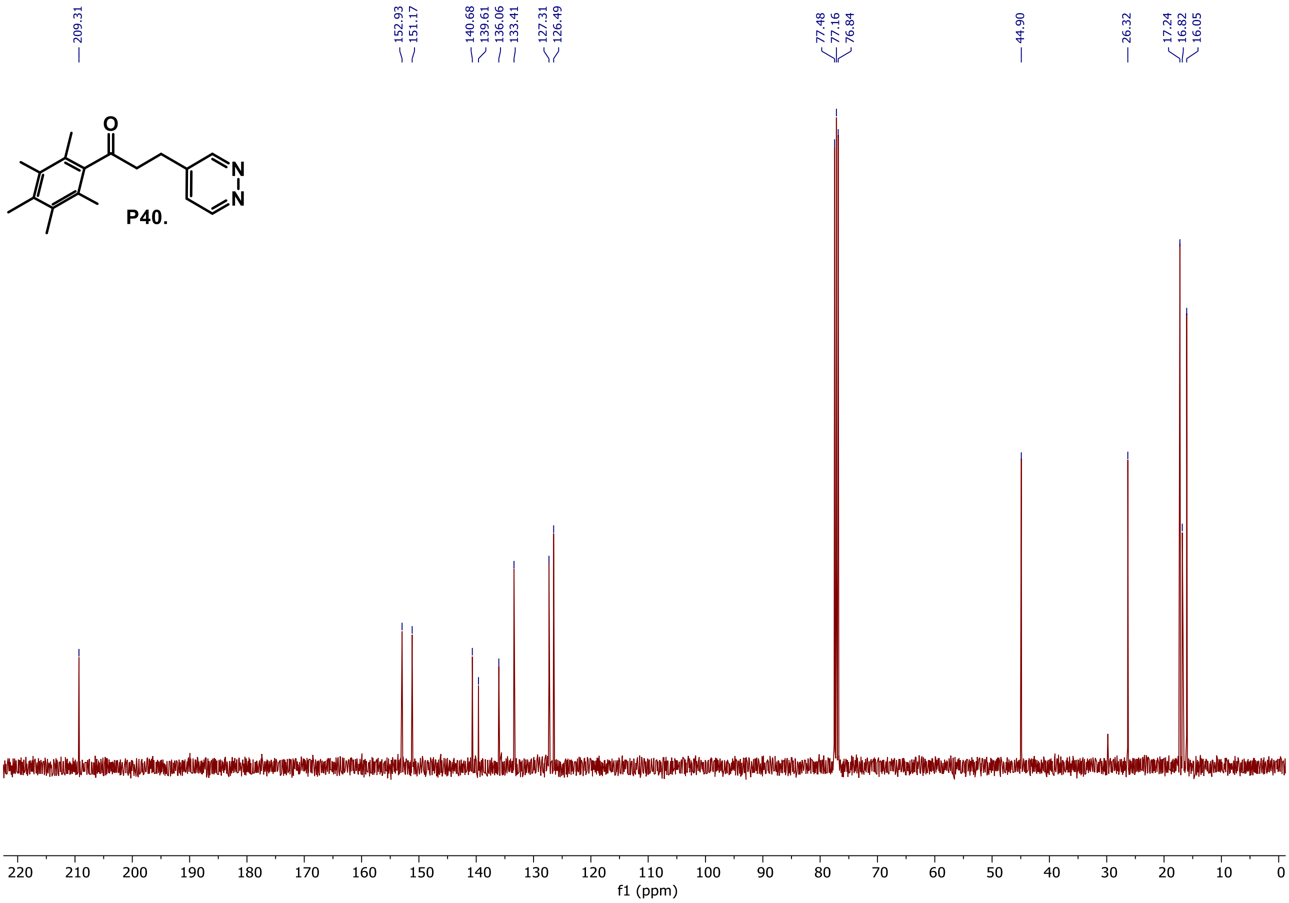

41: ¹H NMR (400 MHz, $CDCl_3$) and **¹³C NMR** (101 MHz, $CDCl_3$) respectively.

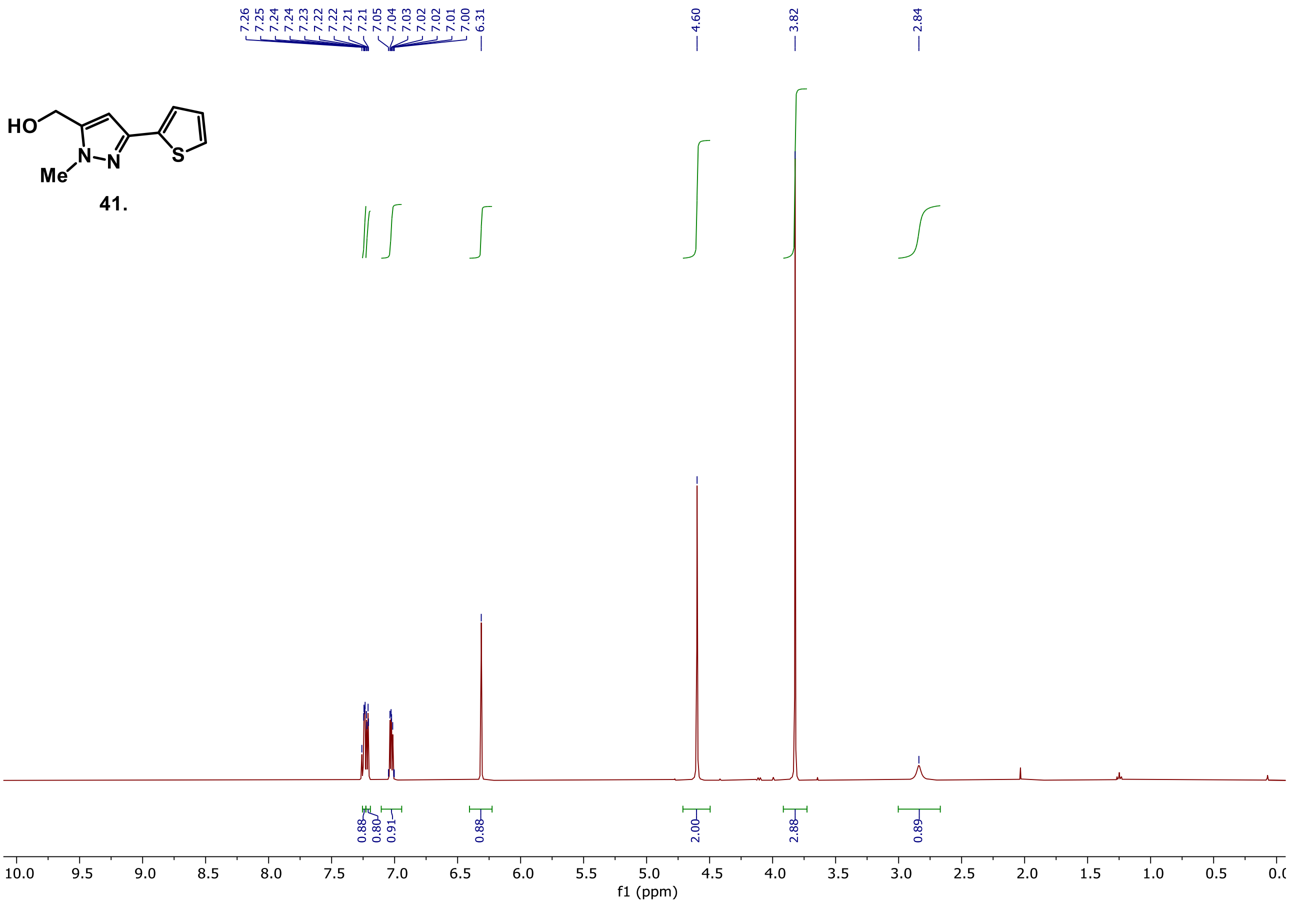

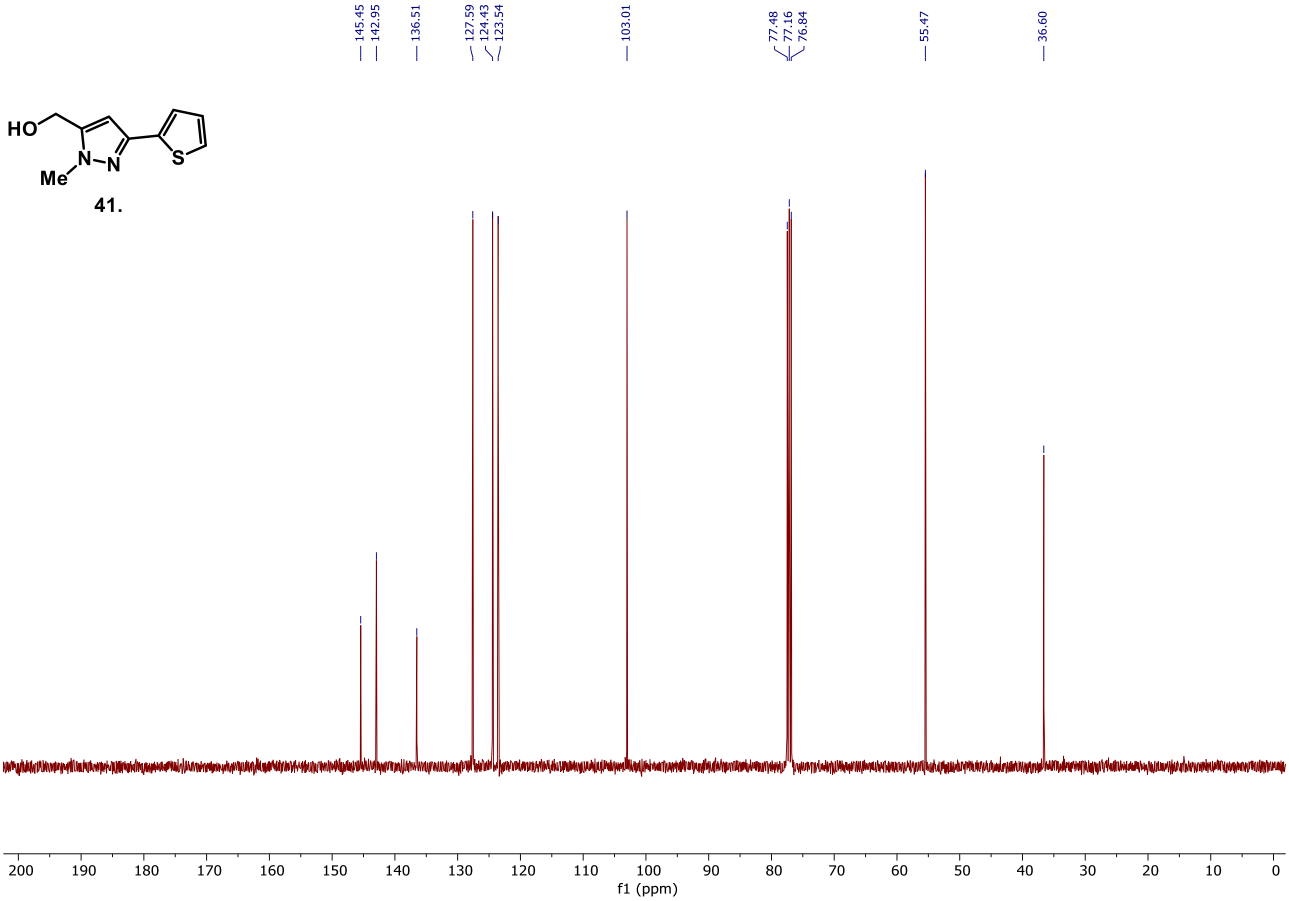

P41: **^{1}H NMR** (400 MHz, $CDCl_3$) and **^{13}C NMR** (101 MHz, $CDCl_3$) respectively.

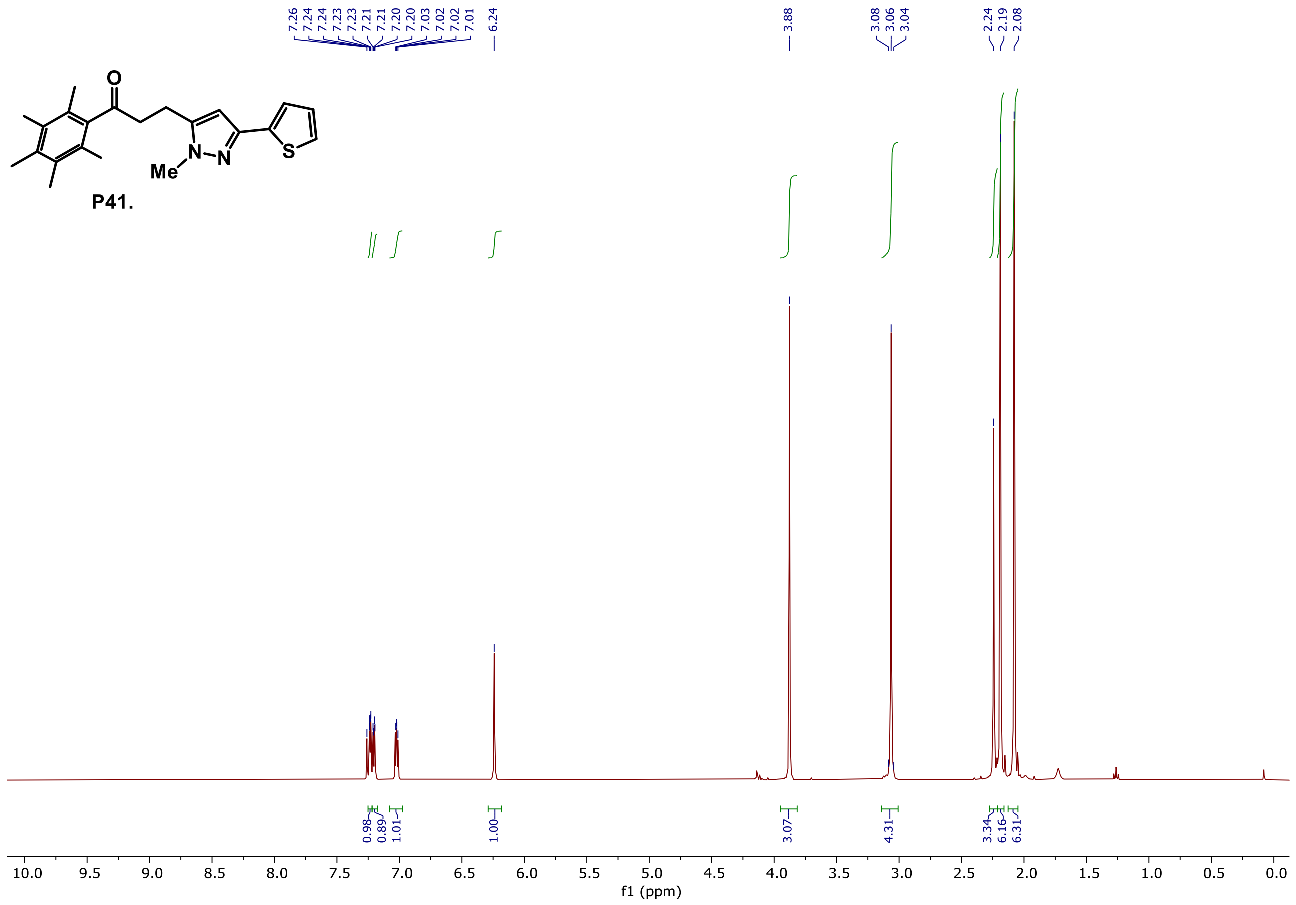

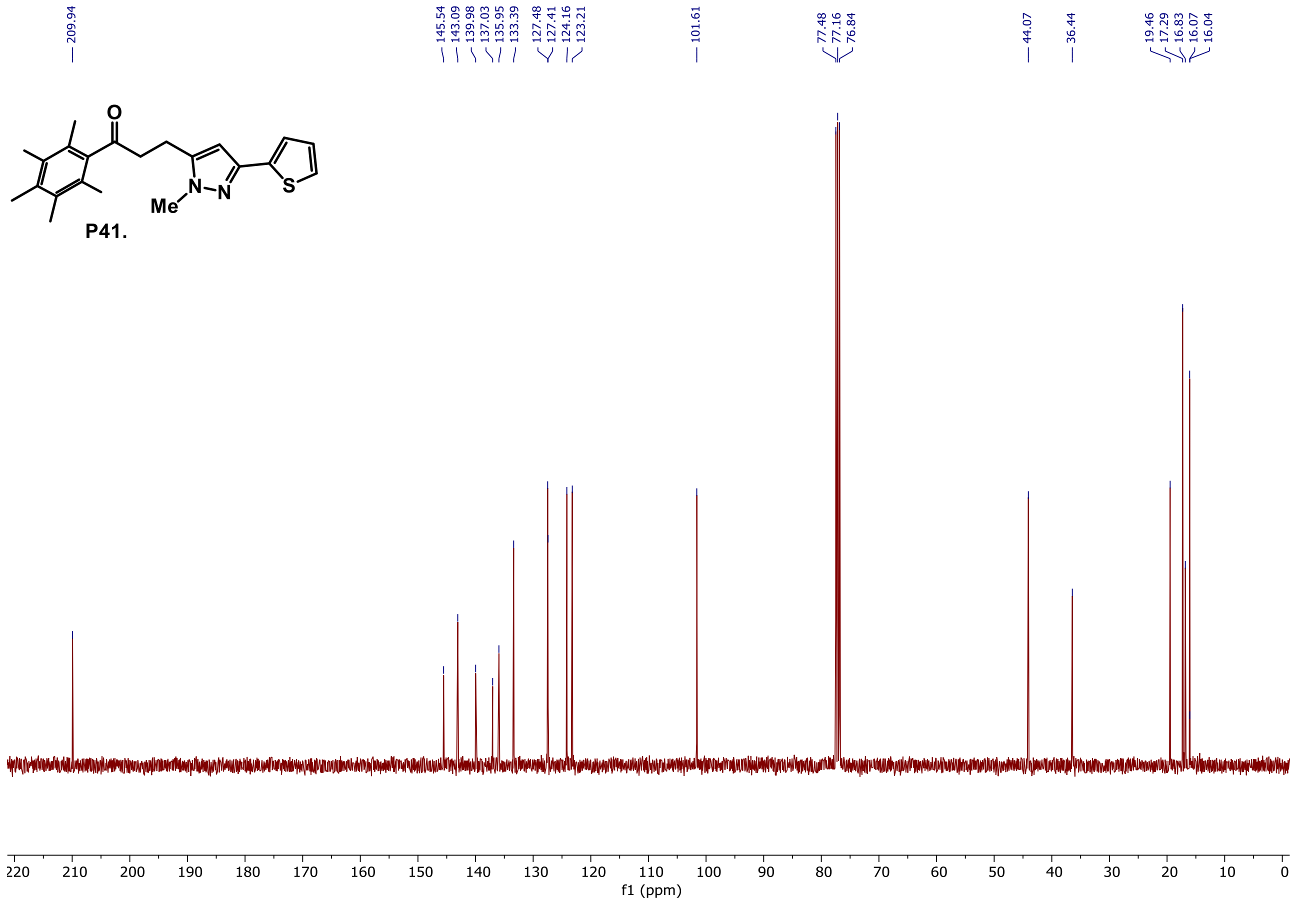

P42: **¹H NMR** (400 MHz, $CDCl_3$) and **¹³C NMR** (101 MHz, $CDCl_3$) respectively.

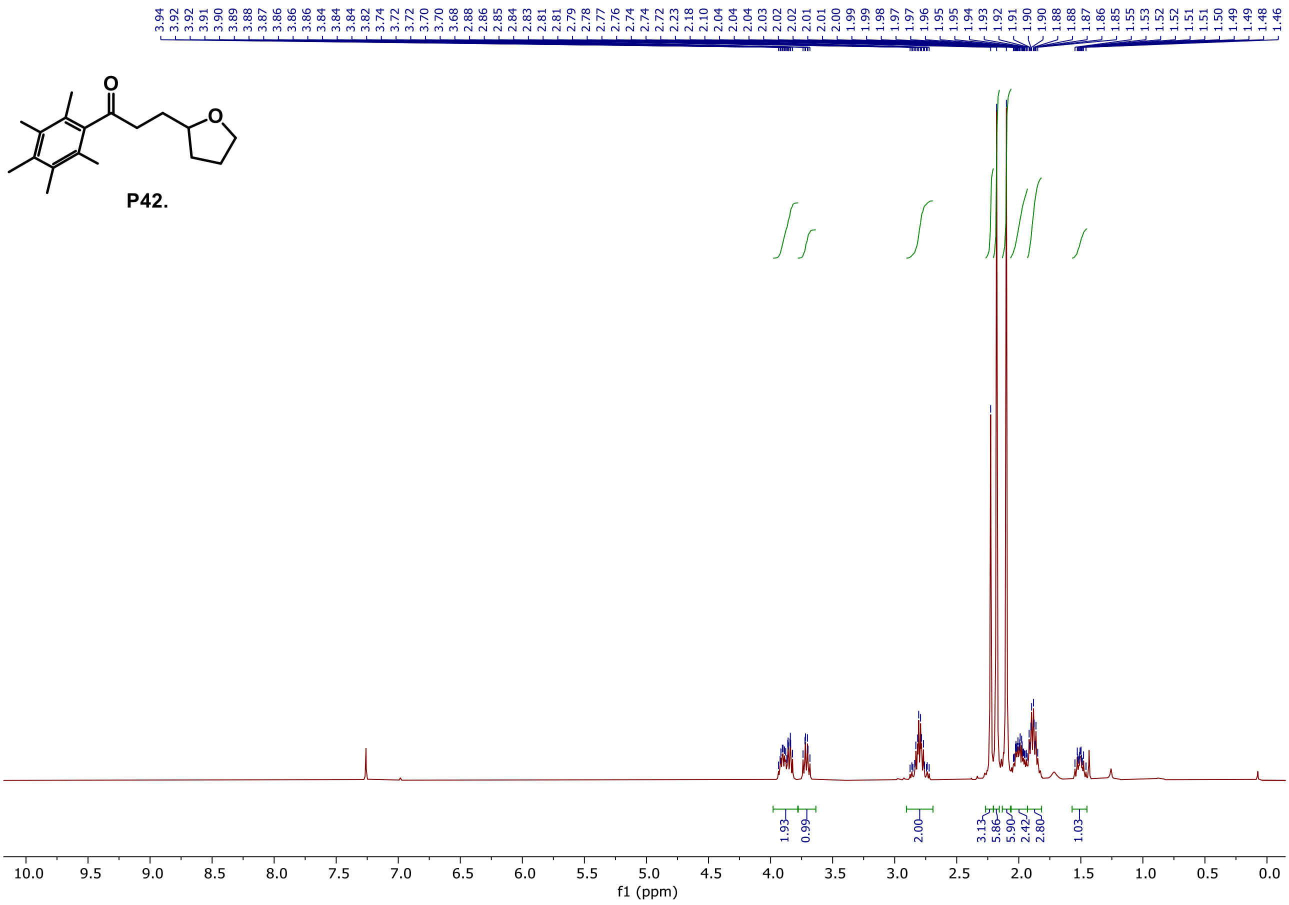

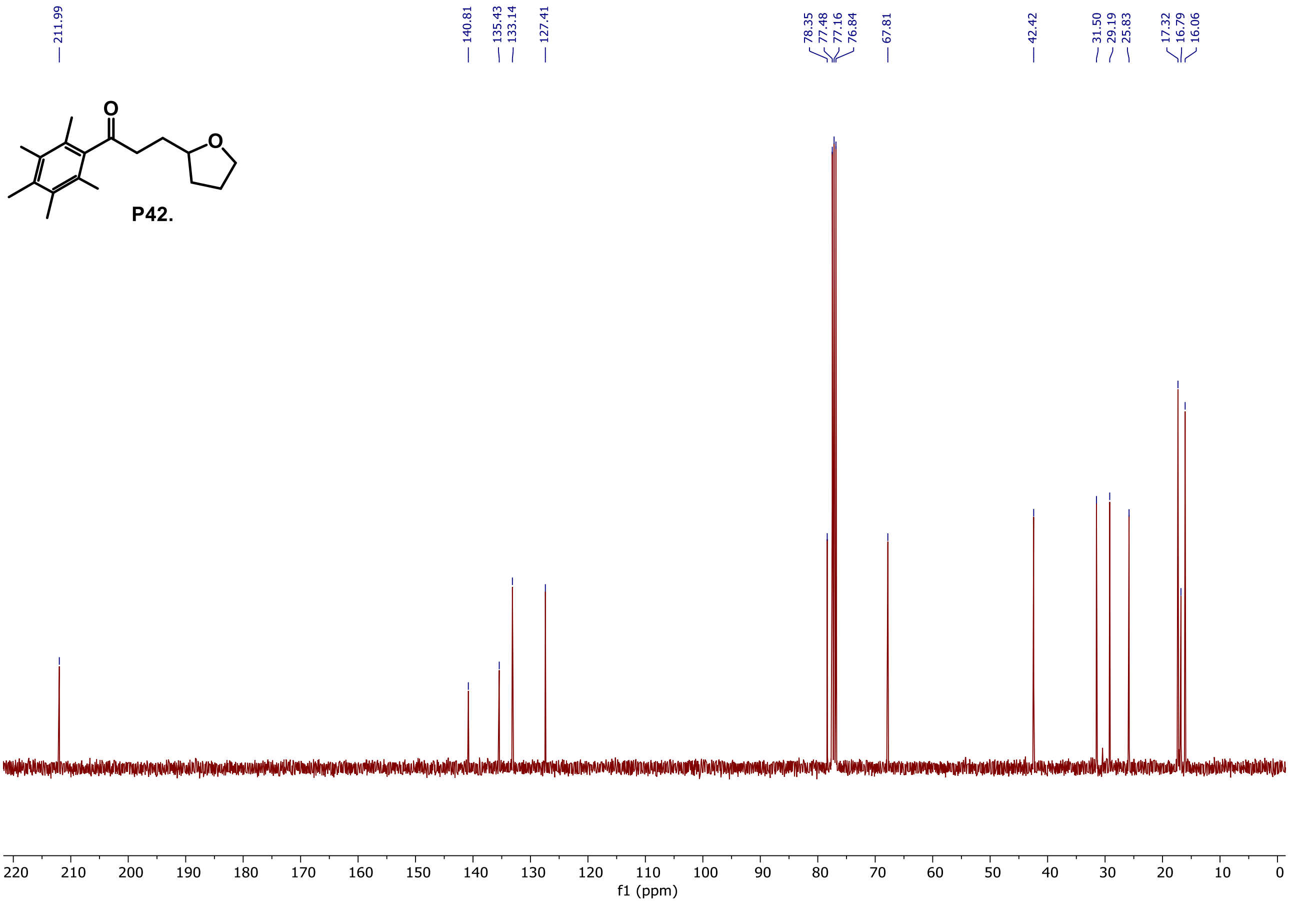

P43: ^{1}**H NMR** (400 MHz, $CDCl_3$) and ^{13}**C NMR** (101 MHz, $CDCl_3$) respectively.

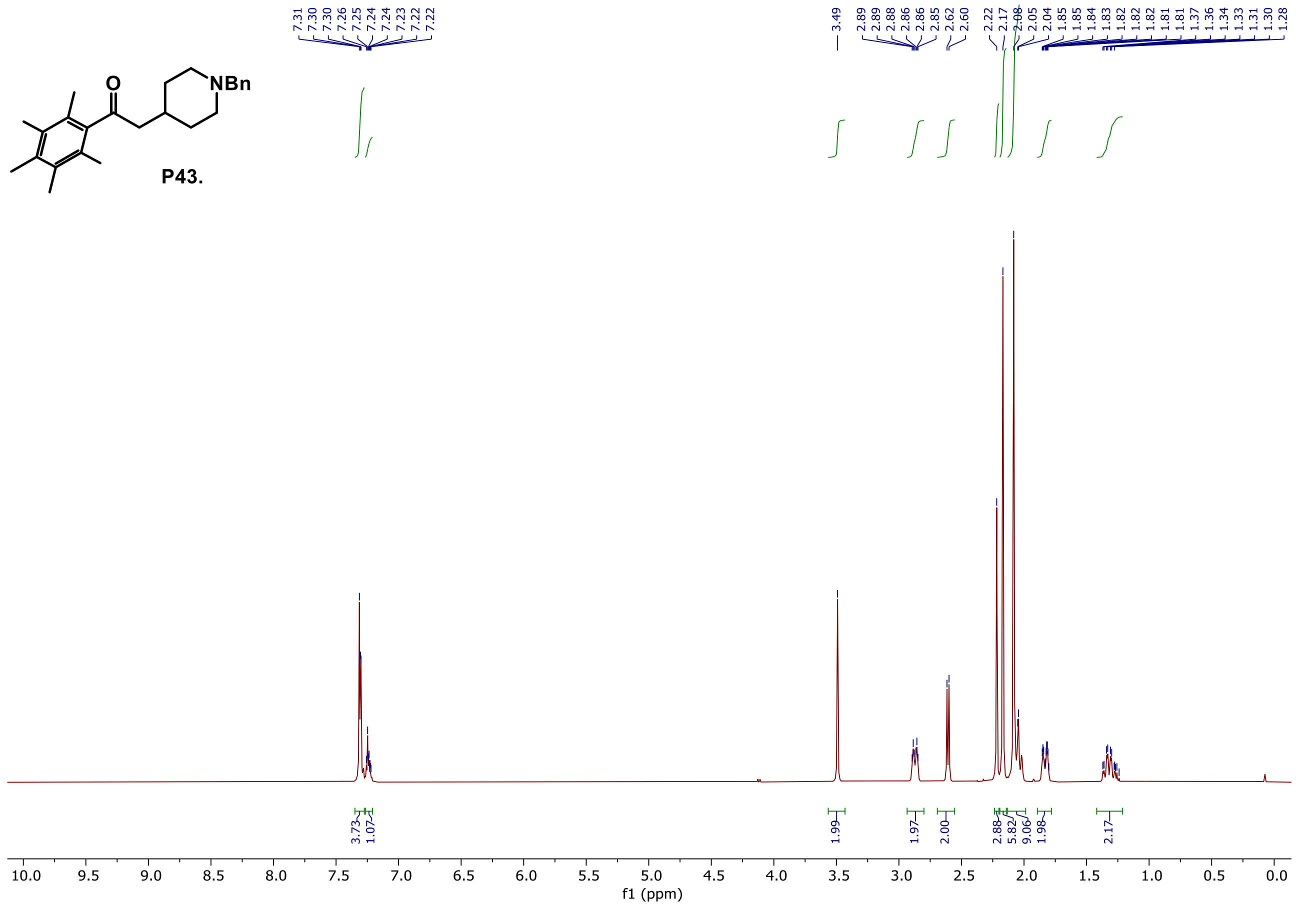

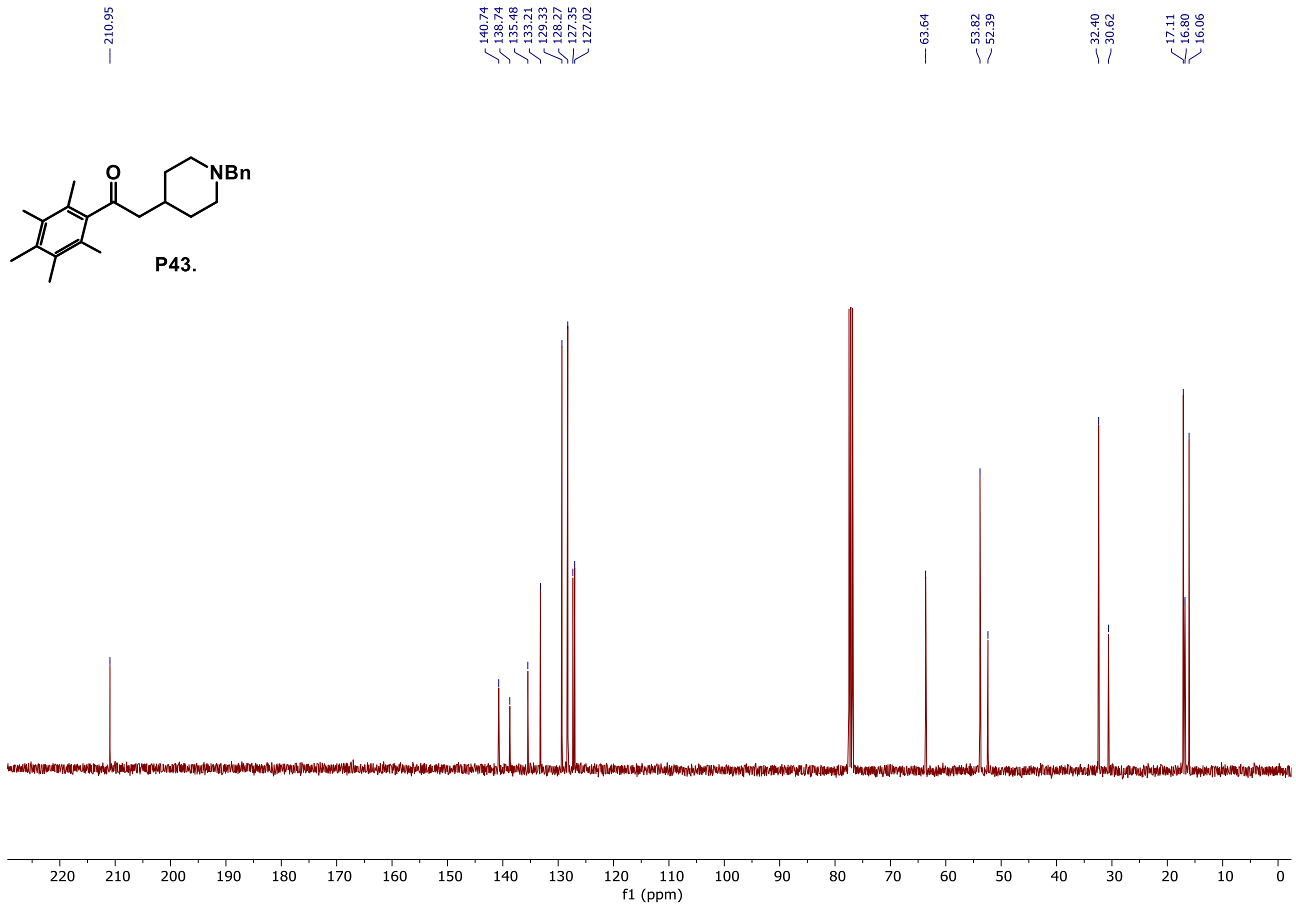

P44: **^{1}H NMR** (400 MHz, $CDCl_3$) and **^{13}C NMR** (101 MHz, $CDCl_3$) respectively.

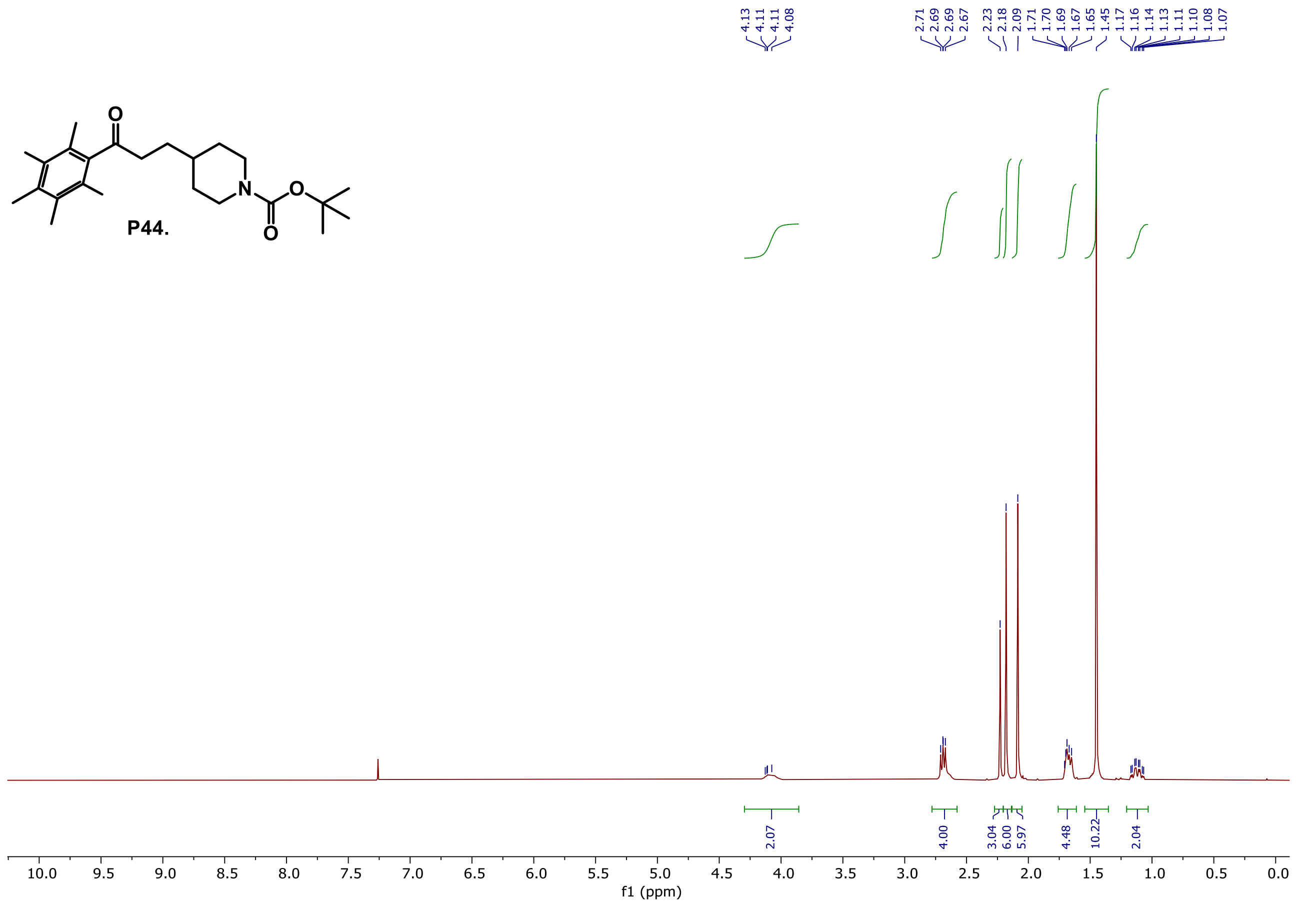

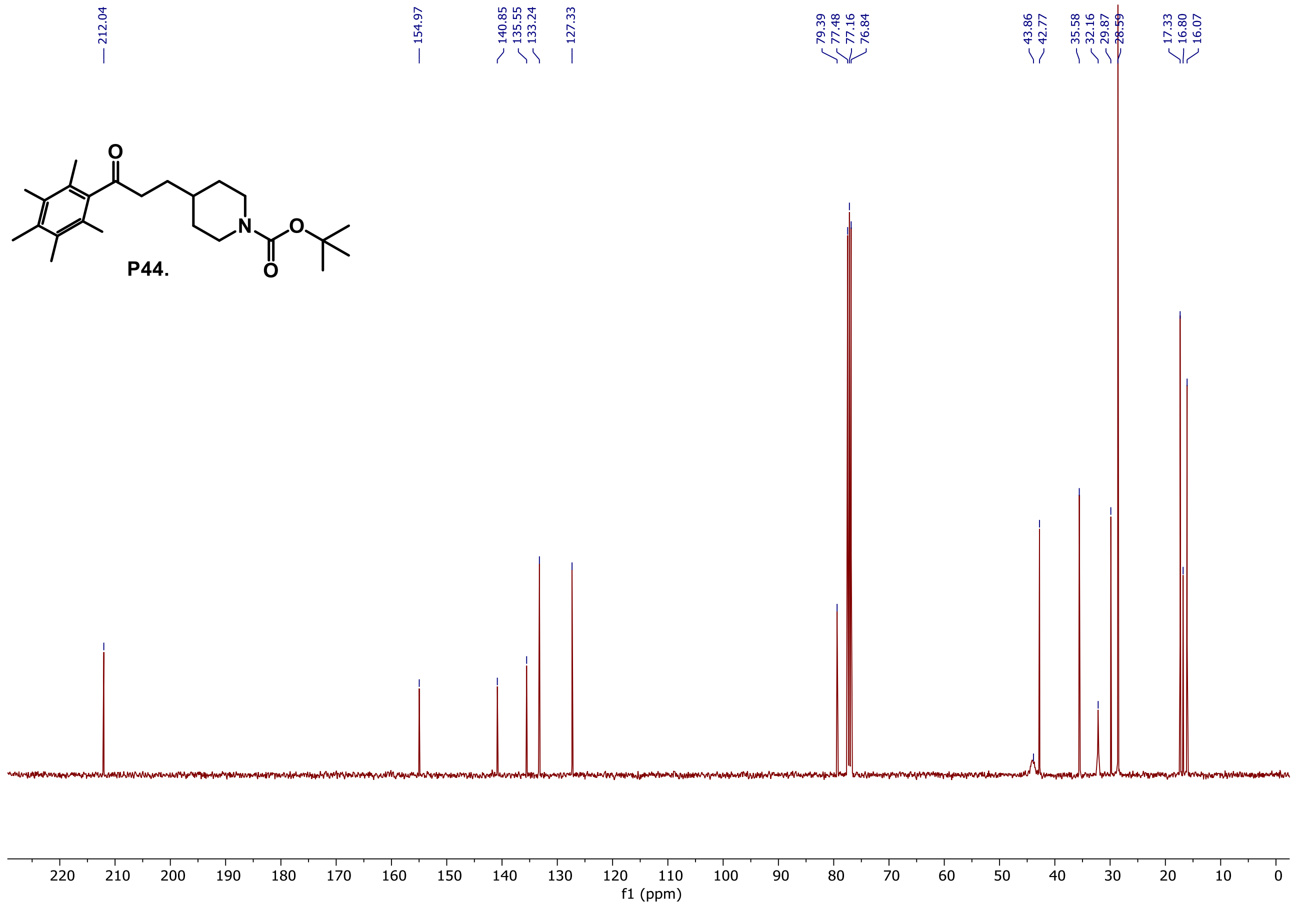

P45: **^{1}H NMR** (400 MHz, $CDCl_3$) and **^{13}C NMR** (101 MHz, $CDCl_3$) respectively.

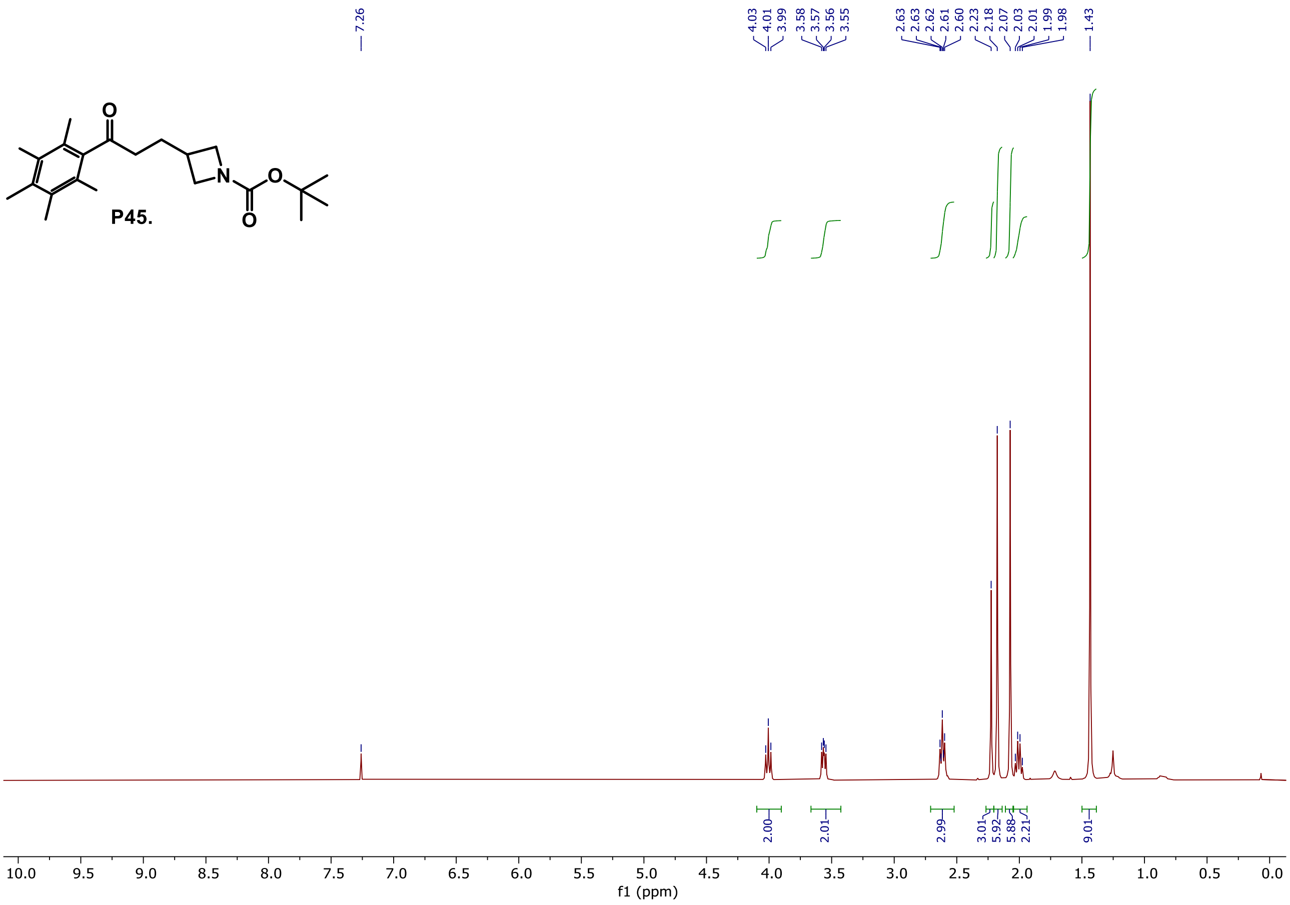

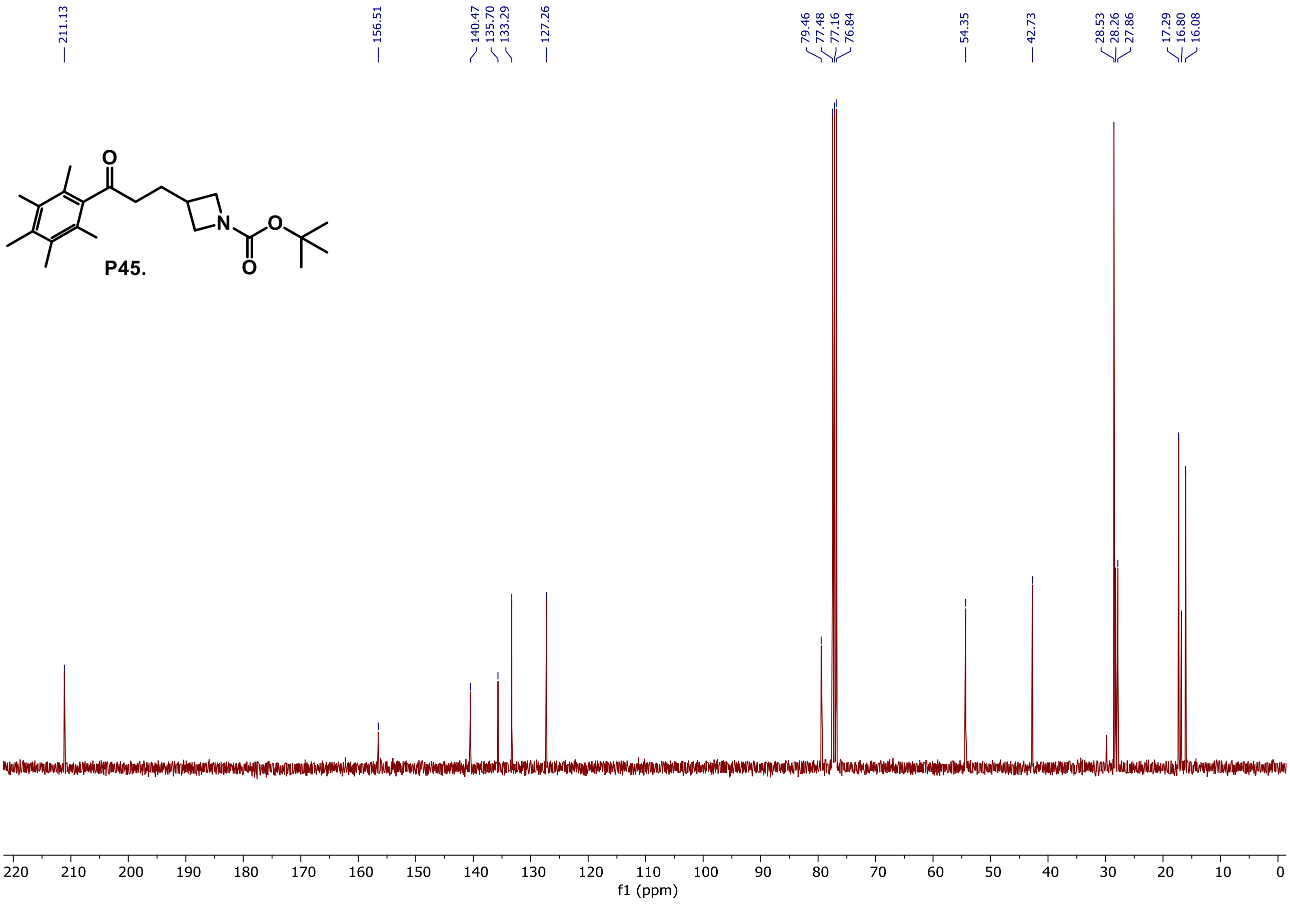

P46: ¹H NMR (400 MHz, $CDCl_3$) and **¹³C NMR** (101 MHz, $CDCl_3$) respectively.

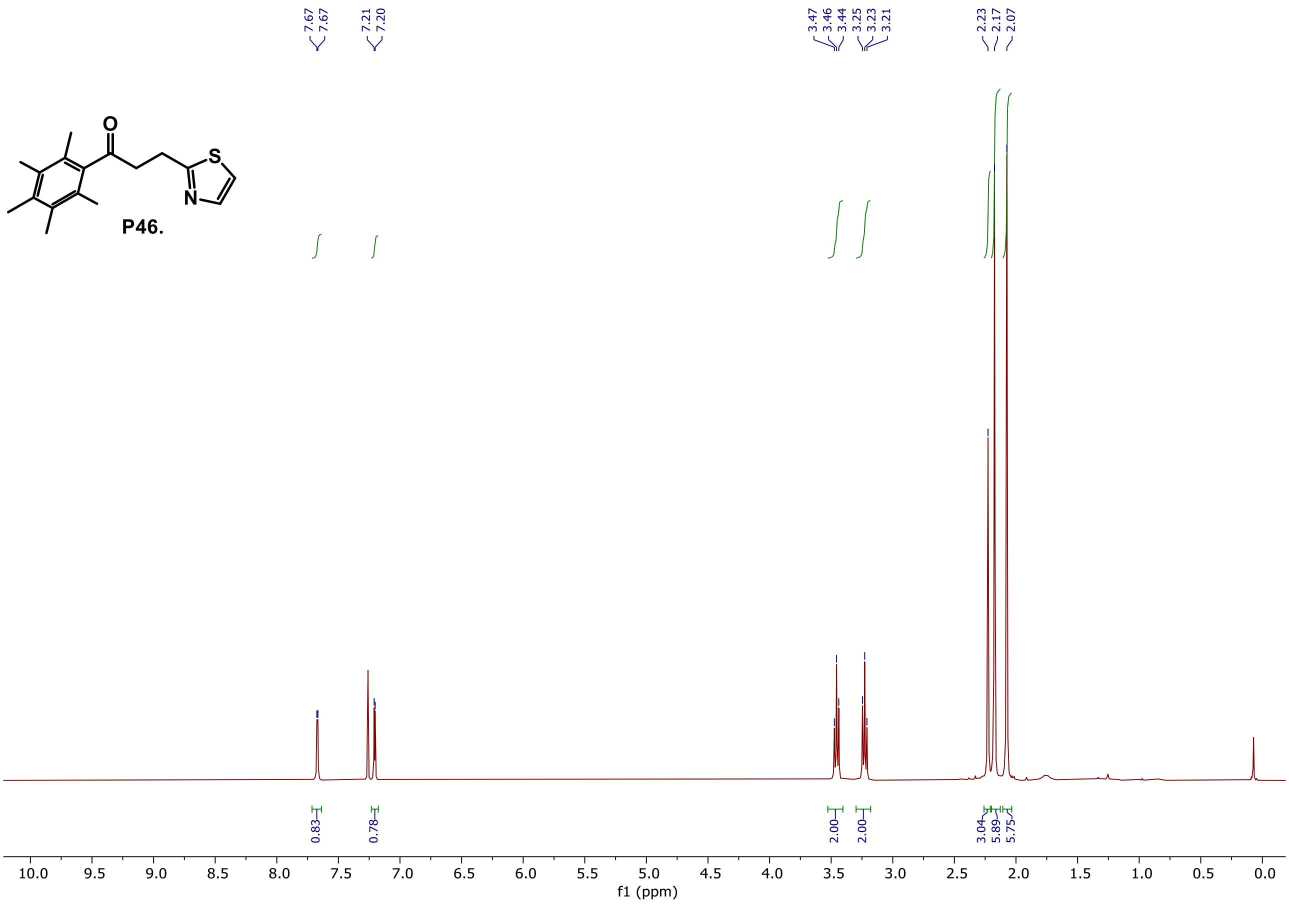

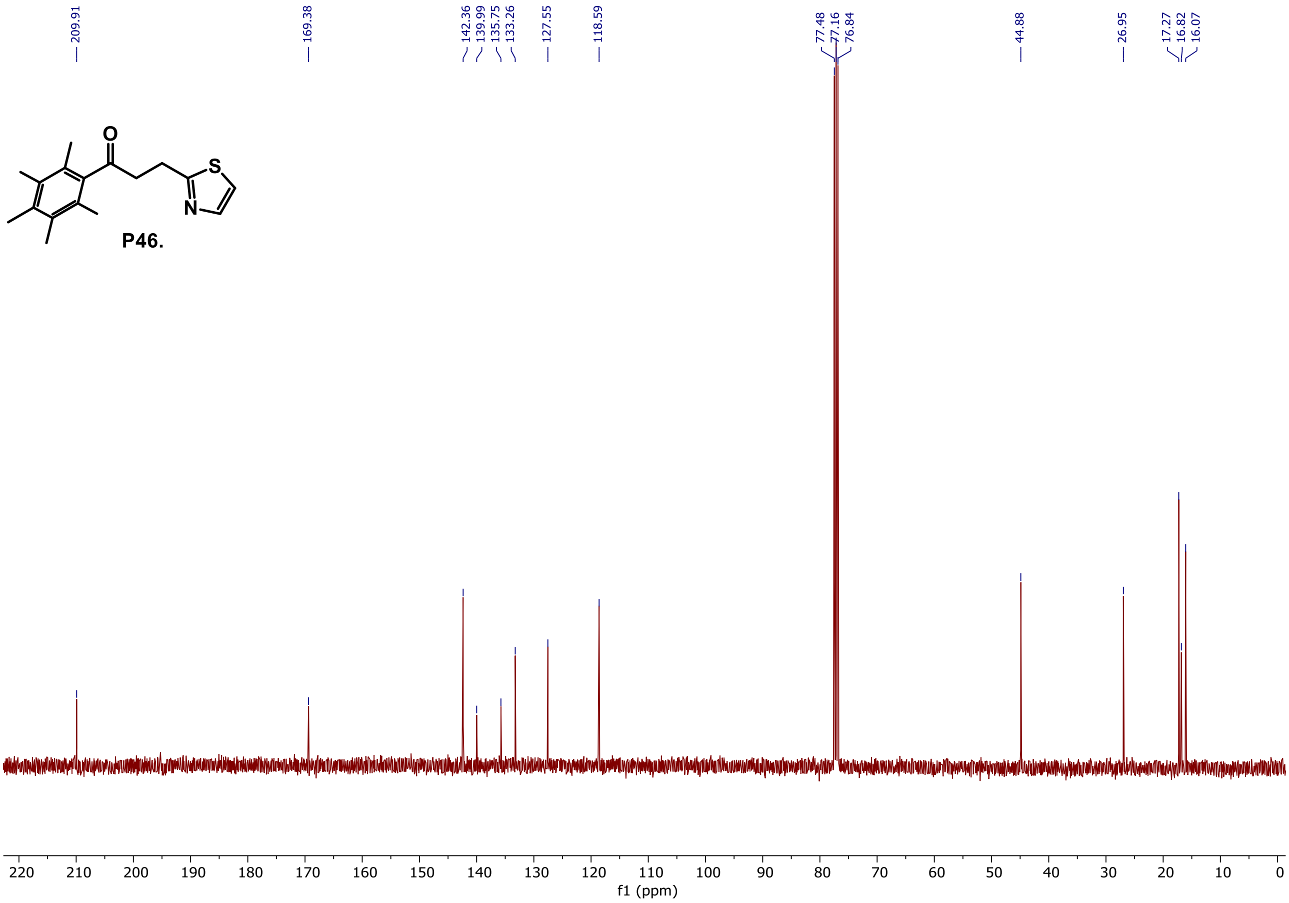

P47: ^{1}**H NMR** (400 MHz, $CDCl_3$) and ^{13}**C NMR** (101 MHz, $CDCl_3$) respectively.

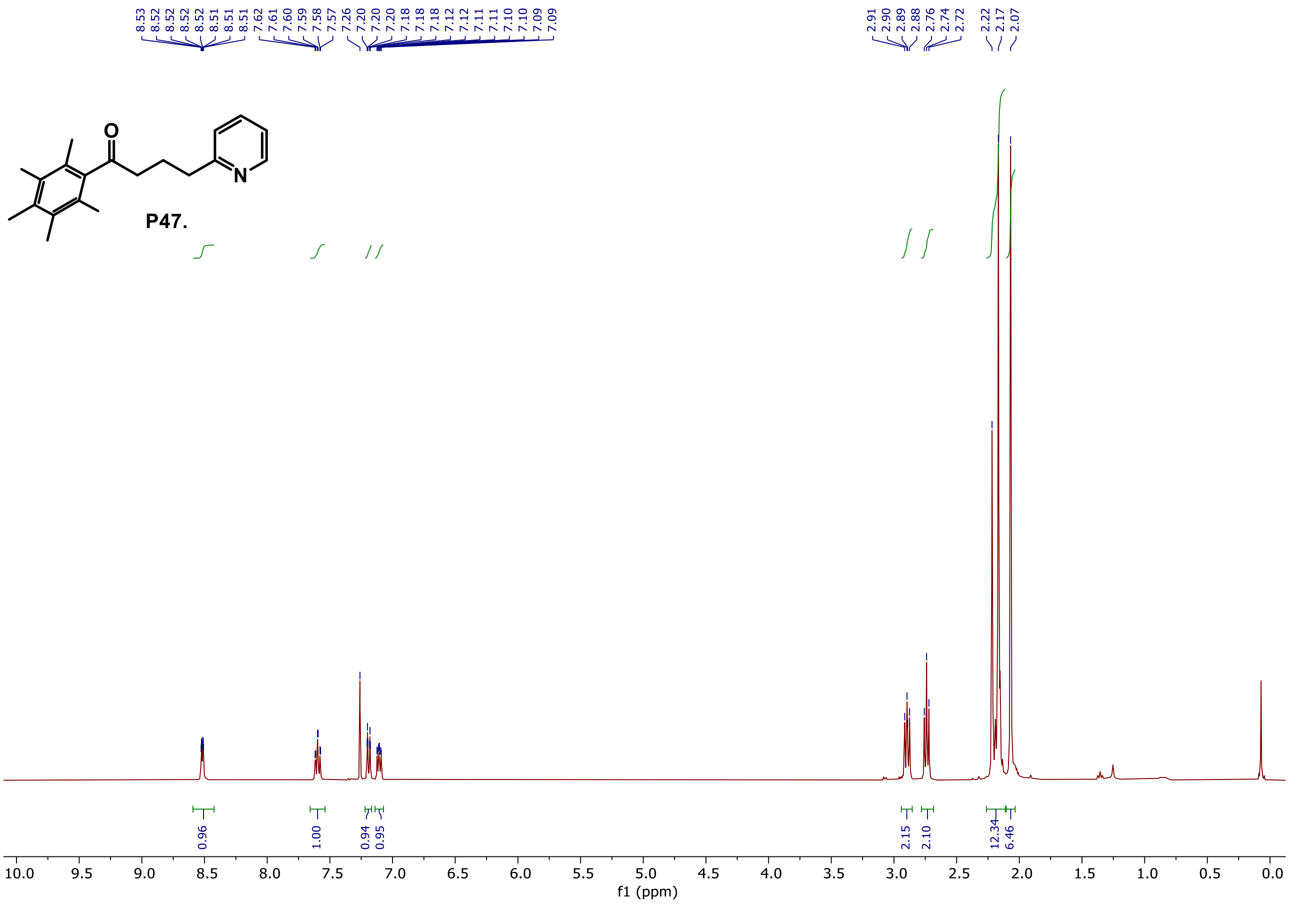

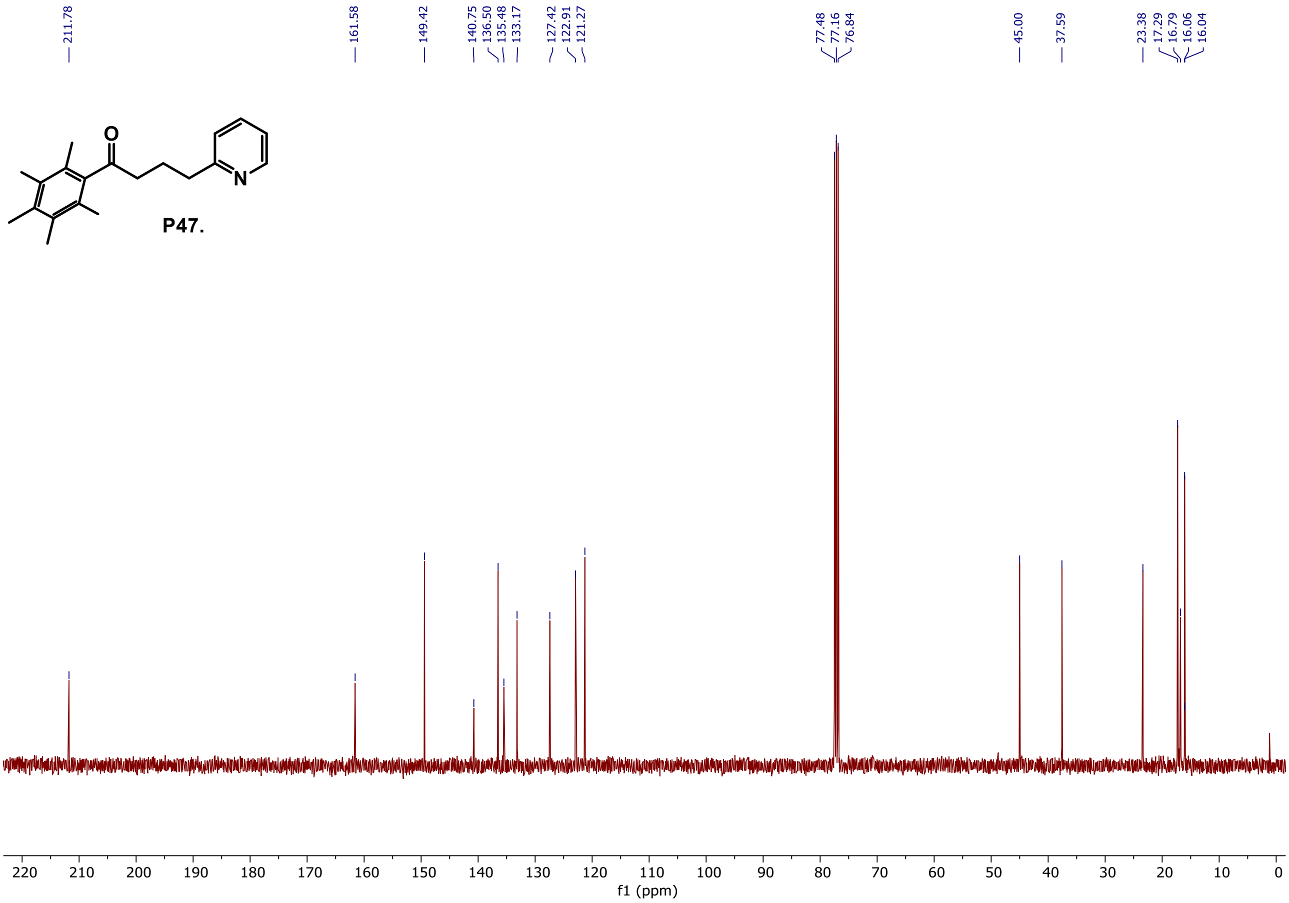

P48: ¹**H NMR** (400 MHz, $CDCl_3$) and ¹³**C NMR** (101 MHz, $CDCl_3$) respectively.

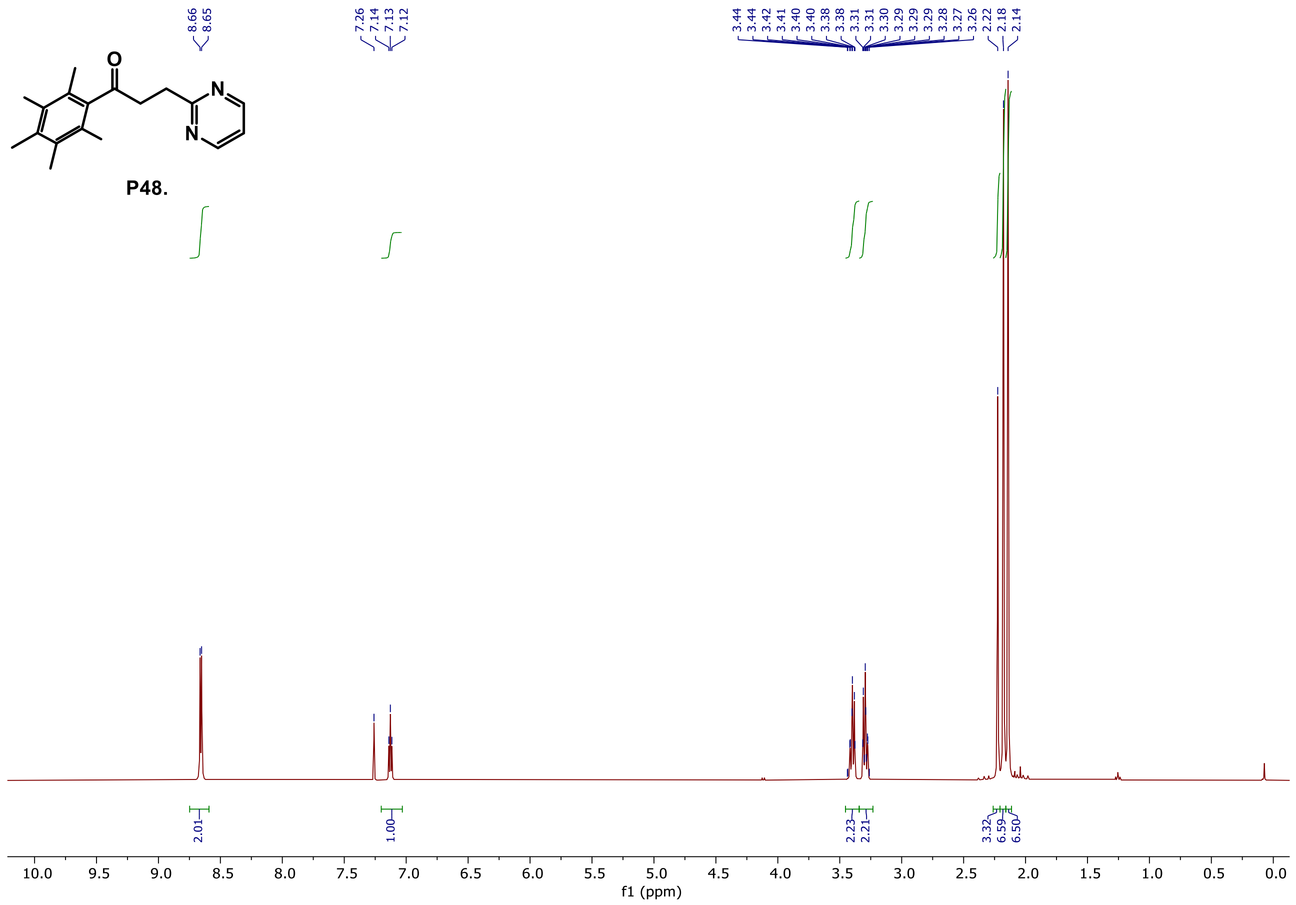

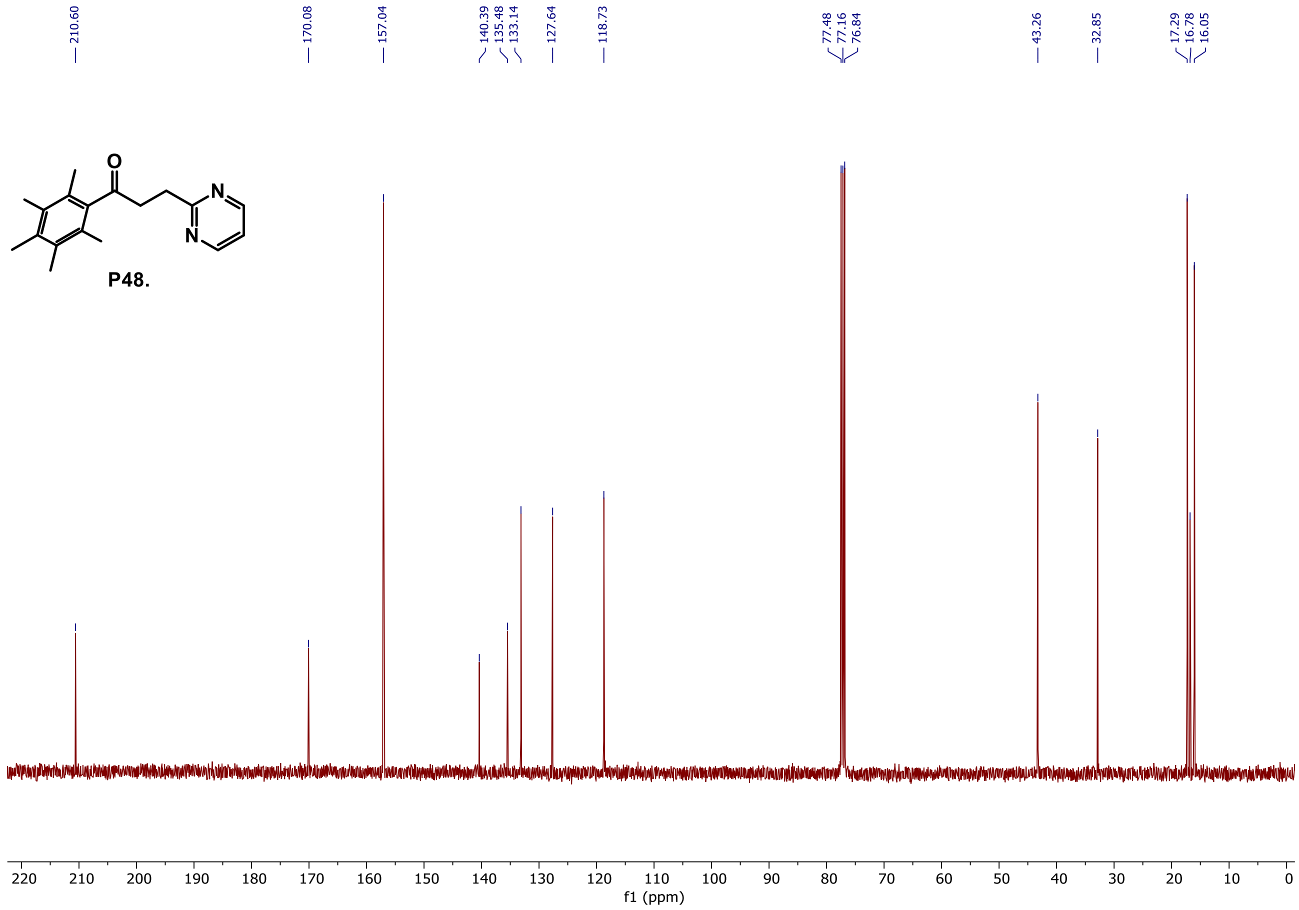

P49: ^{1}**H NMR** (400 MHz, $CDCl_3$) and ^{13}**C NMR** (101 MHz, $CDCl_3$) respectively.

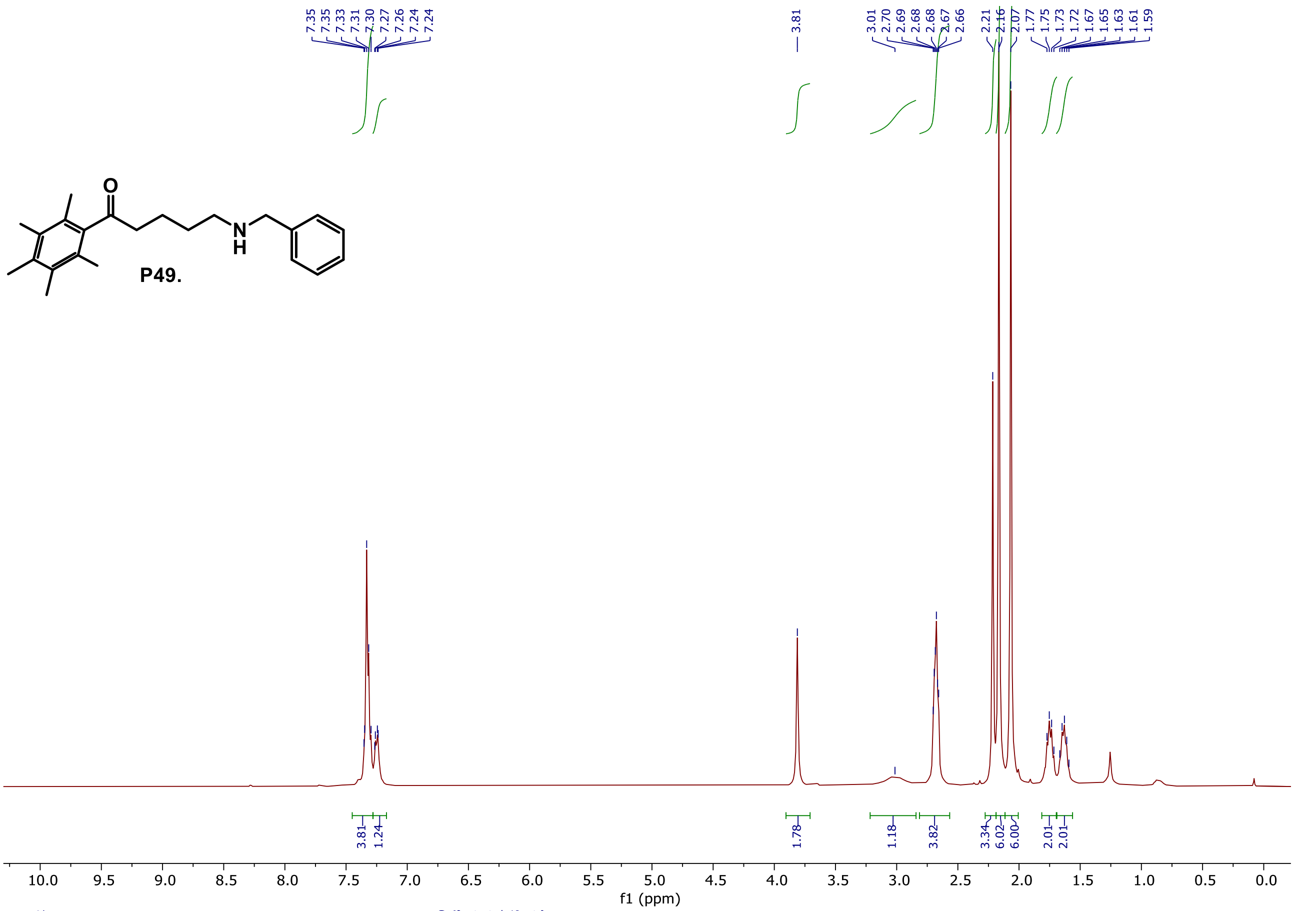

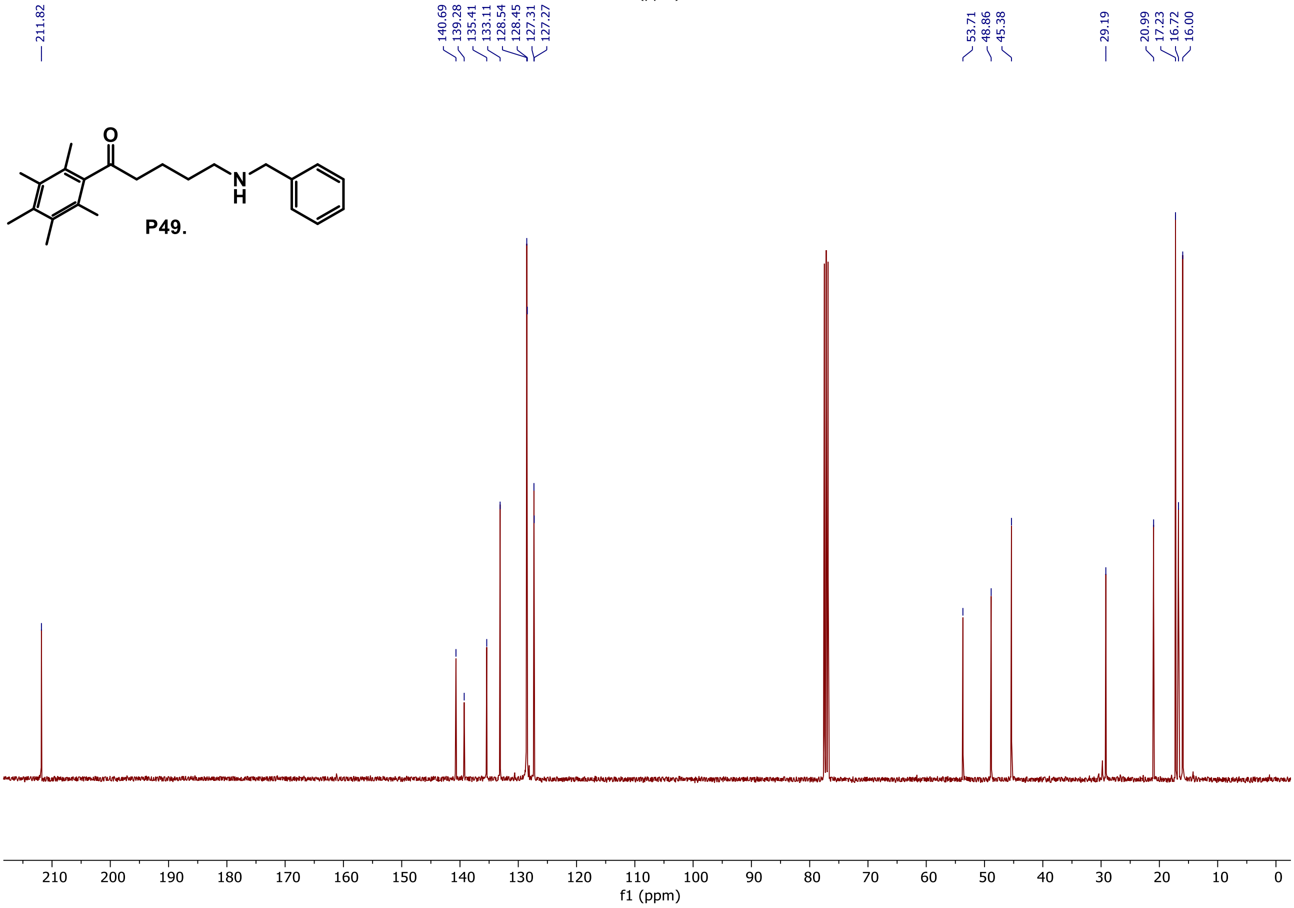

P50: [1]**H NMR** (400 MHz, $CDCl_3$) and [13]**C NMR** (101 MHz, $CDCl_3$) respectively.

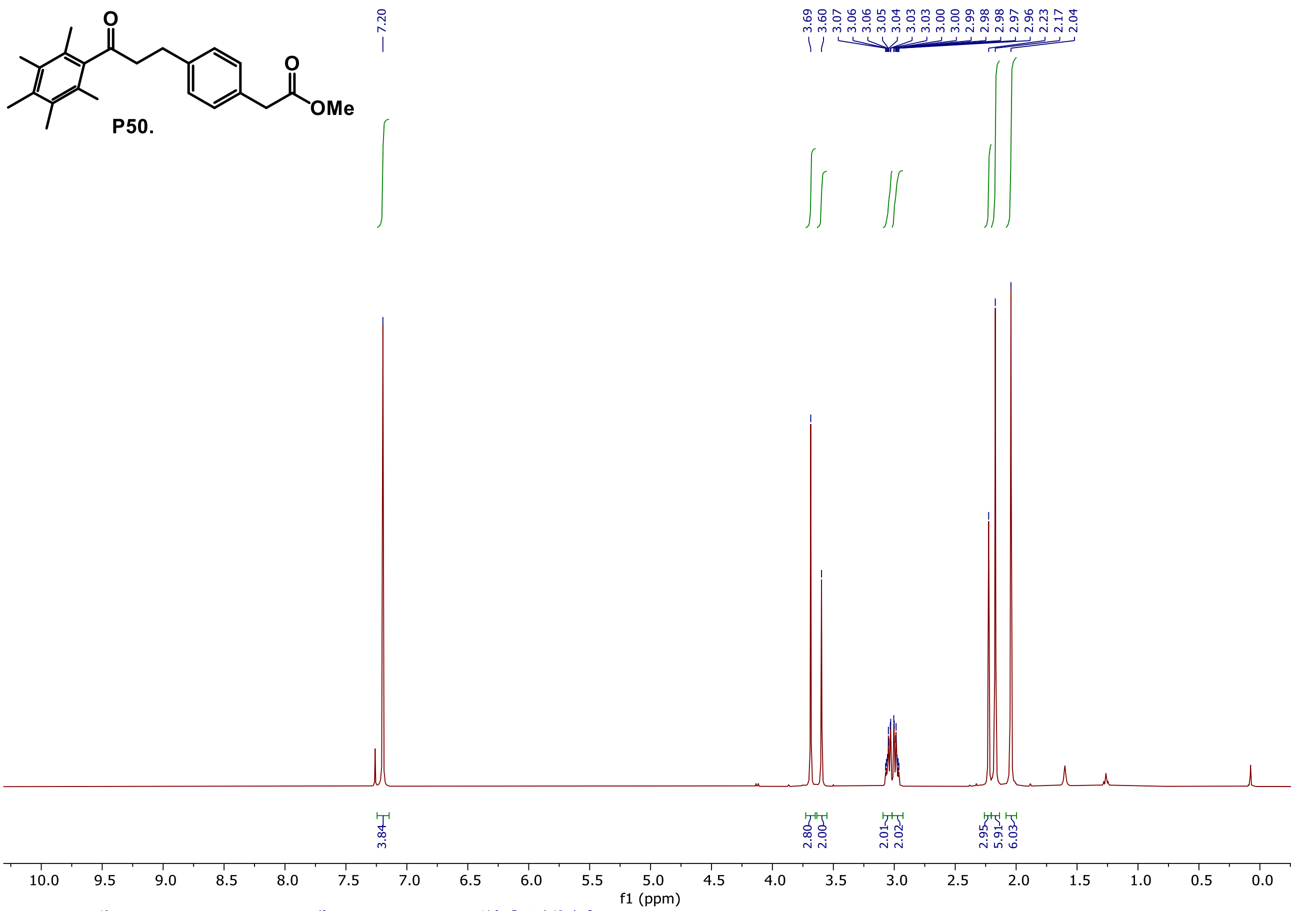

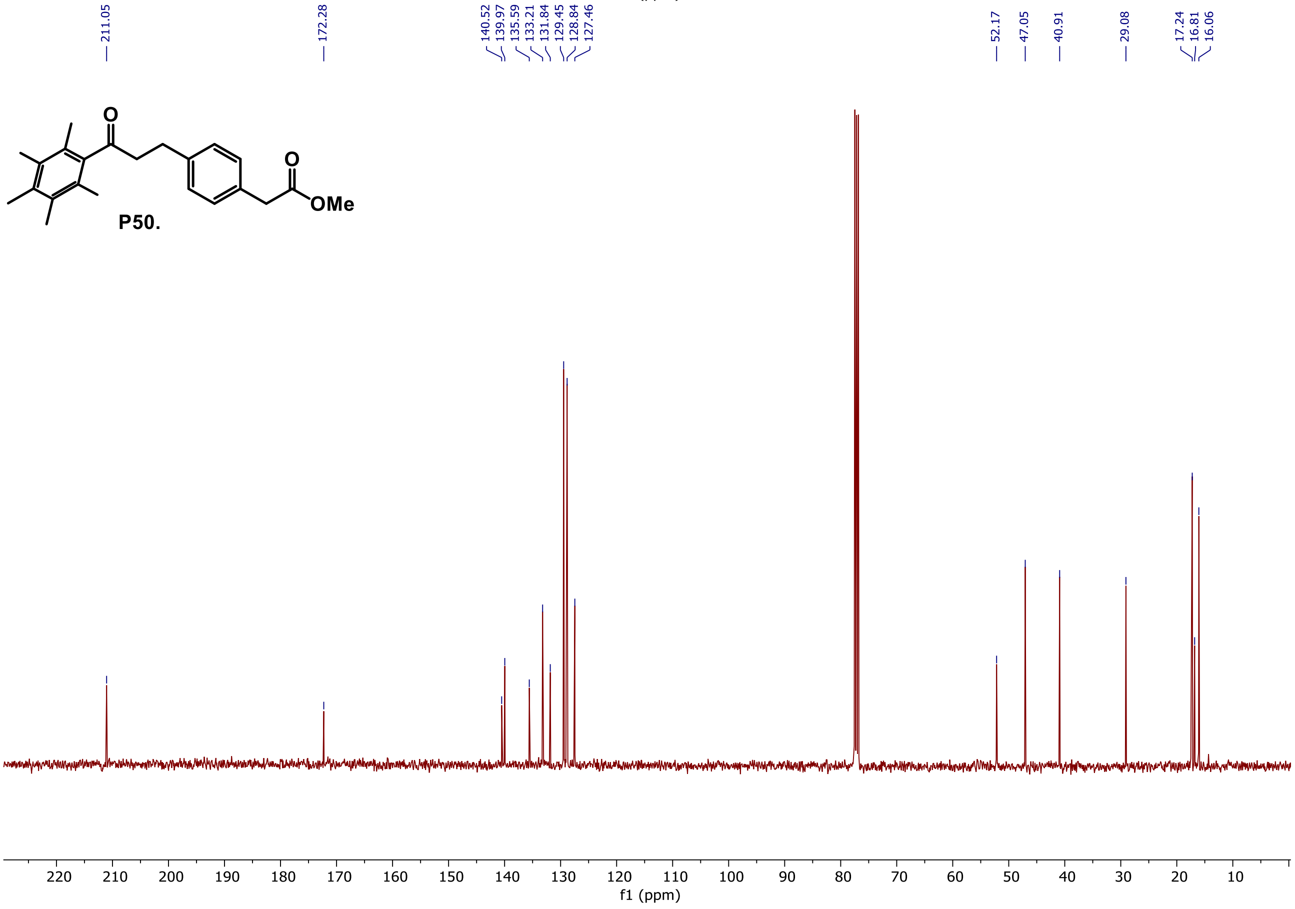

P51: **¹H NMR** (400 MHz, $CDCl_3$) and **¹³C NMR** (101 MHz, $CDCl_3$) respectively.

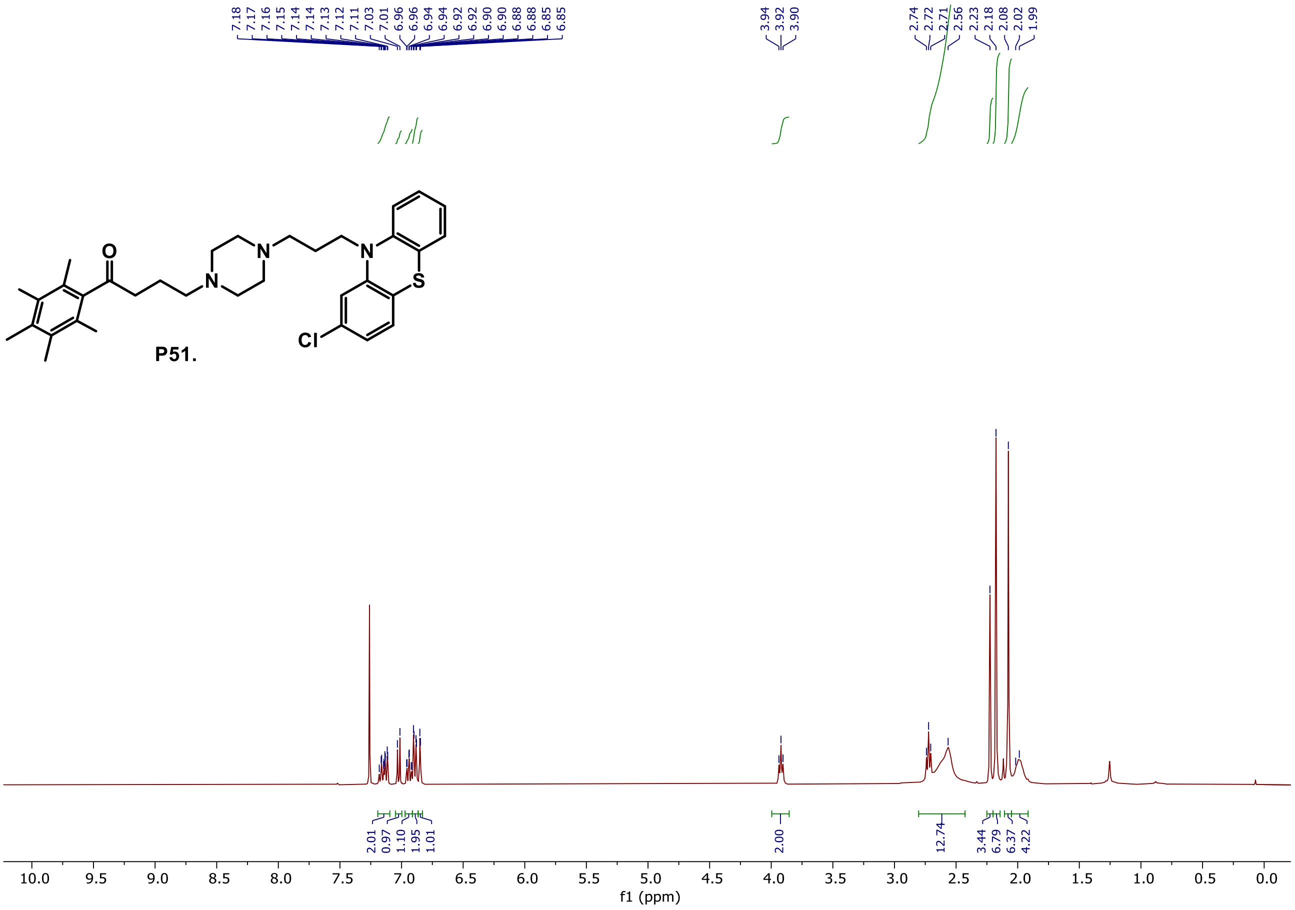

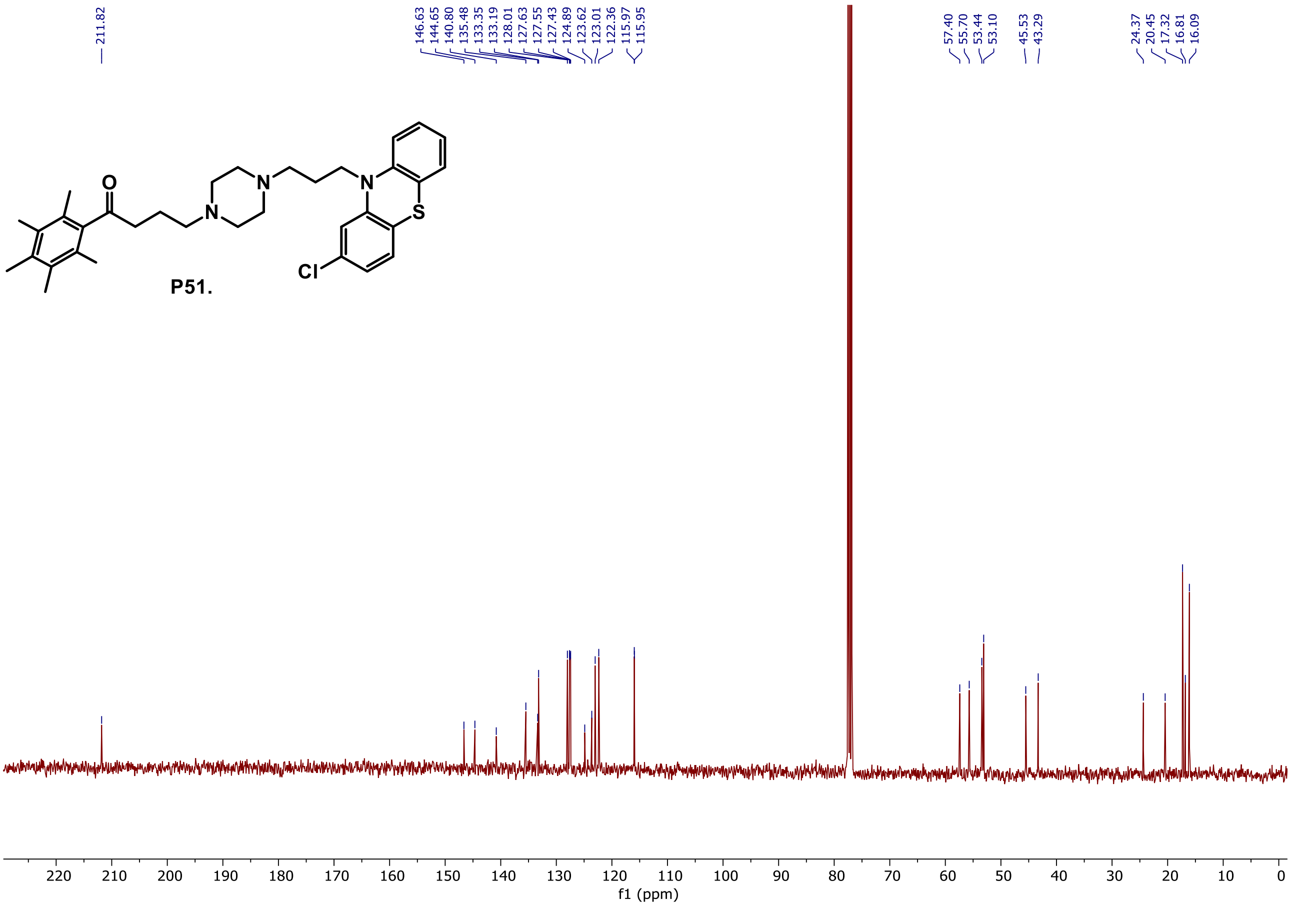

P52: [1]**H NMR** (400 MHz, $CDCl_3$) and [13]**C NMR** (101 MHz, $CDCl_3$) respectively.

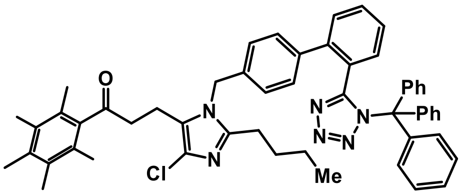

P52.

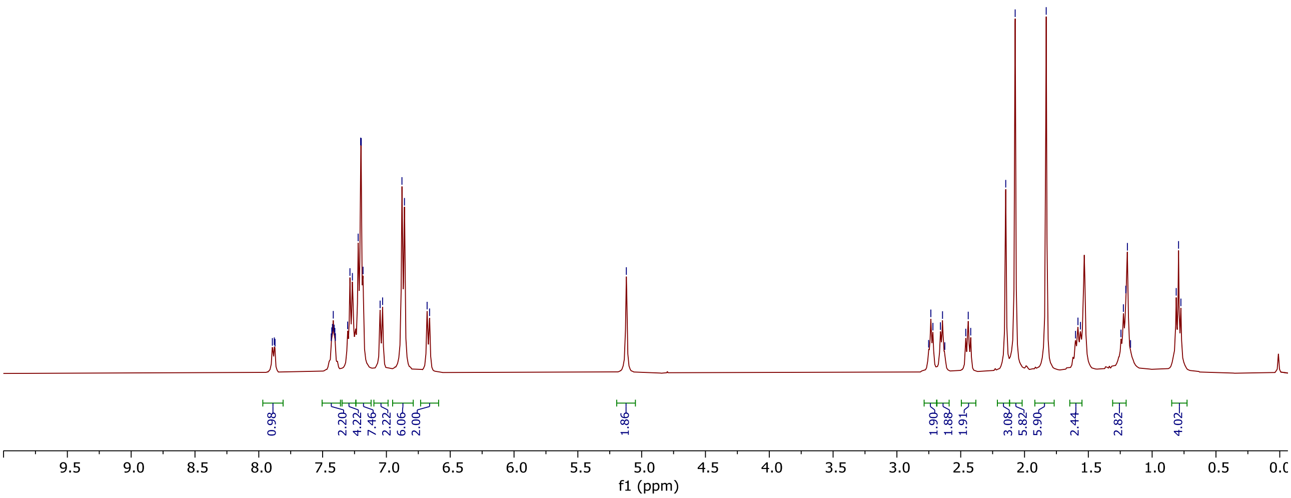

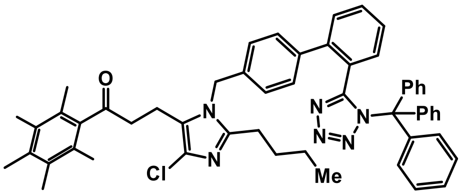

P52.

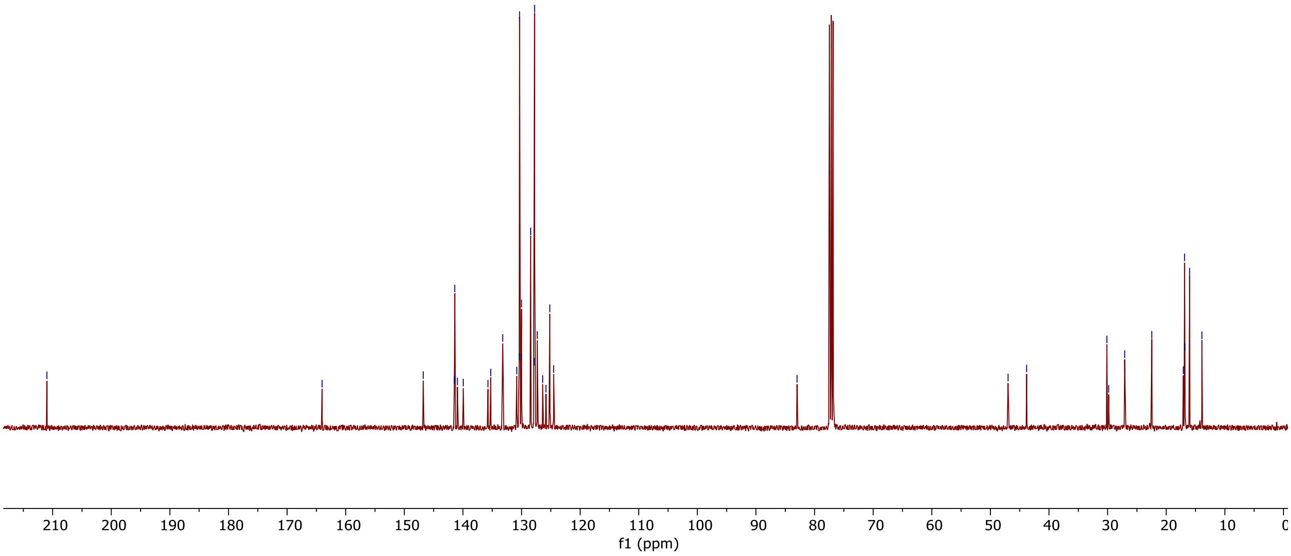

58: ¹H NMR (400 MHz, $CDCl_3$) and **¹³C NMR** (101 MHz, $CDCl_3$) respectively.

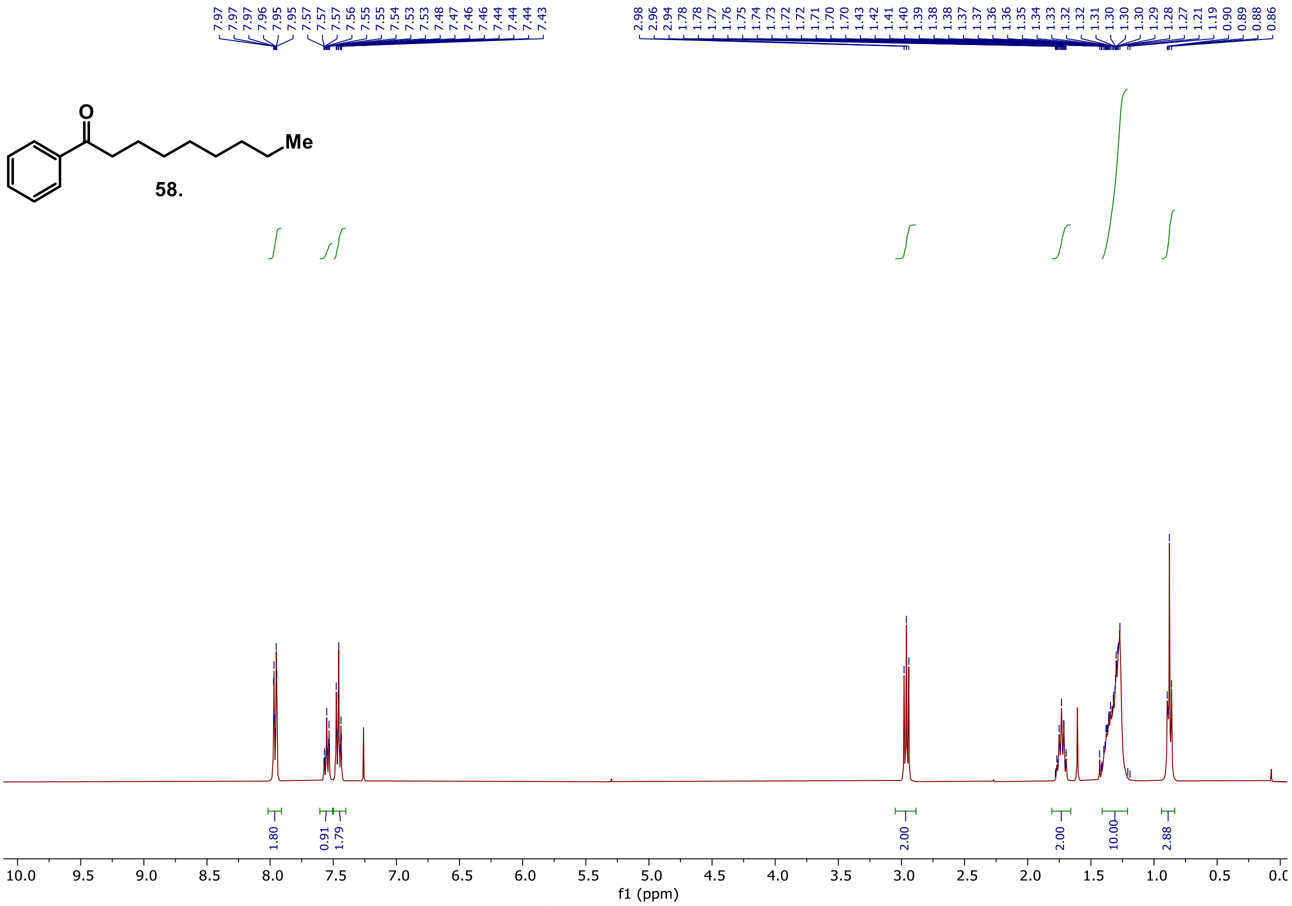

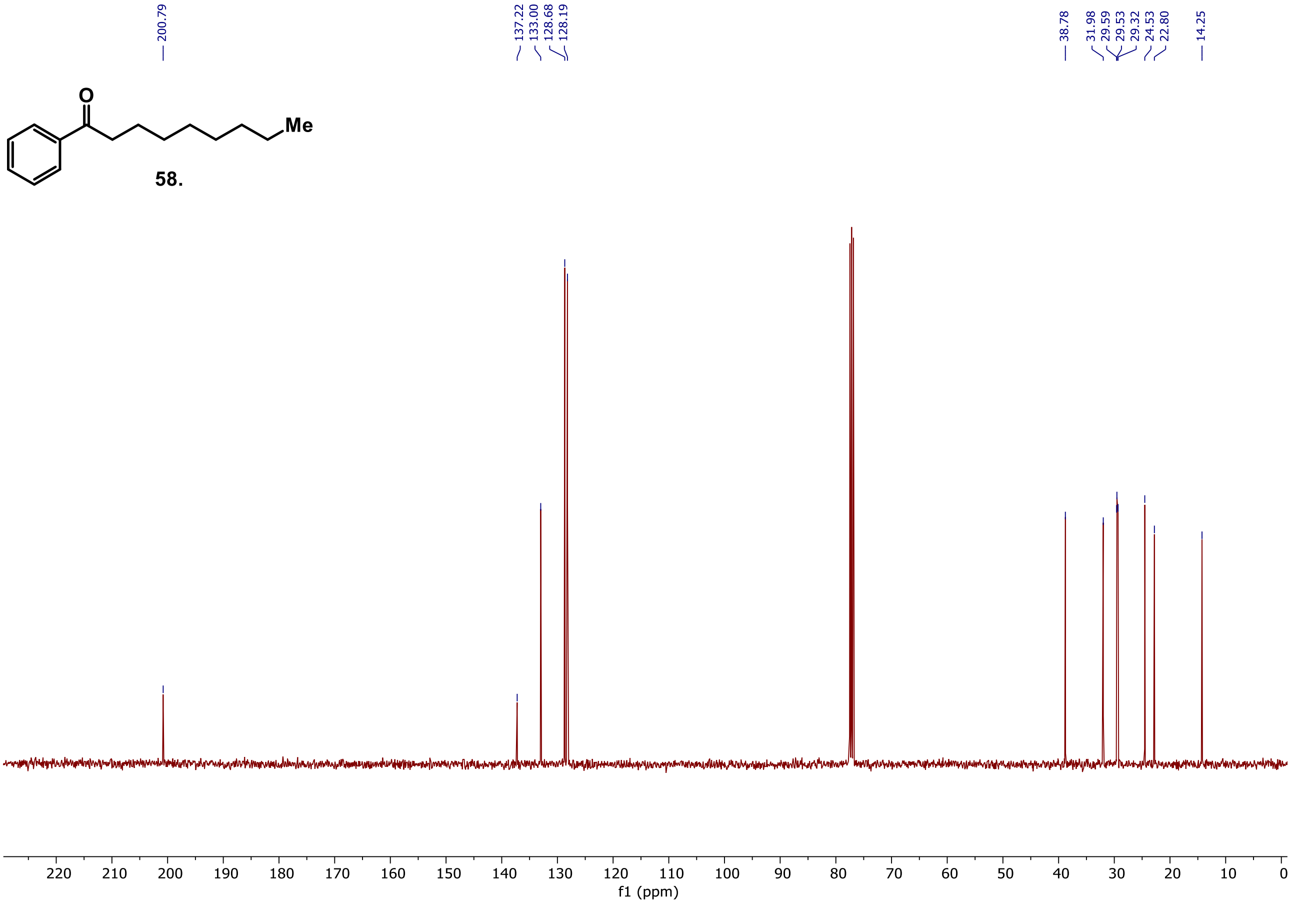

59: ¹H NMR (400 MHz, $CDCl_3$) and ¹³C NMR (101 MHz, $CDCl_3$) respectively.

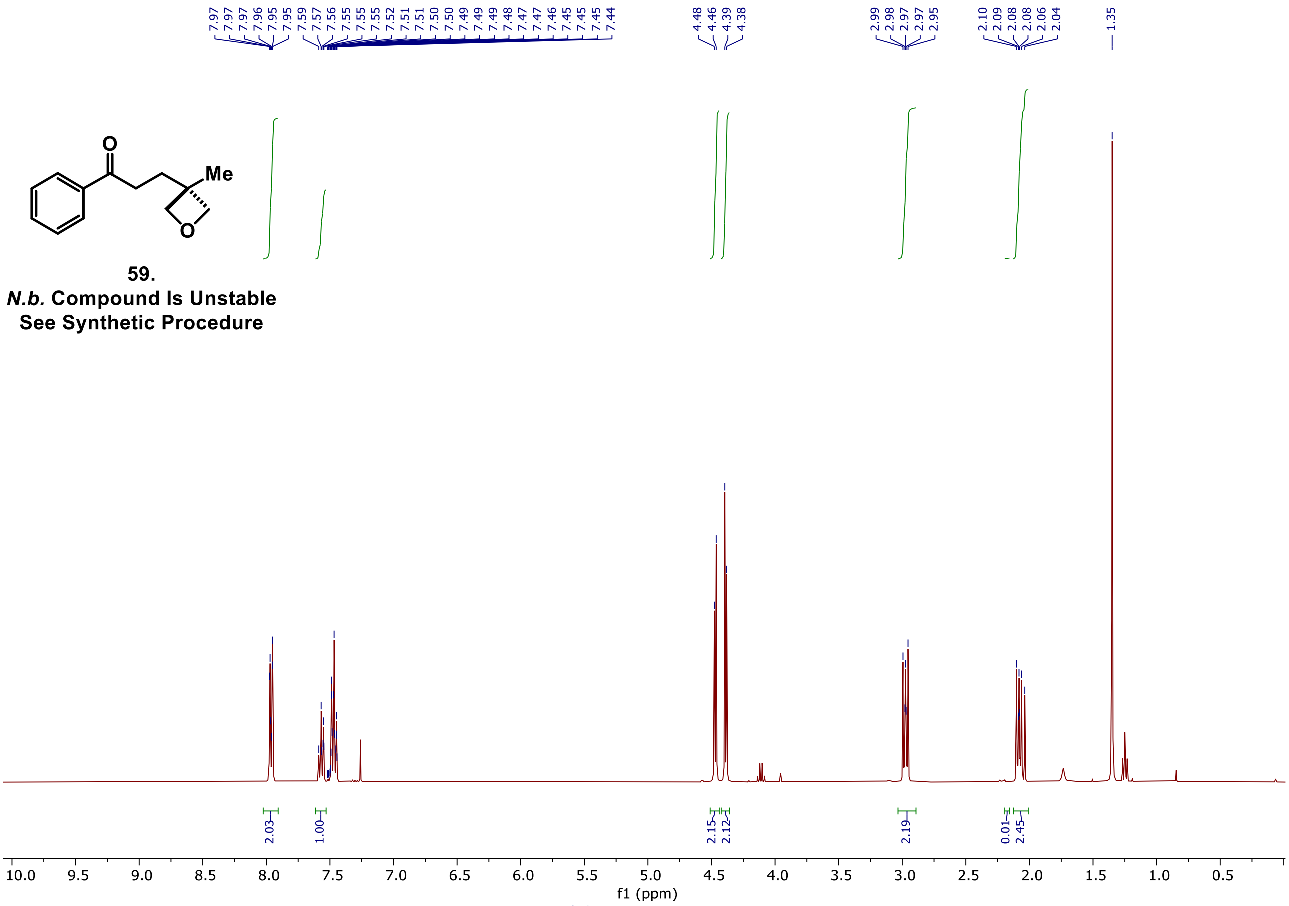

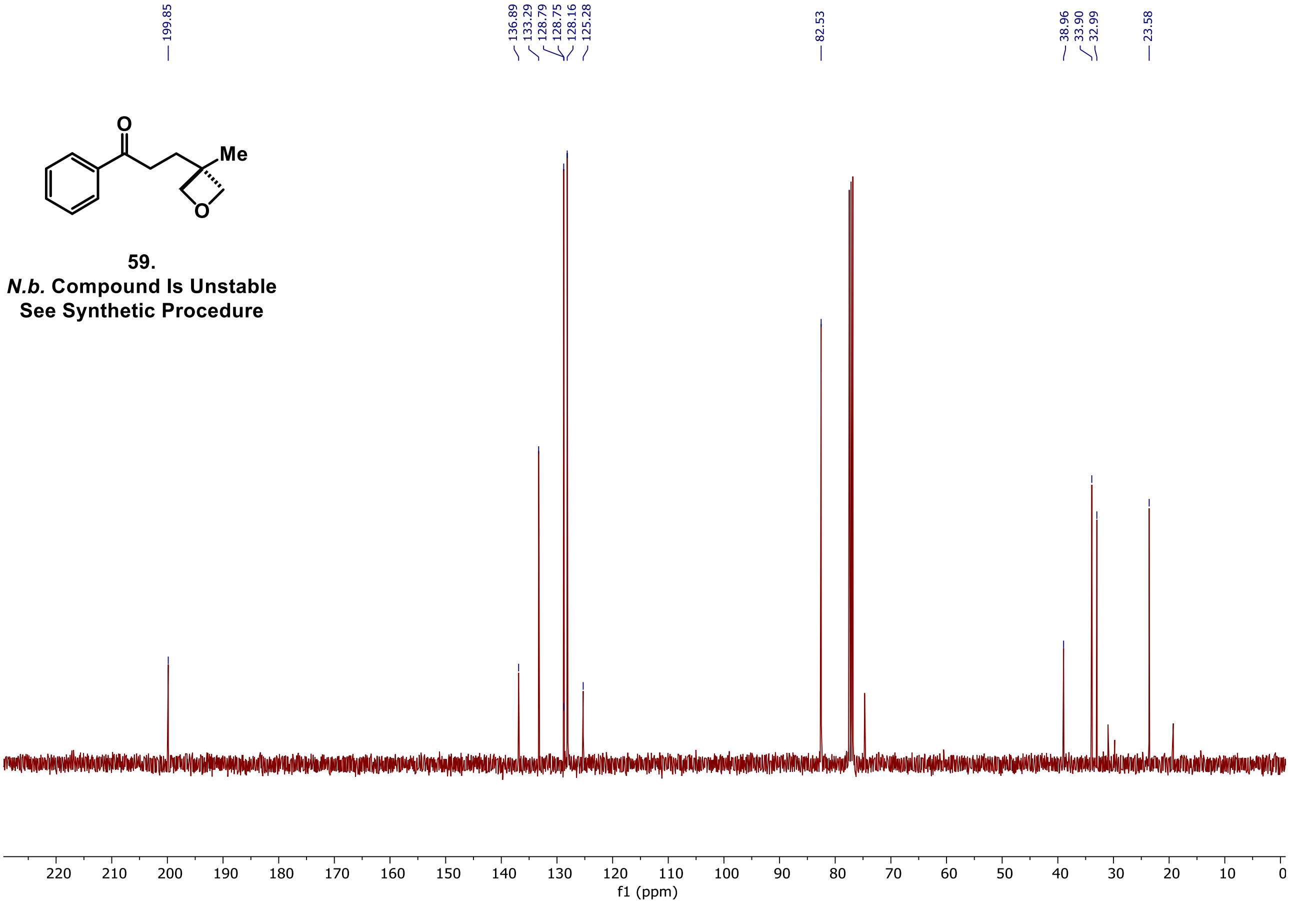

60: ^{1}H NMR (400 MHz, $CDCl_3$) and **^{13}C NMR** (101 MHz, $CDCl_3$) respectively.

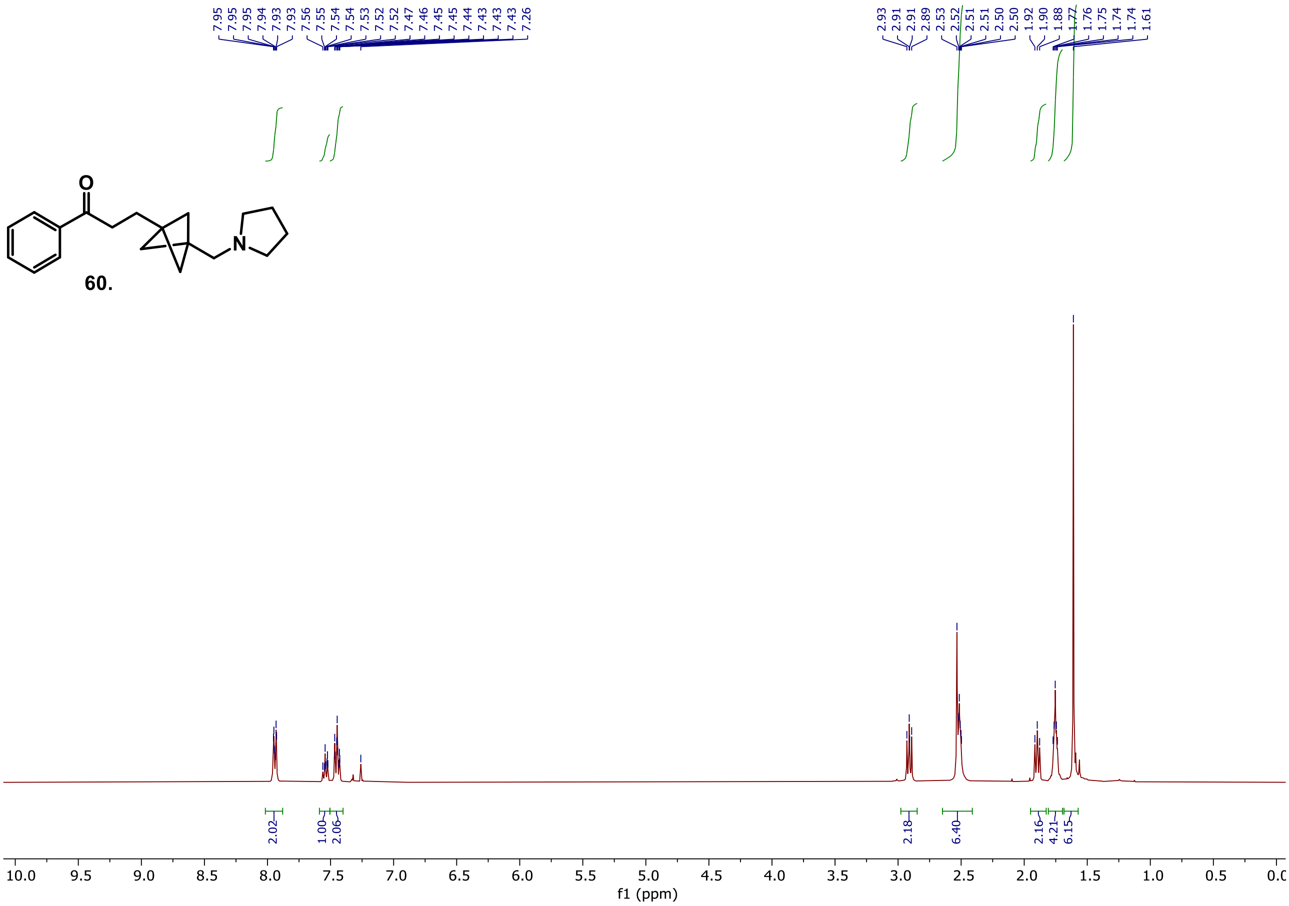

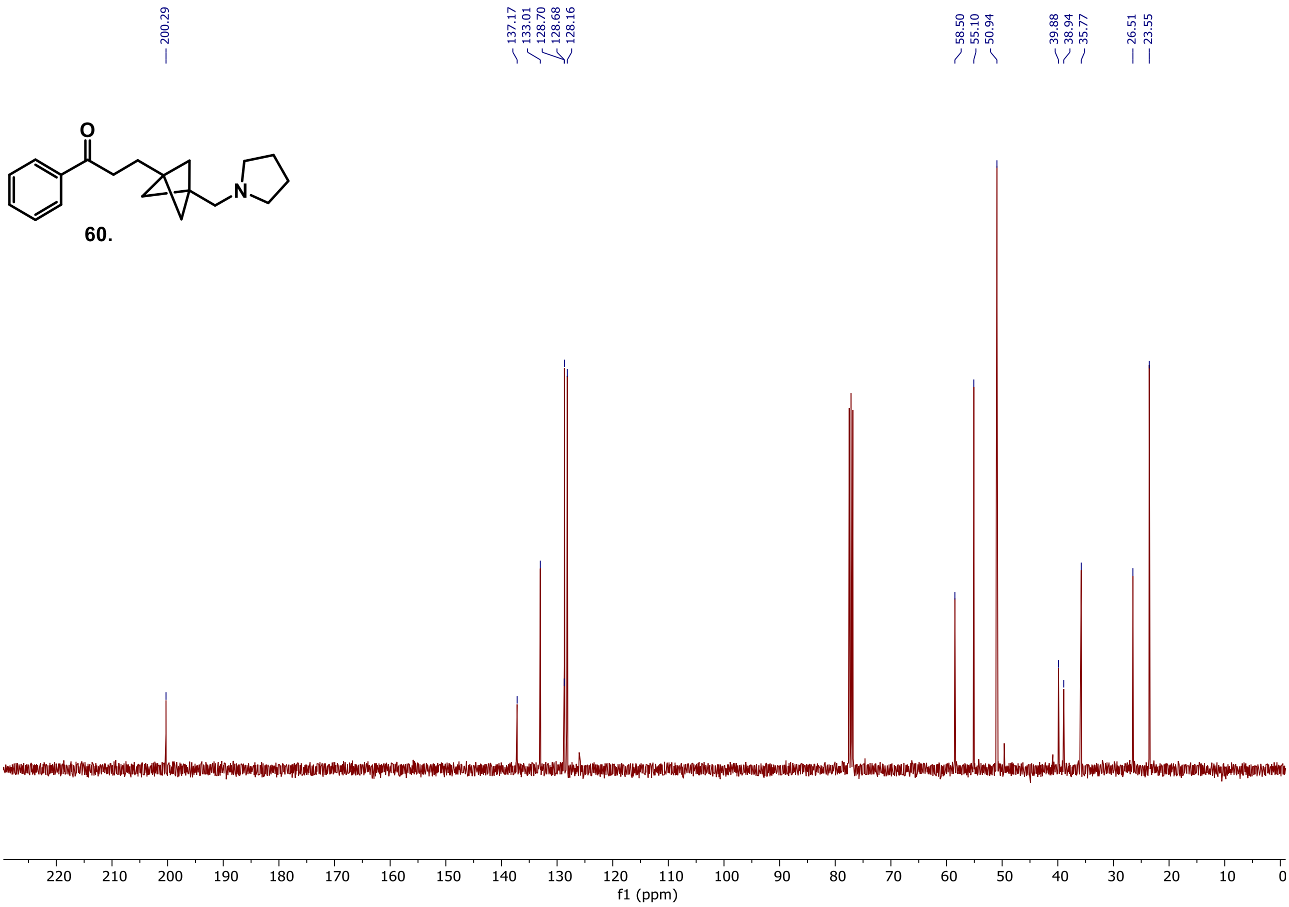

61: ^{1}H NMR (500 MHz, $CDCl_3$) and **^{13}C NMR** (126 MHz, $CDCl_3$) respectively.

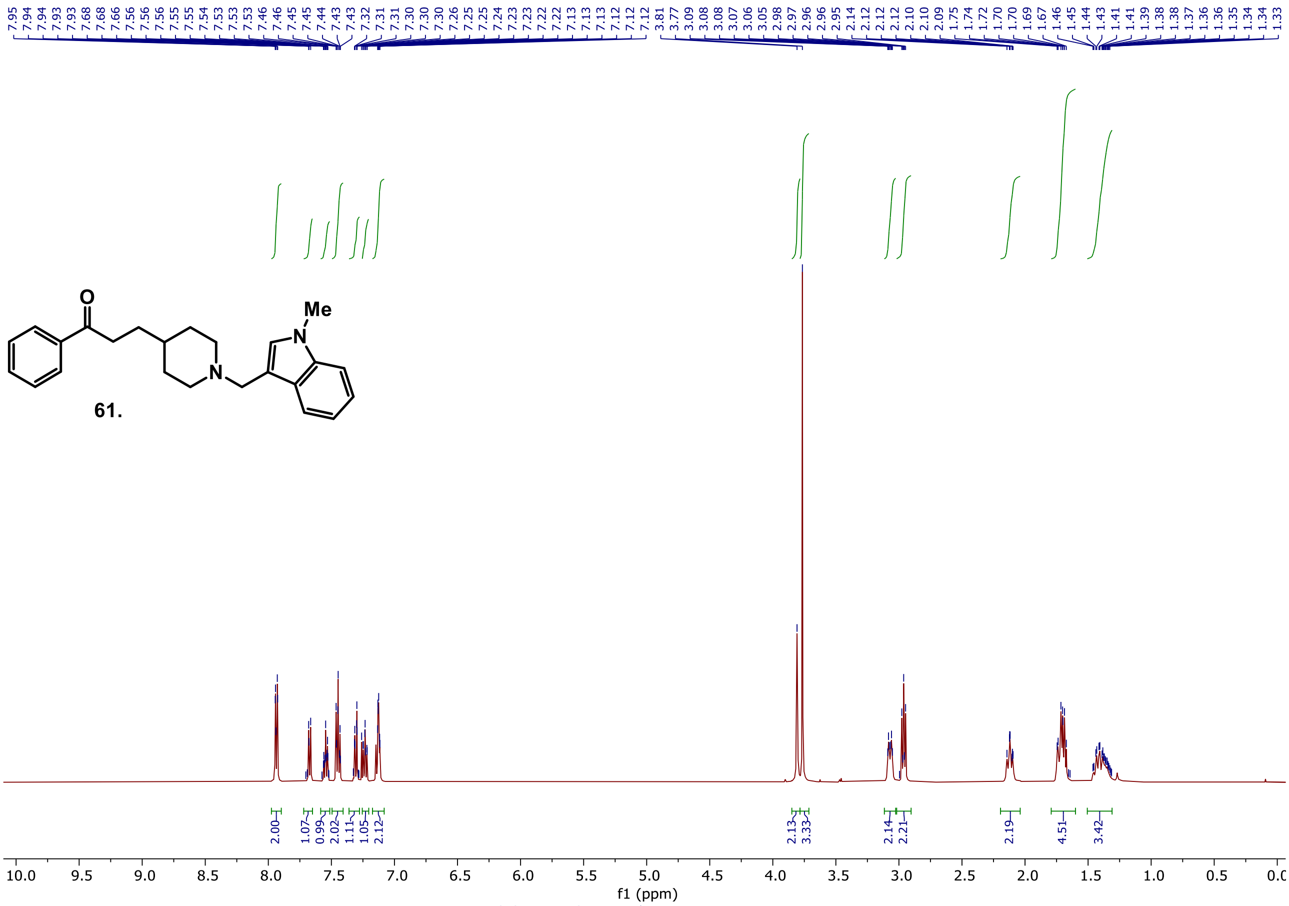

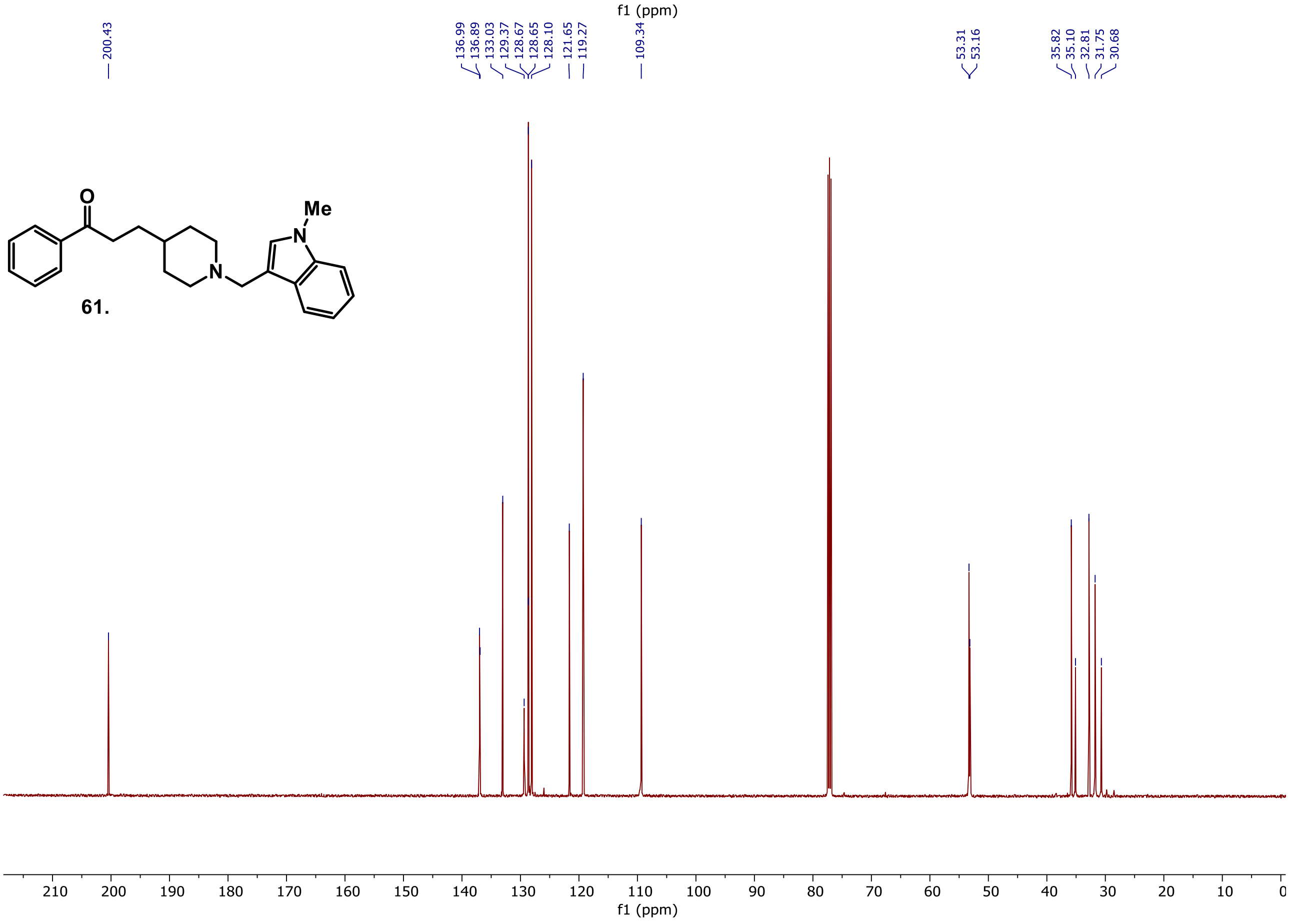

62: ^{1}H NMR (500 MHz, $CDCl_3$) and **^{13}C NMR** (126 MHz, $CDCl_3$) respectively.

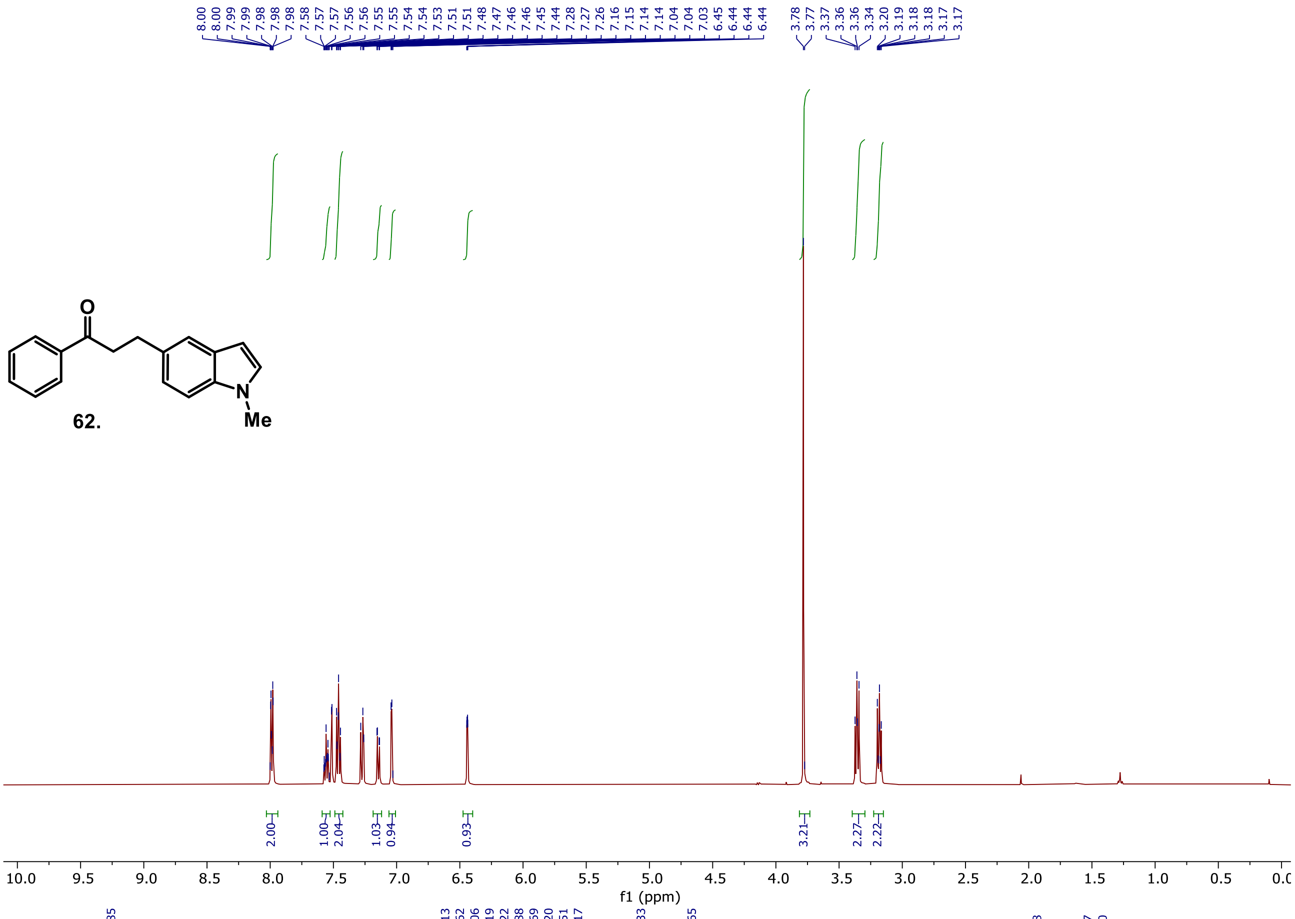

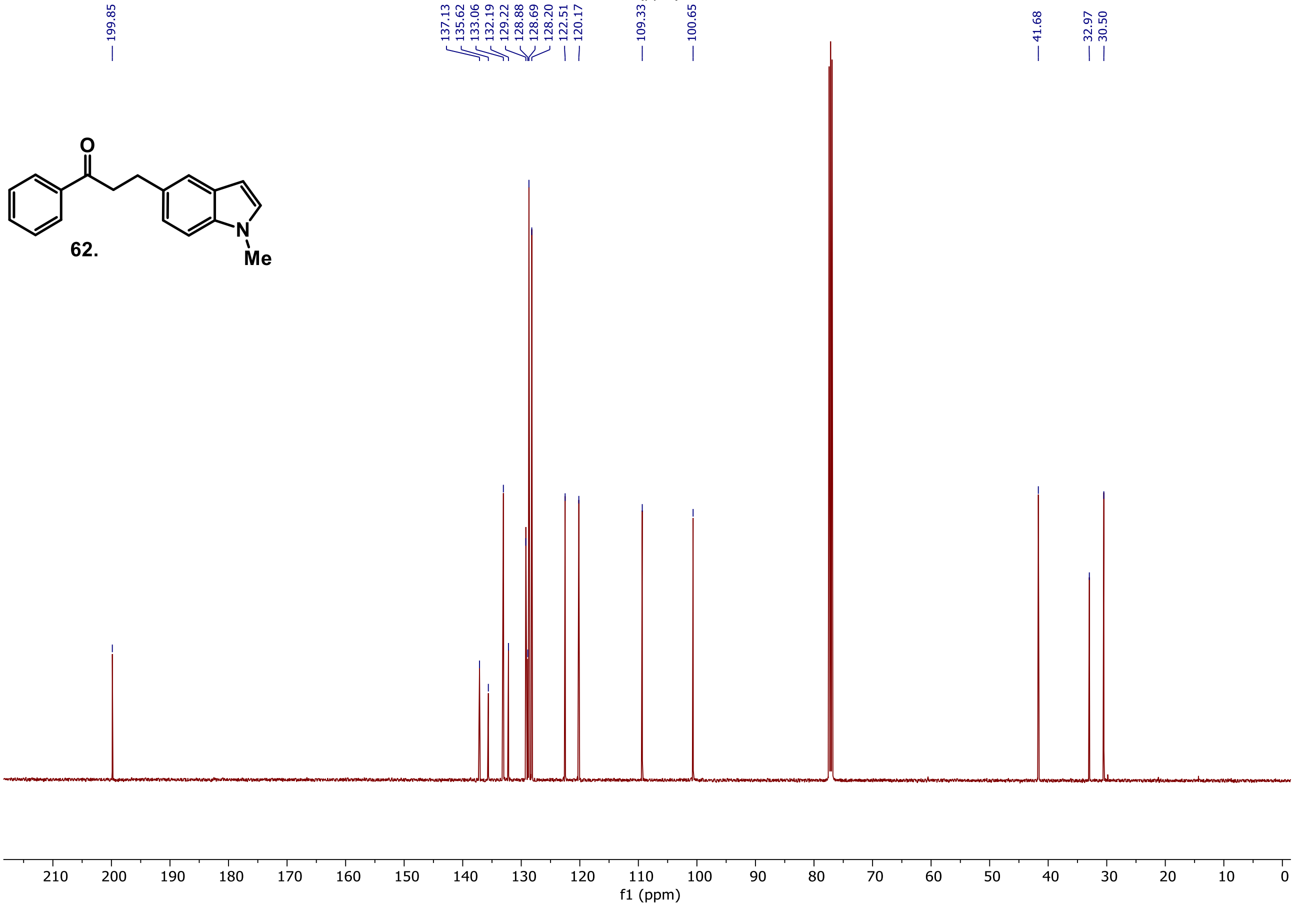

63: ^{1}H NMR (500 MHz, $CDCl_3$) and **^{13}C NMR** (126 MHz, $CDCl_3$) respectively.

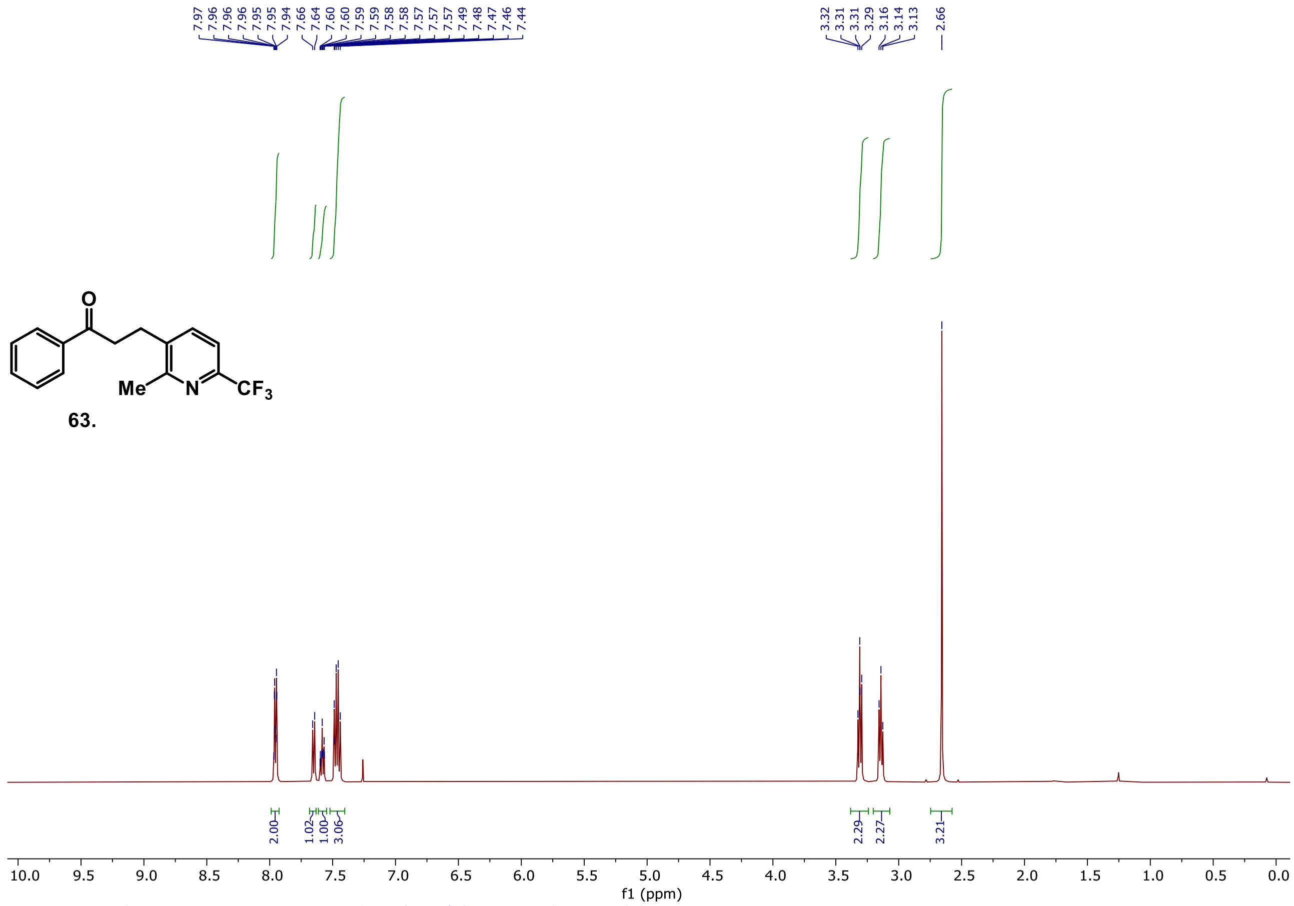

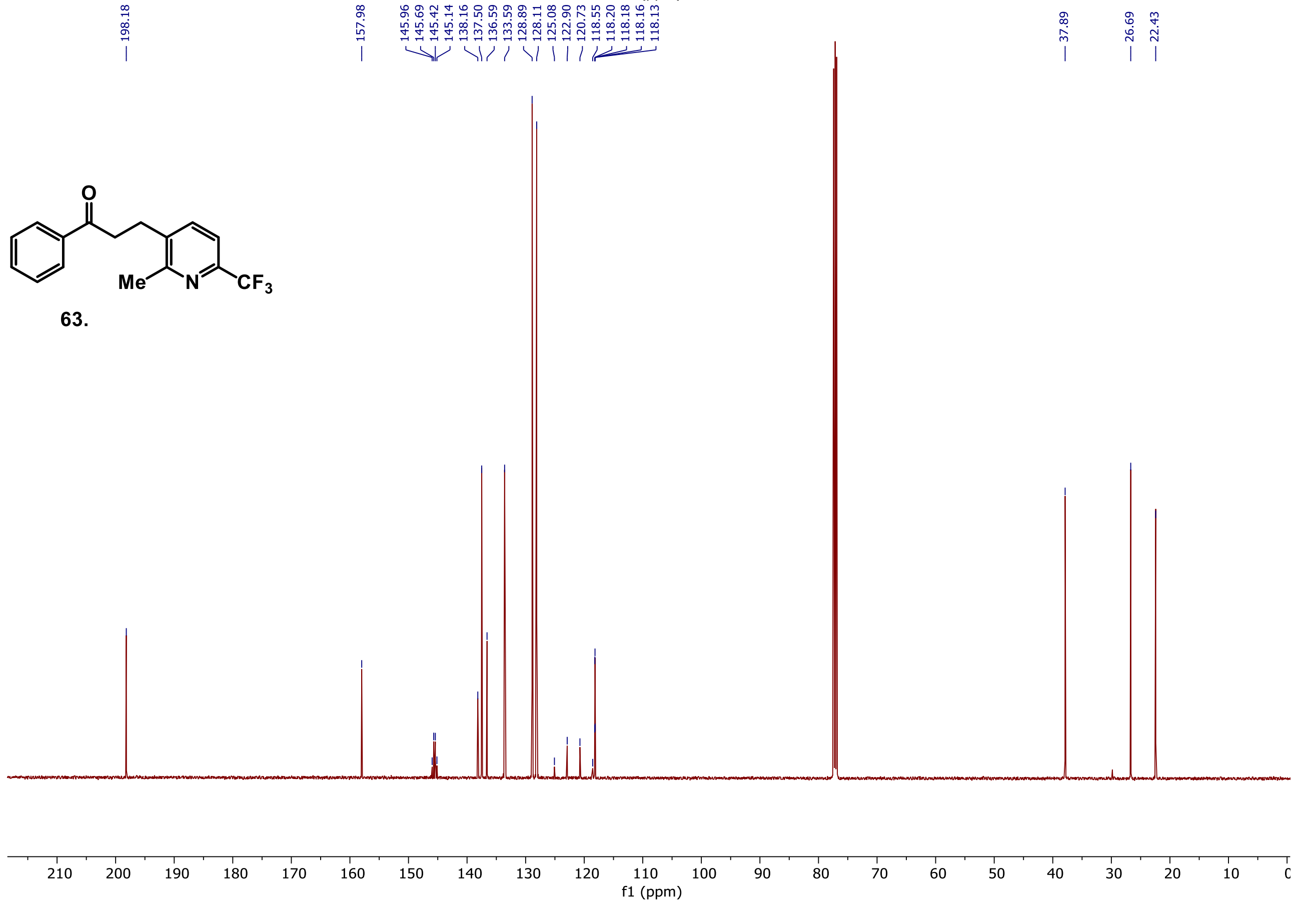

63: **^{19}F NMR** (470 MHz, $CDCl_3$).

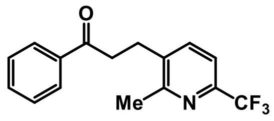

63.

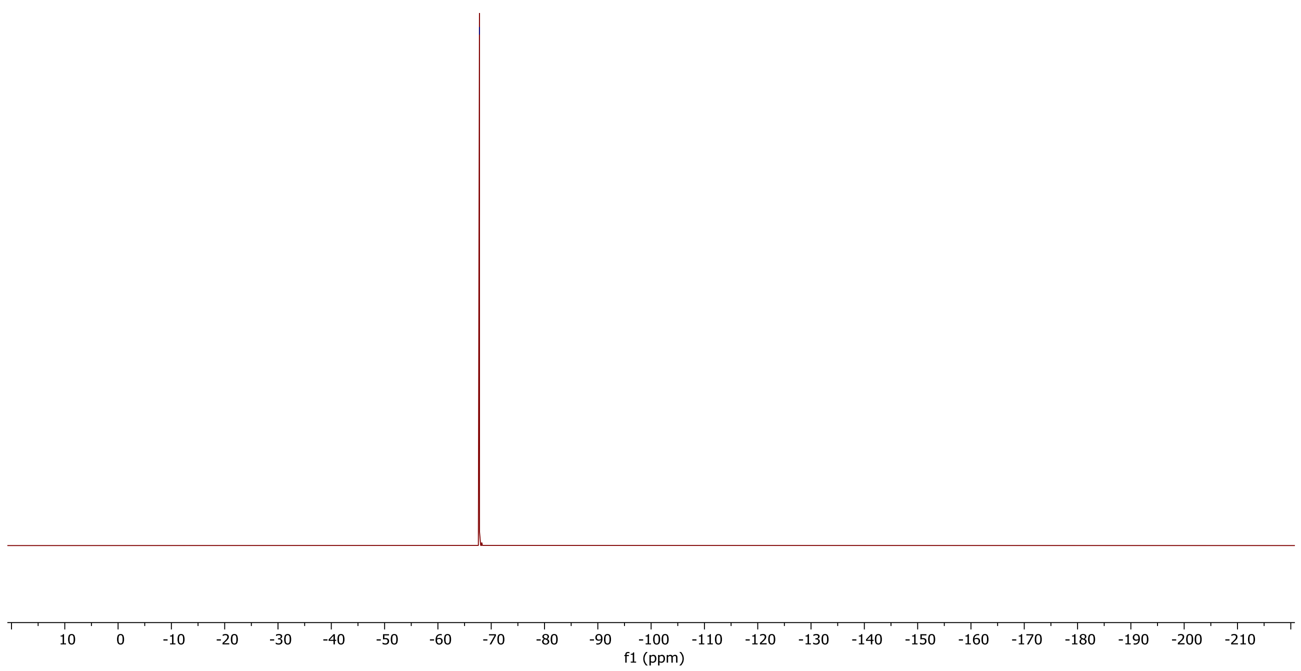

64: ^{1}H NMR (400 MHz, $CDCl_3$) and **^{13}C NMR** (101 MHz, $CDCl_3$) respectively.

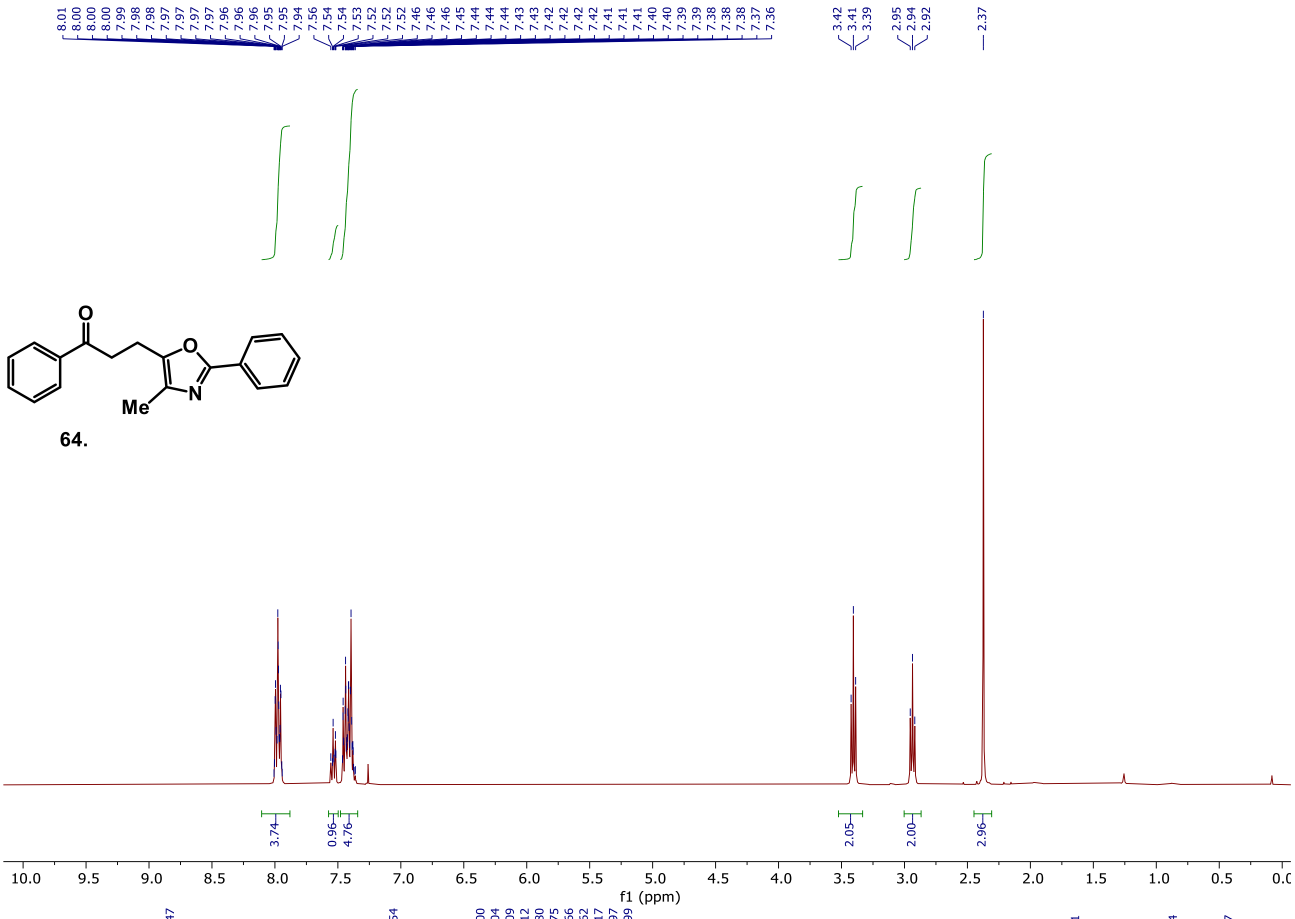

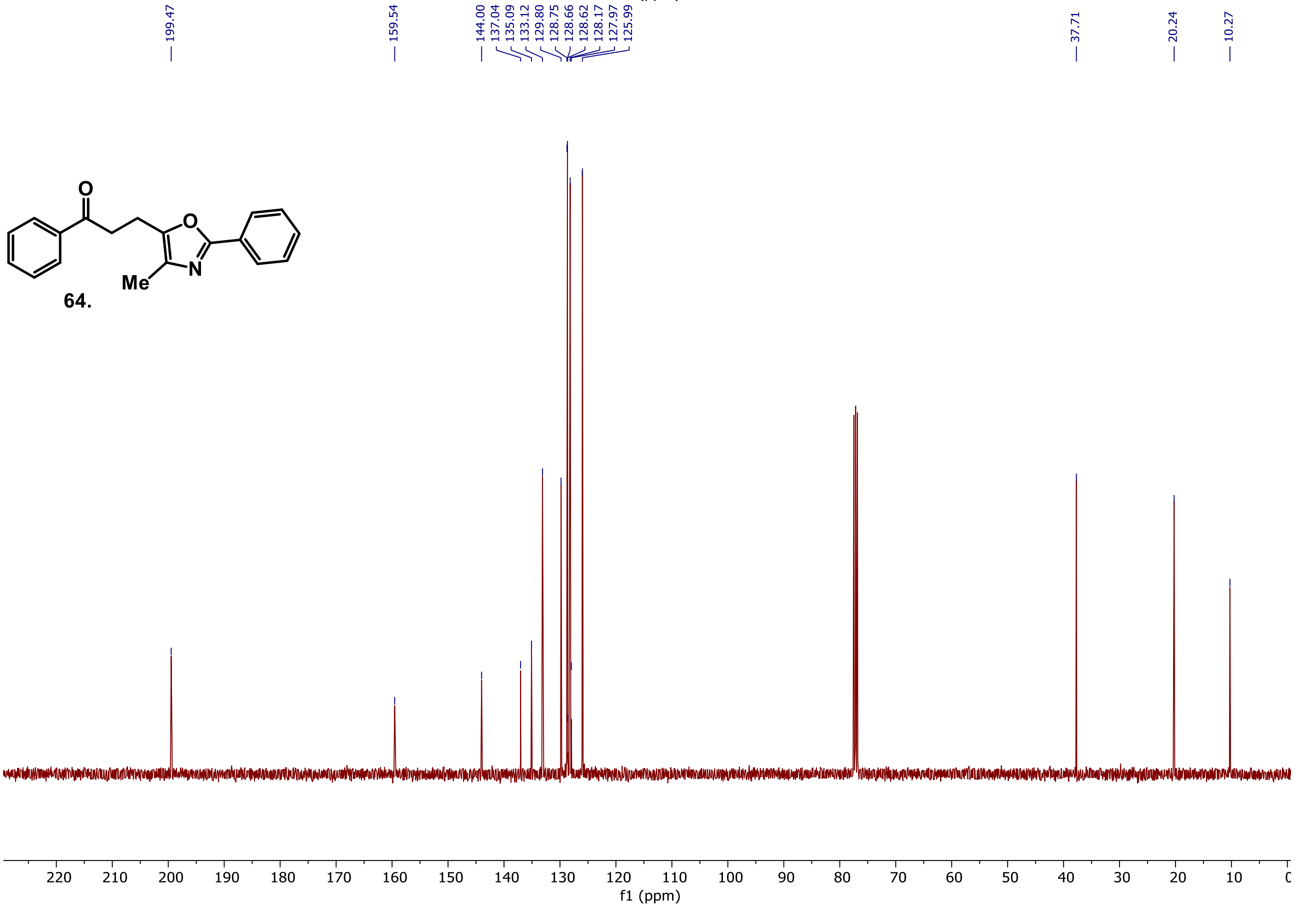

65: ¹H NMR (400 MHz, $CDCl_3$) and **¹³C NMR** (101 MHz, $CDCl_3$) respectively.

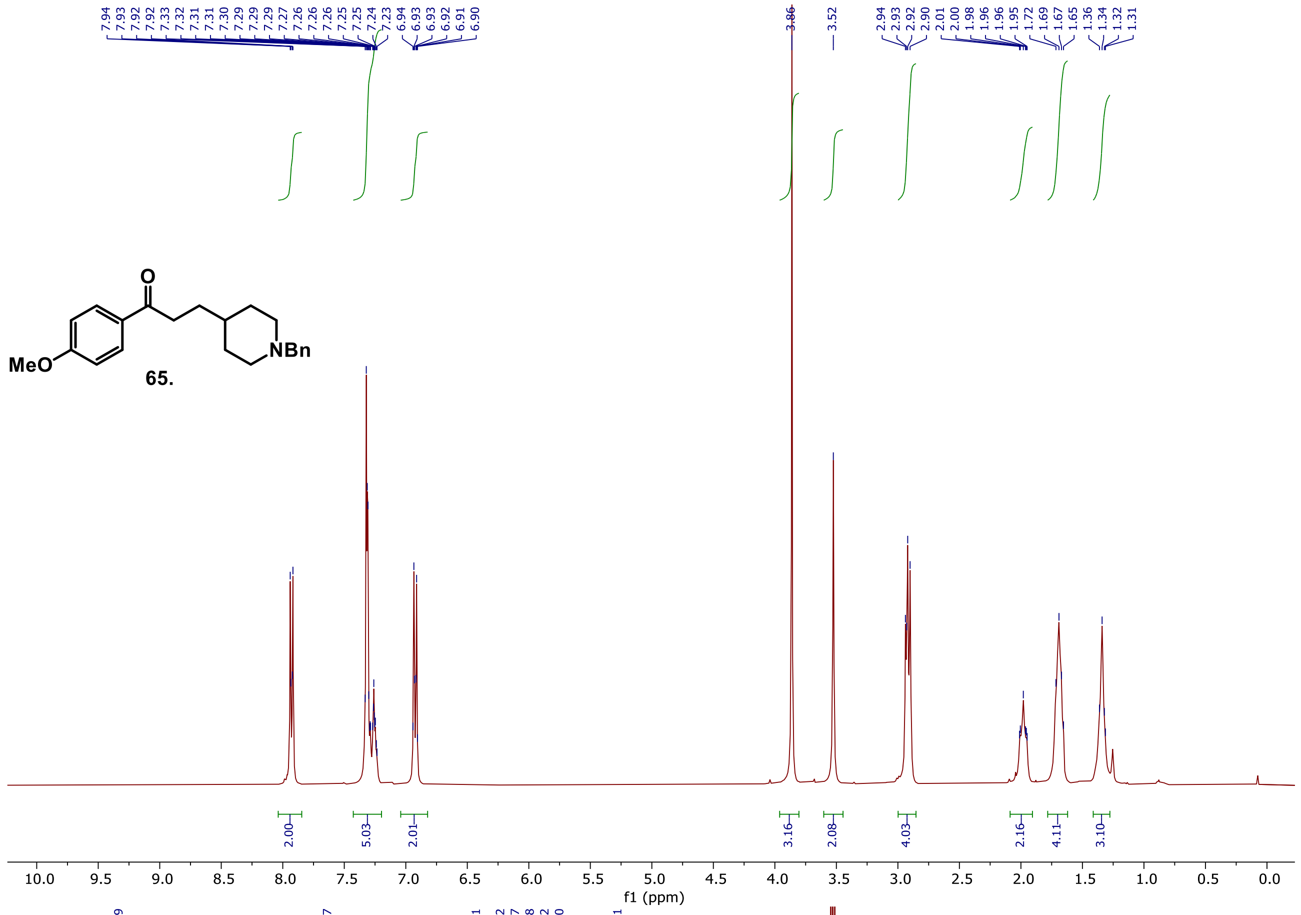

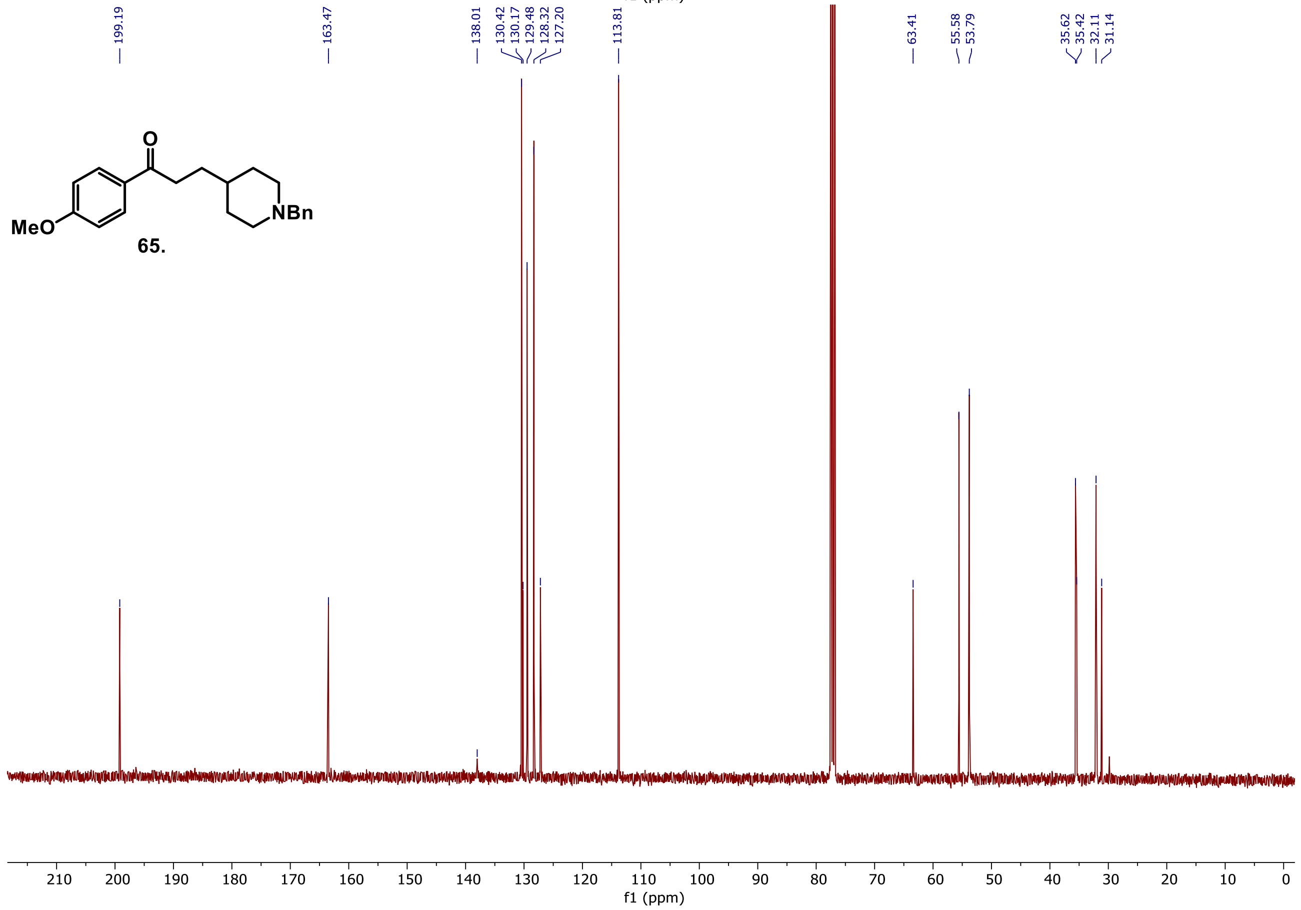

66: ¹H NMR (400 MHz, $CDCl_3$) and **¹³C NMR** (101 MHz, $CDCl_3$) respectively.

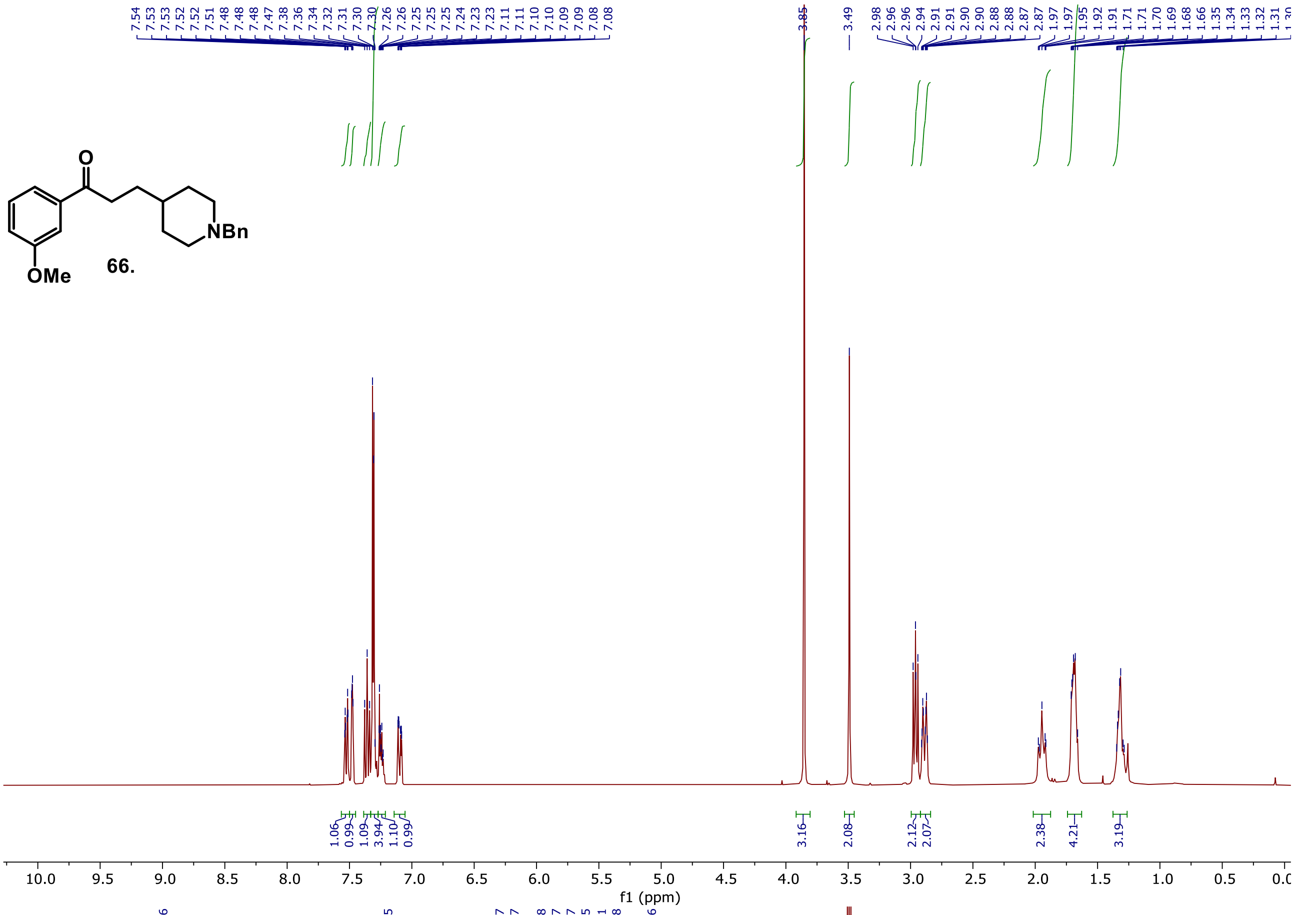

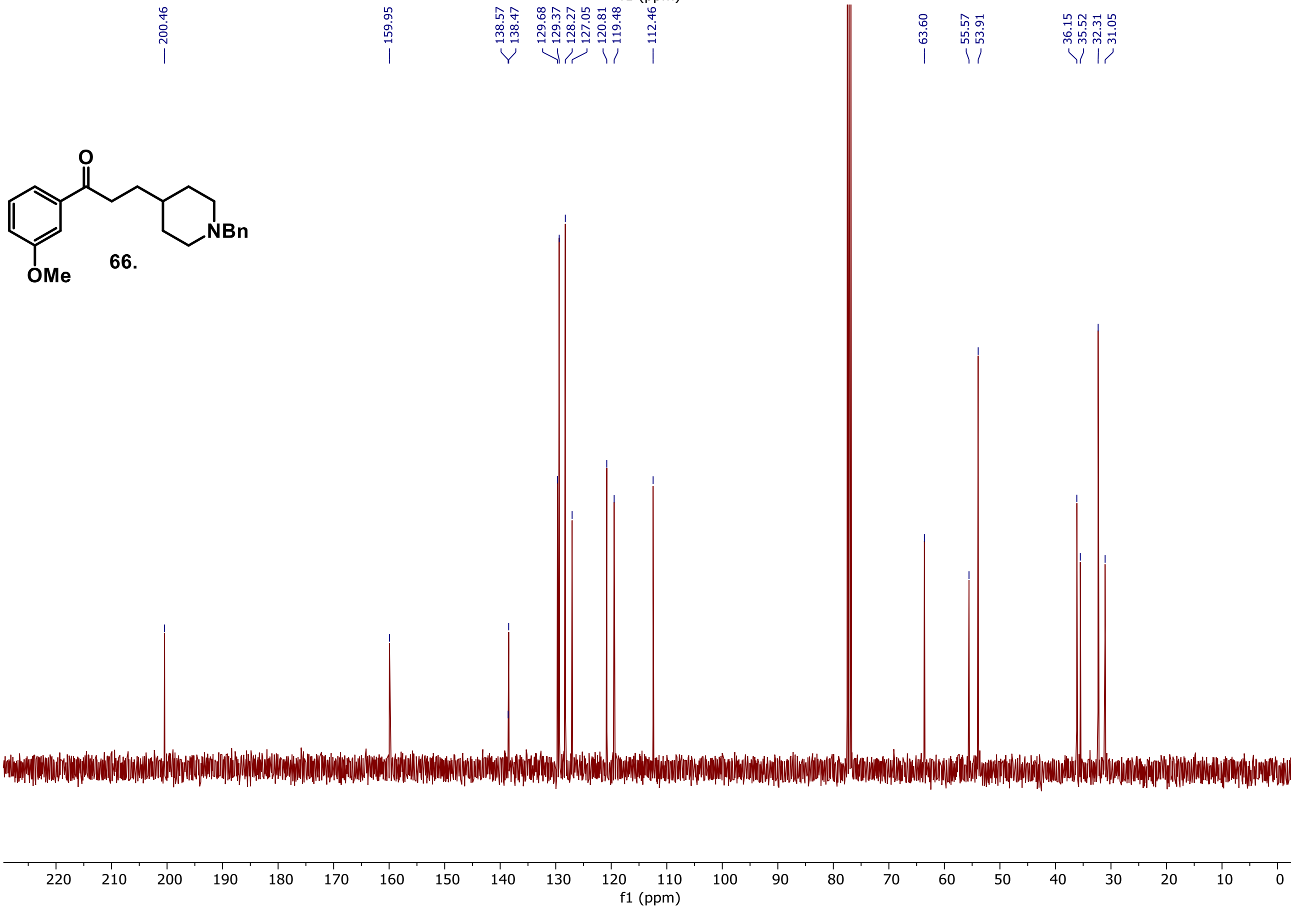

67: ^{1}H NMR (400 MHz, $CDCl_3$) and **^{13}C NMR** (126 MHz, $CDCl_3$) respectively.

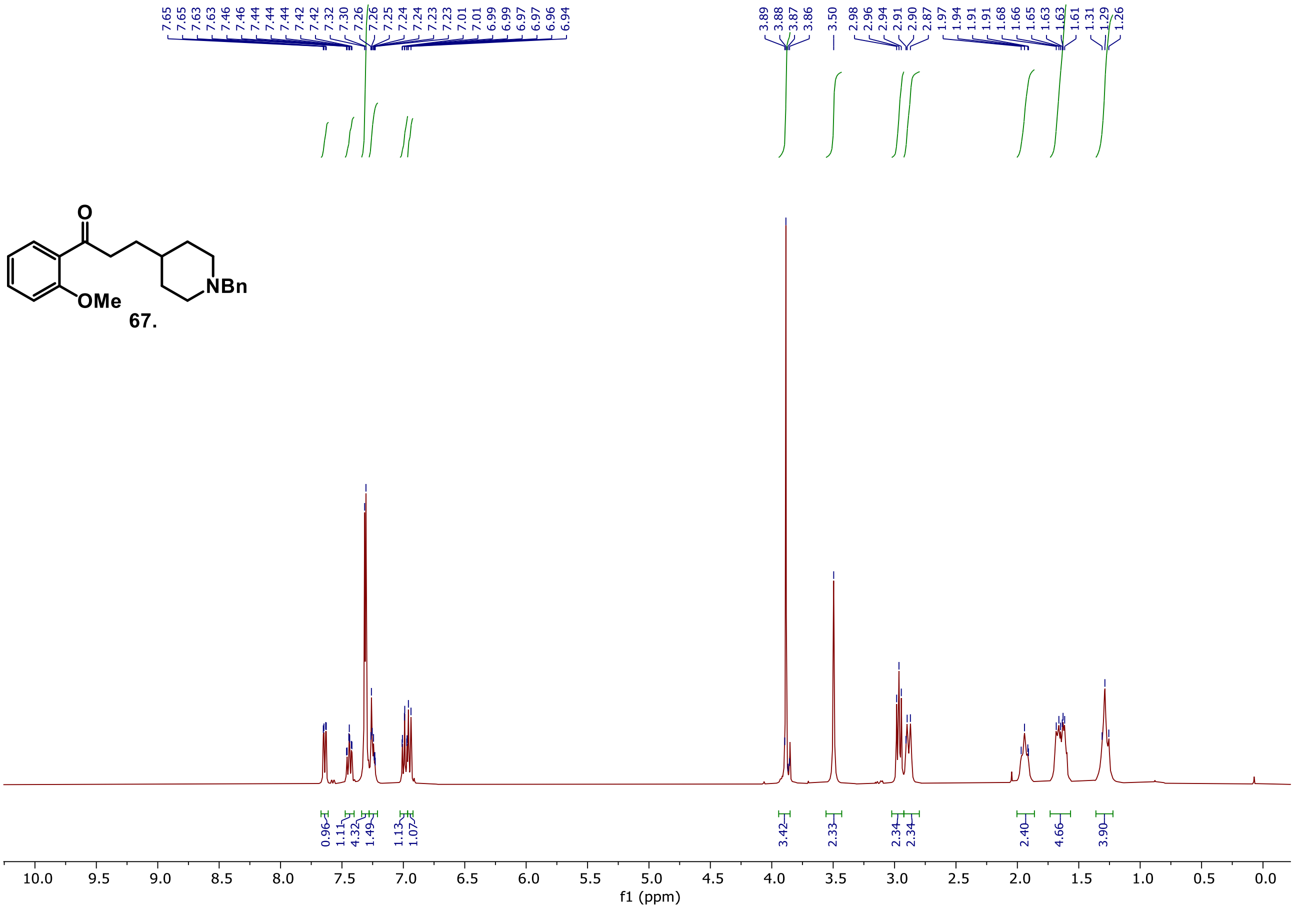

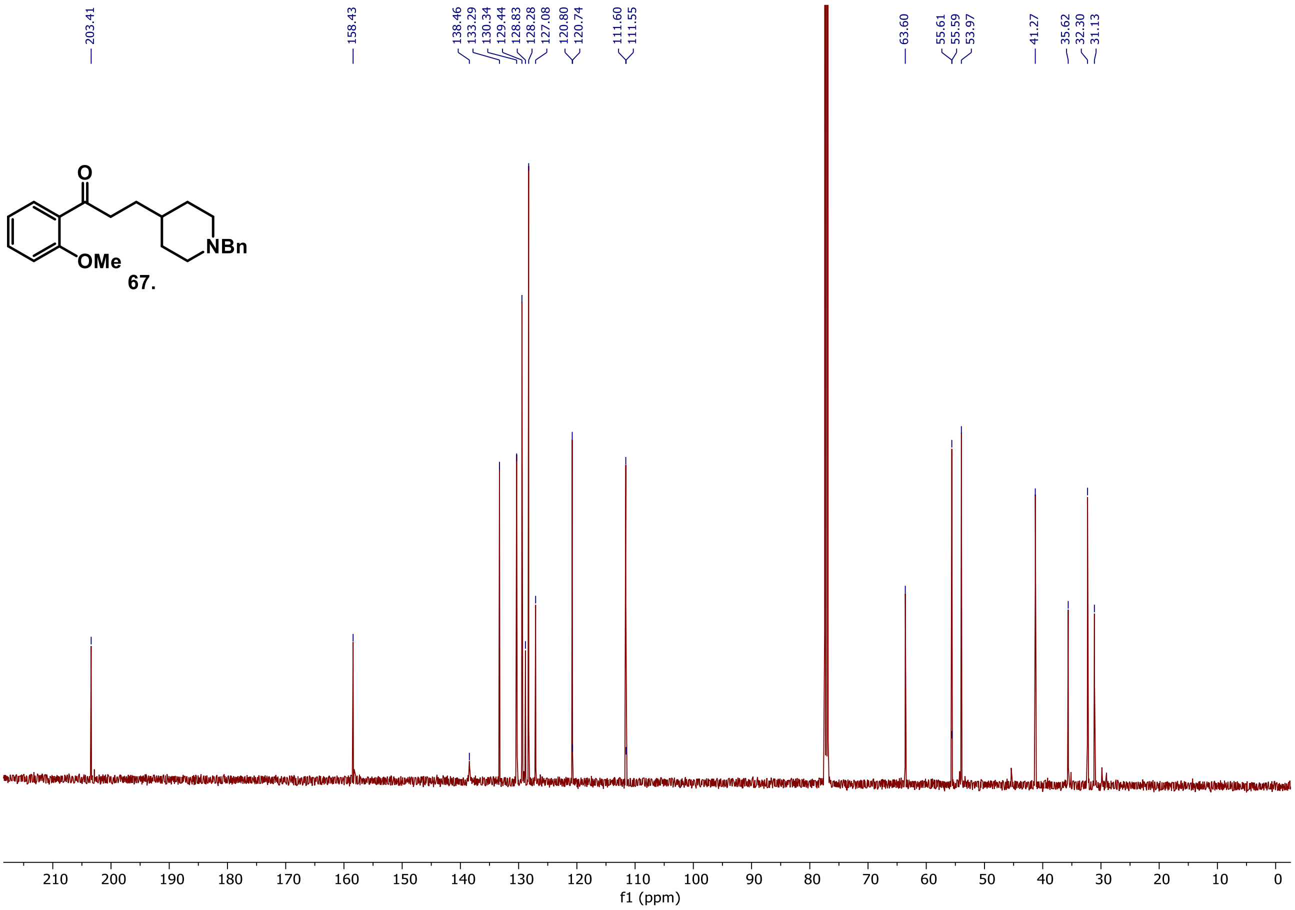

68: ^{1}H NMR (400 MHz, $CDCl_3$) and **^{13}C NMR** (101 MHz, $CDCl_3$) respectively.

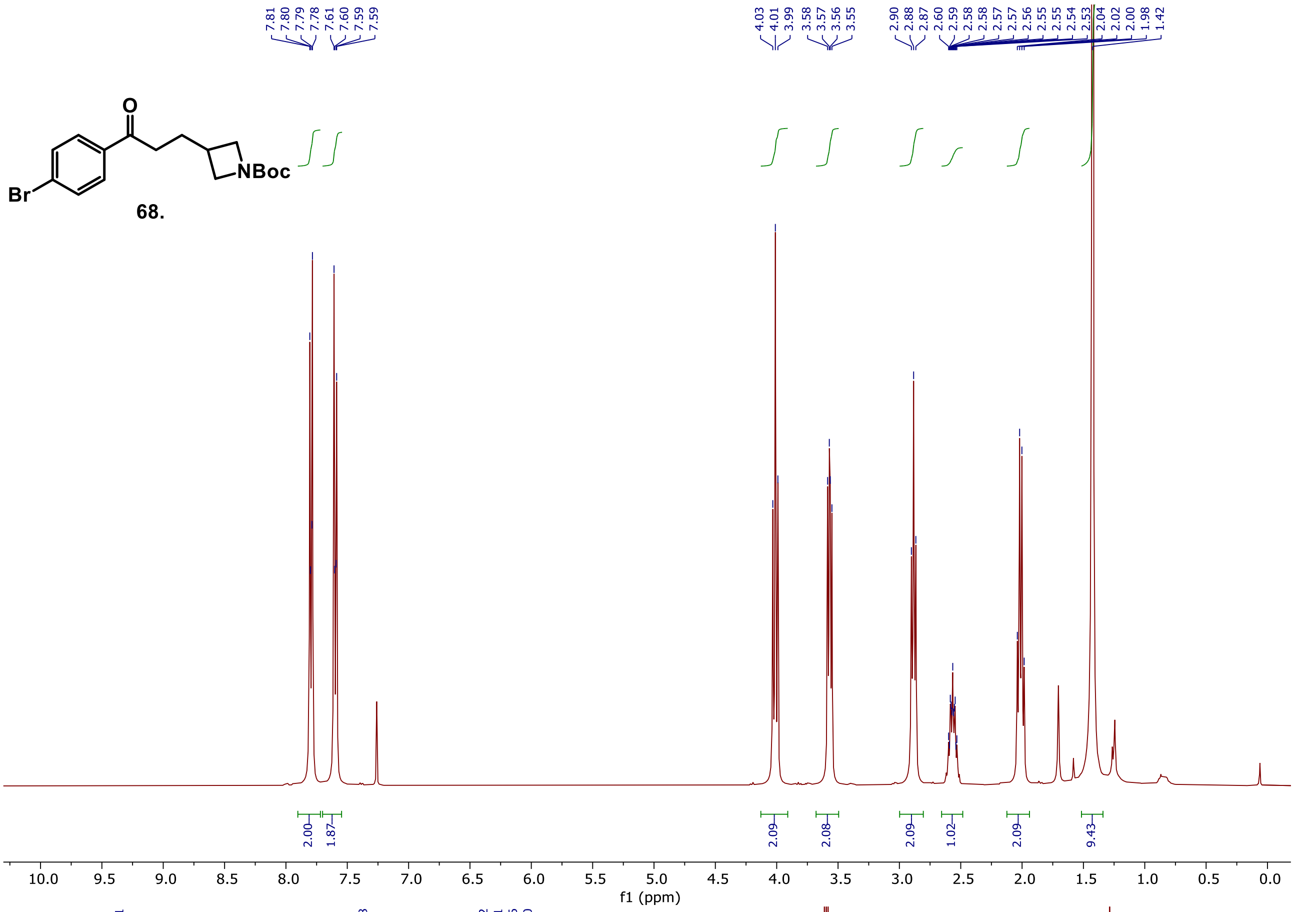

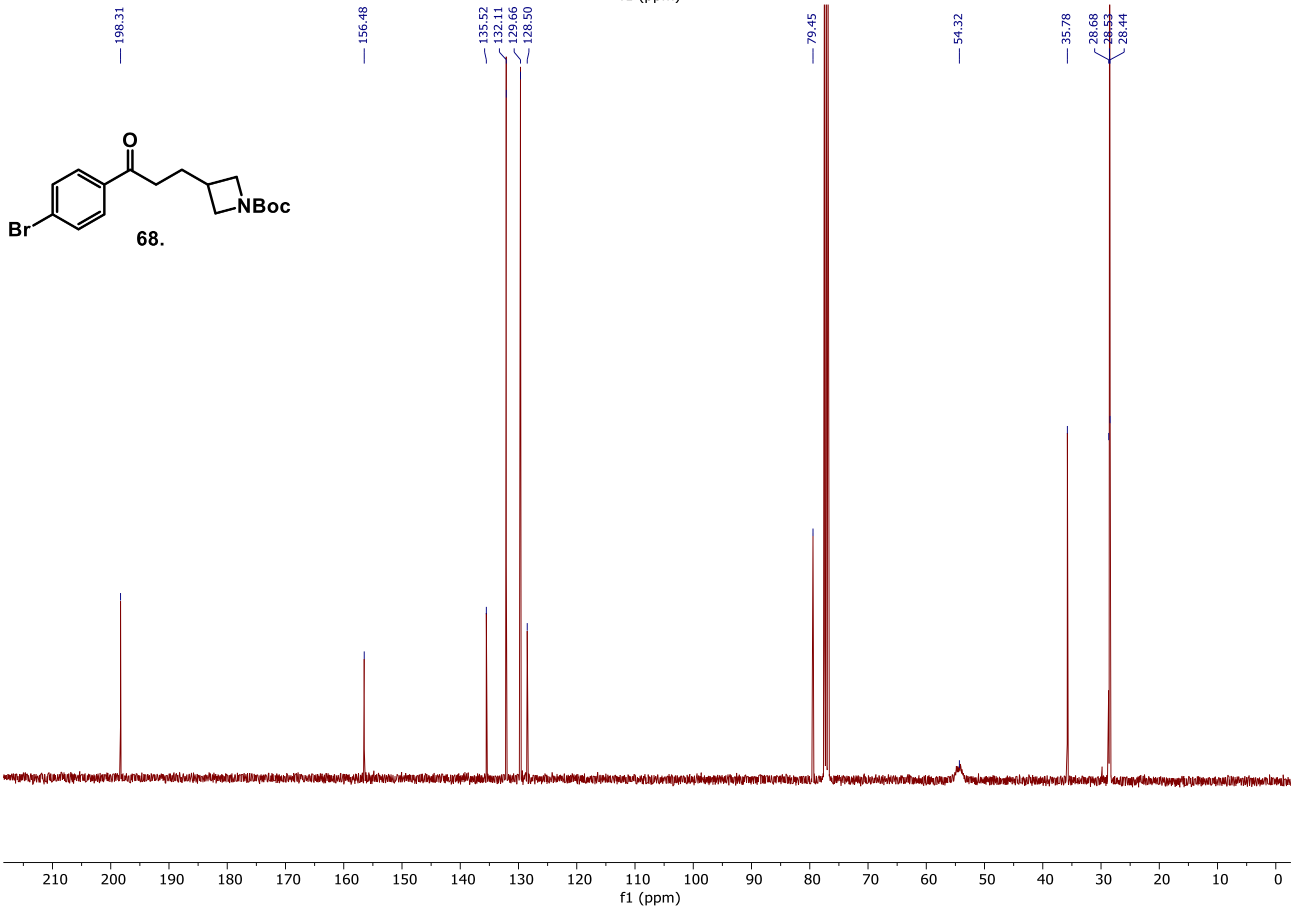

69: ¹H NMR (400 MHz, $CDCl_3$) and **¹³C NMR** (101 MHz, $CDCl_3$) respectively.

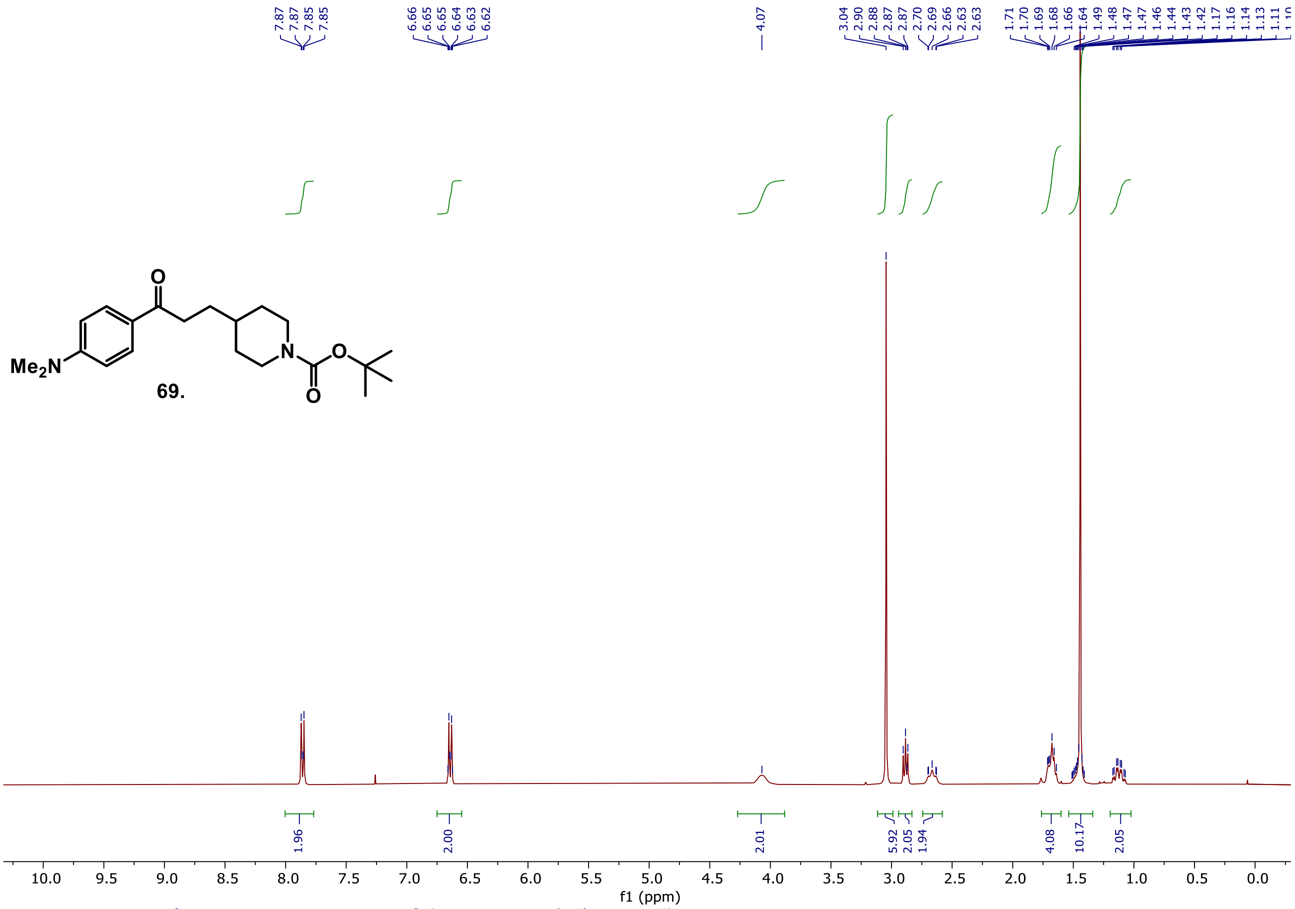

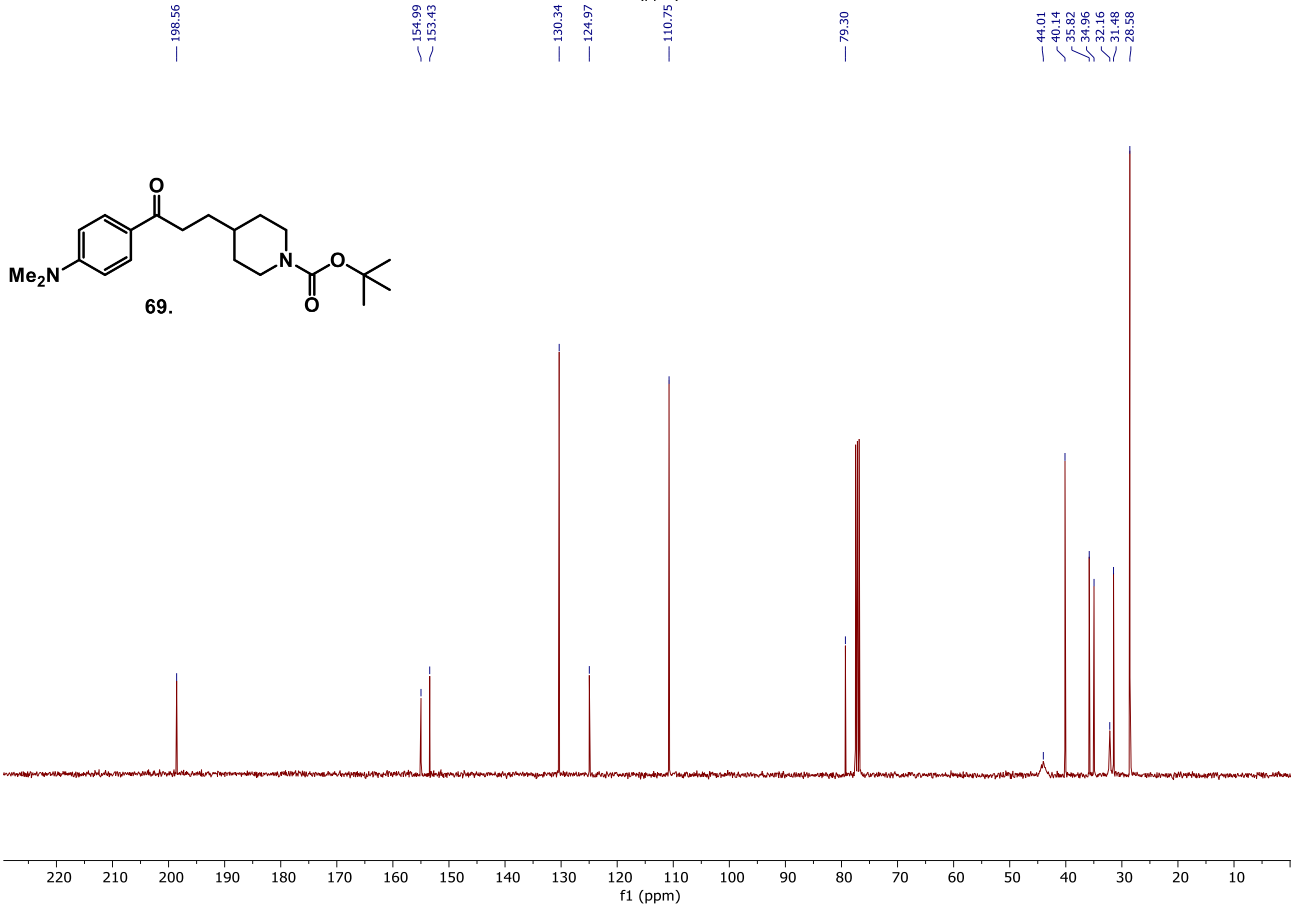

70: ¹H NMR (400 MHz, $CDCl_3$) and **¹³C NMR** (101 MHz, $CDCl_3$) respectively.

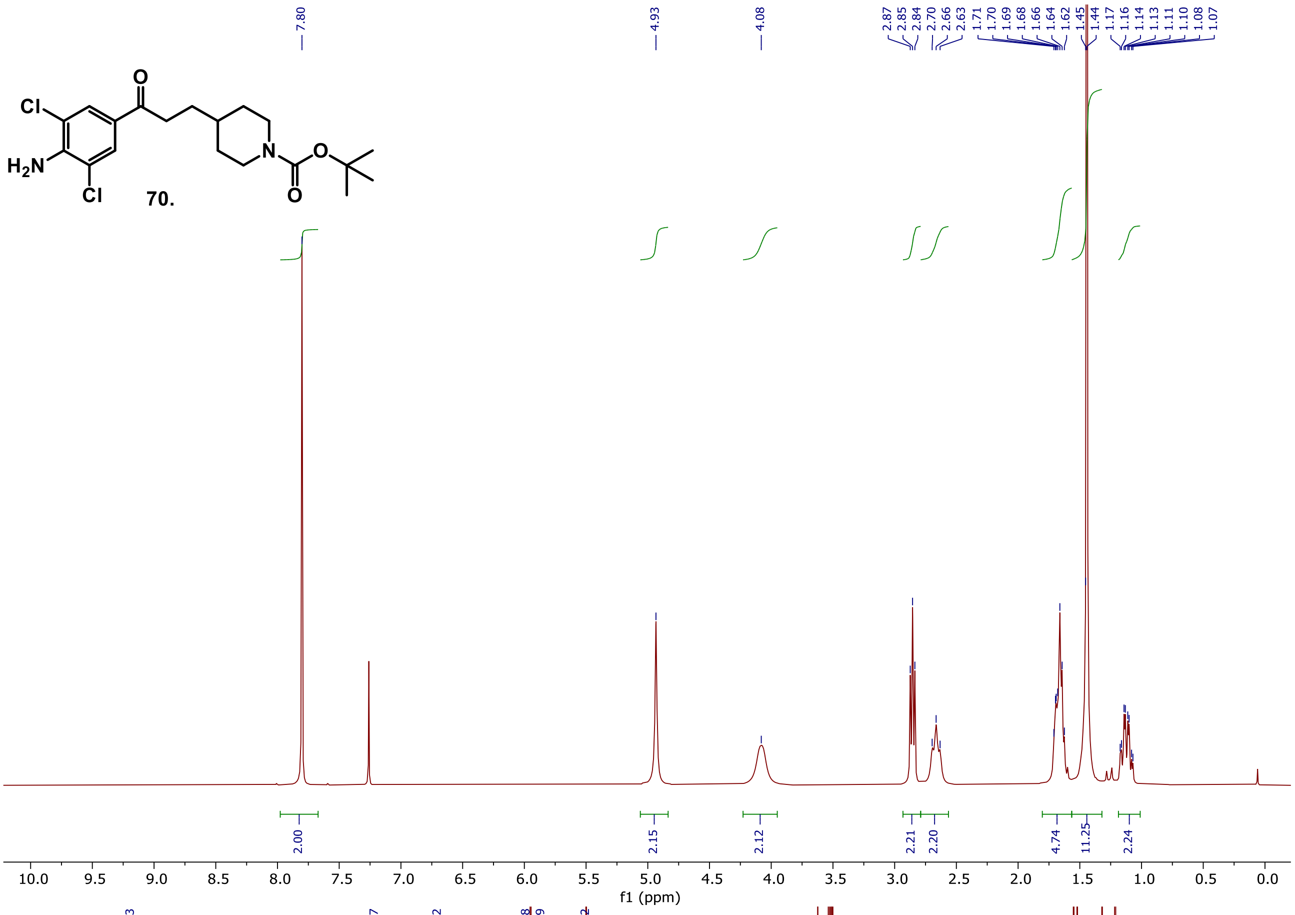

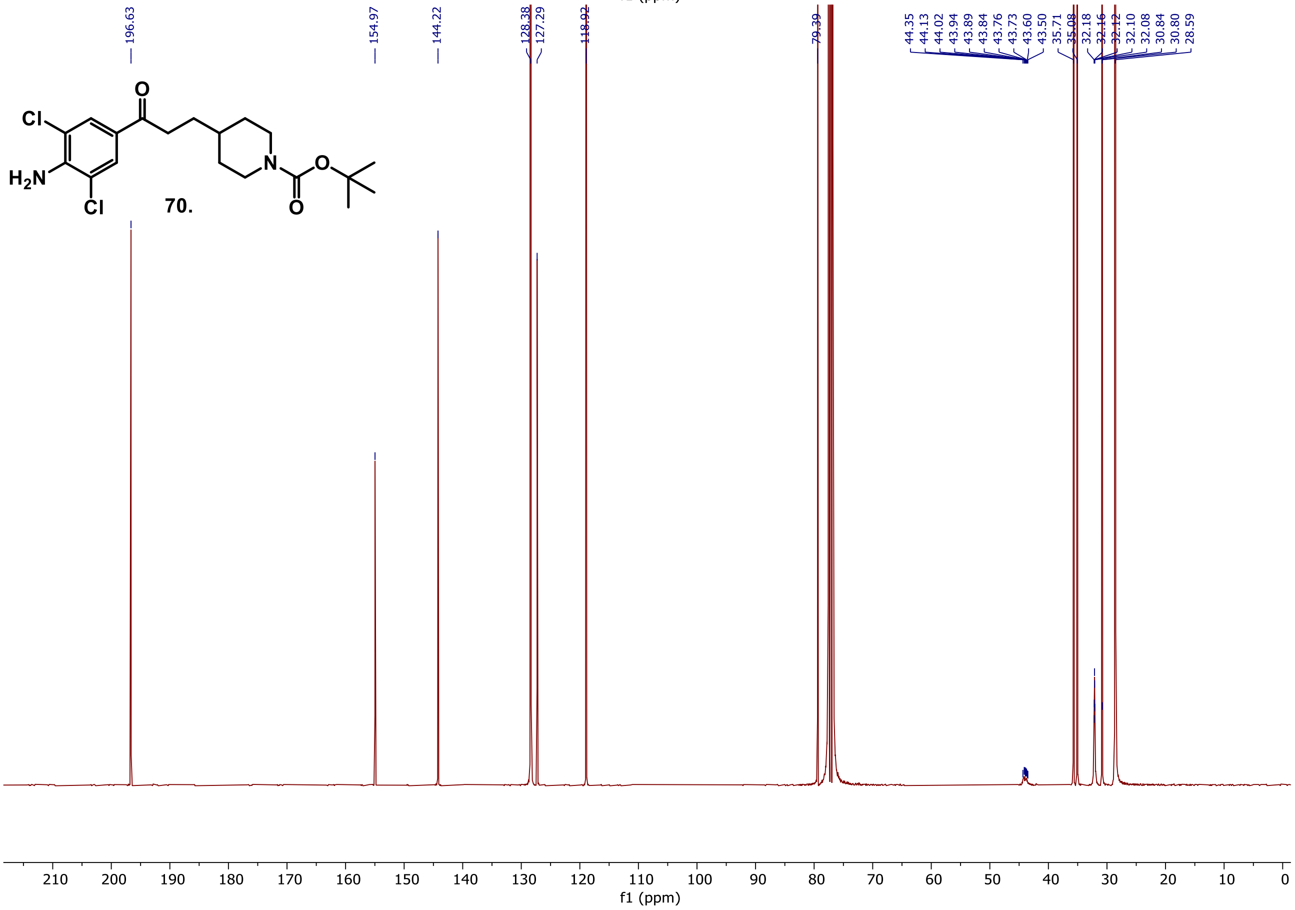

71: ^{1}H NMR (400 MHz, $CDCl_3$) and **^{13}C NMR** (101 MHz, $CDCl_3$) respectively.

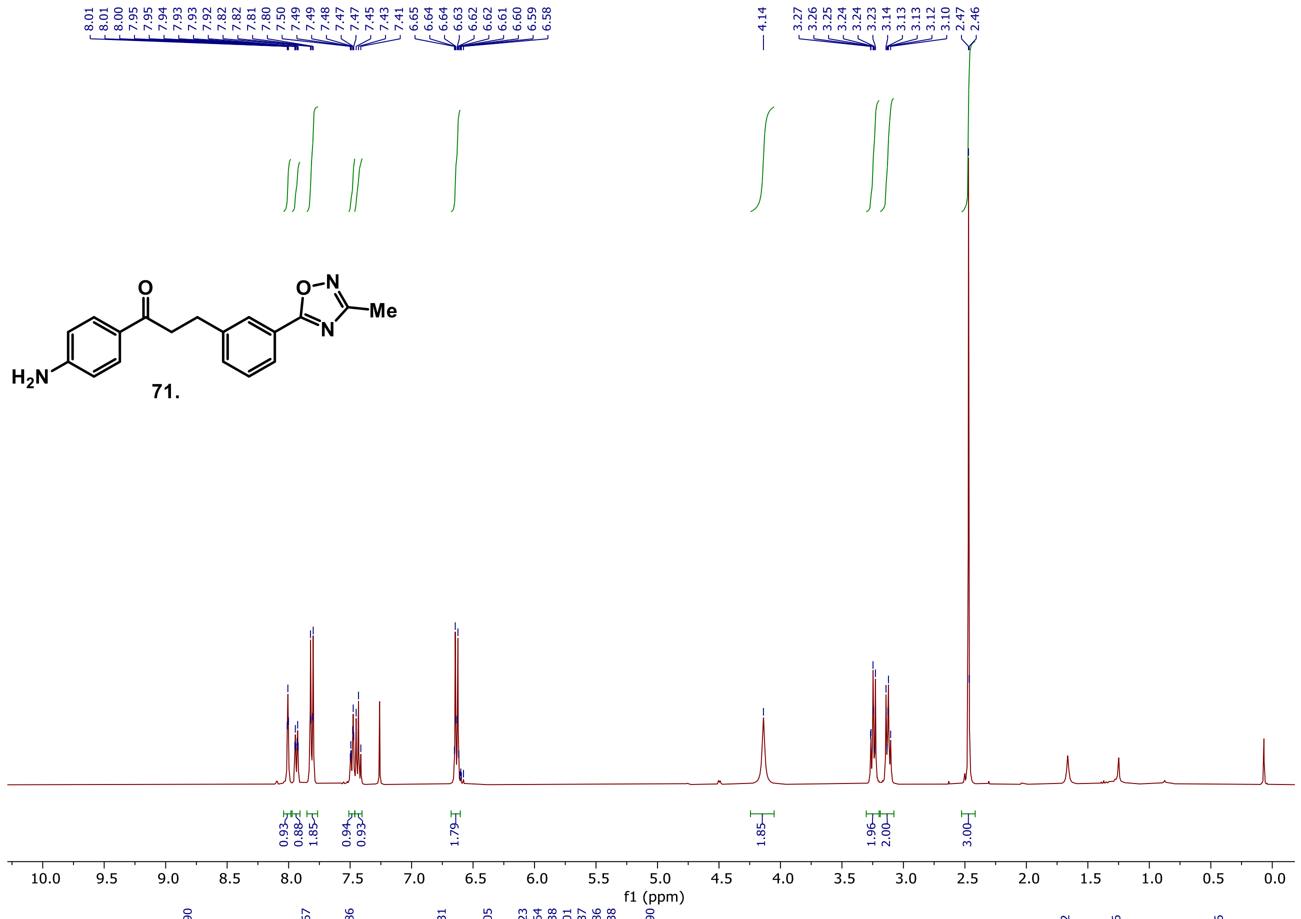

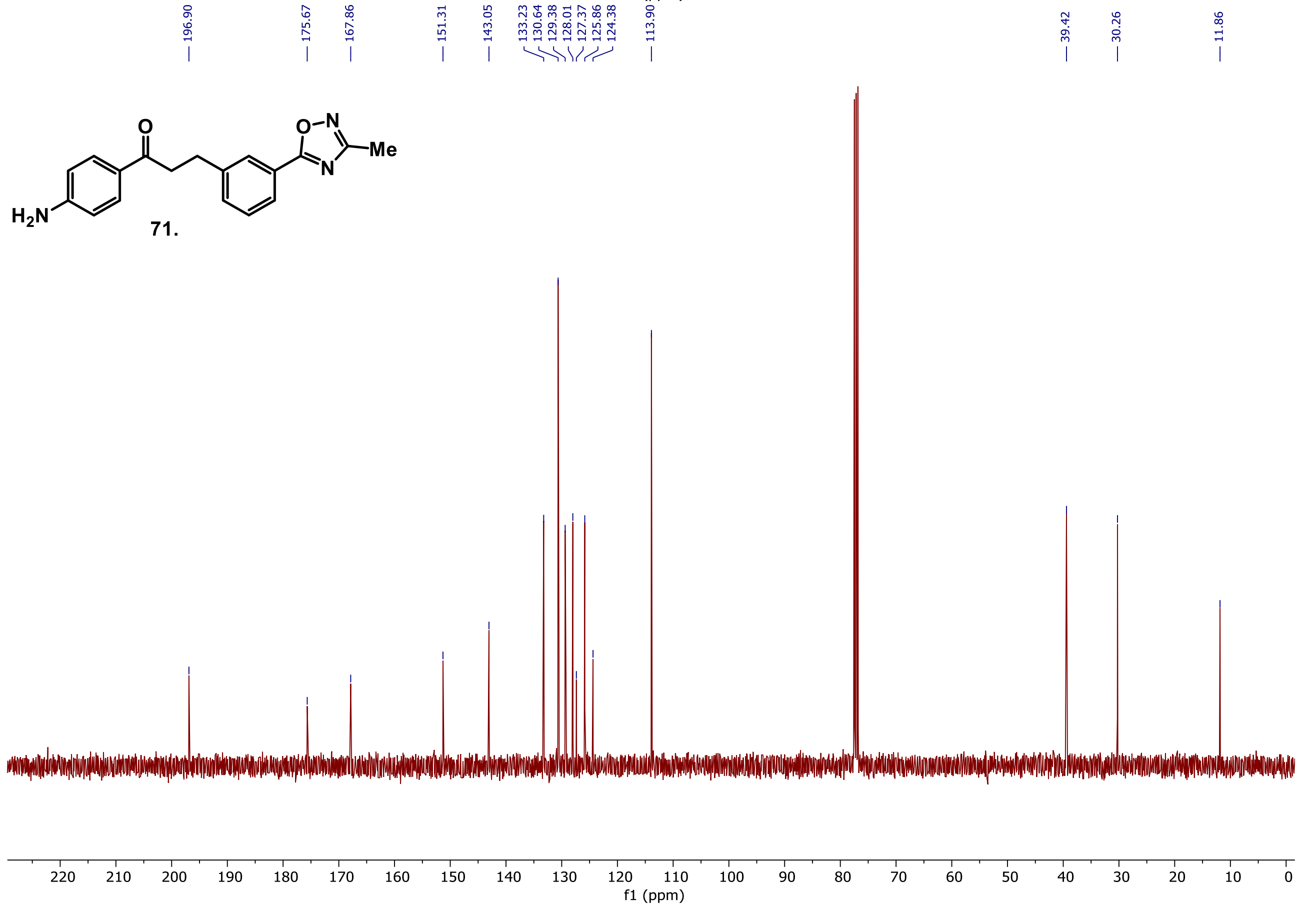

72: **^{1}H NMR** (400 MHz, $CDCl_3$) and **^{13}C NMR** (101 MHz, $CDCl_3$) respectively.

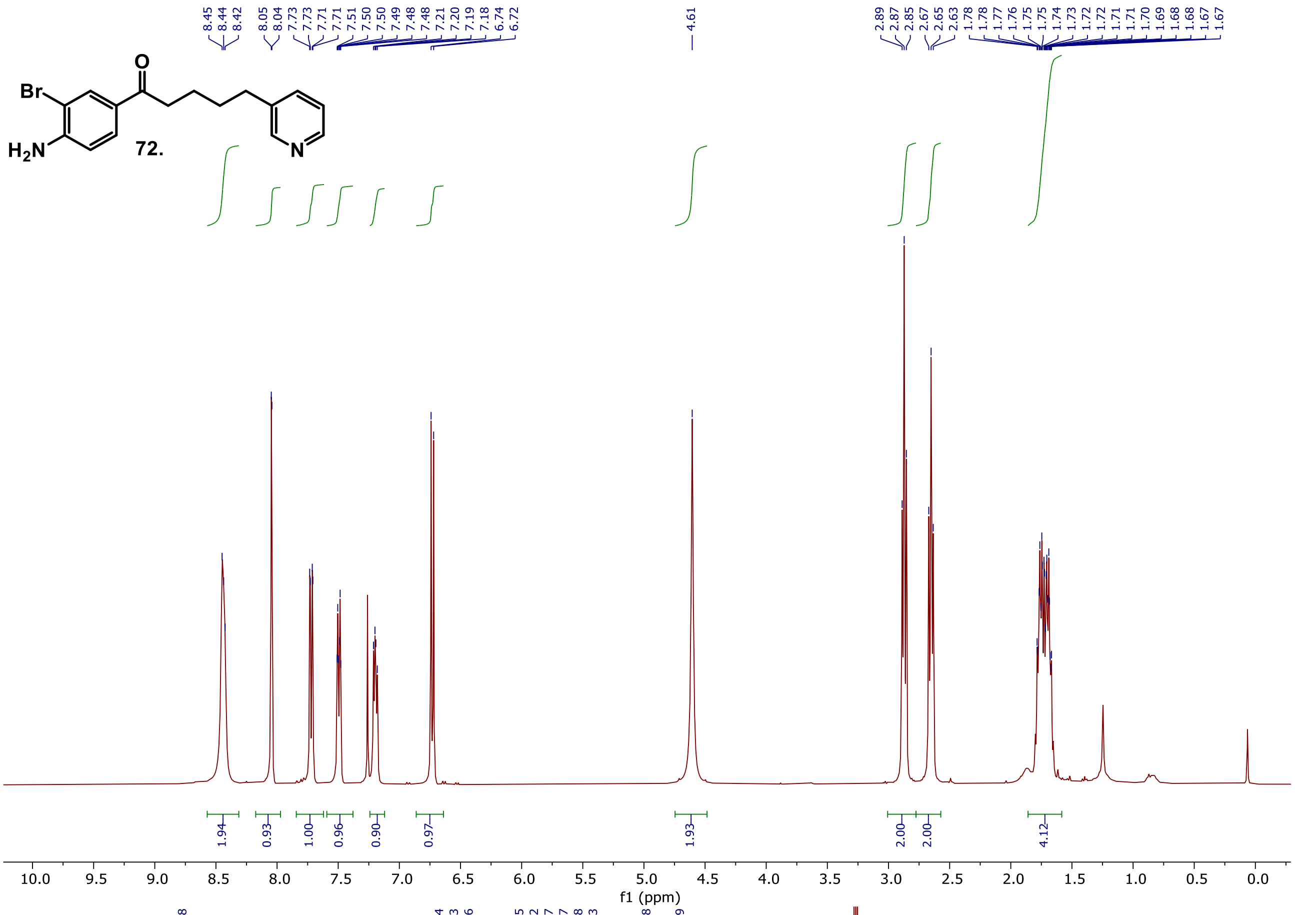

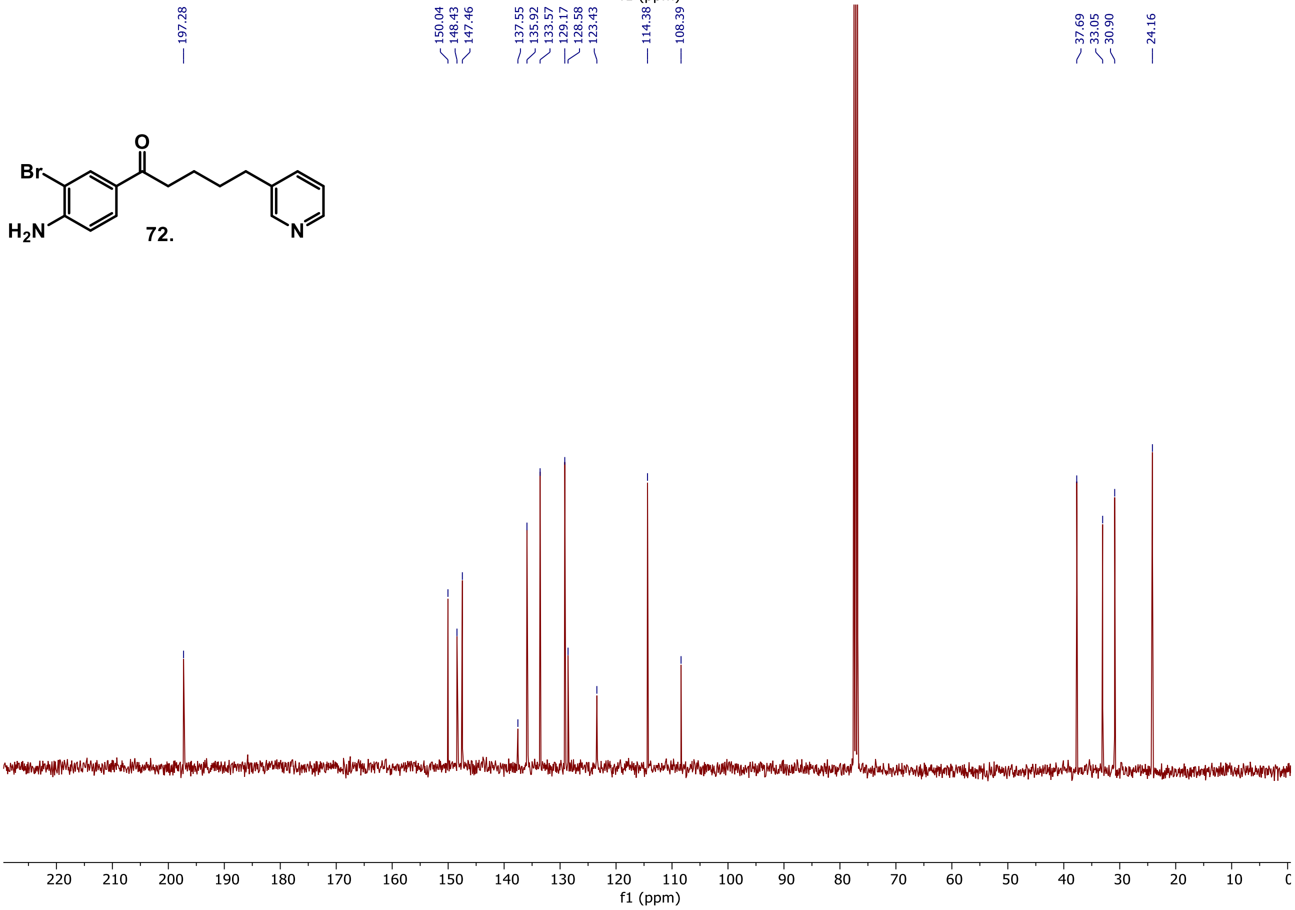

73: ^{1}H NMR (400 MHz, $CDCl_3$) and **^{13}C NMR** (101 MHz, $CDCl_3$) respectively.

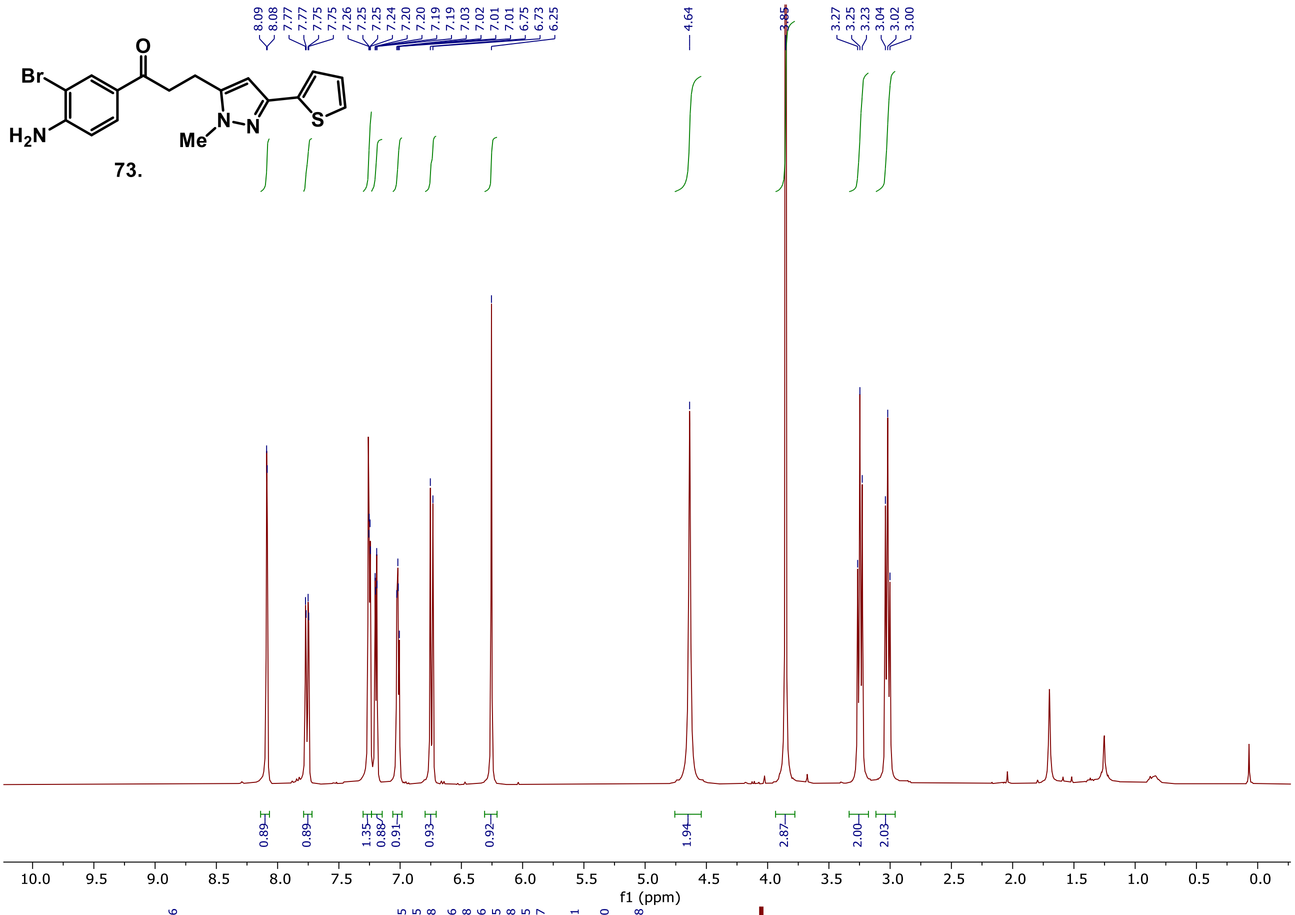

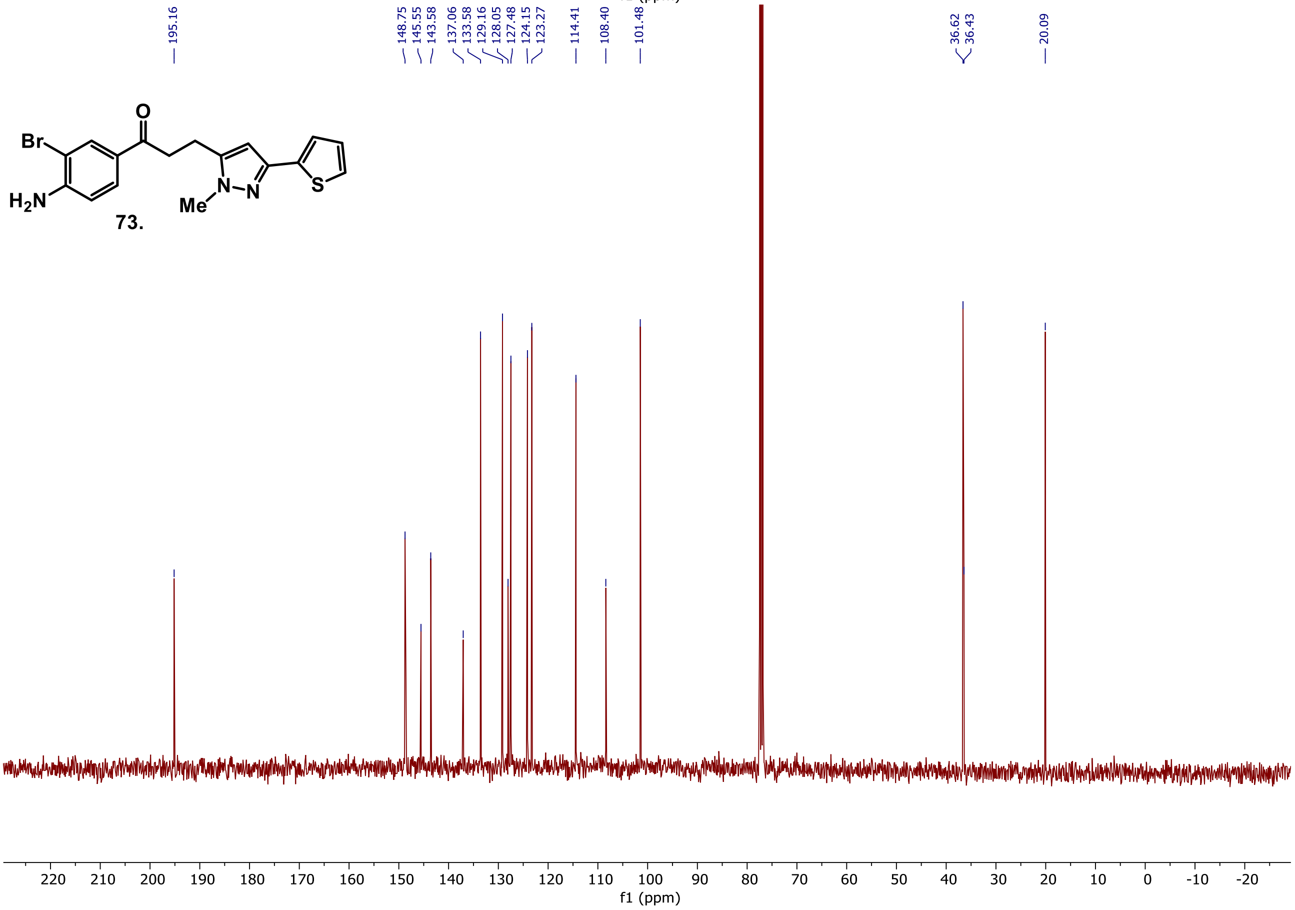

74: ^{1}**H NMR** (400 MHz, $CDCl_3$) and ^{13}**C NMR** (101 MHz, $CDCl_3$) respectively.

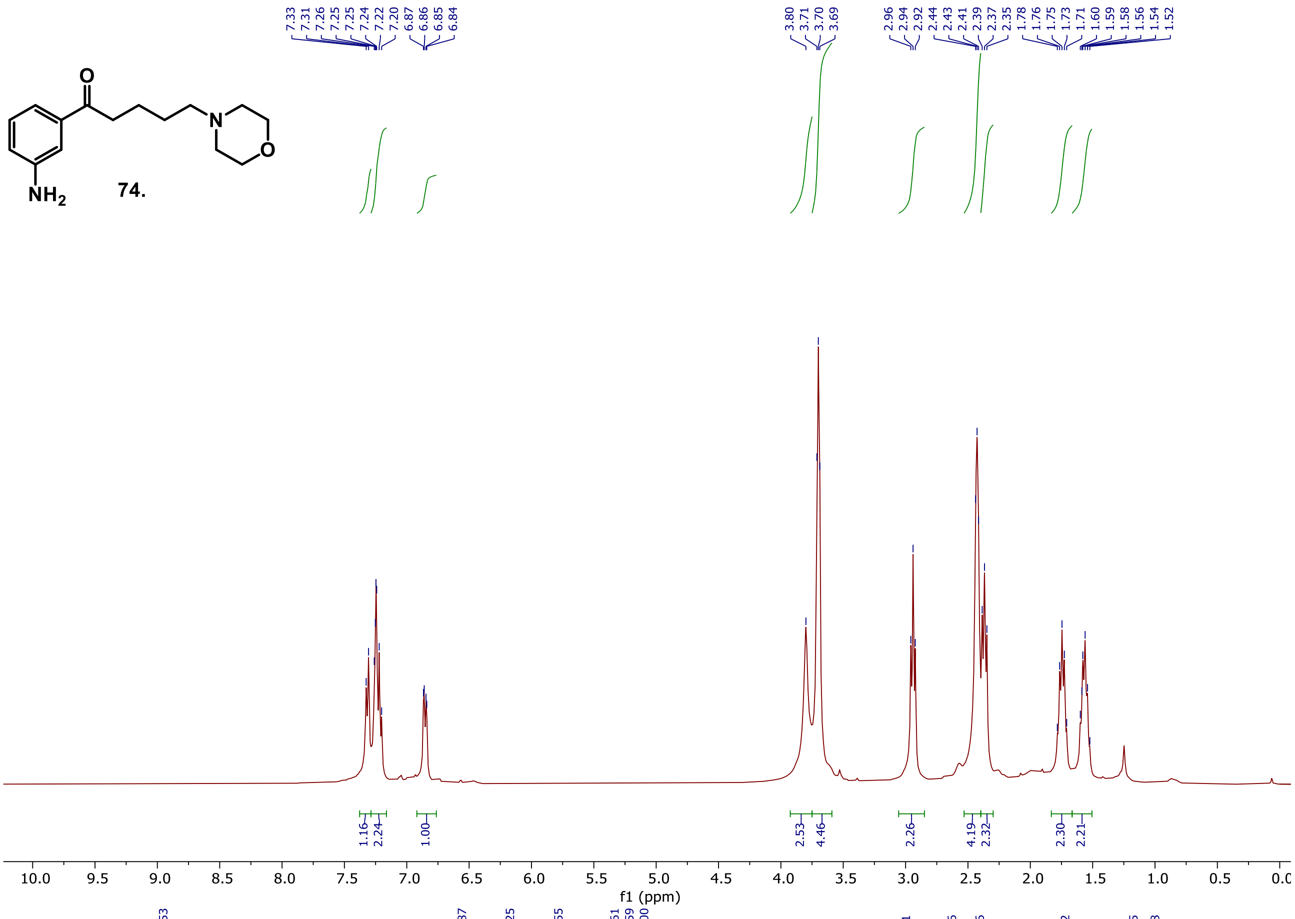

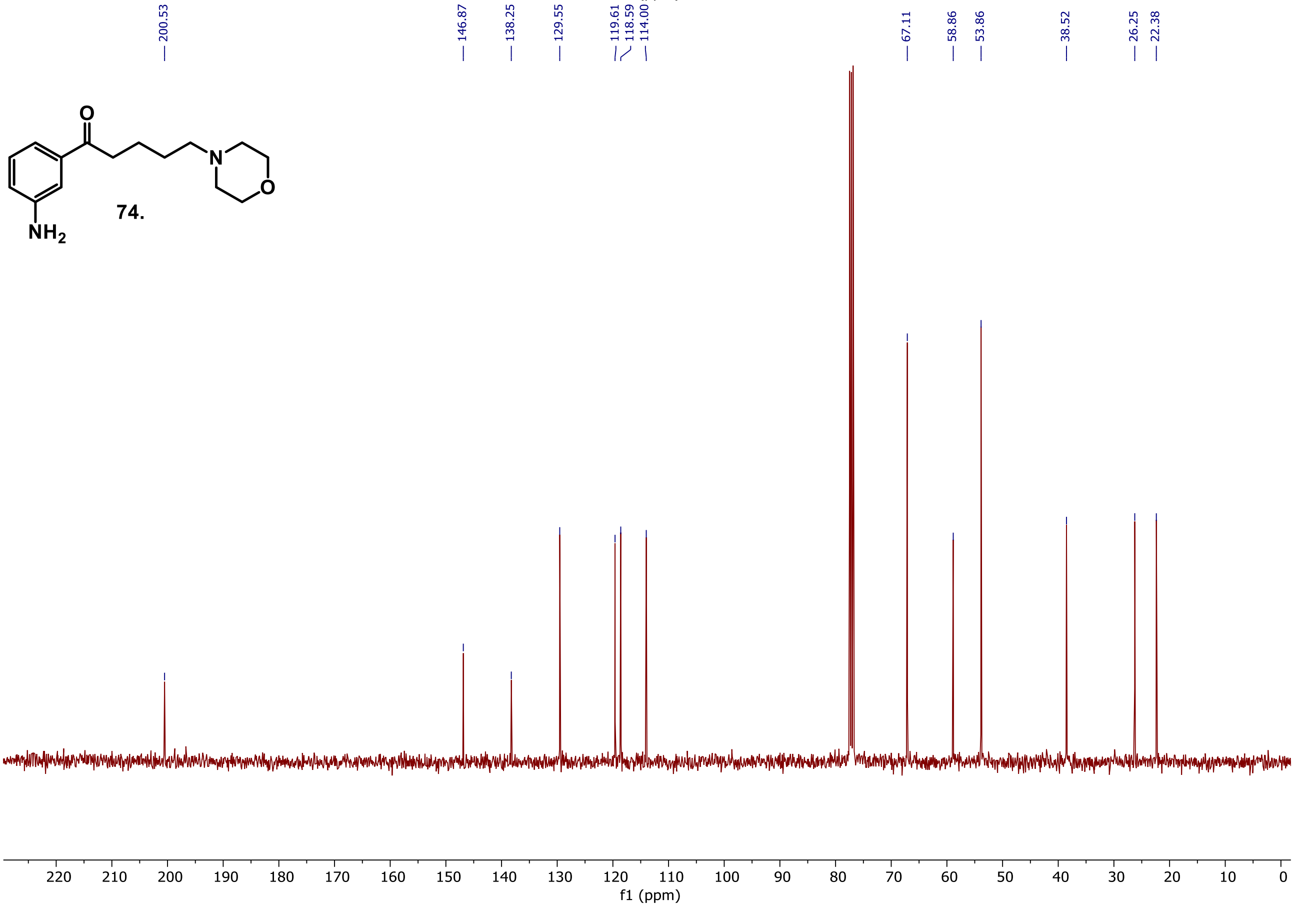

75: ^{1}H NMR (400 MHz, $CDCl_3$) and **^{13}C NMR** (101 MHz, $CDCl_3$) respectively.

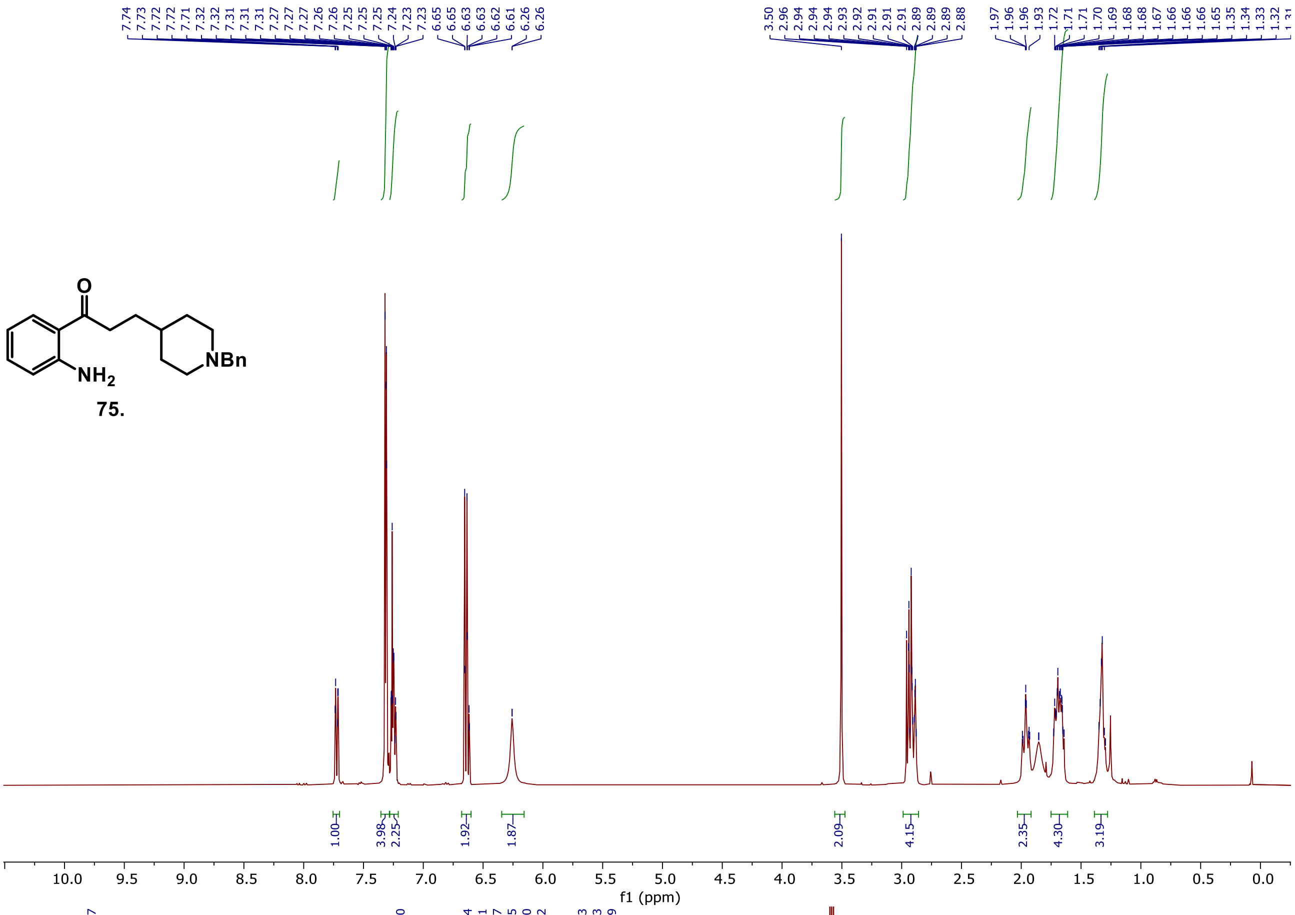

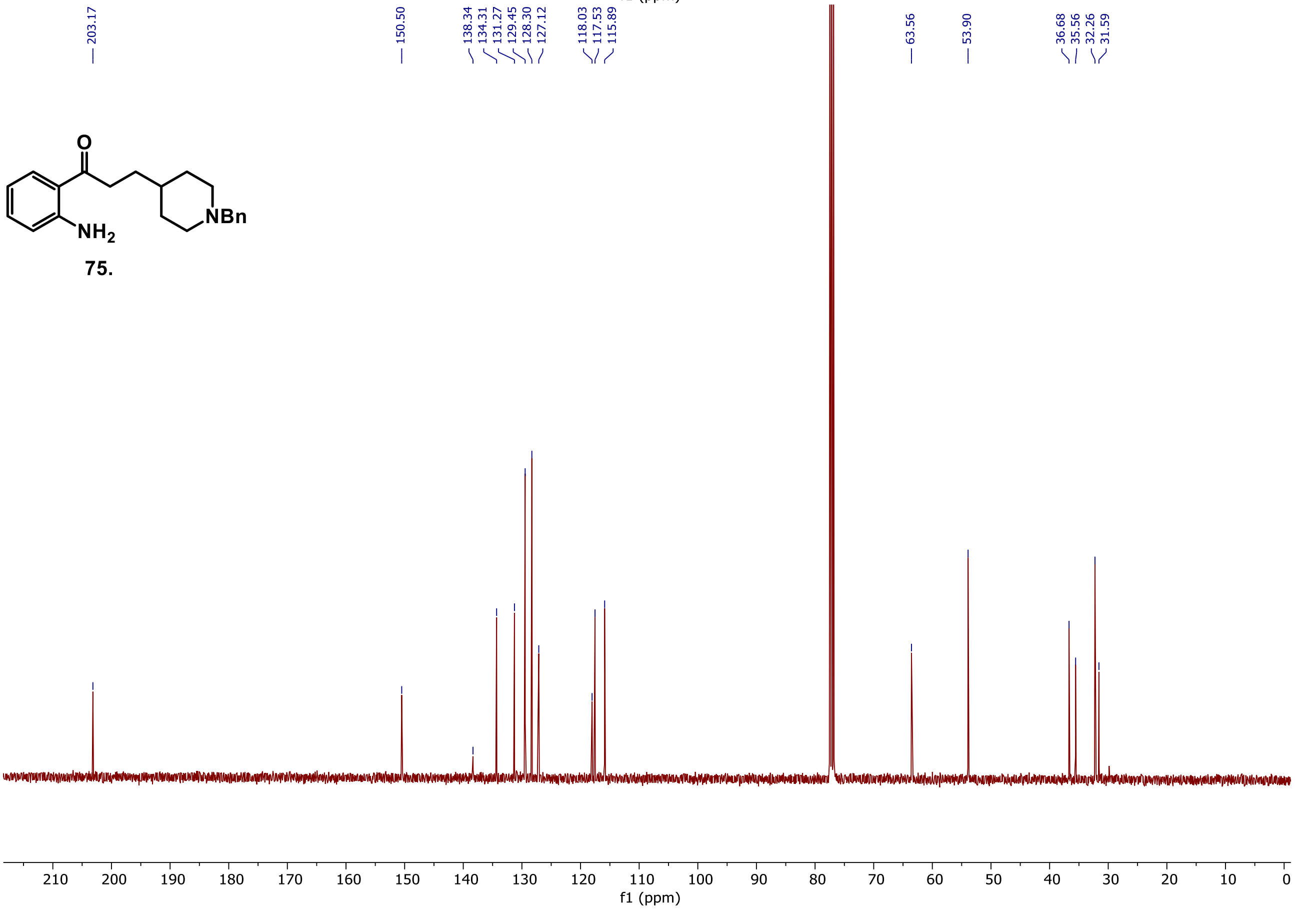

76: ^{1}H NMR (400 MHz, $CDCl_3$) and **^{13}C NMR** (101 MHz, $CDCl_3$) respectively.

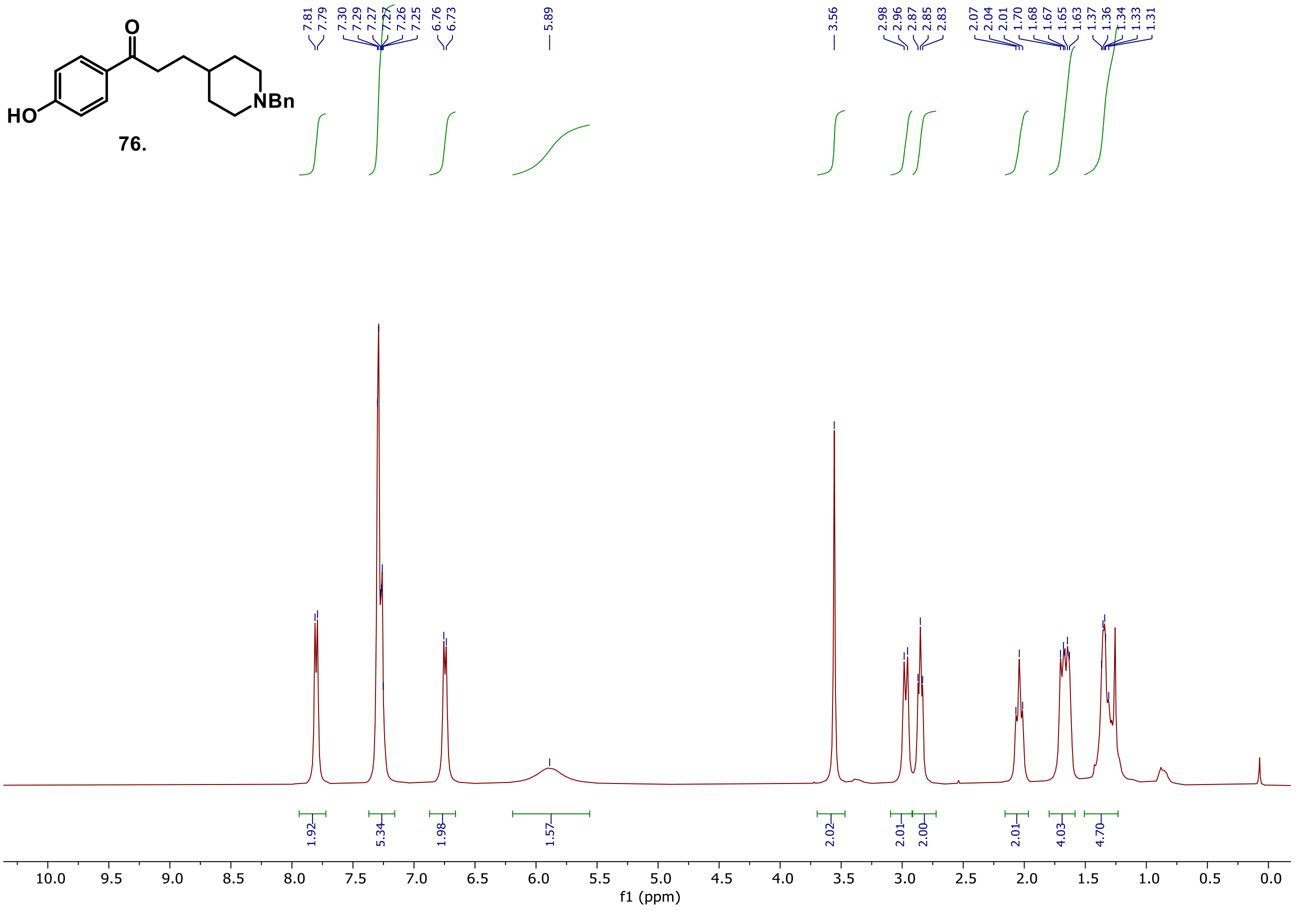

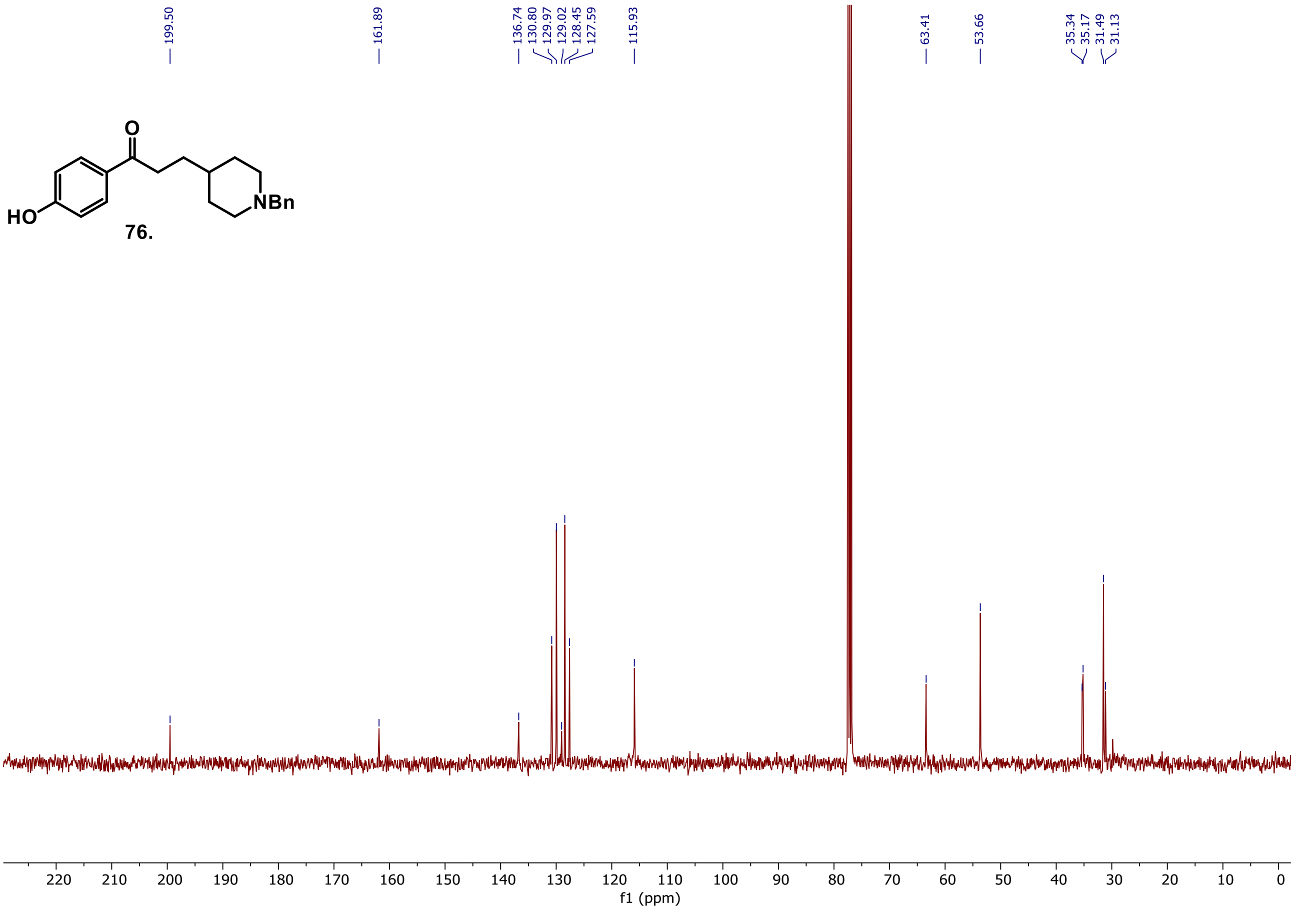

77: ^{1}H NMR (400 MHz, $CDCl_3$) and **^{13}C NMR** (101 MHz, $CDCl_3$) respectively.

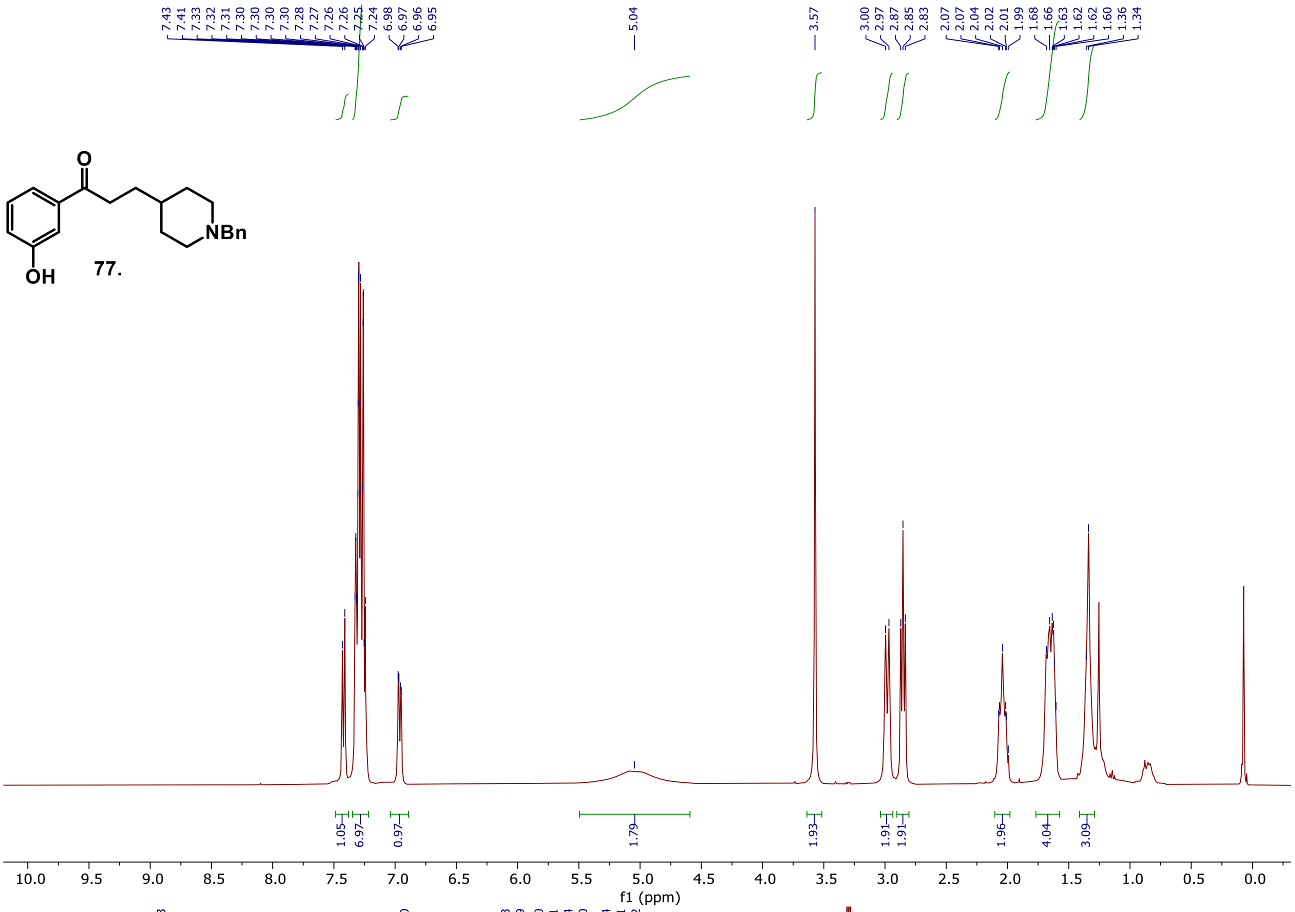

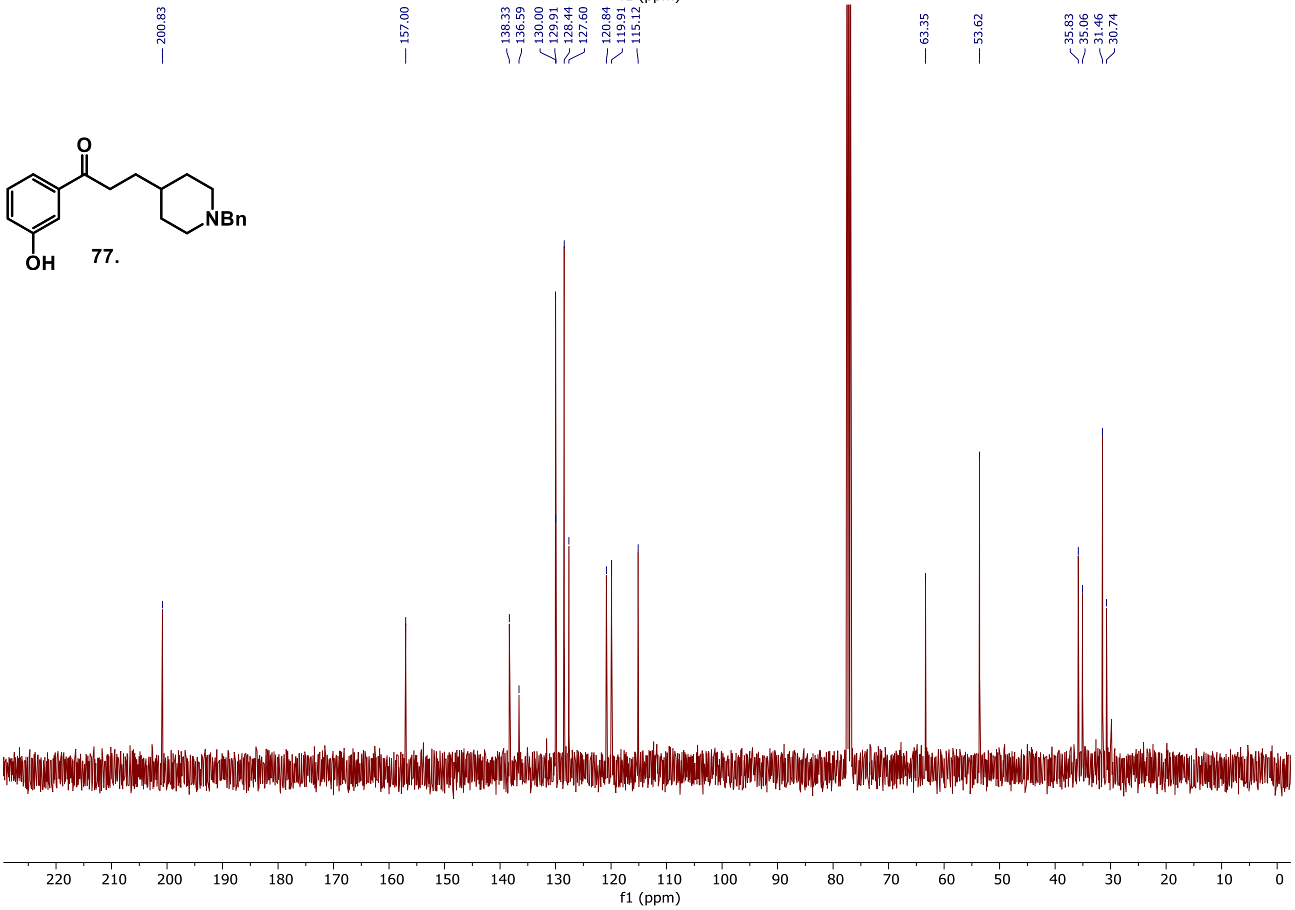

78: **^{1}H NMR** (400 MHz, $CDCl_3$) and **^{13}C NMR** (101 MHz, $CDCl_3$) respectively.

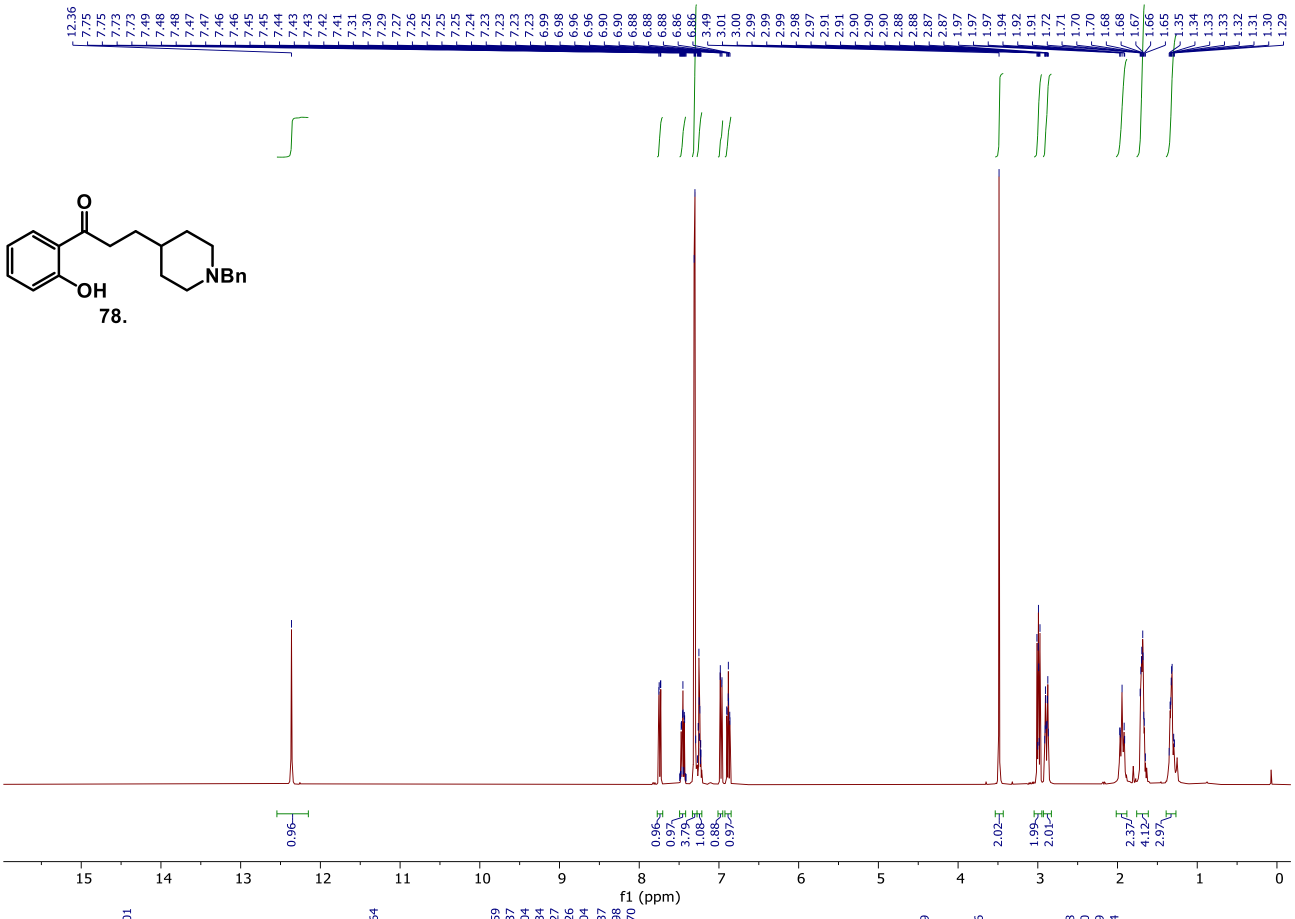

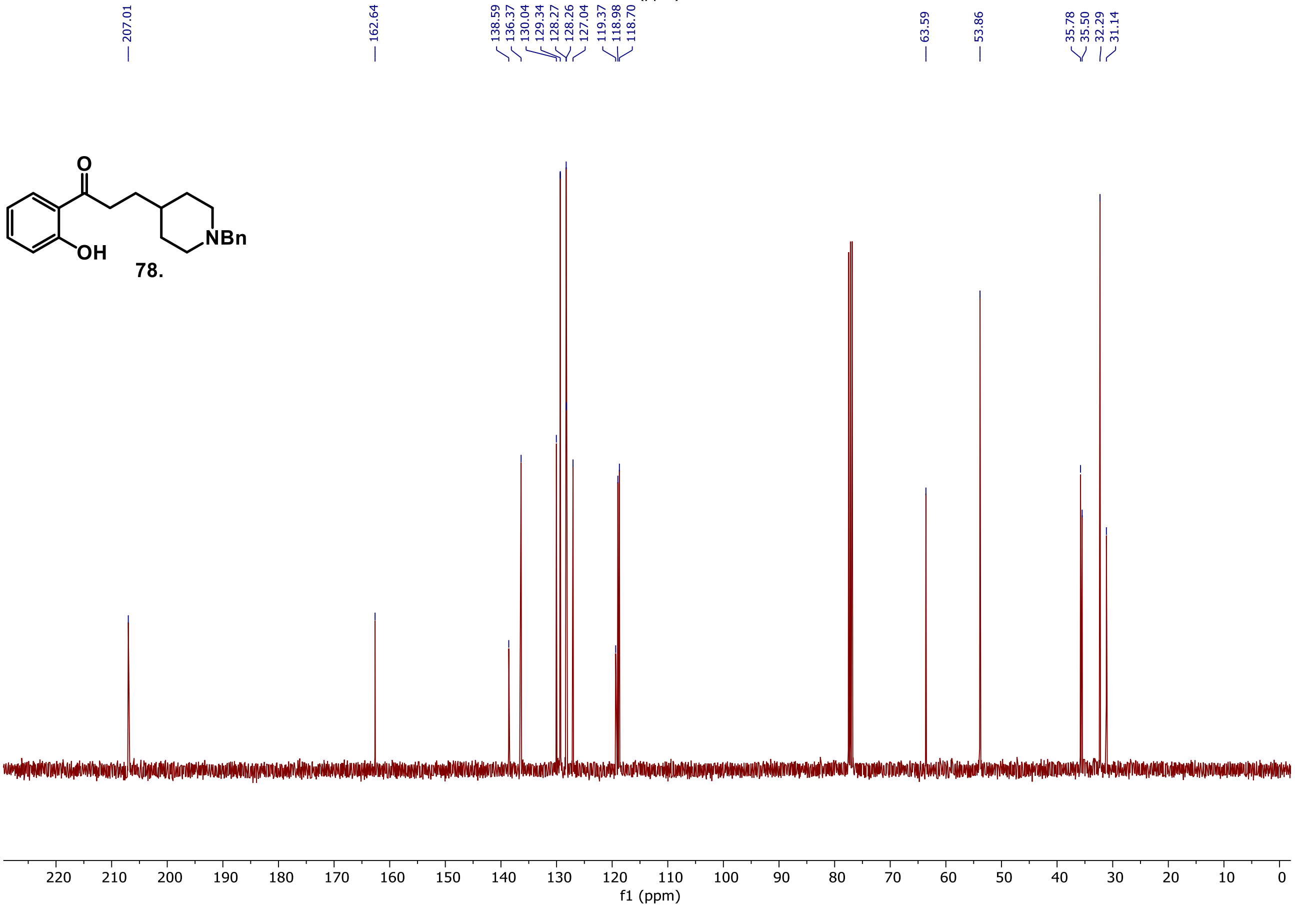

79: ^{1}H NMR (400 MHz, $CDCl_3$) and **^{13}C NMR** (101 MHz, $CDCl_3$) respectively.

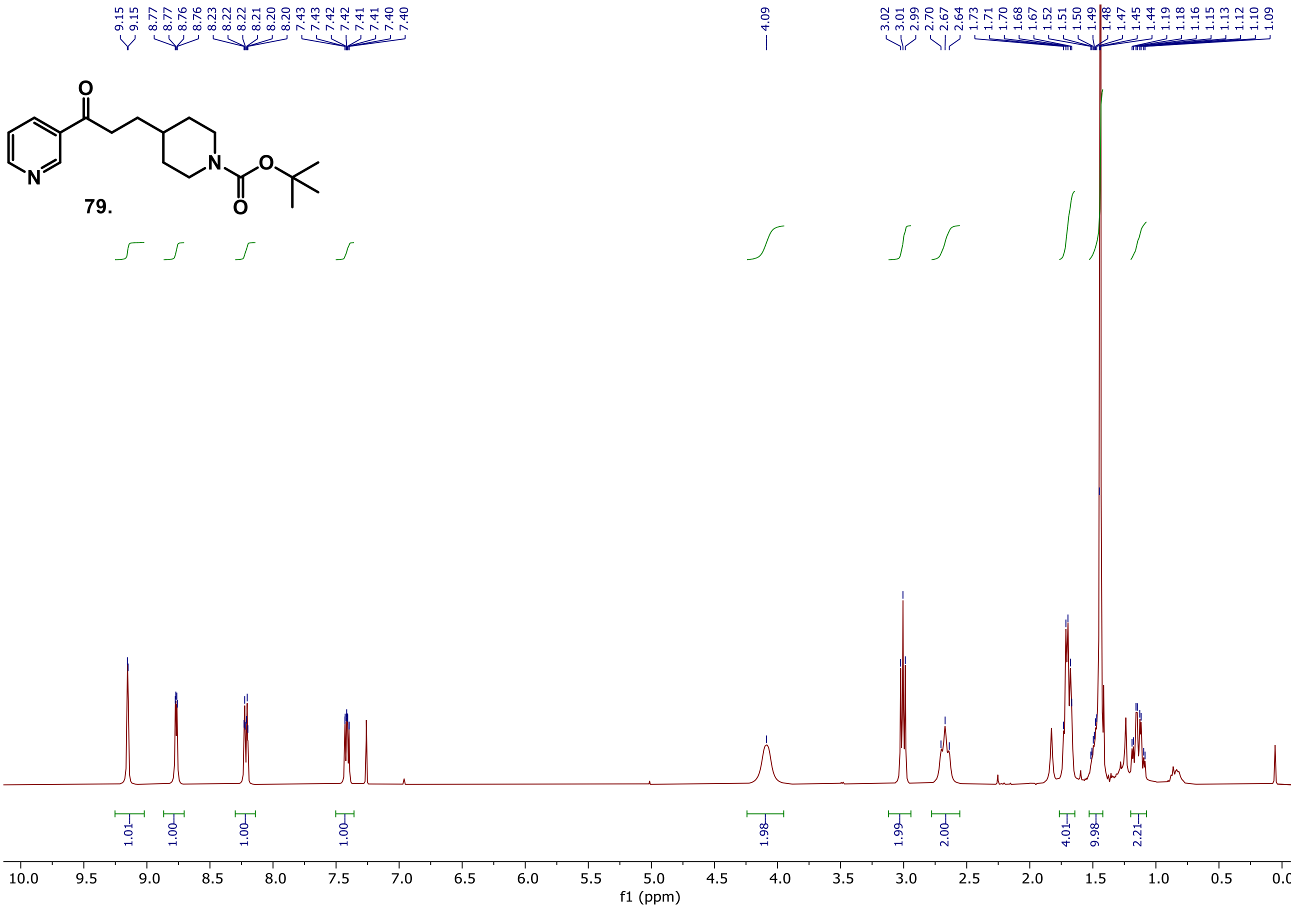

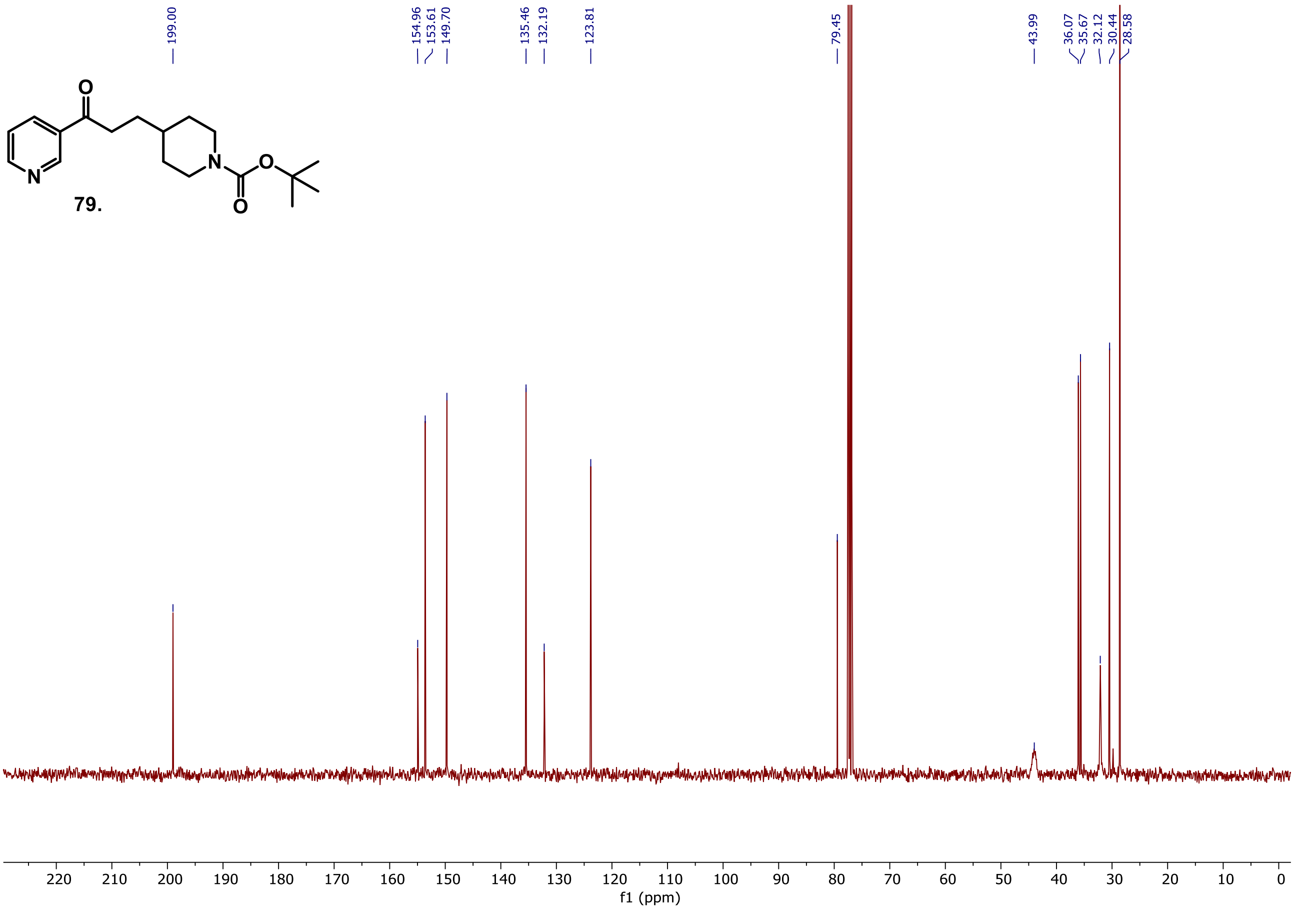

80: ^{1}**H NMR** (400 MHz, $CDCl_3$) and ^{13}**C NMR** (101 MHz, $CDCl_3$) respectively.

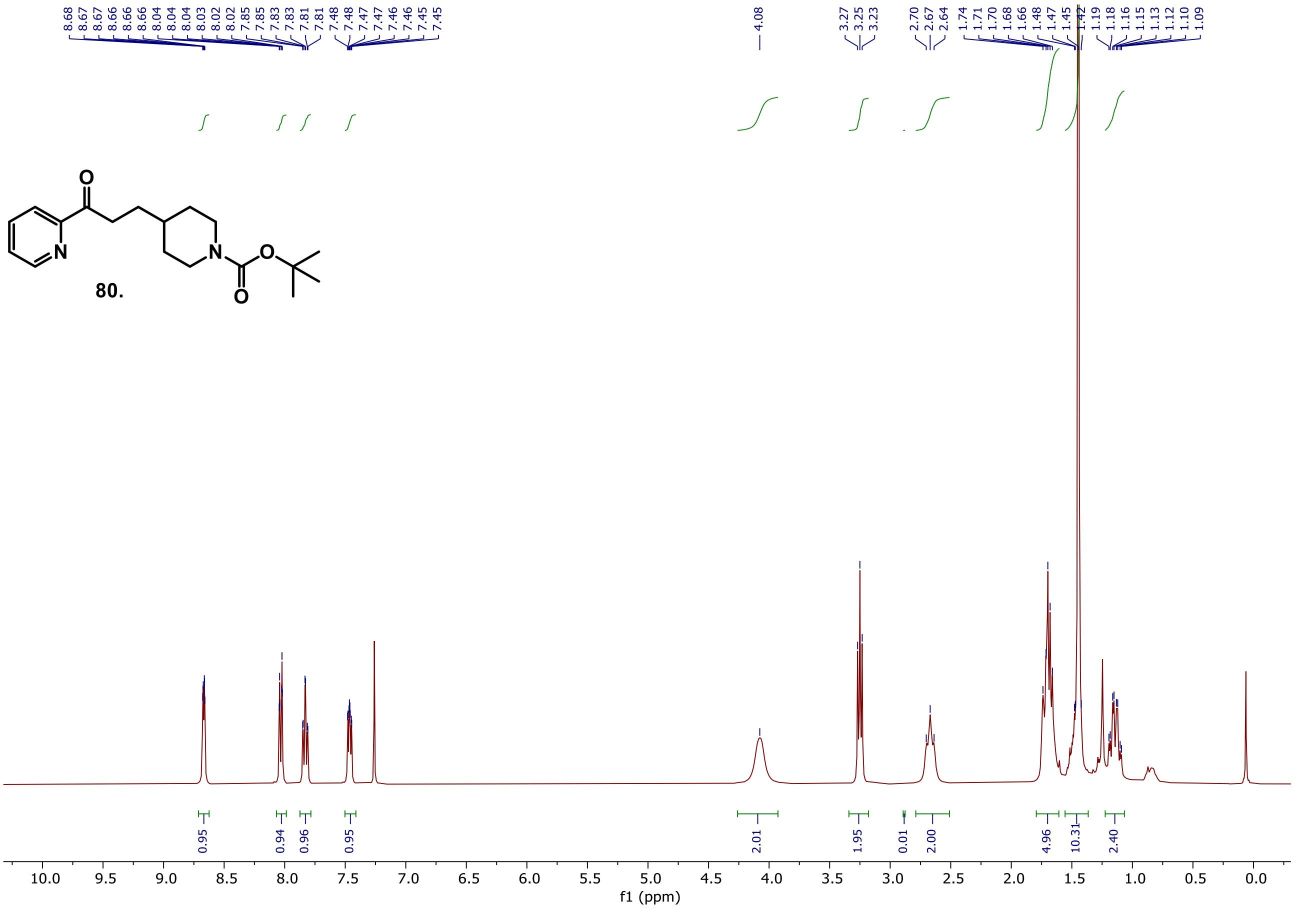

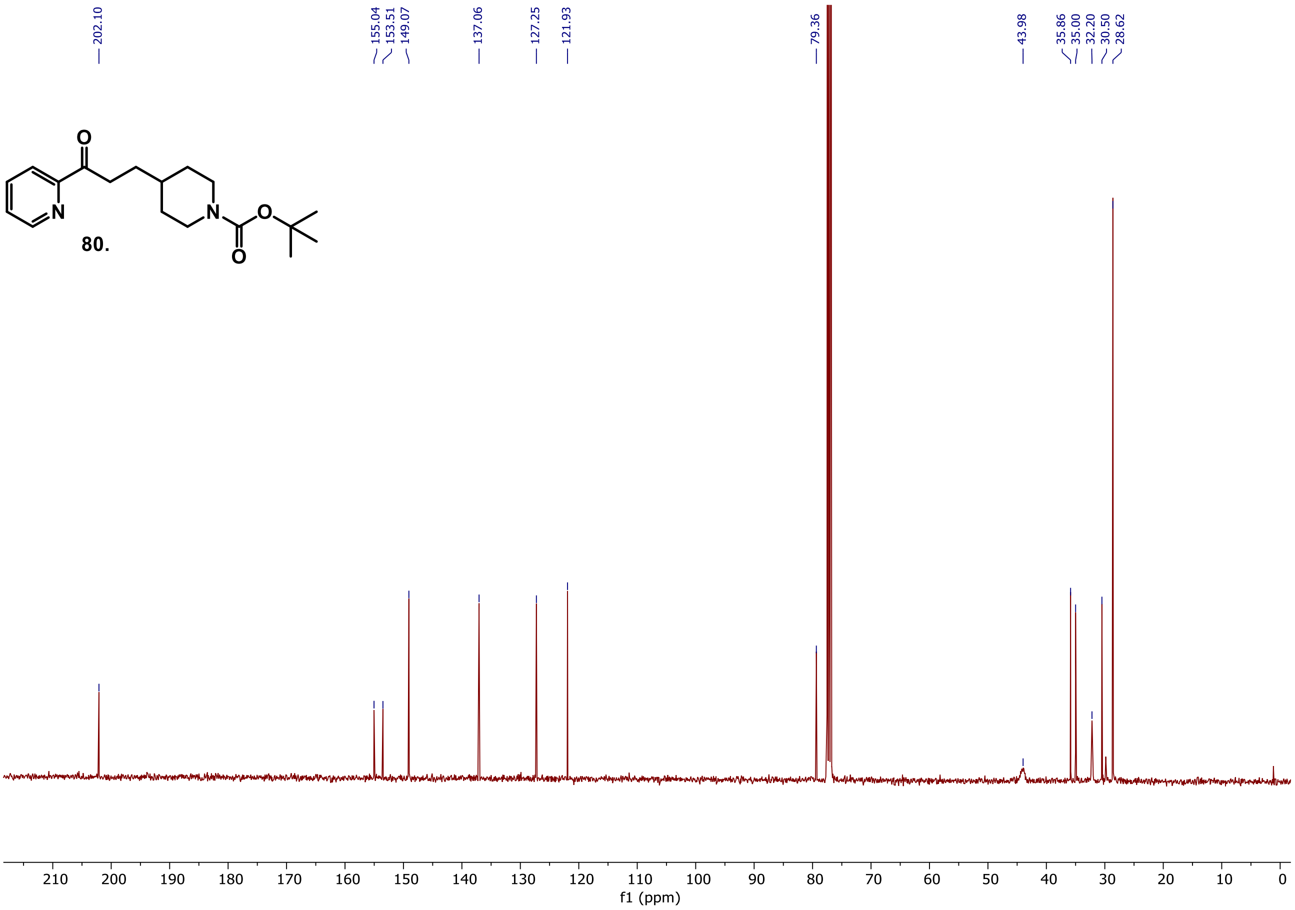

81: ^{1}H NMR (400 MHz, $CDCl_3$) and **^{13}C NMR** (101 MHz, $CDCl_3$) respectively.

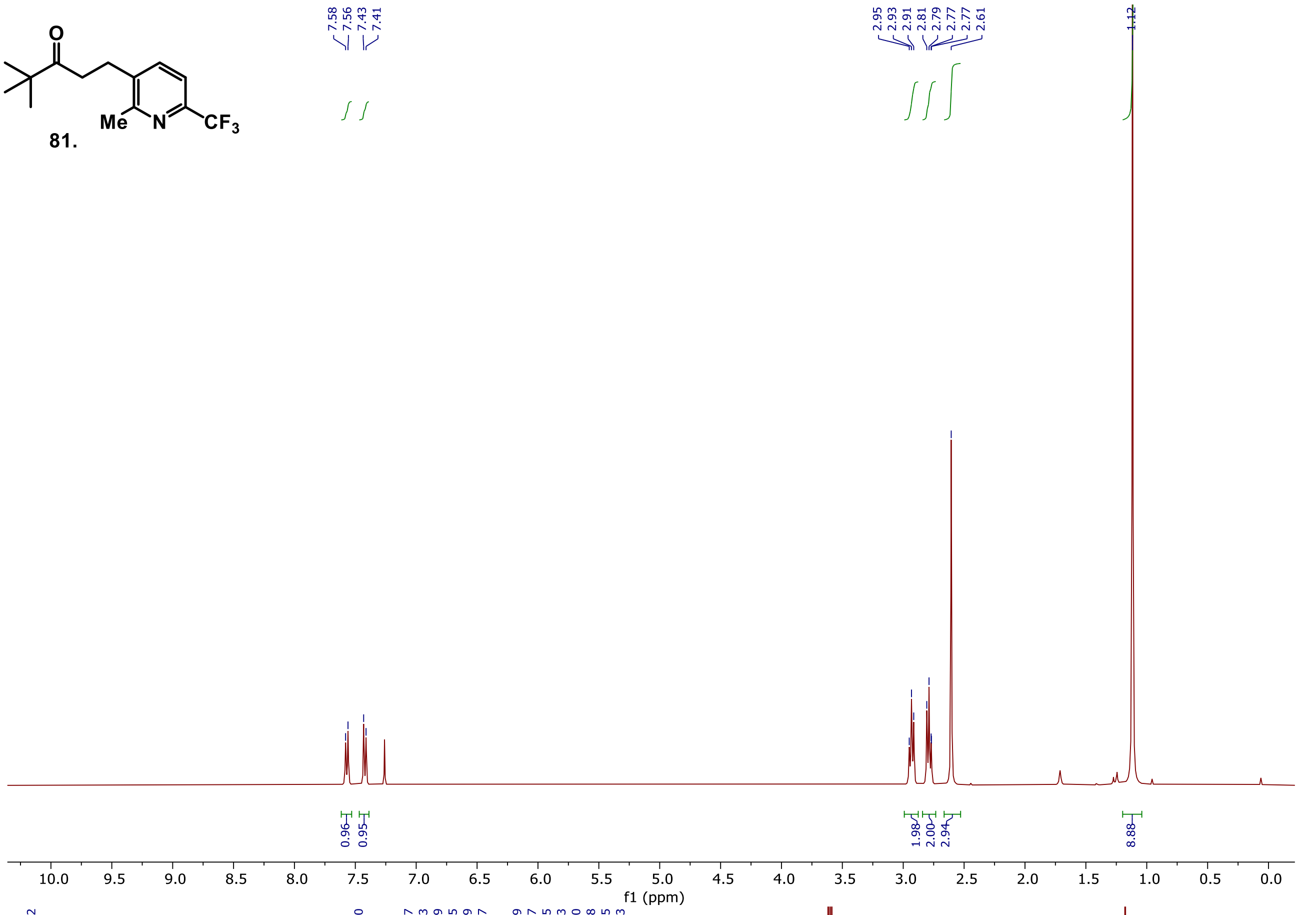

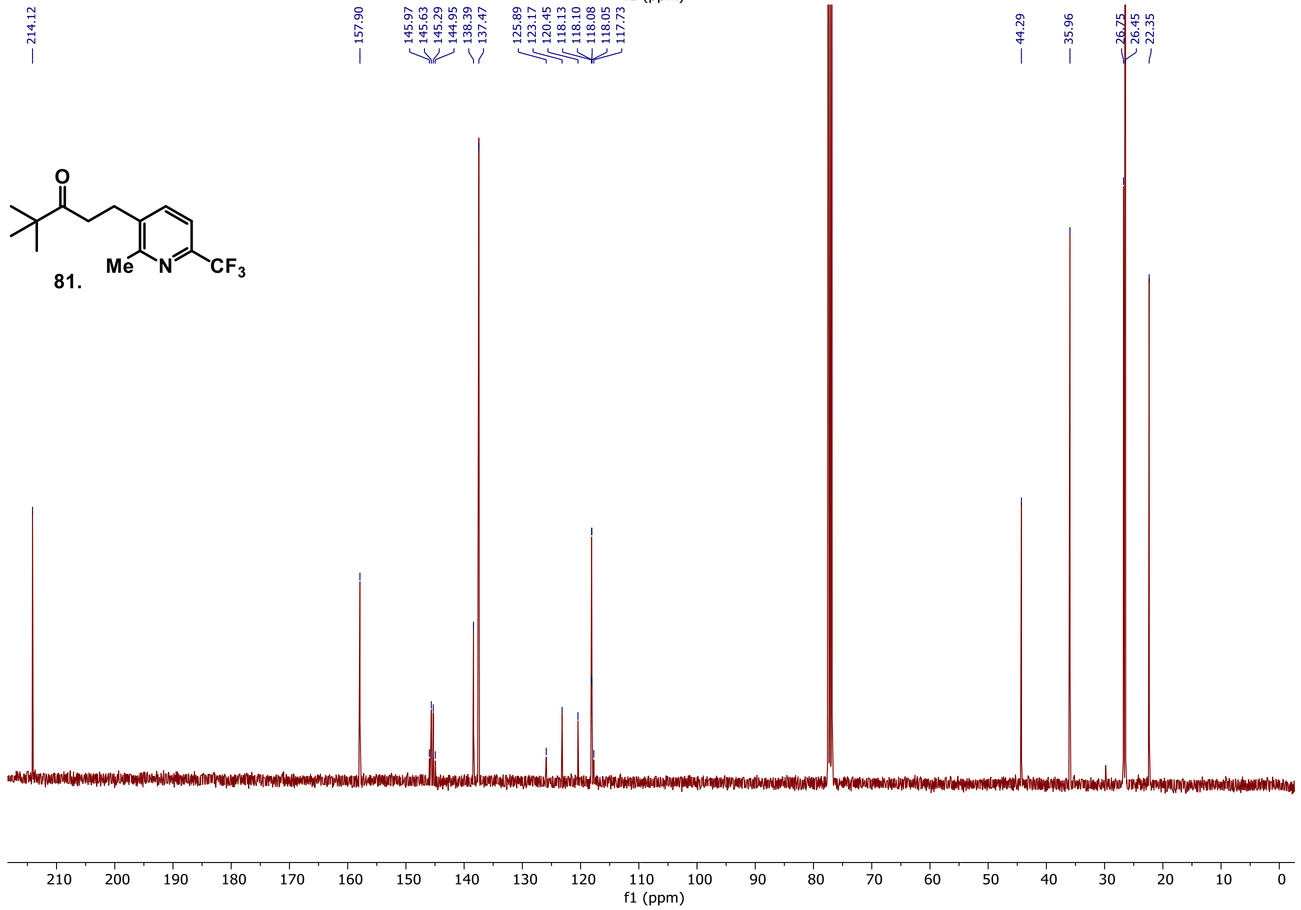

81: ¹⁹F NMR (376 MHz, $CDCl_3$) and **82: ¹H NMR** (500 MHz, $CDCl_3$) respectively.

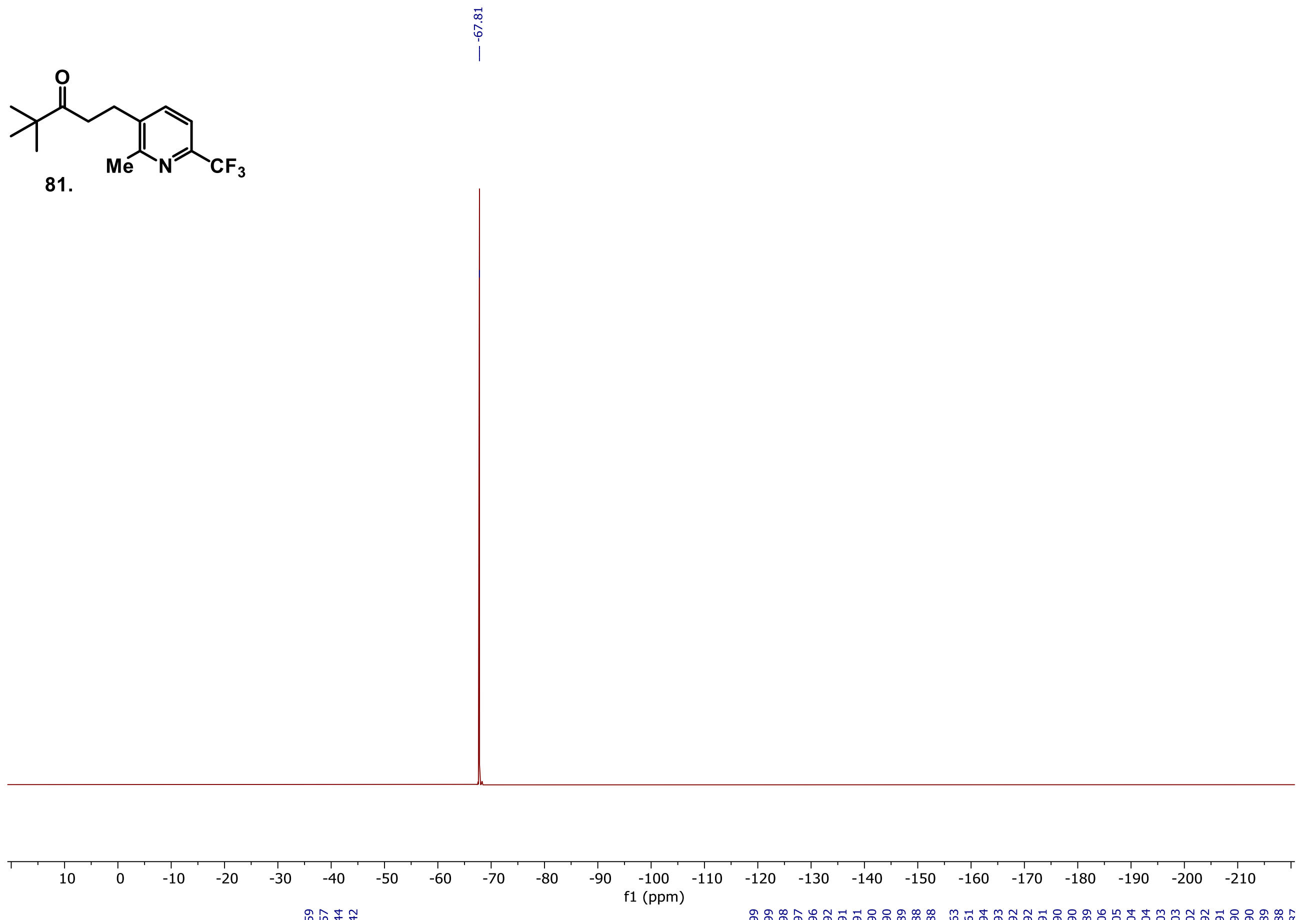

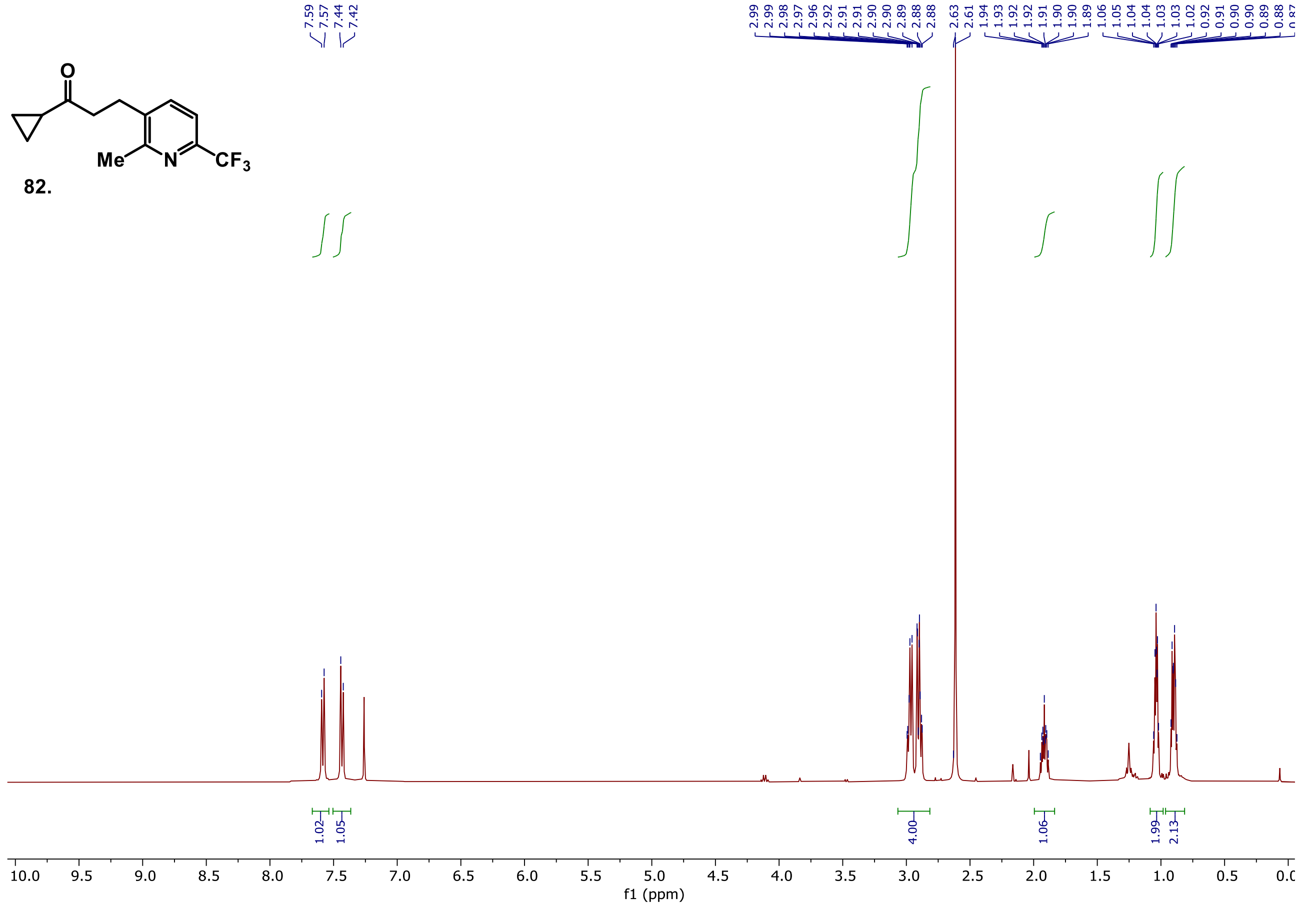

82: ^{13}**C NMR** (126 MHz, $CDCl_3$) and ^{19}**F NMR** (470 MHz, $CDCl_3$) respectively.

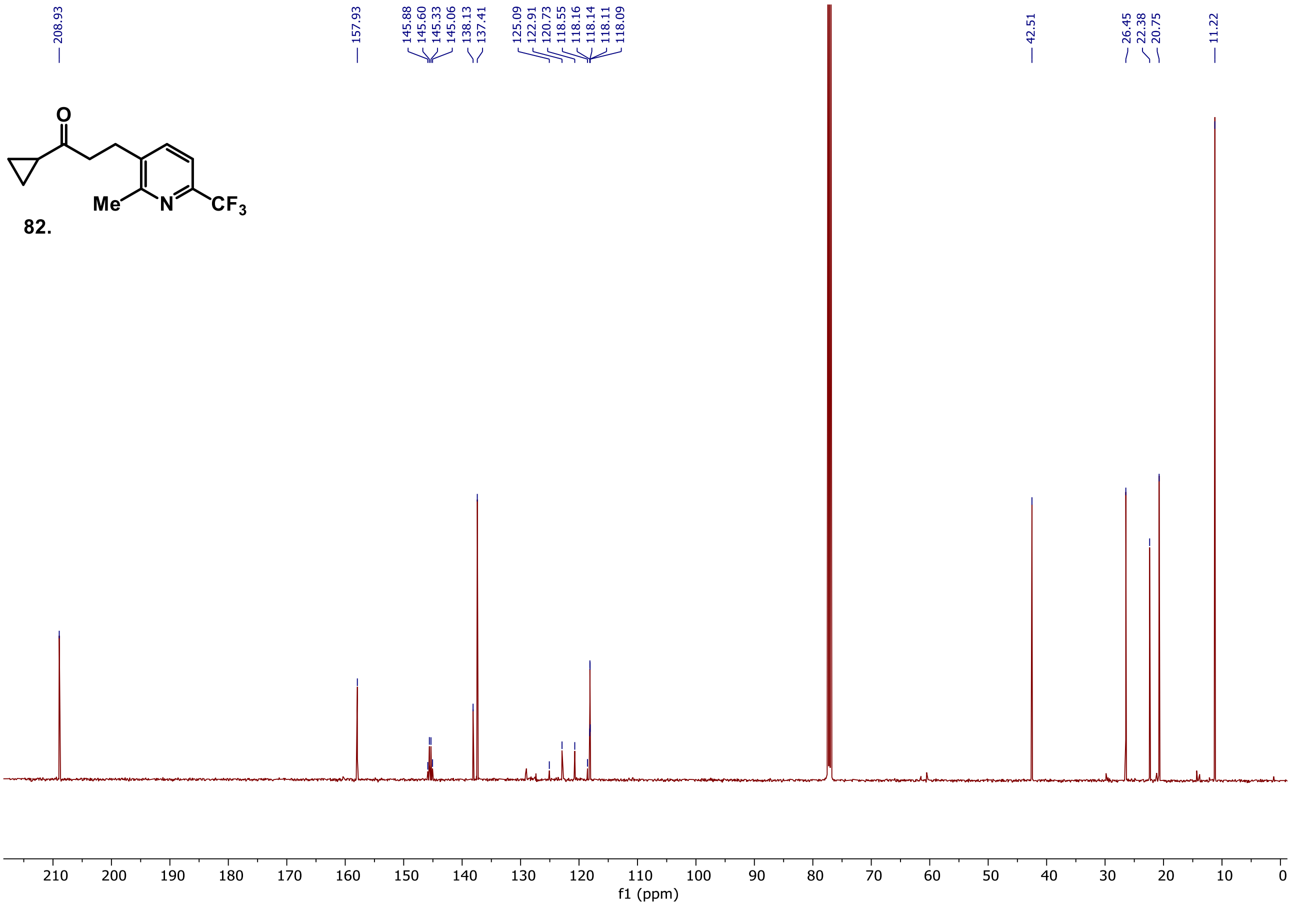

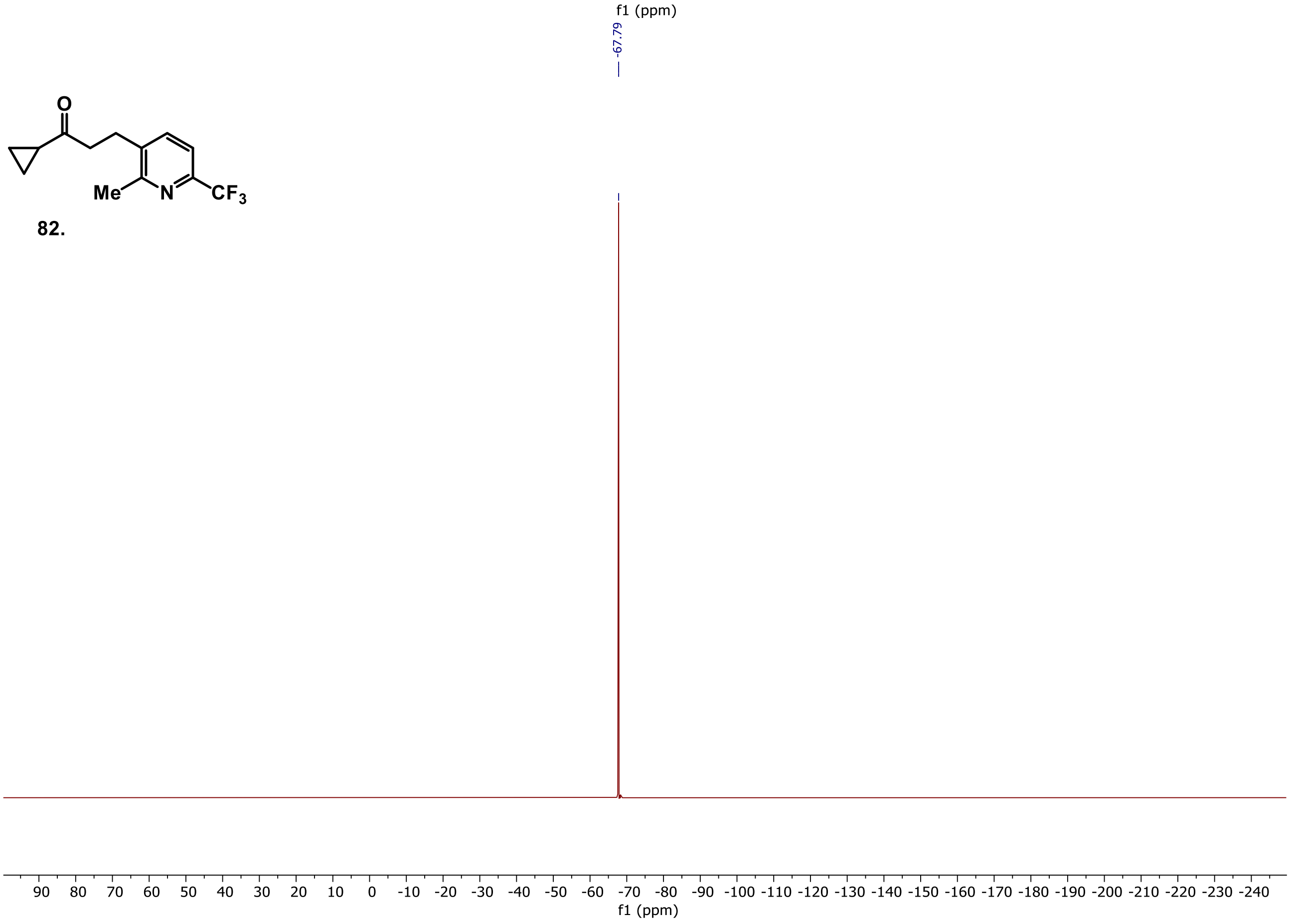

S82: [1]**H NMR** (400 MHz, $CDCl_3$) and [13]**C NMR** (126 MHz, $CDCl_3$) respectively.

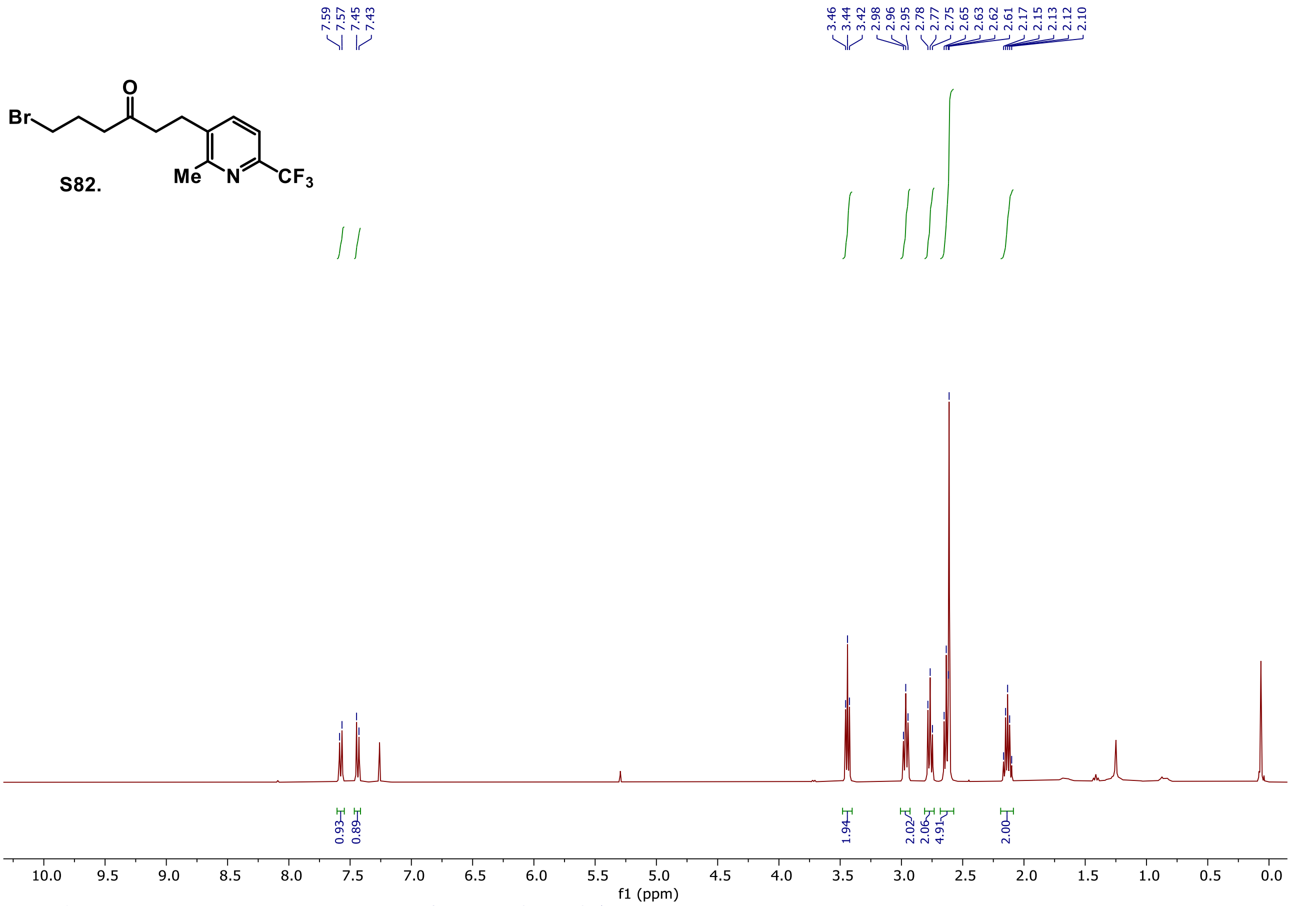

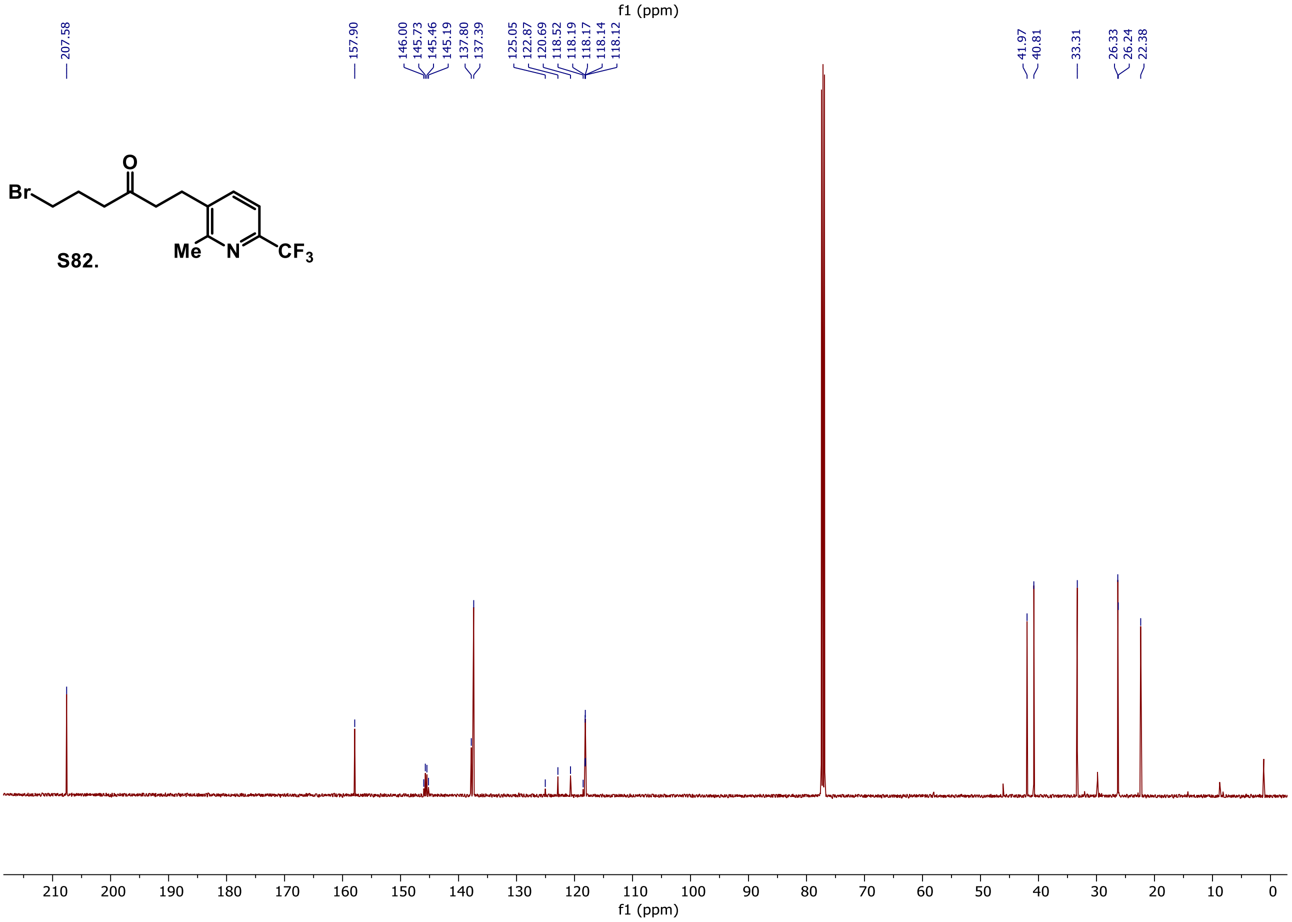

S82: ^{19}F NMR (376 MHz, $CDCl_3$).

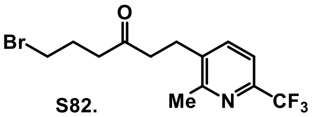

S82.

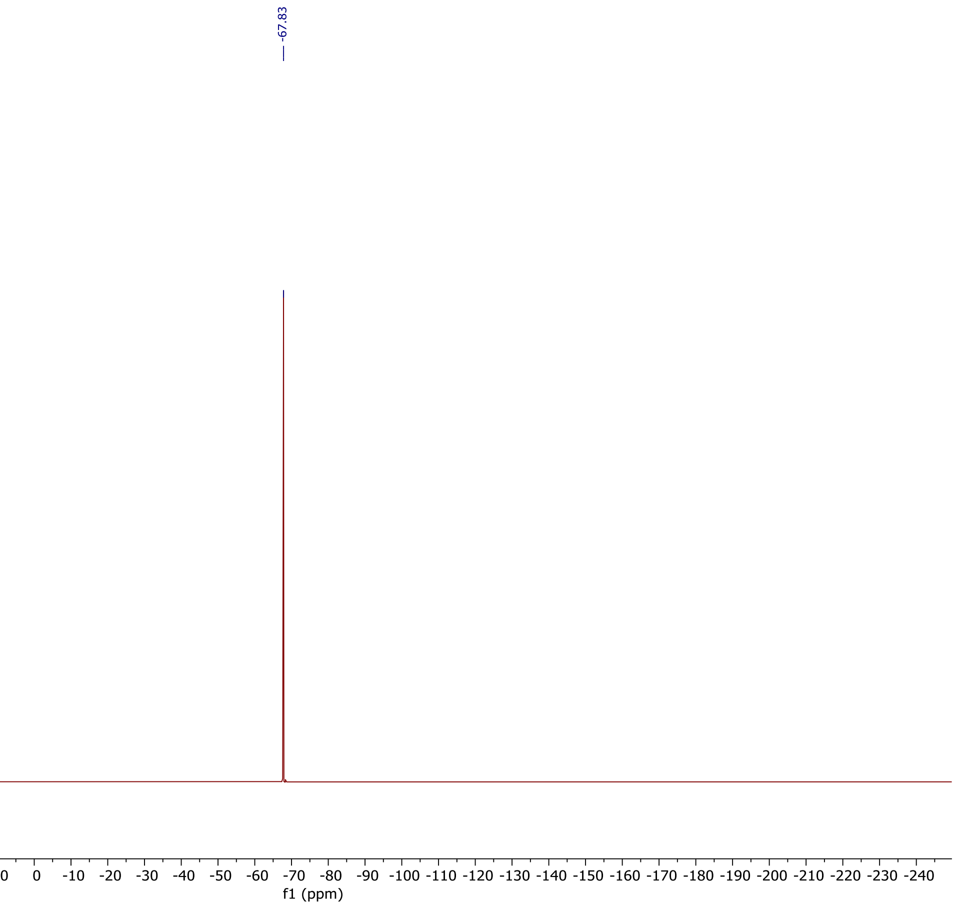

83: ^{1}H NMR (400 MHz, D_2O) and **^{13}C NMR** (101 MHz, D_2O) respectively.

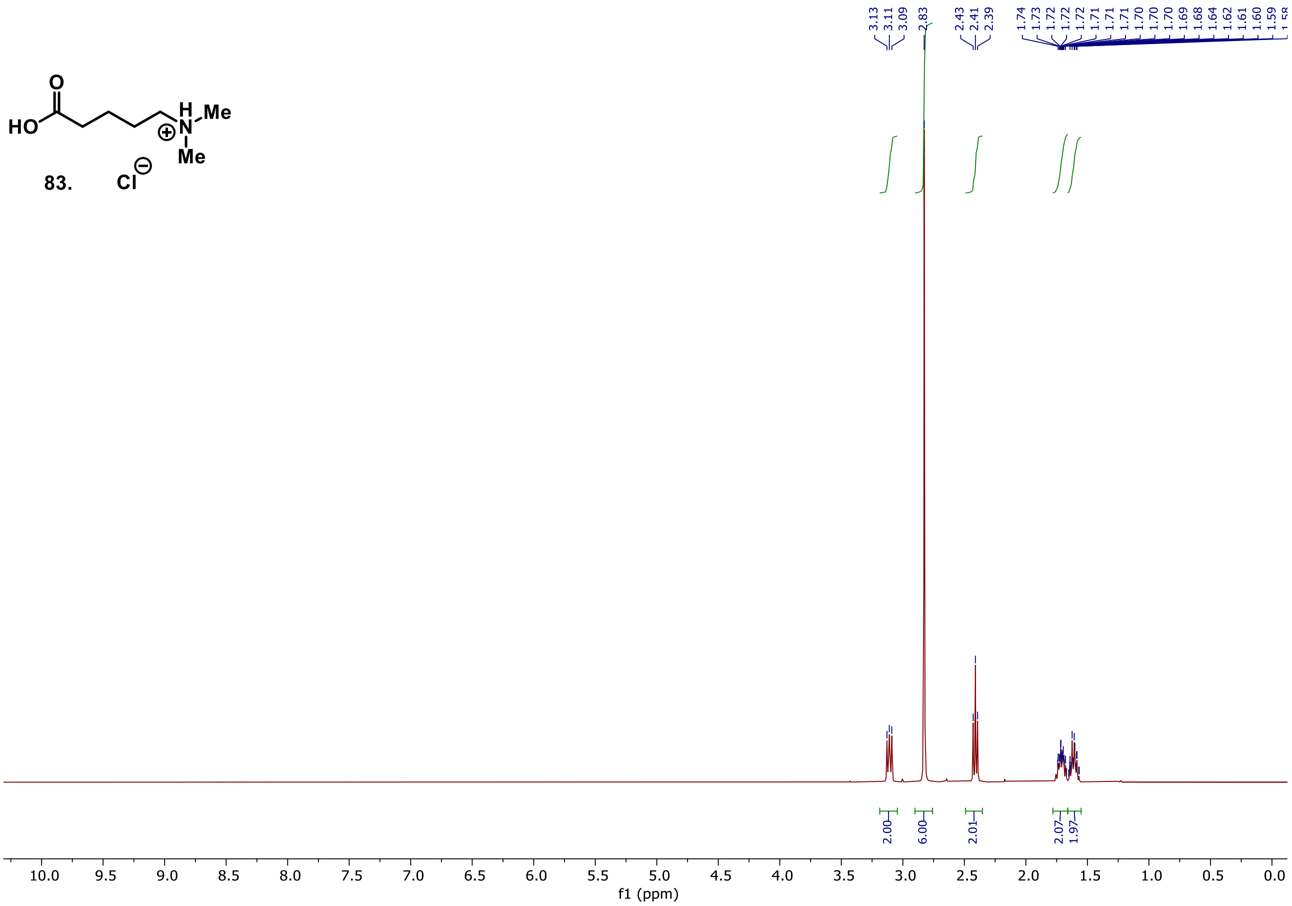

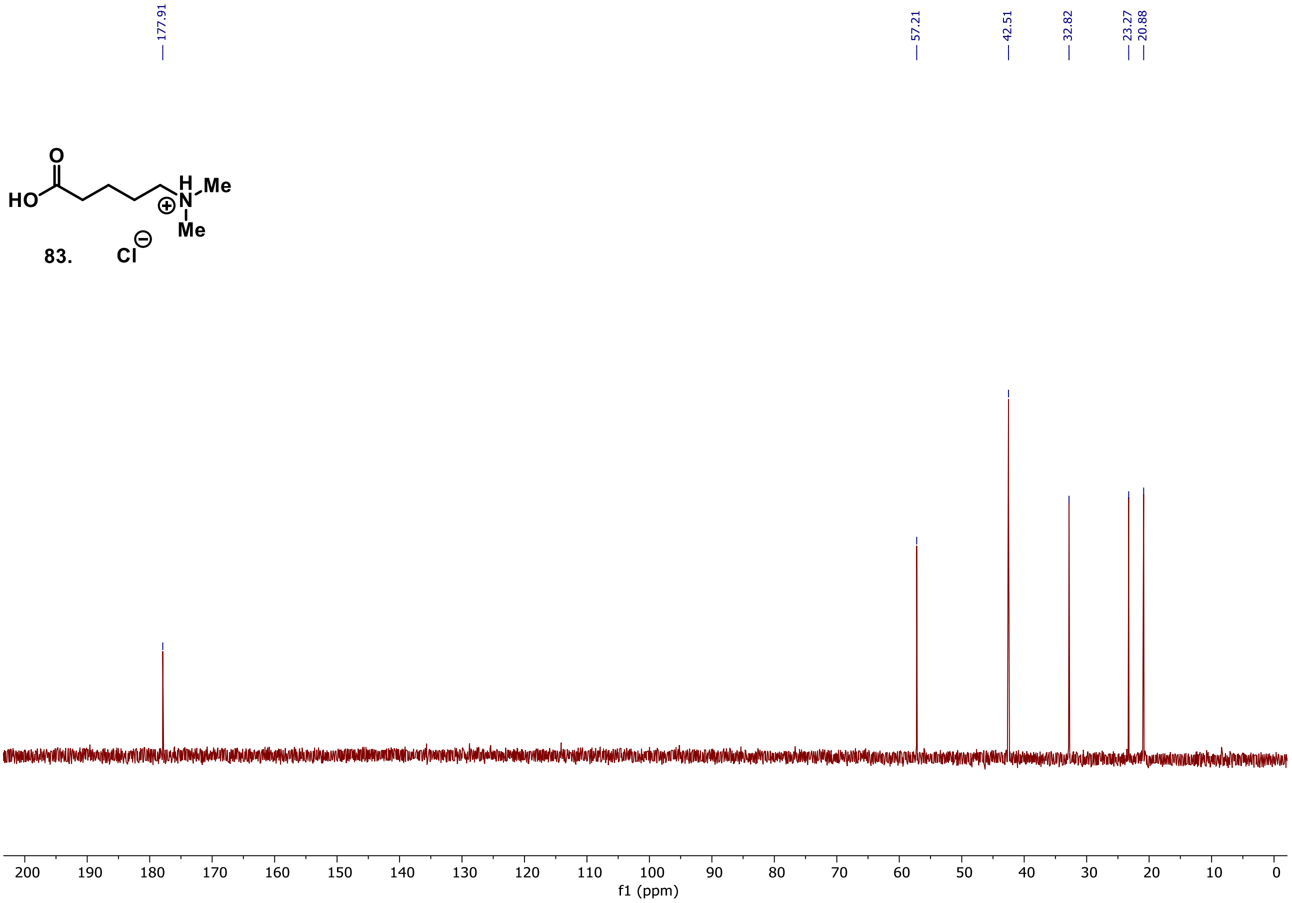

84: ^{1}H NMR (400 MHz, D_2O) and **^{13}C NMR** (101 MHz, D_2O) respectively.

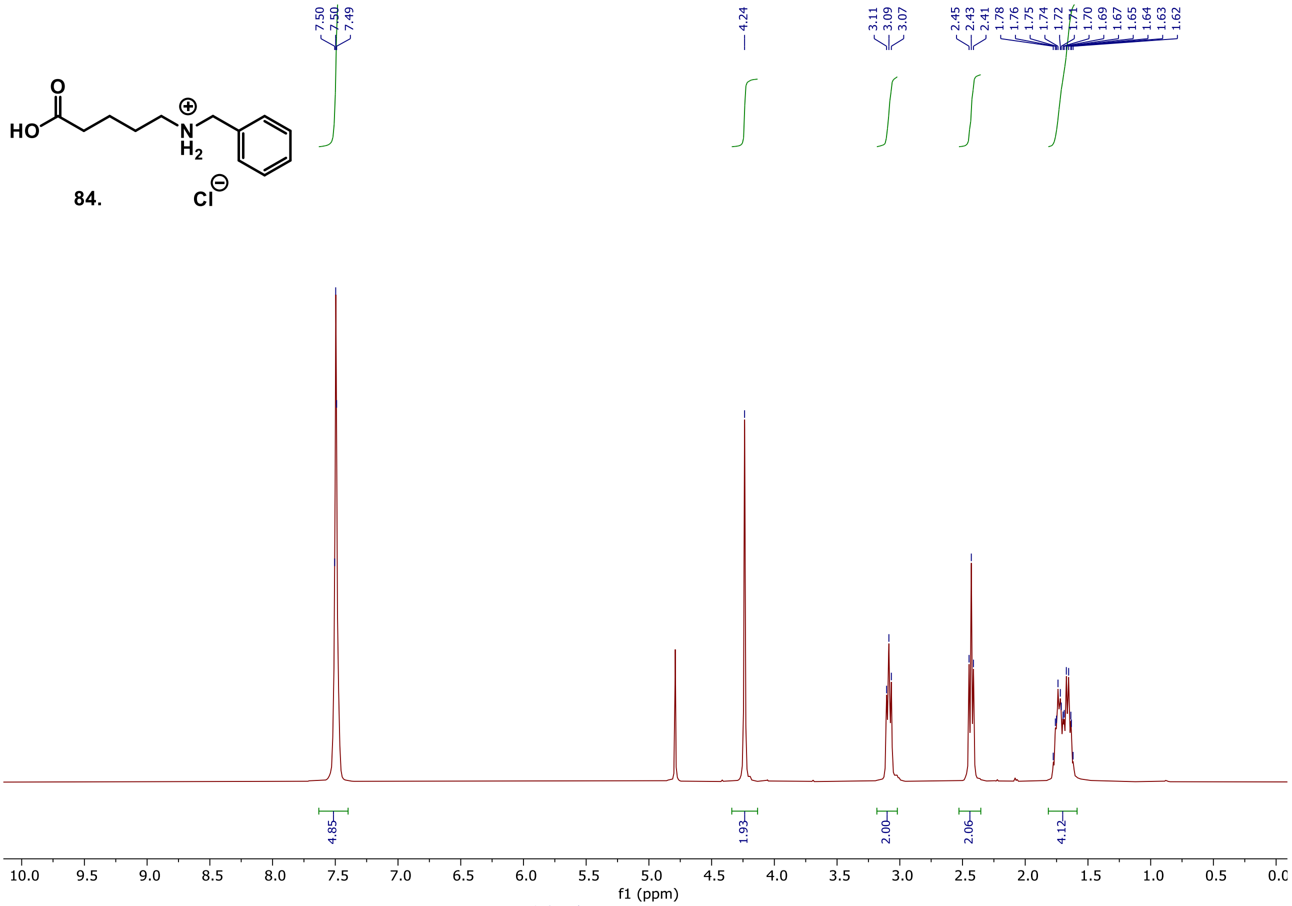

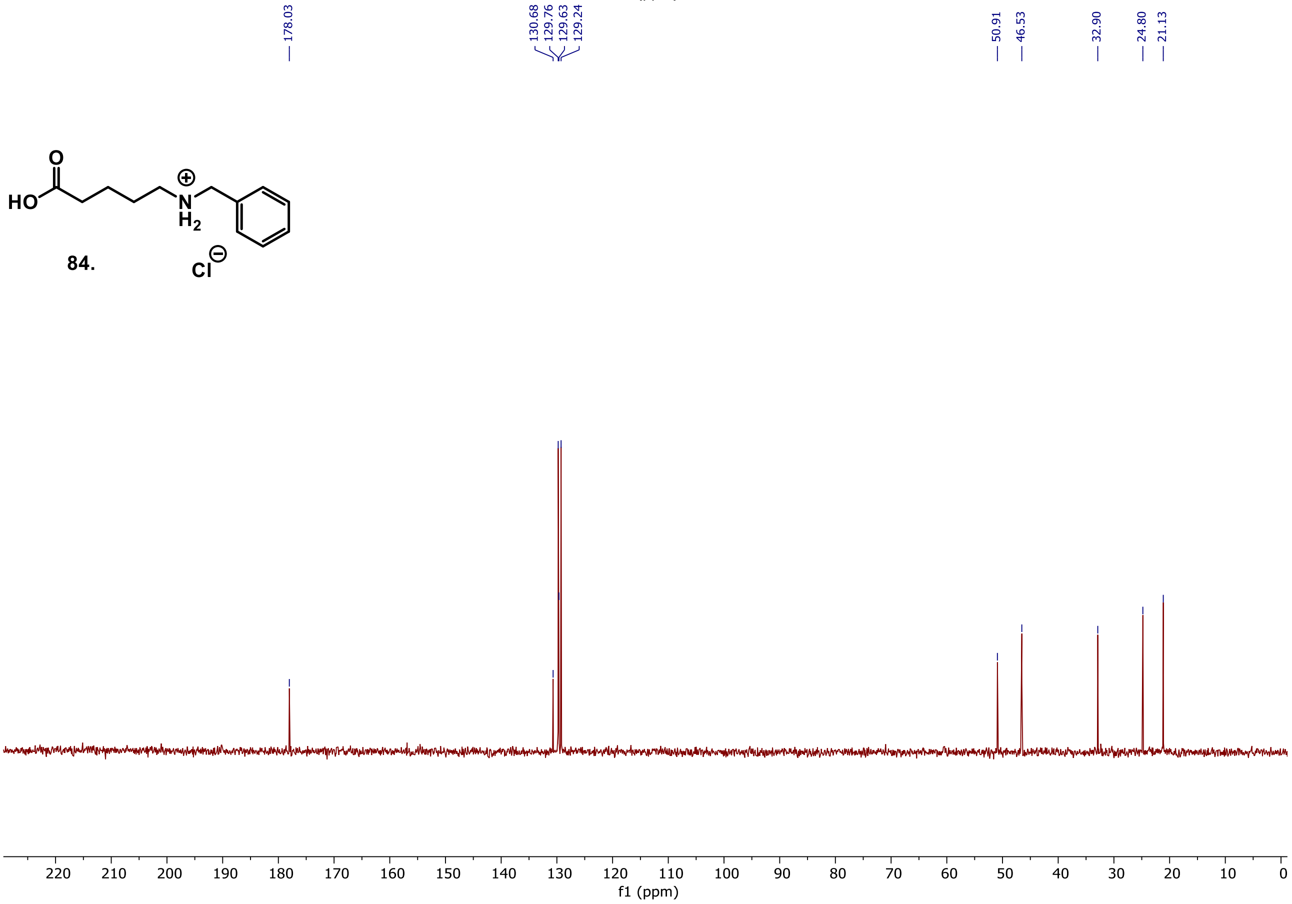

85: ¹H NMR (400 MHz, D_2O) and **¹³C NMR** (101 MHz, D_2O) respectively.

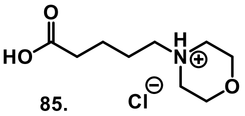

85.

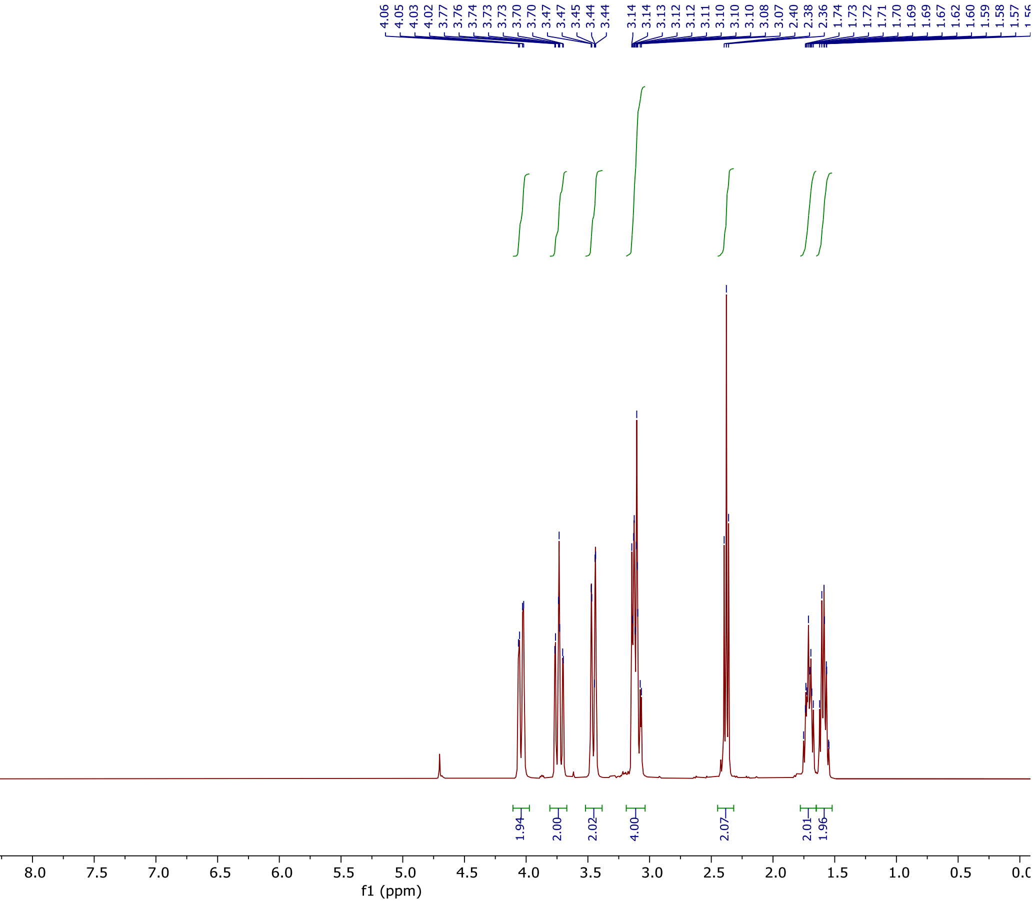

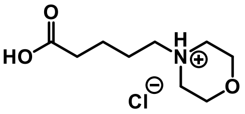

85.

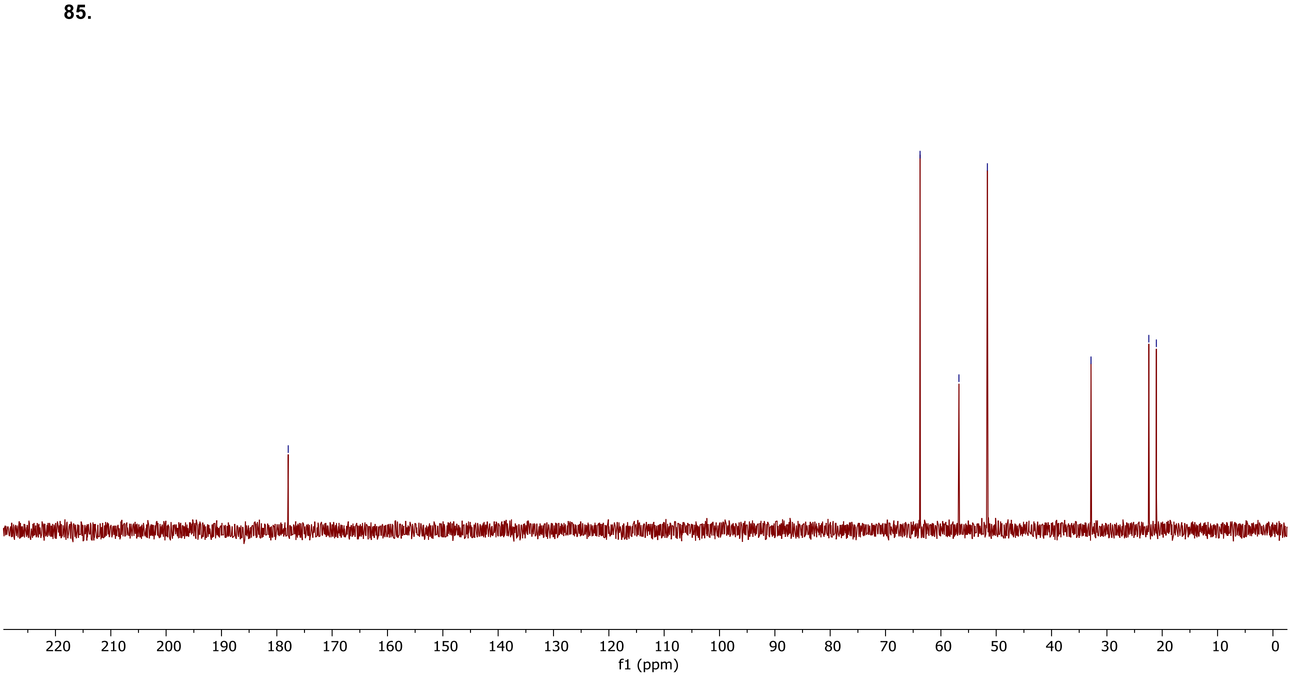

86: **^{1}H NMR** (400 MHz, D_2O) and **^{13}C NMR** (101 MHz, D_2O) respectively.

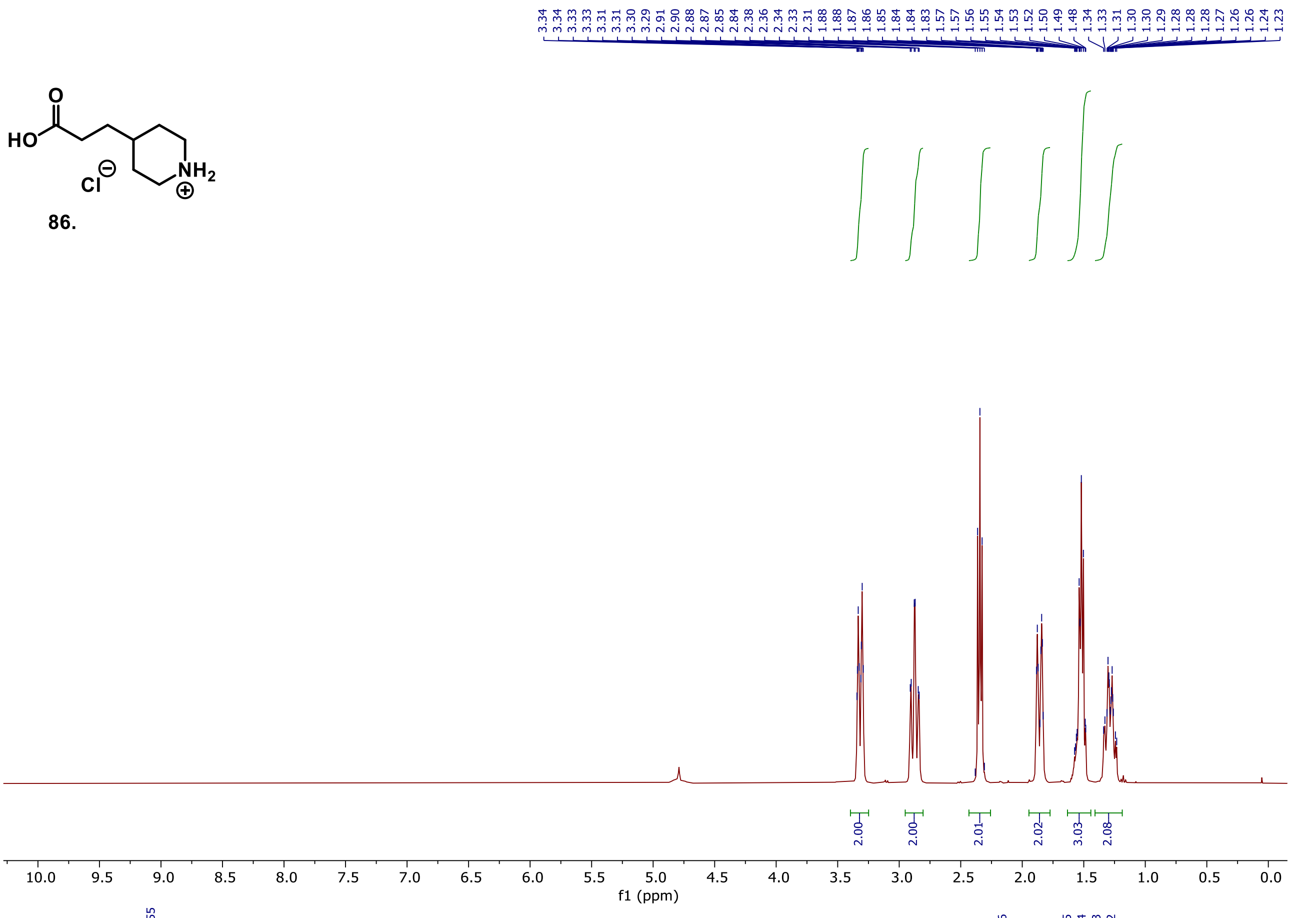

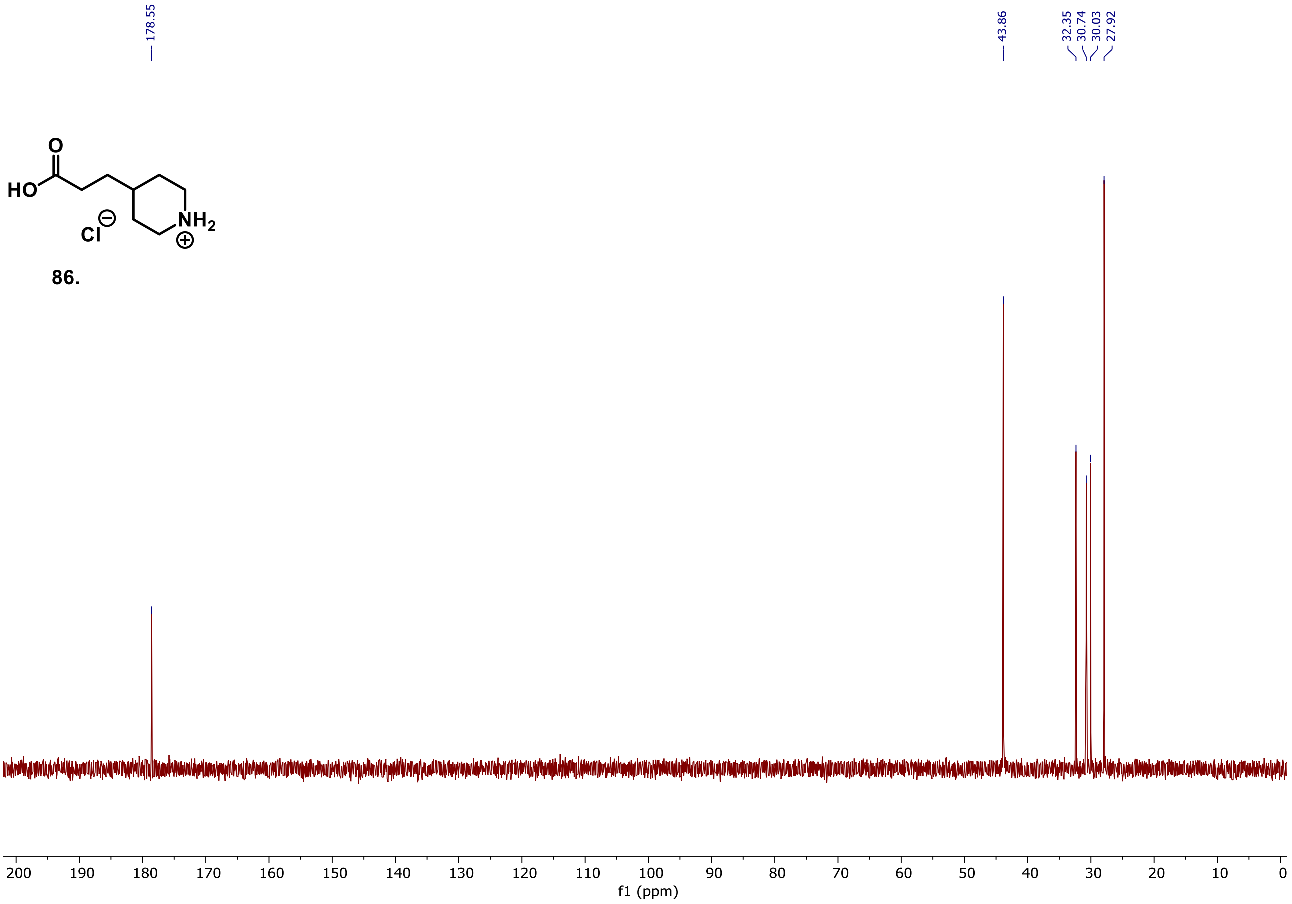

87: ^{1}H NMR (400 MHz, D_2O) and **^{13}C NMR** (126 MHz, D_2O) respectively.

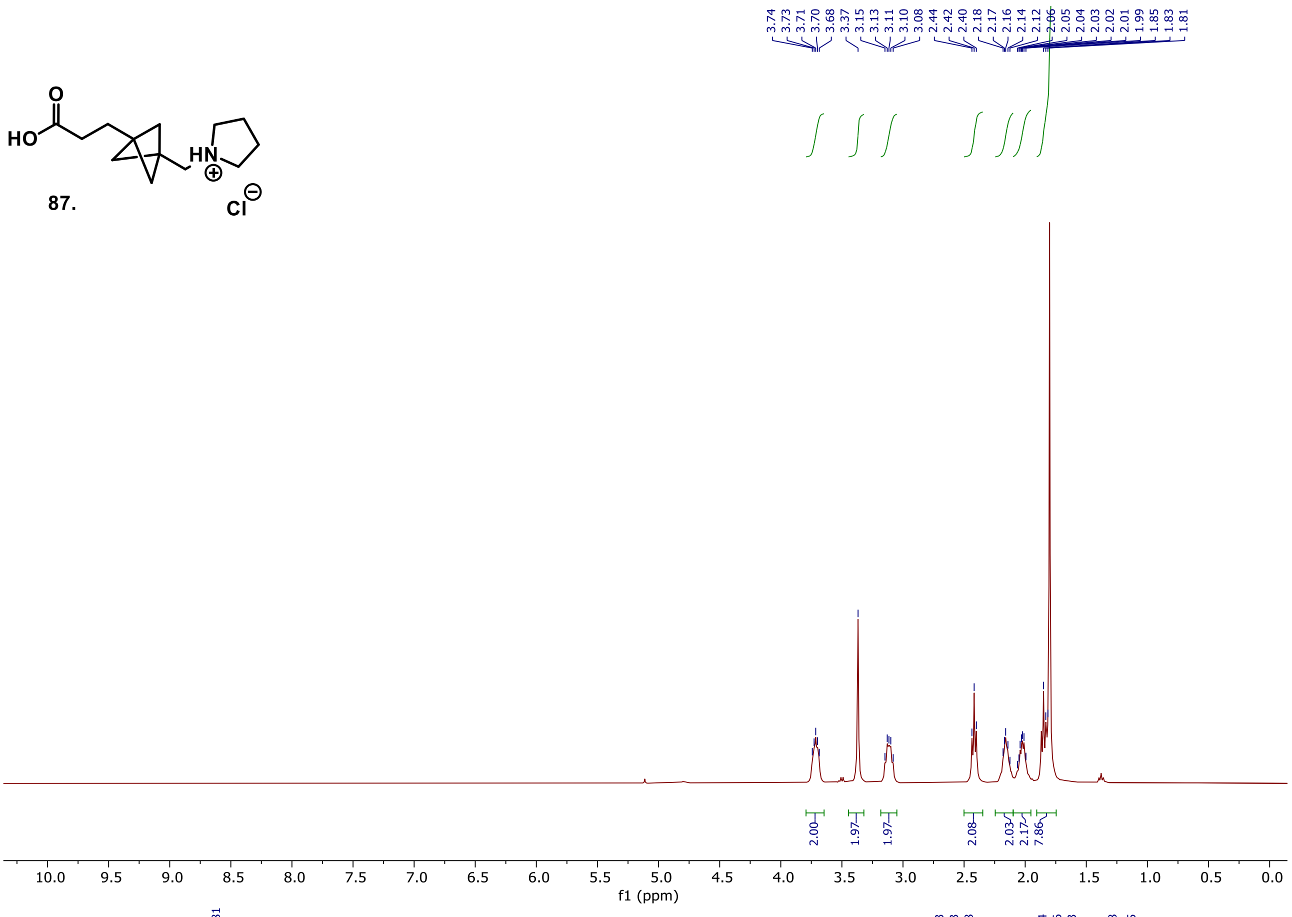

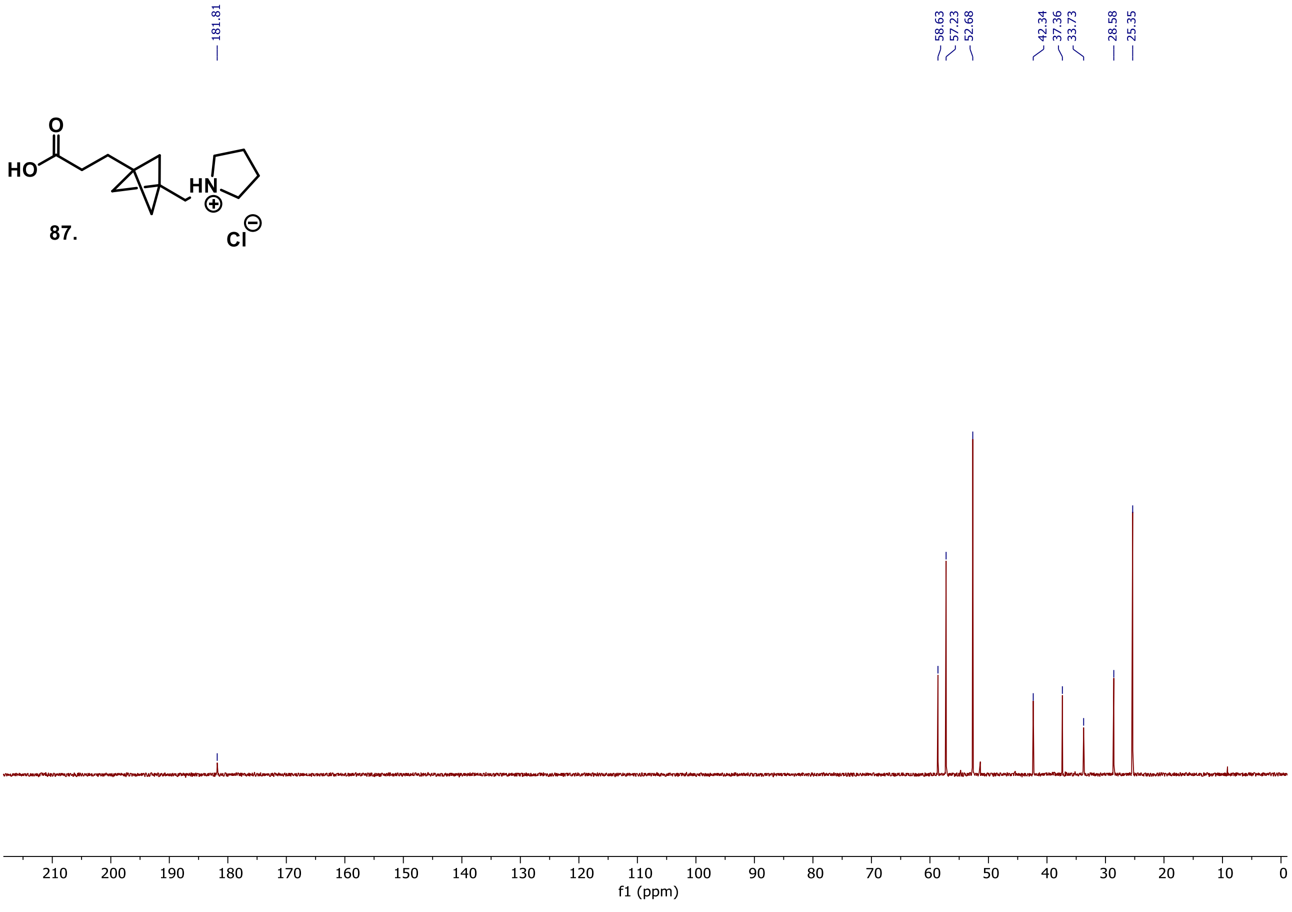

88: ^{1}H NMR (400 MHz, D_2O) and **^{13}C NMR** (126 MHz, D_2O) respectively.

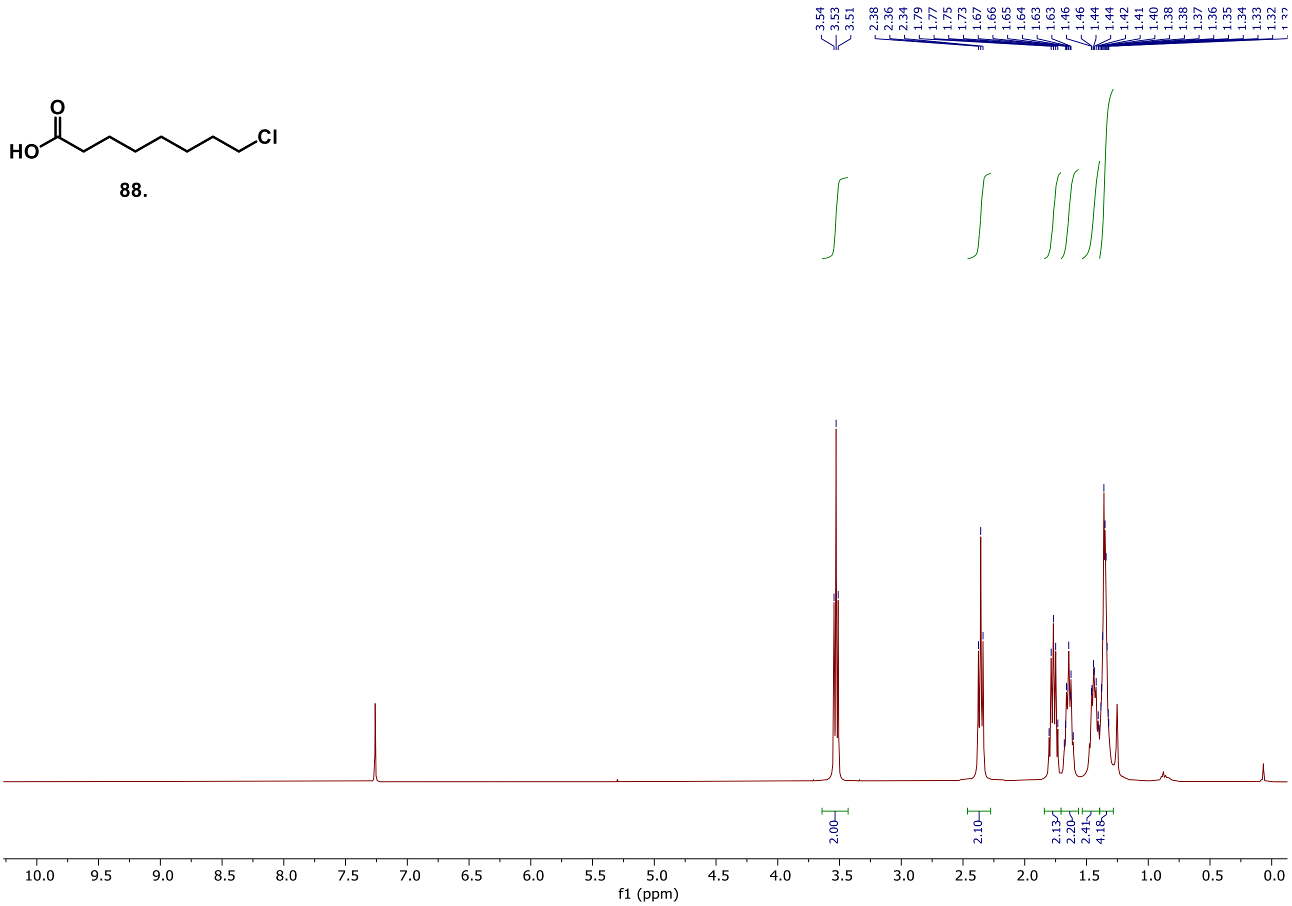

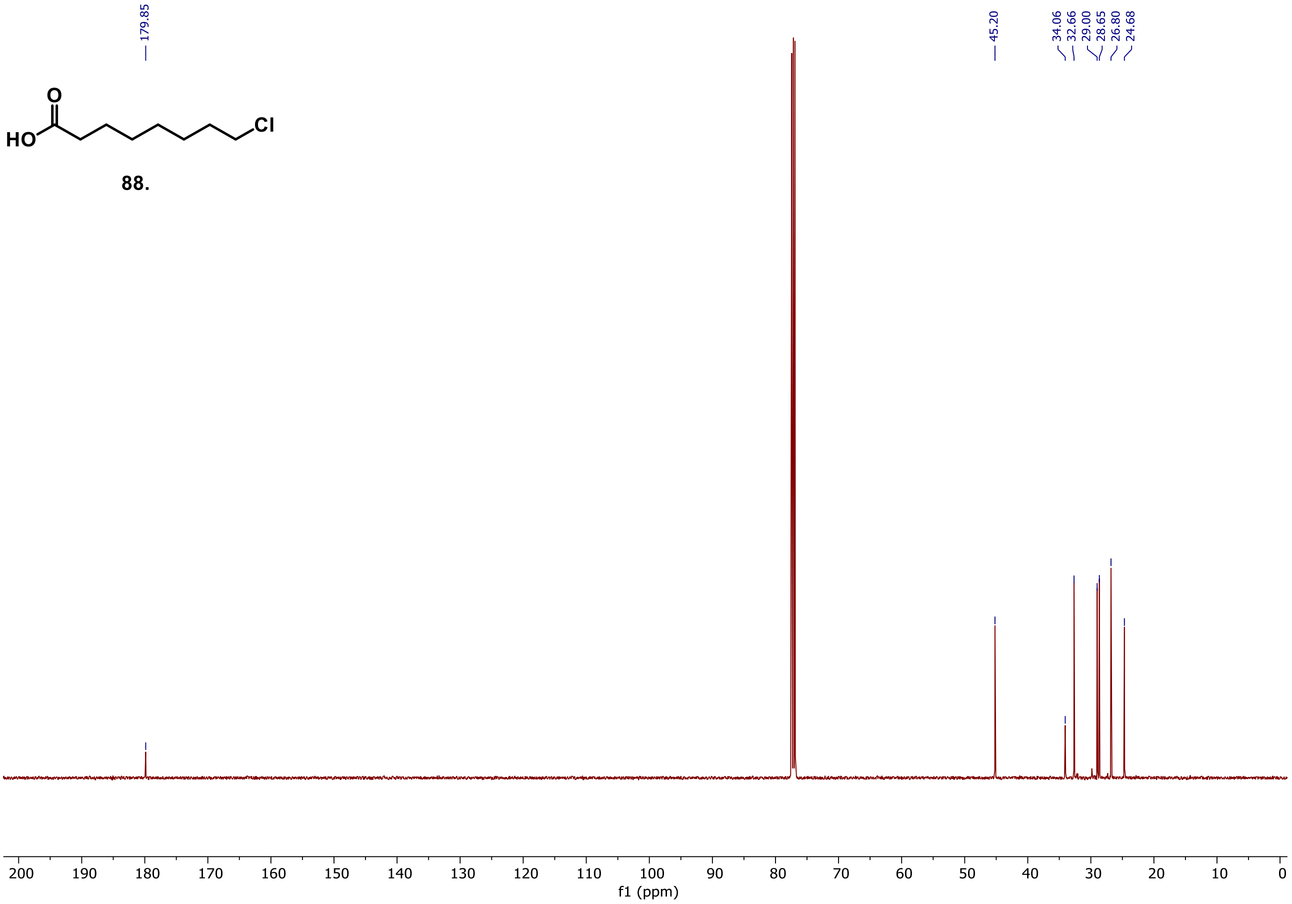

89: ^{1}H NMR (400 MHz, D_2O) and **^{13}C NMR** (126 MHz, D_2O) respectively.

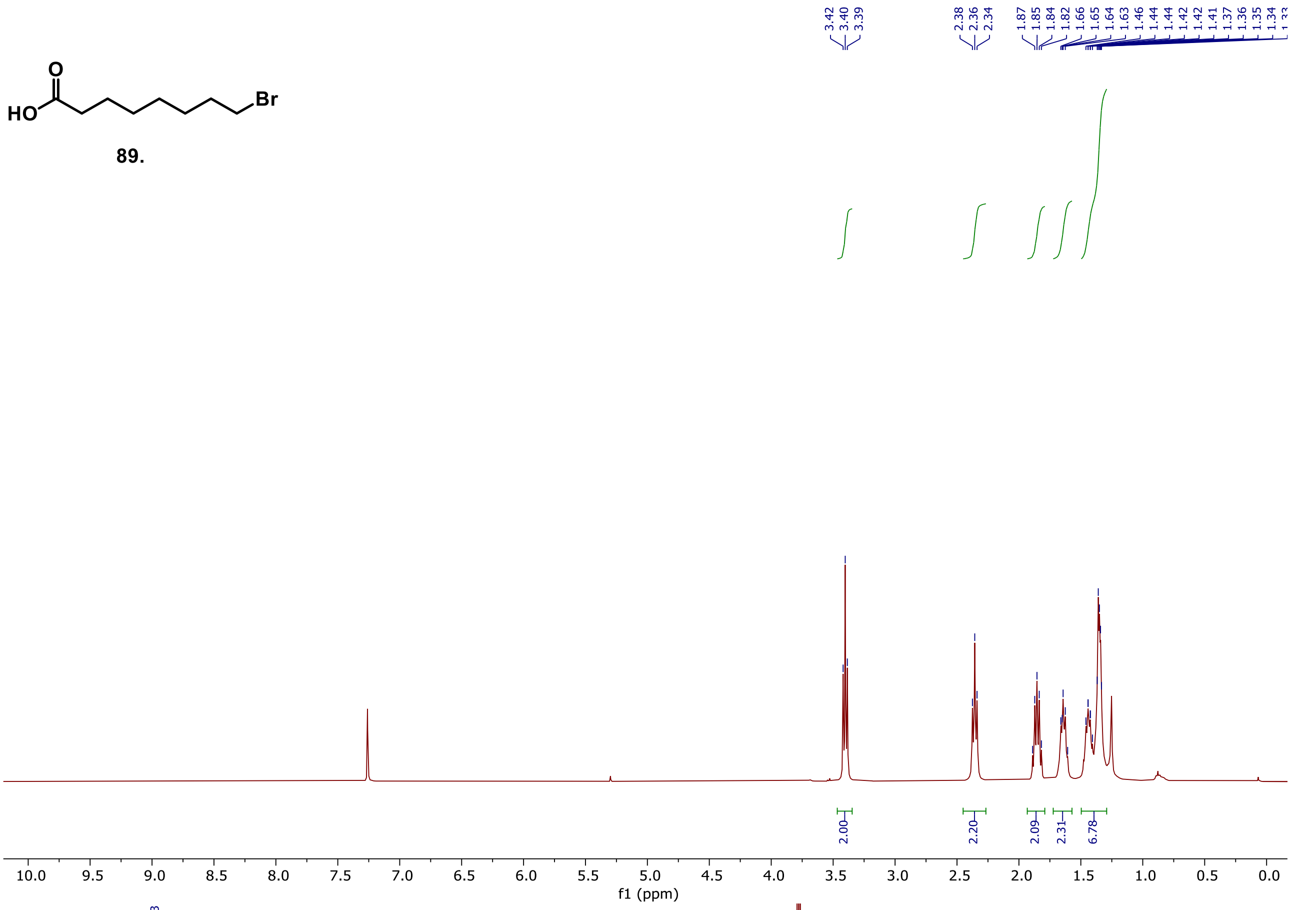

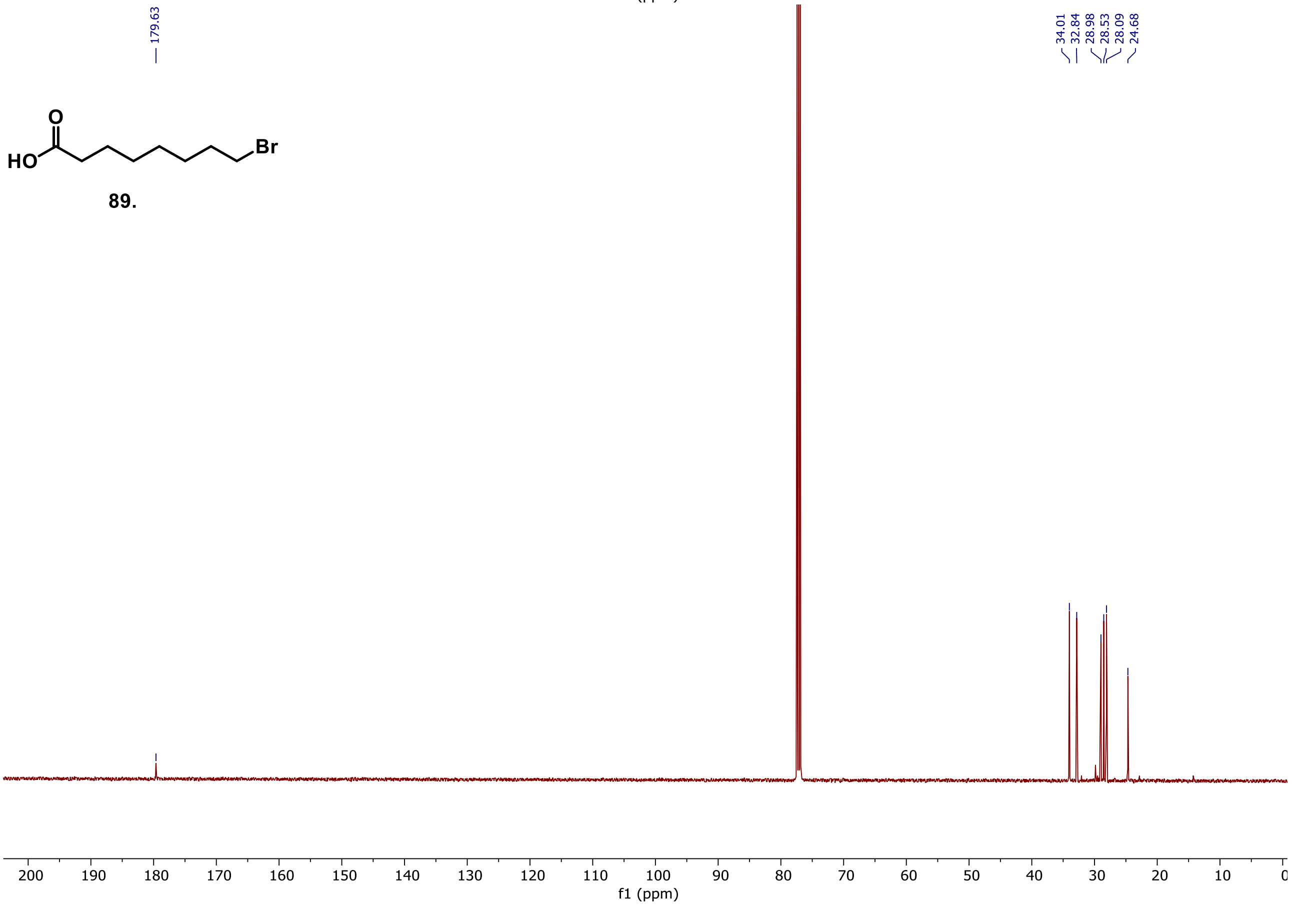

90: ^{1}**H NMR** (400 MHz, D_2O) and ^{13}**C NMR** (126 MHz, D_2O) respectively.

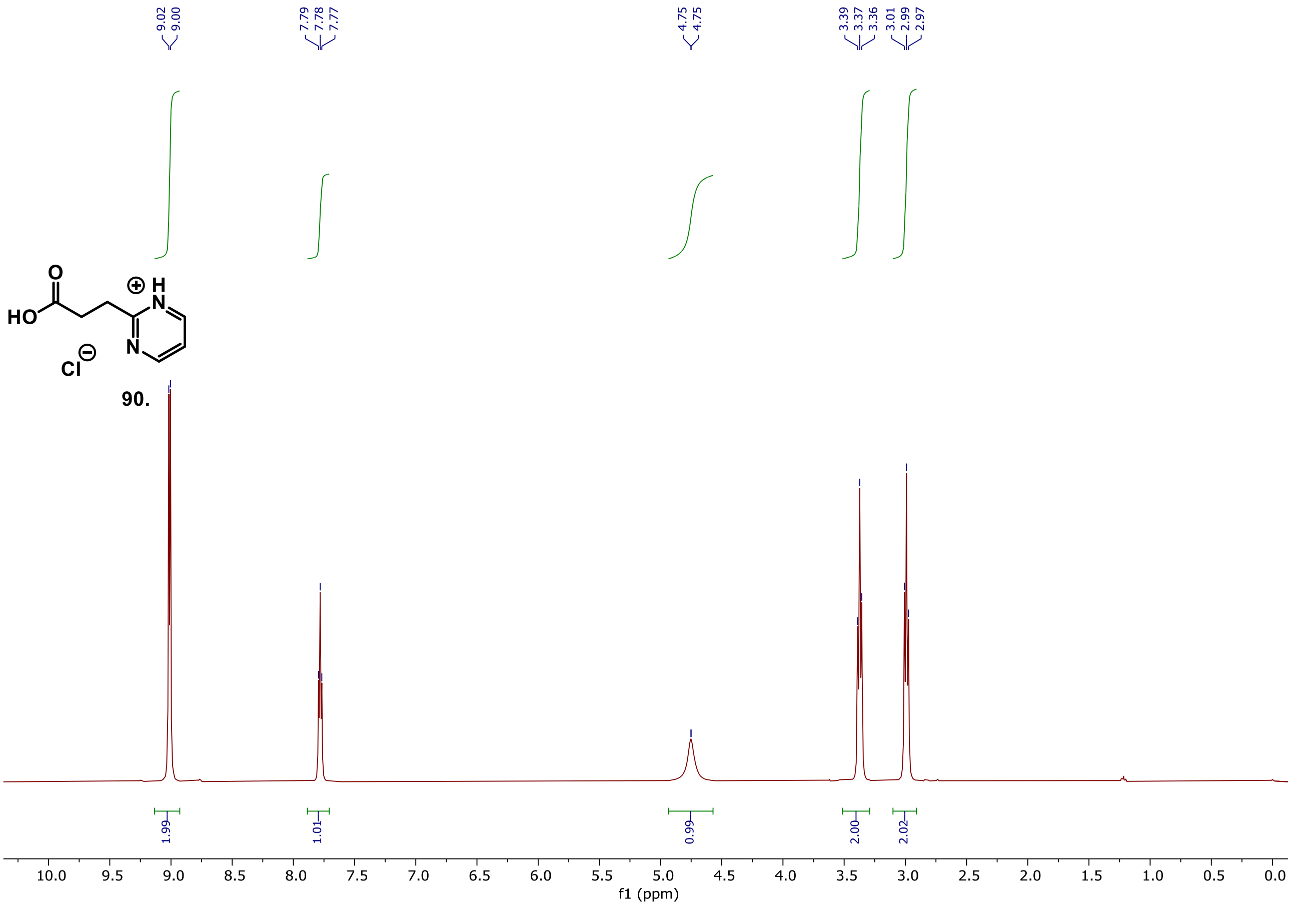

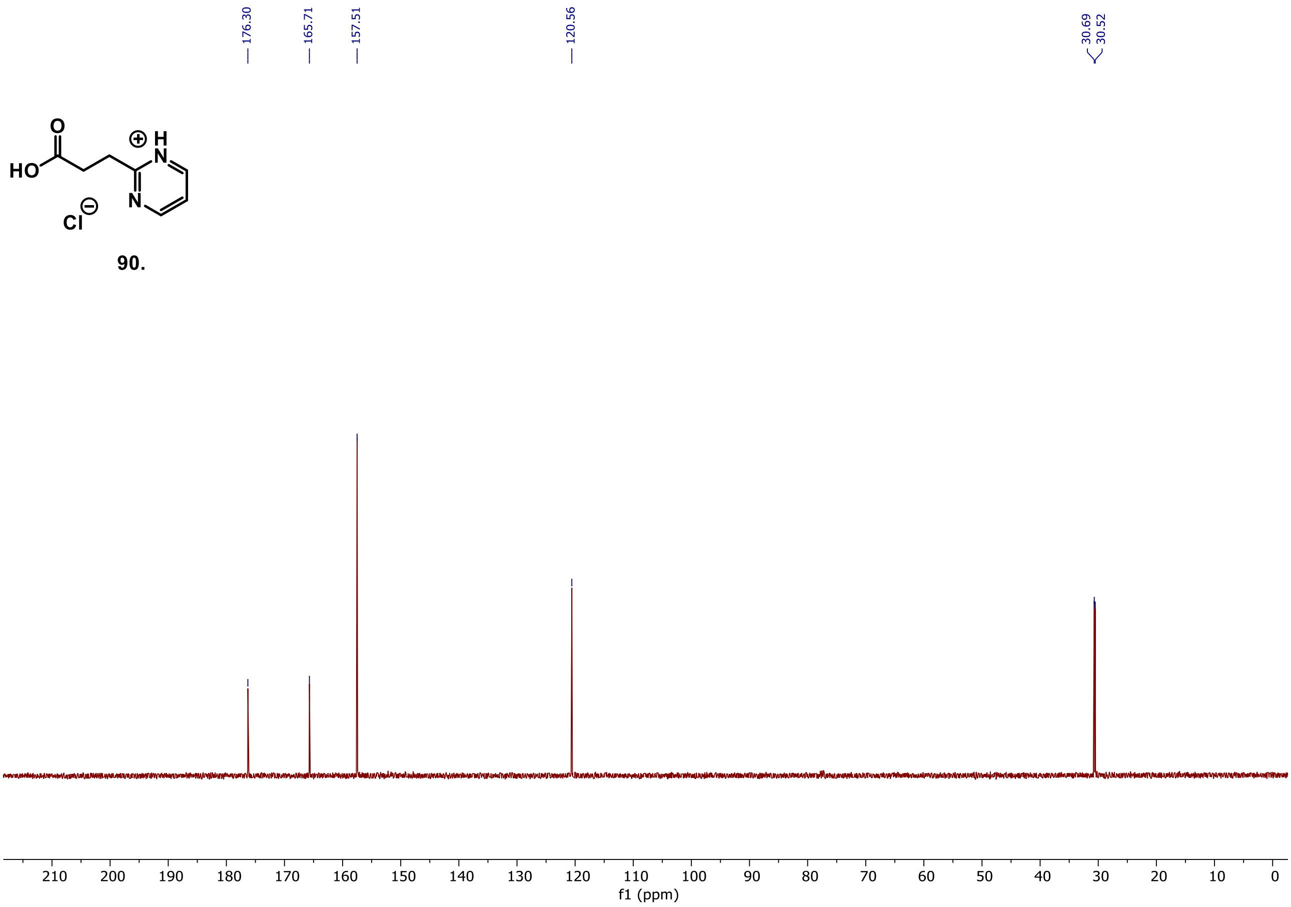

91: ^{1}H NMR (400 MHz, D_2O) and **^{13}C NMR** (126 MHz, D_2O) respectively.

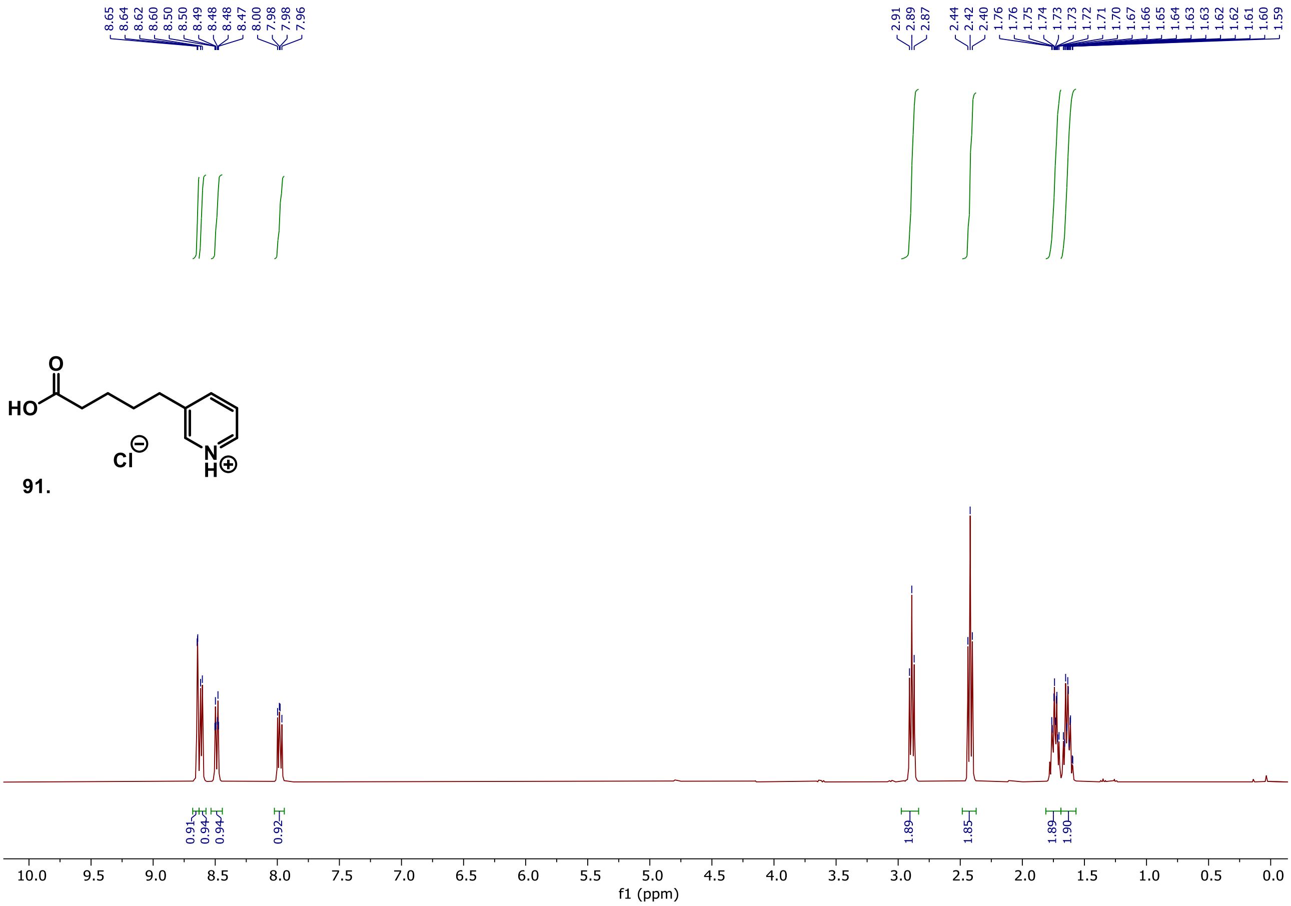

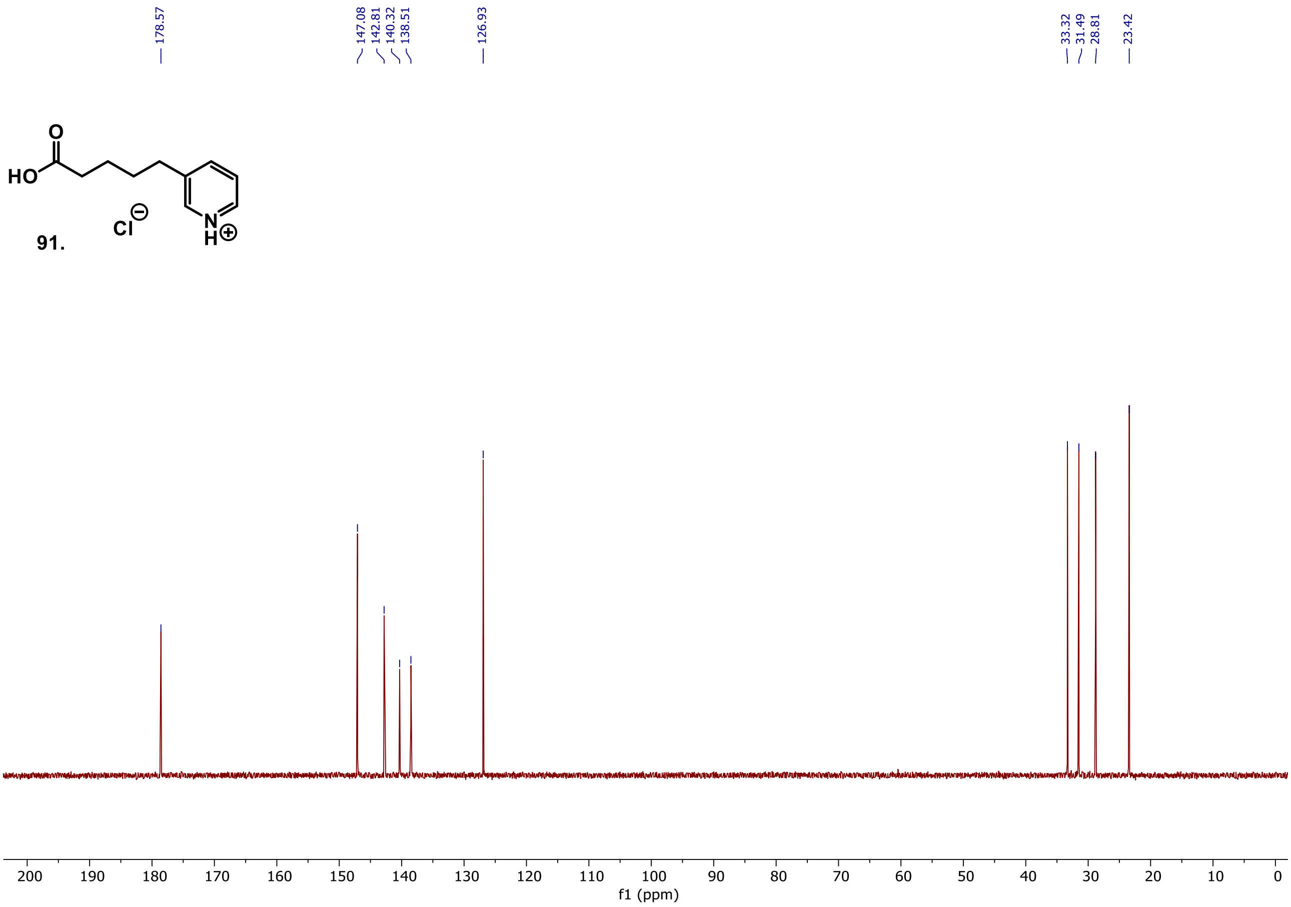

92: ^{1}**H NMR** (400 MHz, D_2O) and ^{13}**C NMR** (101 MHz, D_2O) respectively.

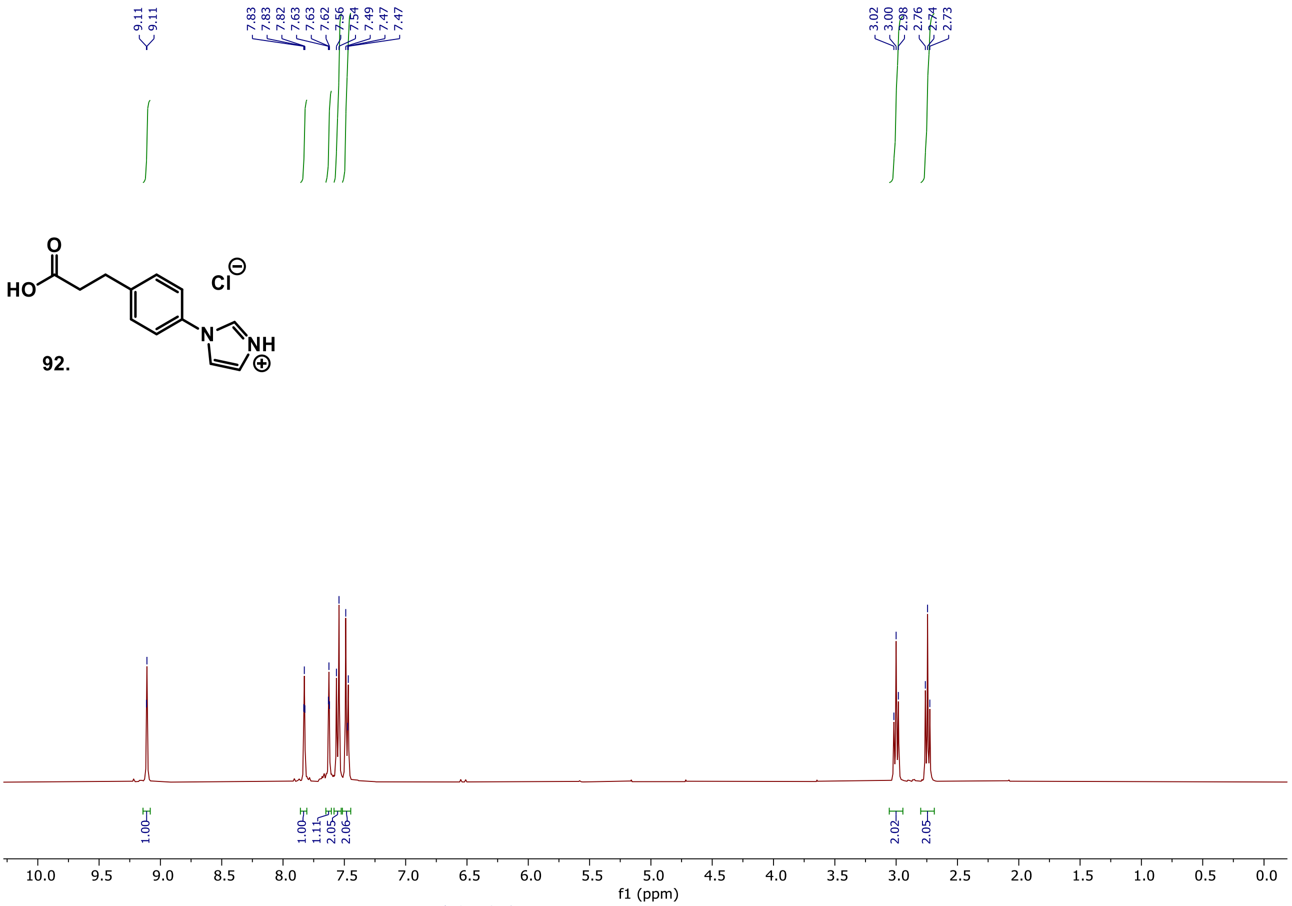

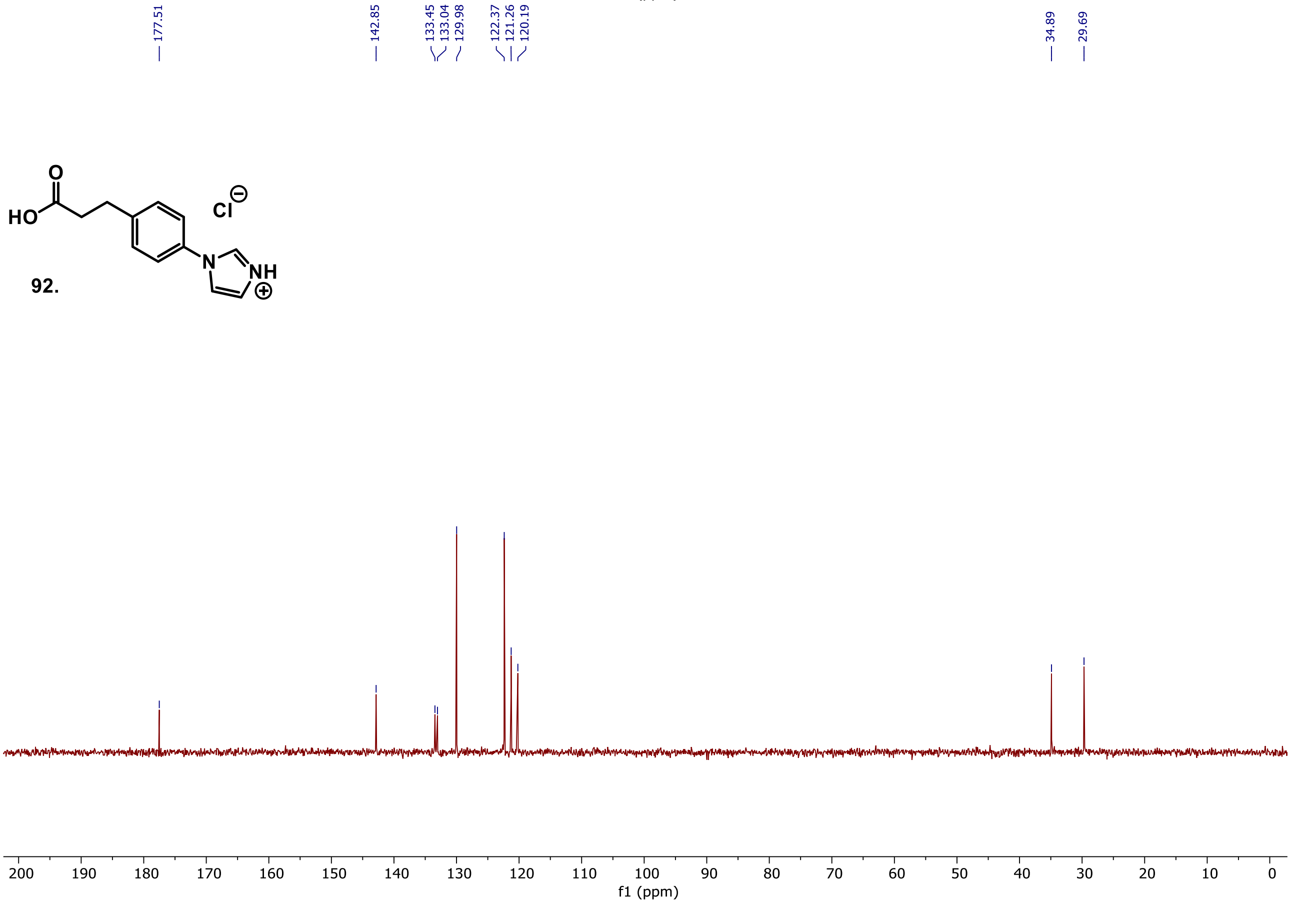

93: ¹H NMR (400 MHz, $CDCl_3$) and **¹³C NMR** (101 MHz, $CDCl_3$) respectively.

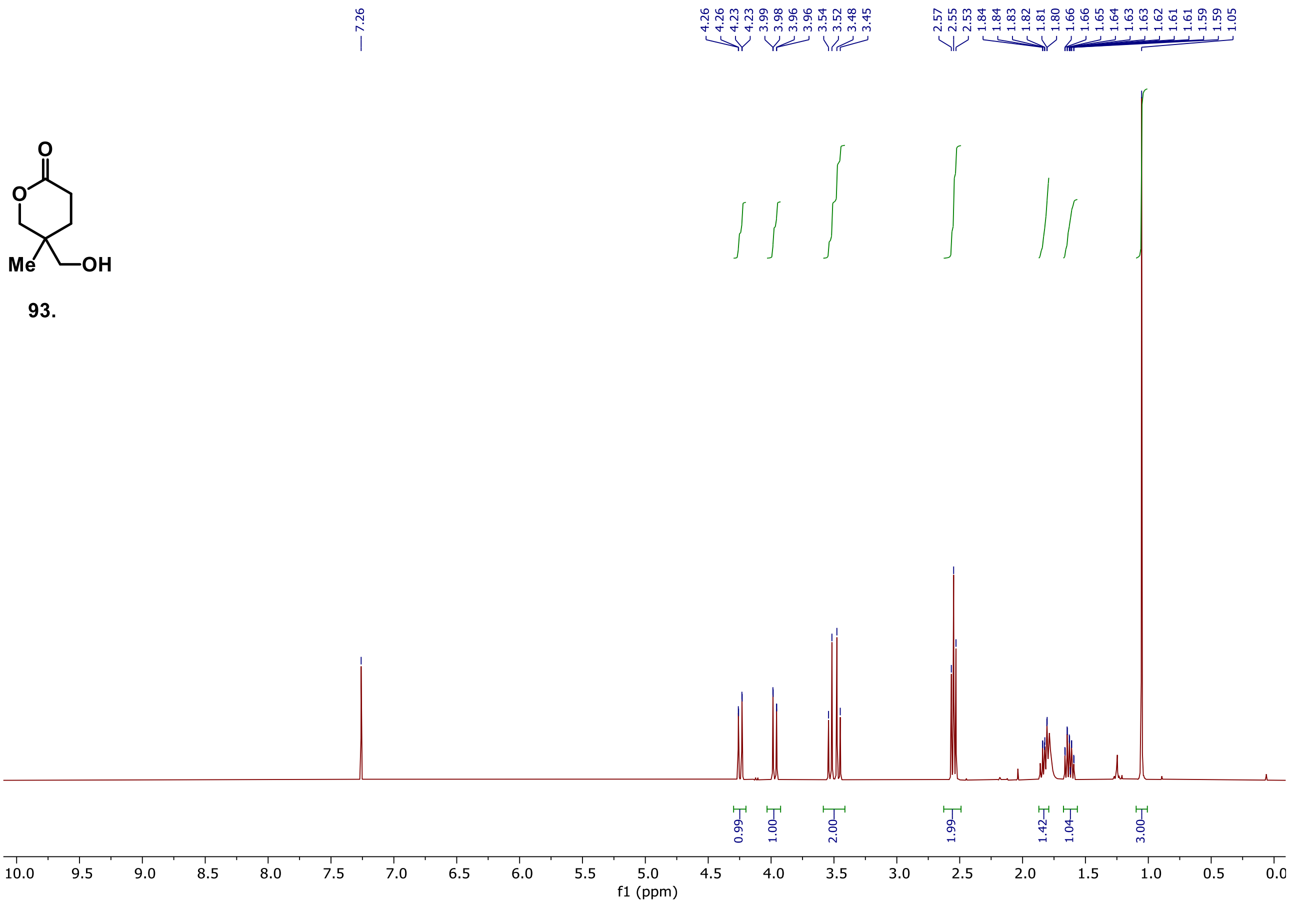

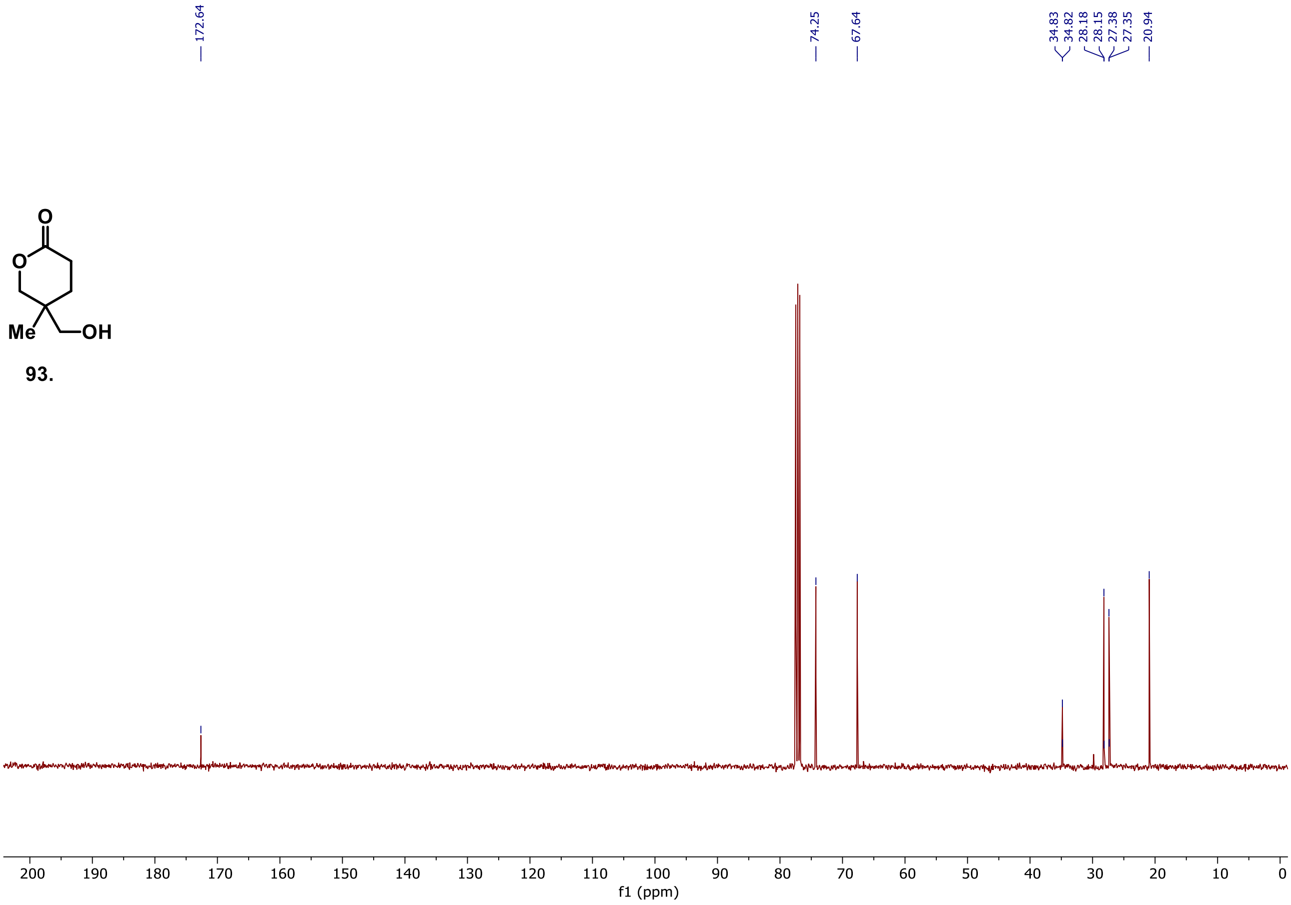

94 (or P2): **^{1}H NMR** (400 MHz, $CDCl_3$) and **^{13}C NMR** (101 MHz, $CDCl_3$) respectively.

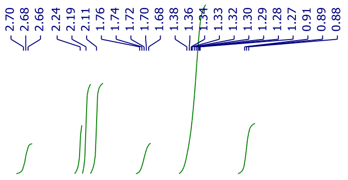

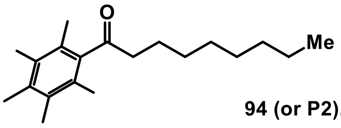

94 (or P2).

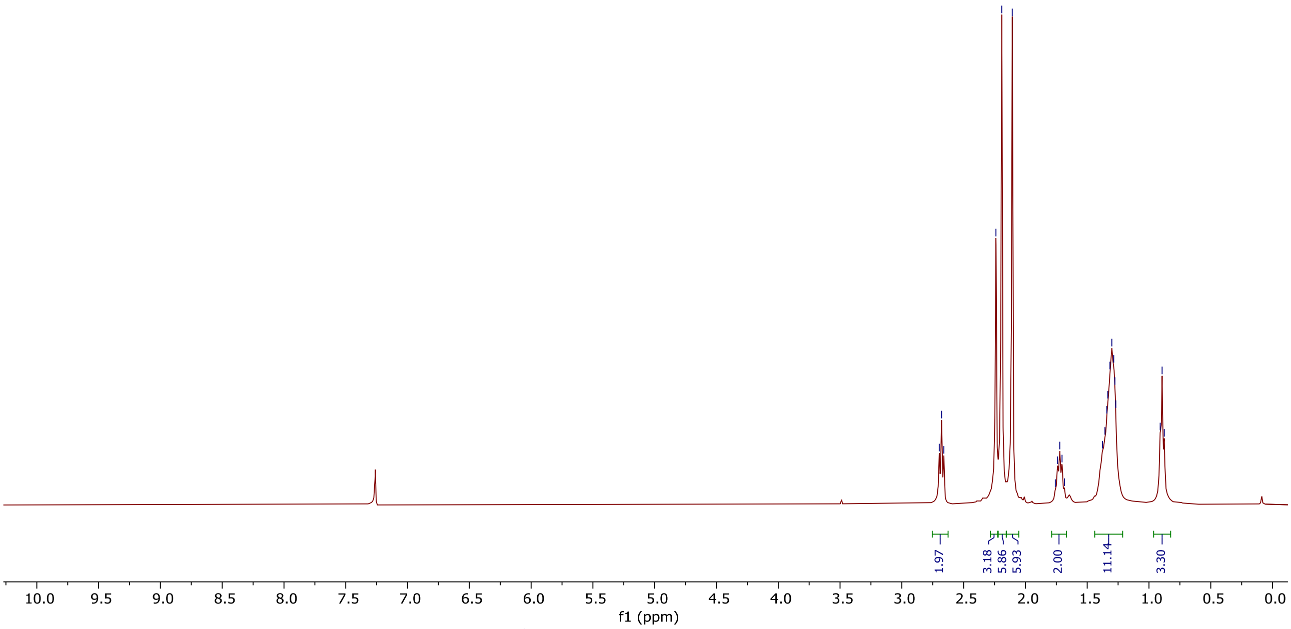

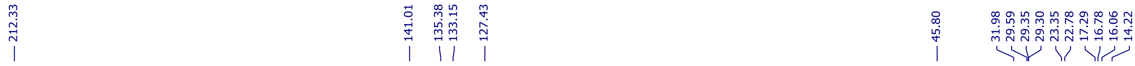

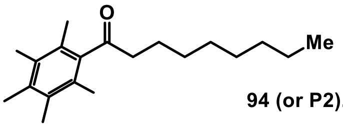

94 (or P2).

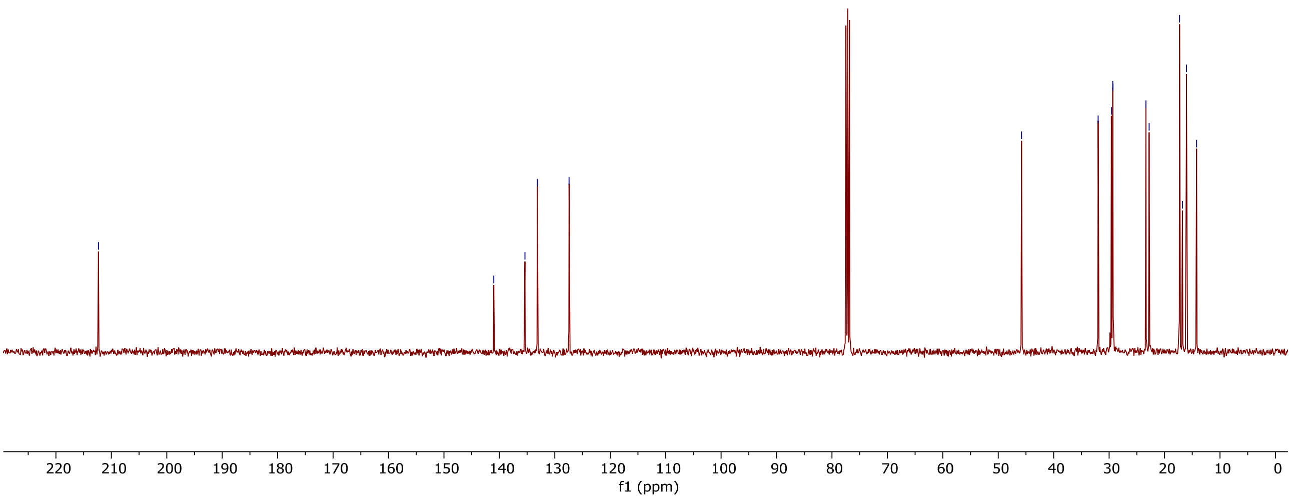

Supplementary References

1. Enyong, A. B. & Moasser, B. Ruthenium-catalyzed N-alkylation of amines with alcohols under mild conditions using the borrowing hydrogen methodology. *J. Org. Chem.* **79**, 7553–7563 (2014).
2. Quintard, A., Constantieux, T. & Rodriguez, J. An iron/amine-catalyzed cascade process for the enantioselective functionalization of allylic alcohols. *Angew. Chemie Int. Ed.* **52**, 12883–12887 (2013).
3. Reed-Berendt, B. G., Polidano, K. & Morrill, L. C. Recent advances in homogeneous borrowing hydrogen catalysis using earth-abundant first row transition metals. *Org. Biomol. Chem.* **17**, 1595–1607 (2019).
4. Corma, A., Navas, J. & Sabater, M. J. Advances in One-Pot Synthesis through Borrowing Hydrogen Catalysis. *Chem. Rev.* **118**, 1410–1459 (2018).
5. Frost, J. R. *et al.* Strategic Application and Transformation of ortho-Disubstituted Phenyl and Cyclopropyl Ketones to Expand the Scope of Hydrogen Borrowing Catalysis. *J. Am. Chem. Soc.* **137**, 15664–15667 (2015).
6. Akhtar, W. M., Armstrong, R. J., Frost, J. R., Stevenson, N. G. & Donohoe, T. J. Stereoselective Synthesis of Cyclohexanes via an Iridium Catalyzed (5 + 1) Annulation Strategy. *J. Am. Chem. Soc.* **140**, 11916–11920 (2018).
7. Frankowski, K. J., Golden, J. E., Zeng, Y., Lei, Y. & Aubé, J. Syntheses of the Stemona alkaloids (±)-stenine, (±)- neostenine, and (±)-13-epineostenine using a stereodivergent Diels-Alder/azido-Schmidt reaction. *J. Am. Chem. Soc.* **130**, 6018–6024 (2008).
8. Hall, C. J. J., Goundry, W. R. F. & Donohoe, T. J. Hydrogen-Borrowing Alkylation of 1,2-Amino Alcohols in the Synthesis of Enantioenriched γ-Aminobutyric Acids. *Angew. Chemie* **133**, 7057–7061 (2021).
9. Schierle, S. *et al.* Design and Structural Optimization of Dual FXR/PPARδActivators. *J. Med. Chem.* **63**, 8369–8379 (2020).
10. Porter, J. D. *et al.* An anthrone-based Kv7.2/7.3 channel blocker with improved properties for the investigation of psychiatric and neurodegenerative disorders. *Bioorganic Med. Chem. Lett.* **29**, 126681 (2019).
11. Heinisch, G. Synthesen und Reaktionen von Pyridazinderivaten, 2. Mitt.: 4-Hydroxymethyl-pyridazin. *Monatshefte für Chemie* **104**, 1354–1359 (1973).
12. Akhtar, W. M. *et al.* Hydrogen Borrowing Catalysis with Secondary Alcohols: A New Route for the Generation of β-Branched Carbonyl Compounds. *J. Am. Chem. Soc.* **139**, 2577–2580 (2017).
13. Hooper, J. F., Young, R. D., Weller, A. S. & Willis, M. C. Traceless chelation-controlled rhodium-catalyzed intermolecular alkene and alkyne hydroacylation. *Chem. - A Eur. J.* **19**, 3125–3130 (2013).
14. Schmidt, A. K. C. & Stark, C. B. W. TPAP-catalyzed direct oxidation of primary alcohols to carboxylic acids through stabilized aldehyde hydrates. *Org. Lett.* **13**, 4164–4167 (2011).
15. Ashush, N., Fallek, R., Fallek, A., Dobrovetsky, R. & Portnoy, M. Base- And Catalyst-Induced Orthogonal Site Selectivities in Acylation of Amphiphilic Diols. *Org. Lett.* **22**, 3749–3754 (2020).
16. Ramma Rao, A. V, Pulla Reddy, S. & Rajarathnam Reddy, E. Short and Efficient Syntheses of Coriolic Acid. *J. Org. Chem.* **51**, 4158–4159 (1986).
17. Chalyk, B. *et al.* Unexpected Isomerization of Oxetane-Carboxylic Acids. *Org. Lett.* **24**, 4722–4728 (2022).
18. Jiang, B., Feng, Y. & Ison, E. A. Mechanistic investigations of the iridium(III)-catalyzed aerobic oxidation of primary and secondary alcohols. *J. Am. Chem. Soc.* **130**, 14462–14464 (2008).
